# The direction, timing and demography of *Popillia japonica* (Coleoptera) invasion reconstructed using complete mitochondrial genomes

**DOI:** 10.1038/s41598-024-57667-x

**Published:** 2024-03-26

**Authors:** Francesco Nardi, Sara Boschi, Rebecca Funari, Claudio Cucini, Elena Cardaioli, Daniel Potter, Shin-Ichiro Asano, Duarte Toubarro, Michela Meier, Francesco Paoli, Antonio Carapelli, Francesco Frati

**Affiliations:** 1 Department of Life Sciences, University of Siena, via Aldo Moro 2, 53100 Siena, Italy; 2 NBFC, National Biodiversity Future Center, Palermo, Italy; 3 BAT-Center, Interuniversity Center for Studies on Bioinspired Agro‐Environmental Technology, Portici, Italy; 4 Department of Entomology, University of Kentucky, Lexington, USA; 5 Research Faculty of Agriculture, Hokkaido University, Sapporo, Japan; 6 Biotechnology Centre of Azores, University of the Azores, Ponta Delgada, Portugal; 7 Servizio fitosanitario cantonale, Dipartimento delle finanze e dell’economia, Bellinzona, Switzerland; 8 Council for Agricultural Research and Agricultural Economy Analysis (CREA), Florence, Italy

**Keywords:** Genetic variation, Evolutionary genetics

## Abstract

The Japanese beetle *Popillia japonica* is a pest insect that feeds on hundreds of species of wild and cultivated plants including important fruit, vegetable, and field crops. Native to Japan, the pest has invaded large areas of the USA, Canada, the Azores (Portugal), Italy, and Ticino (Switzerland), and it is considered a priority for control in the European Union. We determined the complete mitochondrial genome sequence in 86 individuals covering the entire distribution of the species. Phylogenetic analysis supports a major division between South Japan and Central/North Japan, with invasive samples coming from the latter. The origin of invasive USA samples is incompatible, in terms of the timing of the event, with a single introduction, with multiple Japanese lineages having been introduced and one accounting for most of the population expansion locally. The origin of the two invasive European populations is compatible with two different invasions followed by minimal differentiation locally. Population analyses provide the possibility to estimate the rate of sequence change from the data and to date major invasion events. Demographic analysis identifies a population expansion followed by a period of contraction prior to the invasion. The present study adds a time and demographic dimension to available reconstructions.

## Introduction

The Japanese beetle *Popillia japonica* Newman, 1841 (Coleoptera: Scarabaeidae: Rutelinae) is an invasive pest of significant interest due to its capacity to cause serious injury to multiple wild and cultivated plant species^1,2^. Larvae live in the soil and feed on roots, mainly of grasses, causing damage to pastures, lawns, and sport tuft. After emergence in late spring, adults attack leaves, flowers and fruits, including species of economic importance such as grape, corn and soybean^1,2^.

The European Commission has listed *P. japonica* within priority pests in 2019.

The genus *Popillia* Serville, 1825 includes 324 species^3^, with an Indo-African distribution. In the oriental and palearctic region, 134 species are known^4^. The species *P. japonica* originated in Japan, where it is nevertheless regarded as a pest of minor importance^5^. It was reported as common but not abundant in the islands of Kyushu, Shikoku and southern Honshu, and abundant in northern Honshu and Hokkaido^5,6^ where it finds a suitable habitat, although with low population densities compared to the invasive populations in the USA^5^. It is listed as present in all four major islands of Japan^7^.

The species was first reported in North America in 1916^5^ near Philadelphia (PA, USA). Contemporary investigations (reconstructed in^8^) tracked down the introduction to a shipment of iris bulbs from Japan into a renowned plant nursery in the town of Riverton, NJ six years before (i.e., ~ 1910), suggesting that *P. japonica* grubs may have been travelling undiscovered among iris roots. By 1920, the insect was present in the area in millions^8^ and estimates for the peak year 1929 were of half a billion insects per square mile^9^. Expansion westward continued in the following decades to cover the central lowlands in northeastern USA^2,10^ up to the Rocky Mountains, a natural barrier to *P. japonica* due to dry climate. A number of local outbreaks have been registered in the last half century in western USA (i.e., California, Colorado, Oregon, Wyoming) as a likely consequence of human-mediated long-range introductions. Localized outbreaks have been eradicated or attempts are ongoing to do so. The origin of such outbreaks is of major interest per se, and has been studied using different methods, including isotopic signatures^11^. As of 2021, *P. japonica* is listed as present with partial infestation in 9 states and present with a general infestation in 27 states^7^. The first record in the neighboring South-Eastern Canada dates back to 1944 in Nova Scotia (a slightly earlier date, 1939, is reported on the Canadian Food and Inspection Agency website^12^), where the pest would eventually become established in southern Ontario and Quebec. *P. japonica* was further reported on the West coast in 2017 in British Columbia^1,2,5^. It is currently listed as present, with restricted distribution, in 5 Canadian provinces, with localized infestations also present in British Columbia^7^.

The species was further introduced in the Azores (Portugal) where it was first reported in the 1970s in the island of Terceira as a likely introduction through the Lajes American military base^13^. The pest has been later reported from the islands of Faial, Sao Miguel, Pico, Flores and Sao Jorge, most recently in Graciosa^13,14^.

*P. japonica* was first observed in Italy in 2014^15,16^ in the Ticino Valley Natural Park, at a distance of ~ 10 km from both the Milano-Malpensa International Airport and the Cameri military airport. Starting from an initial 80 km^2^ in 2014, the infested area grew to exceed 800 km^2^ in 2017 and, as of 2021, a radius of 80 km from the original site is considered infested, with a further buffer zone of ~ 20 km (see^17^ for an interactive visualization of *P. japonica* distribution in northern Italy through time).

Following land contiguity, the pest was first detected at the Switzerland border in 2017 and is considered established in Southern Ticino since 2020^7,18,19^. Despite the currently limited distribution of the pest in Europe, a predictive study^20^ indicated that most Central Europe may have appropriate conditions for further spread of the pest, raising the utmost concern, whereas southern and northern countries may be at lower risk.

Reports from other countries (i.e., China, India, Korea, Taiwan, some northern European countries) are unconfirmed^7^ and may have arisen from false identifications^1^.

As such, the temporal series of introductions is as follows: Japan (original), USA (~ 1910), Canada (~ 1940), Azores (~ 1970), Italy (~ 2014), Ticino (~ 2017). The direction of introduction from Japan to the USA is certain, as the pest was present only in Japan at the time. The origin of the Canadian population from the USA and of the Ticino population from Italy are very plausible given the geographic contiguity and the timing of the expansion, but lack confirmation from genetic analyses. Key points of uncertainty are the origin of the Azorean population and the origin of the Italian population either as two independent or sequential events, as an example of a bridgehead effect^21^. The first has been hypothesized to originate from the USA given the presence of a large US military base at the site of introduction^5,13,22^. The latter may also be hypothesized to originate from the US through air transportation given haplotype sharing and the presence of two large airports in the area of the initial introduction^23^.

Available genetic and genomic data on *P. japonica* consist in the complete mitochondrial genome, a draft of the nuclear genome, microsatellite loci and mitochondrial haplotypes. The complete sequence of the mitochondrial genome (NC_038115^24^) was derived from an individual sampled in 2017 in the region of Ottawa, South-Eastern Canada, and as such it belongs to the USA/Canada invasive population. The current reference genome was produced by Canada's Genomic Enterprise (https://www.cgen.ca; unpublished) in the context of the CanSeq150 initiative and made available in 2019 as BioProject PRJNA514809. The original material is a male from Vancouver in South-Western Canada, that also belongs to the USA/Canada invasive population. Furthermore, the gut microbiome has been extensively studied^25^ in specimens from Oleggio (Italy, Milan area) collected in 2017, which are therefore representative of the Italian invasive population. The only data produced that includes representatives from the entire geographic distribution, targeting the reconstruction of the invasion, are microsatellite loci and mitochondrial haplotypes studied in^23^.

Starting with the analysis of mitochondrial haplotypes^26^, complete mitochondrial genomes have been sequenced in different species (reviewed in^27^) to study intraspecific relationships^28^, invasion processes^29^, speciation^30^, phylogeography and demography^31–33^. Compared to other molecular markers for population studies (i.e., microsatellites, single nucleotide polymorphisms, see^34^), complete mitochondrial genomes have both strengths and weaknesses^35^. As opposed to microsatellites and nuclear single nucleotide polymorphisms (SNPs), the mitochondrial genome is inherited as a single locus and therefore provides a very partial representation of genome level processes. At the same time, microsatellite and SNP variants are scored based on their presence/absence only, with limited to no possibility to quantify evolutionary differences among alleles, while relationships among mitochondrial haplotypes can be exploited extensively in terms of the genealogy of variants (i.e., applying a phylogenetic approach) and the timing of splitting events (i.e., coalescence models, molecular dating). As such, the two methods can be seen as complementary and liable to shed light on different aspects of a population history.

## Results

### Reference mitochondrial genome

Sequencing of the complete mitochondrial genome in an individual from Cameri (Italy), here used as reference, produced a non-ambiguous circular sequence of 18,406 bp. Coverage was stable at ~ 150× for long reads, with 255 remapping reads of average length 17,866 bp. Illumina reads produced a reasonably uniform coverage of ~ 269×. Specifically, coverage was high (~ 180 to 600×) over most of the mitogenome but dropped to as low as 11 × in the final part of the area interested by the repeats (see below). GC content, MiniIon coverage and Illumina coverage are shown in Supplementary Fig. S1.

The newly determined sequence is identical in structure and very similar in primary sequence to the previously available sequence NC_038115 throughout the coding part of the mitogenome and the initial/final part of the control region. Differences in primary sequence are limited to 0.39% of nucleotides plus 3 short indels (TΨC arm of *trnG*, *ttnL*, non repeated part of the control region), well in the range expected for intraspecific variability^36^. On the other hand, the repeated portion of the control region differs in length and structure. Compared to a relatively simple repeat structure of NC_038115, with three short and two long repeats, the newly determined sequence includes 25 repetitions of an 81 bp unit followed by a partial repeat of 38 bp and two repetitions of a 134 bp unit separated by a 49 bp spacer. Repeats differ at most by 1nt substitution (in the 81 bp units) and 2nt substitutions (in the 134 bp units).

### Sequencing and dataset assembly

The complete mitochondrial genome was successfully obtained for 85 individuals (see Supplementary Table S1 for details). The geographic distribution of samples was reasonably uniform at the level of regions, with 21 mitogenomes from Japan, 22 from the USA + Canada, 20 from the Azores and 22 from Italy + Ticino. Within regions, sequencing efforts were subdivided among sampling sites aiming at a uniform representation within the region (Supplementary Table S1). While some parameter optimization was performed for trimming and variant filtering, the high coverage, low error rate and simple genetic structure (i.e., a ploidy of 1) make the system very resistant to analytical biases, as evident after direct visualization of mapping and variant calling results in IGV. With the exception of the repeated region, coverage was over 100× in all mitogenomes and all gene regions, well above the threshold (DP > 10) used for variant filtering throughout (Supplementary Fig. S2). On the other hand, remapping over the region interested by the repeats was problematic, with all reads accumulating in the initial repeats, and this area was therefore removed from the analysis.

Following the addition of NCBI sequences and removal of the repeats region, the final datasets included 86 sequences of 16,031 aligned positions (*P. japonica* only; Supplementary Data S1) and 88 sequences of 16,085 positions (including outgroups; see “Methods”). Considering that all N-masked stretches fall within the repeated region, removal of the latter produced datasets that are complete and fully resolved. Ka/Ks estimates (0.0076–0.097 in individual PCGs; 0.0304 in concatenated PCGs) as well as the Fisher exact test, that did not identify directional selection among any sequence pair in the dataset in individual as well as concatenated PCGs, support the idea that no strong directional selection is at play (Supplementary Table S2).

### Population analyses

Collapsing of identical mitogenomes (*P. japonica* only, without outgroups) led to the identification of 37 unique sequences, with 28 being observed in one single individual and 9 in multiple (2 to 23) individuals. Sharing of haplotypes was most common within the same sampling location (4) or within different locations in the same region (4). Only one sequence was shared across regions, the unique haplotype found in the entire Italian + Ticino populations (22 occurrences) and occurring also in one individual from the Azores.

Overall, a stark decrease in variability (i.e., number of different mitogenomes, haplotype diversity and nucleotide diversity) was observed going from Japan to USA + Canada, to the Azores and to Italy + Ticino (Table. 1).

**Table 1:** Number of individuals, unique mitogenomes, haplotype diversity and nucleotide diversity per region.

Region	Individuals	Unique mitogenomes	Haplotype diversity	Nucleotide diversity
Japan	21	18	0.98571	0.01042
USA + Canada	23	14	0.91304	0.00267
Azores	20	5	0.63158	0.00009
Italy + Ticino	22	1	0	0

**Figure 1:**
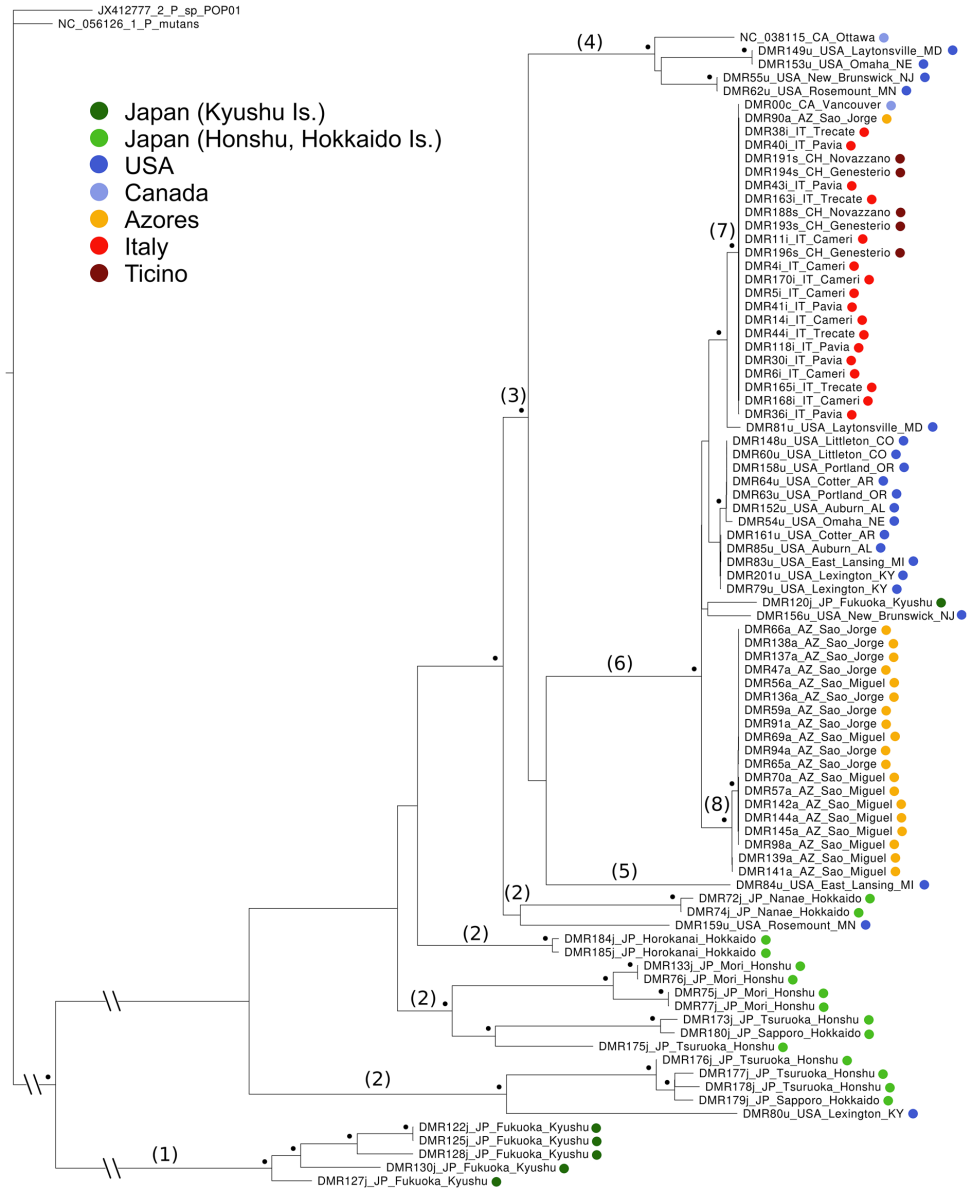
Phylogenetic tree of *Popillia japonica* mitochondrial genomes, with outgroups. Black dots indicate nodes with a bootstrap support over 95. Source of samples is color coded. Some nodes are marked with numbers that are reported in the text and other figures to ease cross referencing. Basal and outgroup nodes not to scale.

The phylogenetic tree of mitogenomes (Fig. 1) exhibits a basal bifurcation separating mitogenomes from the Southern Japanese island Kyushu (5 of the 6 sequences), indicated in figures as node (1), and the rest of the samples. Four additional nodes, indicated as (2), form a paraphyletic cluster that includes almost all sequences from the Central/Northern part of Japan (Honshu and Hokkaido islands), as well as two sequences from the USA (DMR159u, DMR80u). The next node (3) includes sixty-four sequences comprising all the individuals from the presumed invaded areas (with the exception of the two aforementioned sequences from USA and one sequence from Southern Japan: DMR120j). Within this latter, six mitogenomes from the USA diverge basally (nodes 4–5), whereas all others (fifteen from USA, all Azores, all Italy + Ticino) are grouped in a shallow nested node (6) that represents the major invasive group (see Discussion), characterized by a limited divergence. Within this latter group, all Italian + Ticino samples cluster together in node (7), with the inclusion of one individual from Vancouver (Canada: DMR00c) and one sample from Sao Jorge (Azores: DMR90a), whereas all other samples from the Azores cluster together in node (8). Notably, one mitogenome from Southern Japan (DMR120j) clusters within node (6).

ABC analysis produced simulated sets that encompass observed data, although the latter occupies a peripheral position among simulated sets. Scenario comparison based on the direct approach produced posterior probabilities of 0.55 (95% highest posterior density [HPD] 0.33 to 0.77) for a single introduction (i.e., Japan-to-USA, USA-to-the-Azores, Azores-to-Italy) and 0.45 (0.23 to 0.67) for a double introduction (i.e., Japan-to-USA, USA-to-the-Azores, USA-to-Italy). Based on the logistic approach, posterior probabilities were 0.91 (0.89 to 0.93) and 0.09 (0.07 to 0.11), respectively. Summarized over both scenarios, the rate of sequence evolution was 1.14E^−6^ substitutions per site per generation (s/s/g; 95%HPD 4.46E^−7^ to 2.31E^−6^), corresponding to 1.14 substitutions per site per million years (s/s/My).

**Figure 2:**
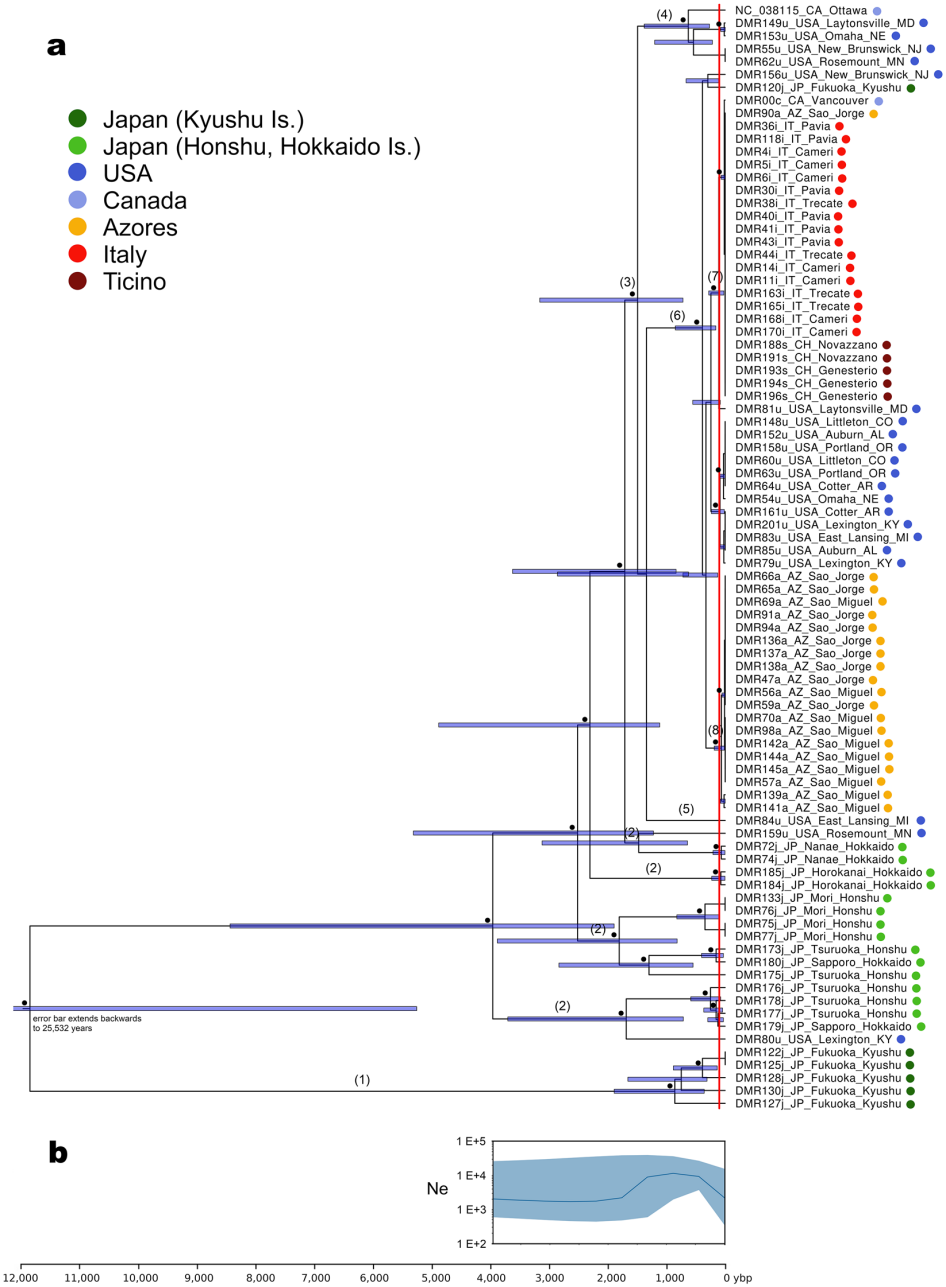
Dating and demography of *Popillia japonica.* Dated phylogenetic tree of *P. japonica* mitochondrial genomes (**a**). Black dots indicate nodes with a posterior probability over 0.98; red vertical line set at 112 ybp represents invasion to the USA; bars represent 95% HPD limits of nodal age. Source of samples is color coded. Some nodes are marked with numbers that are in turn reported in the text and other figures to ease cross referencing. Groups of identical haplotypes were reintroduced in the tree graphically to facilitate readability. Bayesian skyline plot (**b**) of Ne as a function of time before present. Both panels are on the same temporal scale.

The dating analysis (Fig. 2a) produced a tree with a topology identical (except for a short unsupported node within the node identified as (6) in Figs. 1 and 2) to the phylogenetic tree obtained based on Maximum Likelihood (see above). Date estimates, encompassing the last 25,000 years, are characterized by large confidence intervals (95%HPD interval grossly 1.5× node age) that, given the procedure applied, should account for the uncertainty in rate estimation in the ABC analysis coupled with the uncertainty in dating from the BEAST analysis. The basal separation between the Southern Japan group and all other samples was dated to 11,885 years before present (ybp; 95%HPD 5274–25,532). The Central-North Japan group differentiated (i.e., excluding root node) starting at 3972 ybp (95%HPD 1899–8465). Group (3), representing the bulk of invasive samples, differentiated starting at 1497 ybp (722–3170) and group (6), representing the major invasive lineage supposed to have differentiated within the USA (see below), at 389 ybp (159–852).

Comparison of minimal age (minimum of 95%HPD) of lineages against an introduction to the USA at 112 ybp identifies a minimum of nine mitochondrial lineages that were independently introduced from Japan to the USA [7 within node (3), 2 stemming from nodes identified as (2)]. Minimal relaxation of the 112 ybp threshold (i.e., assuming that group (6) differentiated within the USA) identifies 7 lineages. Based on minimal age, both nodes including invasive European samples are compatible with a local differentiation, with Azorean samples in node (8) differentiating at 65 ybp (8–187) and Italian + Ticino samples [node (7), inclusive of one Azorean and one Canadian] differentiating at 22 ybp (0–69).

The Bayesian skyline plot (Fig. 2b) resulted in an estimation of Ne through time between 1E^+3^ and 1E^+4^, with an ample margin of uncertainty. The temporal trend is of a stable Ne from 4000 to 2000 ybp, an increase of ~ 10× at 1000 ybp, and a slight decrease in the last 500 years. Noteworthy, this reconstruction focuses on the Central North Japan clade and worldwide invasion only, as South Japan samples were not included to minimize the confounding effects of population structure in the sample (see “Methods”). Somewhat counterintuitively, the population expansion following *P. japonica* invasion worldwide, possibly substantial, is not captured by the analysis as the process is too recent to have produced a sizable number of new mitogenome variants leading to new coalescence events in the tree. The number of individuals analyzed, alongside the limited variability observed outside Japan, did not allow conducting demographic analyses on individual areas, something that may be desirable in the future.

## Discussion

The analyses presented in this study allowed us to reconstruct the details of the tempo and mode of the differentiation and colonization of *P. japonica*, confirming previous findings from the microsatellite and mitochondrial haplotype analyses presented in^23^ and complementing these with regards to the temporal patterns and demographic aspects of the invasion.

### Ancient history in Japan

**Figure 3:**
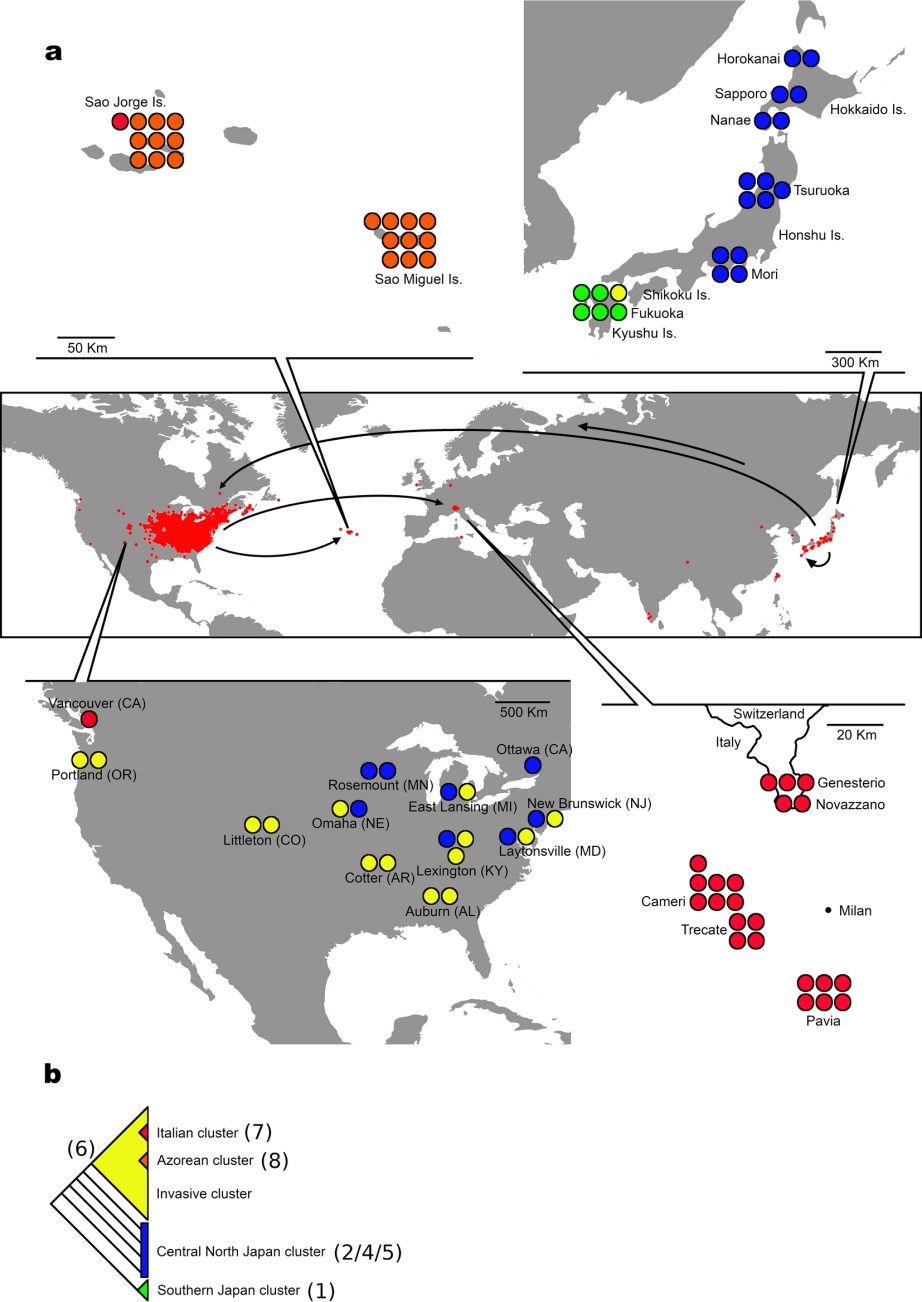
Distribution of *Popillia japonica* haplotype groups. Circles in (**a**) represent individuals, color coded according to haplotype group (see (**b**)). Small red dots in the World map represent the current distribution of the pest (based on^20^) and arrows indicate the hypothesized route of *P. japonica* serial introductions. Tree of color codes in panel b is a simplified version of Fig. 1. Some nodes are marked with numbers that are in turn reported in the text and other figures to ease cross referencing.

The basalmost separation is observed between South Japan and North-Central Japan mitogenomes (Figs. 1, 3). This well supported differentiation was dated at 5274–25,532 ybp (Fig. 2) and confirms previous observations based on microsatellites and mitochondrial haplotypes^23^. The split corresponds to the geographic division between the island of Kyushu in South Japan and the larger islands of Honshu and Hokkaido in North-Central Japan, with the two areas being separated by the Seto inland sea. The observation^5,6^ that the species is present but not frequent in the Southern islands, while abundant in Honshu and Hokkaido, coupled with the larger diversity observed in Honshu and Hokkaido, may suggest the Central-Northern islands as the location where the species originated, with Kyushu as a possible relict population that persisted in geographic isolation. Noticeably, the islands of Kyushu and Honshu were connected in a single non glaciated landmass during the last glaciation (~ 20,000 ybp; Fig. 8 in^37^), and the separation observed based on mitogenome analysis, dated at 5274–25,532 ybp, may be a result of the subsequent separation between the two and the post-glacial formation of the Seto inland sea. Only one individual sampled in Kyushu clusters with the invasive samples [node (6)], a fact that might be attributed to a backward secondary introduction from the USA to Kyushu, something that is not implausible given the high propensity of the species for man mediated long range invasions. This aspect will be addressed in more detail using nuclear SNP markers (in preparation).

Differentiation in the North-Central Japan cluster, strictly speaking a paraphyletic cluster, is represented by nodes marked as (2) in Figs. 1 and 2. These four basal nodes all include samples from Japan, plus one mitogenome from Kentucky and one from Minnesota. A geographic pattern emerges in the temporal distribution of nodes, with most of the samples from Honshu being basal and two clusters of samples from Hokkaido being sister to the node comprising all invasive non-Japanese samples. This phase of differentiation started at 1899–8465 ybp, and the demographic analysis identified a population expansion (10×) in the second half of this period. In line with the observed higher population densities currently observed in the northern islands, we envision the possibility of a radiation in Japan concurrent with a northward expansion to colonize areas that are climatically more suitable for the species. The identified population expansion finds external support in^23^ who observed negative Tajima’s D and Fu’s Fs overall and an excess of haplotypes in Central-Northern Japan.

### Introduction in North America

The geographic distribution of samples per se (Figs. 1, 3) would suggest, with equal reliance, that both node (3) and node [6; nested within (3)] may represent the major invasive event of the species to North America, and may have differentiated locally within the USA after the invasion. However, node (3) differentiated at 722–3170 ybp, hence substantially predating the reported introduction in the USA, historically dated at ~ 1910^5,8^. At variance, node (6) differentiated at 159–852 ybp. This figure, although strictly speaking predating the invasion, is arguably within a sufficiently recent time frame, in its minimal range, as to be deemed compatible with the historical dating. As such, based on both geographic distribution and dating, we favor the view that the group depicted as node (6) may mark the major event of introduction to the USA and be the outcome of a local differentiation within the USA post-invasion.

Compared to node (6), node (3) contains 6 additional North American samples (5 from USA: MD, NE, NJ, MN, MI; one from Canada: Ottawa), whose inferred splitting dates are much earlier than the introduction of *P. japonica* in the continent (~ 1910) and which may be the outcome of separate introductions. Noticeably, such a hypothesis was explicitly discussed also in^23^. Five of these sequences are clearly associated [node (4)], and might be the signature of a subsequent differentiation starting at 269–1384 ybp. The sixth individual is an isolated sample from Michigan, reported as the sister taxon of node (6). Time-wise, the origin and differentiation of these latter lineages is fairly old, suggesting that they may have diverged within Japan prior to the invasion. While the introduction of multiple lineages from Japan is well supported, the question remains open as whether this was the outcome of a single event of introduction, with multiple individuals carrying across multiple and different mitogenomes [as in the case of the individuals of node (4)], or multiple independent events of introduction, possibly non contemporary.

From the point of view of USA samples, quite a heterogeneous pattern emerges. The vast majority of the samples, belonging to node (6), seems to be part of the major invasion event, occurred at the beginning of the nineteenth century, while other sequences may represent the traces of additional events of independent introductions.

The geographic distribution of mitogenomes within the USA does not suggest the existence of a clear local population structuring, in line with the idea that expansion following an invasion bottleneck is not expected to create population structure in the short term. Nevertheless, the major invasive lineage (node 6, yellow dots in Fig. 3, USA map) includes samples from all the USA distribution, whereas other invasive lineages are restricted to the northeastern USA, the known site of the original introduction (blue dots in Fig. 3, USA map). As such, we envision the possibility that multiple lineages characterized the introduction and expansion in the northeastern USA, with only one subsequently giving rise to a substantial expansion and being responsible of all recent introductions in the USA, as well as in Europe.

Of major interest would be the possibility to identify the location within Japan from which the USA invasion originated. The phylogenetic analysis does not provide information for the origin of node (6), the major invasive group. Considering, in turn, node (3), and aiming at a broader-scale characterization of the source area, some additional information can be obtained. Both Japan nodes that are more closely related to node (3) include only mitogenomes from Hokkaido (locations Nanae and Horokanai), suggesting this as the original source of the USA invasion from northern Japan. Incidentally, this issue was specifically at stake in^23^, with microsatellites supporting an origin in East/Central-West Japan and mitochondrial haplotypes identifying a restricted region in central Honshu. However, in^23^ sampling in Hokkaido was limited to three individuals for mitochondrial haplotypes and none for microsatellites, and, as such, the results are not readily comparable. In summary, and acknowledging the limited sampling in Honshu (9 individuals from two sites) that may have led to an incomplete description of the mitogenomic variability in the island, while an origin in Central Honshu^23^ cannot be excluded given the limited geographic structure within Japan, an origin from Hokkaido appears to be more coherent with our analyses. Noteworthy, Hokkaido currently hosts the pest at high density^5,6^ and, if current density estimates can be extrapolated to ~ 1910, this makes it a plausible origin for the USA outbreak, although it is characterized by lower human population density and arguably a lower rate movement of people and goods towards the USA. Considering other islands as possible sources, our American samples have no direct relationship with Southern Japan (with the one exception of sample DMR120j), and therefore the island of Kyushu can be excluded as a source. Mitogenomes from Honshu, in turn, with location Tsuruoka being central to the area identified as source in^23^, while weakly related to the main invasive cluster (6), are related to one genome sampled in Kentucky, possibly marking a secondary introduction that did not lead to a significant expansion in the USA.

The number of samples in Canada is arguably limited. Nevertheless, both individuals carry mitogenomes that, while not being directly correlated to each other, are both related to USA samples. Based on this observation, as well as the geographic contiguity, it is reasonable to consider Canada as part of the USA expansion. This is in line with^23^ where 16 Canadian individuals were observed to harbor mitochondrial haplotypes that are well represented in the USA and microsatellite data associate the seven individuals studied, all from the East Coast, with the northeastern USA group. Worthy of note, the sample from Ottawa (southeastern Canada) belongs to one of the minor invasive clusters (node 4), and clusters with four northeastern USA individuals. The sample from Vancouver (southwestern Canada) is, in turn, related to the major invasive cluster that characterizes the bulk of the recent expansion in the USA, including the northwestern areas. This observation, as well as the timing of Canadian introductions (~ 1940 in the East and ~ 2017 in the West), give further support to an USA origin of Canadian samples in two distinct events of cross border expansion.

### Introduction in the Azores and mainland Europe

The recent introduction of *P. japonica* in the Azores (~ 1970) and northern Italy (~ 2014) marks the most recent phase of the expansion of this pest. Mitogenomes in both areas display a substantially reduced genetic variability, in line with expectations based on a recent introduction possibly entailing a significant population bottleneck at invasion (Table 1). All 22 samples from Italy + Ticino share a single mitogenome variant that is associated with one sample from the Azores and one from Vancouver, Canada, in a node dated at 0–69 ybp (identified as (7) in Figs. 1 and 2). Samples from the Azores, excluding the one aforementioned mitogenome, in turn share five different mitogenomes, characterized by minimal divergence (0–3 bp difference). With the one aforementioned exception, all samples from the Azores cluster in a monophyletic group dated at 8–187 ybp (identified as (8) in Figs. 1 and 2). Although dating, given the limited number of mutations observed at this level, may carry a significant uncertainty, both clusters are extremely recent and compatible with an initial differentiation subsequent to the invasion. Incidentally, one mitogenome was observed in an individual from Vancouver (Canada) that is almost identical (2 bp difference) to the dominant variant in Italy and Ticino. The observation that the population collected in Vancouver consists of a limited number of individuals detected in an urban area (David Holden, personal communication), as well as the relevant timeline, with appearance in Vancouver actually postdating that in Italy by 3 years, makes an introduction from Vancouver to Italy untenable. Nevertheless, it can be hypothesized that both introductions may stem from one and the same location within the USA where this variant may still be present. The identification of one closely related mitogenome in Laytonsville (MD, USA) may be suggestive in this respect.

Based on the phylogenetic tree, the Italian and Azorean clusters, although very similar, are not associated, with a number of intervening mitogenomes sampled in the USA. Nevertheless, at this shallow level of divergence the confounding effects of ancestral lineage sorting, exacerbated by population bottlenecks at invasion, cannot be overlooked. The ABC analysis, that explicitly addresses this possibility by modeling the mitogenome tree within a population tree, was used to test the two scenarios of a unique serial introduction from the USA to the Azores, and hence to Italy, versus two independent introductions. The third theoretical scenario, a serial introduction from the USA to Italy and hence to the Azores, was not considered as invasion to the Azores predates invasion to Italy by 40 to 50 years. The ABC analysis was inconclusive in this respect, although with a marginal preference for a single introduction in one of the analyses. Nevertheless, the ABC analysis is not expected to perform optimally and provide a solid resolution with only one locus; work is underway to repeat the analysis using multiple unlinked nuclear SNPs.

As for the source area, within the USA, of the two invasive groups, their belonging to node (6) links European samples to the main lineage that spread in the country, that, albeit present throughout the species’ range in the USA, is dominant in the southeastern areas at the border of the current distribution. Fine scale identification of the most closely related mitogenomes in the phylogenetic tree is inconclusive for the Azorean clade, whereas one mitogenome from Maryland is associated to the Italian cluster.

Finally, Ticino and Italian individuals form a unique population, given the sharing of one single mitogenome variant not detected elsewhere. Geographical continuity and timing, with initial detection in 2017^18,19^ concurrent to the expansion of the Italian population northward to the Italian-Switzerland border^17^, further support this view.

### Reevaluation of available genomic data

Relevant for backward reference, individual DMR00c, which was sampled in Vancouver and used by Canada's Genomic Enterprise to produce the current draft of the nuclear genome of the species (PRJNA514809), belongs to the major invasive group that differentiated and spread in the USA (6) and, within this latter, is very closely related to the invasive group observed in Italy and Ticino. Individual NC_038115, that was sampled in Ottawa and used in^24^ to produce the current reference mitochondrial genome, in turn belongs to one secondary and unrelated group (4), likely a separate invasion from Japan that gave rise to a limited expansion in North America.

### Mode of introduction

Related to the geographic origin of the introduction is the mode of introduction, of interest from a historical perspective but also of primary importance in terms of possible quarantine strategies and control.

The observation that some sites associated to introductions are characterized by the presence of major international airports may suggest that passive relocation may take place in association with air transport of people and goods. The initial introduction from Japan to the USA cannot have taken place by aircraft, as intercontinental transportation by air was nonexistent in ~ 1910. On the other hand, the Lajes American military base in Terceira (Azores) was very active in the 1970s when, after being established during the Second World War, it gained momentum during the Cold War^38^. The Malpensa Airport of Milan, standing 10 km from the site where *P. japonica* was first detected in Italy, was reorganized as a civil airport after the Second World War and operates regular connections with the USA since 1959. It is now the largest Italian airport in terms of goods transportation, with over half a million tons of cargo per year^39^. The Cameri military airport, at a similar distance, opened as a flight school in 1910. Rebuilt after Word War II, it hosted different military forces and is now use for aviation maintenance^40^.

While intercontinental long range introductions are arguably rare and unlikely events, episodes of contiguous range expansion (USA-to-Canada, Italy-to-Ticino) are easily explained given the high reproductive potential and flight capabilities of *P. japonica.* In fact, adults were observed to fly for up to 12 km in 24 h^41^. Furthermore, the overall rate of spread after initial colonization in the USA was reconstructed as exponential during the initial phase, to level off to 7.7 to 11.9 km per year afterwards^10^. Similarly high rates of spread have been described in Italy, where *P. japonica* range expansion is actively monitored at the municipality scale^17^.

## Conclusions

While the study presented here provides a significant contribution to the understanding of the colonization process of *P. japonica*, supporting some of the conclusions of^23^ and adding an explicit time dimension and initial demographic analysis, a number of issues remain open to investigation and/or may require different sets of data to be addressed in full. The most relevant ones include: (a) a single vs. double (i.e., independent) origin of the Azorean and Italian invasion; (b) modeling of the timing and demographic aspects specifically during invasion bottlenecks; (c) identifying genes that may have been under selection during the invasion; (d) identifying the source of invading populations with a fine scale geographic resolution; e) identifying the means of transportation responsible for the invasions/range expansion. Issues (a), (d) and (e) were recently addressed in^23^ based on microsatellite data and mitochondrial haplotypes. We are currently tackling issues (a)–(c) by studying nuclear SNPs in a selection of genomes worldwide. On the other hand, addressing issues (d) and (e) in more detail would require a different study design, with a relatively small set of microsatellite markers and/or SNPs to be genotyped in a massive number of densely sampled individuals throughout the species’ range, something that may be hindered by the difficulty associated to sampling and shipping samples over such a vast area.

## Methods

A new sequence for the mitochondrial genome of *P. japonica* was produced from an individual sampled in Cameri (Italy) and was used as a reference in all subsequent analyses. The opportunity for a new reference sequence comes from: (a) the fact that the current reference sequence for *P. japonica* (NC_038115) harbors a composite set of repeats in the control region, whose number might be underestimated (as from preliminary remapping of Illumina reads on this genome); and (b) the availability of long reads, that are technically more appropriate to resolve long and complex repeat structures.

DNA from a pool of three individuals collected in Cameri (Italy) was used for Oxford Nanopore MinION sequencing at BioFab Research (Rome), whereas DNA from a single individual from the same location was used for Illumina sequencing at the Department of Medical Biotechnologies of the University of Siena. Raw data were submitted to NCBI as SRR20647940 (MiniIon) and SRR20647946 (Illumina). Long reads were assembled with Canu^42^ (ver 2.0) to obtain a draft of the mitochondrial genome, that was finalized with three rounds of remapping of Illumina reads in bbmap (ver. 35; written by B. Bushnell) and polishing in pilon^43^ (ver. 1.24). Long and short reads were remapped over the final sequence to assess coverage. The sequence was automatically annotated using MITOS^44^ and the annotation manually curated to produce the final annotations. The sequence, processed using aln2tbl^45^ and submitted to NCBI as OP974626, was used as a reference throughout this study.

Many individuals were collected from the entire geographic distribution of *P. japonica*, including its presumed ancestral area in Japan as well as from invasive populations in the USA, Azores (Portugal), Italy and Ticino (Switzerland). Individuals from the same site were collected at a minimum distance of several dozens of meters to avoid sampling of close relatives (see Supplementary Table S1 for full information). In order to obtain high quality DNA while minimizing possible gut and integument contaminants, DNA was purified from manually dissected male testicles using the Wizard Genomic DNA Purification kit (Promega). Sequencing was performed at Macrogen Europe (The Netherlands) on TruSeq DNA PCR free libraries (Illumina) with an insert size of 350 bp and a PE 150 bp strategy, targeting 20 gigabases of sequence data per individual. Sequences from three additional individuals (DMR5, DMR4 and DMR6), obtained in the context of our own attempt at genome sequencing, as well as data from the current genome draft (NCBI accession SRR8479473, unpublished) were added. Raw data of these latter libraries were subsampled, based on initial remapping, to 11.5%, 10.5%, 12% and 21%, respectively, to obtain a coverage comparable to other samples.

Sequence quality was assessed using FastQC (ver. 0.11.9; written by S. Andrews). Trimming was accomplished using fastp^46^ (ver. 0.23.2) with an initial quality trimming (cut_front and cut_tail) of 20, cut_right with a minimal quality of 24 over a sliding window of 4 bp, overlap read correction enabled and a minimal residual length of 50 bp. Clean sequences were remapped over the reference sequence (see above) using bbmap (ver. 35; written by B. Bushnell) with a maximum divergence of 10% and a maximum indel of 20 bp. Per-base coverage was plotted in R based on the bbmap output.

Variant calling was accomplished using bcftools^47^ (ver. 1.13). In detail, sequence variants were visualized using the mpileup function, with the option to avoid SNP calling for high coverage data disabled, and called using the multiallelic caller through the call function with a ploidy of 1. Calls were filtered using the filter function and only variants with a quality > 50 and coverage (DP) > 10 were retained. In a subset of samples, all assembly/variant calling steps were manually revised in IGV^48^ (ver. 2.9.4) to confirm the appropriateness of the procedure. Filtered variants were finally back inserted in the reference sequence using the bcftools consensus function to obtain the final sequence of each individual. Low coverage regions (< 15, all within the repeated region), were masked with N (see Supplementary Commands S1).

The one previously available complete mitochondrial genome from *P. japonica*^24^ (NC_038115; from Canada) was added for backward reference in all analyses. Sequences from the congeneric species *P. mutans*^49^ (NC_056126; from China) and the partial genome of a third unidentified species from the same genus *Popillia* sp. POP01^50^ (JX412777.2; from South Africa) were included as outgroups in some analyses (see Supplementary Table S1).

All *P. japonica* genomes were aligned using MUSCLE^51^ (ver. 3.8.425) through AliView^52^ (ver. 1.26), outgroups were profile-aligned. The alignment was manually verified against gene annotations for consistency. The repeated region (nucleotides 14′740 to 17′118 in the reference sequence, all within the control region) was excluded from all analyses^53^. In order to exclude the possibility of a widespread occurrence of directional selection, that may bias phylogenetic reconstructions, Ka/Ks was measured using MEGA^54^ (ver. 11), individually for each protein coding gene and on concatenated PCGs. Furthermore, the probability of rejecting neutrality in favor to positive selection was computed using MEGA among all sequence pairs of each PCG as well as concatenated PCGs. Given the limited number of variable sites, the Fisher exact test^55^ was used.

Identification of identical haplotypes was performed using the online tool Fabox^56^. Basic statistics were calculated with R packages *ape*^57^ and *pegas*^58^. Partitioning and model selection analyses were conducted (including outgroups) using the online version of IQtree^59,60^. Starting partitions, to be merged as in^61^, were: PCGs, rRNAs, tRNAs and non coding areas. Phylogenetic reconstruction was performed using the tree inference function of IQtree, applying the previously selected partitioning scheme (one partition), model (TN + F + G4) and estimating nodal support based 1000 ultrafast bootstrap replicates.

Population level processes were modelled using an approximate bayes computation approach in DIYABC^62^ (ver 2.1.0). Only *P. japonica* sequences were used. Two alternative scenarios were defined with 4 populations each: Japan, USA, Azores, Italy. Based on limited number and genetic similarity, samples from Canada and Ticino (Switzerland) were merged with USA and Italy, respectively. Invasions were modeled as Japan-to-USA, USA-to-the-Azores (common to both scenarios) and Azores-to-Italy (henceforth ‘single introduction’) or USA-to-Italy (henceforth ‘double introduction’). Invasion dates were set as hard constraints as 112, 52 and 12 generations ago to the USA, Azores and Italy respectively, assuming one generation per year^1^ and predating split times with respect to initial official reports of the pest by 5/6 years in line with the time lag from invasion to first detection reconstructed for the US^8^. Priors for the modeling of sequence evolution were set based on IQtree model selection and parameter optimization performed as described above (single partition; model: TN + I + Г; CT: 25; AG: 41; %invariant: 85; α: 1.17). A flat prior was used for the rate of sequence evolution between 1.115E^−8^ substitutions per site per generation (s/s/g, corresponding to the Brower generalized long term insect clock) and 1.5E^−5^ (5 times faster than the highest figure available in the literature). The number of haplotypes and mean pairwise differences were used as summary statistics for both the one-sample and the two-sample sets. Scenarios were compared based on direct as well as logistic regression. Parameters were estimated based on 1% of closest simulations. Attempts to introduce more complex population models, including a two-step parametrization of Ne following invasion to model the actual bottleneck step, all led to simulated data largely inconsistent with observed data, most likely as an outcome of overparameterization given the availability of one single locus.

Dating of the genome tree, unique haplotypes only, was accomplished in BEAST^63^ (ver. 1.10.4). TN + I + Г, with parameters free to vary, empirical base frequencies, an uncorrelated lognormal clock^64^, and a coalescent with expansion growth were used as model. The prior for the rate of sequence evolution was set to normal, with mean 1.14 s/s/My and standard deviation 0.4 to reproduce the rate, inclusive of uncertainty, estimated in DIYABC. Analysis was continued for 100 million generations and, after assessing convergence in Tracer^65^ (ver. 1.7.1), the initial 10% were removed as burnin. The max clade credibility tree was annotated with median heights in treeannotator (ver. 1.10.4) and visualized in FigTree (ver. 1.4.4; written by A. Rambaut).

Population size through time was studied in BEAST^63^ (ver. 1.10.4). A coalescent bayesian skyline^66^, with piecewise constant population size in 5 intervals, was used as population size prior. Model and parameters of sequence evolution were set as above. Five early diverging genomes from South Japan were removed from the analysis to limit the confounding effect of population structure on bayesian skyline plot estimation^67^. The analysis was continued for 100 million generations and, after assessing convergence in Tracer, the initial 10% generations were removed as burnin. The corresponding skyline plot was obtained in Tracer.

### Ethics declaration

Individuals of *Popillia japonica* have been shipped to the University of Siena, that is currently in a *Popillia*-free area, as dead and preserved in compliance with national safety regulations.

### Supplementary Information

Supplementary Information 1.

Supplementary Table S1.

Supplementary Information 2.

## Data Availability

Raw data as well as annotated genomes were deposited in NCBI within BioProject PRJNA860365. See Supplementary Table S1 for accessions of individual mitogenomes. The dataset, in fasta format, can be downloaded as Supplementary Data S1.
