# Supplementary material for: The direction, timing and demography of Popillia japonica (Coleoptera) invasion reconstructed using complete mitochondrial genomes
Source: Sci Rep. 2024 Mar 26;14:7120. doi: 10.1038/s41598-024-57667-x (PMC10965970; doi:10.1038/s41598-024-57667-x)
Supplement: Supplementary file 1 — Supplementary Information 1. [file 41598_2024_57667_MOESM1_ESM.pdf]

The direction, timing and demography of *Popillia japonica* (Coleoptera) invasion reconstructed using complete mitochondrial genomes.

Francesco Nardi[1,2,3,*], Sara Boschi[1], Rebecca Funari[1], Claudio Cucini[1], Elena Cardaioli[1], Daniel Potter[4], Shin-Ichiro Asano[5], Duarte Toubarro[6], Michela Meier[7], Francesco Paoli[8], Antonio Carapelli[1,2,3], Francesco Frati[1,2,3]

1: Dept. of Life Sciences, University of Siena, Italy.
2: NBFC, National Biodiversity Future Center, Palermo, Italy.
3: BAT-center, Interuniversity Center for Studies on Bioinspired Agro-Environmental Technology, Italy.
4: Dept of Entomology, University of Kentucky, USA
5: Research Faculty of Agriculture, Hokkaido University, Japan.
6: Biotechnology Centre of Azores, University of the Azores, Portugal.
7: Servizio fitosanitario cantonale, Dipartimento delle finanze e dell'economia, Switzerland.
8: Council for Agricultural Research and Agricultural Economy Analysis (CREA), Italy.
*: Corresponding author. Francesco Nardi, Dept. of Life Sciences, University of Siena, via Aldo Moro 2, 53100 Siena, Italy. E-mail: francesco.nardi@unisi.it

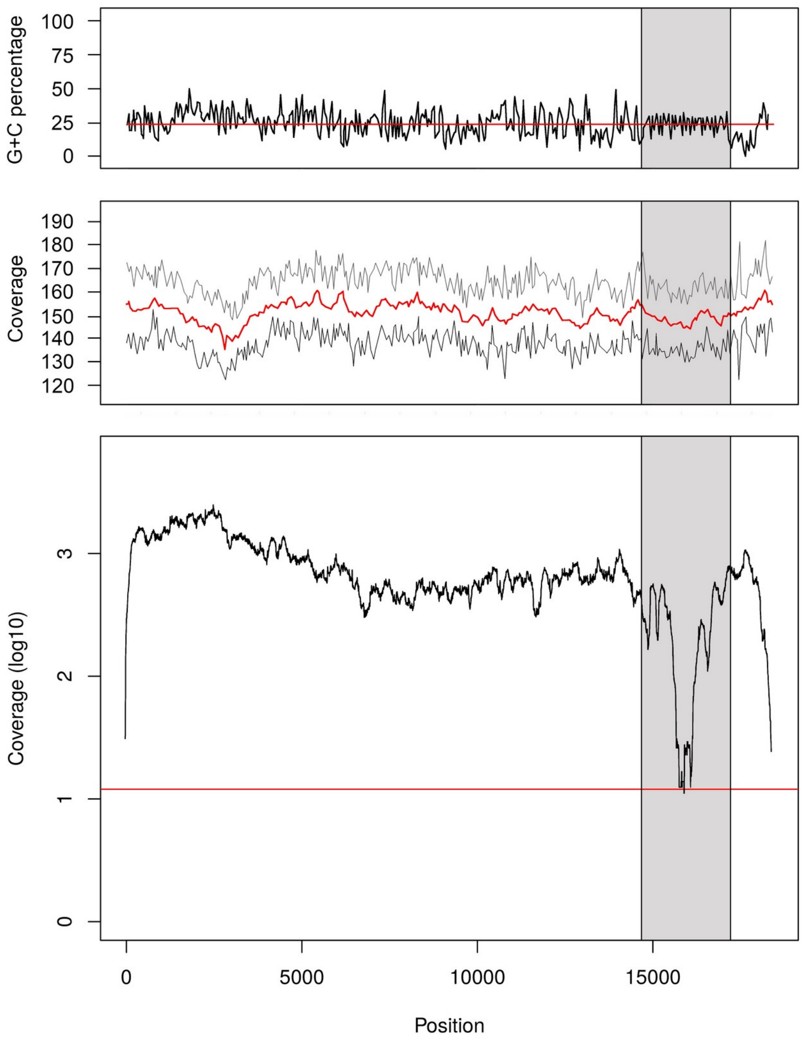

Supplementary Figure S1: Composition and coverage along the reference genome. Upper panel: G+C percentage, red line indicates average (24.2%). Mid panel: coverage based on MiniIon reads (400bp window with standard deviation). Bottom panel: coverage based on Illumina reads, red line indicates the threshold applied for SNP calling (see text). Grey rectangles indicate the repeated region.

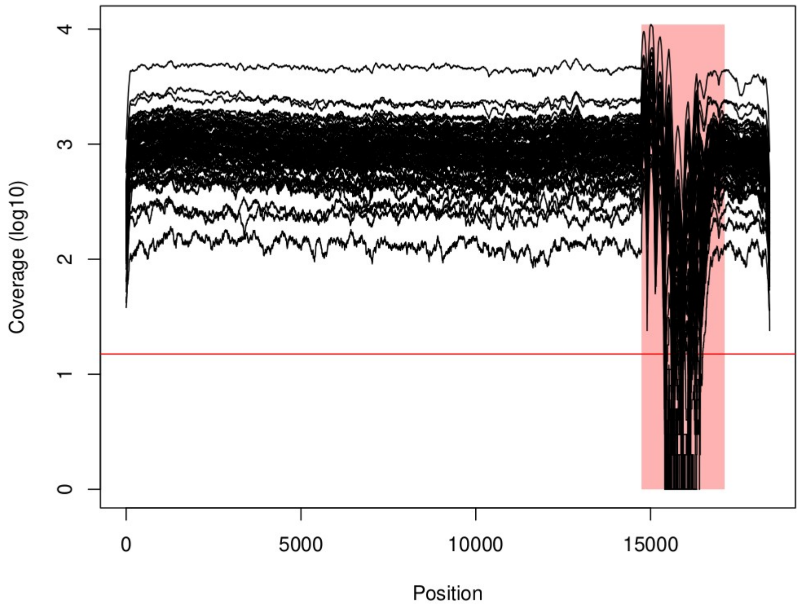

Supplementary Figure S2: Coverage over the reference sequence. Black lines indicate coverage of the 85 sequenced genomes. Red line indicates the minimal threshold applied for variant calling. Red rectangle indicates the repeats area.

Gene	Codons	Ks	Ka	Ka/Ks	Fisher test
COI	513	0.0443	0.0004	0.0079	NS (all pairs)
COII	229	0.0468	0.0012	0.0265	NS (all pairs)
COIII	262	0.0536	0.0010	0.0184	NS (all pairs)
Cytb	380	0.0458	0.0009	0.0204	NS (all pairs)
A6	224	0.0304	0.0026	0.0868	NS (all pairs)
A8	51	0.0351	0.0034	0.0970	NS (all pairs)
ND1	316	0.0409	0.0013	0.0315	NS (all pairs)
ND2	335	0.0352	0.0027	0.0754	NS (all pairs)
ND3	117	0.0472	0.0022	0.0456	NS (all pairs)
ND4	445	0.0387	0.0007	0.0189	NS (all pairs)
ND4L	96	0.0313	0.0002	0.0076	NS (all pairs)
ND5	571	0.0378	0.0013	0.0350	NS (all pairs)
ND6	167	0.0337	0.0005	0.0148	NS (all pairs)
all PCGs	3706	0.0407	0.0012	0.0304	NS (all pairs)

Supplementary Table S2: Directional selection. The number of aligned codons, Ks, Ka, Ka/Ks ratio and the results of the Fisher exact test for selection are presented for each protein coding gene and for concatenated protein coding genes. NS: not significant ($p>0.05$).

```
# Supplementary commands S1
# these commands are not meant to be run as a script. Paths and
resources (i.e. RAM, processors) have to be adapted to the user's
computing environment. Furthermore, in order to process multiple
files efficiently, some sort of looping over individuals is
recommended.


# pre-trimming quality control in fastQC
fastqc -t 32 *.fastq.gz

# trimming in fastp
fastp \
--thread 16 \
-i GENOME_1.fastq.gz -I GENOME_2.fastq.gz \
-o GENOME_1t.fastq.gz -O GENOME_2t.fastq.gz \
-l 50 \
-h GENOME_stats \
--dont_eval_duplication \
--correction \
--cut_right \
--cut_right_window_size 4 \
--cut_right_mean_quality 24 \
--cut_front \
--cut_front_window_size 1 \
--cut_front_mean_quality 20 \
--cut_tail \
--cut_tail_window_size 1 \
--cut_tail_mean_quality 20

# post-trimming quality control in fastQC
fastqc -t 32 *t.fastq.gz

# mapping over reference genome (named reference.fas and indexed)
bbmap.sh t=32 \
ref=reference.fas \
in=GENOME_1t.fastq.gz \
in2=GENOME_2t.fastq.gz \
outm=GENOME.mapped.bam \
minidentity=0.90 \
maxindel=20 \
pairlen=500 \
basecov=GENOME.basecov

# sort and index mapped reads
samtools sort -o GENOME.mapped.sort.bam GENOME.mapped.bam
samtools index -b GENOME.mapped.sort.bam

# creates a 1-based coverage file and masking file for low
coverage regions
awk 'BEGIN {OFS="\t"}; {if (NR!=1) print $1, $2+1, $3}'
GENOME.basecov > GENOME.basecov1
awk '$3 < 15 { print $1, $2 }' GENOME.basecov1 > GENOME.mask
```

```
# call and filter snp, index
bcftools mpileup -Ov -d 10000 -L 10000 -o GENOME.raw.vcf -f
reference.fas GENOME.mapped.sort.bam
bcftools call -Ov -o GENOME.called.vcf -mv --ploidy 1
GENOME.raw.vcf
bcftools filter -s LowQual -Oz -e 'QUAL<50 || DP<10'
GENOME.called.vcf > GENOME.filtered.vcf.bgzip
bcftools index GENOME.filtered.vcf.bgzip

# reintroduce snp in original sequence and mask
bcftools consensus -i 'FILTER="PASS"' -f reference.fas -m
GENOME.mask -o GENOME.mito.fas GENOME.filtered.vcf.bgzip
```
