# Supplementary material for: The direction, timing and demography of Popillia japonica (Coleoptera) invasion reconstructed using complete mitochondrial genomes
Source: Sci Rep. 2024 Mar 26;14:7120. doi: 10.1038/s41598-024-57667-x (PMC10965970; doi:10.1038/s41598-024-57667-x)
Supplement: Supplementary file 2 — Supplementary Table S1. [file 41598_2024_57667_MOESM2_ESM.pdf]

Supplementary Table S1: List of samples. Region is indicated as: JP (Japan), USA (USA), CAN (Canada), AZO (Azore Islands, Portugal), ITA (Italy), TIC (Ticino, Switzerland). NCBI accession numbers are reported for Biosample, SRA and annotated genomes. Sequencing, trimming and variant calling statistics are reported for each genome, outliers are indicated in grey shading.

SAMPLE	REGION	YEAR	LOCATION	COORDINATES	NCBI SAMPLE	NCBI SRA	BEFORE TRIMMING		AFTER TRIMMING		MAPPING		SNP CALLING		NCBI MITOGENOME
							Total reads	Total bases	Total reads	Total bases	%mapped	avg_coverage	snp vs. reference	indel vs. reference	
DMR120j	JP	2021	Japan: Fukuoka (Kyushu island)	33.5929 N 130.2136 E	SAMN31784955	SRR22354758	124.040516 M	18.514079 G	117.868050 M	17.314410 G	0.065	607.064	21	1	OP903023
DMR122j	JP	2021	Japan: Fukuoka (Kyushu island)	33.5929 N 130.2136 E	SAMN31784956	SRR22354759	124.221332 M	18.460042 G	115.249762 M	16.728405 G	0.097	874.995	354	2	OP903024
DMR125j	JP	2021	Japan: Fukuoka (Kyushu island)	33.5929 N 130.2136 E	SAMN31784957	SRR22354772	138.507982 M	20.678714 G	132.208870 M	19.473107 G	0.056	586.921	354	2	OP903025
DMR127j	JP	2021	Japan: Fukuoka (Kyushu island)	33.5929 N 130.2136 E	SAMN31784958	SRR22354792	135.662878 M	20.257868 G	129.618488 M	19.099320 G	0.081	827.655	346	2	OP903026
DMR128j	JP	2021	Japan: Fukuoka (Kyushu island)	33.5929 N 130.2136 E	SAMN31784959	SRR22354757	133.124164 M	19.779447 G	122.497922 M	17.754369 G	0.046	436.685	351	2	OP903027
DMR130j	JP	2021	Japan: Fukuoka (Kyushu island)	33.5929 N 130.2136 E	SAMN31784960	SRR22354747	137.008330 M	20.659057 G	127.518628 M	18.623636 G	0.057	573.072	349	0	OP903028
DMR133j	JP	2021	Japan: Mori (Honshu island)	34.8658 N 137.9495 E	SAMN31784961	SRR22354770	129.542592 M	19.261114 G	120.394848 M	17.522967 G	0.096	901.476	91	3	OP903029
DMR75j	JP	2021	Japan: Mori (Honshu island)	34.8658 N 137.9495 E	SAMN31784962	SRR22354724	136.350782 M	20.272776 G	127.166408 M	18.544699 G	0.097	963.658	104	6	OP903030
DMR76j	JP	2021	Japan: Mori (Honshu island)	34.8658 N 137.9495 E	SAMN31784963	SRR22354727	136.748090 M	20.315353 G	125.951054 M	18.259481 G	0.108	1056.22	92	3	OP903031
DMR77j	JP	2021	Japan: Mori (Honshu island)	34.8658 N 137.9495 E	SAMN31784964	SRR22354725	137.380364 M	20.538061 G	131.698954 M	19.426369 G	0.105	1099.729	104	6	OP903032
DMR173j	JP	2021	Japan: Tsuruoka (Honshu island)	38.6846 N 139.8573 E	SAMN31784965	SRR22354760	141.704926 M	21.364952 G	131.715970 M	19.252525 G	0.124	1289.018	98	5	OP903033
DMR175j	JP	2021	Japan: Tsuruoka (Honshu island)	38.6846 N 139.8573 E	SAMN31784966	SRR22354761	139.565028 M	21.050646 G	130.198244 M	19.066010 G	0.165	1701.6	80	4	OP903034
DMR176j	JP	2021	Japan: Tsuruoka (Honshu island)	38.6846 N 139.8573 E	SAMN31784967	SRR22354762	136.858268 M	20.636021 G	127.368408 M	18.609540 G	0.125	1249.532	144	7	OP903035
DMR177j	JP	2021	Japan: Tsuruoka (Honshu island)	38.6846 N 139.8573 E	SAMN31784968	SRR22354763	138.883452 M	20.939684 G	127.711318 M	18.557219 G	0.126	1262.67	150	6	OP903036
DMR178j	JP	2021	Japan: Tsuruoka (Honshu island)	38.6846 N 139.8573 E	SAMN31784969	SRR22354764	137.536666 M	20.737502 G	127.639070 M	18.632885 G	0.131	1311.759	149	5	OP903037
DMR72j	JP	2021	Japan: Nanae (Hokkaido island)	41.9884 N 140.639 E	SAMN31784970	SRR22354765	145.694254 M	21.967987 G	134.507290 M	19.604990 G	0.062	650.506	73	3	OP903038
DMR74j	JP	2021	Japan: Nanae (Hokkaido island)	41.9884 N 140.639 E	SAMN31784971	SRR22354766	136.069310 M	20.517201 G	126.358112 M	18.454667 G	0.102	1014.033	75	3	OP903039
DMR179j	JP	2021	Japan: Sapporo (Hokkaido island)	43.1881 N 141.3997 E	SAMN31784972	SRR22354767	138.804830 M	20.718527 G	132.484218 M	19.511334 G	0.149	1560.151	145	9	OP903040
DMR180j	JP	2021	Japan: Sapporo (Hokkaido island)	43.1881 N 141.3997 E	SAMN31784973	SRR22354768	142.169830 M	21.435352 G	132.088166 M	19.266795 G	0.137	1419.639	98	5	OP903041
DMR184j	JP	2021	Japan: Horokanai (Hokkaido island)	44.2138 N 142.1769 E	SAMN31784974	SRR22354771	139.195684 M	20.988443 G	129.182992 M	18.857420 G	0.099	1006.829	80	4	OP903042
DMR185j	JP	2021	Japan: Horokanai (Hokkaido island)	44.2138 N 142.1769 E	SAMN31784975	SRR22354773	137.515192 M	20.737470 G	128.094150 M	18.711388 G	0.122	1227.864	78	3	OP903043
DMR152u	USA	2021	USA: Auburn (Alabama)	32.59933574 N 85.497355 W	SAMN31784976	SRR22354774	125.209472 M	18.683346 G	119.426902 M	17.570233 G	0.081	760.613	12	3	OP903044
DMR85u	USA	2021	USA: Auburn (Alabama)	32.59933574 N 85.497355 W	SAMN31784977	SRR22354780	134.967794 M	20.031994 G	123.539118 M	17.823343 G	0.111	1059.836	5	3	OP903045
DMR161u	USA	2021	USA: Cotter (Arkansas)	36.335 N 92.55111111 W	SAMN31784978	SRR22354781	136.746748 M	20.617105 G	126.446644 M	18.400320 G	0.098	970.425	7	3	OP903046
DMR64u	USA	2021	USA: Cotter (Arkansas)	36.335 N 92.55111111 W	SAMN31784979	SRR22354785	140.144782 M	21.121317 G	132.176192 M	19.337033 G	0.1	1044.837	10	3	OP903047
DMR148u	USA	2021	USA: Littleton (Colorado)	39.6102105 N 105.015159 W	SAMN31784980	SRR22354786	137.535126 M	20.734742 G	128.621576 M	18.817826 G	0.103	1048.322	8	3	OP903048
DMR60u	USA	2021	USA: Littleton (Colorado)	39.6102105 N 105.015159 W	SAMN31784981	SRR22354788	131.952226 M	19.890460 G	124.641702 M	18.250999 G	0.097	953.701	8	3	OP903049
DMR79u	USA	2021	USA: Lexington (Kentucky)	38.1277778 N 84.5616667 W	SAMN31784982	SRR22354789	124.341076 M	18.562852 G	118.741114 M	17.490243 G	0.102	959.578	9	3	OP903050
DMR80u	USA	2021	USA: Lexington (Kentucky)	38.1277778 N 84.5616667 W	SAMN31784983	SRR22354790	13.949260 M	2.081806 G	13.331786 M	1.962683 G	0.123	129.703	166	4	OP903051
DMR201u	USA	2021	USA: Lexington (Kentucky)	38.1277778 N 84.5616667 W	SAMN31784984	SRR22354791	131.331222 M	19.527180 G	122.708554 M	17.809867 G	0.149	1432.635	7	3	OP903052
DMR149u	USA	2021	USA: Laytonsville (Maryland)	39.246989 N 77.145480 W	SAMN31784985	SRR22354802	136.928946 M	20.427725 G	130.246902 M	19.138120 G	0.081	829.167	80	4	OP903053
DMR81u	USA	2021	USA: Laytonsville (Maryland)	39.246989 N 77.145480 W	SAMN31784986	SRR22354801	133.999596 M	19.917595 G	124.449696 M	18.113631 G	0.083	808.482	4	1	OP903054
DMR83u	USA	2021	USA: East Lansing (Michigan)	42.7061293 N 84.4897534 W	SAMN31784987	SRR22354800	128.557570 M	19.135808 G	120.118070 M	17.525269 G	0.171	1612.35	7	3	OP903055
DMR84u	USA	2021	USA: East Lansing (Michigan)	42.7061293 N 84.4897534 W	SAMN31784988	SRR22354799	125.222374 M	18.627706 G	116.511654 M	16.997344 G	0.108	990.086	59	3	OP903056
DMR159u	USA	2021	USA: Rosemount (Minnesota)	44.7279521 N 93.0967194 W	SAMN31784989	SRR22354798	140.956868 M	21.254512 G	131.295276 M	19.191581 G	0.12	1236.721	78	3	OP903057
DMR62u	USA	2021	USA: Rosemount (Minnesota)	44.7279521 N 93.0967194 W	SAMN31784990	SRR22354797	139.514546 M	21.029934 G	131.641910 M	19.285964 G	0.093	968.453	77	2	OP903058
DMR153u	USA	2021	USA: Omaha (Nebraska)	41.2487219 N 96.1760343 W	SAMN31784991	SRR22354796	136.655266 M	20.605524 G	125.623814 M	18.261331 G	0.092	900.952	76	3	OP903059
DMR54u	USA	2021	USA: Omaha (Nebraska)	41.2487219 N 96.1760343 W	SAMN31784992	SRR22354795	138.033624 M	20.797555 G	125.896804 M	18.147726 G	0.062	604.57	13	3	OP903060
DMR156u	USA	2021	USA: New Brunswick (New Jersey)	40.481540 N 74.438348 W	SAMN31784993	SRR22354794	137.976080 M	20.802662 G	125.658800 M	18.189637 G	0.105	1030.093	19	3	OP903061
DMR55u	USA	2021	USA: New Brunswick (New Jersey)	40.481540 N 74.438348 W	SAMN31784994	SRR22354793	138.177528 M	20.821193 G	130.751398 M	19.175929 G	0.066	685.938	77	2	OP903062
DMR158u	USA	2021	USA: Portland (Oregon)	45.545923 N 122.795071 W	SAMN31784995	SRR22354756	144.417134 M	21.771585 G	132.871428 M	19.296384 G	0.113	1174.896	7	3	OP903063
DMR63u	USA	2021	USA: Portland (Oregon)	45.545923 N 122.795071 W	SAMN31784996	SRR22354755	136.842356 M	20.623482 G	129.387904 M	18.979236 G	0.113	1154.454	7	3	OP903064
DMR00c	CAN	~2018	Canada: Vancouver	49.272499N 123.124276W	SAMN10734816	SRR8479473	183.343906 M	27.684930 G	142.196450 M	19.054714 G	0.088	961.365	0	1	OP951914
DMR136a	AZO	2021	Portugal: Sao Jorge island (Azores)	38.7011111 N 28.1819444 W	SAMN31784997	SRR22354754	137.485892 M	20.517780 G	131.289462 M	19.330720 G	0.026	270.085	12	2	OP903065
DMR137a	AZO	2021	Portugal: Sao Jorge island (Azores)	38.7011111 N 28.1819444 W	SAMN31784998	SRR22354753	132.662996 M	19.996880 G	120.714328 M	17.469704 G	0.046	429.76	12	2	OP903066
DMR138a	AZO	2021	Portugal: Sao Jorge island (Azores)	38.7011111 N 28.1819444 W	SAMN31784999	SRR22354787	143.453278 M	21.627797 G	132.572482 M	19.295767 G	0.044	455.396	12	2	OP903067
DMR47a	AZO	2021	Portugal: Sao Jorge island (Azores)	38.7011111 N 28.1819444 W	SAMN31785000	SRR22354752	137.711824 M	20.753730 G	130.145948 M	19.055973 G	0.078	806.025	12	2	OP903068
DMR59a	AZO	2021	Portugal: Sao Jorge island (Azores)	38.7011111 N 28.1819444 W	SAMN31785001	SRR22354748	137.502150 M	20.718110 G	129.390294 M	18.921027 G	0.055	556.879	12	2	OP903069
DMR65a	AZO	2021	Portugal: Sao Jorge island (Azores)	38.7011111 N 28.1819444 W	SAMN31785002	SRR22354784	125.525326 M	18.911422 G	117.499140 M	17.166204 G	0.077	709.405	12	2	OP903070
DMR66a	AZO	2021	Portugal: Sao Jorge island (Azores)	38.7011111 N 28.1819444 W	SAMN31785003	SRR22354783	136.179494 M	20.345470 G	130.325282 M	19.193794 G	0.093	970.107	12	2	OP903071
DMR90a	AZO	2021	Portugal: Sao Jorge island (Azores)	38.7011111 N 28.1819444 W	SAMN31785004	SRR22354782	134.349622 M	19.968496 G	124.996592 M	18.195698 G	0.17	1659.515	0	0	OP903072
DMR91a	AZO	2021	Portugal: Sao Jorge island (Azores)	38.7011111 N 28.1819444 W	SAMN31785005	SRR22354746	134.156330 M	19.976579 G	125.769738 M	18.395439 G	0.079	778.302	12	2	OP903073
DMR94a	AZO	2021	Portugal: Sao Jorge island (Azores)	38.7011111 N 28.1819444 W	SAMN31785006	SRR22354779	125.432468 M	18.471158 G	107.010598 M	15.118867 G	0.061	493.235	12	2	OP903074
DMR139a	AZO	2021	Portugal: Sao Miguel island (Azores)	37.7802778 N 25.6844444 W	SAMN31785007	SRR22354778	134.988718 M	20.148705 G	128.698968 M	18.942448 G	0.023	232.109	11	2	OP903075
DMR141a	AZO	2021	Portugal: Sao Miguel island (Azores)	37.7802778 N 25.6844444 W	SAMN31785008	SRR22354777	140.038354 M	21.113929 G	129.321414 M	18.798715 G	0.121	1220.237	11	1	OP903076
DMR142a	AZO	2021	Portugal: Sao Miguel island (Azores)	37.7802778 N 25.6844444 W	SAMN31785009	SRR22354776	139.384406 M	21.016009 G	130.002732 M	19.001435 G	0.059	605.345	12	1	OP903077
DMR144a	AZO	2021	Portugal: Sao Miguel island (Azores)	37.7802778 N 25.6844444 W	SAMN31785010	SRR22354775	141.432670 M	21.325943 G	130.947506 M	19.119874 G	0.052	534.484	12	1	OP903078
DMR145a	AZO	2021	Portugal: Sao Miguel island (Azores)	37.7802778 N 25.6844444 W	SAMN31785011	SRR22354745	136.654990 M	20.606661 G	126.217612 M	18.391246 G	0.073	724.532	12	1	OP903079
DMR56a	AZO	2021	Portugal: Sao Miguel island (Azores)	37.7802778 N 25.6844444 W	SAMN31785012	SRR22354744	137.538534 M	20.726007 G	130.563282 M	19.166286 G	0.098	1008.094	12	2	OP903080
DMR57a	AZO	2021	Portugal: Sao Miguel island (Azores)	37.7802778 N 25.6844444 W	SAMN31785013	SRR22354743	140.389396 M	21.153450 G	132.876488 M	19.478858 G	0.068	718.069	12	1	OP903081
DMR69a	AZO	2021	Portugal: Sao Miguel island (Azores)	37.7802778 N 25.6844444 W	SAMN31785014	SRR22354742	136.515576 M	20.575387 G	129.406438 M	18.993529 G	0.093	951.835	12	2	OP903082
DMR70a	AZO	2021	Portugal: Sao Miguel island (Azores)	37.7802778 N 25.6844444 W	SAMN31785015	SRR22354769	138.095590 M	20.808526 G	129.812398 M	18.989844 G	0.078	791.033	12	1	OP903083
DMR98a	AZO	2021	Portugal: Sao Miguel island (Azores)	37.7802778 N 25.6844444 W	SAMN31785016	SRR22354741	130.827540 M	19.454993 G	121.969830 M	17.768851 G	0.065	620.421	12	1	OP903084
DMR11i	ITA	2021	Italy: Cameri	45.5128460 N 8.6908590 E	SAMN31785017	SRR22354740	130.879226 M	19.720474 G	123.452346 M	18.079879 G	0.076	736.515	0	0	OP903085
DMR14i	ITA	2021	Italy: Cameri	45.5128460 N 8.6908590 E	SAMN31785018	SRR22354739	141.081162 M	21.267279 G	133.413222 M	19.564145 G	0.075	792.417	0	0	OP903086
DMR168i	ITA	2021	Italy: Cameri	45.5128460 N 8.6908590 E	SAMN31785019	SRR22354738	138.318664 M	20.652271 G	131.994120 M	19.434178 G	0.094	978.104	0	0	OP903090
DMR170i	ITA	2021	Italy: Cameri	45.5128460 N 8.6908590 E	SAMN31785020	SRR22354737	136.897764 M	20.644660 G	127.064420 M	18.555940 G	0.069	687.258	0	0	OP903091
DMR4i	ITA	2020	Italy: Cameri	45.5128460 N 8.6908590 E	SAMN29883562	SRR20647930	79.594908 M	18.678957 G	73.060204 M	15.998113 G	0.077	639.587	0	0	OP903087
DMR5i	ITA	2020	Italy: Cameri	45.5128460 N 8.6908590 E	SAMN29883563	SRR20647946	81.253490 M	19.125321 G	73.935736 M	16.103984 G	0.046	387.457	0	0	OP903088
DMR6i	ITA	2020	Italy: Cameri	45.5128460 N 8.6908590 E	SAMN29883564	SRR20647939	80.765458 M	18.927850 G	74.412082 M	16.263202 G	0.048	407.015	0	0	OP903089
DMR163i	ITA	2021	Italy: Trecate	45.4343488 N 8.6951257 E	SAMN31785021	SRR22354736	138.878640 M	20.728655 G	132.425306 M	19.470552 G	0.041	429.414	0	0	OP903092
DMR165i	ITA	2021	Italy: Trecate	45.4343488 N 8.6951257 E	SAMN31785022	SRR22354735	136.290950 M	20.321237 G	129.358364 M	18.990354 G	0.072	738.631	0	0	OP903093
DMR38i	ITA	2021	Italy: Trecate	45.4343488 N 8.6951257 E	SAMN31785023	SRR22354734	140.650376 M	21.191339 G	132.963318 M	19.483797 G	0.154	1615.254	0	0	OP903094
DMR44i	ITA	2021	Italy: Trecate	45.4343488 N 8.6951257 E	SAMN31785024	SRR22354733	139.586340 M	21.032794 G	131.267722 M	19.184481 G	0.132	1360.507	0	0	OP903095
DMR118i	ITA	2021	Italy: Pavia	45.1818310 N 9.0519230 E	SAMN31785025	SRR22354723	134.846472 M	20.018992 G	123.570350 M	17.880283 G	0.088	837.711	0	0	OP903096
DMR30i	ITA	2021	Italy: Pavia	45.1818310 N 9.0519230 E	SAMN31785026	SRR22354722	137.812978 M	20.575920 G	130.904992 M	19.206428 G	0.43	4458.323	0	0	OP903097
DMR36i	ITA	2021	Italy: Pavia	45.1818310 N 9.0519230 E	SAMN31785027	SRR22354732	143.490280 M	21.624070 G	135.539206 M	19.863647 G	0.135	1446.337	0	0	OP903098
DMR40i	ITA	2021	Italy: Pavia	45.1818310 N 9.0519230 E	SAMN31785028	SRR22354731	140.048114 M	21.105481 G	132.100370 M	19.353739 G	0.226	2351.674	0	0	OP903099
DMR41i	ITA	2021	Italy: Pavia	45.1818310 N 9.0519230 E	SAMN31785029	SRR22354730	137.510966 M	20.723751 G	129.734064 M	18.999573 G	0.126	1288.598	0	0	OP903100
DMR43i	ITA	2021	Italy: Pavia	45.1818310 N 9.0519230 E	SAMN31785030	SRR22354729	53.163518 M	7.933387 G	50.739944 M	7.469397 G	0.116	464.892	0	0	OP903101
DMR188s	TIC	2022	Switzerland: Novazzano	45.83672 N 9.00339 E	SAMN31785031	SRR22354728	125.204462 M	18.583273 G	115.590858 M	16.728575 G	0.08	713.795	0	0	OP903102
DMR191s	TIC	2022	Switzerland: Novazzano	45.83672 N 9.00339 E	SAMN31785032	SRR22354751	123.912804 M	18.430053 G	116.046402 M	16.900755 G	0.096	870.546	0	0	OP903103
DMR193s	TIC	2022	Switzerland: Genesterio	45.84803 N 8.95685 E	SAMN31785033	SRR22354750	160.897366 M	23.915975 G	149.378382 M	21.659404 G	0.193	2240.199	0	0	OP903104
DMR194s	TIC	2022	Switzerland: Genesterio	45.84803 N 8.95685 E	SAMN31785034	SRR22354749	125.884410 M	18.711029 G	117.485028 M	17.069938 G	0.143	1311.662	0	0	OP903105
DMR196s	TIC	2022	Switzerland: Genesterio	45.84803 N 8.95685 E	SAMN31785035	SRR22354726	184.634708 M	27.438903 G	172.093554 M	24.995370 G	0.097	1304.808	0	0	OP903106

Additional genomes from NCBI

ACCESSION	SPECIES	YEAR	LOCATION	SOURCE
NC_038115	*P.japonica*	2017	Canada: Ottawa	Yang, W., Zhang, Y., Feng, S., Liu, L. & Li, Z. *Int. J. Biol. Macromol.* **118**, 1406-1413 (2018).
NC_056126.1	*P.mutans*	N/A	China: Zhengzhou, Henan province	Song, N. & Zhang, H. *J. Insect Sci.* **18**, 11 (2018).
JX412777.2	*P.sp.* POP01	N/A	South Africa	Timmermans, M. J. T. N. *et al. Genome Biol. Evol.* **8**, 161-175 (2016).
