# Supplementary material for: The direction, timing and demography of Popillia japonica (Coleoptera) invasion reconstructed using complete mitochondrial genomes
Source: Sci Rep. 2024 Mar 26;14:7120. doi: 10.1038/s41598-024-57667-x (PMC10965970; doi:10.1038/s41598-024-57667-x)
Supplement: Supplementary file 3 — Supplementary Information 2. [file 41598_2024_57667_MOESM3_ESM.docx]

>NC_038115_CA_Ottawa

AATGAAGTGTCTGACTATAGAGTTACTTTGATAGGGTAAAAAAAGTGAATTTTCACCTTCATTATAATTAATAGAATTAAACTATTTCTTTAAGCTTCAA

AAACTTATGTACATTATATACTAAATTATAAAAAGATAAGCTAATTAAGCTACTGGGCTCATACCCCATCAATAAAGGTTACAATCCTTTTCTTTTTAAT

GTATTATAAGCTTTTATTTTTTAATTCTCTTATAATTGGAACCTTAATTGCTATTTCTTCCTACTCTTGGATAGGAATATGAATAGGATTAGAAATTAAT

CTTCTCTCTATGATCCCCCTCATTAGAGACAACAAAAATATAATAGCCTCAGAAGCTGCTTTAAAATATTTCATTATTCAAACAATAGCATCAACATTGT

TATTATTCTCAATTATTATAATATCAATGAAATTTATATATCAAATAAATTTAATCACTTACTTTAATTTAATTTTTAACACTTCATTGTTCATCAAAAT

AGGAGCAGCCCCATTCCATTTTTGATTCCCCGAAATAATAGAAGGATTAAATTGATTAAATGCCATTATCATACTTACTTGACAAAAACTAAGACCCATA

GTATTACTAACGTATTCTAATACAACCTCCATATATCTAATTTTAACAATTATATTTAGAATAATAATCAGAGGAATTATAGGCCTAAATCAAACTAGAT

TACGGAAAATTATAGCTTATTCATCTATCAACCATATTGGATGAATAATCAGTTCAATTATATTAATTGAAATTGTTTGATTTTACTATTTTATTATTTA

TTGCATTATTACTATTAATATCAGAATTATATTTATAAAATTAAATGTTTTTCATATCAATCAATTATATATTTCAATAAATTACCATATTTTACTTAAA

TTATTCTTTGCCTTAAATTTCATGTCTTTAGGAGGATTACCCCCATTTTTAGGGTTTTTCCCTAAATGACTTACCATTCAAACTTTAATCCAAAGAAATA

TGTACTCAATTGCTTTCATTATAATTCTAATAACTTTAATAACACTTTTTTTTTACCTCCGAATTACTTTTTCAATTTTACTACTAAGAAAAGCAGTTTT

AACATTTTACACACAACCAAAAATTAATACTAATTACATTATAGCATTTAATTTTATTACATTATTAAGATTAATTTTCGTTACTTTGATATTCAATTTC

TTATAAATTAAACCTGAAGGATTTAAGTTAAATTAAACTAAGAACCTTCAAAGTTCTAAATAAAGTAAATTCTTTAAGCCTTAGGGCTTAGCCCATCTTT

AAATTTGCAATTTAAAATTCTTTTTGAACTATAAAGCTTGATAAAAGAAACTAATTTCGTATGTAAATTTACAGTTTACCGCCTAAACCTCGGCCATTTT

ATCGAATAAATGGCTATTCTCTACAAATCACAAAGATATCGGAACTTTATACTTTCTATTTGGAAGTTGAGCAGGAATAGTAGGCACTTCCTTAAGTTTA

CTGATTCGTGCTGAATTGGGAAACCCAGGATCTCTAATTGGAGATGATCAAATTTATAACGTAATCGTAACAGCTCATGCTTTCATTATAATTTTTTTTA

TAGTAATACCTATTATAATTGGAGGATTCGGAAACTGATTAGTTCCTTTAATGCTTGGAGCCCCCGACATAGCATTTCCCCGAATAAATAACATAAGATT

TTGACTTTTACCTCCTTCATTAACTCTACTTCTAATAAGAAGATTAGTCGAAAGAGGGGCAGGTACAGGCTGAACAGTTTATCCCCCACTATCAGCCAAT

ATCGCCCATAGAGGAGCATCAGTTGATTTAGCAATTTTTAGACTTCATCTTGCAGGAATTAGCTCAATTTTAGGTGCAGTAAATTTTATTACTACTGTAA

TTAACATACGATCAACAGGAATAACTTTTGATCGAATACCCCTATTTGTTTGATCTGTAGTTTTAACAGCACTTCTTCTGCTATTATCCCTCCCAGTTTT

AGCAGGAGCAATTACTATACTATTAACAGATCGAAATATTAATACAACATTTTTTGATCCCGCAGGTGGAGGAGACCCCATTCTCTACCAACATTTATTT

TGATTTTTTGGTCATCCAGAAGTTTACATTTTAATTTTACCTGGATTTGGTATAATCTCCCATATTATTAGCCAAGAAAGAAGAAAAAAAGAAACATTTG

GAACTTTAGGTATAATTTACGCTATAATAGCTATTGGTTTACTAGGATTTATTGTTTGAGCTCATCATATATTCACAGTAGGTATAGATGTAGACACACG

GGCATATTTTACGTCAGCAACAATAATCATTGCTGTTCCTACAGGAATTAAAATTTTTAGATGATTAGCTACTCTCCATGGATCACAATTAAACTACTCC

CCGTCTCTTTTATGGGCATTAGGATTTGTATTCCTATTTACAGTAGGAGGATTAACAGGAGTAATTCTAGCTAATTCATCAATTGACATTATTTTACATG

ATACTTACTATGTAGTTGCACATTTCCATTATGTCCTTTCCATAGGAGCTGTATTTGCTATTATAGCAGGTTTTGTTCATTGATTCCCTTTATTTACAGG

TTTAACAATAAATTCAAAATTTCTTAAAATTCAATTTTTAACAATATTTATTGGTGTTAATATAACATTCTTCCCTCAACATTTCTTAGGATTAAGAGGA

ATACCTCGACGTTATTCAGATTACCCAGATGCTTACACAACTTGAAATATTATCTCATCTATTGGATCTTTAGTTTCTTTAATTAGTATTTTTATCTTTT

TATTTACTATTTGAGAAAGGCTAATTTCATTACGAAAAAGAATTAGGTCTTTAAGAATATCTACATCAATTGAATGACTCCAACAAATACCCCCTTCAGA

ACATAGTTATTCTGAACTTCCAATGCTTACTAACTTCTAATATGGCAGATTAGTGCAATGGATTTAAACCCCAAATATAAAGATTAAACTTTTTTTAGAA

ATAGCTACTTGAAATACCATTTTACTTCAGGATAGGGCATCCCCATTAATAGAGCAACTCTCATTCTTTCATAACCATGCTCTTCTAATTCTCTTTATAA

TTACCGTTCTAGTAGGTTATTTAATAGGAACTTTATTTTTTAACCAATTTAATTACCGATTTTTATTAGATGGTCAAACTATTGAAATTATTTGAACTAT

TTTACCTGCTGTAACACTAATTTTTATCGCATTACCGTCTTTACGCTTACTTTATCTTCTAGATGAAGTTAATAACCCTTTAGTAACTATCAAAACAATT

GGGCATCAATGATATTGATCATACGAATATAGAGATTTTATAAATTTTGAATTCGATTCCTATATAATTCCTTTAACAGAAATAAAACCTCAAAATTTTC

GTTTATTAGATGTTGATAACCGAGTAATTGTCCCCTTTAACTCCCAAATCCGAATGATAGTAACAGCTGCCGATGTTATTCATTCATGAACTATCCCGGC

TTTTAGTGTAAAAATTGATGCAACACCGGGCCGACTAAATCAAATTAGATTCCTAATTAATCGAACAGGATTATTTTATGGTCAATGCTCAGAAATTTGT

GGAGCAAATCATAGATTTATACCTATTACTGTAGAAAGAATTTCACCTTCATTTTTTACTAAATGAATCTCAAAAATAAATAACCTATCATTAGATGACT

GAAAGTAAGTAATGGTCTCTTAAACCAATTAATAGTAGTTTAACATCTACTTCTGATGGCCAAAAATTTAGTTAAGATATAACATTAGTTTGTCATACTA

AAATAATCATAATTTGATAATTTTTAATTCCACAAATAGCACCTTTAAACTGACTATCTTTATTTTTTTTAATTATTATTATTTTTTTACTTTTTAATGT

ATTAAATTACTTTAGATTCTTACAGCCCTTAAAAACCCAATCTCATAACCCTACAATTAAAAAAATTAATTGAAAATGATAACTAATTTATTTTCATCTT

TTGATCCTAGAACTTCTTTTAATTTAAGATTAAACTGATTAAGAATACTATTAGGGCTAATATTTATCCCCCCAGTATTTTGATTAATTCCTTCACGCCA

TAATTTTCTATGAATTAAAATTATTTTAACATTACACCAAGAATTTAAGGTTTTAATTGGTAATAATAATATTAAAGGAAGAACCTTAATATTTATTTCA

TTATTTTCTATAATTGTTTTCAATAACTTTTTAGGATTATTTCCATATATTTTTACAGGAACAAGACATTTAATTATAACATTATCTCTTGCCTTACCTT

TATGAATTAGATTCATATTATACGGGTGAATTAATAACACTATCCACATACTTGCTCATTTAGTTCCCCAAGGAACACCCCCAGCTCTTATGGCATTCAT

AGTAGTAATTGAATCAATTAGAAATATTATTCGTCCTGGTACTTTAGCTGTTCGATTAGCTGCTAATATAATTGCTGGACATTTACTAATAACTTTACTA

GGAAACACAGGATTAAATTTATCAATTTTTATACTAAGTATTCTTATTATCACACAAATTCTTTTATTAATTTTAGAATCTGCTGTTGCGATCATTCAAT

CTTATGTATTTGCTGTATTAAGAACTTTATACTCTAGAGAAATTAATTAATGTCAAGACATAAAAATCACCCTTATCATTTAGTTGATGCAAGACCTTGA

CCTATTTTAGGCGCTTTTAGAGCTATAATTACAATAATTGGAATTATCAAATGATTCCATTTTTATAATAATTCTTTATTTTACTTAGGGACATTAATCA

CAATTTTAATTATAATTCAATGATGACGAGATATCACTCGTGAGGGAACTTTCCAAGGACTTCATACTTACGCTGTAACTATAGGTTTACGTTGAGGAAT

AATTTTATTTATTACATCAGAAGTATTTTTCTTTATTTCTTTTTTTTGAGCCTTTTTTCATAGTAGCTTAACACCCGCTATTGAACTAGGGATACTCTGA

CCACCCAAAGGAATTACCCCATTTAACCCAATTCAAATTCCATTATTAAACACTTTAATTCTTTTAACTTCAGGATTAACTGTAACTTGAGCTCACCATA

GATTAATAGAAAATGACTATAACCAAACAATGCAAGGTCTTGGTTTAACAGTTTTACTAGGAGTATATTTTACTTTATTACAAGGTTACGAATATTTAGA

AGCCCCCTTTACTATAGCAGATTCTGTTTATGGATCAACATTTTTTATTGCTACTGGTTTCCATGGATTACATGTTATTATTGGCACAACCTTTTTAGCT

GTTTGTTTAATACGACATTTTAATAACCATTTTACTTGTATCCATCACTTTGGATTTGAAGCTGCTGCTTGATACTGACATTTTGTTGATGTAGTATGAC

TATTTCTTTATATTTCTATTTACTGATGAGGTAGATATTTATATAGTATAATAATTATAATTGATTTCCAATCAAAAGATCTAAAAAAAATTAGTATAAA

TAATCATTATAATTTGAAATATAGGGTTAATTATTTTTTCTATCTCTTTTATCTTAATTATACTATCTTTTACAATCTCTAAAAAAAGATTTATAGACCG

AGAAAAAGCTTCTCCATTCGAGTGCGGATTTGACCCCAAAAGATCAGCCCGTTTACCCTTTTCTTTGCATTTTTTTTTAATTGCAGTAATTTTCTTAATT

TTTGATGTTGAAATTACTCTTCTTATCCCTTTAATTCTAACAATAAAAATTACTAATATTACTATATATACCTATATTGCTCTTTTCTTTTTAATGATTC

TTTTAATAGGACTTTACCATGAATGAAACCAAGGGGCCTTAAATTGAGCTCTTTAGGGTAATAGTTAAGTATAACATTTAAGTTGCATTTAAAAAGTATT

GATTTTTCAATTTACCTTAAATAAGAAACAATTAATTGTATTTAGTTTCGACCTAAAATTTAGGTGTATGAACACCCTTATTTAAATTAATTGAAACCAA

AAAGAGGTATATCACTGTTAATGATACTAATGAGAAAAACTCCAATTAAGGAAATAAGATATTCAAGGGTAAGCTTCTAACTTAACTCTTTAGCAGTGAA

AGTCTGTTAATATTTCTATTTATATAGTTTAATAAAACATTATTTTTTCATAATAAAATTAGAATAAATTTATTCTTATAAATATTTAAAAGTAAATTTT

ACTTCCCTGATAACTTCACTATCATACTCTATATAAGCTATTTAAATTAAATATATAAAATTATAAAAATTACCCATATTATAATTAAAAGTATAAAAAT

CTTATAATTGTTATTAAATATAAACTGTAAAAATACAGAAGTATTACTAATTTTACTATATAAATTTTGTCTTCCATAATACTCTGATCAGCCTTGATCA

ATAGTTTTATATAACTTACTACCTAATTTAATTGGATAATAATTTAAACCAAATGTTGAAATATAGGGTATATTCCACATAGAAGAAAAAAAAAGTCTTG

AATTTAACAAATAAAAAGATTTTAATCTATCATTTAAAGTAAACTTAGAAAGTTCAAACCCAAACCAAGCCCCAAAAAATGATACAATTAAAGCTATAAT

TTTTATTGTAAAAGGTAAACAAATAAAATAAGGTGTTGGGAATATCAACCATATTAATATTCTTCCCCCAACAATAACTAAAAAAATTAAACCTGATATC

CCTTGAAGTATAATTTTTCTATTATCATTAATTTTACTTAAAGAATAAAAACAAAAATTTCCTACCAAAACATAATAAATTAAACGAAATGTATAACAAA

CAGTTAATCCTGTTGAAAAAAAGAAAATAACATAAATATAGATATTTAAGTATCTTATAGATAAAACCTCTAAAATTAAATCCTTTGAATAAAACCCCGA

TAAAAAGGGTAAACCGCATAATGCTAAATTTGAAATTATAAAATAAGTACAAGTTAAAGGTATAACTTTAATTAACCCCCCTATATACCGAATATCTTGA

CAATTTCTTAATCTGTGAATTATACACCCTGCGCATATAAATAATAAAGCCTTAAATAAAGCGTGAGTTAATAGATGAAAAAAAGCTAATTGATACTCCC

CTAAAGCTAAAATACTAATTATCAAACCCAATTGTCTTAAAGTAGACAGAGCAATAATTTTTTTTAAATCAAACTCAAAGTTTGCGCCTATTCCTGCTAT

AAATATAGTTATTGTTCCAATAAATAATAAAATTAATATTAAATTACTTGTTAATGCAAAATTAAAACGAATTAATAAGTAAACACCTGCTGTTACTAAG

GTAGAAGAATGAACTAAAGAAGAAACAGGTGTTGGAGCTGCTATTGCCGCAGGCAACCAAGAAGAAAATGGAATTTGAGCTCTTTTAGTTATAGCTGCTA

ATATAATTAATAAAATAATAATATATATTTCTATACTATTTTTATATACATCAATATAAAAAATATAGTTAAATCCCCCAAAATTTATTATTCATGCAAT

TGCTATTAATAAAGCAACATCTCCAATACGGTTAGTTAAAGCTGTAATTATCCCTGCATTATAAGATTTAATATTTTGATAATAAATAACTAAACAATAA

GAAACTAACCCTAACCCATCTCATCCTAATAAAATCCTAATTAAATTAGGAGAAATAATCAATAACATTATAGATAAAACAAATATAGAAACTAATATAA

TAAATCGATGCAAATAAATATCTCCTTCTATATACTCTTCACTATAGTAAATTACTATAGAAGAAATAAATAAAACAAAACTCATAAATAATAATGACAT

CCAATCAAGTAAAATGGTTATAATAATTCTACAAGAATTAATCCTTAATATTTCATATTCTAATATTAGTCTATAATCTAAAATTATAAAATTTAAGCTT

AATAAAAATCTTAATACTCTAAAAAATAAAAACGTTACAAAATAAATTAAACAAATAGAAATAATTTAAAGTAAATTTTACATCTTTGATACCACAAATC

AATATTTTTTATTAAACTATTTAAATTACAATCATAAAACTAAAAATTCTCTCTTTAAAATTAAGATATTCAAGGGTAACCAATGAAGTAATAATAATAA

ATACTCACGAACAAACCCTCTTGAAAAAGAATATAAATTTCTAACTAACTTGCCGTGTTGACTATAAGAATATAAATATAAAGAATACGCAGCTCTAAAA

AAAGATATTAAAGATAAAAAAACCATTGTTCAACTCCTTCATCTAACTAATCTATTAATTAAAATAATCTCACCCAGTAAGTTTAATGAAGGAGGAGCCG

CTATATTACAACAACTAAATAAAAATCATCATATTCTTATTATTGGTATTAAATTGATTAGCCCCTTATTTAAATAAATACTTCGACTATTTAACCGTTC

ATAAGAAATATTTGCTAAACAAAATAATCCTGAAGAACATAAACCATGAGCAATTATTATAACTAAAGCCCCTCTTATACCCCAATAACTTAAAGTTAAA

ATCCCTCTTAATACTAAACCTATATGCGCTACAGAAGAATAAGCAATTAAAGCTTTAATATCAACTTGACGTAAACATATTAATGAAACAAAAAATCCCC

CTACTATTCTAATTGTAATAAAAATATAGTTTACTTGTAAACCTACAGTTAAAAAAATATTTATTAAACGTATTAATCCATACCCTCCTAATTTTAATAT

AACTCCAGCTAAAATTATAGAGCCAGCAACTGGAGCTTCAACATGGGCTTTAGGGAGCCAAAGATGAACAAAAAATATAGGCATTTTAATAAAAAACACT

ATATTTATACATATAAATAACAAGAATCTTTTTACATCATAACATAAAAAGAAAAAATCTAAAGAATGAAATTTTTCATAATAATAAAAAATCCTAATTA

TTATAGGTAATGAAGCAAATAATGTATAAAATAACAAGTAAACCCCTGCTTGCAAACGCTCAGGTTGATACCCCCAACCAATAATTAATAAAAGAGTGGG

AATTAAGCTAAACTCAAAAAACAAATAAAAAATAAATAAATTTAAAGAACTAAATGTTATAACTAAAGATAATAATAAAATAATTATTACTAATAAAAAT

AAATTATAAAAATAGTTTTTTCTATAAATACTTTCAGAAGCTAATAATATTAAAGAACAAATCCACAAACTTAATAAAATTATTATAAAAGATAAAAGGT

CATACCCTATAAAATAAGAAATATTTATATACAAATAATTAAAACTAAATCTTAACCCAAATATAAATGTAATAAAAAAATATATATATTGATTAAATCA

GTATCTCTTTTTAATACAACTTAAAGGTACCAATATTAATATTATAAAAATAAACTTTATCATAATACATTAAAAGTTTGAAAATAATCATTTCCATGTG

TTCGTATTATAGAAACTAATAACGATAAACCTAAAGCTCCTTCACAAACTCTTATAGTTAAAAATACTATACCAAAATAAAATTCAAAATTAAAATATAT

TAAAAATAGATATAAATTAAAATATAACCCTAAAATAATATACTCTAACCTTAATAATATTAAAAGTAAATGTTTACGTTTAATACAAAAAGAAACTAAT

CCTGTAAAATATATAATCACTGAAAATAACATACAAAAAATTAACATTAGTTTTAATAATTTAATAAAAATACTGGTCTTGTAAATCAGAAATAAGGATT

TTCTTTTAAAACTTCAGAGAAAGAGTAAACCTCTATCATTAATCTCCAAAATTAATATTTTAAATAAACTATTCTCTGTATAATCTTATTACTAATAACA

TTATCTTTTATTAGTTCAATAACTTTTATATTTTTAAGTCACCCTTTATCTATAGGGCTTATTTTATTAATACAAACTATTATTATAGCTTTAACTATAG

GTTTTTTTAATATTAATTTCTGATATTCTTATATTTTATTCCTTATTATAATTGGAGGAATATTAGTTTTATTTATTTATATAACAAGAGTAGCTTCAAA

TGAAAAATTTTCATTTTCTATTAAAATTACATTAATAATTAGAATTATAACTTTAGGATTTTTATTCAGTATTGCTATAATAGACCCCTACTTTTCAGAC

ATCAACTCAATTTACACAGAAAACTTAGATAATTATAAAGAATATAATATATCATTTAGAAAATATTTAAGTTATCCTAATATTATCATTATATACATAA

TAATTATTTATTTATTAATTACATTAATTGCAGTTGTAAAAATTACTCAAATTGAAAAAGGACCTTTACGTCAAACTAACTAATGAAAACACCATTACGA

AAAGCTTCCCCCCTATTAAAAATTATTAATAATAGAATTATTGATTTACCTACACCATCTAATATTTCTGCTTGATGAAATTTCGGATCCTTATTAGGAC

TCTGTCTTTTTATTCAAATTATCACTGGAATTTTCTTAGCTATACATTTTACAGCCCATATTGACATAGCATTTAATAGAGTAATTCATATTTGTCGAGA

TGTAAATTATGGCTGATTATTACGAACAATTCATGCTAACGGGGCTTCTTTTTTCTTTATTTGTATTTATCTACATATTGGCCGAGGAATATATTATAGA

AGCTATAATTTGCATTTAACATGAACTATTGGAGTAATCATCCTTTTTATAGTAATAGCTACAGCATTCTTAGGGTATGTATTACCTTGAGGGCAAATAT

CTTTTTGAGGGGCTACTGTTATTACCAATCTTCTCTCTGCTATTCCTTATCTAGGAAATATGATTGTCCAATGATTATGGGGGGGATTTGCAGTAGATAA

CGCCACTTTAACTCGATTTTTTACATTACATTTTATTCTTCCTTTTATTATTTTAGCTTTAATAATTATTCATTTATTATTTCTTCATCAAACTGGTTCC

AATAACCCTTTAGGATTAAATAGAAATATCGATAAAGTACCTTTCCATCCATATTTTACTTACAAAGATACTTTTGGATTCATTATAATAACTATATTAT

TAATATTTCTAGTTTTAATTAATCCCTATCTTTTAGGAGACCCAGAAAACTTTACACCCGCTAACCCTTTAGTAACCCCAGTCCATATCCAACCAGAATG

ATACTTTTTATTCGCATACGCTATTTTACGATCAATTCCTAATAAATTAGGTGGAGTAATTGCTTTAATTATATCAATTGCTATTTTATTAATTATACCT

TTAATTAACAAAAAAAAATTTAGTAGAACTCAATTTTACCCATTAAATAAAATTTTATTTTGATCTTTTGTCTCTATTGTAATCTTATTAACATGAATCG

GAGCTCGCCCAGTGGAAGATCCATACATTTTAACAGGTCAAATTTTAACAATTATATATTTTAGTTATTATTTCTTAAACCCCTTAATTCATAAAATATG

AGATTATATTATTTTTAAAACTTAGTTAATGAACTTGTTAAAGTGTATATTTTGAAAATATAAAAAAGAGTTTATTCTCTATTAACTTTACTAAATTTTA

TTCACTAAATAAAATAAGAAAAGATAACCAACTTTAAACCTAAAAAAAAAAATAAAAAATTTAAAGAAACTGGTAAATAACTTTTTCAACATATATATAT

TAACTTATCATAACGGTACCGAGGTAAAGTACCTCGAACTCAAATCCAAATAAATGCTATAAAAGTTAACTTCAAAAAAAAAAATAAAGAAAAAATATCT

CCCCCTATAAATAACAACACACATAATATACTTATAAATAAAATTCTAGCATACTCAGCCAAAAAGATTAAAGCAAATCCCCCTCTTCTGTACTCTACAT

TAAACCCAGAAACTAACTCTGATTCTCCTTCTGCAAAATCAAAAGGAGTCCGATTAGTCTCAGCTAAACTTGAAGATAATCATATTATTCTTAAAGGTAA

GCACAGAAAAAAAAATCATACTATTTCTTGATATTTTATAAAATCTATTATATTTAAATTTAAAATTAACAATAAAAAAGATAATAAAATTAATGATAAA

CTTACTTCGTATGAAATTGTTTGAGCTACTGATCGAATACCCCCTAATAAAGCATAATTAGAATTTGACGATCAACCAGAAATTATGATAGTATAGACCC

TTAACCTAGAACAACATAAAAAATATAAAATACCTAAATTAAACCTAAATATAAATGTCAAAAAAGGTATACATATCCATAATAAAAGGGCTAAAAATAA

ATTAAAAACTGGAGATATATAATAAATAAAAAAATTTGATATTAAAGGATAAGTTTGTTCTTTAGAAAATAACTTAATAGCATCACTAAAAGGTTGAGGA

ATACCTATAAATCCAACTTTATTGGGCCCTTTACGAATTTGGATATACCCTAAAACTTTACGCTCTATTAATGTTAAAAAAGCTACACCAATTAAAACAC

AAATAATTAAAACTAAACTCGTAAATAATAAAAGAAATAAATCTTGTAATATAATGTATTACTTGTGTTAAACACATATTTAAATTCTAAATTTAAAGCA

CTAATCTGCCAAAGTAATATTCATATTCAAATTATATTAAATTTTAAAGGTATCTGATCCTTTCGTACTAAAATACCTATATTTTTTAAAGATAGAAACC

AACCTGGCTCACGCCGGTTTAAACTCAGATCATGTAAAATTTTAAAGGTCGAACAGACCTAACCTTTTAGCCCCTACACCAAAAGTTAATTTTAATCCAA

CATCGAGGTCGCAAACTTTTTTTTCGATAAGAACTCTAAAAAAAAATTACGCTGTTATCCCTAAGGTAATTTAATCTTGTAATCATTAAAAATGGATCAT

TCAATCATAAATTAATGTTTTTAAATAAAAAAAGTTTAATCAATTTTTCTGCTGCCCCAGCAAAATAGTTTAAATTATTAAATATATAAATATGCTAAAA

TTAAATAATAATTTAAACTATAAAACTCTATAGGGTCTTCTCGTCTTTTAAAATTATATAAGCTTTTTTACTTATAAATAAAATTCTATATTCAATTAAA

TTGAGACAGTTACTTTCTCGTCCAACCGTTCATTCCAGCTTTCAATTAAAAAACTAATGATTATGCTACCTTTGCACGGTCAAATTACCGCGGCCATTCA

AATCCTCATTGGGCAGGTCAGACTTTAAATTATAATCAAAAAGACATGTTTTTAATAAACAGGCGAAAAGTGTATTTGCCGAGTTCCTTAATTTAACCTT

GAAGTTTTAATTTAATTACTAAATTAAAATATATACTAATTTTATCATTATTCTATACAAACCAATATTACATATATTATCTTAATAAACTACTTAAAAA

TAATATAAATCTTATTCTAACAAAAATTATTTATAACAAACTAAAGATTAACACTTCCAATTCTACTAATTTTTATTCAAAATATACATTTTTAACATTT

TATTTTAAAGCTTATCCCCTAAAATATTACTTTTTATATATAAAATACTATATAATTAATATAATACAATAAAAAAACTAAATTAAATTTATTTCTTAAG

AAACTAGATATCTTAAAAAACGTATAACGTTTCATTTCTAATATAATATTTTAAAAATTTATGCCACAATTAAATTTATATTATATTAGCTCTTTATAAT

TCGAGAACACTAAATAATTAAATTATTTTAATAAACCCTGATACACAAGGTACAAAAAATTAATTTTTCTTTTTAAAAAATAAATCTCTATATATTTATA

TTATCTATCTCTATACAAATTAACTATAATAAAATTTTTATATTCTAAAATATACTAATATCAAAAATATTTTTTTTATATATATATATATA----TTAT

AAATTTTTCCTTTCAAATTAAATTGATTTTCACAACTAACTTTTTAATGTAAATAAAATGCTTTTTTACAAGCTCTAATTTGCCATTCCAGGTACACTTT

CCAGTACACCTACTATGTTACGACTTATCCCTCTTTAGAGAGGGAGCGACGGGCGATATGTACATATTCTAGAGCTATACTCATATAATTAAACTAAACT

ATATTACTTTCAAATCCACTTTATAAAATAATGTTAATTATTTTAACCATCTAAATAATTTTATTGTAACCCATCTCTCCTTATCTATACGCTGTATCTT

GATCTGATTTTTTTTATACTTATAAATTTTGAACATTCCAAATTCTTTAAAAACATTCAACCTACGACGATATACAAACCTTTAAAATAAGTACGATTAA

TCGTGGACCATCAATTATAGGACAGGTTCCTCTGAGTAGACTAAAATACCGCCAAATTCTTAAAATTTCAAGAACATAACTACTACTATTCAAGCATCTA

AAATTTGCATTTTTAATAATAGGGTATCTAATCCTAGTTTTTTATAAAAATCTCATAAACTCATTTTTCACATTTAAAAAATTAATTATATTTACTAATT

TCACCTAATAAATACAACATAAATTAATAATAAAATAACTTATTATATACTGAACAAATTTAATTGTATTGTTTGTGTAACCGCAACTGCTGGCACAAAC

TTGGTCAATACTATTATAAATTCCTAAATCAAAATTTCTTTTAAATTTAATCTTCACTATTGCAATTCTTTAATTAAATATATAGAATAATTTATCTTTT

TAAAATAAATTCATTAAACACTAAAATTTACATATAAAATAATTTAAAAATTAAAATCTCAAGCTAGAATAAAACTTTATTTTTTTTTCAATATACATAA

ATTATATAAAATAAAAGTACCCCCC-TACCATTTTAATTAARTGAATTAATTTTTAATGCCTTAAATTTATTTCCAATTTATTTTTATTATATAGTTTTT

TATTTAAAAATAAAACTTTATACCTAAAATTATATACATAAATTATATTTAAAATTTATAATTTAAAGAACTATAAAAATTAAATTAAAATTTAAAATCT

ATCTAATTTATAGATAAACAAATTAAATTAAACTAACTTACCTGATAAATTTAACTATATACGCGCAGGTATATTAATTATTTAATTATAATATAATACA

TTTGTTTTATTAAATAATTATTAAGCAAAATAGCATTAATTTATTATATAAAAGATCTAGATACTATTAATTTAAAGTAAATTTTTTTTTTTTTTGTAGT

ATAATTTATACTAATATACGGCATTTAATATCTTCTATATAAATGTTTATATTATTTATATTTATCTATTTATAATTCTTAATAATAATTTAGATAATTA

TTAATTTATATATTAAATATTTATATATATATATATAATAATAAGGATATATATATATATATGTATATAGATAAATTAAATTTAGTTATATATAAAAAAA

TTAATATTAATTTTACTTTTAATTTTTTCTTTAATTTAAACTATTGGATATTCAGATTCTAATATATTATAATATATCAACCATTAATGAATAACTTTAT

ATATATAATAAAAATGTTAATTAATATATCAATTCATATGAATATATAATATTAATAAATAAATTCTTAATAAATAATAATGATAATATATATATATTGT

ATATTAATGTACTATATATATAAATTCATTAAACATTTATATTTGATACCCCCCCAAAATTTTTTCTAAAAATGTGAAGTTTCAATTTAATTAAAATTTT

CTACAATGGTCAAAAACGGCCAAAAAATGGAAAATAAAATGTGCACAGAAAATGCACATAAAATGCACATTTTTGAAAATTTCCGATTTTTGAAAAACCG

TTTGAACCAAACCTCTGATGTACCTTTTTTCGCCCCCCCTGATTACTAAAATTTATCCTCAATTACTAAGGTTTATCCGCATTTTTGCGACAGGAATTTT

GAGTATAAAATCAATTCCACACTAAATTTTTTTTT-TTTTTTTTGTGTCATTTCTCCAACATGGGCAATTTTTTTTTTAGTCGAAGGACAAATTTTAGTA

AAAAAAGTTTGAAATTAGGTTAAACCCTTGTTTAACCAAAAACTATTACTTTTCAATATTATACCCAATTTTATTTAAAATAAATTTTATTACACTTTTA

TTTATGCAAAAAGATCTTCTTATAAACTTTT

>DMR120j_JP_Fukuoka_Kyushu

AATGAAGTGTCTGACTATAGAGTTACTTTGATAGGGTAAAAAAAGTGAATTTTCACCTTCATTATAATTAACAGAATTAAACTATTTCTTTAAGCTTCAA

AAACTTATGTACATTATATACTAAATTATAAAAAGATAAGCTAATTAAGCTACTGGGCTCATACCCCATCAATAAAGGTTACAATCCTTTTCTTTTTAAT

GTATTATAAGCTTTTATTTTTTAATTCTCTTATAATTGGAACCTTAATTGCTATTTCTTCCTACTCTTGGATAGGAATATGAATAGGATTAGAAATTAAT

CTTCTCTCTATGATCCCCCTCATTAGAGACAACAAAAATATAATAGCCTCAGAAGCTGCTTTAAAATATTTCATTATTCAAACAATAGCATCAACATTGT

TATTATTCTCAATTATTATAATATCAATGAAATTTATATATCAAATAAATTTAATCACTTACTTTAATTTAATTTTTAACACTTCATTGTTCATCAAAAT

AGGAGCAGCCCCATTCCATTTTTGATTCCCCGAAATAATAGAAGGATTAAATTGATTAAATGCCATTATCATACTTACTTGACAAAAACTAAGGCCCATA

GTATTACTAACGTATTCTAATACAACCTCCATATATCTAATTTTAACAATTATATTTAGAATAATAATCAGAGGAATTATAGGCCTAAATCAAACTAGAT

TACGGAAAATTATAGCTTATTCATCTATCAACCATATTGGATGAATAATCAGTTCAATTATATTAATTGAAATTGTTTGATTTTACTATTTTATTATTTA

TTGCATTATTACTATTAATATCAGAATTATATTTATAAAATTAAATGTTTTTCATATCAATCAATTATATATTTCAATAAATTACCATATTTTACTTAAA

TTATTCTTTGCCTTAAATTTCATATCTTTAGGAGGATTACCCCCATTTTTAGGATTTTTCCCTAAATGACTTACAATTCAAACTTTAATCCAAAGAAATA

TGTACTCAATTGCTTTCATTATAATTCTAATAACTTTAATAACACTTTTTTTTTACCTTCGAATTACTTTTTCAATTTTACTACTAAGAAAATCAGTTTT

AACATTTTACACACAACCAAAAATTGATACTAATTACATTATAGCATTTAATTTTATTACATTATTAAGATTAATTTTCGTTACTTTGATATTCAATTTC

TTATAAATTAAACCTGAAGGATTTAAGTTAAATTAAACTAAGAACCTTCAAAGTTCTAAATAAAGTAAATTCTTTAAGCCTTAGGGCTTAGCCCATCTTT

AAATTTGCAATTTAAAATTCTTTTTGAACTATAAAGCTTGATAAAAGAAACTAATTTCGTATGTAAATTTACAGTTTACCGCCTAAACCTCGGCCATTTT

ATCGAATAAATGGCTATTCTCTACAAATCACAAAGATATCGGAACTTTATACTTTCTATTTGGAAGTTGAGCAGGAATAGTAGGTACTTCCTTAAGTTTA

CTGATTCGTGCTGAATTGGGAAACCCAGGATCTCTAATTGGAGATGATCAAATTTATAACGTAATCGTAACAGCTCATGCTTTCATTATAATTTTTTTTA

TAGTAATACCTATTATAATTGGAGGATTCGGAAACTGATTAGTTCCTTTAATGCTTGGAGCCCCCGACATAGCATTTCCCCGAATAAATAACATAAGATT

TTGACTTTTACCTCCTTCATTAACTCTACTTCTAATAAGAAGATTAGTCGAAAGAGGAGCAGGTACAGGCTGAACAGTTTATCCCCCACTATCAGCCAAT

ATCGCCCATAGAGGAGCATCAGTTGATTTAGCAATTTTTAGACTTCATCTTGCAGGAATTAGCTCAATTTTAGGTGCAGTAAATTTTATTACTACTGTAA

TTAACATACGATCAACAGGAATAACTTTTGATCGAATACCCCTATTTGTTTGATCTGTAGTTTTAACAGCACTTCTTCTGCTATTATCCCTCCCAGTTTT

AGCAGGAGCAATTACTATACTATTAACAGATCGAAATATTAATACAACATTTTTTGATCCCGCAGGTGGAGGAGACCCCATTCTCTACCAACATTTATTT

TGATTTTTTGGTCATCCAGAAGTTTACATTTTAATTTTACCTGGATTTGGTATAATCTCCCATATTATTAGCCAAGAAAGAAGAAAAAAAGAAACATTTG

GAACTTTAGGTATAATTTACGCTATAATAGCTATTGGTTTACTAGGATTTATTGTTTGAGCTCACCATATATTTACAGTAGGTATAGATGTAGACACACG

AGCATATTTTACATCAGCAACAATAATCATTGCTGTTCCTACAGGAATTAAAATTTTTAGATGATTAGCTACTCTTCATGGATCACAATTAAACTACTCC

CCGTCTCTTTTATGGGCATTAGGATTTGTATTCCTATTTACAGTAGGAGGATTAACAGGAGTAATTCTAGCTAATTCATCAATTGACATTATTTTACATG

ATACTTACTATGTAGTTGCACATTTCCATTATGTCCTTTCCATAGGAGCTGTATTTGCTATTATAGCAGGTTTTGTTCATTGATTCCCTTTATTCACAGG

TTTAACAATAAATTCAAAATTTCTTAAAATTCAATTTTTAACAATATTTATTGGTGTCAATATAACATTCTTCCCTCAACATTTCTTAGGATTAAGAGGA

ATACCTCGACGTTATTCAGATTACCCAGATGCTTACACAACTTGAAATATTATCTCATCTATTGGATCTTTAGTTTCTTTAATTAGTATTTTTATCTTTT

TATTTACTATTTGAGAAAGGCTAATTTCATTACGAAAAAGAATTAGGTCTTTAAGAATATCTACATCAATTGAATGACTCCAACAAATACCCCCTTCAGA

ACATAGTTATTCTGAACTTCCAATGCTTACTAACTTCTAATATGGCAGATTAGTGCAATGGATTTAAACCCCAAATATAAAGATTAAACTTTTTTTAGAA

ATAGCTACTTGAAACACCATTTTACTTCAGGATAGGGCATCCCCATTAATAGAACAACTCTCATTCTTTCATAACCATGCTCTTCTAATTCTCTTTATAA

TTACCGTTCTAGTAGGTTATTTAATAGGAACTTTATTTTTTAACCAATTTAATTACCGATTTTTATTAGATGGTCAAACTATTGAAATTATTTGAACTAT

TTTACCTGCTGTAACACTAATTTTTATCGCATTACCGTCTTTACGCTTACTTTATCTTCTAGATGAAGTTAATAATCCTTTAGTAACTATCAAAACAATT

GGGCATCAATGATATTGATCATACGAATATAGAGATTTTATAAATTTTGAATTCGATTCCTATATAATTCCTTTGACAGAAATAAAACCTCAAAATTTTC

GTTTATTAGATGTTGATAACCGAGTAATTGTCCCCTTTAACTCCCAAATTCGAATGATAGTAACAGCTGCCGATGTTATTCATTCATGAACTATCCCGGC

TTTTAGTGTAAAAATTGATGCAACACCGGGCCGACTAAATCAAATTAGATTTCTAATTAATCGAACAGGATTATTTTATGGTCAATGCTCAGAAATTTGT

GGAGCAAATCATAGATTTATACCTATTACTGTAGAAAGAATTTCACCTTCATTTTTTACTAAATGAATCTCAAAAATAAATAACCTATCATTAGATGACT

GAAAGTAAGTAATGGTCTCTTAAACCAATTAATAGTAGTTTAACATCTACTTCTGATGGCCAAAAATTTAGTTAAGATATAACATTAGTTTGTCATACTA

AAATAATCATAATTTGATAATTTTTAATTCCACAAATAGCACCTTTAAACTGACTATCTTTATTTTTTTTAATTATTATTATTTTTTTACTTTTTAATGT

ATTAAATTACTTTAGATTCTTACAGCCCTTAAAAACCCAATCTCATAGCCCTACAATTAAAAAAATTAATTGAAAATGATAACTAATTTATTTTCATCTT

TTGATCCTAGAACTTCTTTTAATTTAAGATTAAACTGATTAAGAATACTATTAGGGCTAATATTTATCCCCCCAATATTTTGATTAGTTCCTTCACGCCA

TAATTTTCTATGAATTAAAATTATTTTAACATTACACCAAGAATTTAAGGTTTTAATTGGTAATAATAATATTAAAGGAAGAACCTTAATATTTATTTCA

TTATTTTCTATAATTGTTTTCAATAACTTTTTAGGATTATTTCCGTATATTTTTACAGGAACAAGACATTTAATTATAACATTATCTCTTGCCTTACCTT

TATGAATTAGATTCATATTATACGGGTGAATTAATAACACTATCCACATACTTGCTCATTTAGTTCCCCAAGGAACACCCCCAGCTCTTATGGCATTCAT

AGTAGTAATTGAATCAATTAGAAATATTATTCGTCCTGGTACTTTAGCTGTTCGATTAGCTGCTAATATAATTGCTGGACATTTACTAATAACTTTACTA

GGAAACACAGGATTAAATTTATCAATTTTTATACTAAGTATTCTTATTATCACACAAATTCTTTTATTAATTTTAGAATCTGCTGTTGCGATCATTCAAT

CTTATGTATTTGCTGTATTAAGAACTTTATACTCTAGAGAAATTAATTAATGTCAAGACATAAAAATCACCCTTATCATTTAGTTGATGCAAGACCTTGA

CCTATTTTAGGTGCTTTTAGAGCTATAATTACAATAATTGGAATTATCAAATGATTCCATTTTTATAATAATTCTTTATTTTACTTAGGGACATTAATCA

CAATTTTAATTATAATTCAATGATGACGAGATATCACTCGTGAGGGAACTTTCCAAGGACTTCATACTTACGCTGTAACTATAGGTTTACGTTGAGGAAT

AATTTTATTTATTACATCAGAAGTATTTTTCTTTATTTCTTTTTTTTGAGCCTTTTTTCATAGTAGTTTAACACCCGCTATTGAACTAGGGATACTCTGA

CCACCCAAAGGAATTACCCCATTTAACCCAATTCAAATTCCATTATTAAACACTTTAATTCTTTTAACTTCAGGATTAACTGTAACTTGAGCTCATCATA

GATTAATAGAAAATGACTATAACCAAACAATGCAAGGTCTTGGTTTAACAGTTTTACTAGGAGTATATTTTACTTTATTACAAGGTTACGAATATTTAGA

AGCCCCCTTTACTATAGCAGATTCTGTTTATGGATCAACATTTTTTATTGCTACTGGTTTCCATGGATTACATGTTATTATTGGCACAACCTTTTTAGCT

GTTTGTTTAATACGACATTTTAATAACCATTTTACTTGTATCCATCACTTTGGATTTGAAGCTGCTGCTTGATACTGACATTTTGTTGATGTAGTATGAC

TATTTCTTTATATTTCTATTTACTGATGAGGTAGATATTTATATAGTATAATAATTATAATTGATTTCCAATCAAAAGATCTAAAAAAA-TTAGTATAAA

TAATCATTATAATTTGAAATATAGGGTTAATTATTTTTTCTATCTCTTTTATCTTAATTATACTATCTTTTACAATCTCTAAAAAAAGATTTATAGACCG

AGAAAAAGCTTCTCCATTCGAGTGCGGATTTGACCCCAAAAGATCAGCCCGTTTACCCTTTTCTTTGCATTTTTTTTTAATTGCAGTAATTTTCTTAATT

TTTGATGTTGAAATTACTCTTCTTATCCCTTTAATTCTAACAATAAAAATTACTAATATTACTATATATACCTATATTGCTCTTTTCTTTTTAATGATTC

TTTTAATAGGACTTTACCATGAATGAAACCAGGGGGCCTTAAATTGAGCTCTTTAGGGTAATAGTTAAGTATAACATTTAAGTTGCATTTAAAAAGTATT

GATTTTTCAATTTACCTTAAATAAGAAACAATTAATTGTATTTAGTTTCGACCTAAAATTTAGGTGTATGAACACCCTTATTTAAATTAATTGAAACCAA

AAAGAGGTATATCACTGTTAATGATACTAATGAGAAAAACTCCAATTAAGGAAATAAGATACTCAAGAGTAAGCTTCTAACTTAACTCTTTAGCAGTGAA

AGTCTGTTAATATTTCTATTTATATAGTTTAATAAAACATTATTTTTTCATAATAAAATTAGAATAAATTTATTCTTATAAATATTTAAAAGTAAATTTT

ACTTCCCTGATAACTTCACTATCATACTCTATATAAGCTATTTAAATTAAATATATAAAATTATAAAAATTACCCATATTATAATTAAAAGTATAAAAAT

CTTATAATTGTTATTAAATATAAACTGTAAAAATACAGAAGTATTACTAATTTTACTATATAAATTTTGTCTTCCATAATACTCTGATCAGCCTTGATCA

ATAGTTTTATATAACTTACTACCTAATTTAATTGGATAATAATTTAAACCAAATGTTGAAATATAGGGTATATTCCACATAGAAGAAAAAAAAAGTCTTG

AATTTAACAAATAAAAAGATTTTAATCTATCATTTAAAGTAAACTTAGAAAGTTCAAACCCAAACCAAGCCCCAAAAAATGATACAATTAAAGCTATAAT

TTTTATTGTAAAAGGTAAACAAATAAAATAAGGTGTTGGGAATATCAACCATATTAATATTCTTCCCCCAACAATAACTAAAAAAATTAAACCTGATATC

CCTTGAAGTATAATTTTTCTATTATCATTAATTTTACTTAAAGAATAAAAACAAAAATTTCCTACCAAAACATAATAAATTAAACGAAATGTATAACAAA

CAGTTAATCCTGTTGAAAAAAAGAAAATAACATAAATATAGATATTTAAGTATCTTATAGATAAAACCTCTAAAATTAAATCCTTTGAATAAAACCCCGA

TAAAAAGGGTAAACCGCATAATGCTAAATTTGAAATTATAAAATAAGTACAAGTTAAAGGTATAACTTTAATTAACCCTCCTATATACCGAATATCTTGA

CAATTTCTTAATCTGTGAATTATACACCCTGCGCATATAAATAATAAAGCCTTAAATAAAGCGTGAGTTAATAGGTGAAAAAAAGCTAATTGATACTCCC

CTAAAGCTAAAATACTAATTATCAAACCCAATTGTCTTAAAGTAGACAGAGCAATAATTTTTTTTAAATCAAACTCGAAGTTTGCGCCTATTCCTGCTAT

AAATATAGTTATTGTTCCAATAAATAATAAAATTAATATTAAATTACTTGTTAATGCAAAATTAAAACGAATTAATAAGTAAACACCTGCTGTTACTAAG

GTAGAAGAATGAACTAAAGAAGAAACAGGTGTTGGAGCTGCTATTGCCGCAGGCAACCAAGAAGAAAATGGAATTTGAGCTCTTTTAGTTATAGCTGCTA

ATATAATTAATAAAATAATAATATATATTTCTATACTATTTTTATATACATCAATATAAAAAATATAGTTAAATCCCCCAAAATTTATTATTCATGCAAT

TGCTATTAATAAAGCAACATCTCCAATACGGTTAGTTAAAGCTGTAATTATCCCTGCATTATAAGATTTAATATTTTGATAATAAATAACTAAACAATAA

GAAACTAACCCTAACCCATCTCATCCTAATAAAATCCTAATTAAATTAGGAGAAATAATCAATAACATTATAGATAAAACAAATATAGAAACTAATATAA

TAAATCGATGCAAATAAATATCTCCTTCTATATACTCTTCACTATAGTAAATTACTATAGAAGAAATAAATAAAACAAAACTCATAAATAATAATGACAT

CCAGTCAAGTAAAATGGTTATAATAATTCTACAAGAATTAATCCTTAATATTTCATATTCTAATATTAGCCTATAATCTAAAATTATGAAATTTAAGCTT

AATAAAAATCTTAATACTCTAAAAAATAAAAACGTTACAAAATAAATTAAACAAATAGAAATAATTTAAAGTAAATTTTACATCTTTGATACCACAAATC

AATATTTTTTATTAAACTATTTAAATTACAATCATAAAACTAAAAATTCTCTCTTTAAAATTAAGATATTCAAGGGTAACCAATGAAGTAATAATAATAA

ATACTCACGAACAAACCCTCTTGAAAAAGAATATAAATTTCTAACTAACTTGCCGTGTTGACTATAAGAATATAAATATAAAGAATACGCAGCTCTAAAA

AAAGATATTAAAGATAAAAAAACCATTGTTCAACTCCTTCATCTAACTAATCTATTAATTAAAATAATCTCACCCAGTAAGTTTAATGAAGGAGGAGCCG

CTATATTACAACAACTAAATAAAAATCATCATATTCTTATTATTGGTATTAAATTGATTAGCCCCTTATTTAAATAAATACTTCGGCTATTTAACCGTTC

ATAAGAAATATTTGCTAAACAAAATAATCCTGAAGAACATAAACCATGGGCAATTATTATAACTAAAGCCCCTCTTATACCCCAATAACTTAAAGTTAAA

ATCCCTCTTAATACTAAACCTATATGCGCTACAGAAGAATAAGCAATTAAAGCTTTAATATCAACTTGACGTAAACATATTAATGAAACAAAAAATCCCC

CTACTATTCTAATTGTAATAAAAATATAGTTTACTTGTAAACCTACAGTTAAAAAAATATTTATTAAACGTATTAATCCATACCCCCCTAATTTTAATAT

AACTCCAGCTAAAATTATAGAGCCAGCAACTGGAGCTTCAACATGGGCTTTAGGGAGCCAAAGATGAACAAAAAATATAGGCATTTTAATAAAAAACACT

ATATTTATACATATAAATAACAAGAATCTTTTTACATCATAACATAAAAAGAAAAAATCTAAAGAATGAAATTTTTCATAATAATAAAAAATCCTAATTA

TTATAGGTAATGAAGCAAATAATGTATAAAATAACAAGTAAACACCTGCTTGCAAACGCTCAGGTTGATACCCCCAACCAATAATTAATAAAAGAGTGGG

AATTAAGCTAAACTCAAAAAACAAATAAAAAATAAATAAATTTAAAGAACTAAATGTTATAACTAAAGATAATAATAAAATAATTATTACTAATAAAAAT

AAATTATAAAAATAGTTTTTTCTATAAATACTTTCAGAAGCTAATAATATTAAAGAACAAATCCACAAACTTAATAAAATTATTATAAAAGATAAAAGGT

CATACCCTATAAAATAAGAAATATTTATATACAAATAATTAAAACTAAATCTTAACCCAAATATAAATGTAATAAAAAAATATATATATTGATTAAATCA

GTATCTCTTTTTAATACAACTTAAAGGTACCAATATTAATATTATAAAAATAAACTTTATCATAATACATTAAAAGTTTGAAAATAATCATTTCCATGTG

TTCGTATTATAGAAACTAATAACGATAAACCTAAAGCTCCTTCACAAACTCTTATAGTTAAAAATACTATACCAAAATAAAATTCAAAATTAAAATATAT

TAAAAATAGATATAAATTAAAGTATAATCCTAAAATAATATACTCTAACCTTAATAATATTAAAAGTAAATGTTTACGTTTAATACAAAAAGAAACTAAT

CCTGTAAAATATATAATCACTGAAAATAACATACAAAAAATTAACATTAGTTTTAATAATTTAATAAAAATACTGGTCTTGTAAATCAGAAATAAGGATT

TTCTTTTAAAACTTCAGAGAAAGAGTAAACCTCTATCATTAATCTCCAAAATTAATATTTTAAATAAACTATTCTCTGTATAATCTTATTACTAATAACA

TTATCTTTTATTAGTTCAATAACTTTTATATTTTTAAGTCACCCTTTATCTATAGGGCTTATTTTATTAATACAAACTATTATTATAGCTTTAACTATAG

GTTTTTTTAATATTAATTTCTGATATTCTTATATTTTATTCCTTATTATAATTGGGGGAATATTAGTTTTATTTATTTATATAACAAGAGTAGCTTCAAA

TGAAAAATTTTCATTTTCTATTAAAATTACATTAATAATTAGAATTATAACTTTAGGATTTTTATTCAGTATTGCTATAATAGACCCTTACTTTTCAGAC

ATCAACTCAATTTACACAGAAAACTTAGATAATTATAAAGAATATAATATATCATTTAGAAAATATTTAAGTTATCCTAATATTATCATTATATACATAA

TAATTATTTATTTATTAATTACATTAATTGCAGTTGTAAAAATTACTCAAATTGAAAAAGGACCTTTACGTCAAACTAACTAATGAAAACACCATTACGA

AAAGCTTCCCCCCTATTAAAAATTATTAATAATAGAATTATTGATTTACCTACACCATCTAATATTTCTGCTTGATGAAATTTCGGATCCTTATTAGGAC

TCTGTCTTTTTATTCAAATTATCACTGGAATTTTCTTAGCTATACATTTTACAGCCCATATTGACATAGCATTTAATAGAGTAATTCATATTTGTCGAGA

TGTAAATTATGGCTGATTATTACGAACAATTCATGCTAACGGGGCTTCTTTTTTCTTTATTTGTATTTATCTACATATTGGCCGAGGAATATATTATAGA

AGCTATAATTTACATTTAACATGAACTATTGGAGTAATCATCCTTTTTATAGTAATAGCTACAGCATTCTTAGGGTATGTATTACCTTGAGGGCAAATAT

CTTTTTGAGGGGCTACTGTTATTACCAATCTTCTCTCTGCTATTCCTTATCTAGGGAATATGATTGTCCAATGATTATGGGGGGGATTTGCAGTAGATAA

CGCCACTTTAACTCGATTTTTTACATTACATTTTATTCTTCCTTTTATTATTTTAGCTTTAATAATTATTCATTTATTATTTCTTCATCAAACTGGTTCC

AATAACCCTTTAGGATTAAATAGAAATATCGATAAAGTACCTTTCCATCCATATTTTACTTACAAAGATACTTTTGGATTCATTATAATAACTATATTAT

TAATATTTCTAGTTTTAATTAATCCCTATCTTTTAGGAGACCCAGAAAACTTTACACCCGCTAATCCTTTAGTAACCCCAGTCCATATCCAACCAGAATG

ATACTTTTTATTCGCATACGCTATTTTACGATCAATTCCTAATAAATTAGGTGGAGTAATTGCTTTAGTTATATCAATCGCTATTTTATTAATTATACCT

TTAATTAACAAAAAAAAATTTAGTAGAACTCAATTTTACCCATTAAATAAAATTTTATTTTGATCTTTTGTCTCTATTGTAATCTTATTAACATGAATCG

GAGCTCGCCCAGTGGAAGATCCATACATTTTAACAGGTCAAATTTTAACAATTATATATTTTAGTTATTATTTCTTAAACCCCTTAATTCATAAAATATG

AGATTATATTATTTTTAAAACTTAGTTAATGAACTTGTTAAAGTGTATATTTTGAAAATATAAAAAAGAGTTTATTCTCTATTAACTTTACTAAATTTTA

TTCACTAAATAAAATAAGAAAAGATAGCCAACTTTAAACCTAAAAAAAAAAATAAAAAATTTAAAGAAACTGGTAAATAACTTTTTCAACATATATATAT

TAACTTATCATAACGGTACCGAGGTAAAGTACCTCGAACTCAAACCCAAATAAATGCTATAAAAGTTAACTTCAAAAAAAAAAATAAAGAAAAAATATCC

CCCCCTATAAATAACAACACACATAATATACTTATAAATAAAATTCTAGCATACTCAGCCAAAAAGATTAAAGCAAATCCCCCTCTTCTGTACTCTACAT

TAAACCCAGAAACTAATTCTGATTCTCCTTCTGCAAAATCAAAAGGAGTCCGATTAGTCTCAGCTAAACTTGAAGATAATCATATTATTCTTAAAGGTAA

GCACAGAAAAAAAAATCATACTATTTCTTGATATTTTATAAAATCTATTATATTTAAATTTAAAATTAACAATAAAAAAGATAATAAAATTAATGATAAA

CTTACTTCGTATGAAATTGTTTGAGCTACTGATCGAATACCCCCTAATAAAGCATAATTAGAATTTGACGATCAACCAGAAATTATGATAGTATAGACCC

TTAACCTAGAACAACATAAAAAATATAAAATACCTAAATTAAACCTAAATATAAATGTCAAAAAAGGTATACATATCCATAATAAAAGAGCTAAAAATAA

ATTAAAAACTGGAGATATATAATAAATAAAAAAATTTGATATTAAAGGATAAGTTTGTTCTTTAGAAAATAACTTAATAGCATCACTAAAAGGTTGAGGA

ATACCTATAAATCCAACTTTATTGGGCCCTTTACGAATTTGGATATACCCTAAAACTTTACGCTCTATTAATGTTAAAAAAGCTACACCAATTAAAACAC

AAATAATTAAAACTAAACTTGTAAATAATAAAAGAAATAAATCTTGTAATATAATGTATTACTTGTGTTAAACACATATTTAAATTCTAAATTTAAAGCA

CTAATCTGCCAAAGTAATATTCATATTCAAATTATATTAAATTTTAAAGGTATCTGATCCTTTCGTACTAAAATACCTATGTTTTTTAAAGATAGAAACC

AACCTGGCTCACGCCGGTTTAAACTCAGATCATGTAAAATTTTAAAGGTCGAACAGACCTAACCTTTTAGCCCCTACACCAAAAGTTAATTTTAATCCAA

CATCGAGGTCGCAAACTTTTTTTTCGATAAGAACTCTAAAAAAAAATTACGCTGTTATCCCTAAGGTAATTTAATCTTGTAATCATTAAAAATGGATCAT

TCAATCATAAATTAATGTTTTTAAATAAAAAAAGTTTAATCAATTTTTCTGCTGCCCCAGCAAAATAGTTTAAATTATTAAATATATAAATATACTAAAA

TTAAATAATAATTTAAACTATAAAACTCTATAGGGTCTTCTCGTCTTTTAAAATTATATAAGCTTTTTTACTTATAAATAAAATTCTATATTCAATTAAA

TTGAGACAGTTACTTTCTCGTCCAACCGTTCATTCCAGCTTTCAATTAAAAAACTAATGATTATGCTACCTTTGCACGGTCAAATTACCGCGGCCATTCA

AATCCTCATTGGGCAGGTCAGACTTTAAATTATAATCAAAAAGACATGTTTTTAATAAACAGGCGAAAAGTGTATTTGCCGAGTTCCTTAATTTAACCTT

GAAGTTTTAATTTAATTACTAAATTAAAATATATACTAATTTTATCATTATTCTATATAAACCAATATTACATATATTATCTTAATAAACTACTTAAAAA

TAATATAAATCTTATTCTAACAAAAATTATTTATAACAAACTAAAGATTAACACTTCCAATTCTACTAATTTTTATTCAAAATATACATTTTTAACATTT

TATTTTAAAGCTTATCCCCTAAAATATTACTTTTTATATATAAAATACTAAATAATTAATATAATACAATAAAAAAACTAAATTAAATTTATTTCTTAAG

AAACTAGATATCTTAAAAAACGTATAACGTTTCATTTCTAATATAATATTTTAAAAATTTATGCCACAATTAAATTTATATTATATTAGCTCTTTATAAT

TCGAGAACACTAAATAATTAAATTATTTTAATAAACCCTGATACACAAGGTACAAAAAATTAATTTTTCTTTTTAAAAAATAAATCTCTATATATTTATA

TTATCTATCTCTATACAAATTAACTATAATAAAATTTTTATATTCTAAAATATACTAATATCAAAAATATTTTTTTTATATATATATATATATA--TTAT

AAATTTTTCCTTTCAAATTAAATTGATTTTCACAACTAACTTTTTAATGTAAATAAAATGCTTTATTACAAGCTCTAATTTGCCATTCCAGGTACACTTT

CCAGTACACCTACTATGTTACGACTTATCCCTCTTTAGAGAGGGAGCGACGGGCGATATGTACATATTCTAGAGCTATACTCATATAATTAAACTAAACT

ATATTACTTTCAAATCCACTTTATAAAATAATGTTAATTATTTTAACCATCTAAATAATTTTATTGTAACCCATCTCTCCTTATCTATACGCTGTATCTT

GATCTGATTTTTTTTATACTTATAAATTTTGAACATTCCAAATTCTTTAAAAACATTCAACCTACGACGATATACAAACCTTTAAAATAAGTACGATTAA

TCGTGGACCATCAATTATAGGACAGGTTCCTCTGAGTAGACTAAAATACCGCCAAATTCTTAAAATTTCAAGAACATAACTACTACTATTCAAGCATCTA

AAATTTGCATTTTTAATAATAGGGTATCTAATCCTAGTTTTTTATAAAAATCTCATAAACTCATTTTTCACATTTAAAAAATTAATTATACTTACTAATT

TCACCTAATAAATACAATATAAATTAATAATAAAATAACTTATTATATACTGAACAAATTTAATTGTATTGTTTGTGTAACCGCAACTGCTGGCACAAAC

TTGGTCAATACTATTATAAATTCCTAAATCAAAATTTCTTTTAAATTTAATCTTCACTATTGCAATTCTTTAATTAAATATATAGAATAATTTATCTTTT

TAAAATAAATTCATTAAACACTAAAATTTACATATAAAATAATTTAAAAATTAAAATCTCAAGCTAGAATAAAACTTTATTTTTTTTTCAATATACATAA

ATTATATAAAATAAAAGTACCCCCC-TACTATTTTAATTAAATGAATTAATTTTTAATGCCTTAAATTTATTTCCAATTTATTTTTATTATATAGTTTTT

TATTTAAAAATAAAACTTTATACCTAAAATTATATACATAAATTATATTTAAAATTTATAATTTAAAGAACTATAAAAATTAAATTAAAATTTAAAATCT

ATCTAATTTATAGATAAACAAATTAAATTAAACTAACTTACCTGATAAATTTAACTATATACGCGCAGGTATATTAATTATTTAATTATAATATAATACA

TTTGTTTTATTAAATAATTATTAAGCAAAATAGCATTAATTTATTATATAAAAGATCTAGATACTATTAATTTAAAGTAAATTTTTTTTTTTTTTGTAGT

ATAATTTATACTAATATACGGCATTTAATATCTTCTATATAAATGTTTATATTATTTATATTTATCTATTTATAATTCTTAATAATAATTTAGATAATTA

TTAATTTATATATTAAATATTTATATATATATATATAATAATAAGGATATATATATATATATGTATATAGATAAATTAAATTTAGTTATATATAAAAAAA

TTAATATTAATTTTACTTTTAATTTTTTCTTTAATTTAAACTATTGGATATTCAGATTCTAATATATTATAATATATCAACCATTAATGAATAACTTTAT

ATATATAATAAAAATGTTAATTAATATATCAATTCATATGAATATATAATATTAATAAATAAATTCTTAATAAATAATAATGATAATATATATATATTGT

ATATTAATGTACTATATATATAAATTCATTAAACATTTATATTTGATACCCCCCCAAAATTTTTTCTAAAAATGTGAAGTTTCAATTTAATTAAAATTTT

CTACAATGGTCAAAAACGGCCAAAAAATGGAAAATAAAGTGTGCACAGAAAATGCACATAAAATGCACATTTTTGAAAATTTCCGATTTTTGAAAAACCG

TTTGAACCAAACCTCTGATGTACCTTTTTTCGCCCCCCCTGATTACTAAAATTTATCCTCAATTACTAAGGTTTATCCGCATTTTTGCGACAGGAATTTT

GAGTATAAAATCAATTCCACACTAAATTTTTTTTT-TTTTTTTTGTGTCATTTCTCCAACATGGGCAATTTTTTTTTTAGTCGAAGGACAAATTTTAGTA

AAAAAAGTTTGAAATTAGGTTAAACCCTTGTTTAACCAAAAACTATTACTTTTCAATATTATACCCAATTTTATTTAAAATAAATTTTATTATACTTTTA

TTTATGCAAAAAGATCTTCTTATAAACTTTT

>DMR122j_JP_Fukuoka_Kyushu

AATGAAGTGTCTGACTATAGAGTTACTTTGATAGAGTAAAAAAAGTGAATTTTCACCTTCATTATAATTAACAGAATTAAACTATTTCTTTAAGCTTCAA

AAACTTATGTACATTATATACTAAATTATAAAAAGATAAGCTAATTAAGCTACTGGGCTCATACCCCATCAATAAAGGTTACAATCCTTTTCTTTTTAAT

GTATTATAAGCTTTTATTTTTTAATTCTCTTATAATTGGAACCTTAATTGCTATTTCTTCCTACTCTTGGATAGGAATATGAATAGGATTAGAAATTAAT

CTTCTCTCTATGATCCCCCTCATTAGAGACAATAAAAATATAATAGCCTCAGAAGCTGCTTTAAAATATTTCATTATTCAAACAATAGCATCAACATTGT

TATTATTCTCAATTATTATAATATCAATGAAATTTATATATCAAATAAATTTAATCACTTACTTTAATTTAATTTTTAACACTTCATTATTCATCAAAAT

AGGAGCAGCCCCATTCCATTTTTGATTCCCCGAAATAATAGAAGGATTAAATTGATTAAATGCCATTATCATACTTACTTGACAAAAACTAAGACCTATA

GTATTATTAACATATTCTAATACAACCTCCATATATCTAATTTTAACAATTATATTTAGAATAATAATCAGAGGAATTATAGGTCTAAATCAAACTAGAT

TACGAAAAATTATAGCTTATTCATCTATCAACCATATTGGATGAATAATCAGTTCAATTATATTAATTGAAATTGTTTGATTTTACTATTTTATTATTTA

CTGCATTATTACTATTAATATCAGAATTATATTTATAAAATTAAATGTTTTTCATATCAATCAATTATATATTTCAATAAATTACCATATTTTACTTAAA

TTATTCTTTGCCTTAAATTTCATATCTTTAGGAGGATTACCTCCATTTTTAGGATTTTTCCCTAAATGACTTACAATTCAAACTTTAATCCAAAGAAATA

TACACTCAATTGCTTTCATTATAATTTTAATAACTTTAATAACACTTTTTTTTTACCTCCGAATTACTTTTTCAATTTTATTACTAAGAAAAACAGTTTT

AACATTTTACACACAACCAAAAATTTATACTAATTACATTATAGCATTTAATTTTATTACATTATTAAGATTAATTTTCGTTACTTTGATATTCAATTTC

TTATAAATTAAACATGAAGGATTTAAGTTAAATTAAACTAAGAACCTTCAAAGTTCTAAATAAAGTAAATTCTTTAAGCCTTAGGGCTTAGCCCATCTTT

AAATTTGCAATTTAAAATTCTTTTTGAACTATAAAGCTTGATAAAAGAAACTAATTTCGTATGTAAATTTACAGTTTACCGCCTAAACCTCGGCCATTTT

ATCGAATAAATGGCTATTCTCTACAAATCACAAAGACATCGGAACTTTATACTTTCTATTTGGAAGTTGGGCAGGTATAGTAGGCACTTCATTAAGTTTA

CTGATTCGTGCTGAATTAGGAAACCCGGGATCTCTAATTGGAGATGACCAAATTTATAACGTAATCGTTACAGCTCATGCTTTCATTATAATTTTTTTTA

TAGTAATACCTATTATAATTGGAGGATTTGGAAATTGATTAGTTCCTTTAATGCTTGGAGCCCCTGACATAGCATTCCCTCGAATAAATAACATAAGATT

TTGACTTTTACCTCCTTCATTAACTCTACTTCTAATAAGAAGATTAGTCGAAAGAGGAGCAGGTACAGGCTGAACAGTTTATCCCCCACTATCAGCCAAT

ATTGCCCATAGAGGAGCATCAGTTGATTTAGCAATTTTTAGACTTCATCTTGCAGGAATTAGTTCAATTTTAGGCGCAGTAAATTTCATTACCACTGTAA

TTAACATGCGATCAACAGGAATAACTTTTGATCGAATACCCTTATTTGTTTGATCTGTAGTTTTAACAGCACTTCTTCTGCTATTATCTCTCCCCGTTTT

AGCAGGAGCAATTACTATACTATTAACAGATCGAAATATTAATACAACATTTTTTGATCCCGCAGGTGGAGGAGACCCTATTCTCTACCAACATTTATTT

TGATTTTTTGGTCATCCAGAAGTTTACATTTTAATTCTACCTGGATTTGGTATAATTTCCCATATTATTAGCCAAGAAAGAAGAAAAAAAGAAACATTTG

GAACTTTAGGTATAATTTATGCTATAATAGCTATTGGTTTATTAGGATTTATTGTTTGAGCTCATCATATATTTACAGTAGGTATAGATGTAGACACACG

GGCATATTTTACATCAGCAACAATAATCATTGCTGTTCCTACAGGAATTAAAATTTTTAGATGATTAGCTACTCTCCATGGATCACAATTAAACTACTCT

CCGTCTCTTTTATGAGCATTAGGATTTGTATTCCTATTTACAGTAGGAGGATTAACAGGAGTCATTCTAGCTAATTCATCAATTGACATTATTTTACATG

ATACTTACTATGTAGTTGCACATTTCCATTATGTCCTTTCCATAGGAGCTGTGTTTGCTATTATAGCAGGTTTTGTTCATTGATTCCCTTTATTTACAGG

TTTAACAATAAATTCAAAATTTCTTAAAATTCAATTTTTAACAATATTTATTGGTGTTAATATAACATTCTTCCCCCAACATTTCTTAGGATTAAGAGGA

ATACCCCGACGTTATTCAGATTATCCAGATGCTTATACAACTTGAAATATTATCTCATCTATTGGATCTTTAGTTTCTTTAATTAGTATTTTTATCTTTT

TATTCACTATTTGAGAAAGGCTAATTTCATTGCGAAAAAGAATTAGATCTTTAAGAATATCTACATCAATTGAATGACTTCAACAAATACCCCCTTCAGA

ACATAGTTATTCTGAACTTCCAATGCTTACTAACTTCTAATATGGCAGATTAGTGCAATGGATTTAAACCCCAAATATAAAGATTAAACTTTTTTTAGAA

ATAGCTACTTGAAATACCATTTTACTTCAAGATAGGGCATCCCCATTAATAGAACAACTCTCATTCTTTCATAATCATGCTCTTCTAATTCTCTTAATAA

TTACCGTTTTAGTAGGTTATTTAATAGGAACCTTATTTTTTAACCAATTTAATTACCGATTTTTATTAGATGGTCAAACTATTGAAATTATTTGAACTAT

TTTACCTGCTGTAACACTAATTTTTATCGCATTACCATCTTTACGCTTACTTTATCTTTTAGATGAAGTTAATAACCCTTTAGTAACTATCAAAACAATT

GGGCATCAATGATATTGATCATATGAATACAGAGATTTTATAAATTTTGAATTCGATTCCTATATAATTCCTTTGACAGAAATAAAACCTCAAAATTTTC

GTTTATTAGATGTTGATAACCGAGTAATTGTCCCCTTTAACTCCCAAATTCGAATGATAGTAACAGCTGCCGATGTTATTCATTCATGAACTATCCCAGC

TTTCAGTGTAAAAATTGATGCAACACCAGGCCGACTAAATCAAATTAGATTCCTAATTAATCGAACAGGATTATTCTATGGTCAATGCTCAGAAATTTGC

GGAGCAAATCATAGATTTATACCTATTACTGTAGAAAGAATTTCACCTTCATTTTTTACTAAATGAATCTCAAAAATAAATAACCTATCATTAGATGACT

GAAAGTAAGTAATGGTCTCTTAAACCAATTAATAGTAGTTTAACATCTACTTCTGATGGCCAAAAATTTAGTTAAGATATAACATTAGTTTGTCATACTA

AAATAATCATAATTTGATAATTTTTAATTCCACAAATAGCACCTTTAAACTGACTGTCTTTATTTTTTTTAATTATTATTATTTTTTTACTTTTTAATGT

ATTAAATTACTTTAGATTCTTACAACCCTTAAAAACCCAATCTCATAGCCCTACAATTAAAAAAATTAATTGAAAATGATAACTAATTTATTTTCATCTT

TTGATCCTAGAACTTCTTTTAATTTAAGATTAAATTGATTAAGAATACTATTAGGGCTAATATTTATCCCCCCTATATTTTGATTAGTTCCTTCACGCCA

TAATTTTCTATGAATTAAAATTATTTTGACATTACACCAAGAATTTAAGGTTTTAATTGGTAATAATAATATTAAAGGAAGAACCTTAATATTTATTTCA

TTATTTTCTATAATTGTTTTCAATAACTTTCTAGGGTTATTTCCGTATATTTTTACAGGAACAAGACATTTAATTATAACATTATCTCTTGCCTTACCTT

TATGGATTAGATTCATATTATACGGGTGAATTAATAACACTATCCATATACTTGCACATTTAGTTCCTCAAGGAACACCTCCAGCTCTTATGGCATTCAT

AGTAGTAATTGAATCAATTAGAAATATTATTCGTCCTGGTACTTTAGCGGTTCGATTAGCTGCTAATATAATTGCTGGACATTTACTAATAACTTTACTA

GGAAACACAGGATTAAATTTATCATTTTTTATACTAAGTATTCTTATTATAACACAAATTCTTTTATTAATTTTAGAATCTGCTGTTGCTATTATCCAAT

CTTATGTATTTGCTGTATTAAGAACTTTATACTCTAGAGAAATTAATTAATGTCAAGACATAAAAATCACCCCTATCATTTAGTTGATGCAAGACCTTGA

CCTATTTTAGGTGCTTTTAGAGCTATAATCACAATAATTGGAATTATCAAATGATTCCATTTTTATAATAATTCTTTATTTTACTTAGGGACATTAATCA

CAATTTTAATCATAATTCAATGATGACGGGATATCACTCGTGAAGGAACTTTTCAAGGACTTCATACTTATGCTGTAACTATAGGTTTACGTTGAGGAAT

AATTTTATTTATTACATCAGAAGTATTTTTCTTTATTTCTTTTTTTTGAGCTTTTTTTCATAGTAGTTTAACACCCGCTATTGAACTAGGGATACTATGA

CCACCTAAAGGAATTACTCCATTTAACCCTATTCAAATTCCGTTATTAAATACGTTAATTCTTTTAACTTCAGGATTAACTGTAACTTGAGCTCATCATA

GATTAATAGAAAATGACTATAACCAAACAATACAAGGTCTTGGTTTAACAGTTTTATTAGGAGTATATTTTACTTTGTTACAAGGTTACGAATATTTAGA

AGCCCCCTTTACTATAGCAGATTCTGTTTATGGATCAACATTTTTTATTGCTACTGGTTTCCATGGATTACATGTTATTATTGGTACAACCTTCCTAGCT

GTTTGTTTAATACGACATTTTAATAACCATTTTACTTGTATCCATCACTTTGGGTTTGAAGCTGCTGCTTGATACTGACATTTTGTTGATGTAGTATGAC

TATTTCTTTATATTTCTATTTACTGATGAGGTAGATATTTATATAGTATAATAATTATAATTGATTTCCAATCAAAAGATCTAAAAAAA-TTAGTATAAA

TAATCATTATAATTTGAAATATAAGGTTAATTATTTTTTCTATCTCTTTTATCTTAATTATATTATCTTTTATAATCTCTAAAAAAAGATTTATAGACCG

AGAAAAAGCTTCTCCATTCGAGTGTGGATTTGACCCCAAAAGATCGGCTCGTTTACCTTTTTCTTTACATTTTTTTTTAATTGCAGTAATTTTCTTAATT

TTTGATATTGAAATTACTCTTCTTATCCCTTTAATTCTAACAATAAAAATTACTAATATTACTATATATACTTATATTGCTCTTTTCTTTTTAATGATTC

TTTTAATAGGACTTTATCATGAATGAAACCAAGGAGCCTTAAACTGAGCTCTTTAGGGTAATAGTTAAGTATAACATTTAAGTTGCATTTAAAAAGTATT

GATTTTTCAATTTACCTTAAATAAGAAACAATTAATTGTATTTAGTTTCGACCTAAAATTCAGGTGTATACACACCCTTATTTAAATTAATTGAAACCAA

AAAGAGGTATATCACTGTTAATGATATTAATGAGAAAAGCTCCAATTAAGGAAATAAGATATTCAAGAGTAAGCTTCTAACTTAACTCTTTAGCAGTGAA

AGTCTGTTAATATTTCTATTTATATAGTTTAATAAAACATTATTTTTTCATAATAAAATTAGAATAAATTTATTCTTATAAATATTTAAAAGTAAATTTT

ACTTCCCTGATAACTTCACTATCATACTCTACATAAGCTATTTAAATTAAATATATAAAATTATAAAAATTACCCATATTATAATTAAAAGTATAAAAAT

CTTATAATTGTTATTAAATATAAACTGTAAAAATATAGAAGTATTACTAATTTTACTATATAAATTTTGTCTTCCATAATACTCTGATCAACCTTGATCA

ATAGTTTTATATAACTTACTACCTAATTTAATTGGATAATAATTTAAACCAAATGTTGAAATATAAGGTATATTCCACATAGAAGAAAAAAAAAGTCTTG

AATTTAACAAATAAAAAGATTTTAATCTATCATTTAAAGTAAACTTAGAAAGTTCAAACCCAAATCAAGCCCCAAAAAATGATACAATTAAAGCTATAAT

TTTTATTATAAAAGGTAAACAAATAAAATAAGGTGTTGGGAATATCAACCATATTAATATTCTTCCCCCAACAATAACTAAAAAAATTAAACCCGATATC

CCTTGAAGTATAATTTTTCTATTATCATTAATTTTACTTAAAGAATAAAAAGAAAAATTTCCTACTAAAACATAATAAATTAAACGAAATGTATAACAAA

CCGTTAATCCTGTTGAAAAAAAGAAAATAACATAAATATAGATATTTAAATATCTTATAGATAAAACCTCTAAAATTAAATCCTTTGAATAAAACCCTGA

TAAAAAAGGTAAACCACATAATGCTAAATTTGAAATTATAAAATAAGTACAAGTTAAAGGTATAACTTTAATTAACCCCCCTATATACCGAATATCTTGA

CAATTTCTTAATCTGTGGATTATGCACCCTGCGCATATAAATAATAAAGCCTTAAATAAAGCGTGAGTTAATAGATGAAAAAAAGCTAATTGATACTCCC

CTAAAGCTAAAATACTAATTATCAAACCCAATTGTCTTAAAGTAGACAGAGCAATAATTTTTTTTAAATCAAACTCAAAGTTTGCACCTATTCCTGCTAT

AAATATAGTTATTGTTCCAATAAATAATAAAATTAATATTAAATTACTTGTTAATGCAAAATTAAAACGAATTAATAAGTAAACACCTGCTGTAACTAAA

GTAGAAGAATGAACTAAAGAAGAAACAGGTGTTGGGGCAGCTATTGCCGCAGGCAGCCAAGAAGAAAATGGAATTTGAGCTCTTTTAGTTATAGCTGCTA

ATATAATTAATAATATAATAATATATATCTCTATACTATTTTTATATATATCAATATAAAAAATATAGTTAAATCCCCCGAAATTTACTATTCATGCAAT

TGCTATTAATAAAGCAACATCTCCAATACGGTTAGTTAAAGCTGTAATTATCCCTGCATTATAAGATTTAATATTTTGATAATAAATAACTAAACAATAA

GAAACTAATCCTAATCCATCTCATCCTAATAAAATTCTAATCAAATTAGGAGAAATAATTAATAACATTATAGATAAAACAAATATAGAAACTAATATAA

TAAATCGATGCAAATAAATATCTCCTTCTATATACTCTTCACTATAGTAAATTACTATAGAAGAAATAAATAAAACAAAACTTATAAATAATAATGACAT

TCAGTCAAGTAAAATAGTTATAATAATTCTACAAGAATTAATCCTTAATATTTCATATTCTAATATTAGTCTATAATCTAAAATTATGAAATTTAAGCTT

AATAAAAATCTTAATACTCTAAAAAATAAAAACGTTACAAAATAAATTAAACAAATAGAAATAATTTAAAGTAAACTTTACATCTTTGATACCACAAATC

AATATTTTTTATTAAACTATTTAAATTACAATCATAAAACTAAAAATTCTCTCTTTAAAATTAAGATATTTAAAGGTAATCAATGAAGTAATAATAATAA

ATATTCACGAACAAATCCTCTTGAAAAAGAGTATAAATTTCTAACTAACTTACCATGTTGACTGTAAGAATATAAATATAAAGAATACGCAGCTCTAAAA

AAAGATATTAAAGATAAAAAAACCATTGTTCAACTTCTTCATCTAACCAATCTATTAATCAAAATAATCTCACCCAATAAGTTTAATGAAGGAGGGGCCG

CTATATTACAACAACTAAACAAAAATCATCATATTCTTATTATTGGTATTAAATTAATTAACCCCTTATTTAAATAAATACTTCGACTATTTAGACGTTC

ATAAGAAATATTTGCTAAACAAAATAATCCTGAAGAACATAAACCATGGGCAATTATTATAACTAAAGCCCCTCTTATACCCCAATAACTTAAAGTTAAA

ATCCCTCTTAATACTAAACCTATATGTGCTACAGAAGAATAAGCAATTAAAGCTTTAATATCAACTTGACGTAAACATATTAATGAAACAAAAAATCCCC

CTACTATTCTAATTGTAATAAAAATATAGTTTACTTGTAAACCTACAGTTAAAAAAATATTTATTAAACGTATTAATCCATACCCCCCTAATTTTAATAT

AACTCCAGCTAAAATTATAGAGCCAGCAACTGGAGCTTCAACATGAGCTTTAGGAAGTCAAAGATGAACAAAAAATATAGGCATTTTAATAAAAAACACT

ATATTTATACATATAAATAACAAGAATCTTTTTACATCATAACATATAAAGAAAAAATCTAAAGAATGAAATTTTTCATAATAATAAAAAATCCTAATTA

TTATGGGTAATGAAGCAAATAATGTATAAAATAACAAGTAAACACCTGCCTGCAAACGCTCAGGTTGATATCCTCAACCAATAATTAATAGGAGAGTAGG

AATTAAGCTAAACTCAAAAAACAAATAAAAAATAAATAAATTTAAAGAACTAAATGTTATAACTAAAGACAATAATAAAATAATTATTACTAATAAAAAT

AAATTATAGAAATAGTTTTTTCTATAAATACTTTCAGAAGCTAATAATATCAAAGAACAAATCCACAAACTTAATAAAATTATTATAAAAGATAAAAGAT

CATACCCTATAAAATAAGAAATATTTATATACAAATAATTAAAACTAAATCTTAACCCAAATATAAATGTAATAAAAAAATATATATATTGATTAAATCA

GTATCTCTTTTTAATACAACTTAAAGGTACCAATATTAATATTATAAAAATAAACTTTATCATAATACATTAAAAGTTTGAAAATAATCATTTCCATGCG

TTCGTATTATAGAAACTAATAATGATAAACCTAAAGCTCCTTCACAAACTCTTATAGTTAAAAATACTATACCAAAATAAAATTCAAAATTAAAGTATAT

TAAAAATAGATATAAATTAAAGTATAATCCTAAAATAATATACTCTAACCTTAATAATATTAAAAGTAAATGTTTACGTTTAATACAAAAAGAAACTAAT

CCTGTAAAATATATAATCACTGAAAATAACATACAAAAAATTAACATTAGTTTTAATAATTTAATAAAAATACTGGTCTTGTAAATCAGAAATAAGAATT

TTCTTTTAAAACTTCAGAGAAAGAGTAAACCTCTGTCATTAATCTCCAAAATTAATATTTTAAATAAACTATTCTCTGTATAATCTTATTACTAATAACA

TTATCTTTTATTAGTTCAATAACTTTTATATTTTTAAGTCACCCTTTATCTATAGGGCTTATTTTATTAATACAAACTATTATTATAGCTTTAACTATAG

GTTTTTTTAATATTAATTTCTGATATTCTTATATTTTATTTCTCATTATAATTGGAGGAATATTAGTTTTATTTATTTATATAACAAGAGTAGCTTCAAA

TGAAAAATTTTCATTTTCTATTAAAATTACATTAATAATTGGAATTATAACTTTAGGATTTTTATTTAGTATTGCTATAATAGACCCCTACTTTTCAGAC

ATTAACTCAATTTATACAGAAAACTTAGATAATTATAAAGAATATAATATATCATTTAGAAAATATTTAAGTTACCCTAATATTATCATTATATACATAA

TAATTATTTATTTATTAATTACATTAATTGCAGTTGTAAAAATTACTCAAATTGAAAAAGGACCTTTACGTCAAACTAACTAATGAAAACACCATTACGA

AAAGCTTCCCCCCTATTAAAAATTATTAATAATAGAATTATTGATTTACCTACACCATCTAATATTTCTGCTTGATGAAATTTTGGGTCCTTATTAGGAC

TTTGTCTTTTTATTCAAATTATCACTGGAATTTTCTTAGCTATACATTTTACGGCCCATATTGACATAGCATTTAATAGAGTAATTCATATTTGTCGAGA

TGTAAATTATGGCTGATTATTACGAACAATTCATGCTAACGGAGCTTCCTTTTTCTTTATTTGTATTTATCTACATATTGGCCGAGGAATATATTATAGA

AGCTATAATTTACATTTAACATGAACTATTGGAGTAATCATCCTTTTTATAGTAATAGCAACAGCATTCTTAGGATATGTATTACCTTGAGGGCAAATAT

CTTTTTGAGGAGCTACTGTTATTACCAATCTTCTCTCTGCTATTCCTTACCTAGGAAATATGATTGTCCAATGATTATGGGGAGGATTTGCAGTAGATAA

CGCCACTTTAACTCGATTTTTTACATTACATTTTATTCTTCCTTTTATTATTTTAGCTTTAATAATTATTCATTTATTATTTCTTCATCAAACTGGTTCT

AATAATCCTTTAGGATTAAATAGAAATATCGATAAAGTACCTTTCCATCCGTATTTTACTTACAAAGATACTTTTGGATTCATTATAATAACTATATTAT

TAATATTTTTAGTTTTAATTAATCCCTATCTTTTGGGGGACCCAGAAAACTTTACACCTGCTAACCCTTTAGTAACCCCAGTTCATATCCAACCAGAATG

GTACTTTTTATTCGCATACGCTATTTTACGATCAATTCCTAATAAATTAGGTGGAGTAATTGCTTTAGTTATATCAATTGCTATTTTATTAATTATACCT

TTAATTAACAAAAAAAAATTTAGTAGAACTCAATTTTATCCATTAAATAAAATTTTATTTTGATTTTTTGTTTCTATTGTAATCTTATTGACATGAATCG

GAGCCCGCCCAGTGGAAGATCCATACATTTTAACAGGTCAAATTTTAACAATTATATATTTTAGTTATTATTTCTTAAATCCCTTAATTCATAAAATATG

AGATTATATTATTTTTAAAACTTAGTTAATGAACTTGTTAAAGTGTATATTTTGAAAATATAAAAAAGAGTTTATTCTCTATTAACTTTACTAAATTTTA

TTCACTAAATAAAATAAGAAAAGATAACCAACTTTAATCCTAAAAAGAAAAATAAAAAATTTAAAGAAACTGGTAAATAACTTTTTCAACATATATATAT

TAACTTATCGTAACGATACCGAGGTAAAGTACCTCGAACTCAAATCCAAATAAATGCTATAAAAGTTAACTTCAAAAAAAAAAATAAAGAAAAAATATCC

CCCCCTATAAATAACAACACACATAATATACTTATAAATAAAATTCTAGCATACTCAGCCAAAAAGATTAAAGCGAAACCCCCTCTTCTATACTCTACAT

TAAACCCGGAAACTAACTCTGATTCTCCTTCTGCGAAATCAAAAGGAGTTCGATTAGTCTCAGCTAAACTTGAAGATAATCATATTATTCTTAAAGGTAA

ACATAGAAAAAAAAATCATACTATTTCTTGATATTTTATAAAATCTATTATATTTAAATTTAAAATTAACAATAAAAAAGATAATAAAATTAATGATAAA

CTTACTTCGTATGAAATTGTTTGAGCTACTGATCGAATACCTCCTAATAAAGCATAATTAGAATTTGATGATCAACCAGAAATTATAATAGTATAGACTC

TTAACCTAGAACAACATAAAAAATATAAAATACCTAAATTAAACCTAAATATAAATGTCAAAAAAGGCATACATATCCATAATAAAAGAGCTAAAAATAA

ATTAAAAACTGGAGATATATAATAAATAAAAAAATTTGATATTAAAGGATAAGTTTGTTCTTTAGAAAATAACTTAATAGCATCACTAAAAGGTTGGGGA

ATACCTATAAATCCAACTTTATTGGGCCCTTTACGAATTTGAATATACCCTAAAACTTTACGCTCTATTAATGTTAAAAAGGCTACACCAATTAAAACAC

AAATAATTAAAATTAAACTTGTAAATAATAAAAGAAATAAATCTTGTAATATAATGTATTACTTGTGTTAAACACATATTTAAATTCTAAATTTAAAGCA

CTAATCTGCCAAAGTAATATTCATATTCAAATTATATTAAATTTTAAAGGTATCTGATCCTTTCGTACTAAAATACCTATATTTTTTAAAGATAGAAACC

AACCTGGCTCACGCCGGTTTAAACTCAGATCATGTAAAATTTTAAAGGTCGAACAGACCTAACCTTTTAGCCCCTACACCAAAAGTTAATTTTAATCCAA

CATCGAGGTCGCAAACTTTTTTTTCGATAAGAACTCTAAAAAAAAATTACGCTGTTATCCCTAAGGTAATTTAATCTTATAATCATTAAAAATGGATCAT

TCAATCATAAATTAATGTTTTTAAATAAAAAAAGTTTAATCAATTTTTCTGCTGCCCCAGCAAAATAGTTTAAATTATTAAATATATAAATATACTAAAA

TTAAATAATAATTTAAACTATAAAACTCTATAGGGTCTTCTCGTCTTTTAAAATTATATAAGCTTTTTTACTTATAAATAAAATTCTATATTCAATTAAA

TTGAGACAGTTACTTTCTCGTCCAACCGTTCATTCCAGCTTTCAATTAAAAAACTAATGATTATGCTACCTTTGCACGGTCAAATTACCGCGGCCATTCA

AATCCTCATTGGGCAGGTCAGACTTTAAATTATAATCAAAAAGACATGTTTTTAATAAACAGGCGAAAAGTATATTTGCCGAGTTCCTTAATTTAACCTT

GAAGTTTTAATTTAATTACTAAATTAAAATATATACTAATTTTATCATTATTCTATGTAAACCAATATTACATACATTATCTTAATAAACTACTTAAAAA

TAATATAAATCTTATTCTAACAAAAATTATTTATAACAAACTAAAGATTAACACTTCCAATTCTACTAATTTTTATTCAAAATATACATTTTTAACATTT

TATTTTAAAGCTTATCCCCTAAAATATTACTTTTTATATATAAAATACTAAATAATTAATATAATATAATAAAAAAACTAAATTAAATTTATTTCTTAAG

AAACTAGATATCTTAAAAAACGTATAACGTTTCATTTCTAATATAATATTTTAAAAATTTATGCCACAATTAAATTTATATTATATTAGCTCTTTATAAT

TCGAGAACACCAAATAATTAAATTATTTTAATAAACCCTGATACACAAGGTACAAAAAATTAATTTTTCTTTTTAAAAAATAAATCTCTATATATTTATA

TTATCTATCTCTATACAAATTAACTATAATAAAATTTTTATATTCTAAAATATACTAATATCAAAAATATTTTTTTTATATATATATATATA----TTAT

AAATTTTTCCTTTCAAATTAAATTGATTTTCACAACTAACTTTTTAATGTAAATAAAATGCTTTATTACAAGCTCTAATTTGCCATTCCAGGTACACTTT

CCAGTACACCTACTATGTTACGACTTATCCCCCTTTAGAGAGGGAGCGACGGGCGATATGTACATATTCTAGAGCTATACTCATATAATTAAACTAAACT

ATATTACTTTCAAATCCACTTTATAAAATAATGTTAATTATTTTAACCATCTAAATAATTTTATTGTAACCCATCTCTCCTTATCTATACGCTGTATCTT

GATCTGATTTTTTTTATACTTATAAATTTTGAACATTCCAAATTCTTTAAAAACATTCAACCTACGACGATATACAAACCTTTAAAATAAGTACGATTAA

TCGTGGATCATCAATTACAGGACAGGTTCCTCTGAGTAGACTAAAATACCGCCAAATTCTTAAAATTTCAAGAACATAACTACTACTATTCAAGCATCTA

AAATTTGCATTTTTAATAATAGGGTATCTAATCCTAGTTTTTTATAAAAATCTCATAAACTCATTTTTCACATTTAAAAAATTAATTATACTTACTAATT

TCACCTAATAAATACAATATAAATTAATAATAAAATAACTTATTATATACTGAACAAATTTAATTGCATTGTTTGTGTAACCGCAACTGCTGGCACAAAC

TTGGTCAATACTATTATAAATTCCTAAATCAAAATTTCTTTTAAATTTAATCTTCACTATTGCAATTCTTTAATTAAATATATAGAATAATCTATCTTTT

TAAAATAAATTCATTAAACACTAAAATTTACATATAAAATAATTTAAAAATTAAAATCTCAAGCTAGAATAAAACTTTATTTTTTTTTTAATATACATAA

ATTATATAAAATAAAAGTACCCCCC-TACCATTTTAGTTAAGTGAATTAATTTTTAATACCTTAAATTTATTCTCAATTTATTTTTATTATATAATTTTT

TAGTTTAAAATAAAACTTTATACCTAAAATTATATACATAAATTATATTTAGAATTTATAATTTAAAGAACTATAAAAATTAAATTAAAATTTTAAATCT

ATCTGATTTACAGATAAATAAATTAAATTAAACTAACTTACCTGATAAATTTAACTATATACGCACGGGTATATTAATTATTTAATTATAATATAATACA

TTTGTTTTATTAAATAATTATTAAGCAAAATAGCATTAATTTATTATATAAAAGATCTAGATATTATTAATTTAAAGTAAATTTTTTTTTTTTTTGTAGT

ATAATTTATACTAATATACGGCATTTAATATTTTCTATATAAATGTTTATATTATTTATATTTATCTATTTATAATTCTTAATAATAATTTAGATAATTA

TTAATTTATATATTAAATATTTATATATATATATATAATAATGAGGATATATATATATATATGTATATAGATAAATTAAATTTAGTTATATATAAAAAAA

TTAATATTAATTTTACTTTTAATTTTTTCTTTAATTTAAACTATTGGATATTCAGATTCTAATGTATTATAATATATCAACCATTAATGAATAACTTTAT

ATATATAATAAAAATGTTAATTAATATATCAATTCATATGAATATATAATATTAATAAATAAATTCTTAATAAATAATAATGATAATATATATATATTGT

ATATTAATGTACTATATATATAAATTCATTAAACATTTATATTTGATACCCCCCCAAAATTTTTTCTAAAAATGTGAAGTTTCAATTTAATTAAAATTTT

CTACAATGGTCAAAAACGGCCAAAAAATGGAAAATAAAATGTGCACAGAAAATGCACATAAAATGCACATTTTTGAAAATTTCCGATTTTTGAAAAACCG

TTTGAACCAAACCTCTGATGTACCTTTTTTCGCCCCCCCTGATTACTAAAATTTATCCTCAATTACTAAGGTTTATCCGCATTTTTGCGACAGGAATTTT

GAGTATAAAATCAATTCCACACTAAATTTTTTTTT-TTTTTTTTGTGTCATTTCTCCAACATGGGCAATTTTTTTTTTAGTCGAAGGACAAATTTTAGTA

AAAAAAGTTTGAAATCAGGTTAAACCCCTATTTAACCAAAAACTATTACTTTTTAATATTGTACCCAATTTTATTTAAAATAAATTTTATTATACTTTTA

TTTATGCAAAAAGATCTTCTTATAAACTTTT

>DMR125j_JP_Fukuoka_Kyushu

AATGAAGTGTCTGACTATAGAGTTACTTTGATAGAGTAAAAAAAGTGAATTTTCACCTTCATTATAATTAACAGAATTAAACTATTTCTTTAAGCTTCAA

AAACTTATGTACATTATATACTAAATTATAAAAAGATAAGCTAATTAAGCTACTGGGCTCATACCCCATCAATAAAGGTTACAATCCTTTTCTTTTTAAT

GTATTATAAGCTTTTATTTTTTAATTCTCTTATAATTGGAACCTTAATTGCTATTTCTTCCTACTCTTGGATAGGAATATGAATAGGATTAGAAATTAAT

CTTCTCTCTATGATCCCCCTCATTAGAGACAATAAAAATATAATAGCCTCAGAAGCTGCTTTAAAATATTTCATTATTCAAACAATAGCATCAACATTGT

TATTATTCTCAATTATTATAATATCAATGAAATTTATATATCAAATAAATTTAATCACTTACTTTAATTTAATTTTTAACACTTCATTATTCATCAAAAT

AGGAGCAGCCCCATTCCATTTTTGATTCCCCGAAATAATAGAAGGATTAAATTGATTAAATGCCATTATCATACTTACTTGACAAAAACTAAGACCTATA

GTATTATTAACATATTCTAATACAACCTCCATATATCTAATTTTAACAATTATATTTAGAATAATAATCAGAGGAATTATAGGTCTAAATCAAACTAGAT

TACGAAAAATTATAGCTTATTCATCTATCAACCATATTGGATGAATAATCAGTTCAATTATATTAATTGAAATTGTTTGATTTTACTATTTTATTATTTA

CTGCATTATTACTATTAATATCAGAATTATATTTATAAAATTAAATGTTTTTCATATCAATCAATTATATATTTCAATAAATTACCATATTTTACTTAAA

TTATTCTTTGCCTTAAATTTCATATCTTTAGGAGGATTACCTCCATTTTTAGGATTTTTCCCTAAATGACTTACAATTCAAACTTTAATCCAAAGAAATA

TACACTCAATTGCTTTCATTATAATTTTAATAACTTTAATAACACTTTTTTTTTACCTCCGAATTACTTTTTCAATTTTATTACTAAGAAAAACAGTTTT

AACATTTTACACACAACCAAAAATTTATACTAATTACATTATAGCATTTAATTTTATTACATTATTAAGATTAATTTTCGTTACTTTGATATTCAATTTC

TTATAAATTAAACATGAAGGATTTAAGTTAAATTAAACTAAGAACCTTCAAAGTTCTAAATAAAGTAAATTCTTTAAGCCTTAGGGCTTAGCCCATCTTT

AAATTTGCAATTTAAAATTCTTTTTGAACTATAAAGCTTGATAAAAGAAACTAATTTCGTATGTAAATTTACAGTTTACCGCCTAAACCTCGGCCATTTT

ATCGAATAAATGGCTATTCTCTACAAATCACAAAGACATCGGAACTTTATACTTTCTATTTGGAAGTTGGGCAGGTATAGTAGGCACTTCATTAAGTTTA

CTGATTCGTGCTGAATTAGGAAACCCGGGATCTCTAATTGGAGATGACCAAATTTATAACGTAATCGTTACAGCTCATGCTTTCATTATAATTTTTTTTA

TAGTAATACCTATTATAATTGGAGGATTTGGAAATTGATTAGTTCCTTTAATGCTTGGAGCCCCTGACATAGCATTCCCTCGAATAAATAACATAAGATT

TTGACTTTTACCTCCTTCATTAACTCTACTTCTAATAAGAAGATTAGTCGAAAGAGGAGCAGGTACAGGCTGAACAGTTTATCCCCCACTATCAGCCAAT

ATTGCCCATAGAGGAGCATCAGTTGATTTAGCAATTTTTAGACTTCATCTTGCAGGAATTAGTTCAATTTTAGGCGCAGTAAATTTCATTACCACTGTAA

TTAACATGCGATCAACAGGAATAACTTTTGATCGAATACCCTTATTTGTTTGATCTGTAGTTTTAACAGCACTTCTTCTGCTATTATCTCTCCCCGTTTT

AGCAGGAGCAATTACTATACTATTAACAGATCGAAATATTAATACAACATTTTTTGATCCCGCAGGTGGAGGAGACCCTATTCTCTACCAACATTTATTT

TGATTTTTTGGTCATCCAGAAGTTTACATTTTAATTCTACCTGGATTTGGTATAATTTCCCATATTATTAGCCAAGAAAGAAGAAAAAAAGAAACATTTG

GAACTTTAGGTATAATTTATGCTATAATAGCTATTGGTTTATTAGGATTTATTGTTTGAGCTCATCATATATTTACAGTAGGTATAGATGTAGACACACG

GGCATATTTTACATCAGCAACAATAATCATTGCTGTTCCTACAGGAATTAAAATTTTTAGATGATTAGCTACTCTCCATGGATCACAATTAAACTACTCT

CCGTCTCTTTTATGAGCATTAGGATTTGTATTCCTATTTACAGTAGGAGGATTAACAGGAGTCATTCTAGCTAATTCATCAATTGACATTATTTTACATG

ATACTTACTATGTAGTTGCACATTTCCATTATGTCCTTTCCATAGGAGCTGTGTTTGCTATTATAGCAGGTTTTGTTCATTGATTCCCTTTATTTACAGG

TTTAACAATAAATTCAAAATTTCTTAAAATTCAATTTTTAACAATATTTATTGGTGTTAATATAACATTCTTCCCCCAACATTTCTTAGGATTAAGAGGA

ATACCCCGACGTTATTCAGATTATCCAGATGCTTATACAACTTGAAATATTATCTCATCTATTGGATCTTTAGTTTCTTTAATTAGTATTTTTATCTTTT

TATTCACTATTTGAGAAAGGCTAATTTCATTGCGAAAAAGAATTAGATCTTTAAGAATATCTACATCAATTGAATGACTTCAACAAATACCCCCTTCAGA

ACATAGTTATTCTGAACTTCCAATGCTTACTAACTTCTAATATGGCAGATTAGTGCAATGGATTTAAACCCCAAATATAAAGATTAAACTTTTTTTAGAA

ATAGCTACTTGAAATACCATTTTACTTCAAGATAGGGCATCCCCATTAATAGAACAACTCTCATTCTTTCATAATCATGCTCTTCTAATTCTCTTAATAA

TTACCGTTTTAGTAGGTTATTTAATAGGAACCTTATTTTTTAACCAATTTAATTACCGATTTTTATTAGATGGTCAAACTATTGAAATTATTTGAACTAT

TTTACCTGCTGTAACACTAATTTTTATCGCATTACCATCTTTACGCTTACTTTATCTTTTAGATGAAGTTAATAACCCTTTAGTAACTATCAAAACAATT

GGGCATCAATGATATTGATCATATGAATACAGAGATTTTATAAATTTTGAATTCGATTCCTATATAATTCCTTTGACAGAAATAAAACCTCAAAATTTTC

GTTTATTAGATGTTGATAACCGAGTAATTGTCCCCTTTAACTCCCAAATTCGAATGATAGTAACAGCTGCCGATGTTATTCATTCATGAACTATCCCAGC

TTTCAGTGTAAAAATTGATGCAACACCAGGCCGACTAAATCAAATTAGATTCCTAATTAATCGAACAGGATTATTCTATGGTCAATGCTCAGAAATTTGC

GGAGCAAATCATAGATTTATACCTATTACTGTAGAAAGAATTTCACCTTCATTTTTTACTAAATGAATCTCAAAAATAAATAACCTATCATTAGATGACT

GAAAGTAAGTAATGGTCTCTTAAACCAATTAATAGTAGTTTAACATCTACTTCTGATGGCCAAAAATTTAGTTAAGATATAACATTAGTTTGTCATACTA

AAATAATCATAATTTGATAATTTTTAATTCCACAAATAGCACCTTTAAACTGACTGTCTTTATTTTTTTTAATTATTATTATTTTTTTACTTTTTAATGT

ATTAAATTACTTTAGATTCTTACAACCCTTAAAAACCCAATCTCATAGCCCTACAATTAAAAAAATTAATTGAAAATGATAACTAATTTATTTTCATCTT

TTGATCCTAGAACTTCTTTTAATTTAAGATTAAATTGATTAAGAATACTATTAGGGCTAATATTTATCCCCCCTATATTTTGATTAGTTCCTTCACGCCA

TAATTTTCTATGAATTAAAATTATTTTGACATTACACCAAGAATTTAAGGTTTTAATTGGTAATAATAATATTAAAGGAAGAACCTTAATATTTATTTCA

TTATTTTCTATAATTGTTTTCAATAACTTTCTAGGGTTATTTCCGTATATTTTTACAGGAACAAGACATTTAATTATAACATTATCTCTTGCCTTACCTT

TATGGATTAGATTCATATTATACGGGTGAATTAATAACACTATCCATATACTTGCACATTTAGTTCCTCAAGGAACACCTCCAGCTCTTATGGCATTCAT

AGTAGTAATTGAATCAATTAGAAATATTATTCGTCCTGGTACTTTAGCGGTTCGATTAGCTGCTAATATAATTGCTGGACATTTACTAATAACTTTACTA

GGAAACACAGGATTAAATTTATCATTTTTTATACTAAGTATTCTTATTATAACACAAATTCTTTTATTAATTTTAGAATCTGCTGTTGCTATTATCCAAT

CTTATGTATTTGCTGTATTAAGAACTTTATACTCTAGAGAAATTAATTAATGTCAAGACATAAAAATCACCCCTATCATTTAGTTGATGCAAGACCTTGA

CCTATTTTAGGTGCTTTTAGAGCTATAATCACAATAATTGGAATTATCAAATGATTCCATTTTTATAATAATTCTTTATTTTACTTAGGGACATTAATCA

CAATTTTAATCATAATTCAATGATGACGGGATATCACTCGTGAAGGAACTTTTCAAGGACTTCATACTTATGCTGTAACTATAGGTTTACGTTGAGGAAT

AATTTTATTTATTACATCAGAAGTATTTTTCTTTATTTCTTTTTTTTGAGCTTTTTTTCATAGTAGTTTAACACCCGCTATTGAACTAGGGATACTATGA

CCACCTAAAGGAATTACTCCATTTAACCCTATTCAAATTCCGTTATTAAATACGTTAATTCTTTTAACTTCAGGATTAACTGTAACTTGAGCTCATCATA

GATTAATAGAAAATGACTATAACCAAACAATACAAGGTCTTGGTTTAACAGTTTTATTAGGAGTATATTTTACTTTGTTACAAGGTTACGAATATTTAGA

AGCCCCCTTTACTATAGCAGATTCTGTTTATGGATCAACATTTTTTATTGCTACTGGTTTCCATGGATTACATGTTATTATTGGTACAACCTTCCTAGCT

GTTTGTTTAATACGACATTTTAATAACCATTTTACTTGTATCCATCACTTTGGGTTTGAAGCTGCTGCTTGATACTGACATTTTGTTGATGTAGTATGAC

TATTTCTTTATATTTCTATTTACTGATGAGGTAGATATTTATATAGTATAATAATTATAATTGATTTCCAATCAAAAGATCTAAAAAAA-TTAGTATAAA

TAATCATTATAATTTGAAATATAAGGTTAATTATTTTTTCTATCTCTTTTATCTTAATTATATTATCTTTTATAATCTCTAAAAAAAGATTTATAGACCG

AGAAAAAGCTTCTCCATTCGAGTGTGGATTTGACCCCAAAAGATCGGCTCGTTTACCTTTTTCTTTACATTTTTTTTTAATTGCAGTAATTTTCTTAATT

TTTGATATTGAAATTACTCTTCTTATCCCTTTAATTCTAACAATAAAAATTACTAATATTACTATATATACTTATATTGCTCTTTTCTTTTTAATGATTC

TTTTAATAGGACTTTATCATGAATGAAACCAAGGAGCCTTAAACTGAGCTCTTTAGGGTAATAGTTAAGTATAACATTTAAGTTGCATTTAAAAAGTATT

GATTTTTCAATTTACCTTAAATAAGAAACAATTAATTGTATTTAGTTTCGACCTAAAATTCAGGTGTATACACACCCTTATTTAAATTAATTGAAACCAA

AAAGAGGTATATCACTGTTAATGATATTAATGAGAAAAGCTCCAATTAAGGAAATAAGATATTCAAGAGTAAGCTTCTAACTTAACTCTTTAGCAGTGAA

AGTCTGTTAATATTTCTATTTATATAGTTTAATAAAACATTATTTTTTCATAATAAAATTAGAATAAATTTATTCTTATAAATATTTAAAAGTAAATTTT

ACTTCCCTGATAACTTCACTATCATACTCTACATAAGCTATTTAAATTAAATATATAAAATTATAAAAATTACCCATATTATAATTAAAAGTATAAAAAT

CTTATAATTGTTATTAAATATAAACTGTAAAAATATAGAAGTATTACTAATTTTACTATATAAATTTTGTCTTCCATAATACTCTGATCAACCTTGATCA

ATAGTTTTATATAACTTACTACCTAATTTAATTGGATAATAATTTAAACCAAATGTTGAAATATAAGGTATATTCCACATAGAAGAAAAAAAAAGTCTTG

AATTTAACAAATAAAAAGATTTTAATCTATCATTTAAAGTAAACTTAGAAAGTTCAAACCCAAATCAAGCCCCAAAAAATGATACAATTAAAGCTATAAT

TTTTATTATAAAAGGTAAACAAATAAAATAAGGTGTTGGGAATATCAACCATATTAATATTCTTCCCCCAACAATAACTAAAAAAATTAAACCCGATATC

CCTTGAAGTATAATTTTTCTATTATCATTAATTTTACTTAAAGAATAAAAAGAAAAATTTCCTACTAAAACATAATAAATTAAACGAAATGTATAACAAA

CCGTTAATCCTGTTGAAAAAAAGAAAATAACATAAATATAGATATTTAAATATCTTATAGATAAAACCTCTAAAATTAAATCCTTTGAATAAAACCCTGA

TAAAAAAGGTAAACCACATAATGCTAAATTTGAAATTATAAAATAAGTACAAGTTAAAGGTATAACTTTAATTAACCCCCCTATATACCGAATATCTTGA

CAATTTCTTAATCTGTGGATTATGCACCCTGCGCATATAAATAATAAAGCCTTAAATAAAGCGTGAGTTAATAGATGAAAAAAAGCTAATTGATACTCCC

CTAAAGCTAAAATACTAATTATCAAACCCAATTGTCTTAAAGTAGACAGAGCAATAATTTTTTTTAAATCAAACTCAAAGTTTGCACCTATTCCTGCTAT

AAATATAGTTATTGTTCCAATAAATAATAAAATTAATATTAAATTACTTGTTAATGCAAAATTAAAACGAATTAATAAGTAAACACCTGCTGTAACTAAA

GTAGAAGAATGAACTAAAGAAGAAACAGGTGTTGGGGCAGCTATTGCCGCAGGCAGCCAAGAAGAAAATGGAATTTGAGCTCTTTTAGTTATAGCTGCTA

ATATAATTAATAATATAATAATATATATCTCTATACTATTTTTATATATATCAATATAAAAAATATAGTTAAATCCCCCGAAATTTACTATTCATGCAAT

TGCTATTAATAAAGCAACATCTCCAATACGGTTAGTTAAAGCTGTAATTATCCCTGCATTATAAGATTTAATATTTTGATAATAAATAACTAAACAATAA

GAAACTAATCCTAATCCATCTCATCCTAATAAAATTCTAATCAAATTAGGAGAAATAATTAATAACATTATAGATAAAACAAATATAGAAACTAATATAA

TAAATCGATGCAAATAAATATCTCCTTCTATATACTCTTCACTATAGTAAATTACTATAGAAGAAATAAATAAAACAAAACTTATAAATAATAATGACAT

TCAGTCAAGTAAAATAGTTATAATAATTCTACAAGAATTAATCCTTAATATTTCATATTCTAATATTAGTCTATAATCTAAAATTATGAAATTTAAGCTT

AATAAAAATCTTAATACTCTAAAAAATAAAAACGTTACAAAATAAATTAAACAAATAGAAATAATTTAAAGTAAACTTTACATCTTTGATACCACAAATC

AATATTTTTTATTAAACTATTTAAATTACAATCATAAAACTAAAAATTCTCTCTTTAAAATTAAGATATTTAAAGGTAATCAATGAAGTAATAATAATAA

ATATTCACGAACAAATCCTCTTGAAAAAGAGTATAAATTTCTAACTAACTTACCATGTTGACTGTAAGAATATAAATATAAAGAATACGCAGCTCTAAAA

AAAGATATTAAAGATAAAAAAACCATTGTTCAACTTCTTCATCTAACCAATCTATTAATCAAAATAATCTCACCCAATAAGTTTAATGAAGGAGGGGCCG

CTATATTACAACAACTAAACAAAAATCATCATATTCTTATTATTGGTATTAAATTAATTAACCCCTTATTTAAATAAATACTTCGACTATTTAGACGTTC

ATAAGAAATATTTGCTAAACAAAATAATCCTGAAGAACATAAACCATGGGCAATTATTATAACTAAAGCCCCTCTTATACCCCAATAACTTAAAGTTAAA

ATCCCTCTTAATACTAAACCTATATGTGCTACAGAAGAATAAGCAATTAAAGCTTTAATATCAACTTGACGTAAACATATTAATGAAACAAAAAATCCCC

CTACTATTCTAATTGTAATAAAAATATAGTTTACTTGTAAACCTACAGTTAAAAAAATATTTATTAAACGTATTAATCCATACCCCCCTAATTTTAATAT

AACTCCAGCTAAAATTATAGAGCCAGCAACTGGAGCTTCAACATGAGCTTTAGGAAGTCAAAGATGAACAAAAAATATAGGCATTTTAATAAAAAACACT

ATATTTATACATATAAATAACAAGAATCTTTTTACATCATAACATATAAAGAAAAAATCTAAAGAATGAAATTTTTCATAATAATAAAAAATCCTAATTA

TTATGGGTAATGAAGCAAATAATGTATAAAATAACAAGTAAACACCTGCCTGCAAACGCTCAGGTTGATATCCTCAACCAATAATTAATAGGAGAGTAGG

AATTAAGCTAAACTCAAAAAACAAATAAAAAATAAATAAATTTAAAGAACTAAATGTTATAACTAAAGACAATAATAAAATAATTATTACTAATAAAAAT

AAATTATAGAAATAGTTTTTTCTATAAATACTTTCAGAAGCTAATAATATCAAAGAACAAATCCACAAACTTAATAAAATTATTATAAAAGATAAAAGAT

CATACCCTATAAAATAAGAAATATTTATATACAAATAATTAAAACTAAATCTTAACCCAAATATAAATGTAATAAAAAAATATATATATTGATTAAATCA

GTATCTCTTTTTAATACAACTTAAAGGTACCAATATTAATATTATAAAAATAAACTTTATCATAATACATTAAAAGTTTGAAAATAATCATTTCCATGCG

TTCGTATTATAGAAACTAATAATGATAAACCTAAAGCTCCTTCACAAACTCTTATAGTTAAAAATACTATACCAAAATAAAATTCAAAATTAAAGTATAT

TAAAAATAGATATAAATTAAAGTATAATCCTAAAATAATATACTCTAACCTTAATAATATTAAAAGTAAATGTTTACGTTTAATACAAAAAGAAACTAAT

CCTGTAAAATATATAATCACTGAAAATAACATACAAAAAATTAACATTAGTTTTAATAATTTAATAAAAATACTGGTCTTGTAAATCAGAAATAAGAATT

TTCTTTTAAAACTTCAGAGAAAGAGTAAACCTCTGTCATTAATCTCCAAAATTAATATTTTAAATAAACTATTCTCTGTATAATCTTATTACTAATAACA

TTATCTTTTATTAGTTCAATAACTTTTATATTTTTAAGTCACCCTTTATCTATAGGGCTTATTTTATTAATACAAACTATTATTATAGCTTTAACTATAG

GTTTTTTTAATATTAATTTCTGATATTCTTATATTTTATTTCTCATTATAATTGGAGGAATATTAGTTTTATTTATTTATATAACAAGAGTAGCTTCAAA

TGAAAAATTTTCATTTTCTATTAAAATTACATTAATAATTGGAATTATAACTTTAGGATTTTTATTTAGTATTGCTATAATAGACCCCTACTTTTCAGAC

ATTAACTCAATTTATACAGAAAACTTAGATAATTATAAAGAATATAATATATCATTTAGAAAATATTTAAGTTACCCTAATATTATCATTATATACATAA

TAATTATTTATTTATTAATTACATTAATTGCAGTTGTAAAAATTACTCAAATTGAAAAAGGACCTTTACGTCAAACTAACTAATGAAAACACCATTACGA

AAAGCTTCCCCCCTATTAAAAATTATTAATAATAGAATTATTGATTTACCTACACCATCTAATATTTCTGCTTGATGAAATTTTGGGTCCTTATTAGGAC

TTTGTCTTTTTATTCAAATTATCACTGGAATTTTCTTAGCTATACATTTTACGGCCCATATTGACATAGCATTTAATAGAGTAATTCATATTTGTCGAGA

TGTAAATTATGGCTGATTATTACGAACAATTCATGCTAACGGAGCTTCCTTTTTCTTTATTTGTATTTATCTACATATTGGCCGAGGAATATATTATAGA

AGCTATAATTTACATTTAACATGAACTATTGGAGTAATCATCCTTTTTATAGTAATAGCAACAGCATTCTTAGGATATGTATTACCTTGAGGGCAAATAT

CTTTTTGAGGAGCTACTGTTATTACCAATCTTCTCTCTGCTATTCCTTACCTAGGAAATATGATTGTCCAATGATTATGGGGAGGATTTGCAGTAGATAA

CGCCACTTTAACTCGATTTTTTACATTACATTTTATTCTTCCTTTTATTATTTTAGCTTTAATAATTATTCATTTATTATTTCTTCATCAAACTGGTTCT

AATAATCCTTTAGGATTAAATAGAAATATCGATAAAGTACCTTTCCATCCGTATTTTACTTACAAAGATACTTTTGGATTCATTATAATAACTATATTAT

TAATATTTTTAGTTTTAATTAATCCCTATCTTTTGGGGGACCCAGAAAACTTTACACCTGCTAACCCTTTAGTAACCCCAGTTCATATCCAACCAGAATG

GTACTTTTTATTCGCATACGCTATTTTACGATCAATTCCTAATAAATTAGGTGGAGTAATTGCTTTAGTTATATCAATTGCTATTTTATTAATTATACCT

TTAATTAACAAAAAAAAATTTAGTAGAACTCAATTTTATCCATTAAATAAAATTTTATTTTGATTTTTTGTTTCTATTGTAATCTTATTGACATGAATCG

GAGCCCGCCCAGTGGAAGATCCATACATTTTAACAGGTCAAATTTTAACAATTATATATTTTAGTTATTATTTCTTAAATCCCTTAATTCATAAAATATG

AGATTATATTATTTTTAAAACTTAGTTAATGAACTTGTTAAAGTGTATATTTTGAAAATATAAAAAAGAGTTTATTCTCTATTAACTTTACTAAATTTTA

TTCACTAAATAAAATAAGAAAAGATAACCAACTTTAATCCTAAAAAGAAAAATAAAAAATTTAAAGAAACTGGTAAATAACTTTTTCAACATATATATAT

TAACTTATCGTAACGATACCGAGGTAAAGTACCTCGAACTCAAATCCAAATAAATGCTATAAAAGTTAACTTCAAAAAAAAAAATAAAGAAAAAATATCC

CCCCCTATAAATAACAACACACATAATATACTTATAAATAAAATTCTAGCATACTCAGCCAAAAAGATTAAAGCGAAACCCCCTCTTCTATACTCTACAT

TAAACCCGGAAACTAACTCTGATTCTCCTTCTGCGAAATCAAAAGGAGTTCGATTAGTCTCAGCTAAACTTGAAGATAATCATATTATTCTTAAAGGTAA

ACATAGAAAAAAAAATCATACTATTTCTTGATATTTTATAAAATCTATTATATTTAAATTTAAAATTAACAATAAAAAAGATAATAAAATTAATGATAAA

CTTACTTCGTATGAAATTGTTTGAGCTACTGATCGAATACCTCCTAATAAAGCATAATTAGAATTTGATGATCAACCAGAAATTATAATAGTATAGACTC

TTAACCTAGAACAACATAAAAAATATAAAATACCTAAATTAAACCTAAATATAAATGTCAAAAAAGGCATACATATCCATAATAAAAGAGCTAAAAATAA

ATTAAAAACTGGAGATATATAATAAATAAAAAAATTTGATATTAAAGGATAAGTTTGTTCTTTAGAAAATAACTTAATAGCATCACTAAAAGGTTGGGGA

ATACCTATAAATCCAACTTTATTGGGCCCTTTACGAATTTGAATATACCCTAAAACTTTACGCTCTATTAATGTTAAAAAGGCTACACCAATTAAAACAC

AAATAATTAAAATTAAACTTGTAAATAATAAAAGAAATAAATCTTGTAATATAATGTATTACTTGTGTTAAACACATATTTAAATTCTAAATTTAAAGCA

CTAATCTGCCAAAGTAATATTCATATTCAAATTATATTAAATTTTAAAGGTATCTGATCCTTTCGTACTAAAATACCTATATTTTTTAAAGATAGAAACC

AACCTGGCTCACGCCGGTTTAAACTCAGATCATGTAAAATTTTAAAGGTCGAACAGACCTAACCTTTTAGCCCCTACACCAAAAGTTAATTTTAATCCAA

CATCGAGGTCGCAAACTTTTTTTTCGATAAGAACTCTAAAAAAAAATTACGCTGTTATCCCTAAGGTAATTTAATCTTATAATCATTAAAAATGGATCAT

TCAATCATAAATTAATGTTTTTAAATAAAAAAAGTTTAATCAATTTTTCTGCTGCCCCAGCAAAATAGTTTAAATTATTAAATATATAAATATACTAAAA

TTAAATAATAATTTAAACTATAAAACTCTATAGGGTCTTCTCGTCTTTTAAAATTATATAAGCTTTTTTACTTATAAATAAAATTCTATATTCAATTAAA

TTGAGACAGTTACTTTCTCGTCCAACCGTTCATTCCAGCTTTCAATTAAAAAACTAATGATTATGCTACCTTTGCACGGTCAAATTACCGCGGCCATTCA

AATCCTCATTGGGCAGGTCAGACTTTAAATTATAATCAAAAAGACATGTTTTTAATAAACAGGCGAAAAGTATATTTGCCGAGTTCCTTAATTTAACCTT

GAAGTTTTAATTTAATTACTAAATTAAAATATATACTAATTTTATCATTATTCTATGTAAACCAATATTACATACATTATCTTAATAAACTACTTAAAAA

TAATATAAATCTTATTCTAACAAAAATTATTTATAACAAACTAAAGATTAACACTTCCAATTCTACTAATTTTTATTCAAAATATACATTTTTAACATTT

TATTTTAAAGCTTATCCCCTAAAATATTACTTTTTATATATAAAATACTAAATAATTAATATAATATAATAAAAAAACTAAATTAAATTTATTTCTTAAG

AAACTAGATATCTTAAAAAACGTATAACGTTTCATTTCTAATATAATATTTTAAAAATTTATGCCACAATTAAATTTATATTATATTAGCTCTTTATAAT

TCGAGAACACCAAATAATTAAATTATTTTAATAAACCCTGATACACAAGGTACAAAAAATTAATTTTTCTTTTTAAAAAATAAATCTCTATATATTTATA

TTATCTATCTCTATACAAATTAACTATAATAAAATTTTTATATTCTAAAATATACTAATATCAAAAATATTTTTTTTATATATATATATATA----TTAT

AAATTTTTCCTTTCAAATTAAATTGATTTTCACAACTAACTTTTTAATGTAAATAAAATGCTTTATTACAAGCTCTAATTTGCCATTCCAGGTACACTTT

CCAGTACACCTACTATGTTACGACTTATCCCCCTTTAGAGAGGGAGCGACGGGCGATATGTACATATTCTAGAGCTATACTCATATAATTAAACTAAACT

ATATTACTTTCAAATCCACTTTATAAAATAATGTTAATTATTTTAACCATCTAAATAATTTTATTGTAACCCATCTCTCCTTATCTATACGCTGTATCTT

GATCTGATTTTTTTTATACTTATAAATTTTGAACATTCCAAATTCTTTAAAAACATTCAACCTACGACGATATACAAACCTTTAAAATAAGTACGATTAA

TCGTGGATCATCAATTACAGGACAGGTTCCTCTGAGTAGACTAAAATACCGCCAAATTCTTAAAATTTCAAGAACATAACTACTACTATTCAAGCATCTA

AAATTTGCATTTTTAATAATAGGGTATCTAATCCTAGTTTTTTATAAAAATCTCATAAACTCATTTTTCACATTTAAAAAATTAATTATACTTACTAATT

TCACCTAATAAATACAATATAAATTAATAATAAAATAACTTATTATATACTGAACAAATTTAATTGCATTGTTTGTGTAACCGCAACTGCTGGCACAAAC

TTGGTCAATACTATTATAAATTCCTAAATCAAAATTTCTTTTAAATTTAATCTTCACTATTGCAATTCTTTAATTAAATATATAGAATAATCTATCTTTT

TAAAATAAATTCATTAAACACTAAAATTTACATATAAAATAATTTAAAAATTAAAATCTCAAGCTAGAATAAAACTTTATTTTTTTTTTAATATACATAA

ATTATATAAAATAAAAGTACCCCCC-TACCATTTTAGTTAAGTGAATTAATTTTTAATACCTTAAATTTATTCTCAATTTATTTTTATTATATAATTTTT

TAGTTTAAAATAAAACTTTATACCTAAAATTATATACATAAATTATATTTAGAATTTATAATTTAAAGAACTATAAAAATTAAATTAAAATTTTAAATCT

ATCTGATTTACAGATAAATAAATTAAATTAAACTAACTTACCTGATAAATTTAACTATATACGCACGGGTATATTAATTATTTAATTATAATATAATACA

TTTGTTTTATTAAATAATTATTAAGCAAAATAGCATTAATTTATTATATAAAAGATCTAGATATTATTAATTTAAAGTAAATTTTTTTTTTTTTTGTAGT

ATAATTTATACTAATATACGGCATTTAATATTTTCTATATAAATGTTTATATTATTTATATTTATCTATTTATAATTCTTAATAATAATTTAGATAATTA

TTAATTTATATATTAAATATTTATATATATATATATAATAATGAGGATATATATATATATATGTATATAGATAAATTAAATTTAGTTATATATAAAAAAA

TTAATATTAATTTTACTTTTAATTTTTTCTTTAATTTAAACTATTGGATATTCAGATTCTAATGTATTATAATATATCAACCATTAATGAATAACTTTAT

ATATATAATAAAAATGTTAATTAATATATCAATTCATATGAATATATAATATTAATAAATAAATTCTTAATAAATAATAATGATAATATATATATATTGT

ATATTAATGTACTATATATATAAATTCATTAAACATTTATATTTGATACCCCCCCAAAATTTTTTCTAAAAATGTGAAGTTTCAATTTAATTAAAATTTT

CTACAATGGTCAAAAACGGCCAAAAAATGGAAAATAAAATGTGCACAGAAAATGCACATAAAATGCACATTTTTGAAAATTTCCGATTTTTGAAAAACCG

TTTGAACCAAACCTCTGATGTACCTTTTTTCGCCCCCCCTGATTACTAAAATTTATCCTCAATTACTAAGGTTTATCCGCATTTTTGCGACAGGAATTTT

GAGTATAAAATCAATTCCACACTAAATTTTTTTTT-TTTTTTTTGTGTCATTTCTCCAACATGGGCAATTTTTTTTTTAGTCGAAGGACAAATTTTAGTA

AAAAAAGTTTGAAATCAGGTTAAACCCCTATTTAACCAAAAACTATTACTTTTTAATATTGTACCCAATTTTATTTAAAATAAATTTTATTATACTTTTA

TTTATGCAAAAAGATCTTCTTATAAACTTTT

>DMR127j_JP_Fukuoka_Kyushu

AATGAAGTGTCTGACTATAGAGTTACTTTGATAGAGTAAAAAAAGTGAATTTTCACCTTCATTATAATTAACAGAATTAAACTATTTCTTTAAGCTTCAA

AAACTTATGTACATTATATACTAAATTATAAAAAGATAAGCTAATTAAGCTACTGGGCTCATACCCCATCAATAAAGGTTACAATCCTTTTCTTTTTAAT

GTATTATAAGCTTTTATTTTTTAATTCTCTTATAATTGGAACCTTAATTGCTATTTCTTCCTACTCTTGGATAGGAATATGAATAGGATTAGAAATTAAT

CTTCTCTCTATGATCCCCCTCATTAGAGACAATAAAAATATAATAGCCTCAGAAGCTGCTTTAAAATATTTCATTATTCAAACAATAGCATCAACATTGT

TATTATTCTCAATTATTATAATATCAATGAAATTTATATATCAAATAAATTTAATCACTTACTTTAATTTAATTTTTAACACTTCATTATTCATCAAAAT

AGGAGCAGCCCCATTCCATTTTTGATTCCCCGAAATAATAGAAGGATTAAATTGATTAAATGCCATTATCATACTTACTTGACAAAAACTAAGACCTATA

GTATTATTAACATATTCTAATACAACCTCCATATATCTAATTTTAACAATTATATTTAGAATAATAATCAGAGGAATTATAGGTCTAAATCAAACTAGAT

TACGAAAAATTATAGCTTATTCATCTATCAACCATATTGGATGAATAATCAGTTCAATTATATTAATTGAAATTGTTTGATTTTACTATTTTATTATTTA

CTGCATTATTACTATTAATATCAGAATTATATTTATAAAATTAAATGTTTTTCATATCAATCAATTATATATTTCAATAAATTACCATATTTTACTTAAA

TTATTCTTTGCCTTAAATTTCATGTCTTTAGGAGGATTACCTCCATTTTTAGGATTTTTCCCTAAATGACTTACAATTCAAACTTTAATCCAAAGAAATA

TGTACTCAATTGCTTTCATTATAATTTTAATAACTTTAATAACACTTTTTTTTTACCTCCGAATTACTTTTTCAATTTTATTACTAAGAAAAACAGTTTT

AACATTTTACACACAACCAAAAATTTATACTAATTACATTATAGCATTTAATTTTATTACATTATTAAGATTAATTTTCGTTACTTTGATATTCAATTTC

TTATAAATTAAACATGAAGGATTTAAGTTAAATTAAACTAAGAACCTTCAAAGTTCTAAATAAAGTAAATTCTTTAAGCCTTAGGGCTTAGCCCATCTTT

AAATTTGCAATTTAAAATTCTTTTTGAACTATAAAGCTTGATAAAAGAAACTAATTTCGTATGTAAATTTACAGTTTACCGCCTAAACCTCGGCCATTTT

ATCGAATAAATGGCTATTCTCTACAAATCACAAAGACATCGGAACTTTATACTTTCTATTTGGAAGTTGGGCAGGTATAGTAGGCACTTCATTAAGTTTA

CTGATTCGTGCTGAATTAGGAAACCCGGGATCTCTAATTGGAGATGACCAAATTTATAACGTAATCGTTACAGCTCATGCTTTCATTATAATTTTTTTTA

TAGTAATACCTATTATAATTGGAGGATTTGGAAATTGATTAGTTCCTTTAATGCTTGGAGCCCCTGACATAGCATTCCCTCGAATAAATAACATAAGATT

TTGACTTTTACCTCCTTCATTAACTCTACTTCTAATAAGAAGATTAGTCGAAAGAGGAGCAGGTACAGGCTGAACAGTTTATCCCCCACTATCAGCCAAT

ATTGCCCATAGAGGAGCATCAGTTGATTTAGCAATTTTTAGACTTCATCTTGCAGGAATTAGTTCAATTTTAGGCGCAGTAAATTTCATTACCACTGTAA

TTAACATACGATCAACAGGAATAACTTTTGATCGAATACCCTTATTTGTTTGATCTGTAGTTTTAACAGCACTTCTTCTGCTATTATCTCTCCCCGTTTT

AGCAGGAGCAATTACTATACTATTAACAGATCGAAATATTAATACAACATTTTTTGATCCCGCAGGTGGAGGAGACCCTATTCTCTACCAACATTTATTT

TGATTTTTTGGTCATCCAGAAGTTTACATTTTAATTCTACCTGGATTTGGTATAATTTCCCATATTATTAGCCAAGAAAGAAGAAAAAAAGAAACATTTG

GAACTTTAGGTATAATTTATGCTATAATAGCTATTGGTTTATTAGGATTTATTGTTTGAGCTCATCATATATTTACAGTAGGTATAGATGTAGACACACG

GGCATATTTTACATCAGCAACAATAATCATTGCTGTTCCTACAGGAATTAAAATTTTTAGATGATTAGCTACTCTCCATGGATCACAATTAAACTACTCT

CCGTCTCTTTTATGAGCATTAGGATTTGTATTCCTATTTACAGTAGGAGGATTAACAGGAGTCATTCTAGCTAATTCATCAATTGACATTATTTTACATG

ATACTTACTATGTAGTTGCACATTTCCATTATGTCCTTTCCATAGGAGCTGTGTTTGCTATTATAGCAGGTTTTGTTCATTGATTCCCTTTATTTACAGG

TTTAACAATAAATTCAAAATTTCTTAAAATTCAATTTTTAACAATATTTATTGGTGTTAATATAACATTCTTCCCCCAACATTTCTTAGGATTAAGAGGA

ATACCCCGACGTTATTCAGATTATCCAGATGCTTATACAACTTGAAATATTATCTCATCTATTGGATCTTTAGTTTCTTTAATTAGTATTTTTATCTTTT

TATTCACTATTTGAGAAAGGCTAATTTCATTGCGAAAAAGAATTAGATCTTTAAGAATATCTACATCAATTGAATGACTTCAACAAATACCCCCTTCAGA

ACATAGTTATTCTGAACTTCCAATGCTTACTAACTTCTAATATGGCAGATTAGTGCAATGGATTTAAACCCCAAATATAAAGATTAAACTTTTTTTAGAA

ATAGCTACTTGAAATACCATTTTACTTCAAGATAGGGCATCCCCATTAATAGAACAACTCTCATTCTTTCATAATCATGCTCTTCTAATTCTCTTAATAA

TTACCGTTTTAGTAGGTTATTTAATAGGAACCTTATTTTTTAACCAATTTAATTACCGATTTTTATTAGATGGTCAAACTATTGAAATTATTTGAACTAT

TTTACCTGCTGTAACACTAATTTTTATCGCATTACCATCTTTACGCTTACTTTATCTTCTAGATGAAGTTAATAACCCTTTAGTAACTATCAAAACAATT

GGGCATCAATGATATTGATCATATGAATACAGAGATTTTATAAATTTTGAATTCGATTCCTATATAATTCCTTTGACAGAAATAAAACCTCAAAATTTTC

GTTTATTAGATGTTGATAACCGAGTAATTGTCCCCTTTAACTCCCAAATTCGAATGATAGTAACAGCTGCCGATGTTATTCATTCATGAACTATCCCAGC

TTTCAGTGTAAAAATTGATGCAACACCAGGCCGACTAAATCAAATTAGATTCCTAATTAATCGAACAGGATTATTTTATGGTCAATGCTCAGAAATTTGC

GGAGCAAATCATAGATTTATACCTATTACTGTAGAAAGAATTTCACCTTCATTTTTTACTAAATGAATCTCAAAAATAAATAACCTATCATTAGATGACT

GAAAGTAAGTAATGGTCTCTTAAACCAATTAATAGTAGTTTAACATCTACTTCTGATGGCCAAAAATTTAGTTAAGATATAACATTAGTTTGTCATACTA

AAATAATCATAATTTGATAATTTTTAATTCCACAAATAGCACCTTTAAACTGACTGTCTTTATTTTTTTTAATTATTATTATTTTTTTACTTTTTAATGT

ATTAAATTACTTTAGATTCTTACAACCCTTAAAAACCCAATCTCATAACCCTACAATTAAAAAAATTAATTGAAAATGATAACTAATTTATTTTCATCTT

TTGATCCTAGAACTTCTTTTAATTTAAGATTAAATTGATTAAGAATACTATTAGGGCTAATATTTATCCCCCCTATATTTTGATTAGTTCCTTCACGCCA

TAATTTTCTATGAATTAAAATTATTTTGACATTACACCAAGAATTTAAGGTTTTAATTGGTAATAATAATATTAAAGGAAGAACCTTAATATTTATTTCA

TTATTTTCTATAATTGTTTTCAATAACTTTCTAGGGTTATTTCCGTATATTTTTACAGGAACAAGACATTTAATTATAACATTATCTCTTGCCTTACCTT

TATGGATTAGATTCATATTATACGGGTGAATTAATAACACTATCCATATACTTGCACATTTAGTTCCTCAAGGAACACCTCCAGCTCTTATGGCATTCAT

AGTAGTAATTGAATCAATTAGAAATATTATTCGTCCTGGTACTTTAGCGGTTCGATTAGCAGCTAATATAATTGCTGGACATTTACTAATAACTTTACTA

GGAAACACAGGATTAAATTTATCATTTTTTATACTAAGTATTCTTATTATCACACAAATTCTTTTATTAATTTTAGAATCTGCTGTTGCTATTATCCAAT

CTTATGTATTTGCTGTATTAAGAACTTTATACTCTAGAGAAATTAATTAATGTCAAGACATAAAAATCACCCCTATCATTTAGTTGATGCAAGACCTTGA

CCTATTTTAGGTGCTTTTAGAGCTATAATCACAATAATTGGAATTATCAAATGATTCCATTTTTATAATAATTCTTTATTTTACTTAGGGACATTAATCA

CAATTTTAATCATAATTCAATGATGACGGGATATCACTCGTGAAGGAACTTTCCAAGGACTTCATACTTATGCTGTAACTATAGGTTTACGTTGAGGAAT

AATTTTATTTATTACATCAGAAGTATTTTTCTTTATTTCTTTTTTTTGAGCTTTTTTTCATAGTAGTTTAACACCCGCTATTGAACTAGGGATACTATGA

CCACCTAAAGGAATTACTCCATTTAACCCTATTCAAATTCCGTTATTAAATACGTTAATTCTTTTAACTTCAGGATTAACTGTAACTTGAGCTCATCATA

GATTAATAGAAAATGACTATAACCAAACAATACAAGGTCTTGGTTTAACAGTTTTATTAGGAGTATATTTTACTTTGTTACAAGGTTACGAATATTTAGA

AGCCCCCTTTACTATAGCAGATTCTGTTTATGGATCAACATTTTTTATTGCTACTGGTTTCCATGGATTACATGTTATTATTGGTACAACCTTCCTAGCT

GTTTGTTTAATACGACATTTTAATAACCATTTTACTTGTATCCATCACTTTGGGTTTGAAGCTGCTGCTTGATACTGGCATTTTGTTGATGTAGTATGAC

TATTTCTTTATATTTCTATTTACTGATGAGGTAGATATTTATATAGTATAATAATTATAATTGATTTCCAATCAAAAGATCTAAAAAAA-TTAGTATAAA

TAATCATTATAATTTGAAATATAAGGTTAATTATTTTTTCTATCTCTTTTATCTTAATTATATTATCTTTTATAATCTCTAAAAAAAGATTTATAGACCG

AGAAAAAGCTTCTCCATTCGAGTGTGGATTTGACCCCAAAAGATCGGCTCGTTTACCTTTTTCTTTACATTTTTTTTTAATTGCAGTAATTTTCTTAATT

TTTGATGTTGAAATTACTCTTCTTATCCCTTTAATTCTAACAATAAAAATTACTAATATTACTATATATACTTATATTGCTCTTTTCTTTTTAATGATTC

TTTTAATAGGACTTTATCATGAATGAAACCAAGGAGCCTTAAACTGAGCTCTTTAGGGTAATAGTTAAGTATAACATTTAAGTTGCATTTAAAAAGTATT

GATTTTTCAATTTACCTTAAATAAGAAACAATTAATTGTATTTAGTTTCGACCTAAAATTCAGGTGTATACACACCCTTATTTAAATTAATTGAAACCAA

AAAGAGGTATATCACTGTTAATGATATTAATGAGAAAAGCTCCAATTAAGGAAATAAGATATTCAAGAGTAAGCTTCTAACTTAACTCTTTAGCAGTGAA

AGTCTGTTAATATTTCTATTTATATAGTTTAATAAAACATTATTTTTTCATAATAAAATTAGAATAAATTTATTCTTATAAATATTTAAAAGTAAATTTT

ACTTCCCTGATAACTTCACTATCATACTCTACATAAGCTATTTAAATTAAATATATAAAATTATAAAAATTACCCATATTATAATTAAAAGTATAAAAAT

CTTATAATTGTTATTAAATATAAACTGTAAAAATATAGAAGTATTACTAATTTTACTATATAAATTTTGTCTTCCATAATACTCTGATCAACCTTGATCA

ATAGTTTTATATAACTTACTACCTAATTTAATTGGATAATAATTTAAACCAAATGTTGAAATATAAGGTATATTCCACATAGAAGAAAAAAAAAGTCTTG

AATTTAACAAATAAAAAGATTTTAATCTATCATTTAAAGTAAACTTAGAAAGTTCAAACCCAAATCAAGCCCCAAAAAATGATACAATTAAAGCTATAAT

TTTTATTATAAAAGGTAAACAAATAAAATAAGGTGTTGGGAATATCAACCATATTAATATTCTTCCCCCAACAATAACTAAAAAAATTAAACCCGATATC

CCTTGAAGTATAATTTTTCTATTATCATTAATTTTACTTAAAGAATAAAAAGAAAAATTTCCTACTAAAACATAATAAATTAAACGAAATGTATAACAAA

CCGTTAATCCTGTTGAAAAAAAGAAAATAACATAAATATAGATATTTAAATATCTTATAGATAAAACCTCTAAAATTAAATCCTTTGAATAAAACCCTGA

TAAAAAAGGTAAACCACATAATGCTAAATTTGAAATTATAAAATAAGTACAAGTTAAAGGTATAACTTTAATTAACCCCCCTATATACCGAATATCTTGA

CAATTTCTTAATCTGTGGATTATGCACCCTGCGCATATAAATAATAAAGCCTTAAATAAAGCGTGAGTTAATAGATGAAAAAAAGCTAATTGATACTCCC

CTAAAGCTAAAATACTAATTATCAAACCCAATTGTCTTAAAGTAGACAGAGCAATAATTTTTTTTAAATCAAACTCAAAGTTTGCACCTATTCCTGCTAT

AAATATAGTTATTGTTCCAATAAATAATAAAATTAATATTAAATTACTTGTTAATGCAAAATTAAAACGAATTAATAAGTAAACACCTGCTGTAACTAAA

GTAGAAGAATGAACTAAAGAAGAAACAGGTGTTGGAGCAGCTATTGCCGCAGGCAGCCAAGAAGAAAATGGAATTTGAGCTCTTTTAGTTATAGCTGCTA

ATATAATTAATAATATAATAATATATATCTCTATACTATTTTTATATATATCAATATAAAAAATATAGTTAAATCCCCCGAAATTTACTATTCATGCAAT

TGCTATTAATAAAGCAACATCTCCAATACGGTTAGTTAAAGCTGTAATTATCCCTGCATTATAAGATTTAATATTTTGATAATAAATAACTAAACAATAA

GAAACTAATCCTAATCCATCTCATCCTAATAAAATTCTAATCAAATTGGGAGAAATAATTAATAACATTATAGATAAAACAAATATAGAAACTAATATAA

TAAATCGATGCAAATAAATATCTCCTTCTATATACTCTTCACTATAGTAAATTACTATAGAAGAAACAAATAAAACAAAACTTATAAATAATAATGACAT

CCAGTCAAGTAAAATAGTTATAATAATTCTACAAGAATTAATCCTTAATATTTCATATTCTAATATTAGTCTATAATCTAAAATTATGAAATTTAAGCTT

AATAAAAATCTTAATACTCTAAAAAATAAAAACGTTACAAAATAAATTAAACAAATAGAAATAATTTAAAGTAAACTTTACATCTTTGATACCACAAATC

AATATTTTTTATTAAACTATTTAAATTACAATCATAAAACTAAAAATTCTCTCTTTAAAATTAAGATATTTAAAGGTAACCAATGAAGTAATAATAATAA

ATATTCACGAACAAATCCTCTTGAAAAAGAGTATAAATTTCTAACTAACTTACCATGTTGACTGTAAGAATATAAATATAAAGAATACGCAGCTCTAAAA

AAAGATATTAAAGATAAAAAAACCATTGTTCAACTTCTTCATCTAACCAATCTATTAATCAAAATAATCTCACCCAGTAAGTTTAATGAAGGAGGGGCCG

CTATATTACAACAACTAAACAAAAATCATCATATTCTTATTATTGGTATTAAATTAATTAACCCCTTATTTAAATAAATACTTCGACTATTTAGACGTTC

ATAAGAAATATTTGCTAAACAAAATAATCCTGAAGAACATAAACCATGGGCAATTATTATAACTAAAGCCCCTCTTATACCCCAATAACTTAAAGTTAAA

ATCCCTCTTAATACTAAACCTATATGTGCTACAGAAGAATAAGCAATTAAAGCTTTAATATCAACTTGACGTAAACATATTAATGAAACAAAAAATCCCC

CTACTATTCTAATTGTAATAAAAATATAGTTTACTTGTAAACCTACAGTTAAAAAAATATTTATTAAACGTATTAATCCATACCCCCCTAATTTTAATAT

AACTCCAGCTAAAATTATAGAGCCAGCAACTGGAGCTTCAACATGAGCTTTAGGAAGTCAAAGATGAACAAAAAATATAGGCATTTTAATAAAAAACACT

ATATTTATACATATAAATAACAAGAATCTTTTTACATCATAACATATAAAGAAAAAATCTAAAGAATGAAATTTTTCATAATAATAAAAAATCCTAATTA

TTATGGGTAATGAAGCAAATAATGTATAAAATAACAAGTAAACACCTGCCTGCAAACGCTCAGGTTGATATCCTCAACCAATAATTAATAGGAGAGTAGG

AATTAAGCTAAACTCAAAAAACAAATAAAAAATAAATAAATTTAAAGAACTAAATGTTATAACTAAAGACAATAATAAAATAATTATTACTAATAAAAAT

AAATTATAGAAATAGTTTTTTCTATAAATACTTTCAGAAGCTAATAATATCAAAGAACAAATCCATAAACTTAATAAAATTATTATAAAAGATAAAAGAT

CATACCCTATAAAATAAGAAATATTTATATACAAATAATTAAAACTAAATCTTAACCCAAATATAAATGTAATAAAAAAATATATATATTGATTAAATCA

GTATCTCTTTTTAATACAACTTAAAGGTACCAATATTAATATTATAAAAATAAACTTTATCATAATACATTAAAAGTTTGAAAATAATCATTTCCATGCG

TTCGTATTATAGAAACTAATAATGATAAACCTAAAGCTCCTTCACAAACTCTTATAGTTAAAAATACTATACCAAAATAAAATTCAAAATTAAAGTATAT

TAAAAATAGATATAAATTAAAGTATAATCCTAAAATAATATACTCTAACCTTAATAATATTAAAAGTAAATGTTTACGTTTAATACAAAAAGAAACTAAT

CCTGTAAAATATATAATCACTGAAAATAACATACAAAAAATTAACATTAGTTTTAATAATTTAATAAAAATACTGGTCTTGTAAATCAGAAATAAGAATT

TTCTTTTAAAACTTCAGAGAAAGAGTAAACCTCTGTCATTAATCTCCAAAATTAATATTTTAAATAAACTATTCTCTGTATAATCTTATTACTAATAACA

TTATCTTTTATTAGTTCAATAACTTTTATATTTTTAAGTCACCCTTTATCTATAGGGCTTATTTTATTAATACAAACTATTATTATAGCTTTAACTATAG

GTTTTTTTAATATTAATTTCTGATATTCTTATATTTTATTTCTCATTATAATTGGAGGAATATTAGTTTTATTTATTTATATAACAAGAGTAGCTTCAAA

TGAAAAATTTTCATTTTCTATTAAAATTACATTAATAATTGGAATTATAACTTTAGGATTTTTATTTAGTATTGCTATAATAGACCCCTACTTTTCAGAC

ATTAACTCAATTTATACAGAAAACTTAGATAATTATAAAGAATATAATATATCATTTAGAAAATATTTAAGTTATCCTAATATTATCATTATATACATAA

TAATTATTTATTTATTAATTACATTAATTGCAGTTGTAAAAATTACTCAAATTGAAAAAGGACCTTTACGTCAAACTAACTAATGAAAACACCATTACGA

AAAGCTTCCCCCCTATTAAAAATTATTAATAATAGAATTATTGATTTACCTACACCATCTAATATTTCTGCTTGATGAAATTTTGGGTCCTTATTAGGAC

TTTGTCTTTTTATTCAAATTATCACTGGAATTTTCTTAGCTATACATTTTACGGCCCATATTGACATAGCATTTAATAGAGTAATTCATATTTGTCGAGA

TGTAAATTATGGCTGATTATTACGAACAATTCATGCTAACGGAGCTTCCTTTTTCTTTATTTGTATTTATCTACATATTGGCCGAGGAATATATTATAGA

AGCTATAATTTACATTTAACATGAACTATTGGAGTAATCATCCTTTTTATAGTAATAGCAACAGCATTCTTAGGATATGTATTACCTTGAGGGCAAATAT

CTTTTTGAGGAGCTACTGTTATTACCAATCTTCTCTCTGCTATTCCTTATCTAGGAAATATGATTGTCCAATGATTATGAGGAGGATTTGCAGTAGATAA

CGCCACTTTAACTCGATTTTTTACATTACATTTTATTCTTCCTTTTATTATTTTAGCTTTAATAATTATTCATTTATTATTTCTTCATCAAACTGGTTCT

AATAATCCTTTAGGATTAAATAGAAATATCGATAAAGTACCTTTCCATCCGTATTTTACTTACAAAGATACTTTTGGATTCATTATAATAACTATATTAT

TAATATTTTTAGTTTTAATTAATCCCTATCTTTTGGGGGACCCAGAAAACTTTACACCTGCTAACCCTTTAGTAACCCCAGTTCATATTCAACCAGAATG

GTACTTTTTATTCGCATACGCTATTTTACGATCAATTCCTAATAAATTAGGTGGAGTAATTGCTTTAGTTATATCAATTGCTATTTTATTAATTATACCT

TTAATTAACAAAAAAAAATTTAGTAGAACTCAATTTTATCCATTAAATAAAATTTTATTTTGATTTTTTGTTTCTATTGTAATCTTATTGACATGAATCG

GAGCCCGCCCAGTGGAAGATCCATACATTTTAACAGGTCAAATTTTAACAATTATATATTTTAGTTATTATTTCTTAAATCCCTTAATTCATAAAATATG

AGATTATATTATTTTTAAAACTTAGTTAATGAACTTGTTAAAGTGTATATTTTGAAAATATAAAAAAGAGTTTATTCTCTATTAACTTTACTAAATTTTA

TTCACTAAATAAAATAAGAAAAGATAACCAACTTTAATCCTAAAAAGAAAAATAAAAAATTTAAAGAAACTGGTAAATAACTTTTTCAACATATATATAT

TAACTTATCGTAACGATACCGAGGTAAAGTACCTCGAACTCAAATCCAAATAAATGCTATAAAAGTTAACTTCAAAAAAAAAAATAAAGAAAAAATATCC

CCCCCTATAAATAACAACACACATAATATACTTATAAATAAAATTCTAGCATACTCAGCCAAAAAGATTAAAGCGAAACCCCCTCTTCTATACTCTACAT

TAAACCCGGAAACTAACTCTGATTCTCCTTCTGCGAAATCAAAAGGAGTTCGATTAGTCTCAGCTAAACTTGAAGATAATCATATTATTCTTAAAGGTAA

ACATAGAAAAAAAAATCATACTATTTCTTGATATTTTATAAAATCTATTATATTTAAATTTAAAATTAACAATAAAAAAGATAATAAAATTAATGATAAA

CTTACTTCGTATGAAATTGTTTGAGCTACTGATCGAATACCTCCTAATAAAGCATAATTAGAATTTGATGATCAACCAGAAATTATAATAGTATAGACTC

TTAACCTAGAACAACATAAAAAATATAAAATACCTAAATTAAACCTAAATATAAATGTCAAAAAAGGCATACATATCCATAATAAAAGAGCTAAAAATAA

ATTAAAAACTGGAGATATATAATAAATAAAAAAATTTGATATTAAAGGATAAGTTTGTTCTTTAGAAAATAACTTAATAGCATCACTAAAAGGTTGGGGA

ATACCTATAAATCCAACTTTATTGGGCCCTTTACGAATTTGAATATACCCTAAAACTTTACGCTCTATTAATGTTAAAAAAGCTACACCAATTAAAACAC

AAATAATTAAAATTAAACTTGTAAATAATAAAAGAAATAAATCTTGTAATATAATGTATTACTTGTGTTAAACACATATTTAAATTCTAAATTTAAAGCA

CTAATCTGCCAAAGTAATATTCATATTCAAATTATATTAAATTTTAAAGGTATCTGATCCTTTCGTACTAAAATACCTATATTTTTTAAAGATAGAAACC

AACCTGGCTCACGCCGGTTTAAACTCAGATCATGTAAAATTTTAAAGGTCGAACAGACCTAACCTTTTAGCCCCTACACCAAAAGTTAATTTTAATCCAA

CATCGAGGTCGCAAACTTTTTTTTCGATAAGAACTCTAAAAAAAAATTACGCTGTTATCCCTAAGGTAATTTAATCTTATAATCATTAAAAATGGATCAT

TCAATCATAAATTAATGTTTTTAAATAAAAAAAGTTTAATCAATTTTTCTGCTGCCCCAGCAAAATAGTTTAAATTATTAAATATATAAATATACTAAAA

TTAAATAATAATTTAAACTATAAAACTCTATAGGGTCTTCTCGTCTTTTAAAATTATATAAGCTTTTTTACTTATAAATAAAATTCTATATTCAATTAAA

TTGAGACAGTTACTTTCTCGTCCAACCGTTCATTCCAGCTTTCAATTAAAAAACTAATGATTATGCTACCTTTGCACGGTCAAATTACCGCGGCCATTCA

AATCCTCATTGGGCAGGTCAGACTTTAAATTATAATCAAAAAGACATGTTTTTAATAAACAGGCGAAAAGTATATTTGCCGAGTTCCTTAATTTAACCTT

GAAGTTTTAATTTAATTACTAAATTAAAATATATACTAATTTTATCATTATTCTATGTAAACCAATATTACATACATTATCTTAATAAACTACTTAAAAA

TAATATAAATCTTATTCTAACAAAAATTATTTATAACAAACTAAAGATTAACACTTCCAATTCTACTAATTTTTATTCAAAATATACATTTTTAACATTT

TATTTTAAAGCTTATCCCCTAAAATATTACTTTTTATATATAAAATACTAAATAATTAATATAATATAATAAAAAAACTAAATTAAATTTATTTCTTAAG

AAACTAGATATCTTAAAAAACGTATAACGTTTCATTTCTAATATAATATTTTAAAAATTTATGCCACAATTAAATTTATATTATATTAGCTCTTTATAAT

TCGAGAACACCAAATAATTAAATTATTTTAATAAACCCTGATACACAAGGTACAAAAAATTAATTTTTCTTTTTAAAAAATAAATCTCTATATATTTATA

TTATCTATCTCTATACAAATTAACTATAATAAAATTTTTATATTCTAAAATATACTAATATCAAAAATATTTTTTTTATATATATATATATA----TTAT

AAATTTTTCCTTTCAAATTAAATTGATTTTCACAACTAACTTTTTAATGTAAATAAAATGCTTTATTACAAGCTCTAATTTGCCATTCCAGGTACACTTT

CCAGTACACCTACTATGTTACGACTTATCCCTCTTTAGAGAGGGAGCGACGGGCGATATGTACATATTCTAGAGCTATACTCATATAATTAAACTAAACT

ATATTACTTTCAAATCCACTTTATAAAATAATGTTAATTATTTTAACCATCTAAATAATTTTATTGTAACCCATCTCTCCTTATCTATGCGCTGTATCTT

GATCTGATTTTTTTTATACTTATAAATTTTGAACATTCCAAATTCTTTAAAAACATTCAACCTACGACGATATACAAACCTTTAAAATAAGTACGATTAA

TCGTGGATCATCAATTACAGGACAGGTTCCTCTGAGTAGACTAAAATACCGCCAAATTCTTAAAATTTCAAGAACATAACTACTACTATTCAAGCATCTA

AAATTTGCATTTTTAATAATAGGGTATCTAATCCTAGTTTTTTATAAAAATCTCATAAACTCATTTTTCACATTTAAAAAATTAATTATACTTACTAATT

TCACCTAATAAATACAATATAAATTAATAATAAAATAACTTATTATATACTGAACAAATTTAATTGCATTGTTTGTGTAACCGCAACTGCTGGCACAAAC

TTGGTCAATACTATTATAAATTCCTAAATCAAAATTTCTTTTAAATTTAATCTTCACTATTGCAATTCTTTAATTAAATATATAGAATAATCTATCTTTT

TAAAATAAATTCATTAAACACTAAAATTTACATATAAAATAATTTAAAAATTAAAATCTCAAGCTAGAATAAAACTTTATTTTTTTTTTAATATACATAA

ATTATATAAAATAAAAGTACCCCCC-TACCATTTTAGTTAAGTGAATTAATTTTTAATACCTTAAATTTATTCTCAATTTATTTTTATTATATAATTTTT

TAGTTTAAAATAAAACTTTATACCTAAAATTATATACATAAATTATATTTAGAATTTATAATTTAAAGAACTATAAAAATTAAATTAAAATTTTAAATCT

ATCTAATTTACAGATAAACAAATTAAATTAAACTAACTTACCTGATAAATTTAACTATATACGCACGGGTATATTAATTATTTAATTATAATATAATACA

TTTGTTTTATTAAATAATTATTAAGCAAAATAGCATTAATTTATTATATAAAAGATCTAGATACTATTAATTTAAAGTAAATTTTTTTTTTTTTTGTAGT

ATAATTTATACTAATATACGGCATTTAATATTTTCTATATAAATGTTTATATTATTTATATTTATCTATTTATAATTCTTAATAATAATTTAGATAATTA

TTAATTTATATATTAAATATTTATATATATATATATAATAATGAGGATATATATATATATATGTATATAGATAAATTAAATTTAGTTATATATAAAAAAA

TTAATATTAATTTTACTTTTAATTTTTTCTTTAATTTAAACTATTGGATATTCAGATTCTAATGTATTATAATATATCAACCATTAATGAATAACTTTAT

ATATATAATAAAAATGTTAATTAATATATCAATTCATATGAATATATAATATTAATAAATAAATTCTTAATAAATAATAATGATAATATATATATATTGT

ATATTAATGTACTATATATATAAATTCATTAAACATTTATATTTGATACCCCCCCAAAATTTTTTCTAAAAATGTGAAGTTTCAATTTAATTAAAATTTT

CTACAATGGTCAAAAACGGCCAAAAAATGGAAAATAAAATGTGCACAGAAAATGCACATAAAATGCACATTTTTGAAAATTTCCGATTTTTGAAAAACCG

TTTGAACCAAACCTCTGATGTACCTTTTTTCGCCCCCCCTGATTACTAAAATTTATCCTCAATTACTAAGGTTTATCCGCATTTTTGCGACAGGAATTTT

GAGTATAAAATCAATTCCACACTAAATTTTTTTTT-TTTTTTTTGTGTCATTTCTCCAACATGGGCAATTTTTTTTTTAGTCGAAGGACAAATTTTAGTA

AAAAAAGTTTGAAATCAGGTTAAACCCCTATTTAACCAAAAACTATTACTTTTTAATATTGTACCCAATTTTATTTAAAATAAATTTTATTATACTTTTA

TTTATGCAAAAAGATCTTCTTATAAACTTTT

>DMR128j_JP_Fukuoka_Kyushu

AATGAAGTGTCTGACTATAGAGTTACTTTGATAGAGTAAAAAAAGTGAATTTTCACCTTCATTATAATTAACAGAATTAAACTATTTCTTTAAGCTTCAA

AAACTTATGTACATTATATACTAAATTATAAAAAGATAAGCTAATTAAGCTACTGGGCTCATACCCCATCAATAAAGGTTACAATCCTTTTCTTTTTAAT

GTATTATAAGCTTTTATTTTTTAATTCTCTTATAATTGGAACCTTAATTGCTATTTCTTCCTACTCTTGGATAGGAATATGAATAGGATTAGAAATTAAT

CTTCTCTCTATGATCCCCCTCATTAGAGACAATAAAAATATAATAGCCTCAGAAGCTGCTTTAAAATATTTCATTATTCAAACAATAGCATCAACATTGT

TATTATTCTCAATTATTATAATATCAATGAAATTTATATATCAAATAAATTTAATCACTTACTTTAATTTAATTTTTAACACTTCATTATTCATCAAAAT

AGGAGCAGCCCCATTCCATTTTTGATTCCCCGAAATAATAGAAGGATTAAATTGATTAAATGCCATTATCATACTTACTTGACAAAAACTAAGACCTATA

GTATTATTAACATATTCTAATACAACCTCCATATATCTAATTTTAACAATTATATTTAGAATAATAATCAGAGGAATTATAGGTCTAAATCAAACTAGAT

TACGAAAAATTATAGCTTATTCATCTATCAACCATATTGGATGAATAATCAGTTCAATTATATTAATTGAAATTGTTTGATTTTACTATTTTATTATTTA

CTGCATTATTACTATTAATATCAGAATTATATTCATAAAATTAAATGTTTTTCATATCAATCAATTATATATTTCAATAAATTACCATATTTTACTTAAA

TTATTCTTTGCCTTAAATTTCATGTCTTTAGGAGGATTACCTCCATTTTTAGGATTTTTCCCTAAATGACTTACAATTCAAACTTTAATCCAAAGAAATA

TACACTCAATTGCTTTCATTATAATTTTAATAACTTTAATAACACTTTTTTTTTACCTCCGAATTACTTTTTCAATTTTATTACTAAGAAAAACAGTTTT

AACATTTTACACACAACCAAAAATTTATACTAATTACATTATAGCATTTAATTTTATTACATTATTAAGATTAATTTTCGTTACTTTGATATTCAATTTC

TTATAAATTAAACATGAAGGATTTAAGTTAAATTAAACTAAGAACCTTCAAAGTTCTAAATAAAGTAAATTCTTTAAGCCTTAGGGCTTAGCCCATCTTT

AAATTTGCAATTTAAAATTCTTTTTGAACTATAAAGCTTGATAAAAGAAACTAATTTCGTATGTAAATTTACAGTTTACCGCCTAAACCTCGGCCATTTT

ATCGAATAAATGGCTATTCTCTACAAATCACAAAGACATCGGAACTTTATACTTTCTATTTGGAAGTTGGGCAGGTATAGTAGGCACTTCATTAAGTTTA

CTGATTCGTGCTGAATTAGGAAACCCGGGATCTCTAATTGGAGATGACCAAATTTATAACGTAATCGTTACAGCTCATGCTTTTATTATAATTTTTTTTA

TAGTAATACCTATTATAATTGGAGGATTTGGAAATTGATTAGTTCCTTTAATGCTTGGAGCCCCTGACATAGCATTCCCTCGAATAAATAACATAAGATT

TTGACTTTTACCTCCTTCATTAACTCTACTTCTAATAAGAAGATTAGTCGAAAGAGGAGCAGGTACAGGCTGAACAGTTTATCCCCCACTATCAGCCAAT

ATTGCCCATAGAGGAGCATCAGTTGATTTAGCAATTTTTAGACTTCATCTTGCAGGAATTAGTTCAATTTTAGGCGCAGTAAATTTCATTACCACTGTAA

TTAACATGCGATCAACAGGAATAACTTTTGATCGAATACCCTTATTTGTTTGATCTGTAGTTTTAACAGCACTTCTTCTGCTATTATCTCTCCCCGTTTT

AGCAGGAGCAATTACTATACTATTAACAGATCGAAATATTAATACAACATTTTTTGATCCCGCAGGTGGAGGAGACCCTATTCTCTACCAACATTTATTT

TGATTTTTTGGTCATCCAGAAGTTTACATTTTAATTCTACCCGGATTTGGTATAATTTCCCATATTATTAGCCAAGAAAGAAGAAAAAAAGAAACATTTG

GAACTTTAGGTATAATTTATGCTATAATAGCTATTGGTTTATTAGGATTTATTGTTTGAGCTCATCATATATTTACAGTAGGTATAGATGTAGACACACG

GGCATATTTTACATCGGCAACAATAATCATTGCTGTTCCTACAGGAATTAAAATTTTTAGATGATTAGCTACTCTCCATGGATCACAATTAAACTACTCT

CCGTCTCTTTTATGAGCATTAGGATTTGTATTCCTATTTACAGTAGGAGGATTAACAGGAGTCATTCTAGCTAATTCATCAATTGACATTATTTTACATG

ATACTTACTATGTAGTTGCACATTTTCATTATGTCCTTTCCATAGGAGCTGTGTTTGCTATTATAGCAGGTTTTGTTCATTGATTCCCTTTATTTACAGG

TTTAACAATAAATTCAAAATTTCTTAAAATTCAATTTTTAACAATATTTATTGGTGTTAATATAACATTCTTCCCCCAACATTTCTTAGGATTAAGAGGA

ATACCCCGACGTTATTCAGATTATCCAGATGCTTATACAACTTGAAATATTATCTCATCTATTGGATCTTTAGTTTCTTTAATTAGTATTTTTATCTTTT

TATTCACTATTTGAGAAAGGCTAATTTCATTGCGAAAAAGAATTAGATCTTTAAGAATATCTACATCAATTGAATGACTTCAACAAATACCCCCTTCAGA

ACATAGTTATTCTGAACTTCCAATGCTTACTAACTTCTAATATGGCAGATTAGTGCAATGGATTTAAACCCCAAATATAAAGATTAAACTTTTTTTAGAA

ATAGCTACTTGAAATACCATTTTACTTCAAGATAGGGCATCCCCATTAATAGAACAACTCTCATTCTTTCATAATCATGCTCTTCTAATTCTCTTAATAA

TTACCGTTTTAGTAGGTTATTTAATAGGAACCTTATTTTTTAACCAATTTAATTACCGATTTTTATTAGATGGTCAAACTATTGAAATTATTTGAACTAT

TTTACCTGCTGTAACACTAATTTTTATCGCATTACCATCTTTACGCTTACTTTATCTTCTAGATGAAGTTAATAACCCTTTAGTAACTATCAAAACAATT

GGGCATCAATGATATTGATCATATGAATACAGAGATTTTATAAATTTTGAATTCGATTCCTATATAATTCCTTTGACAGAAATAAAACCTCAAAATTTTC

GTTTATTAGATGTTGATAACCGAGTAATTGTCCCCTTTAACTCCCAAATTCGAATGATAGTAACAGCTGCCGATGTTATTCATTCATGAACTATCCCAGC

TTTCAGTGTAAAAATTGATGCAACACCAGGCCGACTAAATCAAATTAGATTCCTAATTAATCGAACAGGATTATTTTATGGTCAATGCTCAGAAATTTGC

GGAGCAAATCATAGATTTATACCTATTACTGTAGAAAGAATTTCACCTTCATTTTTTACTAAATGAATCTCAAAAATAAATAACCTATCATTAGATGACT

GAAAGTAAGTAATGGTCTCTTAAACCAATTAATAGTAGTTTAACATCTACTTCTGATGGCCAAAAATTTAGTTAAGATATAACATTAGTTTGTCATACTA

AAATAATCATAATTTGATAATTTTTAATTCCACAAATAGCACCTTTAAACTGACTGTCTTTATTTTTTTTAATTATTATTATTTTTTTACTTTTTAATGT

ATTAAATTACTTTAGATTCTTACAACCCTTAAAAACCCAATCTCATAGCCCTACAATTAAAAAAATTAATTGAAAATGATAACTAATTTATTTTCATCTT

TTGATCCTAGAACTTCTTTTAATTTAAGATTAAATTGATTAAGAATACTATTAGGGCTAATATTTATCCCACCTATATTTTGATTAGTTCCTTCACGCCA

TAATTTTCTATGAATTAAAATTATTTTGACATTACACCAAGAATTTAAGGTTTTAATTGGTAATAATAATATTAAAGGAAGAACCTTAATATTTATTTCA

TTATTTTCTATAATTGTTTTCAATAACTTTCTAGGGTTATTTCCGTATATTTTTACAGGAACAAGACATTTAATTATAACATTATCTCTTGCCTTACCTT

TATGGATTAGATTCATATTATACGGGTGAATTAATAACACTATCCATATACTTGCACATTTAGTTCCTCAAGGAACACCTCCAGCTCTTATGGCATTCAT

AGTAGTAATTGAATCAATTAGAAATATTATTCGTCCTGGTACTTTAGCGGTTCGATTAGCTGCTAATATAATTGCTGGACATTTACTAATAACTTTACTA

GGAAACACAGGATTAAATTTATCATTTTTTATACTAAGTATTCTTATTATAACACAAATTCTTTTATTAATTTTAGAATCTGCTGTTGCTATTATCCAAT

CTTATGTATTTGCTGTATTAAGAACTTTATACTCTAGAGAAATTAATTAATGTCAAGACATAAAAATCACCCCTATCATTTAGTTGATGCAAGACCTTGA

CCTATTTTAGGTGCTTTTAGAGCTATAATCACAATAATTGGAATTATCAAATGATTCCATTTTTATAATAATTCTTTATTTTACTTAGGGACATTAATCA

CAATTTTAATCATAATTCAATGATGACGGGATATCACTCGTGAAGGAACTTTTCAAGGACTTCATACTTATGCTGTAACTATAGGTTTACGTTGAGGAAT

AATTTTATTTATTACATCAGAAGTATTTTTCTTTATTTCTTTTTTTTGAGCTTTTTTTCATAGTAGTTTAACACCCGCTATTGAACTAGGGATACTATGA

CCACCTAAAGGAATTACTCCATTTAACCCTATTCAAATTCCGTTATTAAATACGTTAATTCTTTTAACTTCAGGATTAACTGTAACTTGAGCTCATCATA

GATTAATAGAAAATGACTATAACCAAACAATACAAGGTCTTGGTTTAACAGTTTTATTAGGAGTATATTTTACTTTGTTACAAGGTTACGAATATTTAGA

AGCCCCCTTTACTATAGCAGATTCTGTTTATGGATCAACATTTTTTATTGCTACTGGTTTCCATGGATTACATGTTATTATTGGTACAACCTTCCTAGCT

GTTTGTTTAATACGACATTTTAATAACCATTTTACTTGTATCCATCACTTTGGGTTTGAAGCTGCTGCTTGATACTGACATTTTGTTGATGTAGTATGAC

TATTTCTTTATATTTCTATTTACTGATGAGGTAGATATTTATATAGTATAATAATTATAATTGATTTCCAATCAAAAGATCTAAAAAAA-TTAGTATAAA

TAATCATTATAATTTGAAATATAAGGTTAATTATTTTTTCTATCTCTTTTATCTTAATTATATTATCTTTTATAATCTCTAAAAAAAGATTTATAGACCG

AGAAAAAGCTTCTCCATTCGAGTGTGGATTTGACCCCAAAAGATCGGCTCGTTTACCTTTTTCTTTACATTTTTTTTTAATTGCAGTAATTTTCTTAATT

TTTGATGTTGAAATTACTCTTCTTATCCCTTTAATTCTAACAATAAAAATTACTAATATTACTATATATACTTATATTGCTCTTTTCTTTTTAATGATTC

TTTTAATAGGACTTTATCATGAATGAAACCAAGGAGCCTTAAACTGAGCTCTTTAGGGTAATAGTTAAGTATAACATTTAAGTTGCATTTAAAAAGTATT

GATTTTTCAATTTACCTTAAATAAGAAACAATTAATTGTATTTAGTTTCGACCTAAAATTCAGGTGTATACACACCCTTATTTAAATTAATTGAAACCAA

AAAGAGGTATATCACTGTTAATGATATTAATGAGAAAAGCTCCAATTAAGGAAATAAGATATTCAAGAGTAAGCTTCTAACTTAACTCTTTAGCAGTGAA

AGTCTGTTAATATTTCTATTTATATAGTTTAATAAAACATTATTTTTTCATAATAAAATTAGAATAAATTTATTCTTATAAATATTTAAAAGTAAATTTT

ACTTCCCTGATAACTTCACTATCATACTCTACATAAGCTATTTAAATTAAATATATAAAATTATAAAAATTACCCATATTATAATTAAAAGTATAAAAAT

CTTATAATTGTTATTAAATATAAACTGTAAAAATATAGAAGTATTACTAATTTTACTATATAAATTTTGTCTTCCATAATACTCTGATCAACCTTGATCA

ATAGTTTTATATAACTTACTACCTAATTTAATTGGATAATAATTTAAACCAAATGTTGAAATATAAGGTATATTCCACATAGAAGAAAAAAAAAGTCTTG

AATTTAACAAATAAAAAGATTTTAATCTATCATTTAAAGTAAACTTAGAAAGTTCAAACCCAAATCAAGCCCCAAAAAATGATACAATTAAAGCTATAAT

TTTTATTATAAAAGGTAAACAAATAAAATAAGGTGTTGGGAATATCAACCATATTAATATTCTTCCCCCAACAATAACTAAAAAAATTAAACCCGATATC

CCTTGAAGTATAATTTTTCTATTATCATTAATTTTACTTAAAGAATAAAAAGAAAAATTTCCTACTAAAACATAATAAATTAAACGAAATGTATAACAAA

CCGTTAATCCTGTTGAAAAAAAGAAAATAACATAAATATAGATATTTAAATATCTTATAGATAAAACCTCTAAAATTAAATCCTTTGAATAAAACCCTGA

TAAAAAAGGTAAACCACATAATGCTAAATTTGAAATTATAAAATAAGTACAAGTTAAAGGTATAACTTTAATTAACCCCCCTATATACCGAATATCTTGA

CAATTTCTTAATCTGTGGATTATGCACCCTGCGCATATAAATAATAAAGCCTTAAATAAAGCGTGAGTTAATAGATGAAAAAAAGCTAATTGATACTCCC

CTAAAGCTAAAATACTAATTATCAAACCCAATTGTCTTAAAGTAGACAGAGCAATAATTTTTTTTAAATCAAACTCAAAGTTTGCACCTATTCCTGCTAT

AAATATAGTTATTGTTCCAATAAATAATAAAATTAATATTAAATTACTTGTTAATGCAAAATTAAAACGAATTAATAAGTAAACACCTGCTGTAACTAAG

GTAGAAGAATGAACTAAAGAAGAAACAGGTGTTGGGGCAGCTATTGCCGCAGGCAGCCAAGAAGAAAATGGAATTTGAGCTCTTTTAGTTATAGCTGCTA

ATATAATTAATAATATAATAATATATATCTCTATACTATTTTTATATATATCAATATAAAAAATATAGTTAAATCCCCCGAAATTTACTATTCATGCAAT

TGCTATTAATAAAGCAACATCTCCAATACGGTTAGTTAAAGCTGTAATTATCCCTGCATTATAAGATTTAATATTTTGATAATAAATAACTAAACAATAA

GAAACTAATCCTAATCCATCTCATCCTAATAAAATTCTAATCAAATTGGGAGAAATAATTAATAACATTATAGATAAAACAAATATAGAAACTAATATAA

TAAATCGATGCAAATAAATATCTCCTTCTATATACTCTTCACTATAGTAAATTACTATAGAAGAAATAAATAAAACAAAACTTATAAATAATAATGACAT

TCAGTCAAGTAAAATAGTTATAATAATTCTACAAGAATTAATCCTTAATATTTCATATTCTAATATTAGTCTATAATCTAAAATTATGAAATTTAAGCTT

AATAAAAATCTTAATACTCTAAAAAATAAAAACGTTACAAAATAAATTAAACAAATAGAAATAATTTAAAGTAAACTTTACATCTTTGATACCACAAATC

AATATTTTTTATTAAACTATTTAAATTACAATCATAAAACTAAAAATTCTCTCTTTAAAATTAAGATATTTAAAGGTAATCAATGAAGTAATAATAATAA

ATATTCACGAACAAATCCTCTTGAAAAAGAGTATAAATTTCTAACTAACTTACCATGTTGACTGTAAGAATATAAATATAAAGAATACGCAGCTCTAAAA

AAAGATATTAAAGATAAAAAAACCATTGTTCAACTTCTTCATCTAACCAATCTATTAATCAAAATAATCTCACCCAATAAGTTTAATGAAGGAGGGGCCG

CTATATTACAACAACTAAACAAAAATCATCATATTCTTATTATTGGTATTAAATTAATTAACCCCTTATTTAAATAAATACTTCGACTATTTAGACGTTC

ATAAGAAATATTTGCTAAACAAAATAATCCTGAAGAACATAAACCATGGGCAATTATTATAACTAAAGCCCCTCTTATACCCCAATAACTTAAAGTTAAA

ATCCCTCTTAATACTAAACCTATATGTGCTACAGAAGAATAAGCAATTAAAGCTTTAATATCAACTTGACGTAAACATATTAATGAAACAAAAAATCCCC

CTACTATTCTAATTGTAATAAAAATATAGTTTACTTGTAAACCTACAGTTAAAAAAATATTTATTAAACGTATTAATCCATACCCCCCTAATTTTAATAT

AACTCCAGCTAAAATTATAGAGCCAGCAACTGGAGCTTCAACATGAGCTTTAGGAAGTCAAAGATGAACAAAAAATATAGGCATTTTAATAAAAAACACT

ATATTTATACATATAAATAACAAGAATCTTTTTACATCATAACATATAAAGAAAAAATCTAAAGAATGAAATTTTTCATAATAATAAAAAATCCTAATTA

TTATGGGTAATGAAGCAAATAATGTATAAAATAACAAGTAAACACCTGCCTGCAAACGCTCAGGTTGATACCCTCAACCAATAATTAATAGGAGAGTAGG

AATTAAGCTAAACTCAAAAAACAAATAAAAAATAAATAAATTTAAAGAACTAAATGTTATAACTAAAGACAATAATAAAATAATTATTACTAATAAAAAT

AAATTATAGAAATAGTTTTTTCTATAAATACTTTCAGAAGCTAATAATATCAAAGAACAAATCCACAAACTTAATAAAATTATTATAAAAGATAAAAGAT

CATACCCTATAAAATAAGAAATATTTATATACAAATAATTAAAACTAAATCTTAACCCAAATATAAATGTAATAAAAAAATATATATATTGATTAAATCA

GTATCTCTTTTTAATACAACTTAAAGGTACCAATATTAATATTATAAAAATAAACTTTATCATAATACATTAAAAGTTTGAAAATAATCATTTCCATGCG

TTCGTATTATAGAAACTAATAATGATAAACCTAAAGCTCCTTCACAAACTCTTATAGTTAAAAATACTATACCAAAATAAAATTCAAAATTAAAGTATAT

TAAAAATAGATATAAATTAAAGTATAATCCTAAAATAATATACTCTAACCTTAATAATATTAAAAGTAAATGTTTACGTTTAATACAAAAAGAAACTAAT

CCTGTAAAATATATAATCACTGAAAATAACATACAAAAAATTAACATTAGTTTTAATAATTTAATAAAAATACTGGTCTTGTAAATCAGAAATAAGAATT

TTCTTTTAAAACTTCAGAGAAAGAGTAAACCTCTGTCATTAATCTCCAAAATTAATATTTTAAATAAACTATTCTCTGTATAATCTTATTACTAATAACA

TTATCTTTTATTAGTTCAATAACTTTTATATTTTTAAGTCACCCTTTATCTATAGGGCTTATTTTATTAATACAAACTATTATTATAGCTTTAACTATAG

GTTTTTTTAATATTAATTTCTGATATTCTTATATTTTATTTCTCATTATAATTGGAGGAATATTAGTTTTATTTATTTATATAACAAGAGTAGCTTCAAA

TGAAAAATTTTCATTTTCTATTAAAATTACATTAATAATTGGAATTATAACTTTAGGATTTTTATTTAGTATTGCTATAATAGACCCCTACTTTTCAGAC

ATTAACTCAATTTATACAGAAAACTTAGATAATTATAAAGAATATAATATATCATTTAGAAAATATTTAAGTTATCCTAATATTATCATTATATACATAA

TAATTATTTATTTATTAATTACATTAATTGCAGTTGTAAAAATTACTCAAATTGAAAAAGGACCTTTACGTCAAACTAACTAATGAAAACACCATTACGA

AAAGCTTCCCCCCTATTAAAAATTATTAATAATAGAATTATTGATTTACCTACACCATCTAATATTTCTGCTTGATGAAATTTTGGGTCCTTATTAGGAC

TTTGTCTTTTTATTCAAATTATCACTGGAATTTTCTTAGCTATACATTTTACGGCCCATATTGACATAGCATTTAATAGAGTAATTCATATTTGTCGAGA

TGTAAATTATGGCTGATTATTACGAACAATTCATGCTAACGGAGCTTCCTTTTTCTTTATTTGTATTTATCTACATATTGGCCGAGGAATATATTATAGA

AGCTATAATTTACATTTAACATGAACTATTGGAGTAATCATCCTTTTTATAGTAATAGCAACAGCATTCTTAGGATATGTATTACCTTGAGGGCAAATAT

CTTTTTGAGGAGCTACTGTTATTACCAATCTTCTCTCTGCTATTCCTTACCTAGGAAATATGATTGTCCAATGATTATGAGGAGGATTTGCAGTAGATAA

CGCCACTTTAACTCGATTTTTTACATTACATTTTATTCTTCCTTTTATTATTTTAGCTTTAATAATTATTCATTTATTATTTCTTCATCAAACTGGTTCT

AATAATCCTTTAGGATTAAATAGAAATATTGATAAAGTACCTTTCCATCCGTATTTTACTTACAAAGATACTTTTGGATTCATTATAATAACTATATTAT

TAATATTTTTAGTTTTAATTAATCCCTATCTTTTGGGGGACCCAGAAAACTTTACACCTGCTAACCCTTTAGTAACCCCAGTTCATATCCAACCAGAATG

GTACTTTTTATTCGCATACGCTATTTTACGATCAATTCCTAATAAATTAGGTGGAGTAATTGCTTTAGTTATATCAATTGCTATTTTATTAATTATACCT

TTAATTAACAAAAAAAAATTTAGTAGAACTCAATTTTATCCATTAAATAAAATTTTATTTTGATTTTTTGTTTCTATTGTAATCTTATTGACATGAATCG

GAGCCCGCCCAGTGGAAGATCCATACATTTTAACAGGTCAAATTTTAACAATTATATATTTTAGTTATTATTTCTTAAATCCCTTAATTCATAAAATATG

AGATTATATTATTTTTAAAACTTAGTTAATGAACTTGTTAAAGTGTATATTTTGAAAATATAAAAAAGAGTTTATTCTCTATTAACTTTACTAAATTTTA

TTCACTAAATAAAATAAGAAAAGATAACCAACTTTAATCCTAAAAAGAAAAATAAAAAATTTAAAGAAACTGGTAAATAACTTTTTCAACATATATATAT

TAACTTATCGTAACGATACCGAGGTAAAGTACCTCGAACTCAAATCCAAATAAATGCTATAAAAGTTAACTTCAAAAAAAAAAATAAAGAAAAAATATCC

CCCCCTATAAATAACAACACACATAATATACTTATAAATAAAATTCTAGCATACTCAGCCAAAAAGATTAAAGCGAAACCCCCTCTTCTATACTCTACAT

TAAACCCGGAAACTAACTCTGATTCTCCTTCTGCGAAATCAAAAGGAGTTCGATTAGTCTCAGCTAAACTTGAAGATAATCATATTATTCTTAAAGGTAA

ACATAGAAAAAAAAATCATACTATTTCTTGATATTTTATAAAATCTATTATATTTAAATTTAAAATTAACAATAAAAAAGATAATAAAATTAATGATAAA

CTTACTTCGTATGAAATTGTTTGAGCTACTGATCGAATACCTCCTAATAAAGCATAATTAGAATTTGATGATCAACCAGAAATTATAATAGTATAGACTC

TTAACCTAGAACAACATAAAAAATATAAAATACCTAAATTAAACCTAAATATAAATGTCAAAAAAGGCATACATATCCATAATAAAAGAGCTAAAAATAA

ATTAAAAACTGGAGATATATAATAAATAAAAAAATTTGATATTAAAGGATAAGTTTGTTCTTTAGAAAATAACTTAATAGCATCACTAAAAGGTTGGGGA

ATACCTATAAATCCAACTTTATTGGGCCCTTTACGAATTTGAATATACCCTAAAACTTTACGCTCTATTAATGTTAAAAAGGCTACACCAATTAAAACAC

AAATAATTAAAATTAAACTTGTAAATAATAAAAGAAATAAATCTTGTAATATAATGTATTACTTGTGTTAAACACATATTTAAATTCTAAATTTAAAGCA

CTAATCTGCCAAAGTAATATTCATATTCAAATTATATTAAATTTTAAAGGTATCTGATCCTTTCGTACTAAAATACCTATATTTTTTAAAGATAGAAACC

AACCTGGCTCACGCCGGTTTAAACTCAGATCATGTAAAATTTTAAAGGTCGAACAGACCTAACCTTTTAGCCCCTACACCAAAAGTTAATTTTAATCCAA

CATCGAGGTCGCAAACTTTTTTTTCGATAAGAACTCTAAAAAAAAATTACGCTGTTATCCCTAAGGTAATTTAATCTTATAATCATTAAAAATGGATCAT

TCAATCATAAATTAATGTTTTTAAATAAAAAAAGTTTAATCAATTTTTCTGCTGCCCCAGCAAAATAGTTTAAATTATTAAATATATAAATATACTAAAA

TTAAATAATAATTTAAACTATAAAACTCTATAGGGTCTTCTCGTCTTTTAAAATTATATAAGCTTTTTTACTTATAAATAAAATTCTATATTCAATTAAA

TTGAGACAGTTACTTTCTCGTCCAACCGTTCATTCCAGCTTTCAATTAAAAAACTAATGATTATGCTACCTTTGCACGGTCAAATTACCGCGGCCATTCA

AATCCTCATTGGGCAGGTCAGACTTTAAATTATAATCAAAAAGACATGTTTTTAATAAACAGGCGAAAAGTATATTTGCCGAGTTCCTTAATTTAACCTT

GAAGTTTTAATTTAATTACTAAATTAAAATATATACTAATTTTATCATTATTCTATGTAAACCAATATTACATACATTATCTTAATAAACTACTTAAAAA

TAATATAAATCTTATTCTAACAAAAATTATTTATAACAAACTAAAGATTAACACTTCCAATTCTACTAATTTTTATTCAAAATATACATTTTTAACATTT

TATTTTAAAGCTTATCCCCTAAAATATTACTTTTTATATATAAAATACTAAATAATTAATATAATATAATAAAAAAACTAAATTAAATTTATTTCTTAAG

AAACTAGATATCTTAAAAAACGTATAACGTTTCATTTCTAATATAATATTTTAAAAATTTATGCCACAATTAAATTTATATTATATTAGCTCTTTATAAT

TCGAGAACACCAAATAATTAAATTATTTTAATAAACCCTGATACACAAGGTACAAAAAATTAATTTTTCTTTTTAAAAAATAAATCTCTATATATTTATA

TTATCTATCTCTATACAAATTAACTATAATAAAATTTTTATATTCTAAAATATACTAATATCAAAAATATTTTTTTTATATATATATATATA----TTAT

AAATTTTTCCTTTCAAATTAAATTGATTTTCACAACTAACTTTTTAATGTAAATAAAATGCTTTATTACAAGCTCTAATTTGCCATTCCAGGTACACTTT

CCAGTACACCTACTATGTTACGACTTATCCCCCTTTAGAGAGGGAGCGACGGGCGATATGTACATATTCTAGAGCTATACTCATATAATTAAACTAAACT

ATATTACTTTCAAATCCACTTTATAAAATAATGTTAATTATTTTAACCATCTAAATAATTTTATTGTAACCCATCTCTCCTTATCTATACGCTGTATCTT

GATCTGATTTTTTTTATACTTATAAATTTTGAACATTCCAAATTCTTTAAAAACATTCAACCTACGACGATATACAAACCTTTAAAATAAGTACGATTAA

TCGTGGATCATCAATTACAGGACAGGTTCCTCTGAGTAGACTAAAATACCGCCAAATTCTTAAAATTTCAAGAACATAACTACTACTATTCAAGCATCTA

AAATTTGCATTTTTAATAATAGGGTATCTAATCCTAGTTTTTTATAAAAATCTCATAAACTCATTTTTCACATTTAAAAAATTAATTATACTTACTAATT

TCACCTAATAAATACAATATAAATTAATAATAAAATAACTTATTATATACTGAACAAATTTAATTGCATTGTTTGTGTAACCGCAACTGCTGGCACAAAC

TTGGTCAATACTATTATAAATTCCTAAATCAAAATTTCTTTTAAATTTAATCTTCACTATTGCAATTCTTTAATTAAATATATAGAATAATCTATCTTTT

TAAAATAAATTCATTAAACACTAAAATTTACATATAAAATAATTTAAAAATTAAAATCTCAAGCTAGAATAAAACTTTATTTTTTTTTTAATATACATAA

ATTATATAAAATAAAAGTACCCCCCCTACCATTTTAGTTAAGTGAATTAATTTTTAATACCTTAAATTTATTCTCAATTTATTTTTATTATATAATTTTT

TAGTTTAAAATAAAACTTTATACCTAAAATTATATACATAAATTATATTTAGAATTTATAATTTAAAGAACTATAAAAATTAAATTAAAATTTTAAATCT

ATCTAATTTACAGATAAATAAATTAAATTAAACTAACTTACCTGATAAATTTAACTATATACGCACGGGTATATTAATTATTTAATTATAATATAATACA

TTTGTTTTATTAAATAATTATTAAGCAAAATAGCATTAATTTATTATATAAAAGATCTAGATACTATTAATTTAAAGTAAATTTTTTTTTTTTTTGTAGT

ATAATTTATACTAATATACGGCATTTAATATTTTCTATATAAATGTTTATATTATTTATATTTATCTATTTATAATTCTTAATAATAATTTAGATAATTA

TTAATTTATATATTAAATATTTATATATATATATATAATAATGAGGATATATATATATATATGTATATAGATAAATTAAATTTAGTTATATATAAAAAAA

TTAATATTAATTTTACTTTTAATTTTTTCTTTAATTTAAACTATTGGATATTCAGATTCTAATGTATTATAATATATCAACCATTAATGAATAACTTTAT

ATATATAATAAAAATGTTAATTAATATATCAATTCATATGAATATATAATATTAATAAATAAATTCTTAATAAATAATAATGATAATATATATATATTGT

ATATTAATGTACTATATATATAAATTCATTAAACATTTATATTTGATACCCCCCCAAAATTTTTTCTAAAAATGTGAAGTTTCAATTTAATTAAAATTTT

CTACAATGGTCAAAAACGGCCAAAAAATGGAAAATAAAATGTGCACAGAAAATGCACATAAAATGCACATTTTTGAAAATTTCCGATTTTTGAAAAACCG

TTTGAACCAAACCTCTGATGTACCTTTTTTCGCCCCCCCTGATTACTAAAATTTATCCTCAATTACTAAGGTTTATCCGCATTTTTGCGACAGGAATTTT

GAGTATAAAATCAATTCCACACTAAATTTTTTTTT-TTTTTTTTGTGTCATTTCTCCAACATGGGCAATTTTTTTTTTAGTCGAAGGACAAATTTTAGTA

AAAAAAGTTTGAAATCAGGTTAAACCCCTATTTAACCAAAAACTATTACTTTTTAATATTGTACCCAATTTTATTTAAAATAAATTTTATTATACTTTTA

TTTATGCAAAAAGATCTTCTTATAAACTTTT

>DMR130j_JP_Fukuoka_Kyushu

AATGAAGTGTCTGACTATAGAGTTACTTTGATAGAGTAAAAAAAGTGAATTTTCACCTTCATTATAATTAACAGAATTAAACTATTTCTTTAAGCTTCAA

AAACTTATGTACATTATATACTAAATTATAAAAAGATAAGCTAATTAAGCTACTGGGCTCATACCCCATCAATAAAGGTTACAATCCTTTTCTTTTTAAT

GTATTATAAGCTTTTATTTTTTAATTCTCTTATAATTGGAACCTTAATTGCTATTTCTTCCTACTCTTGGATAGGAATATGAATAGGATTAGAAATTAAT

CTTCTCTCTATGATCCCCCTCATTAGAGACAATAAAAATATAATAGCCTCAGAAGCTGCTTTAAAATATTTCATTATTCAAACAATAGCATCAACATTAT

TATTATTCTCAATTATTATAATATCAATGAAATTTATATATCAAATAAATTTAATCACTTACTTTAATTTAATTTTTAACACTTCATTATTCATCAAAAT

AGGAGCAGCCCCATTCCATTTTTGATTCCCCGAAATAATAGAAGGATTAAATTGATTAAATGCCATTATCATACTTACTTGACAAAAACTAAGACCTATA

GTATTATTAACATATTCTAATACAACCTCCATATATCTAATTTTAACAATTATATTTAGAATAATAATCAGAGGAATTATAGGTCTAAATCAAACTAGAT

TACGAAAAATTATAGCTTATTCATCTATCAACCATATTGGATGAATAATCAGTTCAATTATATTAATTGAAATTGTTTGATTTTACTATTTTATTATTTA

CTGCATTATTACTATTAATATCAGAATTATATTTATAAAATTAAATGTTTTTCATATCAATCAATTATATATTTCAATAAATTACCATATTTTACTTAAA

TTATTCTTTGCCTTAAATTTCATGTCTTTAGGAGGATTACCTCCATTTTTAGGATTTTTCCCTAAATGACTTACAATTCAAACTTTAATCCAAAGAAATA

TGCACTCAATTGCTTTCATTATAATTTTAATAACTTTAATAACACTTTTTTTTTACCTCCGAATTACTTTTTCAATTTTATTACTAAGAAAAACAGTTTT

AACATTTTACACACAACCAAAAATTTATACTAATTACATTATAGCATTTAATTTTATTACATTATTAAGATTAATTTTCGTTACTTTGATATTCAATTTC

TTATAAATTAAACATGAAGGATTTAAGTTAAATTAAACTAAGAACCTTCAAAGTTCTAAATAAAGTAAATTCTTTAAGCCTTAGGGCTTAGCCCATCTTT

AAATTTGCAATTTAAAATTCTTTTTGAACTATAAAGCTTGATAAAAGAAACTAATTTCGTATGTAAATTTACAGTTTACCGCCTAAACCTCGGCCATTTT

ATCGAATAAATGGCTATTCTCTACAAATCACAAAGACATCGGAACTTTATACTTTCTATTTGGAAGTTGGGCAGGTATAGTAGGCACTTCATTAAGTTTA

CTGATTCGTGCTGAATTAGGAAACCCGGGATCTCTAATTGGAGATGACCAAATTTATAACGTAATCGTTACAGCTCATGCTTTCATTATAATTTTTTTTA

TAGTAATACCTATTATAATTGGAGGATTTGGAAATTGATTAGTTCCTTTAATGCTTGGAGCCCCTGACATAGCATTCCCTCGAATAAATAACATAAGATT

TTGACTTTTACCTCCTTCATTAACTCTACTTCTAATAAGAAGATTAGTCGAAAGAGGGGCAGGTACAGGCTGAACAGTTTATCCCCCACTATCAGCCAAT

ATTGCCCATAGAGGAGCATCAGTTGATTTAGCAATTTTTAGACTTCATCTTGCAGGAATTAGTTCAATTTTAGGCGCAGTAAATTTCATTACCACTGTAA

TTAACATGCGATCAACAGGAATAACTTTTGATCGAATACCCTTATTTGTTTGATCTGTAGTTTTAACAGCACTTCTTCTGCTATTATCTCTCCCCGTTTT

AGCAGGAGCAATTACTATACTATTAACAGATCGAAATATTAATACAACATTTTTTGATCCCGCAGGTGGAGGAGACCCTATTCTCTACCAACATTTATTT

TGATTTTTTGGTCATCCAGAAGTTTACATTTTAATTCTACCTGGATTTGGTATAATTTCCCATATTATTAGCCAAGAAAGAAGAAAAAAAGAAACATTTG

GAACTTTAGGTATAATTTATGCTATAATAGCTATTGGTTTATTAGGATTTATTGTTTGAGCTCATCATATATTTACAGTAGGTATAGATGTAGACACACG

GGCATATTTTACATCAGCAACAATAATCATTGCTGTTCCTACAGGAATTAAAATTTTTAGATGATTAGCTACTCTCCATGGATCACAATTAAACTACTCT

CCGTCTCTTTTATGAGCATTAGGATTTGTATTCCTATTTACAGTAGGAGGATTAACAGGAGTCATTCTAGCTAATTCATCAATTGACATTATTTTACATG

ATACTTACTATGTAGTTGCGCATTTCCATTATGTCCTTTCCATAGGAGCTGTATTTGCTATTATAGCAGGTTTTGTTCATTGATTCCCTTTATTTACAGG

TTTAACAATAAATTCAAAATTTCTTAAAATTCAATTTTTAACAATATTTATTGGTGTTAATATAACATTCTTCCCCCAACATTTCTTAGGATTAAGAGGA

ATACCCCGACGTTATTCAGATTATCCAGATGCTTATACAACTTGAAATATTATCTCATCTATTGGATCTTTAATTTCTTTAATTAGTATTTTTATCTTTT

TATTCACTATTTGAGAAAGGCTAATTTCATTGCGAAAAAGAATTAGATCTTTAAGAATATCTACATCAATTGAATGACTTCAACAAATACCCCCTTCAGA

ACATAGTTATTCTGAACTTCCAATGCTTACTAACTTCTAATATGGCAGATTAGTGCAATGGATTTAAACCCCAAATATAAAGATTAAACTTTTTTTAGAA

ATAGCTACTTGAAATACCATTTTACTTCAAGATAGGGCATCCCCATTAATAGAACAACTCTCATTCTTTCATAATCATGCTCTTCTAATTCTCTTAATAA

TTACCGTTTTAGTAGGTTATTTAATAGGAACCTTATTTTTTAACCAATTTAATTACCGATTTTTATTAGATGGTCAAACTATTGAAATTATTTGAACTAT

TTTACCTGCTGTAACACTAATTTTTATCGCATTACCATCTTTACGCTTACTTTATCTTCTAGATGAAGTTAATAACCCTTTAGTAACTATCAAAACAATT

GGGCATCAATGATATTGATCATATGAATACAGAGATTTTATAAATTTTGAATTCGATTCCTATATAATTCCTTTGACAGAAATAAAACCTCAAAATTTTC

GTTTATTAGATGTTGATAACCGAGTAATTGTCCCCTTTAACTCCCAAATTCGAATGATAGTAACAGCTGCCGATGTTATTCATTCATGAACTATCCCAGC

TTTCAGTGTAAAAATTGATGCAACACCAGGCCGACTAAATCAAATTAGATTCCTAATTAATCGAACAGGATTATTTTATGGTCAATGCTCAGAAATTTGC

GGAGCAAATCATAGATTTATACCTATTACTGTAGAAAGAATTTCACCTTCATTTTTTACTAAATGAATCTCAAAAATAAATAACCTATCATTAGATGACT

GAAAGTAAGTAATGGTCTCTTAAACCAATTAATAGTAGTTTAACATCTACTTCTGATGGCCAAAAATTTAGTTAAGATATAACATTAGTTTGTCATACTA

AAATAATCATAATTTGATAATTTTTAATTCCACAAATAGCACCTTTAAACTGACTGTCTTTATTTTTTTTAATTATTATTATTTTTTTACTTTTTAATGT

ATTAAATTACTTTAGATTCTTACAACCCTTAAAAACCCAATCTCATAGCCCTACAATTAAAAAAATTAATTGAAAATGATAACTAATTTATTTTCATCTT

TTGATCCTAGAACTTCTTTTAATTTAAGATTAAATTGATTAAGAATACTATTAGGGCTAATATTTATCCCCCCTATATTTTGATTAGTTCCTTCACGCCA

TAATTTTCTATGAATTAAAATTATTTTGACATTACACCAAGAATTTAAGGTTTTAATTGGTAATAATAATATTAAAGGAAGAACCTTAATATTTATTTCA

TTATTTTCTATAATTGTTTTCAATAACTTTCTAGGGTTATTTCCGTATATTTTTACAGGAACAAGACATTTAATTATAACATTATCTCTTGCCTTACCTT

TATGGATTAGATTCATATTATACGGGTGAATTAATAACACTATCCATATACTTGCACATTTAGTTCCTCAAGGAACACCTCCAGCTCTTATGGCATTCAT

AGTAGTAATTGAATCAATTAGAAATATTATTCGTCCTGGTACTTTAGCGGTTCGATTAGCTGCTAATATAATTGCTGGACATTTACTAATAACTTTACTA

GGAAACACAGGATTAAATTTATCATTTTTTATACTAAGTATTCTTATTATCACACAAATTCTTTTATTAATTTTAGAATCTGCTGTTGCTATTATCCAAT

CTTATGTATTTGCTGTATTAAGAACTTTATACTCTAGAGAAATTAATTAATGTCAAGACATAAAAATCACCCCTATCATTTAGTTGATGCAAGACCTTGA

CCTATTTTAGGTGCTTTTAGAGCTATAATCACAATAATTGGAATTATCAAATGATTCCATTTTTATAATAATTCTTTATTTTACTTAGGGACATTAATCA

CAATTTTAATCATAATTCAATGATGACGGGATATCACTCGTGAAGGAACTTTTCAAGGACTTCATACTTATGCTGTAACTATAGGTTTACGTTGAGGAAT

AATTTTATTTATTACATCAGAAGTATTTTTCTTTATTTCTTTTTTTTGAGCTTTTTTTCATAGTAGTTTAACCCCCGCTATTGAACTAGGAATACTATGA

CCACCTAAAGGAATTACTCCATTTAACCCTATTCAAATTCCGTTATTAAATACGTTAATTCTTTTAACTTCAGGATTAACTGTAACTTGAGCTCATCATA

GATTAATAGAAAATGACTATAACCAAACAATACAAGGTCTTGATTTAACAGTTTTATTAGGAGTATATTTTACTTTGTTACAAGGTTACGAATATTTAGA

AGCCCCCTTTACTATAGCAGATTCTGTTTATGGATCAACATTTTTTATTGCTACTGGTTTCCATGGATTACATGTTATTATTGGTACAACCTTCCTAGCT

GTTTGTTTAATACGACATTTTAATAACCATTTTACTTGTATCCATCACTTTGGGTTTGAAGCTGCTGCTTGATACTGACATTTTGTTGATGTAGTATGAC

TATTTCTTTATATTTCTATTTACTGATGAGGTAGATATTTATATAGTATAATAATTATAATTGATTTCCAATCAAAAGATCTAAAAAAA-TTAGTATAAA

TAATCATTATAATTTGAAATATAAGGTTAATTATTTTTTCTATCTCTTTTATCTTAATTATATTATCTTTTATAATCTCTAAAAAAAGATTTATAGACCG

AGAAAAAGCTTCTCCATTCGAGTGTGGATTTGACCCCAAAAGATCGGCTCGTTTACCTTTTTCTTTACATTTTTTTTTAATTGCAGTAATTTTCTTAATT

TTTGATGTTGAAATTACTCTTCTTATCCCTTTAATTCTAACAATAAAAATTACTAATATTACTATATATACTTATATTGCTCTTTTCTTTTTAATGATTC

TTTTAATAGGACTTTATCATGAATGAAACCAAGGAGCCTTAAACTGAGCTCTTTAGGGTAATAGTTAAGTATAACATTTAAGTTGCATTTAAAAAGTATT

GATTTTTCAATTTACCTTAAATAAGAAACAATTAATTGTATTTAGTTTCGACCTAAAATTCAGGTGTATACACACCCTTATTTAAATTAATTGAAACCAA

AAAGAGGTATATCACTGTTAATGATATTAATGAGAAAAGCTCCAATTAAGGAAATAAGATATTCAAGAGTAAGCTTCTAACTTAACTCTTTAGCAGTGAA

AGTCTGTTAATATTTCTATTTATATAGTTTAATAAAACATTATTTTTTCATAATAAAATTAGAATAAATTTATTCTTATAAATATTTAAAAGTAAATTTT

ACTTCCCTGATAACTTCACTATCATACTCTACATAAGCTATTTAAATTAAATATATAAAATTATAAAAATTACCCATATTATAATTAAAAGTATAAAAAT

CTTATAATTGTTATTAAATATAAACTGTAAAAATATAGAAGTATTACTAATTTTACTATATAAATTTTGTCTTCCATAATACTCTGATCAACCTTGATCA

ATAGTTTTATATAACTTACTACCTAATTTAATTGGATAATAATTTAAACCAAATGTTGAAATATAAGGTATATTCCACATAGAAGAAAAAAAAAGTCTTG

AATTTAACAAATAAAAAGATTTTAATCTATCATTTAAAGTAAACTTAGAAAGTTCAAACCCAAATCAAGCCCCAAAAAATGATACAATTAAAGCTATAAT

TTTTATTATAAAAGGTAAACAAATAAAATAAGGTGTTGGGAATATCAACCATATTAATATTCTCCCCCCAACAATAACTAAAAAAATTAAACCCGATATC

CCTTGAAGTATAATTTTTCTATTATCATTAATTTTACTTAAAGAATAAAAAGAAAAATTTCCTACTAAAACATAATAAATTAAACGAAATGTATAACAAA

CCGTTAATCCTGTTGAAAAAAAGAAAATAACATAAATATAGATATTTAAATATCTTATAGATAAAACCTCTAAAATTAAATCCTTTGAATAAAACCCTGA

TAAAAAAGGTAAACCACATAATGCTAAATTTGAAATTATAAAATAAGTACAAGTTAAAGGTATAACTTTAATTAACCCCCCTATATACCGAATATCTTGA

CAATTTCTTAATCTGTGGATTATGCACCCTGCGCATATAAATAATAAAGCCTTAAATAAAGCGTGAGTTAATAGATGAAAAAAAGCTAATTGATACTCCC

CTAAAGCTAAAATACTAATTATCAAACCCAATTGTCTTAAAGTAGACAGAGCAATAATTTTTTTTAAATCAAACTCAAAGTTTGCACCTATTCCTGCTAT

AAATATAGTTATTGTTCCAATAAATAATAAAATTAATATTAAATTACTTGTTAATGCAAAATTAAAACGAATTAATAAGTAAACACCTGCTGTAACTAAA

GTAGAAGAATGAACTAAAGAAGAAACAGGTGTTGGAGCAGCTATTGCCGCAGGCAGCCAAGAAGAAAATGGAATTTGAGCTCTTTTAGTTATAGCTGCTA

ATATAATTAATAATATAATAATATATATCTCTATACTATTTTTATATATATCAATATAAAAAATATAGTTAAATCCCCCGAAATTTACTATTCATGCAAT

TGCTATTAATAAAGCAACATCTCCAATACGGTTAGTTAAAGCTGTAATTATCCCTGCATTATAAGATTTAATATTTTGATAATAAATAACTAAACAATAA

GAAACTAATCCTAATCCATCTCATCCTAATAAAATTCTAATCAAATTGGGAGAAATAATTAATAACATTATAGATAAAACAAATATAGAAACTAATATAA

TAAATCGATGCAAATAAATATCTCCTTCTATATACTCTTCACTATAGTAAATTACTATAGAAGAAATAAATAAAACAAAACTTATAAATAATAATGACAT

CCAGTCAAGTAAAATAGTTATAATAATTCTACAAGAATTAATCCTTAATATTTCATATTCTAATATTAGTCTATAATCTAAAATTATGAAATTTAAGCTT

AATAAAAATCTTAATACTCTAAAAAATAAAAACGTTACAAAATAAATTAAACAAATAGAAATAATTTAAAGTAAACTTTACATCTTTGATACCACAAATC

AATATTTTTTATTAAACTATTTAAATTACAATCATAAAACTAAAAATTCTCTCTTTAAAATTAAGATATTTAAAGGTAACCAATGAAGTAATAATAATAA

ATATTCACGAACAAATCCTCTTGAAAAAGAGTATAAATTTCTAACTAACTTACCATGTTGACTGTAAGAATATAAATATAAAGAATACGCAGCTCTAAAA

AAAGATATTAAAGATAAAAAAACCATTGTTCAACTTCTTCATCTAACCAATCTATTAATCAAAATAATCTCACCCAGTAAGTTTAATGAAGGAGGGGCCG

CTATATTACAACAACTAAACAAAAATCATCATATTCTTATTATTGGTATTAAATTAATTAACCCCTTATTTAAATAAATACTTCGACTATTTAGACGTTC

ATAAGAAATATTTGCTAAACAAAATAATCCTGAAGAACATAAACCATGGGCAATTATTATAACTAAAGCCCCTCTTATACCCCAATAACTTAAAGTTAAA

ATCCCTCTTAATACTAAACCTATATGTGCTACAGAAGAATAAGCAATTAAAGCTTTAATATCAACTTGACGTAAACATATTAATGAAACAAAAAATCCCC

CTACTATTCTAATTGTAATAAAAATATAGTTTACTTGTAAACCTACAGTTAAAAAAATATTTATTAAACGTATTAATCCATACCCCCCTAATTTTAATAT

AACTCCAGCTAAAATTATAGAGCCAGCAACTGGAGCTTCAACATGAGCTTTAGGGAGTCAAAGATGAACAAAAAATATAGGCATTTTAATAAAAAACACT

ATATTTATACATATAAATAACAAGAATCTTTTTACATCATAACATATAAAGAAAAAATCTAAAGAATGAAATTTTTCATAATAATAAAAAATCCTAATTA

TTATGGGTAATGAAGCAAATAATGTATAAAATAACAAGTAAACACCTGCCTGCAAACGCTCAGGTTGATATCCTCAACCAATAATTAATAGGAGAGTAGG

AATTAAGCTAAACTCAAAAAACAAATAAAAAATAAATAAATTTAAAGAACTAAATGTTATAACTAAAGACAATAATAAAATAATTATTACTAATAAAAAT

AAATTATAGAAATAGTTTTTTCTATAAATACTTTCAGAAGCTAATAATATCAAAGAACAAATCCACAAACTTAATAAAATTATTATAAAAGATAAAAGAT

CATACCCTATAAAATAAGAAATATTTATATACAAATAATTAAAACTAAATCTTAACCCAAATATAAATGTAATAAAAAAATATATATATTGATTAAATCA

GTATCTCTTTTTAATACAACTTAAAGGTACCAATATTAATATTATAAAAATAAACTTTATCATAATACATTAAAAGTTTGAAAATAATCATTTCCATGCG

TTCGTATTATAGAAACTAATAATGATAAACCTAAAGCTCCTTCACAAACTCTTATAGTTAAAAATACTATACCAAAATAAAATTCAAAATTAAAGTATAT

TAAAAATAGATATAAATTAAAGTATAATCCTAAAATAATATACTCTAACCTTAATAATATTAAAAGTAAATGTTTACGTTTAATACAAAAAGAAACTAAT

CCTGTAAAATATATAATCACTGAAAATAACATACAAAAAATTAACATTAGTTTTAATAATTTAATAAAAATACTGGTCTTGTAAATCAGAAATAAGAATT

TTCTTTTAAAACTTCAGAGAAAGAGTAAACCTCTGTCATTAATCTCCAAAATTAATATTTTAAATAAACTATTCTCTGTATAATCTTATTACTAATAACA

TTATCTTTTATTAGTTCAATAACTTTTATATTTTTAAGTCACCCTTTATCTATAGGGCTTATTTTATTAATACAAACTATTATTATAGCTTTAACTATAG

GTTTTTTTAATATTAATTTCTGATATTCTTATATTTTATTTCTCATTATAATTGGAGGAATATTAGTTTTATTTATTTATATAACAAGAGTAGCTTCAAA

TGAAAAATTTTCATTTTCTATTAAAATTACATTAATAATTGGAATTATAACTTTAGGATTTTTATTTAGTATTGCTATAATAGACCCCTACTTTTCAGAC

ATTAACTCAATTTATACAGAAAACTTAGATAATTATAAAGAATATAATATATCATTTAGAAAATATTTAAGTTATCCTAATATTATCATTATATACATAA

TAATTATTTATTTATTAATTACATTAATTGCAGTTGTAAAAATTACTCAAATTGAAAAAGGACCTTTACGTCAAACTAACTAATGAAAACACCATTACGA

AAAGCTTCCCCCCTATTAAAAATTATTAATAATAGAATTATTGATTTACCTACACCATCTAATATTTCTGCTTGATGAAATTTTGGGTCCTTATTAGGAC

TTTGTCTTTTTATTCAAATTATCACTGGAATTTTCTTAGCTATACATTTTACGGCCCATATTGACATAGCATTTAATAGAGTAATTCATATTTGTCGAGA

TGTAAATTATGGCTGATTATTACGAACAATTCATGCTAACGGAGCTTCCTTTTTCTTTATTTGTATTTATCTACATATTGGCCGAGGAATATATTATAGA

AGCTATAATTTACATTTAACATGAACTATTGGAGTAATCATCCTTTTTATAGTAATAGCAACAGCATTCTTAGGATATGTATTACCTTGAGGGCAAATAT

CTTTTTGAGGAGCTACTGTTATTACCAATCTTCTCTCTGCTATTCCTTATCTAGGAAATATGATTGTCCAATGATTATGAGGAGGATTTGCAGTAGATAA

CGCCACTTTAACTCGATTTTTTACATTACATTTTATTCTTCCTTTTATTATTTTAGCTTTAATAATTATTCATTTATTATTTCTTCATCAAACTGGTTCT

AATAATCCTTTAGGATTAAATAGAAATATCGATAAAGTACCTTTCCATCCGTATTTTACTTACAAAGATACTTTTGGATTCATTATAATAACTATATTAT

TAATATTTTTAGTTTTAATTAATCCCTATCTTTTGGGGGACCCAGAAAACTTTACACCTGCTAACCCTTTAGTAACCCCAGTTCATATCCAACCAGAATG

GTACTTTTTATTCGCATACGCTATTTTACGATCAATTCCTAATAAATTAGGTGGAGTAATTGCTTTAGTTATATCAATTGCTATTTTATTAATTATACCT

TTAATTAACAAAAAAAAATTTAGTAGAACTCAATTTTATCCATTAAATAAAATTTTATTTTGATTTTTTGTTTCCATTGTAATCTTATTGACATGAATCG

GAGCCCGCCCAGTGGAAGATCCATACATTTTAACAGGTCAAATTTTAACAATTATATATTTTAGTTATTATTTCTTAAATCCCTTAATTCATAAAATATG

AGATTATATTATTTTTAAAACTTAGTTAATGAACTTGTTAAAGTGTATATTTTGAAAATATAAAAAAGAGTTTATTCTCTATTAACTTTACTAAATTTTA

TTCACTAAATAAAATAAGAAAAGATAACCAACTTTAATCCTAAAAAGAAAAATAAAAAATTTAAAGAAACTGGTAAATAACTTTTTCAACATATATATAT

TAACTTATCGTAACGATACCGAGGTAAAGTACCTCGAACTCAAATCCAAATAAATGCTATAAAAGTTAACTTCAAAAAAAAAAATAAAGAAAAAATATCC

CCCCCTATAAATAACAACACACATAATATACTTATAAATAAAATTCTAGCATACTCAGCCAAAAAGATTAAAGCGAAACCCCCTCTTCTATACTCTACAT

TAAACCCGGAAACTAACTCTGATTCTCCTTCTGCGAAATCAAAAGGAGTTCGATTAGTCTCAGCTAAACTTGAAGATAATCATATTATTCTTAAAGGTAA

ACATAGAAAAAAAAATCATACTATTTCTTGATATTTTATAAAATCTATTATATTTAAATTTAAAATTAACAATAAAAAAGATAATAAAATTAATGATAAA

CTTACTTCGTATGAAATTGTTTGAGCTACTGATCGAATACCCCCTAATAAAGCATAATTAGAATTTGATGATCAACCAGAAATTATAATAGTATAGACTC

TTAACCTAGAACAACATAAAAAATATAAAATACCTAAATTAAACCTAAATATAAATGTCAAAAAAGGCATACATATCCATAATAAAAGAGCTAAAAATAA

ATTAAAAACTGGAGATATATAATAAATAAAAAAATTTGATATTAAAGGATAAGTTTGTTCTTTAGAAAATAACTTAATAGCATCACTAAAAGGTTGGGGA

ATACCTATAAATCCAACTTTATTGGGCCCTTTACGAATTTGAATATACCCTAAAACTTTACGCTCTATTAATGTTAAAAAAGCTACACCAATTAAAACAC

AAATAATTAAAATTAAACTTGTAAATAATAAAAGAAATAAATCTTGTAATATAATGTATTACTTGTGTTAAACACATATTTAAATTCTAAATTTAAAGCA

CTAATCTGCCAAAGTAATATTCATATTCAAATTATATTAAATTTTAAAGGTATCTGATCCTTTCGTACTAAAATACCTATATTTTTTAAAGATAGAAACC

AACCTGGCTCACGCCGGTTTAAACTCAGATCATGTAAAATTTTAAAGGTCGAACAGACCTAACCTTTTAGCCCCTACACCAAAAGTTAATTTTAATCCAA

CATCGAGGTCGCAAACTTTTTTTTCGATAAGAACTCTAAAAAAAAATTACGCTGTTATCCCTAAGGTAATTTAATCTTATAATCATTAAAAATGGATCAT

TCAATCATAAATTAATGTTTTTAAATAAAAAAAGTTTAATCAATTTTTCTGCTGCCCCAGCAAAATAGTTTAAATTATTAAATATATAAATATGCTAAAA

TTAAATAATAATTTAAACTATAAAACTCTATAGGGTCTTCTCGTCTTTTAAAATTATATAAGCTTTTTTACTTATAAATAAAATTCTATATTCAATTAAA

TTGAGACAGTTACTTTCTCGTCCAACCGTTCATTCCAGCTTTCAATTAAAAAACTAATGATTATGCTACCTTTGCACGGTCAAATTACCGCGGCCATTCA

AATCCTCATTGGGCAGGTCAGACTTTAAATTATAATCAAAAAGACATGTTTTTAATAAACAGGCGAAAAGTATATTTGCCGAGTTCCTTAATTTAACCTT

GAAGTTTTAATTTAATTACTAAATTAAAATATATACTAATTTTATCATTATTCTATGTAAACCAATATTACATACATTATCTTAATAAACTACTTAAAAA

TAATATAAATCTTATTCTAACAAAAATTATTTATAACAAACTAAAGATTAACACTTCCAATTCTACTAATTTTTATTCAAAATATACATTTTTAACATTT

TATTTTAAAGCTTATCCCCTAAAATATTACTTTTTATATATAAAATACTAAATAATTAATATAATATAATAAAAAAACTAAATTAAATTTATTTCTTAAG

AAACTAGATATCTTAAAAAACGTATAACGTTTCATTTCTAATATAATATTTTAAAAATTTATGCCACAATTAAATTTATATTATATTAGCTCTTTATAAT

TCGAGAACACCAAATAATTAAATTATTTTAATAAACCCTGATACACAAGGTACAAAAAATTAATTTTTCTTTTTAAAAAATAAATCTCTATATATTTATA

TTATCTATCTCTATACAAATTAACTATAATAAAATTTTTATATTCTAAAATATACTAATATCAAAAATATTTTTTTTATATATATATATATATA--TTAT

AAATTTTTCCTTTCAAATTAAATTGATTTTCACAACTAACTTTTTAATGTAAATAAAATGCTTTATTACAAGCTCTAATTTGCCATTCCAGGTACACTTT

CCAGTACACCTACTATGTTACGACTTATCCCCCTTTAGAGAGGGAGCGACGGGCGATATGTACATATTCTAGAGCTATACTCATATAATTAAACTAAACT

ATATTACTTTCAAATCCACTTTATAAAATAATGTTAATTATTTTAACCATCTAAATAATTTTATTGTAACCCATCTCTCCTTATCTATACGCTGTATCTT

GATCTGATTTTTTTTATACTTATAAATTTTGAACATTCCAAATTCTTTAAAAACATTCAACCTACGACGATATACAAACCTTTAAAATAAGTACGATTAA

TCGTGGATCATCAATTACAGGACAGGTTCCTCTGAGTAGACTAAAATACCGCCAAATTCTTAAAATTTCAAGAACATAACTACTACTATTCAAGCATCTA

AAATTTGCATTTTTAATAATAGGGTATCTAATCCTAGTTTTTTATAAAAATCTCATAAACTCATTTTTCACATTTAAAAAATTAATTATACTTACTAATT

TCACCTAATAAATACAATATAAATTAATAATAAAATAACTTATTATATACTGAACAAATTTAATTGCATTGTTTGTGTAACCGCAACTGCTGGCACAAAC

TTGGTCAATACTATTATAAATTCCTAAATCAAAATTTCTTTTAAATTTAATCTTCACTATTGCAATTCTTTAATTAAATATATAGAATAATCTATCTTTT

TAAAATAAATTCATTAAACACTAAAATTTACATATAAAATAATTTAAAAATTAAAATCTCAAGCTAGAATAAAACTTTATTTTTTTTTTAATATACATAA

ATTATATAAAATAAAAGTACCCCCCCTACCATTTTAGTTAAGTGAATTAATTTTTAATACCTTAAATTTATTCTCAATTTATTTTTATTATATAATTTTT

TAGTTTAAAATAAAACTTTATACCTAAAATTATATACATAAATTATATTTAGAATTTATAATTTAAAGAACTATAAAAATTAAATTAAAATTTTAAATCT

ATCTAATTTACAGATAAACAAATTAAATTAAACTAACTTACCTGATAAATTTAACTATATACGCACGGGTATATTAATTATTTAATTATAATATAATACA

TTTGTTTTATTAAATAATTATTAAGCAAAATAGCATTAATTTATTATATAAAAGATCTAGATACTATTAATTTAAAGTAAATTTTTTTTTTTTTTGTAGT

ATAATTTATACTAATATACGGCATTTAATATTTTCTATATAAATGTTTATATTATTTATATTTATCTATTTATAATTCTTAATAATAATTTAGATAATTA

TTAATTTATATATTAAATATTTATATATATATATATAATAATGAGGATATATATATATATATGTATATAGATAAATTAAATTTAGTTATATATAAAAAAA

TTAATATTAATTTTACTTTTAATTTTTTCTTTAATTTAAACTATTGGATATTCAGATTCTAATGTATTATAATATATCAACCATTAATGAATAACTTTAT

ATATATAATAAAAATGTTAATTAATATATCAATTCATATGAATATATAATATTAATAAATAAATTCTTAATAAATAATAATGATAATATATATATATTGT

ATATTAATGTACTATATATATAAATTCATTAAACATTTATATTTGATACCCCCCCAAAATTTTTTCTAAAAATGTGAAGTTTCAATTTAATTAAAATTTT

CTACAATGGTCAAAAACGGCCAAAAAATGGAAAATAAAATGTGCACAGAAAATGCACATAAAATGCACATTTTTGAAAATTTCCGATTTTTGAAAAACCG

TTTGAACCAAACCTCTGATGTACCTTTTTTCGCCCCCCCTGATTACTAAAATTTATCCTCAATTACTAAGGTTTATCCGCATTTTTGCGACAGGAATTTT

GAGTATAAAATCAATTCCACACTAAATTTTTTTTT-TTTTTTTTGTGTCATTTCTCCAACATGGGCAATTTTTTTTTTAGTCGAAGGACAAATTTTAGTA

AAAAAAGTTTGAAATCAGGTTAAACCCCTATTTAACCAAAAACTATTACTTTTTAATATTGTACCCAATTTTATTTAAAATAAATTTTATTATACTTTTA

TTTATGCAAAAAGATCTTCTTATAAACTTTT

>DMR133j_JP_Mori_Honshu

AATGAAGTGTCTGACTATAGAGTTACTTTGATAGAGTAAAAAAAGTGAATTTTCACCTTCATTATAATTAACAGAATTAAACTATTTCTTTAAGCTTCAA

AAACTTATGTACATTATATACTAAATTATAAAAAGATAAGCTAATTAAGCTACTGGGCTCATACCCCATCAATAAAGGTTACAATCCTTTTCTTTTTAAT

GTATTATAAGCTTTTATTTTTTAATTCTCTTATAATTGGAACCTTAATTGCTATTTCTTCCTACTCTTGAATAGGAATATGAATAGGATTAGAAATTAAT

CTTCTCTCTATGATCCCCCTCATTAGAGACAACAAAAATATAATAGCCTCAGAAGCTGCTTTAAAATATTTCATTATTCAAACAATAGCATCAACATTGT

TATTATTCTCAATTATTATAATATCAATGAAATTTATATATCAAATAAATTTAATCACTTACTTTAATTTAATTTTTAACACTTCATTGTTCATCAAAAT

AGGAGCAGCCCCATTCCATTTTTGATTCCCCGAAATAATAGAAGGATTAAATTGATTAAATGCCATTATCATACTTACTTGACAAAAACTAAGGCCCATA

GTATTACTAACGTATTCTAATACAACCTCCATATATCTAATTTTAACAATTATATTTAGAATAATAATCAGAGGAATTATAGGTCTAAATCAAACTAGAT

TACGGAAAATTATAGCTTATTCATCTATCAACCATATTGGATGAATAATCAGTTCAATTATATTAATTGAAATTGTTTGATTTTACTATTTTATTATTTA

TTGCATTATTACTATTAATATCAGAATTATATTTATAAAATTAAATGTTTTTCATATCAATCAATTATATATTTCAATAAATTACCATATTTTACTTAAA

TTATTCTTTGCCTTAAATTTCATGTCTTTAGGAGGATTACCCCCATTTTTAGGGTTTTTCCCTAAATGACTTACAATTCAAACTTTAATCCAAAGAAATA

TATACTCAATTGCTTTCATTATAATTTTAATAACTTTAATAACACTTTTTTTTTACCTCCGAATTACTTTTTCAATTTTACTACTAAGAAAAACAGTTTT

AACATTTTACACACAACCAAAAATTTATACTAATTACATTATAGCATTTAATTTTATTGCATTATTAAGATTAATTTTCGTTACTTTGATATTCAATTTC

TTATAAATTAAACCTGAAGGATTTAAGTTAAATTAAACTAAGAACCTTCAAAGTTCTAAATAAAGTAAATTCTTTAAGCCTTAGGGCTTAGCCCATCTTT

AAATTTGCAATTTAAAATTCTTTTTGAACTATAAAGCTTGATAAAAGAAACTAATTTCGTATGTAAATTTACAGTTTACCGCCTAAACCTCGGCCATTTT

ATCGAATAAATGGCTATTCTCTACAAATCACAAAGATATCGGAACTTTATACTTTCTATTTGGAAGTTGGGCAGGCATAGTAGGCACTTCCTTAAGTTTA

CTGATTCGTGCTGAATTAGGAAACCCAGGATCTCTAATTGGAGATGATCAAATTTATAACGTAATCGTAACAGCTCATGCTTTCATTATAATTTTTTTTA

TAGTAATACCTATTATAATTGGAGGATTCGGAAACTGATTAGTTCCTTTAATGCTTGGAGCCCCCGACATAGCATTTCCCCGAATAAATAACATAAGATT

TTGACTTTTACCTCCTTCATTAACTCTACTTCTAATAAGAAGATTAGTCGAAAGAGGGGCAGGTACAGGCTGAACAGTTTATCCCCCACTATCAGCCAAT

ATCGCCCATAGAGGAGCATCAGTTGATTTAGCAATTTTTAGACTTCATCTTGCAGGAATTAGCTCAATTTTAGGTGCAGTAAATTTTATTACTACTGTAA

TTAACATACGATCAACAGGAATAACTTTTGATCGAATACCCCTATTTGTTTGATCTGTAGTTTTAACAGCACTTCTTCTGCTATTATCCCTCCCAGTTTT

AGCAGGAGCAATTACTATACTATTAACAGATCGAAATATTAATACAACATTTTTTGATCCCGCAGGTGGAGGAGACCCCATTCTCTACCAACATTTATTT

TGATTTTTTGGTCATCCAGAAGTTTACATTTTAATTTTACCTGGATTTGGTATAATCTCCCATATTATTAGCCAAGAAAGAAGAAAAAAAGAAACATTTG

GAACTTTAGGTATAATTTACGCTATAATAGCTATTGGTTTACTAGGATTTATTGTTTGAGCTCATCATATATTTACAGTAGGTATAGATGTAGACACACG

AGCATATTTTACATCAGCAACAATAATCATTGCTGTTCCTACAGGAATTAAAATTTTTAGATGATTAGCTACTCTCCATGGATCACAATTAAACTACTCC

CCGTCTCTTTTATGGGCATTAGGATTTGTATTCCTATTTACAGTAGGAGGATTAACAGGAGTAATTCTAGCTAATTCATCAATTGACATTATTTTACATG

ATACTTACTATGTAGTTGCACATTTCCATTATGTCCTTTCCATAGGAGCTGTATTTGCTATTATAGCAGGTTTTGTTCATTGATTCCCTTTATTTACAGG

TTTAACAATAAATTCAAAATTTCTTAAAATTCAATTTTTAACAATATTTATTGGTGTTAATATAACATTCTTCCCTCAACATTTCTTAGGATTAAGAGGA

ATACCTCGACGTTATTCAGATTACCCAGATGCTTATACAACTTGAAATATTATTTCATCTATTGGATCTTTAGTTTCTTTAATTAGTATTTTTATCTTTT

TATTTACTATTTGAGAAAGGCTAATTTCATTACGAAAAAGAATTAGGTCTTTAAGAATATCTACATCAATTGAATGACTCCAACAAATACCCCCTTCAGA

ACATAGTTATTCTGAACTTCCAATGCTTACTAACTTCTAATATGGCAGATTAGTGCAATGGATTTAAACCCCAAATATAAAGATTAAACTTTTTTTAGAA

ATAGCTACTTGAAATACCATTTTACTTCAGGATAGGGCATCCCCATTAATAGAGCAACTCTCATTCTTTCATAACCATGCTCTTCTAATTCTCTTTATAA

TTACCGTTCTAGTAGGTTATTTAATAGGAACTTTATTTTTTAACCAATTTAATTACCGATTTTTATTAGATGGTCAAACTATTGAAATTATTTGAACTAT

TTTACCTGCTGTAACACTAATTTTTATCGCATTACCGTCTTTACGCTTACTTTATCTTCTAGATGAAGTTAATAACCCTTTAGTAACTATCAAAACAATT

GGGCATCAATGATATTGATCATACGAATATAGAGATTTTATAAATTTTGAATTCGATTCCTATATAATTCCTTTGACAGAAATAAAACCTCAAAATTTTC

GTTTATTAGATGTTGATAACCGAGTAATTGTCCCCTTTAACTCCCAAATCCGAATGATAGTAACAGCTGCCGATGTTATTCATTCATGAACTATCCCGGC

TTTTAGTGTAAAAATTGATGCAACACCAGGCCGACTAAATCAAATTAGATTCCTAATTAATCGAACAGGATTATTTTATGGTCAATGCTCAGAAATTTGT

GGGGCAAATCATAGATTTATACCTATTACTGTAGAAAGAATTTCACCTTCATTTTTTACTAAATGAATCTCAAAAATAAATAACCTATCATTAGATGACT

GAAAGTAAGTAATGGTCTCTTAAACCAATTAATAGTAGTTTAACATCTACTTCTGATGGCCAAAAATTTAGTTAAGATATAACATTAGTTTGTCATACTA

AAATAATCATAATTTGATAATTTTTAATTCCACAAATAGCACCTTTAAACTGACTATCTTTATTTTTTTTAATTATTATTATTTTTTTACTTTTTAATGT

ATTAAATTACTTTAGATTCTTACAGCCCTTAAAAACCCAATCTCATAACCCTACAATTAAAAAAATTAATTGAAAATGATAACTAATTTATTTTCATCTT

TTGATCCTAGAACTTCTTTTAATTTAAGATTAAACTGATTAAGAATACTATTAGGGCTAATATTTATCCCCCCAATATTTTGATTAGTTCCTTCACGCTA

TAATTTTCTATGAATTAAAATTATTTTAACATTACACCAAGAATTTAAGGTTTTAATTGGTAATAATAATATTAAAGGAAGAACCTTAATATTTATTTCA

TTATTTTCTATAATTGTTTTCAATAACTTTTTAGGATTATTTCCGTATATTTTTACAGGAACAAGACATTTAATTATAACATTATCTCTTGCCTTACCTT

TATGAATTAGATTCATATTATACGGGTGAATTAATAACACTATCCACATACTTGCTCATTTAGTCCCCCAAGGAACACCCCCAGCTCTTATGGCATTCAT

AGTAGTAATTGAATCAATTAGAAATATTATTCGTCCTGGTACTTTAGCTGTTCGATTAGCTGCTAATATAATTGCTGGACATTTACTAATAACTTTACTA

GGAAACACAGGAGTAAATTTATCAATTTTTATACTAAGTATTCTTATTATCACACAAATTCTTTTATTAATTTTAGAATCTGCTGTTGCGATCATTCAAT

CTTATGTATTTGCTGTATTAAGAACTTTATACTCTAGAGAAATTAATTAATGTCAAGACATAAAAATCACCCTTATCATTTAGTTGATGCAAGACCTTGA

CCTATTTTAGGTGCTTTTAGAGCTATAATTACAATAATTGGAATTATCAAATGATTCCATTTTTATAATAATTCTTTATTTTACTTAGGGACATTAATCA

CAATTTTAATTATAATTCAATGATGACGAGATATCACTCGTGAGGGAACTTTCCAAGGACTTCATACTTACGCTGTAACTATAGGTTTACGTTGAGGAAT

AATTTTATTTATTACATCAGAAGTATTTTTCTTTATTTCTTTTTTTTGAGCCTTTTTTCATAGTAGCTTAACACCCGCTATTGAACTTGGGATACTCTGA

CCACCCAAAGGAATTACCCCATTTAACCCTATTCAAATTCCATTATTAAACACTTTAATTCTTTTAACTTCAGGATTAACTGTAACTTGAGCTCACCATA

GATTAATAGAAAATGACTATAACCAAACAATGCAAGGTCTTGGTTTAACAGTTTTACTAGGAGTATATTTTACTTTATTACAAGGTTACGAATATTTAGA

AGCCCCCTTTACTATAGCAGATTCTGTTTATGGATCAACATTTTTTATTGCTACTGGTTTCCATGGATTACATGTTATTATTGGCACAACCTTTTTAGCT

GTTTGTTTAATACGGCATTTTAATAACCATTTTACTTGTATCCATCACTTTGGATTTGAAGCTGCTGCTTGATACTGACATTTTGTTGATGTAGTATGAC

TATTTCTTTATATTTCTATTTACTGATGAGGTAGATATTTATATAGTATAATAATTATAATTGATTTCCAATCAAAAGATCTAAAAAAA-TTAGTATAAA

TAATCATTATAATTTGAAATATAGGGTTAATTATTTTTTCTATCTCTTTTATCTTAATTATACTATCTTTTACAATCTCTAAAAAAAGATTTATAGACCG

AGAAAAAGCTTCTCCATTCGAGTGCGGATTTGACCCCAAAAGATCAGCCCGTTTACCCTTTTCTTTGCATTTTTTTTTAATTGCAGTAATTTTCTTAATT

TTTGATGTTGAAATTACTCTTCTTATCCCCTTAATTCTAACAATAAAAATTACTAATATTACTGTATATACCTATATTGCTCTTTTCTTTTTAATGATTC

TTTTAATAGGACTTTACCATGAATGAAACCAAGGGGCCTTAAATTGAGCTCTTTAGGGTAATAGTTAAGTATAACATTTAAGTTGCATTTAAAAAGTATT

GATTTTTCAATTTACCTTAAATAAGAAACAATTAATTGTATTTAGTTTCGACCTAAAATTTAGGTGTATGAACACCCTTATTTAAATTAATTGAAACCAA

AAAGAGGTATATCACTGTTAATGATACTAATGAGAAAAACTCCAATTAAGGAAATAAGATATTCAAGAGTAAGCTTCTAACTTAACTCTTTAGCAGTGAA

AGTCTGTTAATATTTCTATTTATATAGTTTAATAAAACATTATTTTTTCATAATAAAATTAGAATAAATTTATTCTTATAAATATTTAAAAGTAAATTTT

ACTTCCCTGATAACTTCACTATCATACTCTATATAAGCTATTTAAATTAAATATATAAAATTATAAAAATTACCCATATTATAATTAAAAGTATAAAAAT

CTTATAATTGTTATTAAATATAAACTGTAAAAATACAGAAGTATTACTAATTTTACTATATAAATTTTGTCTTCCATAATACTCTGATCAGCCTTGATCA

ATAGTTTTATATAACTTACTACCTAATTTAATTGGATAATAATTTAAACCAAATGTTGAAATATAGGGTATATTCCACATAGAAGAAAGAAAAAGTCTTG

AATTTAACAAATAAAAAGATTTTAATCTATCATTTAAAGTAAACTTAGAAAGTTCAAATCCAAACCAAGCCCCAAAAAATGATACAATTAAAGCTATAAT

TTTTATTGTAAAAGGTAAACAAATAAAATAAGGTGTTGGGAATATCAACCATATTAATATTCTTCCCCCAACAATAACTAAAAAAATTAAACCTGATATC

CCTTGAAGTATAATTTTTCTATTATCATTAATTTTACTTAAAGAATAAAAACAAAAATTTCCTACCAAAACATAATAAATTAAACGAAATGTATAACAAA

CAGTTAATCCTGTTGAAAAAAAGAAAATAACATAAATATAGATATTTAAGTATCTTATAGATAAAACCTCTAAAATTAAATCCTTTGAATAAAACCCCGA

TAAAAAGGGTAAACCACATAATGCTAAATTTGAAATTATAAAATAAGTACAAGTTAAAGGTATAACTTTAATTAACCCCCCTATATACCGAATATCTTGA

CAATTTCTTAATCTGTGAATTATACACCCTGCGCATATAAATAATAAAGCCTTAAATAAAGCGTGAGTTAATAGATGAAAAAAAGCTAATTGATACTCCC

CTAAAGCTAAAATACTAATTATCAAACCCAATTGTCTTAAAGTAGACAGAGCAATAATTTTTTTTAAATCAAACTCAAAGTTTGCGCCTATTCCTGCTAT

AAATATAGTTATTGTTCCAATAAATAATAAAATTAATATTAAATTACTTGTTAATGCAAAATTAAAACGAATTAATAAGTAAACACCTGCTGTTACTAAG

GTAGAAGAATGAACTAAAGAAGAAACAGGTGTTGGAGCCGCTATTGCCGCAGGCAACCAAGAAGAAAATGGAATTTGAGCTCTTTTAGTTATAGCTGCTA

ATATAATTAATAAAATAATAATATATATTTCTATACTATTTTTATATACATCAATATAAAAAATATAGTTAAATCCCCCAAAATTTATTATTCATGCAAT

TGCTATTAATAAAGCAACATCTCCAATACGGTTAGTTAAAGCTGTAATTATCCCTGCATTATAAGATTTAATATTTTGATAATAAATAACTAAACAATAA

GAAACTAACCCTAACCCATCTCATCCTAATAAAATCCTAATTAAATTAGGAGAAATAATCAATAACATTATAGATAAAACAAATATAGAAACTAATATAA

TAAATCGATGCAAATAAATATCTCCTTCTATATACTCTTCACTATAGTAAATTACTATAGAAGAAATAAATAAAACAAAACTCATAAATAATAATGACAT

TCAGTCAAGTAAAATGGTTATAATAATTCTACAAGAATTAATCCTTAATATTTCATATTCTAATATTAGTCTATAATCTAAAATTATGAAATTTAAGCTT

AATAAAAATCTTAATACTCTAAAAAATAAAAACGTTACAAAATAAATTAAACAAATAGAAATAATTTAAAGTAAATTTTACATCTTTGATACCACAAATC

AATATTTTTTATTAAACTATTTAAATTACAATCATAAAACTAAAAATTCTCTCTTTAAAATTAAGATATTCAGGGGTAACCAATGAAGTAATAATAATAA

ATACTCACGAACAAATCCTCTTGAAAAAGAATATAAATTTCTAACTAACTTGCCGTGTTGACTATAAGAATATAAATATAAAGAATACGCAGCTCTAAAA

AAAGATATTAAAGATAAAAAAACCATTGTTCAACTCCTTCATCTAACTAATCTATTAATTAAAATAATCTCACCCAGTAAGTTTAATGAAGGAGGAGCCG

CTATATTACAACAACTAAATAAAAATCATCATATTCTTATTATTGGTATTAAATTGATTAGCCCCTTATTTAAATAAATACTTCGGCTATTTAACCGTTC

ATAGGAAATATTTGCTAAACAAAATAATCCTGAAGAACATAAACCATGGGCAATTATTATAACTAAAGCCCCTCTTATACCCCAATAACTTAAAGTTAAA

ATCCCTCTTAATACTAAACCTATATGCGCTACAGAAGAATAAGCAATTAAAGCTTTAATATCAACTTGACGTAAACATATTAATGAAACAAAAAATCCCC

CTACTATTCTAATTGTAATAAAAATATAGTTTACTTGTAAACCTACAGTTAAAAAAATATTTATTAAACGTATTAATCCATACCCCCCTAATTTTAATAT

AACTCCAGCTAAAATTATAGAGCCAGCAACTGGAGCTTCAACATGGGCTTTAGGGAGCCAAAGATGAACAAAAAATATAGGCATTTTAATAAAAAACACT

ATATTTATACATATAAATAACAAGAATCTTTTTACATCATAACATAAAAAGAAAAAATCTAAAGAATGAAATTTTTCATAATAATAAAAAATCCTAATTA

TTATAGGTAATGAAGCAAATAATGTATAAAATAATAAGTAAACACCTGCTTGCAAACGCTCAGGTTGATACCCCCAACCAATAATTAATAAAAGAGTAGG

AATTAAGCTAAACTCAAAAAACAAATAAAAAATAAATAAATTTAAAGAACTAAATGTTATAACTAAAGATAATAATAAAATAATTATTACTAATAAAAAT

AAATTATAAAAATAGTTTTTTCTATAAATACTTTCAGAAGCTAATAATATTAAAGAACAAATCCACAAACTTAATAAAATTATTATAAAAGATAAAAGGT

CATACCCTATAAAATAAGAAATATTTATATACAAATAATTAAAACTAAATCTTAACCCAAATATAAATGTAATAAAAAAATATATATATTGATTAAATCA

GTATCTCTTTTTAATACAACTTAAAGGTACCAATATTAATATTATAAAAATAAACTTTATCATAATACATTAAAAGTTTGAAAATAATCATTTCCATGTG

TTCGTATTATAGAAACTAATAACGATAAGCCTAAAGCTCCTTCACAAACTCTTATAGTTAAAAATACTATACCAAAATAAAATTCAAAATTAAAATATAT

TAAAAATAGATATAAATTAAAGTATAATCCTAAAATAATATACTCTAACCTTAATAATATTAAAAGTAAATGTTTACGTTTAATACAAAAAGAAACTAAT

CCTGTAAAATATATAATCACTGAAAATAACATACAAAAAATTAACATTAGTTTTAATAATTTAATAAAAATACTGGTCTTGTAAATCAGAAATAAGAATT

TTCTTTTAAAACTTCAGAGAAAGAGTAAACCTCTATCATTAATCTCCAAAATTAATATTTTAAATAAACTATTCTCTGTATAATCTTATTACTAATAACA

TTATCTTTTATTAGTTCAATAACTTTTATATTTTTAAGTCACCCTTTATCTATAGGGCTTATTTTATTAATACAAACTATTATTATAGCTTTAACTATAG

GTTTTTTTAATATTAATTTCTGATATTCTTATATTTTATTTCTTATTATAATTGGAGGAATATTAGTTTTATTTATTTATATAACAAGAGTAGCTTCAAA

TGAAAAATTTTCATTTTCTATTAAAATTACATTAATAATTAGAATTATAACTTTAGGATTTTTATTTAGTATTGCTATAATAGACCCCTACTTTTCAGAC

ATCAACTCAATTTACACAGAAAACTTAGATAATTATAAAGAATATAATATATCATTTAGAAAATATTTAAGTTATCCTAATATTATCATTATATACATAA

TAATTATTTATTTATTAATTACATTAATTGCAGTTGTAAAAATTACTCAAATTGAAAAAGGACCTTTACGTCAAACTAACTAATGAAAACACCATTACGA

AAAGCTTCCCCCCTATTAAAAATTATTAATAATAGAATTATTGATTTACCTACACCATCTAATATTTCTGCTTGATGAAATTTCGGATCCTTATTAGGAC

TCTGTCTTTTTATTCAAATTATCACTGGAATTTTCTTAGCTATACATTTTACAGCCCATATTGATATAGCATTTAATAGAGTAATTCATATTTGTCGAGA

TGTAAATTATGGCTGATTATTACGAACAATTCATGCTAACGGGGCTTCTTTTTTCTTTATTTGTATTTATCTACATATTGGCCGAGGAATATATTATAGA

AGCTATAATTTACATTTAACATGAACTATTGGAGTAATCATCCTTTTTATAGTAATAGCTACAGCATTCTTAGGGTATGTATTACCTTGAGGGCAAATAT

CTTTTTGAGGGGCTACTGTTATTACCAATCTTCTCTCTGCTATTCCTTATCTAGGAAATATGATTGTCCAATGATTATGGGGGGGATTTGCAGTAGATAA

CGCCACTTTAACTCGATTTTTTACATTACATTTTATTCTTCCTTTTATTATTTTAGCTTTAATAATTATTCATTTATTATTTCTTCATCAAACTGGTTCT

AATAACCCTTTAGGATTAAATAGAAATATCGATAAAGTACCTTTCCATCCATATTTTACTTACAAAGATACTTTTGGATTCATTATAATAACTATATTAT

TAATATTTTTAGTTTTAATTAATCCCTATCTTTTAGGAGACCCAGAAAACTTTACACCCGCTAACCCTTTAGTAACCCCAGTCCATATTCAACCAGAATG

ATACTTTTTATTCGCATACGCTATTTTACGATCAATTCCTAATAAATTAGGTGGAGTAATTGCTTTAGTTATATCAATTGCTATTTTATTAATTATACCT

TTAATTAACAAAAAAAAATTTAGTAGAACTCAATTTTACCCATTAAATAAAATTTTATTTTGATCTTTTGTCTCTATTGTAATCTTATTAACATGAATCG

GAGCTCGCCCAGTGGAAGATCCATACATTTTAACAGGTCAAATTTTAACAATTATATATTTTAGTTATTATTTCTTAAACCCCTTAATTCATAAAATATG

AGATTATATTATTTTTAAAACTTAGTTAATGAACTTGTTAAAGTGTATATTTTGAAAATATAAAAAAGAGTTTATTCTCTATTAACTTTACTAAATTTTA

TTCACTAAATAAAATGAGAAAAGATAACCAACTTTAAACCTAAAAAAAAAAATAAAAAATTTAAAGAAACTGGTAAATAACTTTTTCAACATATATATAT

TAACTTATCATAACGATACCGAGGTAAAGTACCTCGAACTCAAATCCAAATAAATGCTATAAAAGTTAACTTCAAAAAAAAAAATAAAGAAAAAATATCC

CCCCCTATAAATAACAACACACATAATATACTTATAAATAAAATTCTAGCATACTCAGCCAAAAAGATTAAAGCAAATCCCCCTCTTCTATACTCTACAT

TAAACCCAGAAACTAACTCTGATTCTCCTTCTGCAAAATCAAAAGGAGTCCGATTAGTCTCAGCTAAACTTGAAGATAATCATATTATTCTTAAAGGTAA

GCACAGAAAAAAAAATCATACTATTTCTTGATATTTTATAAAATCTATTATATTTAAATTTAAAATTAACAATAAAAAAGATAATAAAATTAATGATAAA

CTTACTTCGTATGAAATTGTTTGAGCTACTGATCGAATACCCCCTAATAAAGCATAATTAGAATTTGATGATCAACCAGAAATTATGATAGTATAGACCC

TTAGCCTAGAACAACATAAAAAATATAAAATACCTAAATTAAACCTAAATATAAATGTCAAAAAAGGTATACATATCCATAATAAAAGAGCTAAAAATAA

ATTAAAAACTGGAGATATATAATAAATAAAAAAATTTGATATTAAAGGATAAGTTTGTTCTTTAGAAAATAACTTAATAGCATCACTAAAAGGTTGAGGA

ATACCTATAAATCCAACTTTATTGGGCCCTTTACGAATTTGGATATACCCTAAAACTTTGCGCTCTATTAATGTTAAAAAAGCTACACCAATTAAAACAC

AAATAATTAAAACTAAACTTGTAAATAATAAAAGAAATAAATCTTGTAATATAATGTATTACTTGTGTTAAACACATATTTAAATTCTAAATTTAAAGCA

CTAATCTGCCAAAGTAATATTCATATTCAAATTATATTAAATTTTAAAGGTATTTGATCCTTTCGTACTAAAATACCTATGTTTTTTAAAGATAGAAACC

AACCTGGCTCACGCCGGTTTAAACTCAGATCATGTAAAATTTTAAAGGTCGAACAGACCTAACCTTTTAGCCCCTACACCAAAAGTTAATTTTAATCCAA

CATCGAGGTCGCAAACTTTTTTTTCGATAAGAACTCTAAAAAAAAATTACGCTGTTATCCCTAAGGTAATTTAATCTTGTAATCATTAAAAATGGATCAT

TCAATCATAAATTAATGTTTTTAAATAAAAAAAGTTTAATCAATTTTTCTGCTGCCCCAGCAAAATAGTTTAAATTATTAAATATATAAATATACTAAAA

TTAAATAATAATTTAAACTATAAAACTCTATAGGGTCTTCTCGTCTTTTAAAATTATATAAGCTTTTTTACTTATAAATAAAATTCTATATTCAATTAAA

TTGAGACAGTTACTTTCTCGTCCAACCGTTCATTCCAGCTTTCAATTAAAAAACTAATGATTATGCTACCTTTGCACGGTCAAATTACCGCGGCCATTCA

AATCCTCATTGGGCAGGTCAGACTTTAAATTATAATCAAAAAGACATGTTTTTAATAAACAGGCGAAAAGTGTATTTGCCGAGTTCCTTAATTTAACCTT

GAAGTTTTAATTTAATTACTAAATTAAAATATATACTAATTTTATCATTATTCTATATAAACCAATATTACATATATTATCTTAATAAACTACTTAAAAA

TAATATAAATCTTATTCTAACAAAAATTATTTATAACAAACTAAAGATTAACACTTCCAATTCTACTAATTTTTATTCAAAATATACATTTTTAACATTT

TATTTTAAAGCTTATCCCCTAAAATATTACTTTTTATATATAAAATACTAAATAATTAATATAATACAATAAAAAAACTAAATTAAATTTATTTCTTAAG

AAACTAGATATCTTAAAAAACGTATAACGTTTCATTTCTAATATAATATTTTAAAAATTTATGCCACAATTAAATTTATATTATATTAGCTCTTTATAAT

TCGAGAACACTAAATAATTAAATTATTTTAATAAACCCTGATACACAAGGTACAAAAAATTAATTTTTCTTTTTAAAAAATAAATCTCTATATATTTATA

TTATCTATCTCTATACAAATTAACTATAATAAAATTTTTATATTCTAAAATATACTAATATCAAAAATATTTTTTTTATATATATATATATA----TTAT

AAATTTTTCCTTTCAAATTAAATTGATTTTCACAACTAACTTTTTAATGTAAATAAAATGCTTTATTACAAGCTCTAATTTGCCATTCCAGGTACACTTT

CCAGTACACCTACTATGTTACGACTTATCCCTCTTTAGAGAGGGAGCGACGGGCGATATGTACATATTCTAGAGCTATACTCATATAATTAAACTAAACT

ATATTACTTTCAAATCCACTTTATAAAATAATGTTAATTATTTTAACCATCTAAATAATTTTATTGTAACCCATCTCTCCTTATCTATACGCTGTATCTT

GATCTGATTTTTTTTATACTTATAAATTTTGAACATTCCAAATTCTTTAAAAACATTCAACCTACGACGATATACAAACCTTTAAAATAAGTACGATTAA

TCGTGGATCATCAATTATAGGACAGGTTCCTCTGAGTAGACTAAAATACCGCCAAATTCTTAAAATTTCAAGAACATAACTACTACTATTCAAGCATCTA

AAATTTGCATTTTTAATAATAGGGTATCTAATCCTAGTTTTTTATAAAAATCTCATAAACTCATTTTTCACATTTAAAAAATTAATTATACTTACTAATT

TCACCTAATAAATACAATATAAATTAATAATAAAATAACTTATTATATACTGAACAAATTTAATTGCATTGTTTGTGTAACCGCAACTGCTGGCACAAAC

TTGGTCAATACTATTATAAATTCCTAAATCAAAATTTCTTTTAAATTTAATCTTCACTATTGCAATTCTTTAATTAAATATACAGAATAATTTATTTTTT

TAAAATAAATTCATTAAACACTAAAATTTACATATAAAATAATTTAAAAATTAAAATCTCAAGCTAGAATAAAACTTTATTTTTTTTTCAATATACATAA

ATTATATAAAATAAAAGTACCCCCC-TACTATTTTAATTAAATGAATTAATTTTTAATGCCTTAAATTTATTTCCAATTTATTTTTATTATATAGTTTTT

TATTTAAAAATAAAACTTTATACCTAAAATTATATACATAAATTATATTTAAAATTTATAATTTAAAGAACTATAAAAATTAAATTAAAATTTAAAATCT

ATCTAATTTATAGATAAACAAATTAAATTAAACTAACTTACCTGATAAATTTAACTATATACGCGCAGGTATATTAATTATTTAATTATAATATAATACA

TTTGTTTTATTAAATAATTATTAAGCAAAATAGCATTAATTTATTATATAAAAGATCTAGATACTATTAATTTAAAGTAAATTTTTTTTTTTTTTGTAGT

ATAATTTATACTAATATACAGCATTTAATATCTTCTATATAAATGTTTATATTATTTATATTTATCTATTTATAATTCTTAATAATAATTTAGATAATTA

TTAATTTATATATTAAATATTTATATATATATATATAATAATAAGGATATATATATATATATGTATATAGATAAATTAAATTTAGTTATATATAAAAAAA

TTAATATTAATTTTACTTTTAATTTTTTCTTTAATTTAAACTATTGGATATTCAGATTCTAATATATTATAATATATCAACCATTAATGAATAACTTTAT

ATATATAATAAAAATGTTAATTAATATATCAATTCATATGAATATATAATATTAATAAATAAATTCTTAATAAATAATAGTGATAATATATATATATTGT

ATATTAATGTACTATATATATAAATTCATTAAACATTTATATTTGATACCCCCCCAAAATTTTTTCTAAAAATGTGAAGTTTCAATTTAATTAAAATTTT

CTACAATGGTCAAAAACGGCCAAAAAATGGAAAATAAAATGTGCACAGAAAATGCACATAAAATGCACATTTTTGAAAATTTCCGATTTTTGAAAAACCG

TTTGAACCAAACCTCTGATGTACCTTTTTTCGCCCCCCCTGATTACTAAAATTTATCCTCAATTACTAAGGTTTATCCGCATTTTTGCGACAGGAATTTT

GAGTATAAAATCAATTCCACACTAAATTTTTTTTT-TTTTTTTTGTGTCATTTCTCCAACATGGGCAATTTTTTTTTTAGTCGAAGGACAAATTTTAGTA

AAAAAAGTTTGAAATTAGGTTAAACCCTTGTTTAACCAAAAACTATTACTTTTCAATATTATACCCAATTTTATTTAAAATAAATTTTATTATACTTTTA

TTTATGCAAAAAGATCTTCTTATAAACTTTT

>DMR173j_JP_Tsuruoka_Honshu

AATGAAGTGTCTGACTATAGAGTTACTTTGATAGAGTAAAAAAAGTGAATTTTCACCTTCATTATAATTAACAGAATTAAACTATTTCTTTAAGCTTCAA

AAACTTATGTACATTATATACTAAATTATAAAAAGATAAGCTAATTAAGCTACTGGGCTCATACCCCATCAATAAAGGTTACAATCCTTTTCTTTTTAAT

GTATTATAAGCTTTTATTTTTTAATTCTCTTATAATTGGAACCTTAATTGCTATTTCTTCCTACTCTTGGATAGGAATATGAATAGGATTAGAAATTAAT

CTTCTCTCTATGATCCCCCTCATTAGAGACAACAAAAATATAATAGCCTCAGAAGCTGCTTTAAAATATTTCATTATTCAAACAATAGCATCAACATTAT

TATTATTCTCAATTATTATAATATCAATGAAATTTATATATCAAATAAATTTAATCACTTACTTTAATTTAATTTTTAACACTTCATTATTCATCAAAAT

AGGAGCAGCCCCATTCCATTTTTGATTCCCCGAAATAATAGAAGGATTAAATTGATTAAATGCCATTATCATACTTACTTGACAAAAACTAAGGCCCATA

GTATTACTAACATATTCTAATACAACCTCCATATATCTAATTTTAACAATTATATTTAGAATAATAATCAGAGGAATTATAGGTCTAAATCAAACTAGAT

TACGGAAAATTATAGCTTATTCATCTATCAACCATATTGGATGAATAATCAGTTCAATTATATTAATTGAAATTGTTTGATTTTACTATTTTATTATTTA

TTGCATTATTACTATTAATATCAGAATTATATTTATAAAATTAAATGTTTTTCATATCAATCAATTATATATTTCAATAAATTACCATATTTTACTTAAA

TTATTCTTTGCCTTAAATTTCATGTCTTTAGGAGGATTACCCCCATTTTTAGGGTTTTTCCCTAAATGACTTACAATTCAAACTTTAATCCAAAGAAATA

TGTACTCAATTGCTTTCATTATAATTTTAATAACTTTAATAACACTTTTTTTTTACCTCCGAATTACTTTTTCAATTTTACTACTAAGAAAAACAGTTTT

AACATTTTACACACAACCAAAAATTTATACTAATTACATTATAGCATTTAATTTTATTGCATTATTAAGATTAATTTTCGTTACTTTGATATTCAATTTC

TTATAAATTAAACCTGAAGGATTTAAGTTAAATTAAACTAAGAACCTTCAAAGTTCTAAATAAAGTAAATTCTTTAAGCCTTAGGGCTTAGCCCATCTTT

AAATTTGCAATTTAAAATTCTTTTTGAACTATAAAGCTTGATAAAAGAAACTAATTTCGTATGTAAATTTACAGTTTACCGCCTAAACCTCGGCCATTTT

ATCGAATAAATGGCTATTCTCTACAAATCACAAAGATATCGGAACTTTATACTTTCTATTTGGAAGTTGAGCAGGCATAGTAGGCACTTCCTTAAGTTTA

CTGATTCGTGCTGAATTAGGAAACCCAGGATCTCTAATTGGAGATGATCAAATTTATAACGTAATCGTAACAGCTCATGCTTTCATTATAATTTTTTTTA

TAGTAATACCTATTATAATTGGAGGATTCGGAAACTGATTAGTTCCTTTAATGCTTGGAGCCCCCGACATAGCATTCCCCCGAATAAATAACATAAGATT

TTGACTTTTACCTCCTTCATTAACTCTACTTCTAATAAGAAGATTAGTCGAAAGAGGGGCAGGTACAGGCTGAACAGTTTATCCCCCACTATCAGCCAAT

ATCGCCCATAGAGGAGCATCAGTTGATTTAGCAATTTTTAGACTTCATCTTGCAGGAATTAGCTCAATTTTAGGTGCAGTAAATTTTATTACTACTGTAA

TTAACATACGATCAACAGGAATAACTTTTGATCGAATACCCCTATTTGTTTGATCTGTAGTTTTAACAGCACTTCTTCTGCTATTATCCCTCCCAGTTTT

AGCAGGAGCAATTACTATACTATTAACAGATCGAAATATTAATACAACATTTTTTGATCCCGCAGGTGGAGGAGACCCCATTCTCTACCAACATTTATTT

TGATTTTTTGGTCATCCAGAAGTTTACATTTTAATTTTACCTGGATTTGGTATAATCTCCCATATTATTAGCCAAGAAAGAAGAAAAAAAGAAACATTTG

GAACTTTAGGTATAATTTACGCTATAATAGCTATTGGTTTACTAGGATTTATTGTTTGAGCTCATCATATATTTACAGTAGGTATAGATGTAGACACACG

AGCATATTTTACATCAGCAACAATAATCATTGCTGTTCCTACAGGAATTAAAATTTTTAGATGATTAGCTACTCTCCATGGATCACAATTAAACTACTCC

CCGTCTCTTTTATGGGCATTAGGATTTGTATTCCTATTTACAGTAGGAGGATTAACAGGAGTAATTCTAGCTAATTCATCAATTGACATTATTTTACATG

ATACTTACTATGTAGTTGCACATTTCCATTATGTCCTTTCCATAGGAGCTGTATTTGCTATTATAGCAGGTTTTGTTCATTGATTCCCTTTATTTACAGG

TTTAACAATAAATTCAAAATTTCTTAAAATTCAATTTTTAACAATATTTATTGGTGTTAATATAACATTCTTCCCTCAACATTTCTTAGGATTAAGAGGA

ATACCTCGACGTTATTCAGATTACCCAGATGCTTATACAACTTGAAATATTATCTCATCTATTGGATCTTTAGTTTCTTTAATTAGTATTTTTATCTTTT

TATTTACTATTTGAGAAAGGCTAATTTCATTGCGAAAAAGAATTAGGTCTTTAAGAATATCTACATCAATTGAATGACTCCAACAAATACCCCCTTCAGA

ACATAGTTATTCTGAACTTCCAATGCTTACTAACTTCTAATATGGCAGATTAGTGCAATGGATTTAAACCCCAAATATAAAGATTAAACTTTTTTTAGAA

ATAGCTACTTGAAATACCATTTTACTTCAGGATAGGGCATCCCCATTAATAGAGCAACTCTCATTCTTTCATAACCATGCTCTTCTAATTCTCTTTATAA

TTACCGTTCTAGTAGGTTATTTAATAGGAACTTTATTTTTTAACCAATTTAATTACCGATTTTTATTAGATGGTCAAACTATTGAAATTATTTGAACTAT

TTTACCTGCTGTAACACTAATTTTTATTGCATTACCGTCTTTACGCTTACTTTATCTTCTAGATGAAGTTAATAACCCTTTAGTAACTATCAAAACAATT

GGGCATCAATGATATTGATCATACGAATATAGAGATTTTATAAATTTTGAATTCGATTCCTATATAATTCCTTTAACAGAAATAAAACCTCAAAATTTTC

GTTTATTAGATGTTGATAACCGAGTAATTGTCCCCTTTAACTCCCAAATCCGAATGATAGTAACAGCTGCCGATGTTATTCATTCATGAACTATCCCGGC

TTTTAGTGTAAAAATTGATGCAACACCAGGCCGACTAAATCAAATTAGATTCCTAATTAATCGAACAGGATTATTTTATGGTCAATGCTCAGAAATTTGT

GGGGCAAATCATAGATTTATACCTATTACTGTAGAAAGAATTTCACCTTCATTTTTTACTAAATGAATCTCAAAAATAAATAACCTATCATTAGATGACT

GAAAGTAAGTAATGGTCTCTTAAACCAATTAATAGTAGTTTAACATCTACTTCTGATGGCCAAAAATTTAGTTAAGATATAACATTAGTTTGTCATACTA

AAATAATCATAATTTGATAATTTTTAATTCCACAAATAGCACCTTTAAACTGACTATCTTTATTTTTTTTAATTATTATTATTTTTTTACTTTTTAATGT

ATTAAATTACTTTAGATTCTTACAGCCCTTAAAAACCCAATCTCATAACCCTACAATTAAAAAAATTAATTGAAAATGATAACTAATTTATTTTCATCTT

TTGATCCTAGAACTTCTTTTAATTTAAGATTAAACTGATTAAGAATACTATTAGGGCTAATATTTATCCCCCCAATATTTTGATTAGTTCCTTCACGCCA

TAATTTTCTATGAATTAAAATTATTTTAACATTACACCAAGAATTTAAGGTTTTAATTGGTAATAATAATATTAAAGGAAGAACCTTAATATTTATTTCA

TTATTTTCTATAATTGTTTTCAATAACTTTTTAGGATTATTTCCGTATATTTTTACAGGAACAAGACATTTAATTATAACATTATCTCTTGCCTTACCTT

TATGAATTAGATTCATATTATACGGGTGAATTAATAACACTATCCACATACTTGCTCATTTAGTTCCCCAAGGAACACCCCCAGCTCTTATGGCATTCAT

AGTAGTAATTGAATCAATTAGAAATATTATTCGTCCTGGTACTTTGGCTGTTCGATTAGCTGCTAATATAATTGCTGGACATTTACTAATAACTTTACTA

GGAAACACAGGATTAAATTTATCAATTTTTATACTAAGTATTCTTATTATCACACAAATTCTTTTATTAATTTTAGAATCTGCTGTTGCGATCATTCAAT

CTTATGTATTTGCTGTATTAAGAACTTTATACTCTAGAGAAATTAATTAATGTCAAGACATAAAAATCACCCCTATCATTTAGTTGATGCAAGACCTTGA

CCTATTTTAGGTGCTTTTAGAGCTATAATTACAATAATTGGAATTATCAAATGATTCCATTTTTATAATAATTCTTTATTTTACTTAGGGACATTAATCA

CAATTTTAATTATAATTCAATGATGACGAGATATCACTCGTGAGGGAACTTTCCAAGGACTTCATACTTACGCTGTAACTATAGGTTTACGTTGAGGAAT

AATTTTATTTATTACATCAGAAGTATTTTTCTTTATTTCTTTTTTTTGAGCCTTTTTTCATAGTAGTTTAACACCCGCTATTGAACTAGGAATACTCTGA

CCACCCAAAGGAATTACCCCATTTAACCCTATTCAAATTCCATTATTAAACACTTTAATTCTTTTAACTTCAGGATTAACTGTAACTTGAGCTCACCATA

GATTAATAGAAAATGACTATAACCAAACAATGCAAGGTCTTGGTTTAACAGTTTTACTAGGAGTATATTTTACTTTATTACAAGGTTACGAATATTTAGA

AGCCCCCTTTACTATAGCAGATTCTGTTTATGGATCAACATTTTTTATTGCTACTGGTTTCCATGGATTACATGTTATTATTGGCACAACCTTTTTAGCT

GTTTGTTTAATACGGCATTTTAATAACCATTTTACTTGTATCCATCACTTTGGATTTGAAGCTGCTGCTTGATACTGACATTTTGTTGATGTAGTATGAC

TATTTCTTTATATTTCTATTTACTGATGAGGTAGATATTTATATAGTATAATAATTATAATTGATTTCCAATCAAAAGATCTAAAAAA--TTAGTATAAA

TAATCATTATAATTTGAAATATAGGGTTAATTATTTTTTCTATCTCTTTTATCTTAATTATACTATCTTTTACAATCTCTAAAAAAAGATTTATAGACCG

AGAAAAAGCTTCTCCATTCGAGTGCGGATTTGACCCCAAAAGATCAGCCCGTTTACCCTTTTCTTTGCATTTTTTTTTAATTGCAGTAATTTTCTTAATT

TTTGATGTTGAAATTACTCTTCTTATCCCTTTAATTCTAACAATAAAAATTACTAATATTACTATATATACCTATATTGCTCTTTTCTTTTTAATGATTC

TTTTAATAGGACTTTACCATGAATGAAACCAAGGGGCCTTAAATTGAGCTCTTTAGGGTAATAGTTAAGTATAACATTTAAGTTGCATTTAAAAAGTATT

GATTTTTCAATTTACCTTAAATAAGAAACAATTAATTGTATTTAGTTTCGACCTAAAATTTAGGTGTATGAACACCCTTATTTAAATTAATTGAAACCAA

AAAGAGGTATATCACTGTTAATGATACTAATGAGAAAAACTCCAATTAAGGAAATAAGATATTCAAGAGTAAGCTTCTAACTTAACTCTTTAGCAGTGAA

AGTCTGTTAATATTTCTATTTATATAGTTTAATAAAACATTATTTTTTCATAATAAAATTAGAATAAATTTATTCTTATAAATATTTAAAAGTAAATTTT

ACTTCCCTGATAACTTCACTATCATACTCTATATAAGCTATTTAAATTAAATATATAAAATTATAAAAATTACCCATATTATAATTAAAAGTATAAAAAT

CTTATAATTGTTATTAAATATAAACTGTAAAAATACAGAAGTATTACTAATTTTACTATATAAATTTTGTCTTCCATAATACTCCGATCAGCCTTGATCA

ATAGTTTTATATAACTTACTACCTAATTTAATTGGATAATAATTTAAACCAAATGTTGAAATATAGGGCATATTCCACATAGAAGAAAAAAAAAGTCTTG

AATTTAACAAATAAAAAGATTTTAATCTATCATTTAAAGTAAACTTAGAAAGTTCAAACCCAAACCAAGCCCCAAAAAATGATACAATTAAAGCTATAAT

TTTTATTGTAAAAGGTAAACAAATAAAATAAGGTGTTGGGAATATCAACCATATTAATATTCTTCCCCCAACAATAACTAAAAAAATTAAACCTGATATC

CCTTGAAGTATAATTTTTCTATTATCATTAATTTTACTTAAAGAATAAAAACAAAAATTTCCTACCAAAACATAATAAATTAAACGAAATGTATAACAAA

CAGTTAATCCTGTTGAAAAAAAGAAAATAACATAAATATAGATATTTAAGTATCTTATAGATAAAACCTCTAAAATTAAATCCTTTGAATAAAACCCCGA

TAAAAAGGGTAAACCACATAATGCTAAATTTGAAATTATAAAATAAGTACAAGTTAAAGGTATAACTTTAATTAACCCCCCTATATACCGAATATCTTGA

CAATTTCTTAATCTGTGAATTATACACCCTGCGCATATAAATAATAAAGCCTTAAATAAAGCGTGAGTTAATAGATGAAAAAAGGCTAATTGATACTCCC

CTAAAGCTAAAATACTAATTATCAAACCCAATTGTCTTAAAGTAGACAGAGCAATAATTTTTTTTAAATCAAACTCAAAGTTTGCGCCTATTCCTGCTAT

AAATATAGTTATTGTTCCAATAAATAATAAAATTAATATTAAATTACTTGTTAATGCAAAATTAAAACGAATTAATAAGTAAACACCTGCTGTTACTAAG

GTAGAAGAATGAACTAAAGAAGAAACAGGTGTTGGAGCCGCTATTGCCGCAGGCAACCAAGAAGAAAATGGAATTTGAGCTCTTTTAGTTATAGCTGCTA

ATATAATTAATAAAATAATAATATATATTTCTATACTATTTTTATATACATCAATATAAAAAATATAGTTAAATCCCCCAAAATTTATTATTCATGCAAT

TGCTATTAATAAAGCAACATCTCCAATACGGTTAGTTAAAGCTGTAATTATCCCTGCATTATAAGATTTAATATTTTGATAATAAATAACTAAACAATAA

GAAACTAACCCTAACCCATCTCATCCTAATAAAATCCTAATTAAATTAGGAGAAATAATCAATATCATTATAGATAAAACAAATATAGAAACTAATATAA

TAAATCGATGCAAATAAATATCTCCTTCTATATACTCTTCACTATAGTAAATTACTATAGAAGAAATAAATAAAACAAAACTCATAAATAATAATGACAT

CCAGTCAAGTAAAATGGTTATAATAATTCTACAAGAATTAATCCTTAATATTTCATATTCTAATATTAGTCTATAATCTAAAATTATGAAATTTAAGCTT

AATAAAAATCTTAATACTCTAAAAAATAAAAACGTTACAAAATAAATTAAACAAATAGAAATAATTTAAAGTAAATTTTACATCTTTGATACCACAAATC

AATATTTTTTATTAAACTATTTAAATTACAATCATAAAACTAAAAATTCTCTCTTTAAAATTAAGATATTCAAGGGTAACCAATGAAGTAATATTAATAA

ATACTCACGAACAAACCCTCTTGAAAAAGAATATAAATTTCTAACTAACTTGCCGTGTTGACTATAAGAATATAAATATAAAGAATACGCAGCTCTAAAA

AAAGATATTAAAGATAAAAAAACCATTGTTCAACTCCTTCATCTAACTAATCTATTAATTAAAATAATCTCACCCAGTAAGTTTAATGAAGGAGGAGCCG

CTATATTACAACAACTAAATAAAAATCATCATATTCTTATTATTGGTATTAAATTGATTAGCCCCTTATTTAAATAAATACTTCGGCTATTTAGCCGTTC

ATAAGAAATATTTGCTAAACAAAATAATCCTGAAGAACATAAACCATGAGCAATTATTATAACTAAAGCCCCTCTTATACCCCAATAACTTAAAGTTAAA

ATCCCTCTTAATACTAAACCTATATGCGCTACAGAAGAATAAGCAATTAAAGCTTTAATATCAACTTGACGTAAACATATTAATGAAACAAAAAATCCCC

CTACTATTCTAATTGTAATAAAAATATAGTTTACTTGTAAACCTACAGTTAAAAAAATATTTATTAAACGTATTAATCCATACCCCCCTAATTTTAATAT

AACTCCAGCTAAAATTATAGAGCCAGCAACTGGAGCTTCAACATGGGCTTTAGGGAGCCAAAGATGAACAAAAAATATAGGCATTTTAATAAAAAACACT

ATATTTATACATATAAATAACAAGAATCTTTTTACATCATAACATAAAAAGAAAAAATCTAAAGAATGAAATTTTTCATAATAATAAAAAATCCTAATTA

TTATAGGTAATGAAGCAAATAATGTATAAAATAACAAGTAAACACCTGCTTGCAAACGCTCAGGTTGATACCCCCAACCAATAATTAATAAAAGAGTAGG

AATTAAGCTAAACTCAAAAAACAAATAAAAAATAAATAAATTTAAAGAACTAAATGTTATAATTAAAGATAATAATAAAATAATTATTACTAATAAAAAT

AAATTATAAAAATAGTTTTTTCTATAAATACTTTCAGAAGCTAATAATATTAAAGAACAAATCCACAAACTTAATAAAATTATTATAAAAGATAAAAGGT

CATACCCTATAAAATAAGAAATATTTATATACAAATAATTAAAACTAAATCTTAACCCAAATATAAATGTAATAAAAAAATATATATATTGATTAAATCA

GTATCTCTTTTTAATACAACTTAAAGGTACCAATATTAATATTATAAAAATAAACTTTATCATAATACATTAAAAGTTTGAAAATAATCATTTCCATGTG

TTCGTATTATAGAAACTAATAACGATAAACCTAAAGCTCCTTCACAAACTCTTATAGTTAAAAATACTATACCAAAATAAAATTCAAAATTAAAATATAT

TAAAAATAGATATAAATTAAAGTATAATCCTAAAATAATATACTCTAACCTTAATAATATTAAAAGTAAATGTTTACGTTTAATACAAAAAGAAACTAAT

CCTGTAAAATATATAATCACTGAAAATAATATACAAAAAATTAACATTAGTTTTAATAATTTAATAAAAATACTGGTCTTGTAAATCAGAAATAAGGATT

TTCTTTTAAAACTTCAGAGAAAGAGTAAACCTCTATCATTAATCTCCAAAATTAATATTTTAAATAAACTATTCTCTGTATAATCTTATTACTAATAACA

TTATCTTTTATTAGTTCAATAACTTTTATATTTTTAAGTCACCCTTTATCTATAGGGCTTATTTTATTAATACAAACTATTATTATAGCTTTAACTATAG

GTTTTTTTAATATTAATTTCTGATATTCTTATATTTTATTTCTTATTATAATTGGAGGAATATTAGTTTTATTTATTTATATAACAAGAGTAGCTTCAAA

TGAAAAATTTTCATTTTCTATTAAAATTACATTAATAATTAGAATTATAACTTTAGGATTTTTATTTAGTATTGCTATAATAGACCCCTACTTTTCAGAC

ATCAACTCAATTTACACAGAAAACTTAGATAATTATAAAGAATATAATATATCATTTAGAAAATATTTAAGTTATCCTAATATTATCATTATATACATAA

TAATTATTTATTTATTAATTACATTAATTGCAGTTGTAAAAATTACTCAAATTGAAAAAGGACCTTTACGTCAAACTAACTAATGAAAACACCATTACGA

AAAACTTCCCCCCTATTAAAAATTATTAATAATAGAATTATTGATTTACCTACACCATCTAATATTTCTGCTTGATGAAATTTCGGATCCTTATTAGGAC

TCTGTCTTTTTATTCAAATTATCACTGGAATTTTCTTAGCTATACATTTTACAGCTCATATTGACATAGCATTTAATAGAGTAATTCATATTTGTCGAGA

TGTAAATTATGGCTGATTATTACGAACAATTCATGCTAACGGGGCTTCTTTTTTCTTTATTTGTATTTATCTACATATTGGCCGAGGAATATATTATAGA

AGCTATAATTTACATTTAACATGAACTATTGGAGTAATCATCCTTTTTATAGTAATAGCTACAGCATTCTTAGGGTATGTATTACCTTGAGGGCAAATAT

CTTTTTGAGGGGCTACTGTTATTACCAATCTTCTCTCTGCTATTCCTTATCTAGGAAATGTGATTGTCCAATGATTGTGGGGGGGATTTGCGGTAGATAA

CGCCACTTTAACTCGATTTTTTACATTACATTTTATTCTTCCTTTTATTATTTTAGCTTTAATAATTATTCATTTATTATTTCTTCATCAAACTGGTTCT

AATAACCCTTTAGGATTAAATAGAAATATCGATAAAGTACCTTTCCATCCATATTTTACTTACAAAGATACTTTTGGATTTATTATAATAACTATATTAT

TAATATTTTTAGTTTTAATTAATCCCTATCTTTTAGGAGACCCAGAAAACTTTACACCCGCTAACCCTTTAGTAACCCCAGTCCATATCCAACCAGAATG

ATACTTTTTATTCGCATACGCTATTTTACGATCAATTCCTAATAAATTAGGTGGAGTAATTGCTTTAGTTATATCAATTGCTATTTTATTAATTATACCT

TTAATTAACAAAAAAAAATTTAGTAGAACTCAATTTTACCCATTAAATAAAATTTTATTTTGATCTTTTGTCTCTATTGTAATCTTATTAACATGAATCG

GAGCTCGCCCAGTGGAAGATCCATACATTTTAACAGGTCAAATTTTAACAATTATATATTTTAGTTATTATTTCTTAAACCCCTTAATTCATAAAATATG

AGATTATATTATTTTTAAAACTTAGTTAATGAACTTGTTAAAGTGTATATTTTGAAAATATAAAAAAGAGTTTATTCTCTATTAACTTTACTAAATTTTA

TTCACTAAATAAAATAAGAAAAGATAGTCAACTTTAAACCTAAAAAAAAAAATAAAAAATTTAAAGAAACTGGTAAATAACTTTTTCAACATATATATAT

TAACTTATCATAACGATACCGAGGTAAAGTACCTCGAACTCAAATCCAAATAAATGCTATAAAAGTTAACTTCAAAAAAAAAAATAAAGAAAAAATATCT

CCCCCTATAAATAACAACACACATAATATACTTATAAATAAAATTCTAGCATACTCAGCCAAAAAGATTAAAGCAAATCCCCCTCTTCTGTACTCTACAT

TAAACCCAGAAACTAACTCTGATTCTCCTTCTGCAAAATCAAAAGGAGTCCGATTAGTCTCAGCTAAACTTGAAGATAATCATATTATTCTTAAAGGTAA

ACACAGAAAAAAAAATCATACTATTTCTTGATATTTTATAAAATCTATTATATTTAAATTTAAAATTAACAATAAAAAAGATAATAAAATTAAGGATAAA

CTTACTTCGTATGAAATTGTTTGAGCTACTGATCGAATACCCCCTAATAAAGCATAATTAGAATTTGATGATCAACCAGAAATTATGATAGTATAGACCC

TTAGCCTAGAACAACATAAAAAATATAAAATACCTAAATTAAACCTAAATATAAATGTCAAAAAAGGTATACATATCCATAATAAAAGAGCTAAAAATAA

ATTAAAAACTGGAGATATATAATAAATAAAAAAATTTGATATTAAAGGATAAGTTTGTTCTTTAGAAAATAACTTAATAGCATCACTAAAAGGTTGAGGA

ATACCTATAAATCCAACTTTATTGGGCCCTTTACGAATTTGGATATACCCTAAAACTTTGCGCTCTATTAATGTTAAAAAAGCTACACCAATTAAAACAC

AAATAATTAAAACTAAACTTGTAAATAATAAAAGAAATAAATCTTGTAATATAATGTATTACTTGTGTTAAACACATATTTAAATTCTAAATTTAAAGCA

CTAATCTGCCAAAGTAATATTCATATTCAAATTATATTAAATTTTAAAGGTATTTGATCCTTTCGTACTAAAATACCTATGTTTTTTAAAGATAGAAACC

AACCTGGCTCACGCCGGTTTAAACTCAGATCATGTAAAATTTTAAAGGTCGAACAGACCTAACCTTTTAGCCCCTACACCAAAAGTTAATTTTAATCCAA

CATCGAGGTCGCAAACTTTTTTTTCGATAAGAACTCTAAAAAAAA-TTACGCTGTTATCCCTAAGGTAATTTAATCTTGTAATCATTAAAAATGGATCAT

TCAATCATAAATTAATGTTTTTAAATAAAAAAAGTTTAATCAATTTTTCTGCTGCCCCAGCAAAATAGTTTAAATTATTAAATATATAAATATACTAAAA

TTAAATAATAATTTAAACTATAAAACTCTATAGGGTCTTCTCGTCTTTTAAAATTATATAAGCTTTTTTACTTATAAATAAAATTCTATATTCAATTAAA

TTGAGACAGTTACTTTCTCGTCCAACCGTTCATTCCAGCTTTCAATTAAAAAACTAATGATTATGCTACCTTTGCACGGTCAAATTACCGCGGCCATTCA

AATCCTCATTGGGCAGGTCAGACTTTAAATTATAATCAAAAAGACATGTTTTTAATAAACAGGCGAAAAGTGTATTTGCCGAGTTCCTTAATTTAACCTT

GAAGTTTTAATTTAATTACTAAATTAAAATATATACTAATTTTATCATTATTCTATATAAACCAATATTACATATATTATCTTAATAAACTACTTAAAAA

TAATATAAATCTTATTCTAACAAAAATTATTTATAACAAACTAAAGATTAACACTTCCAATTCTACTAATTTTTATTCAAAATATACATTTTTAACATTT

TATTTTAAAGCTTATCCCCTAAAATATTACTTTTTATATATAAAATACTAAATAATTAATATAATACAATAAAAAAACTAAATTAAATTTATTTCTTAAG

AAACTAGATATCTTAAAAAACGTATAACGTTTCATTTCTAATATAATATTTTAAAAATTTATGCCACAATTAAATTTATATTATATTAGCTCTTTATAAT

TCGAGAACACTAAATAATTAAATTATTTTAATAAACCCTGATACACAAGGTACAAAAAATTAATTTTTCTTTTTAAAAAATAAATCTCTATATATTTATA

TTATCTATCTCTATACAAATTAACTATAATAAAATTTTTATATTCTAAAATATACTAATATCAAAAATATTTTTTTTATATATATATATATATA--TTAT

AAATTTTTCCTTTCAAATTAAATTGATTTTCACAACTAACTTTTTAATGTAAATAAAATGCTTTATTACAAGCTCTAATTTGCCATTCCAGGTACACTTT

CCAGTACACCTACTATGTTACGACTTATCCCTCTTTAGAGAGGGAGCGACGGGCGATATGTACATATTCTAGAGCTATACTCATATAATTAAACTAAACT

ATATTACTTTCAAATCCACTTTATAAAATAATGTTAATTATTTTAACCATCTAAATAATTTTATTGTAACCCATCTCTCCTTATCTATACGCTGTATCTT

GATCTGATTTTTTTTATACTTATAAATTTTGAACATTCCAAATTCTTTAAAAACATTCAACCTACGACGATATACAAACCTTTAAAATAAGTACGATTAA

TCGTGGATCATCAATTATAGGACAGGTTCCTCTGAGTAGACTAAAATACCGCCAAATTCTTAAAATTTCAAGAACATAACTACTACTATTCAAGCATCTA

AAATTTGCATTTTTAATAATAGGGTATCTAATCCTAGTTTTTTATAAAAATCTCATAAACTCATTTTTCACATTTAAAAAATTAATTATACTTACTAATT

TCACCTAATAAATACAATATAAATTAATAATAAAATAACTTATTATATACTGAACAAATTTAATTGCATTGTTTGTGTAACCGCAACTGCTGGCACAAAC

TTGGTCAATACTATTATAAATTCCTAAATCAAAATTTCTTTTAAATTTAATCTTCACTATTGCAATTCTTTAATTAAATATACAGAATAATTTATCTTTT

TAAAATAAATTCATTAAACACTAAAATTTACATATAAAATAATTTAAAAATTAAAATCTCAAGCTAGAATAAAACTTTATTTTTTTTTCAATATACATAA

ATTATATAAAATAAAAGTACCCCCC-TACTATTTTAATTAAGTGAATTAATTTTTAATGCCTTAAATTTATTTCCAATTTATTTTTATTATATAGTTTTT

TATTTAAAAATAAAACTTTATACCTAAAATTATATACATAAATTATATTTAAAATTTATAATTTAAAGAACTATAAAAATTAAATTAAAATTTAAAATCT

ATCTAATTTATAGATAAACAAATTAAATTAAACTAACTTACCTGATAAATTTAACTATATACGCGCAGGTATATTAATTATTTAATTATAATATAATACA

TTTGTTTTATTAAATAATTATTAAGCAAAATAGCATTAATTTATTATATAAAAGATCTAGGTACTATTAATTTAAAGTAAATTTTTTTTTTTTTTGTAGT

ATAATTTATACTAATATACGGCATTTAATATCTTCTATATAAATGTTTATATTATTTATATTTATCTATTTATAATTCTTAATAATAATTTAGATAATTG

TTAATTTATATATTAAATATTTATATATATATATATAATAATAAGGATATATATATATATATGTATATAGATAAATTAAATTTAGTTATATATAAAAAAA

TTAATATTAATTTTACTTTTAATTTTTTCTTTAATTTAAACTATTGGATATTCAGATTCTAATATATTATAATATATCAACCGTTAATGAATAACTTTAT

ATATATAATAAAAATGTTAATTAATATATCAATTCATATGAATATATAATATTAATAAATAAATTCTTAATAAATAATAATGATAATATATATATATTGT

ATATTAATGTACTATATATATAAATTCATTAAACATTTATATTTGATACCCCCCCAAAATTTTTTCTAAAAATGTGAAGTTTCAATTTAATTAAAATTTT

CTACAATGGTCAAAAACGGCCAAAAAATGGAAAATAAAGTGTGCACAGAAAATGCACATAAAATGCACATTTTTGAAAATTTCCGATTTTTGAAAAACCG

TTTGAACCAAACCTCTGATGTACCTTTTTTCGCCCCCCCTGATTACTAAAATTTATCCTCAATTACTAAGGTTTATCCGCATTTTTGCGACAGGAATTTT

GAGTATAAAATCAATTCCACACTAAATTTTTTTTTGTTTTTTTTGTGTCATTTCTCCAACATGGGCAATTTTTTTTTTAGTCGAAGGACAAATTTTAGTA

AAAAAAGTTTGAAATTAGGTTAAACCCTTGTTTAACCAAAAACTATTACTTTCCAATATTATACCCAATTTTATTTAAAATAAATTTTATTATACTTTTA

TTTATGCAAAAAGATCTTCTTATAAACTTTT

>DMR175j_JP_Tsuruoka_Honshu

AATGAAGTGTCTGACTATAGAGTTACTTTGATAGAGTAAAAAAAGTGAATTTTCACCTTCATTATAATTAACAGAATTAAACTATTTCTTTAAGCTTCAA

AAACTTATGTACATTATATACTAAATTATAAAAAGATAAGCTAATTAAGCTACTGGGCTCATACCCCATCAATAAAGGTTACAATCCTTTTCTTTTTAAT

GTATTATAAGCTTTTATTTTTTAATTCTCTTATAATTGGAACCTTAATTGCTATTTCTTCCTACTCTTGGATAGGAATATGAATAGGATTAGAAATTAAT

CTTCTCTCTATGATCCCCCTCATTAGAGACAACAAAAATATAATAGCCTCAGAAGCTGCTTTAAAATATTTCATTATTCAAACAATAGCATCAACATTGT

TATTATTCTCAATTATTATAATATCAATGAAATTTATATATCAAATAAATTTAATCACTTACTTTAATTTAATTTTTAACACTTCATTGTTCATCAAAAT

AGGAGCAGCCCCATTCCATTTTTGATTCCCCGAAATAATAGAAGGATTAAATTGATTAAATGCCATTATCATACTTACTTGACAAAAACTAAGACCCATA

GTATTACTAACGTATTCTAATACAACCTCCATATATCTAATTTTAACAATTATATTTAGAATAATAATCAGAGGAATTATAGGTCTAAATCAAACTAGAT

TACGGAAAATTATAGCTTATTCATCTATCAACCATATTGGATGAATAATCAGTTCAATTATATTAATTGAAATTGTTTGATTTTACTATTTTATTATTTA

TTGCATTATTACTATTAATATCAGAATTATATTTATAAAATTAAATGTTTTTCATATCAATCAATTATATATTTCAATAAATTACCATATTTTACTTAAA

TTATTCTTTGCCTTAAATTTCATGTCTTTAGGAGGATTACCCCCATTTTTAGGGTTTTTCCCTAAATGACTTACAATTCAAACTTTAATCCAAAGAAATA

TGTACTCAATTGCTTTCATTATAATTTTAATAACTTTAATAACACTTTTTTTTTACCTCCGAATTACTTTTTCAATTTTACTACTAAGAAAAACAGTTTT

AACATTTTACACACAACCAAAAATTTATACTAATTACATTATAGCATTTAATTTTATTACATTATTAAGATTAATTTTCGTTACTTTGATATTCAATTTC

TTATAAATTAAACCTGAAGGATTTAAGTTAAATTAAACTAAGAACCTTCAAAGTTCTAAATAAAGTAAATTCTTTAAGCCTTAGGGCTTAGCCCATCTTT

AAATTTGCAATTTAAAATTCTTTTTGAACTATAAAGCTTGATAAAAGAAACTAATTTCGTATGTAAATTTACAGTTTACCGCCTAAACCTCGGCCATTTT

ATCGAATAAATGGCTATTCTCTACAAATCACAAAGATATCGGAACTTTATACTTTCTATTTGGAAGTTGAGCAGGCATAGTAGGCACTTCCTTAAGTTTA

CTGATTCGTGCTGAATTAGGAAACCCAGGATCTCTAATTGGAGATGATCAAATTTATAACGTAATCGTAACAGCTCATGCTTTCATTATAATTTTTTTTA

TAGTAATACCTATTATAATTGGAGGATTCGGAAACTGATTAGTTCCTTTAATGCTTGGAGCCCCCGACATAGCATTTCCCCGAATAAATAACATAAGATT

TTGACTTTTACCTCCTTCATTAACTCTACTTCTAATAAGAAGATTAGTCGAAAGAGGGGCAGGTACAGGCTGAACAGTTTATCCCCCACTATCAGCCAAT

ATCGCCCATAGAGGAGCATCAGTTGATTTAGCAATTTTTAGACTTCATCTTGCAGGAATTAGCTCAATTTTAGGTGCAGTAAATTTTATTACTACTGTAA

TTAACATACGATCAACAGGAATAACTTTTGATCGAATACCCCTATTTGTTTGATCTGTAGTTTTAACAGCACTTCTTCTGCTATTATCCCTCCCAGTTTT

AGCAGGAGCAATTACTATACTATTAACAGATCGAAATATTAATACAACATTTTTTGATCCCGCAGGTGGAGGAGACCCCATTCTCTACCAACATTTATTT

TGATTTTTTGGTCATCCAGAAGTTTACATTTTAATTTTACCTGGATTTGGTATAATCTCCCATATTATTAGCCAAGAAAGAAGAAAAAAAGAAACATTTG

GAACTTTAGGCATAATTTACGCTATAATAGCTATTGGTTTACTAGGATTTATTGTTTGAGCTCATCATATATTTACAGTAGGTATAGATGTAGACACACG

AGCATATTTTACATCAGCAACAATAATCATTGCTGTTCCTACAGGAATTAAAATTTTTAGATGATTAGCTACTCTCCATGGATCACAATTAAACTACTCC

CCGTCTCTTTTATGGGCATTAGGATTTGTATTCCTATTTACAGTAGGAGGATTAACAGGAGTAATTCTAGCTAATTCATCAATTGACATTATTTTACATG

ATACTTACTATGTAGTTGCACATTTCCATTATGTCCTTTCCATAGGAGCTGTATTTGCTATTATAGCAGGTTTTGTTCATTGATTCCCTTTATTTACAGG

TTTAACAATAAATTCAAAATTTCTTAAAATTCAATTTTTAACAATATTTATTGGTGTTAATATAACATTCTTCCCTCAACATTTCTTAGGATTAAGAGGA

ATACCTCGACGTTATTCAGATTACCCAGATGCTTATACAACTTGAAATATTATCTCATCTATTGGATCTTTAGTTTCTTTAATTAGTATTTTTATCTTTT

TATTTACTATTTGAGAAAGGCTAATTTCACTGCGAAAAAGAATTAGGTCTTTAAGAATATCTACATCAATTGAATGACTCCAACAAATACCCCCTTCAGA

ACATAGTTATTCTGAACTTCCAATGCTTACTAACTTCTAATATGGCAGATTAGTGCAATGGATTTAAACCCCAAATATAAAGATTAAACTTTTTTTAGAA

ATAGCTACTTGAAATACCATTTTACTTCAGGATAGGGCATCCCCATTAATAGAGCAACTCTCATTCTTTCATAACCATGCTCTTCTAATTCTCTTTATAA

TTACCGTTCTAGTAGGTTATTTAATAGGAACTTTATTTTTTAACCAATTTAATTACCGATTTTTATTAGATGGTCAAACTATTGAAATTATTTGAACTAT

TTTACCTGCTGTAACACTAATTTTTATCGCATTACCGTCTTTACGCTTACTTTATCTTCTAGATGAAGTTAATAACCCTTTAGTAACTATCAAAACAATT

GGGCATCAATGATATTGATCATACGAATATAGAGATTTTATAAATTTTGAATTCGATTCCTATATAATTCCTTTGACAGAAATAAAACCTCAAAATTTTC

GTTTATTAGATGTTGATAACCGAGTAATTGTCCCCTTTAACTCCCAAATCCGAATGATAGTAACAGCTGCCGATGTTATTCATTCATGAACTATCCCGGC

TTTTAGTGTAAAAATTGATGCAACACCAGGCCGACTAAATCAAATTAGATTCCTAATTAATCGAACAGGATTATTTTATGGTCAATGCTCAGAAATTTGT

GGGGCAAATCATAGATTTATACCTATTACTGTAGAAAGAATTTCACCTTCATTTTTTACTAAATGAATCTCAAAAATAAATAACCTATCATTAGATGACT

GAAAGTAAGTAATGGTCTCTTAAACCAATTAATAGTAGTTTAACATCTACTTCTGATGGCCAAAAATTTAGTTAAGATATAACATTAGTTTGTCATACTA

AAATAATCATAATTTGATAATTTTTAATTCCACAAATAGCACCTTTAAACTGACTATCTTTATTTTTTTTAATTATTATTATTTTTTTACTTTTTAATGT

ATTAAATTACTTTAGATTCTTACAGCCCTTAAAAACCCAATCTCATAACCCTACAATTAAAAAAATTAATTGAAAATGATAACTAATTTATTTTCATCTT

TTGATCCTAGAACTTCTTTTAATTTAAGATTAAACTGATTAAGAATACTATTAGGGCTAATATTTATCCCCCCAATATTTTGATTAGTTCCTTCACGCCA

TAATTTTCTATGAATTAAAATTATTTTAACATTACACCAAGAATTTAAGGTTTTAATTGGTAATAATAATATTAAAGGAAGAACCTTAATATTTATTTCA

TTATTTTCTATAATTGTTTTCAATAACTTTTTAGGATTATTTCCGTATATTTTTACAGGAACAAGACATTTAATTATAACATTATCTCTTGCCTTACCTT

TATGAATTAGATTCATATTATACGGGTGAATTAATAACACTATCCACATACTTGCTCATTTAGTTCCCCAAGGAACACCCCCAGCTCTTATGGCATTCAT

AGTAGTAATTGAATCAATTAGAAATATTATTCGTCCTGGTACTTTAGCTGTTCGATTAGCTGCTAATATAATTGCTGGACATTTACTAATAACTTTACTA

GGAAACACAGGATTAAATTTATCAATTTTTATACTAAGTATTCTTATTATCACACAAATTCTTTTATTAATTTTAGAATCTGCTGTTGCGATCATTCAAT

CTTATGTATTTGCTGTATTAAGAACTTTATACTCTAGAGAAATTAATTAATGTCAAGACATAAAAATCACCCTTATCATTTAGTTGATGCAAGACCTTGA

CCTATTTTAGGTGCTTTTAGAGCTATAATTACAATAATTGGAATTATCAAATGATTCCATTTTTATAATAATTCTTTATTTTACTTAGGGACATTAATCA

CAATTTTAATTATAATTCAATGATGACGAGATATCACTCGTGAGGGAACTTTCCAAGGACTTCATACTTACGCTGTAACTATAGGTTTACGTTGAGGAAT

AATTTTATTTATTACATCAGAAGTATTTTTCTTTATTTCTTTTTTTTGAGCCTTTTTTCATAGTAGCTTAACACCCGCTATTGAACTAGGGATACTCTGA

CCACCCAAAGGAATTACCCCATTTAACCCTATTCAAATTCCATTATTAAACACTTTAATTCTTTTAACTTCAGGATTAACTGTAACTTGAGCTCACCATA

GATTAATAGAAAATGACTATAACCAAACAATGCAAGGTCTTGGTTTAACAGTTTTACTAGGAGTATATTTTACTTTATTACAAGGTTACGAATATTTAGA

AGCCCCCTTTACTATAGCAGATTCTGTTTATGGATCAACATTTTTTATTGCTACTGGTTTCCATGGATTACATGTTATTATTGGCACAACCTTTTTAGCT

GTTTGTTTAATACGGCATTTTAATAACCATTTTACTTGTATCCATCACTTTGGATTTGAAGCTGCTGCTTGATACTGACATTTTGTTGATGTAGTATGAC

TATTTCTTTATATTTCTATTTACTGATGAGGTAGATATTTATATAGTATAATAATTATAATTGATTTCCAATCAAAAGATCTAAAAAAAATTAGTATAAA

TAATCATTATAATTTGAAATATAGGGTTAATTATTTTTTCTATCTCTTTTATCTTAATTATACTATCTTTTACAATCTCTAAAAAAAGATTTATAGACCG

AGAAAAAGCTTCTCCATTCGAGTGCGGATTTGACCCCAAAAGATCAGCCCGTTTACCCTTTTCTTTGCATTTTTTTTTAATTGCAGTAATTTTCTTAATT

TTTGATGTTGAAATTACTCTTCTTATCCCTTTAATTCTAACAATAAAAATTACTAATATTACTATATATACCTATATTGCTCTTTTCTTTTTAATGATTC

TTTTAATAGGACTTTACCATGAATGAAACCAAGGGGCCTTAAATTGAGCTCTTTAGGGTAATAGTTAAGTATAACATTTAAGTTGCATTTAAAAAGTATT

GATTTTTCAATTTACCTTAAATAAGAAACAATTAATTGTATTTAGTTTCGACCTAAAATTTAGGTGTATGAACACCCTTATTTAAATTAATTGAAACCAA

AAAGAGGTATATCACTGTTAATGATACTAATGAGAAAAACTCCAATTAAGGAAATAAGATATTCAAGAGTAAGCTTCTAACTTAACTCTTTAGCAGTGAA

AGTCTGTTAATATTTCTATTTATATAGTTTAATAAAACATTATTTTTTCATAATAAAATTAGAATAAATTTATTCTTATAAATATTTAAAAGTAAATTTT

ACTTCCCTGATAACTTCACTATCATACTCTATATAAGCTATTTAAATTAAATATATAAAATTATAAAAATTACCCATATTATAATTAAAAGTATAAAAAT

CTTATAATTGTTATTAAATATAAACTGTAAAAATACAGAAGTATTACTAATTTTACTATATAAATTTTGTCTTCCATAATACTCCGATCAGCCTTGATCA

ATAGTTTTATATAACTTACTACCTAATTTAATTGGATAATAATTTAAACCAAATGTTGAAATATAGGGTATATTCCACATAGAAGAAAAAAAAAGTCTTG

AATTTAACAAATAAAAAGATTTTAATCTATCATTTAAAGTAAACTTAGAAAGTTCAAACCCAAACCAAGCCCCAAAAAATGATACAATTAAAGCTATAAT

TTTTATTGTAAAAGGTAAACAAATAAAATAAGGTGTTGGGAATATCAACCATATTAATATTCTTCCCCCAACAATAACTAAAAAAATTAAACCTGATATC

CCTTGAAGTATAATTTTTCTATTATCATTAATTTTACTTAAAGAATAAAAACAAAAATTTCCTACCAAAACATAATAAATTAAACGAAATGTATAACAAA

CAGTTAATCCTGTTGAAAAAAAGAAAATAACATAAATATAGATATTTAAGTATCTTATAGATAAAACCTCTAAAATTAAATCCTTTGAATAAAACCCCGA

TAAAAAGGGTAAACCACATAATGCTAAATTTGAAATTATAAAATAAGTACAAGTTAAAGGTATAACTTTAATTAACCCCCCTATATACCGAATATCTTGA

CAATTTCTTAATCTGTGAATTATACACCCTGCGCATATAAATAATAAAGCCTTAAATAAAGCGTGAGTTAATAGGTGAAAAAAAGCTAATTGATACTCCC

CTAAAGCTAAAATACTAATTATCAAACCCAATTGTCTTAAAGTAGACAGAGCAATAATTTTTTTTAAATCAAACTCAAAGTTTGCGCCTATTCCTGCTAT

AAATATAGTTATTGTTCCAATAAATAATAAAATTAATATTAAATTACTTGTTAATGCAAAATTAAAACGAATTAATAAGTAAACACCTGCTGTTACTAAG

GTAGAAGAATGAACTAAAGAAGAAACAGGTGTTGGAGCCGCTATTGCCGCAGGCAACCAAGAAGAAAATGGAATTTGAGCTCTTTTAGTTATAGCTGCTA

ATATAATTAATAAAATAATAATATATATTTCTATACTATTTTTATATACATCAATATAAAAAATATAGTTAAATCCCCCAAAATTTATTATTCATGCAAT

TGCTATTAATAAAGCAACATCTCCAATACGGTTAGTTAAAGCTGTAATTATCCCTGCATTATAAGATTTAATATTTTGATAATAAATAACTAAACAATAA

GAAACTAACCCTAACCCATCTCATCCTAATAAAATCCTAATTAAATTAGGAGAAATAATCAATAACATTATAGATAAAACAAATATAGAAACTAATATAA

TAAATCGATGCAAATAAATATCTCCTTCTATATACTCTTCACTATAGTAAATTACTATAGAAGAAATAAATAAAACAAAACTCATAAATAATAATGACAT

CCAGTCAAGTAAAATGGTTATAATAATTCTACAAGAATTAATCCTTAATATTTCATATTCTAATATTAGTCTATAATCTAAAATTATGAAATTTAAGCTT

AATAAAAATCTTAATACTCTAAAAAATAAAAACGTTACAAAATAAATTAAACAAATAGAAATAATTTAAAGTAAATTTTACATCTTTGATACCACAAATC

AATATTTTTTATTAAACTATTTAAATTACAATCATAAAACTAAAAATTCTCTCTTTAAAATTAAGATATTCAAGGGTAACCAATGAAGTAATATTAATAA

ATACTCACGAACAAACCCTCTTGAAAAAGAATATAAATTTCTAACTAACTTGCCGTGTTGACTATAAGAATATAAATATAAAGAATACGCAGCTCTAAAA

AAAGATATTAAAGATAAAAAAACCATTGTTCAACTCCTTCATCTAACTAATCTATTAATTAAAATAATCTCACCCAGTAAGTTTAATGAAGGAGGAGCCG

CTATATTACAACAACTAAATAAAAATCATCATATTCTTATTATTGGTATTAAATTGATTAGCCCCTTATTTAAATAAATACTTCGGCTATTTAACCGTTC

ATAAGAAATATTTGCTAAACAAAATAATCCTGAAGAACATAAACCATGGGCAATTATTATAACTAAAGCCCCTCTTATACCCCAATAACTTAAAGTTAAA

ATCCCTCTTAATACTAAACCTATATGCGCTACAGAAGAATAAGCAATTAAAGCTTTAATATCAACTTGACGTAAACATATTAATGAAACAAAAAATCCCC

CTACTATTCTAATTGTAATAAAAATATAGTTTACTTGTAAACCTACAGTTAAAAAAATATTTATTAAACGTATTAATCCATACCCCCCTAATTTTAATAT

AACTCCAGCTAAAATTATAGAGCCAGCAACTGGAGCTTCAACATGGGCTTTAGGGAGCCAAAGATGAACAAAAAATATAGGCATTTTAATAAAAAACACT

ATATTTATACATATAAATAACAAGAATCTTTTTACATCATAACATAAAAAGAAAAAATCTAAAGAATGAAATTTTTCATAATAATAAAAAATCCTAATTA

TTATAGGTAATGAAGCAAATAATGTATAAAATAGCAAGTAAACACCTGCTTGCAAACGCTCAGGTTGATACCCCCAACCAATAATTAATAAAAGAGTAGG

AATTAAGCTAAACTCAAAAAACAAATAAAAAATAAATAAATTTAAAGAACTAAATGTTATAACTAAAGATAATAATAAAATAATTATTACTAATAAAAAT

AAATTATAAAAATAGTTTTTTCTGTAAATACTTTCAGAAGCTAATAATATTAAAGAACAAATCCACAAACTTAATAAAATTATTATAAAAGATAAAAGGT

CATACCCTATAAAATAAGAAATATTTATATACAAATAATTAAAACTAAATCTTAACCCAAATATAAATGTAATAAAAAAATATATATATTGATTAAATCA

GTATCTCTTTTTAATACAACTTAAAGGTACCAATATTAATATTATAAAAATAAACTTTATCATAATACATTAAAAGTTTGAAAATAATCATTTCCATGTG

TTCGTATTATAGAAACTAATAACGATAAACCTAAAGCTCCTTCACAAACTCTTATAGTTAAAAATACTATACCAAAATAAAATTCAAAATTAAAATATAT

TAAAAATAGATATAAATTAAAGTATAATCCTAAAATAATATACTCTAACCTTAATAATATTAAAAGTAAATGTTTACGTTTAATACAAAAAGAAACTAAT

CCTGTAAAATATATAATCACTGAAAATAACATACAAAAAATTAACATTAGTTTTAATAATTTAATAAAAATACTGGTCTTGTAAATCAGAAATAAGGATT

TTCTTTTAAAACTTCAGAGAAAGAGTAAACCTCTATCATTAATCTCCAAAATTAATATTTTAAATAAACTATTCTCTGTATAATCTTATTACTAATAACA

TTATCTTTTATTAGTTCAATAACTTTTATATTTTTAAGTCACCCTTTATCTATAGGGCTTATTTTATTAATACAAACTATTATTATAGCTTTAACTATAG

GTTTTTTTAATATTAATTTCTGATATTCTTATATTTTATTTCTTATTATAATTGGAGGAATATTAGTTTTATTTATTTATATAACAAGAGTAGCTTCAAA

TGAAAAATTTTCATTTTCTATTAAAATTACATTAATAATTAGAATTATAACTTTAGGATTTTTATTTAGTATTGCTATAATAGACCCCTACTTTTCAGAC

ATCAACTCAATTTACACAGAAAACTTAGATAATTATAAAGAATATAATATATCATTTAGAAAATATTTAAGTTATCCTAATATTATCATTATATACATAA

TAATTATTTATTTATTAATTACATTAATTGCAGTTGTAAAAATTACTCAAATTGAAAAAGGACCTTTACGTCAAACTAACTAATGAAAACACCATTACGA

AAAGCTTCCCCCCTATTAAAAATTATTAATAATAGAATTATTGATTTACCTACACCATCTAATATTTCTGCTTGATGAAATTTCGGATCCTTATTAGGAC

TCTGTCTTTTTATTCAAATTATCACTGGAATTTTCTTAGCTATACATTTTACAGCCCATATTGACATAGCATTTAATAGAGTAATTCATATTTGTCGAGA

TGTAAATTATGGCTGATTATTACGAACAATTCATGCTAACGGGGCTTCTTTTTTCTTTATTTGTATTTATCTACATATTGGCCGAGGAATATATTATAGA

AGCTATAATTTACATTTAACATGAACTATTGGAGTAATCATCCTTTTTATAGTAATAGCTACAGCATTCTTAGGGTATGTATTACCTTGAGGGCAAATAT

CTTTTTGAGGGGCTACTGTTATTACAAATCTTCTCTCTGCTATTCCTTATCTAGGAAATATGATTGTCCAATGATTGTGGGGGGGATTTGCAGTAGATAA

CGCCACTTTAACTCGATTTTTTACATTACATTTTATTCTTCCTTTTATTATTTTAGCTTTAATAATTATTCATTTATTATTTCTTCATCAAACTGGTTCT

AATAACCCTTTAGGATTAAATAGAAATATCGATAAAGTACCTTTCCATCCATATTTTACTTACAAAGATACTTTTGGATTTATTATAATAACTATATTAT

TAATATTTTTAGTTTTAATTAATCCCTATCTTTTAGGAGACCCAGAAAACTTTACACCCGCTAACCCTTTAGTAACCCCAGTCCATATCCAACCAGAATG

ATACTTTTTATTCGCATACGCTATTTTACGATCAATTCCTAATAAATTAGGTGGAGTAATTGCTTTAGTTATATCAATTGCTATTTTATTAATTATACCT

TTAATTAACAAAAAAAAATTTAGTAGAACTCAATTTTACCCATTAAATAAAATTTTATTTTGATCTTTTGTCTCTATTGTAATCTTATTAACATGAATCG

GAGCTCGCCCAGTGGAAGATCCATACATTTTAACAGGTCAAATTTTAACAATTATATATTTTAGTTATTATTTCTTAAACCCCTTAATTCATAAAATATG

AGATTATATTATTTTTAAAACTTAGTTAATGAACTTGTTAAAGTGTATATTTTGAAAATATAAAAAAGAGTTTATTCTCTATTAACTTTACTAAATTTTA

TTCACTAAATAAAATAAGAAAAGATAACCAACTTTAAACCTAAAAAAAAAAATAAAAAATTTAAAGAAACTGGTAAATAACTTTTTCAACATATATATAT

TAACTTATCATAACGATACCGAGGTAAAGTACCTCGAACTCAAATCCAAATAAATGCTATAAAAGTTAACTTCAAAAAAAAAAATAAAGAAAAAATATCT

CCCCCTATAAATAACAACACACATAATATACTTATAAATAAAATTCTAGCATACTCAGCCAAAAAGATTAAAGCAAATCCCCCTCTTCTGTACTCTACAT

TAAACCCAGAAACTAACTCTGATTCTCCTTCTGCAAAATCAAAAGGAGTCCGATTAGTCTCAGCTAAACTTGAAGATAATCATATTATTCTTAAAGGTAA

ACACAGAAAAAAAAATCATACTATTTCTTGATATTTTATAAAATCTATTATATTTAAATTTAAAATTAGCAATAAAAAAGATAATAAAATTAATGATAAA

CTTACTTCGTATGAAATTGTTTGAGCTACTGATCGAATACCCCCTAATAAAGCATAATTAGAATTTGATGATCAACCAGAAATTATGATAGTATAGACCC

TTAACCTAGAACAACATAAAAAATATAAAATACCTAAATTAAATCTAAATATAAATGTCAAAAAAGGTATACATATCCATAATAAAAGAGCTAAAAATAA

ATTAAAAACTGGAGATATATAATAAATAAAAAAATTTGATATTAAAGGATAAGTTTGTTCTTTAGAAAATAACTTAATAGCATCACTAAAAGGTTGAGGA

ATACCTATAAATCCAACTTTATTGGGTCCTTTACGAATTTGGATATACCCTAAAACTTTGCGCTCTATTAATGTTAAAAAAGCTACACCAATTAAAACAC

AAATAATTAAAACTAAACTTGTAAATAATAAAAGAAATAAATCTTGTAATATAATGTATTACTTGTGTTAAACACATATTTAAATTCTAAATTTAAAGCA

CTAATCTGCCAAAGTAATATTCATATTCAAATTATATTAAATTTTAAAGGTATTTGATCCTTTCGTACTAAAATACCTATGTTTTTTAAAGATAGAAACC

AACCTGGCTCACGCCGGTTTAAACTCAGATCATGTAAAATTTTAAAGGTCGAACAGACCTAACCTTTTAGCCCCTACACCAAAAGTTAATTTTAATCCAA

CATCGAGGTCGCAAACTTTTTTTTCGATAAGAACTCTAAAAAAAAATTACGCTGTTATCCCTAAGGTAATTTAATCTTGTAATCATTAAAAATGGATCAT

TCAATCATAAATTAATGTTTTTAAATAAAAAAAGTTTAATCAATTTTTCTGCTGCCCCAGCAAAATAGTTTAAATTATTAAATATATAAATATACTAAAA

TTAAATAATAATTTAAACTATAAAACTCTATAGGGTCTTCTCGTCTTTTAAAATTATATAAGCTTTTTTACTTATAAATAAAATTCTATATTCAATTAAA

TTGAGACAGTTACTTTCTCGTCCAACCGTTCATTCCAGCTTTCAATTAAAAAACTAATGATTATGCTACCTTTGCACGGTCAAATTACCGCGGCCATTCA

AATCCTCATTGGGCAGGTCAGACTTTAAATTATAATCAAAAAGACATGTTTTTAATAAACAGGCGAAAAGTGTATTTGCCGAGTTCCTTAATTTAACCTT

GAAGTTTTAATTTAATTACTAAATTAAAATATATACTAATTTTATCATTATTCTATATAAACCAATATTACATATATTATCTTAATAAATTACTTAAAAA

TAATATAAATCTTATTCTAACAAAAATTATTTATAACAAACTAAAGATTAACACTTCCAATTCTACTAATTTTTATTCAAAATATACATTTTTAACATTT

TATTTTAAAGCTTATCCCCTAAAATATTACTTTTTATATATAAAATACTAAATAATTAATATAATACAATAAAAAAACTAAATTAAATTTATTTCTTAAG

AAACTAGATATCTTAAAAAACGTATAACGTTTCATTTCTAATATAATATTTTAAAAATTTATGCCACAATTAAATTTATATTATATTAGCTCTTTATAAT

TCGAGAACACTAAATAATTAAATTATTTTAATAAACCCTGATACACAAGGTACAAAAAATTAATTTTTCTTTTTAAAAAATAAATCTCTATATATTTATA

TTATCTATCTCTATACAAATTAACTATAATAAAATTTTTATATTCTAAAATATACTAATATCAAAAATATTTTTTTTATATATATATATATATATATTAT

AAATTTTTCCTTTCAAATTAAATTGATTTTCACAACTAACTTTTTAATGTAAATAAAATGCTTTATTACAAGCTCTAATTTGCCATTCCAGGTACACTTT

CCAGTACACCTACTATGTTACGACTTATCCCTCTTTAGAGAGGGAGCGACGGGCGATATGTACATATTCTAGAGCTATACTCATATAATTAAACTAAACT

ATATTACTTTCAAATCCACTTTATAAAATAATGTTAATTATTTTAACCATCTAAATAATTTTATTGTAACCCATCTCTCCTTATCTATACGCTGTATCTT

GATCTGATTTTTTTTATACTTATAAATTTTGAACATTCCAAATTCTTTAAAAACATTCAACCTACGACGATATACAAACCTTTAAAATAAGTACGATTAA

TCGTGGATCATCAATTATAGGACAGGTTCCTCTGAGTAGACTAAAATACCGCCAAATTCTTAAAATTTCAAGAACATAACTACTACTATTCAAGCATCTA

AAATTTGCATTTTTAATAATAGGGTATCTAATCCTAGTTTTTTATAAAAATCTCATAAACTCATTTTTCACATTTAAAAAATTAATTATACTTACTAATT

TCACCTAATAAATACAATATAAATTAATAATAAAATAACTTATTATATACTGAACAAATTTAATTGCATTGTTTGTGTAACCGCAACTGCTGGCACAAAC

TTGGTCAATACTATTATAAATTCCTAAATCAAAATTTCTTTTAAATTTAATCTTCACTATTGCAATTCTTTAATTAAATATATAGAATAATTTATCTTTT

TAAAATAAATTCATTAAACACTAAAATTTACATATAAAATAATTTAAAAATTAAAATCTCAAGCTAGAATAAAACTTTATTTTTTTTTCAATATACATAA

ATTATATAAAATAAAAGTACCCCCC-TACTATTTTAATTAAGTGAATTAATTTTTAATGCCTTAAATTTATTCCCAATTTATTTTTATTATATAGTTTTT

TATTTAAAAATAAAACTTTATACCTAAAATTATATACATAAATTATATTTAAAATTTATAATTTAAAGAACTATAAAAATTAAATTAAAATTTAAAATCT

ATCTAATTTATAGATAAACAAATTAAATTAAACTAATTTACCTGATAAATTTAACTATATACGCGCAGGTATATTAATTATTTAATTATAATATAATACA

TTTGTTTTATTAAATAATTATTAAGCAAAATAGCATTAATTTATTATATAAAAGATCTAGGTACTATTAATTTAAAGTAAATTTTTTTTTTTTTTGTAGT

ATAATTTATACTAATATACGGCATTTAATATCTTCTATATAAATGTTTATATTATTTATATTTATCTATTTATAATTCTTAATAATAATTTAGATAATTA

TTAATTTATATATTAAATATTTATATATATATATATAATAATAAGGATATATATATATATATGTATATAGATAAATTAAATTTAGTTATATATAAAAAAA

TTAATATTAATTTTACTTTTAATTTTTTCTTTAATTTAAACTATTGGATATTCAGATTCTAATATATTATAATATATCAACCATTAATGAATAACTTTAT

ATATATAATAAAAATGTTAATTAATATATCAATTCATATGAATATATAATATTAATAAATAAATTCTTAATAAATAATAATGATAATATATATATATTGT

ATATTAATGTACTATATATATAAATTCATTAAACATTTATATTTGATACCCCCCCAAAATTTTTTCTAAAAATGTGAAGTTTCAATTTAATTAAAATTTT

CTACAATGGTCAAAAACGGCCAAAAAATGGAAAATAAAGTGTGCACAGAAAATGCACATAAAATGCACATTTTTGAAAATTTCCGATTTTTGAAAAACCG

TTTGAACCAAACCTCTGATGTACCTTTTTTCGCCCCCCCTGATTACTAAAATTTATCCTCAATTACTAAGGTTTATCCGCATTTTTGCGACAGGAATTTT

GAGTATAAAATCAATTCCACACTAAATTTTTTTTT-TTTTTTTTGTGTCATTTCTCCAACATGGGCAATTTTTTTTTTAGTCGAAGGACAAATTTTAGTA

AAAAAAGTTTGAAATTAGGTTAAACCCTTGTTTAACCAAAAACTATTACTTTTCAATATTATACCCAATTTTATTTAAAATAAATTTTATTATACTTTTA

TTTATGCAAAAAGATTTTCTTATAAACTTTT

>DMR176j_JP_Tsuruoka_Honshu

AATGAAGTGTCTGACTATAGAGTTACTTTGATAGAGTAAAAAAAGTGAATTTTCACCTTCATTATAATTAACAGAATTAAACTATTTCTTTAAGCTTCAA

AAACTTATGTACATTATATACTAAATTATAAAAAGATAAGCTAATTAAGCTACTGGGCTCATACCCCATCAATAAAGGTTACAATCCTTTTCTTTTTAAT

GTATTATAAGCTTTTATTTTTTAATTCTCTTATAATTGGAACCTTAATTGCTATTTCTTCCTACTCTTGGATAGGAATATGAATAGGATTAGAAATTAAT

CTTCTCTCTATGATCCCCCTCATTAGAGACAACAAAAATATAATAGCCTCAGAAGCTGCTTTAAAATATTTCATTATTCAAACAATAGCATCAACATTGT

TATTATTCTCAATTATTATAATATCAATGAAATTTATATATCAAATAAATTTAATCACTTACTTTAATTTAATTTTTAACACTTCATTATTCATCAAAAT

AGGAGCAGCCCCATTCCATTTTTGATTCCCCGAAATAATAGAAGGATTAAATTGATTAAATGCCATTATCATACTTACTTGACAAAAACTAAGACCCATA

GTATTACTAACGTATTCTAATACAACCCCCATATATCTAATTTTAACAATTATATTTAGAATAATAATCAGAGGAATTATAGGTCTAAATCAAACTAGAT

TACGAAAAATTATAGCTTATTCATCTATCAACCATATTGGATGAATAATCAGTTCAATTATACTAATTGAAATTGTTTGATTTTACTATTTTATTATTTA

TTGCATTATTACTATTAATATCAGAATTATATTTATAAAATTAAATGTTTTCCATATCAATCAATTATATATTTCAATAAATTACCATATTTTACTTAAA

TTATTCTTTGCCTTAAATTTCATGTCTTTAGGAGGATTACCCCCATTTTTAGGGTTTTTCCCTAAATGACTTACAATTCAAACTTTAATCCAAAGAAATA

TATACTCAATTGCTTTCATTATAATTTTAATAACTTTAATAACACTTTTTTTTTACCTCCGAATTACTTTTTCAATTTTACTACTAAGAAAAACAGTTTT

AACATTTTACGCACAACCAAAAATTTATACTAATTACATTATAGCATTTAATTTTATTACATTATTAAGATTAATTTTCGTTACTTTGATATTCAATTTC

TTATAAATTAAACCTGAAGGATTTAAGTTAAATTAAACTAAGAACCTTCAAAGTTCTAAATAAAGTAAATTCTTTAAGCCTTAGGGCTTAGCCCATCTTT

AAATTTGCAATTTAAAATTCTTTTTGAACTATAAAGCTTGATAAAAGAAACTAATTTCGTATGTAAATTTACAGTTTACCGCCTAAACCTCGGCCATTTT

ATCGAATAAATGGCTATTCTCTACAAATCACAAAGATATCGGAACTTTATACTTTCTATTTGGAAGTTGAGCAGGCATAGTAGGCACTTCCTTAAGTTTA

TTGATTCGTGCTGAATTGGGAAACCCAGGATCTCTAATTGGAGATGATCAAATTTATAACGTAATCGTAACAGCTCATGCTTTCATTATAATTTTTTTTA

TAGTAATACCTATTATAATTGGGGGATTCGGAAACTGATTAGTTCCTTTAATGCTTGGAGCCCCCGACATAGCATTTCCCCGAATAAATAACATAAGATT

TTGACTTTTACCTCCTTCATTAACTCTACTTCTAATAAGAAGATTAGTCGAAAGAGGAGCAGGTACAGGCTGAACAGTTTATCCCCCACTATCAGCCAAT

ATCGCCCATAGAGGAGCATCAGTTGATTTAGCAATTTTTAGACTTCATCTTGCAGGAATTAGCTCAATTTTAGGTGCAGTAAATTTTATTACTACTGTAA

TTAACATACGATCAACAGGAATAACTTTTGATCGAATACCCCTATTTGTTTGATCTGTAGTTTTAACAGCACTTCTTCTGCTATTATCCCTCCCAGTTTT

AGCAGGAGCAATTACTATACTATTAACAGATCGAAATATTAACACAACATTTTTTGACCCCGCAGGTGGAGGAGACCCCATTCTCTACCAACATTTATTT

TGATTTTTTGGTCATCCAGAAGTTTACATTTTAATTTTACCTGGATTTGGTATAATCTCCCATATTATTAGCCAAGAAAGAAGAAAAAAAGAAACATTTG

GAACTTTAGGTATAATTTACGCTATAATAGCTATTGGTTTACTAGGATTTATTGTATGAGCTCATCATATATTTACAGTAGGTATAGATGTAGACACACG

GGCATATTTTACATCAGCAACAATAATCATTGCTGTTCCTACAGGAATTAAAATTTTTAGATGATTAGCTACTCTCCATGGATCACAATTAAACTATTCC

CCGTCTCTTTTATGGGCATTGGGATTTGTATTCCTATTTACAGTAGGAGGATTAACAGGGGTAATTCTAGCTAATTCATCAATTGACATTATTTTACATG

ATACTTACTATGTAGTTGCACATTTCCATTATGTCCTTTCCATAGGAGCTGTATTTGCTATTATAGCAGGTTTTGTTCATTGATTCCCTTTATTCACAGG

TTTAACAATAAATTCAAAATTTCTTAAAATTCAATTTTTAACAATATTTATTGGTGTTAATATCACATTCTTCCCCCAACATTTCTTAGGATTAAGAGGA

ATACCTCGACGTTATTCAGATTACCCAGATGCTTATACAACTTGAAATATTATCTCATCTATTGGATCTTTAGTTTCTTTAATTAGTATTTTTATCTTTT

TATTTACTATTTGAGAAAGGTTAATTTCATTACGAAAAAGAATTAGGTCTTTAAGAATATCTACATCAATTGAATGACTCCAACAAATACCCCCTTCAGA

ACATAGCTATTCTGAACTTCCAATGCTTACTAACTTCTAATATGGCAGATTAGTGCAATGGATTTAAACCCCAAATATAAAGATTAAACTTTTTTTAGAA

ATAGCTACTTGAAATACCATTTTACTTCAAGATAGGGCATCCCCATTAATAGAGCAACTCTCATTCTTTCATAACCATGCTCTTCTAATTCTCTTTATAA

TTACCGTTCTAGTAGGTTATTTAATAGGAACTTTATTTTTTAACCAATTTAATTACCGATTTTTATTAAATGGTCAAACTATTGAAATTATTTGAACTAT

TTTACCTGCTGTAACACTAATTTTTATCGCATTACCGTCTTTACGCTTACTTTATCTTCTAGATGAAGTTAATAACCCTTTAGTAACTATCAAAACAATT

GGGCATCAATGATATTGATCATACGAATATAGAGATTTTATAAATTTTGAATTCGATTCCTATATAATTCCTTTGACAGAAATAAAACCTCAAAATTTTC

GTTTATTAGATGTTGATAACCGAGTAATTGTCCCCTTTAACTCCCAAATCCGAATGATAGTAACAGCTGCCGATGTTATTCATTCATGAACTATCCCAGC

TTTTAGTGTAAAAATTGATGCAACACCGGGCCGACTAAATCAAATTAGATTCCTAATTAATCGAACAGGATTATTTTATGGTCAATGCTCAGAAATTTGT

GGAGCAAATCATAGATTTATACCTATTACTGTAGAAAGAATTTCACCTTCATTTTTTACTAAATGAATCTCAAAAATAAATAACCTATCATTAGATGACT

GAAAGTAAGTAATGGTCTCTTAAACCAATTAATAGTAGTTTAACATCTACTTCTGATGGCCAAAAATTTAGTTAAGATATAACATTAGTTTGTCATACTA

AAATAATCATAATTTGATAATTTTTAATTCCACAAATAGCACCTTTAAACTGACTATCTTTATTTTTTTTAATTATTATTATTTTTTTACTTTTTAATGT

ATTAAATTACTTTAGATTCTTACAACCTTTAAAAACCCAATCTCATAACCCTACAATTAAAAAAATTAATTGAAAATGATAACTAATTTATTTTCATCTT

TTGATCCTAGAACTTCTTTTAATTTGAGATTAAACTGATTAAGAATACTATTAGGGCTAATATTTATCCCCCCAATATTTTGATTAGTTCCTTCACGCCA

TAATTTTCTATGAATTAAAATTATTTTAACATTACACCAAGAATTTAAGGTTTTAATTGGTAATAATAATATTAAAGGAAGAACCTTAATATTTATTTCA

TTATTTTCTATAATTGTTTTCAATAACTTTTTAGGATTATTTCCGTATATTTTTACAGGAACAAGGCATTTAATTATAACATTATCTCTTGCCTTACCTT

TATGAATTAGATTCATATTATACGGGTGAATTAATAACACTATCCACATACTTGCTCATTTAGTTCCCCAAGGAACACCCCCAGCTCTTATGGCATTCAT

AGTAGTAATTGAATCAATTAGAAATATTATTCGTCCTGGTACTTTAGCTGTTCGATTAGCTGCTAATATAATTGCTGGACATTTACTAATAACTTTACTA

GGAAACACAGGATTAAATTTATCAATTTTTATATTAAGTATTCTTATTATCATACAAATTCTTTTATTAATTTTAGAATCTGCTGTTGCGATCATTCAAT

CTTATGTATTTGCTGTATTAAGAACTTTATACTCTAGAGAAATTAATTAATGTCAAGACATAAAAATCACCCTTATCATTTAGTTGATGCAAGGCCTTGA

CCTATTTTAGGTGCTTTTAGAGCTATAATTACAATAATTGGAATTATCAAATGATTCCATTTTTATAATAATTCTTTATTTTACTTAGGGACATTAATTA

CAATTTTAATTATAATTCAATGATGACGAGATATCACTCGTGAGGGAACTTTCCAAGGACTTCATACTTACGCTGTAACTATAGGTTTACGTTGAGGAAT

AATTTTATTTATTACATCAGAAGTATTTTTCTTTATTTCTTTTTTTTGAGCCTTTTTTCATAGTAGTTTAACACCCGCTATTGAACTAGGGATACTCTGA

CCACCTAAAGGAATTACTCCATTTAACCCTATTCAAATTCCATTATTAAACACTTTAATTCTTTTAACTTCGGGATTAACTGTAACTTGAGCTCACCATA

GATTAATAGAAAATGACTATAACCAAACAATACAAGGTCTTGGTTTAACAGTTTTACTAGGAATATATTTTACTTTATTACAAGGTTACGAATATTTAGA

AGCCCCCTTTACTATAGCAGATTCTGTTTATGGATCAACATTTTTTATTGCTACTGGTTTCCATGGATTACATGTTATTATTGGCACAACCTTTTTAGCT

GTTTGTTTAATACGGCATTTTAATAACCATTTTACTTGCATCCATCACTTTGGATTTGAAGCTGCTGCTTGATACTGACATTTTGTTGATGTAGTATGAC

TATTTCTTTATATTTCTATTTACTGATGAGGTAGATATTTATATAGTATAATAATTATAATTGATTTCCAATCAAAAGATCTAAAAAA--TTAGTATAAA

TAATCATTATAATTTGAAATATAGGGTTAATTATTTTTTCTATCTCTTTTATCTTAATTATACTATCTTTTACAATCTCTAAAAAAAGATTTATAGACCG

AGAAAAAGCTTCTCCATTCGAGTGTGGATTTGACCCCAAAAGATCAGCCCGTTTACCCTTTTCTTTGCATTTTTTTTTAATTGCAGTAATTTTCTTAATT

TTTGATGTTGAAATTACTCTTCTTATCCCTTTAATTCTAACAATAAAAATTACTAATATTACTATATATACCTATATTGCTCTTTTCTTTTTAATGATTC

TTTTAATAGGACTTTACCATGAATGAAACCAAGGAGCCTTAAATTGAGCTCTTTAGGGTAATAGTTAAGTATAACATTTAAGTTGCATTTAAAAAGTATT

GATTTTTCAATTTACCTTAAATAAGAAACAATTAATTGTATTTAGTTTCGACCTAAAATTTAGGTGTATGAACACCCTTATTTAAATTAATTGAAACCAA

AAAGAGGTATATCACTGTTAATGATACTAATGAGAAAAGCTCCAATTAAGGAAATAAGATATTCAAGAGTAAGCTTCTAACTTAACTCTTTAGCAGTGAA

AGTCTGTTAATATTTCTATTTATATAGTTTAATAAAACATTATTTTTTCATAATAAAATTAGAATAAATTTATTCTTATAAATATTTAAAAGTAAATTTT

ACTTCCCTGATAACTTCACTATCATACTCTCTATAAGCTATTTAAATTAAATATATAAAATTATAAAAATTACCCATATTATAATTAAAAGTATAAAAAT

CTTATAATTGTTATTAAATATAAACTGTAAAAATACAGAAGTATTACTAATTTTACTATATAAATTTTGTCTTCCATAATACTCTGATCACCCTTGATCA

ATAGTTTTATATAACTTACTACCTAATTTAATTGGATAATAATTTAAACCAAATGTTGAAATATAAGGTATATTCCACATAGAAGAAAAAAAAAGTCTTG

AATTTAACAAATAAAAAGATTTTAATCTATCATTTAAAGTAAACTTAGAAAGTTCAAACCCAAACCAAGCCCCAAAAAATGATACAATTAAAGCTATAAT

TTTTATTGTAAAAGGTAAACAAATAAAATAAGGTGTTGGGAATATCAGCCATATCAATATTCTTCCCCCAACAATAACTAAAAAAATTAAACCTGATATC

CCTTGAAGTATAATTTTTCTATTATCATTAATTTTACTTAAAGAATAAAAACAAAAATTTCCTACTAAAACATAATAAATTAAACGAAATGTATAACAAA

CAGTTAATCCTGTTGAAAAAAAGAAAATAACATAAATATAGATATTTAAATATCTTATAGATAAAACCTCTAAAATTAAATCCTTTGAATAAAACCCTGA

TAAAAAGGGTAAACCACATAATGCTAAATTTGAAATTATAAAATAAGTACAAGTTAAAGGTATAACTTTAATTAACCCCCCTATATACCGAATATCTTGA

CAATTTCTTAATCTGTGAATTATACACCCTGCGCATATAAATAATAAAGCCTTAAATAAAGCGTGAGTTAATAGATGAAAAAAAGCTAATTGATACTCCC

CTAAAGCTAAAATACTAATTATCAAACCCAATTGTCTTAAAGTAGATAGAGCAATAATTTTTTTTAAATCAAATTCAAAGTTTGCACCTATTCCTGCTAT

AAATATAGTTATTGTTCCAATAAATAATAAAATTAATATTAAATTACTTGTTAATGCAAAATTAAAACGAATTAATAAGTAAACACCTGCTGTTACTAAG

GTAGAAGAATGAACTAAAGAAGAAACAGGCGTTGGAGCTGCTATTGCCGCAGGCAACCAAGAAGAAAATGGAATTTGAGCTCTTTTAGTTATAGCTGCTA

ATATAATTAATAAAATAATAATATATATTTCTATGCTATTTTTATACACATCAATATAAAAAATATAGTTAAATCCCCCAAAATTTATTATTCATGCAAT

TGCTATTAATAAAGCAACATCTCCAATACGGTTAGTTAAAGCTGTAATTATCCCTGCATTATAAGATTTAATATTTTGATAATAAATAACTAAACAATAA

GAAACTAACCCTAACCCATCTCATCCTAATAAAATCCTAATTAAATTAGGAGAAATAATCAATAACATTATAGATAAAACAAATATAGAAACTAATATAA

TAAATCGATGCAAATAAATATCTCCTTCTATATACTCTTCACTATAGTAAATTACTATAGAAGAAATAAATAAAACAAAACTCATAAATAATAATGACAT

CCAGTCAAGTAAAATAGTTATAATAATTCTACAAGAATTAATCCTTAATATTTCATATTCTAATATTAGTCTATAATCTAAAATTATGAAATTTAAGCTT

AATAAAAATCTTAATACTCTAAAAAATAAAAACGTTACAAAATAAATTAAACAAATAGAAATAATTTAAAGTAAATTTTACATCTTTGATACCACAAATC

AATATTTTTTATTAAACTATTTAAATTACAATCATAAAACTAAAAATTCTCTCTTTAAAATTAAGATATTCAAGGGTAACCAATGAAGTAATAATAATAA

ATACTCACGAACAAACCCTCTTGAAAAAGAATATAAATTTCTAACTAACTTGCCGTGTTGACTATAAGAATATAAATATAAAGAATACGCAGCTCTAAAA

AAAGATATTAAAGATAAAAAAACCATTGTTCAACTCCTTCATCTAACTAATCTATTAATTAAAATAATCTCACCCAGTAAGTTTAATGAAGGAGGAGCCG

CTATATTACAACAACTAAATAAAAATCACCATATTCTTATTATTGGCATTAAATTGATTAGCCCCTTATTTAAATAAATACTTCGGCTATTTAGCCGTTC

ATAAGAAATATTTGCTAAACAAAATAATCCTGAAGAACATAAACCATGGGCAATTATTATAACTAAAGCCCCTCTTATACCCCAATAACTTAAAGTTAAA

ATCCCTCTTAATACTAAACCTATATGCGCTACAGAAGAATAAGCAATTAAAGCTTTAATATCAACTTGACGTAAACATATTAATGAAACAAAAAATCCCC

CTACTATTCTAATTGTAATAAAAATATAGTTTACTTGTAAACCTACAGTTAAAAAAATATTTATTAAACGTATTAATCCATACCCCCCTAATTTTAATAT

AACTCCAGCTAAAATTATAGAGCCAGCAACTGGAGCTTCAACATGGGCTTTAGGGAGCCAAAGATGAACAAAAAATATAGGCATTTTAATAAAAAACACT

ATATTTATACATATAAATAACAAGAATCTTTTTACATCATAACATAAAAAGAAAAAATCTAAAGAATGAAATTTTTCATAATAATAAAAAATCCTAATTA

TTATAGGTAATGAAGCAAATAATGTATAAAATAACAAGTAAACACCTGCTTGCAAACGCTCAGGTTGATACCCCCAACCAATAATTAATAGAAGAGTGGG

AATTAAGCTAAACTCAAAAAACAAATAAAAAATAAATAAATTTAAAGAACTAAATGTTATAACTAAAGATAATAATAAAATAATTATTACTAATAAAAAT

AAATTATAAAAATAATTTTTTCTATAAATACTTTCAGAAGCTAATAATATTAAAGAACAAATCCACAAACTTAATAAAATTATTATAAAAGATAAAAGGT

CATACCCTATAAAATAAGAAATATTTATATACAAATAATTAAAACTAAATCTTAACCCAAATATAAATGTAATAAAAAAATATATATATTGATTAAATCA

GTATCTCTTTTTAACACAACTTAAAGGTACCAATATTAATATTATAAAAATAAACTTTATCATAATACATTAAAAGTTTGAAAATAATCATTTCCATGTG

TTCGTATTATAGAAACTAATAACGATAAACCTAAAGCTCCTTCACAAACTCTTATAGTTAAAAATACTATACCAAAATAAAATTCAAAATTAAAATATAT

TAAAAATAGGTATAAATTAAAGTATAATCCTAAAATAATATACTCTAACCTTAATAATATTAAAAGTAAATGTTTACGTTTAATGCAAAAAGAAACTAAT

CCTGTAAAATATATAATCACTGAAAATAACATACAAAAAATTAACATTAGTTTTAATAATTTAATAAAAATACTGGTCTTGTAAATCAGAAATAAGAATT

TTCTTTTAAAACTTCAGAGAAAGAGTAAACCTCTATCATTAATCTCCAAAATTAATATTTTAAATAAACTATTCTCTGTATAATCTTATTACTAATAACA

TTATCTTTTATTAGTTCAATAACTTTTATATTTTTAAGTCACCCTTTATCTATAGGGCTTATTTTATTAATACAAACTATTATTATAGCTTTAACTATAG

GTTTTTTTAATATTAATTTCTGATATTCTTATATTTTATTTCTTATTATAATTGGAGGAATATTAGTTTTATTTATTTATATAACAAGAGTAGCTTCAAA

TGAAAAATTTTCATTTTCTATTAAAATTACATTAATAATTAGAATTATAACTTTAGGATTTTTATTTAGTATTGCTATAATAGATCCCTACTTTTCAGAC

ATCAACTCAATTTACACAGAAAACTTAGATAATTATAAAGAATATAATATATCATTTAGAAAATATTTAAGTTATCCTAATATTATCATTATATACATAA

TAATTATTTATTTATTAATTACCTTAATTGCAGTTGTAAAAATTACTCAAATTGAAAAAGGACCTTTACGTCAAACTAACTAATGAAAACACCATTACGA

AAAGCTTCTCCCCTATTAAAAATTATTAATAATAGAATTATTGATTTACCTACACCATCTAATATTTCTGCTTGATGAAATTTTGGATCCTTATTAGGAC

TCTGTCTTTTTATTCAAATTATCACTGGAATTTTCTTAGCTATACATTTTACAGCCCATATTGACATAGCATTTAATAGAGTAATTCATATTTGTCGAGA

TGTAAATTATGGCTGATTATTACGAACAATTCATGCTAACGGAGCTTCTTTTTTCTTTATTTGTATTTATCTACATATTGGCCGAGGAATATATTATAGA

AGCTATAATTTACATTTAACATGAACTATTGGAGTAATCATCCTTTTTATAGTAATAGCTACAGCATTCTTAGGGTATGTATTACCTTGAGGGCAAATAT

CTTTTTGAGGGGCTACTGTTATTACCAATCTTCTCTCTGCTATTCCTTATCTAGGAAATATGATTGTCCAATGATTATGAGGGGGATTTGCAGTAGATAA

CGCTACTTTAACTCGATTTTTTACATTACATTTTATTCTTCCTTTTATTATTTTAGCTTTAATAATTATTCATTTATTATTTCTTCATCAAACTGGTTCT

AATAACCCTTTAGGATTAAATAGAAATATCGATAAAGTACCTTTCCATCCATATTTTACTTACAAAGATACTTTTGGATTCATTATAATAACTATATTAT

TAATATTTTTAGTTTTAATTAATCCCTATCTTTTAGGAGACCCAGAAAACTTTACACCTGCTAATCCTTTAGTAACCCCAGTCCATATCCAACCAGAATG

ATACTTTTTATTTGCATACGCTATTTTACGATCAATTCCTAATAAATTAGGTGGAGTAATTGCTTTAGTTATATCAATTGCTATTTTATTAATTATACCT

TTAATTAACAAAAAAAAATTTAGTAGAACTCAATTTTACCCATTAAATAAAATTTTATTTTGATCTTTTGTCTCTATTGTAATCTTATTAACATGAATCG

GAGCTCGCCCAGTGGAAGATCCATACATTTTAACAGGTCAAATTTTAACAATTATATATTTTAGTTATTATTTCTTAAACCCCTTAATTCATAAAATATG

AGATTATATTATTTTTAAAACTTAGTTAATGAACTTGTTAAAGTGTATATTTTGAAAATATAAAAAAGAGTTTATTCTCTATTAACTTTACTAAATTTTA

TTCACTAAATAAAATAAGTAAAGATAACCAACTTTAAACCTAAAAAAAAAAATAAAAAATTTAAAGAAACTGGTAAATAACTTTTTCAACATATATATAT

TAACTTATCATAACGGTACCGAGGTAAAGTACCTCGAACTCAAATCCAAATAAATGCTATAAAAGTTAACTTCAAAAAAAAAAATAAAGAAAAAATATCT

CCCCCTATAAATAACAACACACATAATATACTTATAAATAAAATTCTAGCATACTCAGCCAAAAAGATTAAAGCAAATCCCCCTCTTCTGTACTCTACAT

TAAACCCAGAAACTAACTCTGATTCTCCTTCTGCAAAATCAAAAGGAGTTCGATTAGTCTCAGCTAAACTTGAAGATAATCATATTATTCTTAAAGGTAA

GCACAGAAAAAAAAATCATACTATTTCTTGATATTTTATAAAATCTATTATATTTAAATTTAAAATTAACAATAAAAAAGATAATAAAATTAATGATAAA

CTTACTTCATATGAAATTGTTTGAGCTACTGATCGAATACCCCCTAATAAAGCATAATTAGAATTTGATGATCAACCAGAAATTATGATAGTATAGACCC

TTAGCCTAGAACAACATAAAAAATATAAAATACCTAAATTAAACCTAAATATAAATGTCAAAAAAGGTATACATATCCATAATAAAAGAGCTAAAAATAA

ATTAAAAACTGGAGATATATAATAAATAAAAAAATTTGATATTAAAGGATAAGTTTGTTCTTTAGAAAATAACTTAATAGCATCACTAAAAGGTTGAGGA

ATACCTATAAATCCAACTTTATTGGGCCCTTTACGAATTTGGATATACCCTAAAACTTTACGCTCTATTAATGTTAAGAAAGCTACACCAATTAAAACAC

AAATAATTAAAACTAAACTTGTAAATAATAAAAGAAATAAATCTTGTAATATAATGTATTACTTGTGTTAAACACATATTTAAATTCTAAATTTAAAGCA

CTAATCTGCCAAAGTAATATTCATATTCAAATTATATTAAATTTTAAAGGTATCTGATCCTTTCGTACTAAAATACCTATGTTTTTTAAAGATAGAAACC

AACCTGGCTCACGCCGGTTTAAACTCAGATCATGTAAAATTTTAAAGGTCGAACAGACCTAACCTTTTAGCCCCTACACCAAAAGTTAATTTTAATCCAA

CATCGAGGTCGCAAACTTTTTTTTCGATAAGAACTCTAAAAAAAAATTACGCTGTTATCCCTAAGGTAATTTAATCTTGTAATCATTAAAAATGGATCAT

TCAATCATAAATTAATGTTTTTAAATAAAAAAAGTTTAATCAATTTTTCTGCTGCCCCAGCAAAATAGTTTAAATTATTAAATATATAAATATACTAAAA

TTAAATAATAATTTAAACTATAAAACTCTATAGGGTCTTCTCGTCTTTTAAAATTATATAAGCTTTTTTACTTATAAATAAAATTCTATATTCAATTAAA

TTGAGACAGTTACTTTCTCGTCCAACCGTTCATTCCAGCTTTCAATTAAAAAACTAATGATTATGCTACCTTTGCACGGTCAAATTACCGCGGCCATTCA

AATCCTCATTGGGCAGGTCAGACTTTAAATTATAATCAAAAAGACATGTTTTTAATAAACAGGCGAAAAGTGTATTTGCCGAGTTCCTTAATTTAACCTT

GAAGTTTTAATTTAATTACTAAATTAAAATATATACTAATTTTATCATTATTCTATATAAACCAATATTACATATATTATCTTAATAAACTACTTAAAAA

TAATATAAATCTTATTCTAACAAAAATTATTTATAACAAACTAAAGATTAACACTTCCAATTCTACTAATTTTTATTCAAAATATACATTTTTAACATTT

TATTTTAAAGCTTATCCCCTAAAATATTACTTTTTATATATAAAATACTAAATAATTAATATAATACAATAAAAAAACTAAATTAAATTTATTTCTTAAG

AAACTAGATATCTTAAAAAACGTATAACGTTTCATTTCTAATATAATATTTTAAAAATTTATGCCACAATTAAATTTATATTATATTAGCTCTTTATAAT

TCGAGAACACTAAATAATTAAATTATTTTAATAAACCCTGATACACAAGGTACAAAAAATTAATTTTTCTTTTTAAAAAATAAATCTCTATATATTTATA

TTATCTATCTCTATACAAATTAACTATAATAAAATTTTTATATTCTAAAATATACTAATATCAAAAATATTTTTTTTATATATATATATATA----TTAT

AAATTTTTCCTTTCAAATTAAATTGATTTTCACAACTAACTTTTTAATGTAAATAAAATGCTTTATTACAAGCTCTAATTTGCCATTCCAGGTACACTTT

CCAGTACACCTACTATGTTACGACTTATCCCTCTTTAGAGAGGGAGCGACGGGCGATATGTACATATTCTAGAGCTATACTCATATAATTAAACTAAACT

ATATTACTTTCAAATCCACTTTATAAAATAATGTTAATTATTTTAACCATCTAAATAATTTTATTGTAACCCATCTCTCCTTATCTATACGCTGTATCTT

GATCTGATTTTTTTTATACTTATAAATTTTGAACATTCCAAATTCTTTAAAAACATTCAACCTACGACGATATACAAACCTTTAAAATAAGTACGATTAA

TCGTGGATCATCAATTATAGGACAGGTTCCTCTGAGTAGACTAAAATACCGCCAAATTCTTAAAATTTCAAGAACATAACTACTACTATTCAAGCATCTA

AAATTTGCATTTTTAATAATAGGGTATCTAATCCTAGTTTTTTATAAAAATCTCATAAACTCATTTTTCACATTTAAAAAATTAATTATACTTACTAATT

TCACCTAATAAATATAATATAAATTAATAATAAAATAACTTATTATATACTGAACAAATTTAATTGCATTGTTTGTGTAACCGCAACTGCTGGCACAAAC

TTGGTCAATACTATTATAAATTCCTAAATCAAAATTTCTTTTAAATTTAATCTTCACTATTGCAATTCTTTAATTAAATATATAGAATAATTTATCTTTT

TAAAATAAATTCATTAAACACTAAAATTTACATATAAAATAATTTAAAAATTAAAATCTCAAGCTAGAATAAAACTTTATTTTTTTTTCAATATACATAA

ATTATATAAAATAAAAGTACCCCCC-TACCATTTTAATTAAGTGAATTAATTTTTAATGCCTTAAATTTATTTCCAATTTATTTTTATTATATAGTTTTT

TATTTAAAAATAAAACTTTATACCTAAAATTATATACATAAATTATATTTAAAATTTATAATTTAAAGAACTATAAAAATTAAATTAAAATTTAGAATCT

ATCTAATTTATAGATAAACAAATTAAATTAAACTAACTTACCTGATAAATTTAACTATATACGCGCAGGTATATTAATTATTTAATTATAATATAATACA

TTTGTTTTATTAAATAATTATTAAGCAAAATAGCATTAATTTATTATATAAAAGATCTAGATACTATTAATTTAAAGTAAATTTTTTTTTTTTTTGTAGT

ATAATTTATACTAATATACGGCATTTAATATTTTCTATATAAATGTTTATATTATTTATATTTATCTATTTATAATTCTTAATAATAATTTAGATAATTA

TTAATTTATATATTAAATATTTATATATATATATATAATAATAAGGATATATATATATATATGTATATAGATAAATTAAATTTAGTTATATATAAAAAAA

TTAATATTAATTTTACTTTTAATTTTTTCTTTAATTTAAACTATTGGATATTCAGATTCTAATATATTATAATATATCAACCATTAATGAATAACTTTAT

ATATATAATAAAAATGTTAATTAATATATCAATTCATATGAATATATAATATTAATAAATAAATTCTTAATAAATAATAATGATAATATATATATATTGT

ATATTAATGTACTATATATATAAATTCATTAAACATTTATATTTGATACCCCCCCAAAATTTTTTCTAAAAATGTGAAGTTTCAATTTAATTAAAATTTT

CTACAATGGTCAAAAACGGCCAAAAAATGGAAAATAAAATGTGCACAGAAAATGCACATAAAATGCACATTTTTGAAAATTTCCGATTTTTGAAAAACCG

TTTGAACCAAACCTCTGATGTACCTTTTTTCGCCCCCCCTGATTACTAAAATTTATCCTCAATTACTAAGGTTTATCCGCATTTTTGCGACAGGAATTTT

GAGTATAAAATCAATTCCACACTAAATTTTTTTTT-TTTTTTTTGTGTCATTTCTCCAACATGGGCAATTTTTTTTTTAGTCGAAGGACAAATTTTAGTA

AAAAAAGTTTGAAATTAAGTTAAACCCTTGTTTAACCAAAAACTATTACTTTTCAATATTATACCCAATTTTATTTAAAATAAATTTTATTATACTTTTA

TTTATGCAAAAAGATCTTCTTATAAACTTTT

>DMR177j_JP_Tsuruoka_Honshu

AATGAAGTGTCTGACTATAGAGTTACTTTGATAGAGTAAAAAAAGTGAATTTTCACCTTCATTATAATTAACAGAATTAAACTATTTCTTTAAGCTTCAA

AAACTTATGTACATTATATACTAAATTATAAAAAGATAAGCTAATTAAGCTACTGGGCTCATACCCCATCAATAAAGGTTACAATCCTTTTCTTTTTAAT

GTATTATAAGCTTTTATTTTTTAATTCTCTTATAATTGGAACCTTAATTGCTATTTCTTCCTACTCTTGGATAGGAATATGAATAGGATTAGAAATTAAT

CTTCTCTCTATGATCCCCCTCATTAGAGACAACAAAAATATAATAGCCTCAGAAGCTGCTTTAAAATATTTCATTATTCAAACAATAGCATCAACATTGT

TATTATTCTCAATTATTATAATATCAATGAAATTTATATATCAAATAAATTTAATCACTTACTTTAATTTAATTTTTAACACTTCATTATTCATCAAAAT

AGGAGCAGCCCCATTCCATTTTTGATTCCCCGAAATAATAGAAGGATTAAATTGATTAAATGCCATTATCATACTTACTTGACAAAAACTAAGACCCATA

GTATTACTAACGTATTCTAATACAACCCCCATATATCTAATTTTAACAATTATATTTAGAATAATAATCAGAGGAATTATAGGTCTAAATCAAACTAGAT

TACGAAAAATTATAGCTTATTCATCTATCAACCATATTGGATGAATAATCAGTTCAATTATACTAATTGAAATTGTTTGATTTTACTATTTTATTATTTA

TTGCATTATTACTATTAATATCAGAATTATATTTATAAAATTAAATGTTTTCCATATCAATCAATTATATATTTCAATAAATTACCATATTTTACTTAAA

TTATTCTTTGCCTTAAATTTCATGTCTTTAGGAGGATTACCCCCATTTTTAGGGTTTTTCCCTAAATGACTTACAATTCAAACTTTAATCCAAAGAAATA

TATACTCAATTGCTTTCATTATAATTTTAATAACTTTAATAACACTTTTTTTTTACCTCCGAATTACTTTTTCAATTTTACTACTAAGAAAAACAGTTTT

AACATTTTACGCACAACCAAAAATTTATACTAATTACATTATAGCATTTAATTTTATTACATTATTAAGATTAATTTTCGTTACTTTGATATTCAATTTC

TTATAAATTAAACCTGAAGGATTTAAGTTAAATTAAACTAAGAACCTTCAAAGTTCTAAATAAAGTAAATTCTTTAAGCCTTAGGGCTTAGCCCATCTTT

AAATTTGCAATTTAAAATTCTTTTTGAACTATAAAGCTTGATAAAAGAAACTAATTTCGTATGTAAATTTACAGTTTACCGCCTAAACCTCGGCCATTTT

ATCGAATAAATGGCTATTCTCTACAAATCACAAAGATATCGGAACTTTATACTTTCTATTTGGAAGTTGAGCAGGCATAGTAGGCACTTCCTTAAGTTTA

TTGATTCGTGCTGAATTGGGAAACCCAGGATCTCTAATTGGAGATGATCAAATTTATAACGTAATCGTAACAGCTCATGCTTTCATTATAATTTTTTTTA

TAGTAATACCTATTATAATTGGGGGATTCGGAAACTGATTAGTTCCTTTAATGCTTGGAGCCCCCGACATAGCATTTCCCCGAATAAATAACATAAGATT

TTGACTTTTACCTCCTTCATTAACTCTACTTCTGATAAGAAGATTAGTCGAAAGAGGAGCAGGTACAGGCTGAACAGTTTATCCCCCACTATCAGCCAAT

ATCGCCCATAGAGGAGCATCAGTTGATTTAGCAATTTTTAGACTTCATCTTGCAGGAATTAGCTCAATTTTAGGTGCAGTAAATTTTATTACTACTGTAA

TTAACATACGATCAACAGGAATAACTTTTGATCGAATACCCCTATTTGTTTGATCTGTAGTTTTAACAGCACTTCTTCTGCTATTATCCCTCCCAGTTTT

AGCAGGAGCAATTACTATACTATTAACAGATCGAAATATTAACACAACATTTTTTGACCCCGCAGGTGGAGGAGACCCCATTCTCTACCAACATTTATTT

TGATTTTTTGGTCATCCAGAAGTTTACATTTTAATTTTACCTGGATTTGGTATAATCTCCCATATTATTAGCCAAGAAAGAAGAAAAAAAGAAACATTTG

GAACTTTAGGTATAATTTACGCTATAATAGCTATTGGTTTACTAGGATTTATTGTATGAGCTCATCATATATTTACAGTAGGTATAGATGTAGACACACG

GGCATATTTTACATCAGCAACAATAATCATTGCTGTTCCTACAGGAATTAAAATTTTTAGATGATTAGCTACTCTCCATGGATCACAATTAAACTATTCC

CCGTCTCTTTTATGGGCATTGGGATTTGTATTCCTATTTACAGTAGGAGGATTAACAGGGGTAATTCTAGCTAATTCATCAATTGACATTATTTTACATG

ATACTTACTATGTAGTTGCACATTTCCATTATGTCCTTTCCATAGGAGCTGTATTTGCTATTATAGCAGGTTTTGTTCATTGATTCCCTTTATTCACAGG

TTTAACAATAAATTCAAAATTTCTTAAAATTCAATTTTTAACAATATTTATTGGTGTTAATATCACATTCTTCCCCCAACATTTCTTAGGATTAAGAGGA

ATACCTCGACGTTATTCAGATTACCCAGATGCTTATACAACTTGAAATATTATCTCATCTATTGGATCTTTAGTTTCTTTAATTAGTATTTTTATCTTTT

TATTTACTATTTGAGAAAGGTTAATTTCATTACGAAAAAGAATTAGGTCTTTAAGAATATCTACATCAATTGAATGACTCCAACAAATACCCCCTTCAGA

ACATAGCTATTCTGAACTTCCAATGCTTACTAACTTCTAATATGGCAGATTAGTGCAATGGATTTAAACCCCAAATATAAAGATTAAACTTTTTTTAGAA

ATAGCTACTTGAAATACCATTTTACTTCAAGATAGGGCATCCCCATTAATAGAGCAACTCTCATTCTTTCATAACCATGCTCTTCTAATTCTCTTTATAA

TTACCGTTCTAGTAGGTTATTTAATAGGAACTTTATTTTTTAACCAATTTAATTACCGATTTTTATTAAATGGTCAAACTATTGAAATTATTTGAACTAT

TTTACCTGCTGTAACACTAATTTTTATCGCATTACCGTCTTTACGCTTACTTTATCTTCTAGATGAAGTTAATAACCCTTTAGTAACTATCAAAACAATT

GGGCATCAATGATATTGATCATACGAATATAGAGATTTTATAAATTTTGAATTCGATTCCTATATAATTCCTTTGACAGAAATAAAACCTCAAAATTTTC

GTTTATTAGATGTTGATAACCGAGTAATTGTCCCCTTTAACTCCCAAATCCGAATGGTAGTAACAGCTGCCGATGTTATTCATTCATGAACTATCCCAGC

TTTTAGTGTAAAAATTGATGCAACACCGGGCCGACTAAATCAAATTAGATTCCTAATTAATCGAACAGGATTATTTTATGGTCAATGCTCAGAAATTTGT

GGGGCAAATCATAGATTTATACCTATTACTGTAGAAAGAATTTCACCTTCATTTTTTACTAAATGAATCTCAAAAATAAATAACCTATCATTAGATGACT

GAAAGTAAGTAATGGTCTCTTAAACCAATTAATAGTAGTTTAACATCTACTTCTGATGGCCAAAAATTTAGTTAAGATATAACATTAGTTTGTCATACTA

AAATAATCATAATTTGATAATTTTTAATTCCACAAATAGCACCTTTAAACTGACTATCTTTATTTTTTTTAATTATTATTATTTTTTTACTTTTTAATGT

ATTAAATTACTTTAGATTCTTACAACCTTTAAAAACCCAATCTCATAACCCTACAATTAAAAAAATTAATTGAAAATGATAACTAATTTATTTTCATCTT

TTGATCCTAGAACTTCTTTTAATTTGAGATTAAACTGATTAAGAATACTATTAGGGCTAATATTTATCCCCCCAATATTTTGATTAGTTCCTTCACGCCA

TAATTTTCTATGAATTAAAATTATTTTAACATTACACCAAGAATTTAAGGTTTTAATTGGTAATAATAATATTAAAGGAAGAACCTTAATATTTATTTCA

TTATTTTCTATAATTGTTTTCAATAACTTTTTAGGATTATTTCCGTATATTTTTACAGGAACAAGACATTTAATTATAACATTATCTCTTGCCTTACCTT

TATGAATTAGATTCATATTATACGGGTGAATTAATAACACTATCCACATACTTGCTCATTTAGTTCCCCAAGGAACACCCCCAGCTCTTATGGCATTCAT

AGTAGTAATTGAATCAATTAGAAATATTATTCGTCCTGGTACTTTAGCTGTTCGATTAGCTGCTAATATAATTGCTGGACATTTACTAATAACTTTACTA

GGAAACACAGGATTAAATTTATCAATTTTTATATTAAGTATTCTTATTATCATACAAATTCTTTTATTAATTTTAGAATCTGCTGTTGCGATCATTCAAT

CTTATGTATTTGCTGTATTAAGAACTTTATACTCTAGAGAAATTAATTAATGTCAAGACATAAAAATCACCCTTATCATTTAGTTGATGCAAGGCCTTGA

CCTATTTTAGGTGCTTTTAGAGCTATAATTACAATAATTGGAATTATCAAATGATTCCATTTTTATAATAATTCTTTATTTTACTTAGGGACATTAATTA

CAATTTTAATTATAATTCAATGATGACGAGATATCACTCGTGAGGGAACTTTCCAAGGACTTCATACTTACGCTGTAACTATAGGTTTACGTTGAGGAAT

AATTTTATTTATTACATCAGAAGTATTTTTCTTTATTTCTTTTTTTTGAGCCTTTTTTCATAGTAGTTTAACACCCGCTATTGAACTAGGGATACTCTGA

CCACCTAAAGGAATTACTCCATTTAACCCTATTCAAATTCCATTATTAAACACTTTAATTCTTTTAACTTCGGGATTAACTGTAACTTGAGCTCACCATA

GATTAATAGAAAATGACTATAACCAAACAATACAAGGTCTTGGTTTAACAGTTTTACTAGGAATATATTTTACTTTATTACAAGGTTACGAATATTTAGA

AGCCCCCTTTACTATAGCAGATTCTGTTTATGGATCAACATTTTTTATTGCTACTGGTTTCCATGGATTACATGTTATTATTGGCACAACCTTTTTAGCT

GTTTGTTTAATACGGCATTTTAATAACCATTTTACTTGCATCCATCACTTTGGATTTGAAGCTGCTGCTTGATACTGACATTTTGTTGATGTAGTATGAC

TATTTCTTTATATTTCTATTTACTGATGAGGTAGATATTTATATAGTATAATAATTATAATTGATTTCCAATCAAAAGATCTAAAAAA--TTAGTATAAA

TAATCATTATAATTTGAAATATAGGGTTAATTATTTTTTCTATCTCTTTTATCTTAATTATACTATCTTTTACAATCTCTAAAAAAAGATTTATAGACCG

AGAAAAAGCTTCTCCATTCGAGTGTGGATTTGACCCCAAAAGATCAGCCCGTTTACCCTTTTCTTTGCATTTTTTTTTAATTGCAGTAATTTTCTTAATT

TTTGATGTTGAAATTACTCTTCTTATCCCTTTAATTCTAACAATAAAAATTACTAATATTACTATATATACCTATATTGCTCTTTTCTTTTTAATGATTC

TTTTAATAGGACTTTACCATGAATGAAACCAAGGAGCCTTAAATTGAGCTCTTTAGGGTAATAGTTAAGTATAACATTTAAGTTGCATTTAAAAAGTATT

GATTTTTCAATTTACCTTAAATAAGAAACAATTAATTGTATTTAGTTTCGACCTAAAATTTAGGTGTATGAACACCCTTATTTAAATTAATTGAAACCAA

AAAGAGGTATATCACTGTTAATGATACTAATGAGAAAAGCTCCAATTAAGGAAATAAGATATTCAAGAGTAAGCTTCTAACTTAACTCTTTAGCAGTGAA

AGTCTGTTAATATTTCTATTTATATAGTTTAATAAAACATTATTTTTTCATAATAAAATTAGAATAAATTTATTCTTATAAATATTTAAAAGTAAATTTT

ACTTCCCTGATAACTTCACTATCATACTCTCTATAAGCTATTTAAATTAAATATATAAAATTATAAAAATTACCCATATTATAATTAAAAGTATAAAAAT

CTTATAATTGTTATTAAATATAAACTGTAAAAATACAGAAGTATTACTAATTTTACTATATAAATTTTGTCTTCCATAATACTCTGATCACCCTTGATCA

ATAGTTTTATATAACTTACTACCTAATTTAATTGGATAATAATTTAAACCAAATGTTGAAATATAAGGTATATTCCACATAGAAGAAAAAAAAAGTCTTG

AATTTAACAAATAAAAAGATTTTAATCTATCATTTAAAGTAAACTTAGAAAGTTCAAACCCAAACCAAGCCCCAAAAAATGATACAATTAAAGCTATAAT

TTTTATTGTAAAAGGTAAACAAATAAAATAAGGTGTTGGGAATATCAGCCATATCAATATTCTTCCCCCAACAATAACTAAAAAAATTAAACCTGATATC

CCTTGAAGTATAATTTTTCTATTATCATTAATTTTACTTAAAGAATAAAAACAAAAATTTCCTACTAAAACATAATAAATTAAACGAAATGTATAACAAA

CAGTTAATCCTGTTGAAAAAAAGAAAATAACATAAATATAGATATTTAAATATCTTATAGATAAAACCTCTAAAATTAAATCCTTTGAATAAAACCCTGA

TAAAAAGGGTAAACCACATAATGCTAAATTTGAAATTATAAAATAAGTACAAGTTAAAGGTATAACTTTAATTAACCCCCCTATATACCGAATATCTTGA

CAATTTCTTAATCTGTGAATTATACACCCTGCGCATATAAATAATAAAGCCTTAAATAAAGCGTGAGTTAATAGATGAAAAAAAGCTAATTGATACTCCC

CTAAAGCTAAAATACTAATTATCAAACCCAATTGTCTTAAAGTAGATAGAGCAATAATTTTTTTTAAATCAAATTCAAAGTTTGCACCTATTCCTGCTAT

AAATATAGTTATTGTTCCAATAAATAATAAAATTAATATTAAATTACTTGTTAATGCAAAATTAAAACGAATTAATAAGTAAACACCTGCTGTTACTAAG

GTAGAAGAATGAACTAAAGAAGAAACAGGCGTTGGAGCTGCTATTGCCGCAGGCAACCAAGAAGAAAATGGAATTTGAGCTCTTTTAGTTATAGCTGCTA

ATATAATTAATAAAATAATAATATATATTTCTATGCTATTTTTATACACATCAATATAAAAAATATAGTTAAATCCCCCAAAATTTATTATTCATGCAAT

TGCTATTAATAAAGCAACATCTCCAATACGGTTAGTTAAAGCTGTAATTATCCCTGCATTATAAGATTTAATATTTTGATAATAAATAACTAAACAATAA

GAAACTAACCCTAACCCATCTCATCCTAATAAAATCCTAATTAAATTAGGAGAAATAATCAATAACATTATAGATAAAACAAATATAGAAACTAATATAA

TAAATCGATGCAAATAAATATCTCCTTCTATATACTCTTCACTATAGTAAATTACTATAGAAGAAATAAATAAAACAAAACTCATAAATAATAATGACAT

CCAGTCAAGTAAAATAGTTATAATAATTCTACAAGAATTAATCCTTAATATTTCATATTCTAATATTAGTCTATAATCTAAAATTATGAAATTTAAGCTT

AATAAAAATCTTAATACTCTAAAAAATAAAAACGTTACAAAATAAATTAAACAAATAGAAATAATTTAAAGTAAATTTTACATCTTTGATACCACAAATC

AATATTTTTTATTAAACTATTTAAATTACAATCATAAAACTAAAAATTCTCTCTTTAAAATTAAGATATTCAAGGGTAACCAATGAAGTAATAATAATAA

ATACTCACGAACAAACCCTCTTGAAAAAGAATATAAATTTCTAACTAACTTGCCGTGTTGACTATAAGAATATAAATATAAAGAATACGCAGCTCTAAAA

AAAGATATTAAAGATAAAAAAACCATTGTTCAACTCCTTCATCTAACTAATCTATTAATTAAAATAATCTCACCCAGTAAGTTTAATGAAGGAGGAGCCG

CTATATTACAACAACTAAATAAAAATCACCATATTCTTATTATTGGCATTAAATTGATTAGCCCCTTATTTAAATAAATACTTCGGCTATTTAGCCGTTC

ATAAGAAATATTTGCTAAACAAAATAATCCTGAAGAACATAAACCATGGGCAATTATTATAACTAAAGCCCCTCTTATACCCCAATAACTTAAAGTTAAA

ATCCCTCTTAATACTAAACCTATATGCGCTACAGAAGAATAAGCAATTAAAGCTTTAATATCAACTTGACGTAAACATATTAATGAAACAAAAAATCCCC

CTACTATTCTAATTGTAATAAAAATATAGTTTACTTGTAAACCTACAGTTAAAAAAATATTTATTAAACGTATTAATCCATACCCCCCTAATTTTAATAT

AACTCCAGCTAAAATTATAGAGCCAGCAACTGGAGCTTCAACATGGGCTTTAGGGAGCCAAAGATGAACAAAAAATATAGGCATTTTAATAAAAAACACT

ATATTTATACATATAAATAACAAGAATCTTTTTACATCATAACATAAAAAGAAAAAATCTAAAGAATGAAATTTTTCATAATAATAAAAAATCCTAATTA

TTATAGGTAATGAAGCAAATAATGTATAAAATAACAAGTAAACACCTGCTTGCAAACGCTCAGGTTGATACCCCCAACCAATAATTAATAGAAGAGTGGG

AATTAAGCTAAACTCAAAAAACAAATAAAAAATAAATAAATTTAAAGAACTAAATGTTATAACTAAAGATAATAATAAAATAATTATTACTAATAAAAAT

AAATTATAAAAATAATTTTTTCTATAAATACTTTCAGAAGCTAATAATATTAAAGAACAAATCCACAAACTTAATAAAATTATTATAAAAGATAAAAGGT

CATACCCTATAAAATAAGAAATATTTATATACAAATAATTAAAACTAAATCTTAACCCAAATATAAATGTAATAAAAAAATATATATATTGATTAAATCA

GTATCTCTTTTTAACACAACTTAAAGGTACCAATATTAATATTATAAAAATAAACTTTATCATAATACATTAAAAGTTTGAAAATAATCATTTCCATGTG

TTCGTATTATAGAAACTAATAACGATAAACCTAAAGCTCCTTCACAAACTCTTATAGTTAAAAATACTATACCAAAATAAAATTCAAAATTAAAATATAT

TAAAAATAGGTATAAATTAAAGTATAATCCTAAAATAATATACTCTAACCTTAATAATATTAAAAGTAAATGTTTACGTTTAATGCAAAAAGAAACTAAT

CCTGTAAAATATATAATCACTGAAAATAACATACAAAAAATTAACATTAGTTTTAATAATTTAATAAAAATACTGGTCTTGTAAATCAGAAATAAGAATT

TTCTTTTAAAACTTCAGAGAAAGAGTAAACCTCTATCATTAATCTCCAAAATTAATATTTTAAATAAACTATTCTCTGTATAATCTTATTACTAATAACA

TTATCTTTTATTAGTTCAATAACTTTTATATTTTTAAGTCACCCTTTATCTATAGGGCTTATTTTATTAATACAAACTATTATTATAGCTTTAACTATAG

GTTTTTTTAATATTAATTTCTGATATTCTTATATTTTATTTCTTATTATAATTGGAGGAATATTAGTTTTATTTATTTATATAACAAGAGTAGCTTCAAA

TGAAAAATTTTCGTTTTCTATTAAAATTACATTAATAATTAGAATTATAACTTTAGGATTTTTATTTAGTATTGCTATAATAGATCCCTACTTTTCAGAC

ATCAACTCAATTTACACAGAAAACTTAGATAATTATAAAGAATATAATATATCATTTAGAAAATATTTAAGTTATCCTAATATTATCATTATATACATAA

TAATTATTTATTTATTAATTACCTTAATTGCAGTTGTAAAAATTACTCAAATTGAAAAAGGACCTTTACGTCAAACTAACTAATGAAAACACCATTACGA

AAAGCTTCTCCCCTATTAAAAATTATTAATAATAGAATTATTGATTTACCTACACCATCTAATATTTCTGCTTGATGAAATTTTGGATCCTTATTAGGAC

TCTGTCTTTTTATTCAAATTATCACTGGAATTTTCTTAGCTATACATTTTACAGCCCATATTGACATAGCATTTAATAGAGTAATTCATATTTGTCGAGA

TGTAAATTATGGCTGATTATTACGAACAATTCATGCTAACGGAGCTTCTTTTTTCTTTATTTGTATTTATCTACATATTGGCCGAGGAATATATTATAGA

AGCTATAATTTACATTTAACATGAACTATTGGAGTAATCATCCTTTTTATAGTAATAGCTACAGCATTCTTAGGGTATGTATTACCTTGAGGGCAAATAT

CTTTTTGAGGGGCTACTGTTATTACCAATCTTCTCTCTGCTATTCCTTATCTAGGAAATATGATTGTCCAATGATTATGAGGGGGATTTGCAGTAGATAA

CGCTACTTTAACTCGATTTTTTACATTACATTTTATTCTTCCTTTTATTATTTTAGCTTTAATAATTATTCATTTATTATTTCTTCATCAAACTGGTTCT

AATAACCCTTTAGGATTAAATAGAAATATCGATAAAGTACCTTTCCATCCATATTTTACTTACAAAGATACTTTTGGATTCATTATAATAACTATATTAT

TAATATTTTTAGTTTTAATTAATCCCTATCTTTTAGGAGACCCAGAAAACTTTACACCTGCTAATCCTTTAGTAACCCCAGTCCATATCCAACCAGAATG

ATACTTTTTATTTGCATACGCTATTTTACGATCAATTCCTAATAAATTAGGTGGAGTAATTGCTTTAGTTATATCAATTGCTATTTTATTAATTATACCT

TTAATTAACAAAAAAAAATTTAGTAGAACTCAATTTTACCCATTAAATAAAATTTTATTTTGATCTTTTGTCTCTATTGTAATCTTATTAACATGAATCG

GAGCTCGCCCAGTGGAAGATCCATACATTTTAACAGGTCAAATTTTAACAATTATATATTTTAGTTATTATTTCTTAAACCCCTTAATTCATAAAATATG

AGATTATATTATTTTTAAAACTTAGTTAATGAACTTGTTAAAGTGTATATTTTGAAAATATAAAAAAGAGTTTATTCTCTATTAACTTTACTAAATTTTA

TTCACTAAATAAAATAAGTAAAGATAACCAACTTTAAACCTAAAAAAAAAAATAAAAAATTTAAAGAAACTGGTAAATAACTTTTTCAACATATATATAT

TAACTTATCATAACGGTACCGAGGTAAAGTACCTCGAACTCAAATCCAAATAAATGCTATAAAAGTTAACTTCAAAAAAAAAAATAAAGAAAAAATATCT

CCCCCTATAAATAACAACACACATAATATACTTATAAATAAAATTCTAGCATACTCAGCCAAAAAGATTAAAGCAAATCCCCCTCTTCTGTACTCTACAT

TAAACCCAGAAACTAACTCTGATTCTCCTTCTGCAAAATCAAAAGGAGTTCGATTAGTCTCAGCTAAACTTGAAGATAATCATATTATTCTTAAAGGTAA

GCACAGAAAAAAAAATCATACTATTTCTTGATATTTTATAAAATCTATTATATTTAAATTTAAAATTAACAATAAAAAAGATAATAAAATTAATGATAAA

CTTACTTCATATGAAATTGTTTGAGCTACTGATCGAATACCCCCTAATAAAGCATAATTAGAATTTGATGATCAACCAGAAATTATGATAGTATAGACCC

TTAGCCTAGAACAACATAAAAAATATAAAATACCTAAATTAAACCTAAATATAAATGTCAAAAAAGGTATACATATCCATAATAAAAGAGCTAAAAATAA

ATTAAAAACTGGAGATATATAATAAATAAAAAAATTTGATATTAAAGGATAAGTTTGTTCTTTAGAAAATAACTTAATAGCATCACTAAAAGGTTGAGGA

ATACCTATAAATCCAACTTTATTGGGCCCTTTACGAATTTGGATATACCCTAAAACTTTACGCTCTATTAATGTTAAGAAAGCTACACCAATTAAAACAC

AAATAATTAAAACTAAACTTGTAAATAATAAAAGAAATAAATCTTGTAATATAATGTATTACTTGTGTTAAACACATATTTAAATTCTAAATTTAAAGCA

CTAATCTGCCAAAGTAATATTCATATTCAAATTATATTAAATTTTAAAGGTATCTGATCCTTTCGTACTAAAATACCTATGTTTTTTAAAGATAGAAACC

AACCTGGCTCACGCCGGTTTAAACTCAGATCATGTAAAATTTTAAAGGTCGAACAGACCTAACCTTTTAGCCCCTACACCAAAAGTTAATTTTAATCCAA

CATCGAGGTCGCAAACTTTTTTTTCGATAAGAACTCTAAAAAAAAATTACGCTGTTATCCCTAAGGTAATTTAATCTTGTAATCATTAAAAATGGATCAT

TCAATCATAAATTAATGTTTTTAAATAAAAAAAGTTTAATCAATTTTTCTGCTGCCCCAGCAAAATAGTTTAAATTATTAAATATATAAATATACTAAAA

TTAAATAATAATTTAAACTATAAAACTCTATAGGGTCTTCTCGTCTTTTAAAATTATATAAGCTTTTTTACTTATAAATAAAATTCTATATTCAATTAAA

TTGAGACAGTTACTTTCTCGTCCAACCGTTCATTCCAGCTTTCAATTAAAAAACTAATGATTATGCTACCTTTGCACGGTCAAATTACCGCGGCCATTCA

AATCCTCATTGGGCAGGTCAGACTTTAAATTATAATCAAAAAGACATGTTTTTAATAAACAGGCGAAAAGTGTATTTGCCGAGTTCCTTAATTTAACCTT

GAAGTTTTAATTTAATTACTAAATTAAAATATATACTAATTTTATCATTATTCTATATAAACCAATATTACATATATTATCTTAATAAACTACTTAAAAA

TAATATAAATCTTATTCTAACAAAAATTATTTATAACAAACTAAAGATTAACACTTCCAATTCTACTAATTTTTATTCAAAATATACATTTTTAACATTT

TATTTTAAAGCTTATCCCCTAAAATATTACTTTTTATATATAAAATACTAAATAATTAATATAATACAATAAAAAAACTAAATTAAATTTATTTCTTAAG

AAACTAGATATCTTAAAAAACGTATAACGTTTCATTTCTAATATAATATTTTAAAAATTTATGCCACAATTAAATTTATATTATATTAGCTCTTTATAAT

TCGAGAACACTAAATAATTAAATTATTTTAATAAACCCTGATACACAAGGTACAAAAAATTAATTTTTCTTTTTAAAAAATAAATCTCTATATATTTATA

TTATCTATCTCTATACAAATTAACTATAATAAAATTTTTATATTCTAAAATATACTAATATCAAAAATATTTTTTTTATATATATATATATA----TTAT

AAATTTTTCCTTTCAAATTAAATTGATTTTCACAACTAACTTTTTAATGTAAATAAAATGCTTTATTACAAGCTCTAATTTGCCATTCCAGGTACACTTT

CCAGTACACCTACTATGTTACGACTTATCCCTCCTTAGAGAGGGAGCGACGGGCGATATGTACATATTCTAGAGCTATACTCATATAATTAAACTAAACT

ATATTACTTTCAAATCCACTTTATAAAATAATGTTAATTATTTTAACCATCTAAATAATTTTATTGTAACCCATCTCTCCTTATCTATACGCTGTATCTT

GATCTGATTTTTTTTATACTTATAAATTTTGAACATTCCAAATTCTTTAAAAACATTCAACCTACGACGATATACAAACCTTTAAAATAAGTACGATTAA

TCGTGGATCATCAATTATAGGACAGGTTCCTCTGAGTAGACTAAAATACCGCCAAATTCTTAAAATTTCAAGAACATAACTACTACTATTCAAGCATCTA

AAATTTGCATTTTTAATAATAGGGTATCTAATCCTAGTTTTTTATAAAAATCTCATAAACTCATTTTTCACATTTAAAAAATTAATTATACTTACTAATT

TCACCTAATAAATATAATATAAATTAATAATAAAATAACTTATTATATACTGAACAAATTTAATTGCATTGTTTGTGTAACCGCAACTGCTGGCACAAAC

TTGGTCAATACTATTATAAATTCCTAAATCAAAATTTCTTTTAAATTTAATCTTCACTATTGCAATTCTTTAATTAAATATATAGAATAATTTATCTTTT

TAAAATAAATTCATTAAACACTAAAATTTACATATAAAATAATTTAAAAATTAAAATCTCAAGCTAGAATAAAACTTTATTTTTTTTTCAATATACATAA

ATTATATAAAATAAAAGTACCCCCC-TACCATTTTAATTAAGTGAATTAATTTTTAATGCCTTAAATTTATTTCCAATTTATTTTTATTATATAGTTTTT

TATTTAAAAATAAAACTTTATACCTAAAATTATATACATAAATTATATTTAAAATTTATAATTTAAAGAACTATAAAAATTAAATTAAAATTTAGAATCT

ATCTAATTTATAGATAAACAAATTAAATTAAACTAACTTACCTGATAAATTTAACTATATACGCGCAGGTATATTAATTATTTAATTATAATATAATACA

TTTGTTTTATTAAATAATTATTAAGCAAAATAGCATTAATTTATTATATAAAAGATCTAGATACTATTAATTTAAAGTAAATTTTTTTTTTTTTTGTAGT

ATAATTTATACTAATATACGGCATTTAATATTTTCTATATAAATGTTTATATTATTTATATTTATCTATTTATAATTCTTAATAATAATTTAGATAATTA

TTAATTTATATATTAAATATTTATATATATATATATAATAATAAGGATATATATATATATATGTATATAGATAAATTAAATTTAGTTATATATAAAAAAA

TTAATATTAATTTTACTTTTAATTTTTTCTTTAATTTAAACTATTGGATATTCAGATTCTAATATATTATAATATATCAACCATTAATGAATAACTTTAT

ATATATAATAAAAATGTTAATTAATATATCAATTCATATGAATATATAATATTAATAAATAAATTCTTAATAAATAATAATGATAATATATATATATTGT

ATATTAATGTACTATATATATAAATTCATTAAACATTTATATTTGATACCCCCCCAAAATTTTTTCTAAAAATGTGAAGTTTCAATTTAATTAAAATTTT

CTACAATGGTCAAAAACGGCCAAAAAATGGAAAATAAAATGTGCACAGAAAATGCACATAAAATGCACATTTTTGAAAATTTCCGATTTTTGAAAAACCG

TTTGAACCAAACCTCTGATGTACCTTTTTTCGCCCCCCCTGATTACTAAAATTTATCCTCAATTACTAAGGTTTATCCGCATTTTTGCGACAGGAATTTT

GAGTATAAAATCAATTCCACACTAAATTTTTTTTT-TTTTTTTTGTGTCATTTCTCCAACATGGGCAATTTTTTTTTTAGTCGAAGGACAAATTTTAGTA

AAAAAAGTTTGAAATTAAGTTAAACCCTTGTTTAACCAAAAACTATTACTTTTCAATATTATACCCAATTTTATTTAAAATAAATTTTATTATACTTTTA

TTTATGCAAAAAGATCTTCTTATAAACTTTT

>DMR178j_JP_Tsuruoka_Honshu

AATGAAGTGTCTGACTATAGAGTTACTTTGATAGAGTAAAAAAAGTGAATTTTCACCTTCATTATAATTAACAGAATTAAACTATTTCTTTAAGCTTCAA

AAACTTATGTACATTATATACTAAATTATAAAAAGATAAGCTAATTAAGCTACTGGGCTCATACCCCATCAATAAAGGTTACAATCCTTTTCTTTTTAAT

GTATTATAAGCTTTTATTTTTTAATTCTCTTATAATTGGAACCTTAATTGCTATTTCTTCCTACTCTTGGATAGGAATATGAATAGGATTAGAAATTAAT

CTTCTCTCTATGATCCCCCTCATTAGAGACAACAAAAATATAATAGCCTCAGAAGCTGCTTTAAAATATTTCATTATTCAAACAATAGCATCAACATTGT

TATTATTCTCAATTATTATAATATCAATGAAATTTATATATCAAATAAATTTAATCACTTACTTTAATTTAATTTTTAACACTTCATTATTCATCAAAAT

AGGAGCAGCCCCATTCCATTTTTGATTCCCCGAAATAATAGAAGGATTAAATTGATTAAATGCCATTATCATACTTACTTGACAAAAACTAAGACCCATA

GTATTACTAACGTATTCTAATACAACCCCCATATATCTAATTTTAACAATTATATTTAGAATAATAATCAGAGGAATTATAGGTCTAAATCAAACTAGAT

TACGAAAAATTATAGCTTATTCATCTATCAACCATATTGGATGAATAATCAGTTCAATTATACTAATTGAAATTGTTTGATTTTACTATTTTATTATTTA

TTGCATTATTACTATTAATATCAGAATTATATTTATAAAATTAAATGTTTTCCATATCAATCAATTATATATTTCAATAAATTACCATATTTTACTTAAA

TTATTCTTTGCCTTAAATTTCATGTCTTTAGGAGGATTACCCCCATTTTTAGGGTTTTTCCCTAAATGACTTACAATTCAAACTTTAATCCAAAGAAATA

TATACTCAATTGCTTTCATTATAATTTTAATAACTTTAATAACACTTTTTTTTTACCTCCGAATTACTTTTTCAATTTTACTACTAAGAAAAACAGTTTT

AACATTTTACGCACAACCAAAAATTTATACTAATTACATTATAGCATTTAATTTTATTACATTATTAAGATTAATTTTCGTTACTTTGATATTCAATTTC

TTATAAATTAAACCTGAAGGATTTAAGTTAAATTAAACTAAGAACCTTCAAAGTTCTAAATAAAGTAAATTCTTTAAGCCTTAGGGCTTAGCCCATCTTT

AAATTTGCAATTTAAAATTCTTTTTGAACTATAAAGCTTGATAAAAGAAACTAATTTCGTATGTAAATTTACAGTTTACCGCCTAAACCTCGGCCATTTT

ATCGAATAAATGGCTATTCTCTACAAATCACAAAGATATCGGAACTTTATACTTTCTATTTGGAAGTTGAGCAGGCATAGTAGGCACTTCCTTAAGTTTA

TTGATTCGTGCTGAATTGGGAAACCCAGGATCTCTAATTGGAGATGATCAAATTTATAACGTAATCGTAACAGCTCATGCTTTCATTATAATTTTTTTTA

TAGTAATACCTATTATAATTGGGGGATTCGGAAACTGATTAGTTCCTTTAATGCTTGGAGCCCCCGACATAGCATTTCCCCGAATAAATAACATAAGATT

TTGACTTTTACCTCCTTCATTAACTCTACTTCTAATAAGAAGATTAGTCGAAAGAGGAGCAGGTACAGGCTGAACAGTTTATCCCCCACTATCAGCCAAT

ATCGCCCATAGAGGAGCATCAGTTGATTTAGCAATTTTTAGACTTCATCTTGCAGGAATTAGCTCAATTTTAGGTGCAGTAAATTTTATTACTACTGTAA

TTAACATACGATCAACAGGAATAACTTTTGATCGAATACCCCTATTTGTTTGATCTGTAGTTTTAACAGCACTTCTTCTGCTATTATCCCTCCCAGTTTT

AGCAGGAGCAATTACTATACTATTAACAGATCGAAATATTAACACAACATTTTTTGACCCCGCAGGTGGAGGAGACCCCATTCTCTACCAACATTTATTT

TGATTTTTTGGTCATCCAGAAGTTTACATTTTAATTTTACCTGGATTTGGTATAATCTCCCATATTATTAGCCAAGAAAGAAGAAAAAAAGAAACATTTG

GAACTTTAGGTATAATTTACGCTATAATAGCTATTGGTTTACTAGGATTTATTGTATGAGCTCATCATATATTTACAGTAGGTATAGATGTAGACACACG

GGCATATTTTACATCAGCAACAATAATCATTGCTGTTCCTACAGGAATTAAAATTTTTAGATGATTAGCTACTCTCCATGGATCACAATTAAACTATTCC

CCGTCTCTTTTATGGGCATTGGGATTTGTATTCCTATTTACAGTAGGAGGATTAACAGGGGTAATTCTAGCTAATTCATCAATTGACATTATTTTACATG

ATACTTACTATGTAGTTGCACATTTCCATTATGTCCTTTCCATAGGAGCTGTATTTGCTATTATAGCAGGTTTTGTTCATTGATTCCCTTTATTCACAGG

TTTAACAATAAATTCAAAATTTCTTAAAATTCAATTTTTAACAATATTTATTGGTGTTAATATCACATTCTTCCCCCAACATTTCTTAGGATTAAGAGGA

ATACCTCGACGTTATTCAGATTACCCAGATGCTTATACAACTTGAAATATTATCTCATCTATTGGATCTTTAGTTTCTTTAATTAGTATTTTTATCTTTT

TATTTACTATTTGAGAAAGGTTAATTTCATTACGAAAAAGAATTAGGTCTTTAAGAATATCTACATCAATTGAATGACTCCAACAAATACCCCCTTCAGA

ACATAGCTATTCTGAACTTCCAATGCTTACTAACTTCTAATATGGCAGATTAGTGCAATGGATTTAAACCCCAAATATAAAGATTAAACTTTTTTTAGAA

ATAGCTACTTGAAATACCATTTTACTTCAAGATAGGGCATCCCCATTAATAGAGCAACTCTCATTCTTTCATAACCATGCTCTTTTAATTCTCTTTATAA

TTACCGTTCTAGTAGGTTATTTAATAGGAACTTTATTTTTTAACCAATTTAATTACCGATTTTTATTAAATGGTCAAACTATTGAAATTATTTGAACTAT

TTTACCTGCTGTAACACTAATTTTTATCGCATTACCGTCTTTACGCTTACTTTATCTTCTAGATGAAGTTAATAACCCTTTAGTAACTATCAAAACAATT

GGGCATCAATGATATTGATCATACGAATATAGAGATTTTATAAATTTTGAATTCGATTCCTATATAATTCCTTTGACAGAAATAAAACCTCAAAATTTTC

GTTTATTAGATGTTGATAACCGAGTAATTGTCCCCTTTAACTCCCAAATCCGAATGATAGTAACAGCTGCCGATGTTATTCATTCATGAACTATCCCAGC

TTTTAGTGTAAAAATTGATGCAACACCGGGCCGACTAAATCAAATTAGATTCCTAATTAATCGAACAGGATTATTTTATGGTCAATGCTCAGAAATTTGT

GGAGCAAATCATAGATTTATACCTATTACTGTAGAAAGAATTTCACCTTCATTTTTTACTAAATGAATCTCAAAAATAAATAACCTATCATTAGATGACT

GAAAGTAAGTAATGGTCTCTTAAACCAATTAATAGTAGTTTAACATCTACTTCTGATGGCCAAAAATTTAGTTAAGATATAACATTAGTTTGTCATACTA

AAATAATCATAATTTGATAATTTTTAATTCCACAAATAGCACCTTTAAACTGACTATCTTTATTTTTTTTAATTATTATTATTTTTTTACTTTTTAATGT

ATTAAATTACTTTAGATTCTTACAACCTTTAAAAACCCAATCTCATAACCCTACAATTAAAAAAATTAATTGAAAATGATAACTAATTTATTTTCATCTT

TTGATCCTAGAACTTCTTTTAATTTGAGATTAAACTGATTAAGAATACTATTAGGGCTAATATTTATCCCCCCAATATTTTGATTAGTTCCTTCACGCCA

TAATTTTCTATGAATTAAAATTATTTTAACATTACACCAAGAATTTAAGGTTTTAATTGGTAATAATAATATTAAAGGAAGAACCTTAATATTTATTTCA

TTATTTTCTATAATTGTTTTCAATAACTTTTTAGGATTATTTCCGTATATTTTTACAGGAACAAGACATTTAATTATAACATTATCTCTTGCCTTACCTT

TATGAATTAGATTCATATTATACGGGTGAATTAATAACACTATCCACATACTTGCTCATTTAGTTCCCCAAGGAACACCCCCAGCTCTTATGGCATTCAT

AGTAGTAATTGAATCAATTAGAAATATTATTCGTCCTGGTACTTTAGCTGTTCGATTAGCTGCTAATATAATTGCTGGACATTTACTAATAACTTTACTA

GGAAACACAGGATTAAATTTATCAATTTTTATATTAAGTATTCTTATTATCATACAAATTCTTTTATTAATTTTAGAATCTGCTGTTGCGATCATTCAAT

CTTATGTATTTGCTGTATTAAGAACTTTATACTCTAGAGAAATTAATTAATGTCAAGACATAAAAATCACCCTTATCATTTAGTTGATGCAAGGCCTTGA

CCTATTTTAGGTGCTTTTAGAGCTATAATTACAATAATTGGAATTATCAAATGATTCCATTTTTATAATAATTCTTTATTTTACTTAGGGACATTAATTA

CAATTTTAATTATAATTCAATGATGACGAGATATCACTCGTGAGGGAACTTTCCAAGGACTTCATACTTACGCTGTAACTATAGGTTTACGTTGAGGAAT

AATTTTATTTATTACATCAGAAGTATTTTTCTTTATTTCTTTTTTTTGAGCCTTTTTTCATAGTAGTTTAACACCCGCTATTGAACTAGGGATACTCTGA

CCACCTAAAGGAATTACTCCATTTAACCCTATTCAAATTCCATTATTAAACACTTTAATTCTTTTAACTTCAGGATTAACTGTAACTTGAGCTCACCATA

GATTAATAGAAAATGACTATAACCAAACAATACAAGGTCTTGGTTTAACAGTTTTACTAGGAATATATTTTACTTTATTACAAGGTTACGAATATTTAGA

AGCCCCCTTTACTATAGCAGATTCTGTTTATGGATCAACATTTTTTATTGCTACTGGTTTCCATGGATTACATGTTATTATTGGCACAACCTTTTTAGCT

GTTTGTTTAATACGGCATTTTAATAACCATTTTACTTGCATCCATCACTTTGGATTTGAAGCTGCTGCTTGATACTGACATTTTGTTGATGTAGTATGAC

TATTTCTTTATATTTCTATTTACTGATGAGGTAGATATTTATATAGTATAATAATTATAATTGATTTCCAATCAAAAGATCTAAAAAA--TTAGTATAAA

TAATCATTATAATTTGAAATATAGGGTTAATTATTTTTTCTATCTCTTTTATCTTAATTATACTATCTTTTACAATCTCTAAAAAAAGATTTATAGACCG

AGAAAAAGCTTCTCCATTCGAGTGTGGATTTGACCCCAAAAGATCAGCCCGTTTACCCTTTTCTTTGCATTTTTTTTTAATTGCAGTAATTTTCTTAATT

TTTGATGTTGAAATTACTCTTCTTATCCCTTTAATTCTAACAATAAAAATTACTAATATTACTATATATACCTATATTGCTCTTTTCTTTTTAATGATTC

TTTTAATAGGACTTTACCATGAATGAAACCAAGGAGCCTTAAATTGAGCTCTTTAGGGTAATAGTTAAGTATAACATTTAAGTTGCATTTAAAAAGTATT

GATTTTTCAATTTACCTTAAATAAGAAACAATTAATTGTATTTAGTTTCGACCTAAAATTTAGGTGTATGAACACCCTTATTTAAATTAATTGAAACCAA

AAAGAGGTATATCACTGTTAATGATACTAATGAGAAAAGCTCCAATTAAGGAAATAAGATATTCAAGAGTAAGCTTCTAACTTAACTCTTTAGCAGTGAA

AGTCTGTTAATATTTCTATTTATATAGTTTAATAAAACATTATTTTTTCATAATAAAATTAGAATAAATTTATTCTTATAAATATTTAAAAGTAAATTTT

ACTTCCCTGATAACTTCACTATCATACTCTCTATAAGCTATTTAAATTAAATATATAAAATTATAAAAATTACCCATATTATAATTAAAAGTATAAAAAT

CTTATAATTGTTATTAAATATAAACTGTAAAAATACAGAAGTATTACTAATTTTACTATATAAATTTTGTCTTCCATAATACTCTGATCACCCTTGATCA

ATAGTTTTATATAACTTACTACCTAATTTAATTGGATAATAATTTAAACCAAATGTTGAAATATAAGGTATATTCCACATAGAAGAAAAAAAAAGTCTTG

AATTTAACAAATAAAAAGATTTTAATCTATCATTTAAAGTAAACTTAGAAAGTTCAAACCCAAACCAAGCCCCAAAAAATGATACAATTAAAGCTATAAT

TTTTATTGTAAAAGGTAAACAAATAAAATAAGGTGTTGGGAATATCAGCCATATCAATATTCTTCCCCCAACAATAACTAAAAAAATTAAACCTGATATC

CCTTGAAGTATAATTTTTCTATTATCATTAATTTTACTTAAAGAATAAAAACAAAAATTTCCTACTAAAACATAATAAATTAAACGAAATGTATAACAAA

CAGTTAATCCTGTTGAAAAAAAGAAAATAACATAAATATAGATATTTAAATATCTTATAGATAAAACCTCTAAAATTAAATCCTTTGAATAAAACCCTGA

TAAAAAGGGTAAACCACATAATGCTAAATTTGAAATTATAAAATAAGTACAAGTTAAAGGTATAACTTTAATTAACCCCCCTATATACCGAATATCTTGA

CAATTTCTTAATCTGTGAATTATACACCCTGCGCATATAAATAATAAAGCCTTAAATAAAGCGTGAGTTAATAGATGAAAAAAAGCTAATTGATACTCCC

CTAAAGCTAAAATACTAATTATCAAACCCAATTGTCTTAAAGTAGATAGAGCAATAATTTTTTTTAAATCAAATTCAAAGTTTGCACCTATTCCTGCTAT

AAATATAGTTATTGTTCCAATAAATAATAAAATTAATATTAAATTACTTGTTAATGCAAAATTAAAACGAATTAATAAGTAAACACCTGCTGTTACTAAG

GTAGAAGAATGAACTAAAGAAGAAACAGGCGTTGGAGCTGCTATTGCCGCAGGCAACCAAGAAGAAAATGGAATTTGAGCTCTTTTAGTTATAGCTGCTA

ATATAATTAATAAAATAATAATATATATTTCTATGCTATTTTTATACACATCAATATAAAAAATATAGTTAAATCCCCCAAAATTTATTATTCATGCAAT

TGCTATTAATAAAGCAACATCTCCAATACGGTTAGTTAAAGCTGTAATTATCCCTGCATTATAAGATTTAATATTTTGATAATAAATAACTAAACAATAA

GAAACTAACCCTAACCCATCTCATCCTAATAAAATCCTAATTAAATTAGGAGAAATAATCAATAACATTATAGATAAAACAAATATAGAAACTAATATAA

TAAATCGATGCAAATAAATATCTCCTTCTATATACTCTTCACTATAGTAAATTACTATAGAAGAAATAAATAAAACAAAACTCATAAATAATAATGACAT

CCAGTCAAGTAAAATAGTTATAATAATTCTACAAGAATTAATCCTTAATATTTCATATTCTAATATTAGTCTATAATCTAAAATTATGAAATTTAAGCTT

AATAAAAATCTTAATACTCTAAAAAATAAAAACGTTACAAAATAAATTAAACAAATAGAAATAATTTAAAGTAAATTTTACATCTTTGATACCACAAATC

AATATTTTTTATTAAACTATTTAAATTACAATCATAAAACTAAAAATTCTCTCTTTAAAATTAAGATATTCAAGGGTAACCAATGAAGTAATAATAATAA

ATACTCACGAACAAACCCTCTTGAAAAAGAATATAAATTTCTAACTAACTTGCCGTGTTGACTATAAGAATATAAATATAAAGAATACGCAGCTCTAAAA

AAAGATATTAAAGATAAAAAAACCATTGTTCAACTCCTTCATCTAACTAATCTATTAATTAAAATAATCTCACCCAGTAAGTTTAATGAAGGAGGAGCCG

CTATATTACAACAACTAAATAAAAATCACCATATTCTTATTATTGGCATTAAATTGATTAGCCCCTTATTTAAATAAATACTTCGGCTATTTAGCCGTTC

ATAAGAAATATTTGCTAAACAAAATAATCCTGAAGAACATAAACCATGGGCAATTATTATAACTAAAGCCCCTCTTATACCCCAATAACTTAAAGTTAAA

ATCCCTCTTAATACTAAACCTATATGCGCTACAGAAGAATAAGCAATTAAAGCTTTAATATCAACTTGACGTAAACATATTAATGAAACAAAAAATCCCC

CTACTATTCTAATTGTAATAAAAATATAGTTTACTTGTAAACCTACAGTTAAAAAAATATTTATTAAACGTATTAATCCATACCCCCCTAATTTTAATAT

AACTCCAGCTAAAATTATAGAGCCAGCAACTGGAGCTTCAACATGGGCTTTAGGGAGCCAAAGATGAACAAAAAATATAGGCATTTTAATAAAAAACACT

ATATTTATACATATAAATAACAAGAATCTTTTTACATCATAACATAAAAAGAAAAAATCTAAAGAATGAAATTTTTCATAATAATAAAAAATCCTAATTA

TTATAGGTAATGAAGCAAATAATGTATAAAATAACAAGTAAACACCTGCTTGCAAACGCTCAGGTTGATACCCCCAACCAATAATTAATAGAAGAGTGGG

AATTAAGCTAAACTCAAAAAACAAATAAAAAATAAATAAATTTAAAGAACTAAATGTTATAACTAAAGATAATAATAAAATAATTATTACTAATAAAAAT

AAATTATAAAAATAATTTTTTCTATAAATACTTTCAGAAGCTAATAATATTAAAGAACAAATCCACAAACTTAATAAAATTATTATAAAAGATAAAAGGT

CATACCCTATAAAATAAGAAATATTTATATACAAATAATTAAAACTAAATCTTAACCCAAATATAAATGTAATAAAAAAATATATATATTGATTAAATCA

GTATCTCTTTTTAACACAACTTAAAGGTACCAATATTAATATTATAAAAATAAACTTTATCATAATACATTAAAAGTTTGAAAATAATCATTTCCATGTG

TTCGTATTATAGAAACTAATAACGATAAACCTAAAGCTCCTTCACAAACTCTTATAGTTAAAAATACTATACCAAAATAAAATTCAAAATTAAAATATAT

TAAAAATAGGTATAAATTAAAGTATAATCCTAAAATAATATACTCTAACCTTAATAATATTAAAAGTAAATGTTTACGTTTAATGCAAAAAGAAACTAAT

CCTGTAAAATATATAATCACTGAAAATAACATACAAAAAATTAACATTAGTTTTAATAATTTAATAAAAATACTGGTCTTGTAAATCAGAAATAAGAATT

TTCTTTTAAAACTTCAGAGAAAGAGTAAACCTCTATCATTAATCTCCAAAATTAATATTTTAAATAAACTATTCTCTGTATAATCTTATTACTAATAACA

TTATCTTTTATTAGTTCAATAACTTTTATATTTTTAAGTCACCCTTTATCTATAGGGCTTATTTTATTAATACAAACTATTATTATAGCTTTAACTATAG

GTTTTTTTAATATTAATTTCTGATATTCTTATATTTTATTTCTTATTATAATTGGAGGAATATTAGTTTTATTTATTTATATAACAAGAGTAGCTTCAAA

TGAAAAATTTTCGTTTTCTATTAAAATTACATTAATAATTAGAATTATAACTTTAGGATTTTTATTTAGTATTGCTATAATAGATCCCTACTTTTCAGAC

ATCAACTCAATTTACACAGAAAACTTAGATAATTATAAAGAATATAATATATCATTTAGAAAATATTTAAGTTATCCTAATATTATCATTATATACATAA

TAATTATTTATTTATTAATTACCTTAATTGCAGTTGTAAAAATTACTCAAATTGAAAAAGGACCTTTACGTCAAACTAACTAATGAAAACACCATTACGA

AAAGCTTCTCCCCTATTAAAAATTATTAATAATAGAATTATTGATTTACCTACACCATCTAATATTTCTGCTTGATGAAATTTTGGATCCTTATTAGGAC

TCTGTCTTTTTATTCAAATTATCACTGGAATTTTCTTAGCTATACATTTTACAGCCCATATTGACATAGCATTTAATAGAGTAATTCATATTTGTCGAGA

TGTAAATTATGGCTGATTATTACGAACAATTCATGCTAACGGAGCTTCTTTTTTCTTTATTTGTATTTATCTACATATTGGCCGAGGAATATATTATAGA

AGCTATAATTTACATTTAACATGAACTATTGGAGTAATCATCCTTTTTATAGTAATAGCTACAGCATTCTTAGGGTATGTATTACCTTGAGGGCAAATAT

CTTTTTGAGGGGCTACTGTTATTACCAATCTTCTCTCTGCTATTCCTTATCTAGGAAATATGATTGTCCAATGATTATGAGGGGGATTTGCAGTAGATAA

CGCTACTTTAACTCGATTTTTTACATTACATTTTATTCTTCCTTTTATTATTTTAGCTTTAATAATTATTCATTTATTATTTCTTCATCAAACTGGTTCT

AATAACCCTTTAGGATTAAATAGAAATATCGATAAAGTACCTTTCCATCCATATTTTACTTACAAAGATACTTTTGGATTCATTATAATAACTATATTAT

TAATATTTTTAGTTTTAATTAATCCCTATCTTTTAGGAGACCCAGAAAACTTTACACCTGCTAATCCTTTAATAACCCCAGCCCATATCCAACCAGAATG

ATACTTTTTATTTGCATACGCTATTTTACGATCAATTCCTAATAAATTAGGTGGAGTAATTGCTTTAGTTATATCAATTGCTATTTTATTAATTATACCT

TTAATTAACAAAAAAAAATTTAGTAGAACTCAATTTTACCCATTAAATAAAATTTTATTTTGATCTTTTGTCTCTATTGTAATCTTATTAACATGAATCG

GAGCTCGCCCAGTGGAAGATCCATACATTTTAACAGGTCAAATTTTAACAATTATATATTTTAGTTATTATTTCTTAAACCCCTTAATTCATAAAATATG

AGATTATATTATTTTTAAAACTTAGTTAATGAACTTGTTAAAGTGTATATTTTGAAAATATAAAAAAGAGTTTATTCTCTATTAACTTTACTAAATTTTA

TTCACTAAATAAAATAAGTAAAGATAACCAACTTTAAACCTAAAAAAAAAAATAAAAAATTTAAAGAAACTGGTAAATAACTTTTTCAACATATATATAT

TAACTTATCATAACGGTACCGAGGTAAAGTACCTCGAACTCAAATCCAAATAAATGCTATAAAAGTTAACTTCAAAAAAAAAAATAAAGAAAAAATATCT

CCCCCTATAAATAACAACACACATAATATACTTATAAATAAAATTCTAGCATACTCAGCCAAAAAGATTAAAGCAAATCCCCCTCTTCTGTACTCTACAT

TAAACCCAGAAACTAACTCTGATTCTCCTTCTGCAAAATCAAAAGGAGTTCGATTAGTCTCAGCTAAACTTGAAGATAATCATATTATTCTTAAAGGTAA

GCACAGAAAAAAAAATCATACTATTTCTTGATATTTTATAAAATCTATTATATTTAAATTTAAAATTAACAATAAAAAAGATAATAAAATTAATGATAAA

CTTACTTCATATGAAATTGTTTGAGCTACTGATCGAATACCCCCTAATAAAGCATAATTAGAATTTGATGATCAACCAGAAATTATGATAGTATAGACCC

TTAGCCTAGAACAACATAAAAAATATAAAATACCTAAATTAAACCTAAATATAAATGTCAAAAAAGGTATACATATCCATAATAAAAGAGCTAAAAATAA

ATTAAAAACTGGAGATATATAATAAATAAAAAAATTTGATATTAAAGGATAAGTTTGTTCTTTAGAAAATAACTTAATAGCATCACTAAAAGGTTGAGGA

ATACCTATAAATCCAACTTTATTGGGCCCTTTACGAATTTGGATATACCCTAAAACTTTACGCTCTATTAATGTTAAGAAAGCTACACCAATTAAAACAC

AAATAATTAAAACTAAACTTGTAAATAATAAAAGAAATAAATCTTGTAATATAATGTATTACTTGTGTTAAACACATATTTAAATTCTAAATTTAAAGCA

CTAATCTGCCAAAGTAATATTCATATTCAAATTATATTAAATTTTAAAGGTATCTGATCCTTTCGTACTAAAATACCTATGTTTTTTAAAGATAGAAACC

AACCTGGCTCACGCCGGTTTAAACTCAGATCATGTAAAATTTTAAAGGTCGAACAGACCTAACCTTTTAGCCCCTACACCAAAAGTTAATTTTAATCCAA

CATCGAGGTCGCAAACTTTTTTTTCGATAAGAACTCTAAAAAAAAATTACGCTGTTATCCCTAAGGTAATTTAATCTTGTAATCATTAAAAATGGATCAT

TCAATCATAAATTAATGTTTTTAAATAAAAAAAGTTTAATCAATTTTTCTGCTGCCCCAGCAAAATAGTTTAAATTATTAAATATATAAATATACTAAAA

TTAAATAATAATTTAAACTATAAAACTCTATAGGGTCTTCTCGTCTTTTAAAATTATATAAGCTTTTTTACTTATAAATAAAATTCTATATTCAATTAAA

TTGAGACAGTTACTTTCTCGTCCAACCGTTCATTCCAGCTTTCAATTAAAAAACTAATGATTATGCTACCTTTGCACGGTCAAATTACCGCGGCCATTCA

AATCCTCATTGGGCAGGTCAGACTTTAAATTATAATCAAAAAGACATGTTTTTAATAAACAGGCGAAAAGTGTATTTGCCGAGTTCCTTAATTTAACCTT

GAAGTTTTAATTTAATTACTAAATTAAAATATATACTAATTTTATCATTATTCTATATAAACCAATATTACATATATTATCTTAATAAACTACTTAAAAA

TAATATAAATCTTATTCTAACAAAAATTATTTATAACAAACTAAAGATTAACACTTCCAATTCTACTAATTTTTATTCAAAATATACATTTTTAACATTT

TATTTTAAAGCTTATCCCCTAAAATATTACTTTTTATATATAAAATACTAAATAATTAATATAATACAATAAAAAAACTAAATTAAATTTATTTCTTAAG

AAACTAGATATCTTAAAAAACGTATAACGTTTCATTTCTAATATAATATTTTAAAAATTTATGCCACAATTAAATTTATATTATATTAGCTCTTTATAAT

TCGAGAACACTAAATAATTAAATTATTTTAATAAACCCTGATACACAAGGTACAAAAAATTAATTTTTCTTTTTAAAAAATAAATCTCTATATATTTATA

TTATCTATCTCTATACAAATTAACTATAATAAAATTTTTATATTCTAAAATATACTAATATCAAAAATATTTTTTTTATATATATATATATA----TTAT

AAATTTTTCCTTTCAAATTAAATTGATTTTCACAACTAACTTTTTAATGTAAATAAAATGCTTTATTACAAGCTCTAATTTGCCATTCCAGGTACACTTT

CCAGTACACCTACTATGTTACGACTTATCCCTCCTTAGAGAGGGAGCGACGGGCGATATGTACATATTCTAGAGCTATACTCATATAATTAAACTAAACT

ATATTACTTTCAAATCCACTTTATAAAATAATGTTAATTATTTTAACCATCTAAATAATTTTATTGTAACCCATCTCTCCTTATCTATACGCTGTATCTT

GATCTGATTTTTTTTATACTTATAAATTTTGAACATTCCAAATTCTTTAAAAACATTCAACCTACGACGATATACAAACCTTTAAAATAAGTACGATTAA

TCGTGGATCATCAATTATAGGACAGGTTCCTCTGAGTAGACTAAAATACCGCCAAATTCTTAAAATTTCAAGAACATAACTACTACTATTCAAGCATCTA

AAATTTGCATTTTTAATAATAGGGTATCTAATCCTAGTTTTTTATAAAAATCTCATAAACTCATTTTTCACATTTAAAAAATTAATTATACTTACTAATT

TCACCTAATAAATATAATATAAATTAATAATAAAATAACTTATTATATACTGAACAAATTTAATTGCATTGTTTGTGTAACCGCAACTGCTGGCACAAAC

TTGGTCAATACTATTATAAATTCCTAAATCAAAATTTCTTTTAAATTTAATCTTCACTATTGCAATTCTTTAATTAAATATATAGAATAATTTATCTTTT

TAAAATAAATTCATTAAACACTAAAATTTACATATAAAATAATTTAAAAATTAAAATCTCAAGCTAGAATAAAACTTTATTTTTTTTTCAATATACATAA

ATTATATAAAATAAAAGTACCCCCC-TACCATTTTAATTAAGTGAATTAATTTTTAATGCCTTAAATTTATTTCCAATTTATTTTTATTATATAGTTTTT

TATTTAAAAATAAAACTTTATACCTAAAATTATATACATAAATTATATTTAAAATTTATAATTTAAAGAACTATAAAAATTAAATTAAAATTTAGAATCT

ATCTAATTTATAGATAAACAAATTAAATTAAACTAACTTACCTGATAAATTTAACTATATACGCGCAGGTATATTAATTATTTAATTATAATATAATACA

TTTGTTTTATTAAATAATTATTAAGCAAAATAGCATTAATTTATTATATAAAAGATCTAGATACTATTAATTTAAAGTAAATTTTTTTTTTTTTTGTAGT

ATAATTTATACTAATATACGGCATTTAATATTTTCTATATAAATGTTTATATTATTTATATTTATCTATTTATAATTCTTAATAATAATTTAGATAATTA

TTAATTTATATATTAAATATTTATATATATATATATAATAATAAGGATATATATATATATATGTATATAGATAAATTAAATTTAGTTATATATAAAAAAA

TTAATATTAATTTTACTTTTAATTTTTTCTTTAATTTAAACTATTGGATATTCAGATTCTAATATATTATAATATATCAACCATTAATGAATAACTTTAT

ATATATAATAAAAATGTTAATTAATATATCAATTCATATGAATATATAATATTAATAAATAAATTCTTAATAAATAATAATGATAATATATATATATTGT

ATATTAATGTACTATATATATAAATTCATTAAACATTTATATTTGATACCCCCCCAAAATTTTTTCTAAAAATGTGAAGTTTCAATTTAATTAAAATTTT

CTACAATGGTCAAAAACGGCCAAAAAATGGAAAATAAAATGTGCACAGAAAATGCACATAAAATGCACATTTTTGAAAATTTCCGATTTTTGAAAAACCG

TTTGAACCAAACCTCTGATGTACCTTTTTTCGCCCCCCCTGATTACTAAAATTTATCCTCAATTACTAAGGTTTATCCGCATTTTTGCGACAGGAATTTT

GAGTATAAAATCAATTCCACACTAAATTTTTTTTT-TTTTTTTTGTGTCATTTCTCCAACATGGGCAATTTTTTTTTTAGTCGAAGGACAAATTTTAGTA

AAAAAAGTTTGAAATTAAGTTAAACCCTTGTTTAACCAAAAACTATTACTTTTCAATATTATACCCAATTTTATTTAAAATAAATTTTATTATACTTTTA

TTTATGCAAAAAGATCTTCTTATAAACTTTT

>DMR179j_JP_Sapporo_Hokkaido

AATGAAGTGTCTGACTATAGAGTTACTTTGATAGAGTAAAAAAAGTGAATTTTCACCTTCATTATAATTAACAGAATTAAACTATTTCTTTAAGCTTCAA

AAACTTATGTACATTATATACTAAATTATAAAAAGATAAGCTAATTAAGCTACTGGGCTCATACCCCATCAATAAAGGTTACAATCCTTTTCTTTTTAAT

GTATTATAAGCTTTTATTTTTTAATTCTCTTATAATTGGAACCTTAATTGCTATTTCTTCCTACTCTTGGATAGGAATATGAATAGGATTAGAAATTAAT

CTTCTCTCTATGATCCCCCTCATTAGAGACAACAAAAATATAATAGCCTCAGAAGCTGCTTTAAAATATTTCATTATTCAAACAATAGCATCAACATTGT

TATTATTCTCAATTATTATAATATCAATGAAATTTATATATCAAATAAATTTAATCACTTACTTTAATTTAATTTTTAACACTTCATTATTCATCAAAAT

AGGAGCAGCCCCATTCCATTTTTGATTCCCCGAAATAATAGAAGGATTAAATTGATTAAATGCCATTATCATACTTACTTGACAAAAACTAAGACCCATA

GTATTACTAACGTATTCTAATACAACCCCCATATATCTAATTTTAACAATTATATTTAGAATAATAATCAGAGGAATTATAGGTCTAAATCAAACTAGAT

TACGAAAAATTATAGCTTATTCATCTATCAACCATATTGGATGAATAATCAGTTCAATTATACTAATTGAAATTGTTTGATTTTACTATTTTATTATTTA

TTGCATTATTACTATTAATATCAGAATTATATTTATAAAATTAAATGTTTTCCATATCAATCAATTATATATTTCAATAAATTACCATATTTTACTTAAA

TTATTCTTTGCCTTAAATTTCATGTCTTTAGGAGGATTACCCCCATTTTTAGGGTTTTTCCCTAAATGACTTACAATTCAAACTTTAATCCAAAGAAATA

TATACTCAATTGCTTTCATTATAATTTTAATAACTTTAATAACACTTTTTTTTTACCTCCGAATTACTTTTTCAATTTTACTACTAAGAAAAACAGTTTT

AACATTTTACGCACAACCAAAAATTTATACTAATTACATTATAGCATTTAATTTTATTACATTATTAAGATTAATTTTCGTTACTTTGATATTCAATTTC

TTATAAATTAAACCTGAAGGATTTAAGTTAAATTAAACTAAGAACCTTCAAAGTTCTAAATAAAGTAAATTCTTTAAGCCTTAGGGCTTAGCCCATCTTT

AAATTTGCAATTTAAAATTCTTTTTGAACTATAAAGCTTGATAAAAGAAACTAATTTCGTATGTAAATTTACAGTTTACCGCCTAAACCTCGGCCATTTT

ATCGAATAAATGGCTATTCTCTACAAATCACAAAGATATCGGAACTTTATACTTTCTATTTGGAAGTTGAGCAGGCATAGTAGGCACTTCCTTAAGTTTA

TTGATTCGTGCTGAATTGGGAAACCCAGGATCTCTAATTGGAGATGATCAAATTTATAACGTAATCGTAACAGCTCATGCTTTCATTATAATTTTTTTTA

TAGTAATACCTATTATAATTGGGGGATTCGGAAACTGATTAGTTCCTTTAATGCTTGGAGCCCCCGACATAGCATTTCCCCGAATAAATAACATAAGATT

TTGACTTTTACCTCCTTCATTAACTCTACTTCTAATAAGAAGATTAGTCGAAAGAGGAGCAGGTACAGGCTGAACAGTTTATCCCCCACTATCAGCCAAT

ATCGCCCATAGAGGAGCATCAGTTGATTTAGCAATTTTTAGACTTCATCTTGCAGGAATTAGCTCAATTTTAGGTGCAGTAAATTTTATTACTACTGTAA

TTAACATACGATCAACAGGAATAACTTTTGATCGAATACCCCTATTTGTTTGATCTGTAGTTTTAACAGCACTTCTTCTGCTATTATCCCTCCCAGTTTT

AGCAGGAGCAATTACTATACTATTAACAGATCGAAATATTAACACAACATTTTTTGACCCCGCAGGTGGAGGAGACCCCATTCTCTACCAACATTTATTT

TGATTTTTTGGTCATCCAGAAGTTTACATTTTAATTTTACCTGGATTTGGTATAATCTCCCATATTATTAGCCAAGAAAGAAGAAAAAAAGAAACATTTG

GAACTTTAGGTATAATTTACGCTATAATAGCTATTGGTTTACTAGGATTTATTGTATGAGCTCATCATATATTTACAGTAGGTATAGATGTAGACACACG

GGCATATTTTACATCAGCAACAATAATCATTGCTGTTCCTACAGGAATTAAAATTTTTAGATGATTAGCTACTCTCCATGGATCACAATTAAACTATTCC

CCGTCTCTTTTATGGGCATTGGGATTTGTATTCCTATTTACAGTAGGAGGATTAACAGGGGTAATTCTAGCTAATTCATCAATTGACATTATTTTACATG

ATACTTACTATGTAGTTGCACATTTCCATTATGTCCTTTCCATAGGAGCTGTATTTGCTATTATAGCAGGTTTTGTTCATTGATTCCCTTTATTCACAGG

TTTAACAATAAATTCAAAATTTCTTAAAATTCAATTTTTAACAATATTTATTGGTGTTAATATCACATTCTTCCCCCAACATTTCTTAGGATTAAGAGGA

ATACCTCGACGTTATTCAGATTACCCAGATGCTTATACAACTTGAAATATTATCTCATCTATTGGATCTTTAGTTTCTTTAATTAGTATTTTTATCTTTT

TATTTACTATTTGAGAAAGGTTAATTTCATTACGAAAAAGAATTAGGTCTTTAAGAATATCTACATCAATTGAATGACTCCAACAAATACCCCCTTCAGA

ACATAGCTATTCTGAACTTCCAATGCTTACTAACTTCTAATATGGCAGATTAGTGCAATGGATTTAAACCCCAAATATAAAGATTAAACTTTTTTTAGAA

ATAGCTACTTGAAATACCATTTTACTTCAAGATAGGGCATCCCCATTAATAGAGCAACTCTCATTCTTTCATAACCATGCTCTTCTAATTCTCTTTATAA

TTACCGTTCTAGTAGGTTATTTAATAGGAACTTTATTTTTTAACCAATTTAATTACCGATTTTTATTAAATGGTCAAACTATTGAAATTATTTGAACTAT

TTTACCTGCTGTAACACTAATTTTTATCGCATTACCGTCTTTACGCTTACTTTATCTTCTAGATGAAGTTAATAACCCTTTAGTAACTATCAAAACAATT

GGGCATCAATGATATTGATCATACGAATATAGAGATTTTATAAATTTTGAATTCGATTCCTATATAATTCCTTTGACAGAAATAAAACCTCAAAATTTTC

GTTTATTAGATGTTGATAACCGAGTAATTGTCCCCTTTAACTCCCAAATCCGAATGATAGTAACAGCTGCCGATGTTATTCATTCATGAACTATCCCAGC

TTTTAGTGTAAAAATTGATGCAACACCGGGCCGACTAAATCAAATTAGATTCCTAATTAATCGAACAGGATTATTTTATGGTCAATGCTCAGAAATTTGT

GGAGCAAATCATAGATTTATACCTATTACTGTAGAAAGAATTTCACCTTCATTTTTTACTAAATGAATCTCAAAAATAAATAACCTATCATTAGATGACT

GAAAGTAAGTAATGGTCTCTTAAACCAATTAATAGTAGTTTAACATCTACTTCTGATGGCCAAAAATTTAGTTAAGATATAACATTAGTTTGTCATACTA

AAATAATCATAATTTGATAATTTTTAATTCCACAAATAGCACCTTTAAACTGACTATCTTTATTTTTTTTAATTATTATTATTTTTTTACTTTTTAATGT

ATTAAATTACTTTAGATTCTTACAACCTTTAAAAACCCAATCTCATAACCCTACAATTAAAAAAATTAATTGAAAATGATAACTAATTTATTTTCATCTT

TTGATCCTAGAACTTCTTTTAATTTGAGATTAAACTGATTAAGAATACTATTAGGGCTAATATTTATCCCCCCAATATTTTGATTAGTTCCTTCACGCCA

TAATTTTCTATGAATTAAAATTATTTTAACATTACACCAAGAATTTAAGGTTTTAATTGGTAATAATAATATTAAAGGAAGAACCTTAATATTTATTTCA

TTATTTTCTATAATTGTTTTCAATAACTTTTTAGGATTATTTCCGTATATTTTTACAGGAACAAGACATTTAATTATAACATTATCTCTTGCCTTACCTT

TATGAATTAGATTCATATTATACGGGTGAATTAATAACACTATCCACATACTTGCTCATTTAGTTCCCCAAGGAACACCCCCAGCTCTTATGGCATTCAT

AGTAGTAATTGAATCAATTAGAAATATTATTCGTCCTGGTACTTTAGCTGTTCGATTAGCTGCTAATATAATTGCTGGACATTTACTAATAACTTTACTA

GGAAACACAGGATTAAATTTATCAATTTTTATATTAAGTATTCTTATTATCATACAAATTCTTTTATTAATTTTAGAATCTGCTGTTGCGATCATTCAAT

CTTATGTATTTGCTGTATTAAGAACTTTATACTCTAGAGAAATTAATTAATGTCAAGACATAAAAATCACCCTTATCATTTAGTTGATGCAAGGCCTTGA

CCTATTTTAGGTGCTTTTAGAGCTATAATTACAATAATTGGAATTATCAAATGATTCCATTTTTATAATAATTCTTTATTTTACTTAGGGACATTAATTA

CAATTTTAATTATAATTCAATGATGACGAGATATCACTCGTGAGGGAACTTTCCAAGGACTTCATACTTACGCTGTAACTATAGGTTTACGTTGAGGAAT

AATTTTATTTATTACATCAGAAGTATTTTTCTTTATTTCTTTTTTTTGAGCCTTTTTTCATAGTAGTTTAACACCCGCTATTGAACTAGGGATACTCTGA

CCACCTAAAGGAATTACTCCATTTAACCCTATTCAAATTCCATTATTAAACACTTTAATTCTTTTAACTTCGGGATTAACTGTAACTTGAGCTCACCATA

GATTAATAGAAAATGACTATAACCAAACAATACAAGGTCTTGGTTTAACAGTTTTACTAGGAATATATTTTACTTTATTACAAGGTTACGAATATTTAGA

AGCCCCCTTTACTATAGCAGATTCTGTTTATGGATCAACATTTTTTATTGCTACTGGTTTCCATGGATTACATGTTATTATTGGCACAACCTTTTTAGCT

GTTTGTTTAATACGGCATTTTAATAACCATTTTACTTGCATCCATCACTTTGGATTTGAAGCTGCTGCTTGATACTGACATTTTGTTGATGTAGTATGAC

TATTTCTTTATATTTCTATTTACTGATGAGGTAGATATTTATATAGTATAATAATTATAATTGATTTCCAATCAAAAGATCTAAAAAA--TTAGTATAAA

TAATCATTATAATTTGAAATATAGGGTTAATTATTTTTTCTATCTCTTTTATCTTAATTATACTATCTTTTACAATCTCTAAAAAAAGATTTATAGACCG

AGAAAAAGCTTCTCCATTCGAGTGTGGATTTGACCCCAAAAGATCAGCCCGTTTACCCTTTTCTTTGCATTTTTTTTTAATTGCAGTAATTTTCTTAATT

TTTGATGTTGAAATTACTCTTCTTATCCCTTTAATTCTAACAATAAAAATTACTAATATTACTATATATACCTATATTGCTCTTTTCTTTTTAATGATTC

TTTTAATAGGACTTTACCATGAATGAAACCAAGGAGCCTTAAATTGAGCTCTTTAGGGTAATAGTTAAGTATAACATTTAAGTTGCATTTAAAAAGTATT

GATTTTTCAATTTACCTTAAATAAGAAACAATTAATTGTATTTAGTTTCGACCTAAAATTTAGGTGTATGAACACCCTTATTTAAATTAATTGAAACCAA

AAAGAGGTATATCACTGTTAATGATACTAATGAGAAAAGCTCCAATTAAGGAAATAAGATATTCAAGAGTAAGCTTCTAACTTAACTCTTTAGCAGTGAA

AGTCTGTTAATATTTCTATTTATATAGTTTAATAAAACATTATTTTTTCATAATAAAATTAGAATAAATTTATTCTTATAAATATTTAAAAGTAAATTTT

ACTTCCCTGATAACTTCACTATCATACTCTCTATAAGCTATTTAAATTAAATATATAAAATTATAAAAATTACCCATATTATAATTAAAAGTATAAAAAT

CTTATAATTGTTATTAAATATAAACTGTAAAAATACAGAAGTATTACTAATTTTACTATATAAATTTTGTCTTCCATAATACTCTGATCACCCTTGATCA

ATAGTTTTATATAACTTACTACCTAATTTAATTGGATAATAATTTAAACCAAATGTTGAAATATAAGGTATATTCCACATAGAAGAAAAAAAAAGTCTTG

AATTTAACAAATAAAAAGATTTTAATCTATCATTTAAAGTAAACTTAGAAAGTTCAAACCCAAACCAAGCCCCAAAAAATGATACAATTAAAGCTATAAT

TTTTATTGTAAAAGGTAAACAAATAAAATAAGGTGTTGGGAATATCAGCCATATCAATATTCTTCCCCCAACAATAACTAAAAAAATTAAACCTGATATC

CCTTGAAGTATAATTTTTCTATTATCATTAATTTTACTTAAAGAATAAAAACAAAAATTTCCTACTAAAACATAATAAATTAAACGAAATGTATAACAAA

CAGTTAATCCTGTTGAAAAAAAGAAAATAACATAAATATAGATATTTAAATATCTTATAGATAAAACCTCTAAAATTAAATCCTTTGAATAAAACCCTGA

TAAAAAGGGTAAACCACATAATGCTAAATTTGAAATTATAAAATAAGTACAAGTTAAAGGTATAACTTTAATTAACCCCCCTATATACCGAATATCTTGA

CAATTTCTTAATCTGTGAATTATACACCCTGCGCATATAAATAATAAAGCCTTAAATAAAGCGTGAGTTAATAGATGAAAAAAAGCTAATTGATACTCCC

CTAAAGCTAAAATACTAATTATCAAACCCAATTGTCTTAAAGTAGATAGAGCAATAATTTTTTTTAAATCAAATTCAAAGTTTGCACCTATTCCTGCTAT

AAATATAGTTATTGTTCCAATAAATAATAAAATTAATATTAAATTACTTGTTAATGCAAAATTAAAACGAATTAATAAGTAAACACCTGCTGTTACTAAG

GTAGAAGAATGAACTAAAGAAGAAACAGGCGTTGGAGCTGCTATTGCCGCAGGCAACCAAGAAGAAAATGGAATTTGAGCTCTTTTAGTTATAGCTGCTA

ATATAATTAATAAAATAATAATATATATTTCTATGCTATTTTTATACACATCAATATAAAAAATATAGTTAAATCCCCCAAAATTTATTATTCATGCAAT

TGCTATTAATAAAGCAACATCTCCAATACGGTTAGTTAAAGCTGTAATTATCCCTGCATTATAAGATTTAATATTTTGATAATAAATAACTAAACAATAA

GAAACTAACCCTAACCCATCTCATCCTAATAAAATCCTAATTAAATTAGGAGAAATAATCAATAACATTATAGATAAAACAAATATAGAAACTAATATAA

TAAATCGATGCAAATAAATATCTCCTTCTATATACTCTTCACTATAGTAAATTACTATAGAAGAAATAAATAAAACAAAACTCATAAATAATAATGACAT

CCAGTCAAGTAAAATAGTTATAATAATTCTACAAGAATTAATCCTTAATATTTCATATTCTAATATTAGTCTATAATCTAAAATTATGAAATTTAAGCTT

AATAAAAATCTTAATACTCTAAAAAATAAAAACGTTACAAAATAAATTAAACAAATAGAAATAATTTAAAGTAAATTTTACATCTTTGATACCACAAATC

AATATTTTTTATTAAACTATTTAAATTACAATCATAAAACTAAAAATTCTCTCTTTAAAATTAAGATATTCAAGGGTAACCAATGAAGTAATAATAATAA

ATACTCACGAACAAACCCTCTTGAAAAAGAATATAAATTTCTAACTAACTTGCCGTGTTGACTATAAGAATATAAATATAAAGAATACGCAGCTCTAAAA

AAAGATATTAAAGATAAAAAAACCATTGTTCAACTCCTTCATCTAACTAATCTATTAATTAAAATAATCTCACCCAGTAAGTTTAATGAAGGAGGAGCCG

CTATATTACAACAACTAAATAAAAATCACCATATTCTTATTATTGGCATTAAATTGATTAGCCCCTTATTTAAATAAATACTTCGGCTATTTAGCCGTTC

ATAAGAAATATTTGCTAAACAAAATAATCCTGAAGAACATAAACCATGGGCAATTATTATAACTAAAGCCCCTCTTATACCCCAATAACTTAAAGTTAAA

ATCCCTCTTAATACTAAACCTATATGCGCTACAGAAGAATAAGCAATTAAAGCTTTAATATCAACTTGACGTAAACATATTAATGAAACAAAAAATCCCC

CTACTATTCTAATTGTAATAAAAATATAGTTTACTTGTAAACCTACAGTTAAAAAAATATTTATTAAACGTATTAATCCATACCCCCCTAATTTTAATAT

AACTCCAGCTAAAATTATAGAGCCAGCAACTGGAGCTTCAACATGGGCTTTAGGGAGCCAAAGATGAACAAAAAATATAGGCATTTTAATAAAAAACACT

ATATTTATACATATAAATAACAAGAATCTTTTTACATCATAACATAAAAAGAAAAAATCTAAAGAATGAAATTTTTCATAATAATAAAAAATCCTAATTA

TTATAGGTAATGAAGCAAATAATGTATAAAATAACAAGTAAACACCTGCTTGCAAACGCTCAGGTTGATACCCCCAACCAATAATTAATAGAAGAGTGGG

AATTAAGCTAAACTCAAAAAACAAATAAAAAATAAATAAATTTAAAGAACTAAATGTTATAACTAAAGATAATAATAAAATAATTATTACTAATAAAAAT

AAATTATAAAAATAATTTTTTCTATAAATACTTTCAGAAGCTAATAATATTAAAGAACAAATCCACAAACTTAATAAAATTATTATAAAAGATAATAGGT

CATACCCTATAAAATAAGAAATATTTATATACAAATAATTAAAACTAAATCTTAACCCAAATATAAATGTAATAAAAAAATATATATATTGATTAAATCA

GTATCTCTTTTTAACACAACTTAAAGGTACCAATATTAATATTATAAAAATAAACTTTATCATAATACATTAAAAGTTTGAAAATAATCATTTCCATGTG

TTCGTATTATAGAAACTAATAACGATAAACCTAAAGCTCCTTCACAAACTCTTATAGTTAAAAATACTATACCAAAATAAAATTCAAAATTAAAATATAT

TAAAAATAGGTATAAATTAAAGTATAATCCTAAAATAATATACTCTAACCTTAATAATATTAAAAGTAAATGTTTACGTTTAATGCAAAAAGAAACTAAT

CCTGTAAAATATATAATCACTGAAAATAACATACAAAAAATTAACATTAGTTTTAATAATTTAATAAAAATACTGATCTTGTAAATCAGAAATAAGAATT

TTCTTTTAAAACTTCAGAGAAAGAGTAAACCTCTATCATTAATCTCCAAAATTAATATTTTAAATAAACTATTCTCTGTATAATCTTATTACTAATAACA

TTATCTTTTATTAGTTCAATAACTTTTATATTTTTAAGTCACCCTTTATCTATAGGGCTTATTTTATTAATACAAACTATTATTATAGCTTTAACTATAG

GTTTTTTTAATATTAATTTCTGATATTCTTATATTTTATTTCTTATTATAATTGGAGGAATATTAGTTTTATTTATTTATATAACAAGAGTAGCTTCAAA

TGAAAAATTTTCGTTTTCTATTAAAATTACATTAATAATTAGAATTATAACTTTAGGATTTTTATTTAGTATTGCTATAATAGATCCCTACTTTTCAGAC

ATCAACTCAATTTACACAGAAAACTTAGATAATTATAAAGAATATAATATATCATTTAGAAAATATTTAAGTTATCCTAATATTATCATTATATACATAA

TAATTATTTATTTATTAATTACCTTAATTGCAGTTGTAAAAATTACTCAAATTGAAAAAGGACCTTTACGTCAAACTAACTAATGAAAACACCATTACGA

AAAGCTTCTCCCCTATTAAAAATTATTAATAATAGAATTATTGATTTACCTACACCATCTAATATTTCTGCTTGATGAAATTTTGGATCCTTATTAGGAC

TCTGTCTTTTTATTCAAATTATCACTGGAATTTTCTTAGCTATACATTTTACAGCCCATATTGACATAGCATTTAATAGAGTAATTCATATTTGTCGAGA

TGTAAATTATGGCTGATTATTACGAACAATTCATGCTAACGGAGCTTCTTTTTTCTTTATTTGTATTTATCTACATATTGGCCGAGGAATATATTATAGA

AGCTATAATTTACATTTAACATGAACTATTGGAGTAATCATCCTTTTTATAGTAATAGCTACAGCATTCTTAGGGTATGTATTACCTTGAGGGCAAATAT

CTTTTTGAGGGGCTACTGTTATTACCAATCTTCTCTCTGCTATTCCTTATCTAGGAAATATGATTGTCCAATGATTATGAGGGGGATTTGCAGTAGATAA

CGCTACTTTAACTCGATTTTTTACATTACATTTTATTCTTCCTTTTATTATTTTAGCTTTAATAATTATTCATTTATTATTTCTTCATCAAACTGGTTCT

AATAACCCTTTAGGATTAAATAGAAATATCGATAAAGTACCTTTCCATCCATATTTTACTTACAAAGATACTTTTGGATTCATTATAATAACTATATTAT

TAATATTTTTAGTTTTAATTAATCCCTATCTTTTAGGAGACCCAGAAAACTTTACACCTGCTAATCCTTTAGTAACCCCAGTCCATATCCAACCAGAATG

ATACTTTTTATTTGCATACGCTATTTTACGATCAATTCCTAATAAATTAGGTGGAGTAATTGCTTTAGTTATATCAATTGCTATTTTATTAATTATACCT

TTAATTAACAAAAAAAAATTTAGTAGAACTCAATTTTACCCATTAAATAAAATTTTATTTTGATCTTTTGTCTCTATTGTAATCTTATTAACATGAATCG

GAGCTCGCCCAGTGGAAGATCCATACATTTTAACAGGTCAAATTTTAACAATTATATATTTTAGTTATTATTTCTTAAACCCCTTAATTCATAAAATATG

AGATTATATTATTTTTAAAACTTAGTTAATGAACTTGTTAAAGTGTATATTTTGAAAATATAAAAAAGAGTTTATTCTCTATTAACTTTACTAAATTTTA

TTCACTAAATAAAATAAGTAAAGATAACCAACTTTAAACCTAAAAAAAAAAATAAAAAATTTAAAGAAACTGGTAAATAACTTTTTCAACATATATATAT

TAACTTATCATAACGGTACCGAGGTAAAGTACCTCGAACTCAAATCCAAATAAATGCTATAAAAGTTAACTTCAAAAAAAAAAATAAAGAAAAAATATCT

CCCCCTATAAATAACAACACACATAATATACTTATAAATAAAATTCTAGCATACTCAGCCAAAAAGATTAAAGCAAATCCCCCTCTTCTGTACTCTACAT

TAAACCCAGAAACTAACTCTGATTCTCCTTCTGCAAAATCAAAAGGAGTTCGATTAGTCTCAGCTAAACTTGAAGATAATCATATTATTCTTAAAGGTAA

GCACAGAAAAAAAAATCATACTATTTCTTGATATTTTATAAAATCTATTATATTTAAATTTAAAATTAACAATAAAAAAGATAATAAAATTAATGATAAA

CTTACTTCATATGAAATTGTTTGAGCTACTGATCGAATACCCCCTAATAAAGCATAATTAGAATTTGATGATCAACCAGAAATTATGATAGTATAGACCC

TTAGCCTAGAACAACATAAAAAATATAAAATACCTAAATTAAACCTAAATATAAATGTCAAAAAAGGTATACATATCCATAATAAAAGAGCTAAAAATAA

ATTAAAAACTGGAGATATATAATAAATAAAAAAATTTGATATTAAAGGATAAGTTTGTTCTTTAGAAAATAACTTAATAGCATCACTAAAAGGTTGAGGA

ATACCTATAAATCCAACTTTATTGGGCCCTTTACGAATTTGGATATACCCTAAAACTTTACGCTCTATTAATGTTAAGAAAGCTACACCAATTAAAACAC

AAATAATTAAAACTAAACTTGTAAATAATAAAAGAAATAAATCTTGTAATATAATGTATTACTTGTGTTAAACACATATTTAAATTCTAAATTTAAAGCA

CTAATCTGCCAAAGTAATATTCATATTCAAATTATATTAAATTTTAAAGGTATCTGATCCTTTCGTACTAAAATACCTATGTTTTTTAAAGATAGAAACC

AACCTGGCTCACGCCGGTTTAAACTCAGATCATGTAAAATTTTAAAGGTCGAACAGACCTAACCTTTTAGCCCCTACACCAAAAGTTAATTTTAATCCAA

CATCGAGGTCGCAAACTTTTTTTTCGATAAGAACTCTAAAAAAAAATTACGCTGTTATCCCTAAGGTAATTTAATCTTGTAATCATTAAAAATGGATCAT

TCAATCATAAATTAATGTTTTTAAATAAAAAAAGTTTAATCAATTTTTCTGCTGCCCCAGCAAAATAGTTTAAATTATTAAATATATAAATATACTAAAA

TTAAATAATAATTTAAACTATAAAACTCTATAGGGTCTTCTCGTCTTTTAAAATTATATAAGCTTTTTTACTTATAAATAAAATTCTATATTCAATTAAA

TTGAGACAGTTACTTTCTCGTCCAACCGTTCATTCCAGCTTTCAATTAAAAAACTAATGATTATGCTACCTTTGCACGGTCAAATTACCGCGGCCATTCA

AATCCTCATTGGGCAGGTCAGACTTTAAATTATAATCAAAAAGACATGTTTTTAATAAACAGGCGAAAAGTGTATTTGCCGAGTTCCTTAATTTAACCTT

GAAGTTTTAATTTAATTACTAAATTAAAATATATACTAATTTTATCATTATTCTATATAAACCAATATTACATATATTATCTTAATAAACTACTTAAAAA

TAATATAAATCTTATTCTAACAAAAATTATTTATAACAAACTAAAGATTAACACTTCCAATTCTACTAATTTTTATTCAAAATATACATTTTTAACATTT

TATTTTAAAGCTTATCCCCTAAAATATTACTTTTTATATATAAAATACTAAATAATTAATATAATACAATAAAAAAACTAAATTAAATTTATTTCTTAAG

AAACTAGATATCTTAAAAAACGTATAACGTTTCATTTCTAATATAATATTTTAAAAATTTATGCCACAATTAAATTTATATTATATTAGCTCTTTATAAT

TCGAGAACACTAAATAATTAAATTATTTTAATAAACCCTGATACACAAGGTACAAAAAATTAATTTTTCTTTTTAAAAAATAAATCTCTATATATTTATA

TTATCTATCTCTATACAAATTAACTATAATAAAATTTTTATATTCTAAAATATACTAATATCAAAAATATTTTTTTTATATATATATATATA----TTAT

AAATTTTTCCTTTCAAATTAAATTGATTTTCACAACTAACTTTTTAATGTAAATAAAATGCTTTATTACAAGCTCTAATTTGCCATTCCAGGTACACTTT

CCAGTACACCTACTATGTTACGACTTATCCCTCCTTAGAGAGGGAGCGACGGGCGATATGTACATATTCTAGAGCTATACTCATATAATTAAACTAAACT

ATATTACTTTCAAATCCACTTTATAAAATAATGTTAATTATTTTAACCATCTAAATAATTTTATTGTAACCCATCTCTCCTTATCTATACGCTGTATCTT

GATCTGATTTTTTTTATACTTATAAATTTTGAACATTCCAAATTCTTTAAAAACATTCAACCTACGACGATATACAAACCTTTAAAATAAGTACGATTAA

TCGTGGATCATCAATTATAGGACAGGTTCCTCTGAGTAGACTAAAATACCGCCAAATTCTTAAAATTTCAAGAACATAACTACTACTATTCAAGCATCTA

AAATTTGCATTTTTAATAATAGGGTATCTAATCCTAGTTTTTTATAAAAATCTCATAAACTCATTTTTCACATTTAAAAAATTAATTATACTTACTAATT

TCACCTAATAAATATAATATAAATTAATAATAAAATAACTTATTATATACTGAACAAATTTAATTGCATTGTTTGTGTAACCGCAACTGCTGGCACAAAC

TTGGTCAATACTATTATAAATTCCTAAATCAAAATTTCTTTTAAATTTAATCTTCACTATTGCAATTCTTTAATTAAATATATAGAATAATTTATCTTTT

TAAAATAAATTCATTAAACACTAAAATTTACATATAAAATAATTTAAAAATTAAAATCTCAAGCTAGAATAAAACTTTATTTTTTTTTCAATATACATAA

ATTATATAAAATAAAAGTACCCCCC-TACCATTTTAATTAAGTGAATTAATTTTTAATGCCTTAAATTTATTTCCAATTTATTTTTATTATATAGTTTTT

TATTTAAAAATAAAACTTTATACCTAAAATTATATACATAAATTATATTTAAAATTTATAATTTAAAGAACTATAAAAATTAAATTAAAATTTAGAATCT

ATCTAATTTATAGATAAACAAATTAAATTAAACTAACTTACCTGATAAATTTAACTATATACGCGCAGGTATATTAATTATTTAATTATAATATAATACA

TTTGTTTTATTAAATAATTATTAAGCAAAATAGCATTAATTTATTATATAAAAGATCTAGATACTATTAATTTAAAGTAAATTTTTTTTTTTTTTGTAGT

ATAATTTATACTAATATACGGCATTTAATATTTTCTATATAAATGTTTATATTATTTATATTTATCTATTTATAATTCTTAATAATAATTTAGATAATTA

TTAATTTATATATTAAATATTTATATATATATATATAATAATAAGGATATATATATATATATGTATATAGATAAATTAAATTTAGTTATATATAAAAAAA

TTAATATTAATTTTACTTTTAATTTTTTCTTTAATTTAAACTATTGGATATTCAGATTCTAATATATTATAATATATCAACCATTAATGAATAACTTTAT

ATATATAATAAAAATGTTAATTAATATATCAATTCATATGAATATATAATATTAATAAATAAATTCTTAATAAATAATAATGATAATATATATATATTGT

ATATTAATGTACTATATATATAAATTCATTAAACATTTATATTTGATACCCCCCCAAAATTTTTTCTAAAAATGTGAAGTTTCAATTTAATTAAAATTTT

CTACAATGGTCAAAAACGGCCAAAAAATGGAAAATAAAATGTGCACAGAAAATGCACATAAAATGCACATTTTTGAAAATTTCCGATTTTTGAAAAACCG

TTTGAACCAAACCTCTGATGTACCTTTTTTCGCCCCCCCTGATTACTAAAATTTATCCTCAATTACTAAGGTTTATCCGCATTTTTGCGACAGGAATTTT

GAGTATAAAATCAATTCCACACTAAATTTTTTTTT-TTTTTTTTGTGTCATTTCTCCAACATGGGCAATTTTTTTTTTAGTCGAAGGACAAATTTTAGTA

AAAAAAGTTTGAAATTAAGTTAAACCCTTGTTTAACCAAAAACTATTACTTTTCAATATTATACCCAATTTTATTTAAAATAAATTTTATTACACTTTTA

TTTATGCAAAAAGATCTTCTTATAAACTTTT

>DMR180j_JP_Sapporo_Hokkaido

AATGAAGTGTCTGACTATAGAGTTACTTTGATAGAGTAAAAAAAGTGAATTTTCACCTTCATTATAATTAACAGAATTAAACTATTTCTTTAAGCTTCAA

AAACTTATGTACATTATATACTAAATTATAAAAAGATAAGCTAATTAAGCTACTGGGCTCATACCCCATCAATAAAGGTTACAATCCTTTTCTTTTTAAT

GTATTATAAGCTTTTATTTTTTAATTCTCTTATAATTGGAACCTTAATTGCTATTTCTTCCTACTCTTGGATAGGAATATGAATAGGATTAGAAATTAAT

CTTCTCTCTATGATCCCCCTCATTAGAGACAACAAAAATATAATAGCCTCAGAAGCTGCTTTAAAATATTTCATTATTCAAACAATAGCATCAACATTAT

TATTATTCTCAATTATTATAATATCAATGAAATTTATATATCAAATAAATTTAATCACTTACTTTAATTTAATTTTTAACACTTCATTATTCATCAAAAT

AGGAGCAGCCCCATTCCATTTTTGATTCCCCGAAATAATAGAAGGATTAAATTGATTAAATGCCATTATCATACTTACTTGACAAAAACTAAGGCCCATA

GTATTACTAACATATTCTAATACAACCTCCATATATCTAATTTTAACAATTATATTTAGAATAATAATCAGAGGAATTATAGGTCTAAATCAAACTAGAT

TACGGAAAATTATAGCTTATTCATCTATCAACCATATTGGATGAATAATCAGTTCAATTATATTAATTGAAATTGTTTGATTTTACTATTTTATTATTTA

TTGCATTATTACTATTAATATCAGAATTATATTTATAAAATTAAATGTTTTTCATATCAATCAATTATATATTTCAATAAATTACCATATTTTACTTAAA

TTATTCTTTGCCTTAAATTTCATGTCTTTAGGAGGATTACCCCCATTTTTAGGGTTTTTCCCTAAATGACTTACAATTCAAACTTTAATCCAAAGAAATA

TGTACTCAATTGCTTTCATTATAATTTTAATAACTTTAATAACACTTTTTTTTTACCTCCGAATTACTTTTTCAATTTTACTACTAAGAAAAACAGTTTT

AACATTTTACACACAACCAAAAATTTATACTAATTACATTATAGCATTTAATTTTATTGCATTATTAAGATTAATTTTCGTTACTTTGATATTCAATTTC

TTATAAATTAAACCTGAAGGATTTAAGTTAAATTAAACTAAGAACCTTCAAAGTTCTAAATAAAGTAAATTCTTTAAGCCTTAGAGCTTAGCCCATCTTT

AAATTTGCAATTTAAAATTCTTTTTGAACTATAAAGCTTGATAAAAGAAACTAATTTCGTATGTAAATTTACAGTTTACCGCCTAAACCTCGGCCATTTT

ATCGAATAAATGGCTATTCTCTACAAATCACAAAGATATCGGAACTTTATACTTTCTATTTGGAAGTTGAGCAGGCATAGTAGGCACTTCCTTAAGTTTA

CTGATTCGTGCTGAATTAGGAAACCCAGGATCTCTAATTGGAGATGATCAAATTTATAACGTAATCGTAACAGCTCATGCTTTCATTATAATTTTTTTTA

TAGTAATACCTATTATAATTGGAGGATTCGGAAACTGATTAGTTCCTTTAATGCTTGGAGCCCCCGACATAGCATTCCCCCGAATAAATAACATAAGATT

TTGACTTTTACCTCCTTCATTAACTCTACTTCTAATAAGAAGATTAGTCGAAAGAGGGGCAGGTACAGGCTGAACAGTTTATCCCCCACTATCAGCCAAT

ATCGCCCATAGAGGAGCATCAGTTGATTTAGCAATTTTTAGACTTCATCTTGCAGGAATTAGCTCAATTTTAGGTGCAGTAAATTTTATTACTACTGTAA

TTAACATACGATCAACAGGAATAACTTTTGATCGAATACCCCTATTTGTTTGATCTGTAGTTTTAACAGCACTTCTTCTGCTATTATCCCTCCCAGTTTT

AGCAGGAGCAATTACTATACTATTAACAGATCGAAATATTAATACAACATTTTTTGATCCCGCAGGTGGAGGAGACCCCATTCTCTACCAACATTTATTT

TGATTTTTTGGTCATCCAGAAGTTTACATTTTAATTTTACCTGGATTTGGTATAATCTCCCATATTATTAGCCAAGAAAGAAGAAAAAAAGAAACATTTG

GAACTTTAGGTATAATTTACGCTATAATAGCTATTGGTTTACTAGGATTTATTGTTTGAGCTCATCATATATTTACAGTAGGTATAGATGTAGACACACG

AGCATATTTTACATCAGCAACAATAATCATTGCTGTTCCTACAGGAATTAAAATTTTTAGATGATTAGCTACTCTCCATGGATCACAATTAAACTACTCC

CCGTCTCTTTTATGGGCATTAGGATTTGTATTCCTATTTACAGTAGGAGGATTAACAGGAGTAATTCTAGCTAATTCATCAATTGACATTATTTTACATG

ATACTTACTATGTAGTTGCACATTTCCATTATGTCCTTTCCATAGGAGCTGTATTTGCTATTATAGCAGGTTTTGTTCATTGATTCCCTTTATTTACAGG

TTTAACAATAAATTCAAAATTTCTTAAAATTCAATTTTTAACAATATTTATTGGTGTTAATATAACATTCTTCCCTCAACATTTCTTAGGATTAAGAGGA

ATACCTCGACGTTATTCAGATTACCCAGATGCTTATACAACTTGAAATATTATCTCATCTATTGGATCTTTAGTTTCTTTAATTAGTATTTTTATCTTTT

TATTTACTATTTGAGAAAGGCTAATTTCATTGCGAAAAAGAATTAGGTCTTTAAGAATATCTACATCAATTGAATGACTCCAACAAATACCCCCTTCAGA

ACATAGTTATTCTGAACTTCCAATGCTTACTAACTTCTAATATGGCAGATTAGTGCAATGGATTTAAACCCCAAATATAAAGATTAAACTTTTTTTAGAA

ATAGCTACTTGAAATACCATTTTACTTCAGGATAGGGCATCCCCATTAATAGAGCAACTCTCATTCTTTCATAACCATGCTCTTCTAATTCTCTTTATAA

TTACCGTTCTAGTAGGTTATTTAATAGGAACTTTATTTTTTAACCAATTTAATTACCGATTTTTATTAGATGGTCAAACTATTGAAATTATTTGAACTAT

TTTACCTGCTGTAACACTAATTTTTATTGCATTACCGTCTTTACGCTTACTTTATCTTCTAGATGAAGTTAATAACCCTTTAGTAACTATCAAAACAATT

GGGCATCAATGATATTGATCATACGAATATAGAGATTTTATAAATTTTGAATTCGATTCCTATATAATTCCTTTAACAGAAATAAAACCTCAAAATTTTC

GTTTATTAGATGTTGATAACCGAGTAATTGTCCCCTTTAACTCCCAAATCCGAATGATAGTAACAGCTGCCGATGTTATTCATTCATGAACTATCCCGGC

TTTTAGTGTAAAAATTGATGCAACACCAGGCCGACTAAATCAAATTAGATTCCTAATTAATCGAACAGGATTATTTTATGGTCAATGCTCAGAAATTTGT

GGGGCAAATCATAGATTTATACCTATTACTGTAGAAAGAATTTCACCTTCATTTTTTACTAAATGAATCTCAAAAATAAATAACCTATCATTAGATGACT

GAAAGTAAGTAATGGTCTCTTAAACCAATTAATAGTAGTTTAACATCTACTTCTGATGGCCAAAAATTTAGTTAAGATATAACATTAGTTTGTCATACTA

AAATAATCATAATTTGATAATTTTTAATTCCACAAATAGCACCTTTAAACTGACTATCTTTATTTTTTTTAATTATTATTATTTTTTTACTTTTTAATGT

ATTAAATTACTTTAGATTCTTACAGCCCTTAAAAACCCAATCTCATAACCCTACAATTAAAAAAATTAATTGAAAATGATAACTAATTTATTTTCATCTT

TTGATCCTAGAACTTCTTTTAATTTAAGATTAAACTGATTAAGAATACTATTAGGGCTAATATTTATCCCCCCAATATTTTGATTAGTTCCTTCACGCCA

TAATTTTCTATGAATTAAAATTATTTTAACATTACACCAAGAATTTAAGGTTTTAATTGGTAATAATAATATTAAAGGAAGAACCTTAATATTTATTTCA

TTATTTTCTATAATTGTTTTCAATAACTTTTTAGGATTATTTCCGTATATTTTTACAGGAACAAGACATTTAATTATAACATTATCTCTTGCCTTACCTT

TATGAATTAGATTCATATTATACGGGTGAATTAATAACACTATCCACATACTTGCTCATTTAGTTCCCCAAGGAACACCCCCAGCTCTTATGGCATTCAT

AGTAGTAATTGAATCAATTAGAAATATTATTCGTCCTGGTACTTTGGCTGTTCGATTAGCTGCTAATATAATTGCTGGACATTTACTAATAACTTTACTA

GGAAACACAGGATTAAATTTATCAATTTTTATACTAAGTATTCTTATTATCACACAAATTCTTTTATTAATTTTAGAATCTGCTGTTGCGATCATTCAAT

CTTATGTATTTGCTGTATTAAGAACTTTATACTCTAGAGAAATTAATTAATGTCAAGACATAAAAATCACCCCTATCATTTAGTTGATGCAAGACCTTGA

CCTATTTTAGGTGCTTTTAGAGCTATAATTACAATAATTGGAATTATCAAATGATTCCATTTTTATAATAATTCTTTATTTTACTTAGGGACATTAATCA

CAATTTTAATTATAATTCAATGATGACGAGATATCACTCGTGAGGGAACTTTCCAAGGACTTCATACTTACGCTGTAACTATAGGTTTACGTTGAGGAAT

AATTTTATTTATTACATCAGAAGTATTTTTCTTTATTTCTTTTTTTTGAGCCTTTTTTCATAGTAGTTTAACACCCGCTATTGAACTAGGAATACTCTGA

CCACCCAAAGGAATTACCCCATTTAACCCTATTCAAATTCCATTATTAAACACTTTAATTCTTTTAACTTCAGGATTAACTGTAACTTGAGCTCACCATA

GATTAATAGAAAATGACTATAACCAAACAATGCAAGGTCTTGGTTTAACAGTTTTACTAGGAGTATATTTTACTTTATTACAAGGTTACGAATATTTAGA

AGCCCCCTTTACTATAGCAGATTCTGTTTATGGATCAACATTTTTTATTGCTACTGGTTTCCATGGATTACATGTTATTATTGGCACAACCTTTTTAGCT

GTTTGTTTAATACGGCATTTTAATAACCATTTTACTTGTATCCATCACTTTGGATTTGAAGCTGCTGCTTGATACTGACATTTTGTTGATGTAGTATGAC

TATTTCTTTATATTTCTATTTACTGATGAGGTAGATATTTATATAGTATAATAATTATAATTGATTTCCAATCAAAAGATCTAAAAAA--TTAGTATAAA

TAATCATTATAATTTGAAATATAGGGTTAATTATTTTTTCTATCTCTTTTATCTTAATTATACTATCTTTTACAATCTCTAAAAAAAGATTTATAGACCG

AGAAAAAGCTTCTCCATTCGAGTGCGGATTTGACCCCAAAAGATCAGCCCGTTTACCCTTTTCTTTGCATTTTTTTTTAATTGCAGTAATTTTCTTAATT

TTTGATGTTGAAATTACTCTTCTTATCCCTTTAATTCTAACAATAAAAATTACTAATATTACTATATATACCTATATTGCTCTTTTCTTTTTAATGATTC

TTTTAATAGGACTTTACCATGAATGAAACCAAGGGGCCTTAAATTGAGCTCTTTAGGGTAATAGTTAAGTATAACATTTAAGTTGCATTTAAAAAGTATT

GATTTTTCAATTTACCTTAAATAAGAAACAATTAATTGTATTTAGTTTCGACCTAAAATTTAGGTGTATGAACACCCTTATTTAAATTAATTGAAACCAA

AAAGAGGTATATCACTGTTAATGATACTAATGAGAAAAACTCCAATTAAGGAAATAAGATATTCAAGAGTAAGCTTCTAACTTAACTCTTTAGCAGTGAA

AGTCTGTTAATATTTCTATTTATATAGTTTAATAAAACATTATTTTTTCATAATAAAATTAGAATAAATTTATTCTTATAAATATTTAAAAGTAAATTTT

ACTTCCCTGATAACTTCACTATCATACTCTATATAAGCTATTTAAATTAAATATATAAAATTATAAAAATTACCCATATTATAATTAAAAGTATAAAAAT

CTTATAATTGTTATTAAATATAAACTGTAAAAATACAGAAGTATTACTAATTTTACTATATAAATTTTGTCTTCCATAATACTCCGATCAGCCTTGATCA

ATAGTTTTATATAACTTACTACCTAATTTAATTGGATAATAATTTAAACCAAATGTTGAAATATAGGGCATATTCCACATAGAAGAAAAAAAAAGTCTTG

AATTTAACAAATAAAAAGATTTTAATCTATCATTTAAAGTAAACTTAGAAAGTTCAAACCCAAACCAAGCCCCAAAAAATGATACAATTAAAGCTATAAT

TTTTATTGTAAAAGGTAAACAAATAAAATAAGGTGTTGGGAATATCAACCATATTAATATTCTTCCCCCAACAATAACTAAAAAAATTAAACCTGATATC

CCTTGAAGTATAATTTTTCTATTATCATTAATTTTACTTAAAGAATAAAAACAAAAATTTCCTACCAAAACATAATAAATTAAACGAAATGTATAACAAA

CAGTTAATCCTGTTGAAAAAAAGAAAATAACATAAATATAGATATTTAAGTATCTTATAGATAAAACCTCTAAAATTAAATCCTTTGAATAAAACCCCGA

TAAAAAGGGTAAACCACATAATGCTAAATTTGAAATTATAAAATAAGTACAAGTTAAAGGTATAACTTTAATTAACCCCCCTATATACCGAATATCTTGA

CAATTTCTTAATCTGTGAATTATACACCCTGCGCATATAAATAATAAAGCCTTAAATAAAGCGTGAGTTAATAGATGAAAAAAGGCTAATTGATACTCCC

CTAAAGCTAAAATACTAATTATCAAACCCAATTGTCTTAAAGTAGACAGAGCAATAATTTTTTTTAAATCAAACTCAAAGTTTGCGCCTATTCCTGCTAT

AAATATAGTTATTGTTCCAATAAATAATAAAATTAATATTAAATTACTTGTTAATGCAAAATTAAAACGAATTAATAAGTAAACACCTGCTGTTACTAAG

GTAGAAGAATGAACTAAAGAAGAAACAGGTGTTGGAGCCGCTATTGCCGCAGGCAACCAAGAAGAAAATGGAATTTGAGCTCTTTTAGTTATAGCTGCTA

ATATAATTAATAAAATAATAATATATATTTCTATACTATTTTTATATACATCAATATAAAAAATATAGTTAAATCCCCCAAAATTTATTATTCATGCAAT

TGCTATTAATAAAGCAACATCTCCAATACGGTTAGTTAAAGCTGTAATTATCCCTGCATTATAAGATTTAATATTTTGATAATAAATAACTAAACAATAA

GAAACTAACCCTAACCCATCTCATCCTAATAAAATCCTAATTAAATTAGGAGAAATAATCAATAACATTATAGATAAAACAAATATAGAAACTAATATAA

TAAATCGATGCAAATAAATATCTCCTTCTATATACTCTTCACTATAGTAAATTACTATAGAAGAAATAAATAAAACAAAACTCATAAATAATAATGACAT

CCAGTCAAGTAAAATGGTTATAATAATTCTACAAGAATTAATCCTTAATATTTCATATTCTAATATTAGTCTATAATCTAAAATTATGAAATTTAAGCTT

AATAAAAATCTTAATACTCTAAAAAATAAAAACGTTACAAAATAAATTAAACAAATAGAAATAATTTAAAGTAAATTTTACATCTTTGATACCACAAATC

AATATTTTTTATTAAACTATTTAAATTACAATCATAAAACTAAAAATTCTCTCTTTAAAATTAAGATATTCAAGGGTAACCAATGAAGTAATATTAATAA

ATACTCACGAACAAACCCTCTTGAAAAAGAATATAAATTTCTAACTAACTTGCCGTGTTGACTATAAGAATATAAATATAAAGAATACGCAGCTCTAAAA

AAAGATATTAAAGATAAAAAAACCATTGTTCAACTCCTTCATCTAACTAATCTATTAATTAAAATAATCTCACCCAGTAAGTTTAATGAAGGAGGAGCCG

CTATATTACAACAACTAAATAAAAATCATCATATTCTTATTATTGGTATTAAATTGATTAGCCCCTTATTTAAATAAATACTTCGGCTATTTAGCCGTTC

ATAAGAAATATTTGCTAAACAAAATAATCCTGAAGAACATAAACCATGAGCAATTATTATAACTAAAGCCCCTCTTATACCCCAATAACTTAAAGTTAAA

ATCCCTCTTAATACTAAACCTATATGCGCTACAGAAGAATAAGCAATTAAAGCTTTAATATCAACTTGACGTAAACATATTAATGAAACAAAAAATCCCC

CTACTATTCTAATTGTAATAAAAATATAGTTTACTTGTAAACCTACAGTTAAAAAAATATTTATTAAACGTATTAATCCATACCCCCCTAATTTTAATAT

AACTCCAGCTAAAATTATAGAGCCAGCAACTGGAGCTTCAACATGGGCTTTAGGGAGCCAAAGATGAACAAAAAATATAGGCATTTTAATAAAAAACACT

ATATTTATACATATAAATAACAAGAATCTTTTTACATCATAACATAAAAAGAAAAAATCTAAAGAATGAAATTTTTCATAATAATAAAAAATCCTAATTA

TTATAGGTAATGAAGCAAATAATGTATAAAATAACAAGTAAACACCTGCTTGCAAACGCTCAGGTTGATACCCCCAACCAATAATTAATAAAAGAGTAGG

AATTAAGCTAAACTCAAAAAACAAATAAAAAATAAATAAATTTAAAGAACTAAATGTTATAATTAAAGATAATAATAAAATAATTATTACTAATAAAAAT

AAATTATAAAAATAGTTTTTTCTATAAATACTTTCAGAAGCTAATAATATTAAAGAACAAATCCACAAACTTAATAAAATTATTATAAAAGATAAAAGGT

CATACCCTATAAAATAAGAAATATTTATATACAAATAATTAAAACTAAATCTTAACCCAAATATAAATGTAATAAAAAAATATATATATTGATTAAATCA

GTATCTCTTTTTAATACAACTTAAAGGTACCAATATTAATATTATAAAAATAAACTTTATCATAATACATTAAAAGTTTGAAAATAATCATTTCCATGTG

TTCGTATTATAGAAACTAATAACGATAAACCTAAAGCTCCTTCACAAACTCTTATAGTTAAAAATACTATACCAAAATAAAATTCAAAATTAAAATATAT

TAAAAATAGATATAAATTAAAGTATAATCCTAAAATAATATACTCTAACCTTAATAATATTAAAAGTAAATGTTTACGTTTAATACAAAAAGAAACTAAT

CCTGTAAAATATATAATCACTGAAAATAATATACAAAAAATTAACATTAGTTTTAATAATTTAATAAAAATACTGGTCTTGTAAATCAGAAATAAGGATT

TTCTTTTAAAACTTCAGAGAAAGAGTAAACCTCTATCATTAATCTCCAAAATTAATATTTTAAATAAACTATTCTCTGTATAATCTTATTACTAATAACA

TTATCTTTTATTAGTTCAATAACTTTTATATTTTTAAGTCACCCTTTATCTATAGGGCTTATTTTATTAATACAAACTATTATTATAGCTTTAACTATAG

GTTTTTTTAATATTAATTTCTGATATTCTTATATTTTATTTCTTATTATAATTGGAGGAATATTAGTTTTATTTATTTATATAACAAGAGTAGCTTCAAA

TGAAAAATTTTCATTTTCTATTAAAATTACATTAATAATTAGAATTATAACTTTAGGATTTTTATTTAGTATTGCTATAATAGACCCCTACTTTTCAGAC

ATCAACTCAATTTACACAGAAAACTTAGATAATTATAAAGAATATAATATATCATTTAGAAAATATTTAAGTTATCCTAATATTATCATTATATACATAA

TAATTATTTATTTATTAATTACATTAATTGCAGTTGTAAAAATTACTCAAATTGAAAAAGGACCTTTACGTCAAACTAACTAATGAAAACACCATTACGA

AAAGCTTCCCCCCTATTAAAAATTATTAATAATAGAATTATTGATTTACCTACACCATCTAATATTTCTGCTTGATGAAATTTCGGATCCTTATTAGGAC

TCTGTCTTTTTATTCAAATTATCACTGGAATTTTCTTAGCTATACATTTTACAGCTCATATTGACATAGCATTTAATAGAGTAATTCATATTTGTCGAGA

TGTAAATTATGGCTGATTATTACGAACAATTCATGCTAACGGGGCTTCTTTTTTCTTTATTTGTATTTATCTACATATTGGCCGAGGAATATATTATAGA

AGCTATAATTTACATTTAACATGAACTATTGGAGTAATCATCCTTTTTATAGTAATAGCTACAGCATTCTTAGGGTATGTATTACCTTGAGGGCAAATAT

CTTTTTGAGGGGCTACTGTTATTACCAATCTTCTCTCTGCTATTCCTTATCTAGGAAATGTGATTGTCCAATGATTGTGGGGGGGATTTGCGGTAGATAA

CGCCACTTTAACTCGATTTTTTACATTACATTTTATTCTTCCTTTTATTATTTTAGCTTTAATAATTATTCATTTATTATTTCTTCATCAAACTGGTTCT

AATAACCCTTTAGGATTAAATAGAAATATCGATAAAGTACCTTTCCATCCATATTTTACTTACAAAGATACTTTTGGATTTATTATAATAACTATATTAT

TAATATTTTTAGTTTTAATTAATCCCTATCTTTTAGGAGACCCAGAAAACTTTACACCCGCTAACCCTTTAGTAACCCCAGTCCATATCCAACCAGAATG

ATACTTTTTATTCGCATACGCTATTTTACGATCAATTCCTAATAAATTAGGTGGAGTAATTGCTTTAGTTATATCAATTGCTATTTTATTAATTATACCT

TTAATTAACAAAAAAAAATTTAGTAGAACTCAATTTTACCCATTAAATAAAATTTTATTTTGATCTTTTGTCTCTATTGTAATCTTATTAACATGAATCG

GAGCTCGCCCAGTGGAAGATCCATACATTTTAACAGGTCAAATTTTAACAATTATATATTTTAGTTATTATTTCTTAAACCCCTTAATTCATAAAATATG

AGATTATATTATTTTTAAAACTTAGTTAATGAACTTGTTAAAGTGTATATTTTGAAAATATAAAAAAGAGTTTATTCTCTATTAACTTTACTAAATTTTA

TTCACTAAATAAAATAAGAAAAGATAGTCAACTTTAAACCTAAAAAAAAAAATAAAAAATTTAAAGAAACTGGTAAATAACTTTTTCAACATATATATAT

TAACTTATCATAACGATACCGAGGTAAAGTACCTCGAACTCAAATCCAAATAAATGCTATAAAAGTTAACTTCAAAAAAAAAAATAAAGAAAAAATATCT

CCCCCTATAAATAACAACACACATAATATACTTATAAATAAAATTCTAGCATACTCAGCCAAAAAGATTAAAGCAAATCCCCCTCTTCTGTACTCTACAT

TAAACCCAGAAACTAACTCTGATTCTCCTTCTGCAAAATCAAAAGGAGTCCGATTAGTCTCAGCTAAACTTGAAGATAATCATATTATTCTTAAAGGTAA

ACACAGAAAAAAAAATCATACTATTTCTTGATATTTTATAAAATCTATTATATTTAAATTTAAAATTAACAATAAAAAAGATAATAAAATTAAGGATAAA

CTTACTTCGTATGAAATTGTTTGAGCTACTGATCGAATACCCCCTAATAAAGCATAATTAGAATTTGATGATCAACCAGAAATTATGATAGTATAGACCC

TTAGCCTAGAACAACATAAAAAATATAAAATACCTAAATTAAACCTAAATATAAATGTCAAAAAAGGTATACATATCCATAATAAAAGAGCTAAAAATAA

ATTAAAAACTGGAGATATATAATAAATAAAAAAATTTGATATTAAAGGATAAGTTTGTTCTTTAGAAAATAACTTAATAGCATCACTAAAAGGTTGAGGA

ATACCTATAAATCCAACTTTATTGGGCCCTTTACGAATTTGGATATACCCTAAAACTTTGCGCTCTATTAATGTTAAAAAAGCTACACCAATTAAAACAC

AAATAATTAAAACTAAACTTGTAAATAATAAAAGAAATAAATCTTGTAATATAATGTATTACTTGTGTTAAACACATATTTAAATTCTAAATTTAAAGCA

CTAATCTGCCAAAGTAATATTCATATTCAAATTATATTAAATTTTAAAGGTATTTGATCCTTTCGTACTAAAATACCTATGTTTTTTAAAGATAGAAACC

AACCTGGCTCACGCCGGTTTAAACTCAGATCATGTAAAATTTTAAAGGTCGAACAGACCTAACCTTTTAGCCCCTACACCAAAAGTTAATTTTAATCCAA

CATCGAGGTCGCAAACTTTTTTTTCGATAAGAACTCTAAAAAAAA-TTACGCTGTTATCCCTAAGGTAATTTAATCTTGTAATCATTAAAAATGGATCAT

TCAATCATAAATTAATGTTTTTAAATAAAAAAAGTTTAATCAATTTTTCTGCTGCCCCAGCAAAATAGTTTAAATTATTAAATATATAAATATACTAAAA

TTAAATAATAATTTAAACTATAAAACTCTATAGGGTCTTCTCGTCTTTTAAAATTATATAAGCTTTTTTACTTATAAATAAAATTCTATATTCAATTAAA

TTGAGACAGTTACTTTCTCGTCCAACCGTTCATTCCAGCTTTCAATTAAAAAACTAATGATTATGCTACCTTTGCACGGTCAAATTACCGCGGCCATTCA

AATCCTCATTGGGCAGGTCAGACTTTAAATTATAATCAAAAAGACATGTTTTTAATAAACAGGCGAAAAGTGTATTTGCCGAGTTCCTTAATTTAACCTT

GAAGTTTTAATTTAATTACTAAATTAAAATATATACTAATTTTATCATTATTCTATATAAACCAATATTACATATATTATCTTAATAAACTACTTAAAAA

TAATATAAATCTTATTCTAACAAAAATTATTTATAACAAACTAAAGATTAACACTTCCAATTCTACTAATTTTTATTCAAAATATACATTTTTAACATTT

TATTTTAAAGCTTATCCCCTAAAATATTACTTTTTATATATAAAATACTAAATAATTAATATAATACAATAAAAAAACTAAATTAAATTTATTTCTTAAG

AAACTAGATATCTTAAAAAACGTATAACGTTTCATTTCTAATATAATATTTTAAAAATTTATGCCACAATTAAATTTATATTATATTAGCTCTTTATAAT

TCGAGAACACTAAATAATTAAATTATTTTAATAAACCCTGATACACAAGGTACAAAAAATTAATTTTTCTTTTTAAAAAATAAATCTCTATATATTTATA

TTATCTATCTCTATACAAATTAACTATAATAAAATTTTTATATTCTAAAATATACTAATATCAAAAATATTTTTTTTATATATATATATATA----TTAT

AAATTTTTCCTTTCAAATTAAATTGATTTTCACAACTAACTTTTTAATGTAAATAAAATGCTTTATTACAAGCTCTAATTTGCCATTCCAGGTACACTTT

CCAGTACACCTACTATGTTACGACTTATCCCTCTTTAGAGAGGGAGCGACGGGCGATATGTACATATTCTAGAGCTATACTCATATAATTAAACTAAACT

ATATTACTTTCAAATCCACTTTATAAAATAATGTTAATTATTTTAACCATCTAAATAATTTTATTGTAACCCATCTCTCCTTATCTATACGCTGTATCTT

GATCTGATTTTTTTTATACTTATAAATTTTGAACATTCCAAATTCTTTAAAAACATTCAACCTACGACGATATACAAACCTTTAAAATAAGTACGATTAA

TCGTGGATCATCAATTATAGGACAGGTTCCTCTGAGTAGACTAAAATACCGCCAAATTCTTAAAATTTCAAGAACATAACTACTACTATTCAAGCATCTA

AAATTTGCATTTTTAATAATAGGGTATCTAATCCTAGTTTTTTATAAAAATCTCATAAACTCATTTTTCACATTTAAAAAATTAATTATACTTACTAATT

TCACCTAATAAATACAATATAAATTAATAATAAAATAACTTATTATATACTGAACAAATTTAATTGCATTGTTTGTGTAACCGCAACTGCTGGCACAAAC

TTGGTCAATACTATTATAAATTCCTAAATCAAAATTTCTTTTAAATTTAATCTTCACTATTGCAATTCTTTAATTAAATATACAGAATAATTTATCTTTT

TAAAATAAATTCATTAAACACTAAAATTTACATATAAAATAATTTAAAAATTAAAATCTCAAGCTAGAATAAAACTTTATTTTTTTTTCAATATACATAA

ATTATATAAAATAAAAGTACCCCCC-TACTATTTTAATTAAGTGAATTAATTTTTAATGCCTTAAATTTATTTCCAATTTATTTTTATTATATAGTTTTT

TATTTAAAAATAAAACTTTATACCTAAAATTATATACATAAATTATATTTAAAATTTATAATTTAAAGAACTATAAAAATTAAATTAAAATTTAAAATCT

ATCTAATTTATAGATAAACAAATTAAATTAAACTAACTTACCTGATAAATTTAACTATATACGCGCAGGTATATTAATTATTTAATTATAATATAATACA

TTTGTTTTATTAAATAATTATTAAGCAAAATAGCATTAATTTATTATATAAAAGATCTAGATACTATTAATTTAAAGTAAATTTTTTTTTTTTTTGTAGT

ATAATTTATACTAATATACGGCATTTAATATCTTCTATATAAATGTTTATATTATTTATATTTATCTATTTATAATTCTTAATAATAATTTAGATAATTG

TTAATTTATATATTAAATATTTATATATATATATATAATAATAAGGATATATATATATATATGTATATAGATAAATTAAATTTAGTTATATATAAAAAAA

TTAATATTAATTTTACTTTTAATTTTTTCTTTAATTTAAACTATTGGATATTCAGATTCTAATATATTATAATATATCAACCGTTAATGAATAACTTTAT

ATATATAATAAAAATGTTAATTAATATATCAATTCATATGAATATATAATATTAATAAATAAATTCTTAATAAATAATAATGATAATATATATATATTGT

ATATTAATGTACTATATATATAAATTCATTAAACATTTATATTTGATACCCCCCCAAAATTTTTTCTAAAAATGTGAAGTTTCAATTTAATTAAAATTTT

CTACAATGGTCAAAAACGGCCAAAAAATGGAAAATAAAATGTGCACAGAAAATGCACATAAAATGCACATTTTTGAAAATTTCCGATTTTTGAAAAACCG

TTTGAACCAAACCTCTGATGTACCTTTTTTCGCCCCCCCTGATTACTAAAATTTATCCTCAATTACTAAGGTTTATCCGCATTTTTGCGACAGGAATTTT

GAGTATAAAATCAATTCCACACTAAATTTTTTTTT-TTTTTTTTGTGTCATTTCTCCAACATGGGCAATTTTTTTTTTAGTCGAAGGACAAATTTTAGTA

AAAAAAGTTTGAAATTAGGTTAAACCCTTGTTTAACCAAAAACTATTACTTTCCAATATTATACCCAATTTTATTTAAAATAAATTTTATTATACTTTTA

TTTATGCAAAAAGATCTTCTTATAAACTTTT

>DMR184j_JP_Horokanai_Hokkaido

AATGAAGTGTCTGACTATAGAGTTACTTTGATAGAGTAAAAAAAGTGAATTTTCACCTTCATTATAATTAACAGAATTAAACTATTTCTTTAAGCTTCAA

AAACTTATGTACATTATATACTAAATTATAAAAAGATAAGCTAATTAAGCTACTGGGCTCATACCCCATCAATAAAGGTTACAATCCTTTTCTTTTTAAT

GTATTATAAGCTTTTATTTTTTAATTCTCTTATAATTGGAACCTTAATTGCTATTTCTTCCTACTCTTGGATAGGAATATGAATAGGATTAGAAATTAAT

CTTCTCTCTATGATCCCCCTCATTAGAGACAACAAAAATATAATAGCCTCAGAAGCTGCTTTAAAATATTTCATTATTCAAACAATAGCATCAACATTGT

TATTATTCTCAATTATTATAATATCAATGAAATTTATATATCAAATAAATTTAATCACTTACTTTAATTTAATTTTTAACACTTCATTGTTCATCAAAAT

AGGAGCAGCCCCATTCCATTTTTGATTCCCCGAAATAATAGAAGGATTAAATTGATTAAATGCCATTATCATACTTACTTGACAAAAACTAAGGCCCATA

GTATTACTAACGTATTCTAATACAACCTCCATATATCTAATTTTAACAATTATATTTAGAATAATAATCAGAGGAATTATAGGTCTAAATCAAACTAGAT

TACGGAAAATTATAGCTTATTCATCTATCAACCATATTGGATGAATAATCAGTTCAATTATATTAATTGAAATTGTTTGATTTTACTATTTTATTATTTA

TTGCATTATTACTATTAATATCAGAATTATATTTATAAAATTAAATGTTTTTCATATCAATCAATTATATATTTCAATAAATTACCATATTTTACTTAAA

TTATTCTTTGCCTTAAATTTCATGTCTTTAGGAGGATTACCCCCATTTTTAGGGTTTTTCCCTAAATGACTTACAATTCAAACTTTAATCCAAAGAAATA

TGTACTCAATTGCTTTCATTATAATTTTAATAACTTTAATAACACTTTTTTTTTACCTCCGAATTACTTTTTCAATTTTACTACTAAGAAAAACAGTTTT

AACATTTTACACACAACCAAAAATTGATACTAATTACATTATAGCATTTAATTTTATTACATTATTAAGATTAATTTTCGTTACTTTGATATTCAATTTC

TTATAAATTAAACCTGAAGGATTTAAGTTAAATTAAACTAAGAACCTTCAAAGTTCTAAATAAAGTAAATTCTTTAAGCCTTAGGGCTTAGCCCATCTTT

AAATTTGCAATTTAAAATTCTTTTTGAACTATAAAGCTTGATAAAAGAAACTAATTTCGTATGTAAATTTACAGTTTACCGCCTAAACCTCGGCCATTTT

ATCGAATAAATGGCTATTCTCTACAAATCACAAAGATATCGGAACTTTATACTTTCTATTTGGAAGTTGAGCAGGCATAGTAGGCACTTCCTTAAGTTTA

CTGATTCGTGCTGAATTAGGAAACCCAGGATCTCTAATTGGAGATGATCAAATTTATAACGTAATCGTAACAGCTCATGCTTTCATTATAATTTTTTTTA

TAGTAATACCTATTATAATTGGAGGATTCGGAAACTGATTAGTTCCTTTAATGCTTGGAGCCCCCGACATAGCATTTCCCCGAATAAATAACATAAGATT

TTGACTTTTACCTCCTTCATTAACTCTACTTCTAATAAGAAGATTAGTCGAAAGAGGGGCAGGTACAGGCTGAACAGTTTATCCCCCACTATCAGCCAAT

ATCGCCCATAGAGGAGCATCAGTTGATTTAGCAATTTTTAGACTTCATCTTGCAGGAATTAGCTCAATTTTAGGTGCAGTAAATTTTATTACTACTGTAA

TTAACATACGATCAACAGGAATAACTTTTGATCGAATACCCCTATTTGTTTGATCTGTAGTTTTAACAGCACTTCTTCTGCTATTATCCCTCCCAGTTTT

AGCAGGAGCAATTACTATACTATTAACAGATCGAAATATTAATACAACATTTTTTGATCCCGCAGGTGGAGGAGACCCCATTCTCTACCAACATTTATTT

TGATTTTTTGGTCATCCAGAAGTTTACATTTTAATTTTACCTGGATTTGGTATAATCTCCCATATTATTAGCCAAGAAAGAAGAAAAAAAGAAACATTTG

GAACTTTAGGTATAATTTACGCTATAATAGCTATTGGTTTACTAGGATTTATTGTTTGAGCTCATCATATATTTACAGTAGGTATAGATGTAGACACACG

GGCATATTTTACATCAGCAACAATAATCATTGCTGTTCCTACAGGAATTAAAATTTTTAGATGATTAGCTACTCTCCATGGATCACAATTAAACTACTCC

CCGTCTCTTTTATGGGCATTAGGATTTGTATTCCTATTTACAGTAGGAGGATTAACAGGAGTAATTCTAGCTAATTCATCAATTGACATTATTTTACATG

ATACTTACTATGTAGTTGCACATTTCCATTATGTCCTTTCCATAGGAGCTGTATTTGCTATTATAGCAGGTTTTGTTCATTGATTCCCTTTATTTACAGG

TTTAACAATAAATTCAAAATTTCTTAAAATTCAATTTTTAACAATATTTATTGGTGTTAATATAACATTCTTCCCTCAACATTTCTTAGGATTAAGAGGA

ATACCTCGACGTTATTCAGATTACCCAGATGCTTATACAACTTGAAATATTATCTCATCTATTGGATCTTTAGTTTCTTTAATTAGTATTTTTATCTTTT

TATTTACTATTTGAGAAAGGCTAATTTCATTACGAAAAAGAATTAGATCTTTAAGAATATCTACATCAATTGAATGACTCCAACAAATACCCCCTTCAGA

ACATAGTTATTCTGAACTTCCAATGCTTACTAACTTCTAATATGGCAGATTAGTGCAATGGATTTAAACCCCAAATATAAAGATTAAACTTTTTTTAGAA

ATAGCTACTTGAAATACCATTTTACTTCAGGATAGGGCATCCCCATTAATAGAACAACTCTCATTCTTTCACAACCATGCTCTTCTAATTCTCTTTATAA

TTACCGTTCTAGTAGGTTATTTAATAGGAACTTTATTTTTTAACCAATTTAATTACCGATTTTTATTAGATGGTCAAACTATTGAAATTATTTGAACTAT

TTTACCTGCTGTAACACTAATTTTTATCGCATTACCGTCTTTACGCTTACTTTATCTTCTAGATGAAGTTAATAACCCTTTAGTAACTATCAAAACAATT

GGGCATCAATGATATTGATCATACGAATATAGAGATTTTATAAATTTTGAATTCGATTCCTATATAATTCCTTTGACAGAAATAAAACCTCAAAATTTTC

GTTTATTAGATGTTGATAACCGAGTAATTGTCCCCTTTAACTCCCAAATCCGAATGATAGTAACAGCTGCCGATGTTATTCATTCATGAACTATCCCGGC

TTTTAGTGTAAAAATTGATGCAACACCGGGCCGACTAAATCAAATTAGATTCCTAATTAATCGAACAGGATTATTTTATGGTCAATGCTCAGAAATTTGT

GGAGCAAATCATAGATTTATACCTATTACTGTAGAAAGAATTTCACCTTCATTTTTTACTAAATGAATCTCAAAAATAAATAACCTATCATTAGATGACT

GAAAGTAAGTAATGGTCTCTTAAACCAATTAATAGTAGTTTAACATCTACTTCTGATGGCCAAAAATTTAGTTAAGATATAACATTAGTTTGTCATACTA

AAATAATCATAATTTGATAATTTTTAATTCCACAAATAGCACCTTTAAACTGACTATCTTTATTTTTTTTAATTATTATTATTTTTTTACTTTTTAATGT

ATTAAATTACTTTAGATTCTTACAGCCCTTAAAAACCCAATCTCATAATCCTACAATTAAAAAAATTAATTGAAAATGATAACTAATTTATTTTCATCTT

TTGATCCTAGAACTTCTTTTAATTTAAGATTAAACTGATTAAGAATACTATTAGGGCTAATATTTATCCCCCCAATATTTTGATTAGTTCCTTCACGCCA

TAATTTTCTATGAATTAAAATTATTTTAACATTACACCAAGAATTTAAGGTTTTAATTGGTAATAATAATATTAAAGGAAGAACCTTAATATTTATTTCA

TTATTTTCTATAATTGTTTTCAATAACTTTTTAGGATTATTTCCGTATATTTTTACAGGAACAAGACATTTAATTATAACATTATCTCTTGCCTTACCTT

TATGAATTAGATTCATATTATATGGGTGAATTAATAACACTATCCACATACTTGCTCATTTAGTCCCCCAAGGAACACCCCCAGCTCTTATGGCATTCAT

AGTAGTAATTGAATCAATTAGAAATATTATTCGTCCTGGTACTTTAGCTGTTCGATTAGCTGCTAATATAATTGCTGGACATTTACTAATAACTTTACTA

GGAAACACAGGATTAAATTTATCAATTTTTATACTAAGTATTCTTATTATCACACAAATTCTTTTATTAATTTTAGAATCTGCTGTTGCGATCATTCAAT

CTTATGTATTTGCTGTATTAAGAACTTTATACTCTAGAGAAATTAATTAATGTCAAGACATAAAAATCACCCTTATCATTTAGTTGATGCAAGACCTTGA

CCTATTTTAGGTGCTTTTAGAGCTATAATTACAATAATTGGAATTATCAAATGATTCCATTTTTATAATAATTCTTTATTTTACTTAGGGACATTAATCA

CAATTTTAATTATAATTCAATGATGACGAGATATCACTCGTGAGGGAACTTTCCAAGGACTTCATACTTACGCTGTAACTATAGGTTTACGTTGAGGAAT

AATTTTATTTATTACATCAGAAGTATTTTTCTTTATTTCTTTTTTTTGAGCCTTTTTTCATAGTAGCTTAACACCCGCTATTGAACTAGGGATACTCTGA

CCACCCAAAGGAATTACCCCATTTAACCCTATTCAAATTCCATTATTAAACACTTTAATTCTTTTAACTTCAGGATTAACTGTAACTTGAGCTCACCATA

GATTAATAGAAAATGACTATAACCAAACAATGCAAAGTCTTGGTTTAACAGTTTTACTAGGAGTATATTTTACTTTATTACAAGGTTATGAATATTTAGA

AGCCCCCTTTACTATAGCAGATTCTGTTTATGGATCAACATTTTTTATTGCTACTGGTTTCCATGGATTACATGTTATTATTGGCACAACCTTTTTAGCT

GTTTGTTTAATACGGCATTTTAATAACCATTTTACTTGTATCCATCACTTTGGATTTGAAGCTGCTGCTTGATACTGACATTTTGTTGATGTAGTATGAC

TATTTCTTTATATTTCTATTTACTGATGAGGTAGATATTTATATAGTATAATAATTATAATTGATTTCCAATCAAAAGATCTAAAAAAA-TTAGTATAAA

TAATCATTATAATTTGAAATATAGGGTTAATTATTTTTTCTATCTCTTTTATCTTAATTATACTATCTTTTACAATCTCTAAAAAAAGATTTATAGACCG

AGAAAAAGCTTCTCCATTCGAGTGCGGATTTGACCCCAAAAGATCCGCCCGTTTACCCTTTTCTTTGCATTTTTTTTTAATTGCAGTAATTTTCTTAATT

TTTGATGTTGAAATTACTCTTCTTATCCCTTTAATTCTAACAATAAAAATTACTAATATTACTATATATACCTATATTGCTCTTTTCTTTTTAATGATTC

TTTTAATAGGACTTTACCATGAATGAAACCAAGGGGCCTTAAATTGAGCTCTTTAGGGTAATAGTTAAGTATAACATTTAAGTTGCATTTAAAAAGTATT

GATTTTTCAATTTACCTTAAATAAGAAACAATTAATTGTATTTAGTTTCGACCTAAAATTTAGGTGTATGAACACCCTTATTTAAATTAATTGAAACCAA

AAAGAGGTATATCACTGTTAATGATACTAATGAGAAAAACTCCAATTAAGGAAATAAGATATTCAAGAGTAAGCTTCTAACTTAACTCTTTAGCAGTGAA

AGTCTGTTAATATTTCTATTTATATAGTTTAATAAAACATTATTTTTTCATAATAAAATTAGAATAAATTTATTCTTATAAATATTTAAAAGTAAATTTT

ACTTCCCTGATAACTTCACTATCATACTCTATATAAGCTATTTAAATTAAATATATAAAATTATAAAAATTACCCATATTATAATTAAAAGTATAAAAAT

CTTATAATTGTTATTAAATATAAACTGTAAAAATACAGAAGTATTACTAATTTTACTATATAAATTTTGTCTTCCATAATACTCTGATCAGCCTTGATCA

ATAGTTTTATATAACTTACTACCTAATTTAATTGGATAATAATTTAAACCAAATGTTGAAATATAGGGTATATTCCACATAGAAGAAAAAAAAAGTCTTG

AATTTAACAAATAAAAAGATTTTAATCTATCATTTAAAGTAAACTTAGAAAGTTCAAACCCAAACCAAGCCCCAAAAAATGATACAATTAAAGCTATAAT

TTTTATTGTAAAAGGTAAACAAATAAAATAAGGTGTTGGGAATATCAACCATATTAATATTCTTCCCCCAACAATAACTAAAAAAATTAAACCTGATATC

CCTTGAAGTATAATTTTTCTATTATCATTAATTTTACTTAAAGAATAAAAACAAAAATTTCCTACCAATACATAATAAATTAAACGAAATGTATAACAAA

CAGTTAATCCTGTTGAAAAAAAGAAAATAACATAAATATAGATATTTAAGTATCTTATAGATAAAACCTCTAAAATTAAATCCTTTGAATAAAACCCCGA

TAAAAAGGGTAAACCACATAATGCTAAATTTGAAATTATAAAATAAGTACAAGTTAAAGGTATAACTTTAATTAACCCCCCTATATACCGAATATCTTGA

CAATTTCTTAATCTGTGAATTATACATCCTGCGCATATAAATAATAAAGCCTTAAATAAAGCGTGAGTTAATAGGTGAAAAAAAGCTAATTGATACTCCC

CTAAAGCTAAAATACTAATTATCAAACCCAATTGTCTTAAAGTAGACAGAGCAATAATTTTTTTTAAATCAAACTCAAAGTTTGCGCCTATTCCTGCTAT

AAATATAGTTATTGTTCCAATAAATAATAAAATTAATATTAAATTACTTGTTAATGCAAAATTAAAACGAATTAATAAGTAAACACCTGCTGTTACTAAG

GTAGAAGAATGAACTAAAGAAGAAACAGGTGTTGGAGCCGCTATTGCCGCAGGCAACCAAGAAGAAAATGGAATTTGAGCTCTTTTAGTTATAGCTGCTA

ATATAATTAATAAAATAATAATATATATTTCTATACTATTTTTATATACATCAATATAAAAAATATAGTTAAATCCCCCAAAATTTATTATTCATGCAAT

TGCTATTAATAAAGCAACATCTCCAATACGGTTAGTTAAAGCTGTAATTATCCCTGCATTATAAGATTTAATATTTTGATAATAAATAACTAAACAATAA

GAAACTAACCCTAACCCATCTCATCCTAATAAAATCCTAATTAAATTAGGAGAAATAATCAATAACATTATAGATAAAACAAATATAGAAACTAATATAA

TAAATCGATGCAAATAGATATCTCCTTCTATATACTCTTCACTATAGTAAATTACTATAGAAGAAATAAATAAAACAAAACTCATAAATAATAATGACAT

CCAGTCAAGTAAAATGGTTATAATAATTCTACAAGAATTAATCCTTAATATTTCATATTCTAATATTAGTCTATAATCTAAAATTATGAAATTTAAGCTT

AATAAAAATCTTAATACTCTAAAAAATAAAAACGTTACAAAATAAATTAAACAAATAGAAATAATTTAAAGTAAATTTTACATCTTTGATACCACAAATC

AATATTTTTTATTAAACTATTTAAATTACAATCATAAAACTAAAAATTCTCTCTTTAAAATTAAGATATTCAAGGGTAACCAATGAAGTAATAATAATAA

ATACTCACGAACAAACCCTCTTGAAAAAGAATATAAATTTCTAACTAACTTGCCGTGTTGACTATAAGAATATAAATATAAAGAATATGCAGCTCTAAAA

AAAGATATTAAAGATAAAAAACCCATTGTTCAACTCCTTCATCTAACTAATCTATTAATTAAAATAATCTCACCCAGTAAGTTTAATGAAGGAGGAGCCG

CTATATTACAACAACTAAATAAAAATCATCATATTCTTATTATTGGTATTAAATTGATTAGCCCCTTATTTAAATAAATACTTCGGCTATTTAACCGTTC

ATAAGAAATATTTGCTAAACAAAATAATCCTGAAGAACATAAACCATGGGCAATTATTATAACTAAAGCCCCTCTTATACCCCAATAACTTAAAGTTAAA

ATCCCTCTTAATACTAAACCTATATGCGCTACAGAAGAATAAGCAATTAAAGCTTTAATATCAACTTGACGTAAACATATTAATGAAACAAAAAATCCCC

CTACTATTCTAATTGTAATAAAAATATAGTTTACTTGTAAACCTACAGTTAAAAAAATATTTATTAAACGTATTAATCCATACCCCCCTAATTTTAATAT

AACTCCAGCTAAAATTATAGAGCCAGCAACTGGAGCTTCAACATGGGCTTTAGGGAGCCAAAGATGAACAAAAAATATAGGCATTTTAATAAAAAACACT

ATATTTATACATATAAATAACAAGAATCTTTTTACATCATAACATAAAAAGAAAAAATCTAAAGAATGAAATTTTTCATAATAATAAAAAATCCTAATTA

TTATAGGTAATGAAGCAAATAATGTATAAAATAACAAGTAAACACCTGCTTGCAAACGCTCAGGTTGATACCCCCAACCAATAATTAATAAAAGAGTGGG

AATTAAGCTAAACTCAAAAAACAAATAAAAAATAAATAAATTTAAAGAACTAAATGTTATAACTAAAGATAATAATAAAATAATTATTACTAATAAAAAT

AAATTATAAAAATAGTTTTTTCTATAAATACTTTCAGAAGCTAATAATATTAAAGAACAAATTCACAAACTTAATAAAATTATTATAAAAGATAAAAGGT

CATACCCTATAAAATAAGAAATATTTATATACAAATAATTAAAACTAAATCTTAACCCAAATATAAATGTAATAAAAAAATATATATATTGATTAAATCA

GTATCTCTTTTTAATACAACTTAAAGGTACCAATATTAATATTATAAAAATAAACTTTATCATAATACATTAAAAGTTTGAAAATAATCATTTCCATGTG

TTCGTATTATAGAAACTAATAACGATAAACCTAAAGCTCCTTCACAAACTCTTATAGTTAAAAATACTATACCAAAATAAAATTCAAAATTAAAATATAT

TAAAAATAGATATAAATTAAAGTATAATCCTAAAATAATATACTCTAACCTTAATAATATTAAAAGTAAATGTTTACGTTTAATACAAAAAGAAACTAAT

CCTGTAAAATATATAATCACTGAAAATAACATACAAAAAATTAACATTAGTTTTAATAATTTAATAAAAATACTGGTCTTGTAAATCAGAAATAAGGATT

TTCTTTTAAAACTTCAGAGAAAGAGTAAACCTCTATCATTAATCTCCAAAATTAATATTTTAAATAAACTATTCTCTGTATAATCTTATTACTAATAACA

TTATCTTTTATTAGTTCAATAACTTTTATATTTTTAAGTCACCCTTTATCTATAGGGCTTATTTTATTAATACAAACTATTATTATAGCTTTAACTATAG

GTTTTTTTAATATTAATTTCTGATATTCTTATATTTTATTTCTTATTATAATTGGAGGAATATTAGTTTTATTTATTTATATAACAAGAGTAGCTTCAAA

TGAAAAATTTTCATTTTCTATTAAAATTACATTAATAATTAGAATTATAACTTTAGGATTTTTATTCAGTATTGCTATAATAGACCCCTACTTTTCAGAC

ATCAACTCAATTTACACAGAAAACTTAGATAATTATAAAGAATATAATATATCATTTAGCAAATATTTAAGTTATCCTAATATTATCATTATATACATAA

TAATTATTTATTTATTAATTACATTAATTGCAGTTGTAAAAATTACTCAAATTGAAAAAGGACCTTTACGTCAAACTAACTAATGAAAACACCATTACGA

AAAGCTTCCCCCCTATTAAAAATTATTAATAATAGAATTATTGATTTACCTACACCATCTAATATTTCTGCTTGATGAAATTTCGGATCCTTATTAGGAC

TCTGTCTTTTTATTCAAATTATCACTGGAATTTTCTTAGCTATACATTTTACAGCCCATATTGACATAGCATTTAATAGAGTAATTCATATTTGTCGAGA

TGTAAATTATGGCTGATTATTACGAACAATTCATGCTAACGGGGCTTCTTTTTTCTTTATTTGTATTTATCTACATATTGGCCGAGGAATATATTATAGA

AGCTATAATTTACATTTAACATGAACTATTGGAGTAATCATCCTTTTTATAGTAATAGCTACAGCATTCTTAGGATATGTATTACCTTGAGGGCAAATAT

CTTTTTGAGGGGCCACTGTTATTACCAATCTTCTCTCTGCTATTCCTTATCTAGGAAATATGATTGTCCAATGATTATGGGGGGGATTTGCAGTAGATAA

CGCCACTTTAACTCGATTTTTTACATTACATTTTATTCTTCCTTTTATTATTTTAGCTTTAATAATTATTCATTTATTATTTCTTCATCAAACTGGTTCT

AATAACCCTTTAGGATTAAATAGAAATATCGATAAAGTACCTTTCCATCCATATTTTACTTACAAAGATACTTTTGGATTCATTATAATAACTATATTAT

TAATATTTTTAGTTTTAATTAATCCCTATCTTTTAGGAGACCCAGAAAACTTTACACCCGCTAACCCTTTAGTAACCCCAGTCCATATCCAACCAGAATG

ATACTTTTTATTCGCATACGCTATTTTACGATCAATTCCTAATAAATTAGGTGGAGTAATTGCTTTAGTTATATCAATTGCTATTTTATTAATTATACCT

TTAATTAACAAAAAAAAATTTAGTAGAACTCAATTTTACCCATTAAATAAAATTTTATTTTGATCTTTTGTCTCTATTGTAATCTTATTAACATGAATCG

GAGCTCGCCCAGTGGAAGATCCCTACATTTTAACAGGTCAAATTTTAACAATTATATATTTTAGTTATTATTTCTTAAACCCCTTAATTCATAAAATATG

AGATTATATTATTTTTAAAACTTAGTTAATGAACTTGTTAAAGTGTATATTTTGAAAATATAAAAAAGAGTTTATTCTCTATTAACTTTACTAAATTTTA

TTCACTAAATAAAATAAGAAAAGATAACCAACTTTAAACCTAAAAAAAAAAATAAAAAATTTAAAGAAACTGGTAAATAACTTTTTCAACATATATATAT

TAACTTATCATAACGGTACCGAGGTAAAGTACCTCGAACTCAAATCCAAATAAATGCTATAAAAGTTAACTTCAAAAAAAAAAATAAAGAAAAAATATCT

CCCCCTATAAATAACAACACACATAATATACTTATAAATAAAATTCTAGCATACTCAGCCAAAAAGATTAAAGCAAATCCCCCTCTTCTGTACTCTACAT

TAAACCCAGAAACTAACTCTGATTCTCCTTCTGCAAAATCAAAAGGAGTCCGATTAGTCTCAGCTAAACTTGAAGATAATCATATTATTCTTAAAGGTAA

GCACAGAAAAAAAAATCATACTATTTCTTGATATTTTATAAAATCTATTATATTTAAATTTAAAATTAACAATAAAAAAGATAATAAAATTAATGATAAA

CTTACTTCGTACGAAATTGTTTGAGCTACTGATCGAATACCCCCTAATAAAGCATAATTAGAATTTGATGATCAACCAGAAATTATGATAGTATAGACCC

TTAACCTAGAACAACATAAAAAATATAAAATACCTAAATTAAACCTAAATATAAATGTCAAAAAAGGTATACATATCCATAATAAAAGAGCTAAAAATAA

ATTAAAAACTGGAGATATATAATAAATAAAAAAATTTGATATTAAAGGATAAGTTTGTTCTTTAGAAAATAACTTAATAGCATCACTAAAAGGTTGAGGA

ATACCTATAAATCCAACTTTATTGGGCCCTTTACGAATTTGGATATACCCTAAAACTTTACGCTCTATTAATGTTAAAAAAGCTACACCAATTAAAACAC

AAATAATTAAAACTAAACTTGTAAATAATAAAAGAAATAAATCTTGTAATATAATGTATTACTTGTGTTAAACACATATTTAAATTCTAAATTTAAAGCA

CTAATCTGCCAAAGTAATATTCATATTCAAATTATATTAAATTTTAAAGGTATCTGATCCTTTCGTACTAAAATACCTATGTTTTTTAAAGATAGAAACC

AACCTGGCTCACGCCGGTTTAAACTCAGATCATGTAAAATTTTAAAGGTCGAACAGACCTAACCTTTTAGCCCCTACACCAAAAGTTAATTTTAATCCAA

CATCGAGGTCGCAAACTTTTTTTTCGATAAGAACTCTAAAAAAAA-TTACGCTGTTATCCCTAAGGTAATTTAATCTTGTAATCATTAAAAATGGATCAT

TCAATCATAAATTAATGTTTTTAAATAAAAAAAGTTTAATCAATTTTTCTGCTGCCCCAGCAAAATAGTTTAAATTATTAAATATATAAATATACTAAAA

TTAAATAATAATTTAAACTATAAAACTCTATAGGGTCTTCTCGTCTTTTAAAATTATATAAGCTTTTTTACTTATAAATAAAATTCTATATTCAATTAAA

TTGAGACAGTTACTTTCTCGTCCAACCGTTCATTCCAGCTTTCAATTAAAAAACTAATGATTATGCTACCTTTGCACGGTCAAATTACCGCGGCCATTCA

AATCCTCATTGGGCAGGTCAGACTTTAAATTATAATCAAAAAGACATGTTTTTAATAAACAGGCGAAAAGTGTATTTGCCGAGTTCCTTAATTTAACCTT

GAAGTTTTAATTTAATTACTAAATTAAAATATATACTAATTTTATCATTATTCTATATAAACCAATATTACATATATTATCTTAATAAACTACTTAAAAA

TAATATAAATCTTATTCTAACAAAAATTATTTATAACAAACTAAAGATTAACACTTCCAATTCTACTAATTTTTATTCAAAATATACATTTTTAACATTT

TATTTTAAAGCTTATCCCCTAAAATATTACTTTTTATATATAAAATACTAAATAATTAATATAATACAATAAAAAAACTAAATTAAATTTATTTCTTAAG

AAACTAGATATCTTAAAAAACGTATAACGTTTCATTTCTAATATAATATTTTAAAAATTTATGCCACAATTAAATTTATATTATATTAGCTCTTTATAAT

TCGAGAACACTAAATAATTAAATTATTTTAATAAACCCTGATACACAAGGTACAAAAAATTAATTTTTCTTTTTAAAAAATAAATCTCTATATATTTATA

TTATCTATCTCTATACAAATTAACTATAATAAAATTTTTATATTCTAAAATATACTAATATCAAAAATATTTTTTTTATATATATATATATA----TTAT

AAATTTTTCCTTTCAAATTAAATTGATTTTCACAACTAACTTTTTAATGTAAATAAAATGCTTTATTACAAGCTCTAATTTGCCATTCCAGGTACACTTT

CCAGTACACCTACTATGTTACGACTTATCCCTCTTTAGAGAGGGAGCGACGGGCGATATGTACATATTCTAGAGCTATACTCATATAATTAAACTAAACT

ATATTACTTTCAAATCCACTTTATAAAATAATGTTAATTATTTTAACCATCTAAATAATTTTATTGTAACCCATCTCTCCTTATCTATACGCTGTATCTT

GATCTGATTTTTTTTATACTTATAAATTTTGAACATTCCAAATTCTTTAAAAACATTCAACCTACGACGATATACAAACCTTTAAAATAAGTATGATTAA

TCGTGGATCATCAATTATAGGACAGGTTCCTCTGAGTAGACTAAAATACCGCCAAATTCTTAAAATTTCAAGAACATAACTACTACTATTCAAGCATCTA

AAATTTGCATTTTTAATAATAGGGTATCTAATCCTAGTTTTTTATAAAAATCTCATAAACTCATTTTTCACATTTAAAAAATTAATTATACTTACTAATT

TCACCTAATAAATACAATATAAATTAATAATAAAATAACTTATTATATACTGAACAAATTTAATTGCATTGTTTGTGTAACCGCAACTGCTGGCACAAAC

TTGGTCAATACTATTATAAATTCCTAAATCAAAATTTCTTTTAAATTTAATCTTCACTATTGCAATTCTTTAATTAAATATATAGAATAATTTATCTTTT

TAAAATAAATTCATTAAACACTAAAATTTACATATAAAATAATTTAAAAATTAAAATCTCAAGCTAGAATAAAACTTTATTTTTTTTTCAATATACATAA

ATTATATAAAATAAAAGTACCCCCC-TACCATTTTAATTAAGTGAATTAATTTTTAATGCCTTAAATTTATTTCCAATTTATTTTTATTATATAGTTTTT

TATTTAAAAATAAAACTTTATACCTAAAATTATATACATAAATTATATTTAAAATTTATAATTTAAAGAACTATAAAAATTAAATTAAAATTTAAAATCT

ATCTAATTTATAAATAAACAAATTAAATTAAACTAACTTACCTGATAAATTTAACTATATACGCGCAGGTATATTAATTATTTAATTATAATATAATACA

TTTGTTTTATTAAATAATTATTAAGCAAAATAGCATTAATTTATTATATAAAAGATCTAGGTACTATTAATTTAAAGTAAATTTTTTTTTTTTTTGTAGT

ATAATTTATACTAATATACGGCATTTAATATCTTCTATATAAATGTTTATATTATTTATATTTATCTATTTATAATTCTTAATAATAATTTAGATAATTA

TTAATTTATATATTAAATATTTATATATATATATATAATAATAAGGATATATATATATATATGTATATAGATAAATTAAATTTAGTTATATATAAAAAAA

TTAATATTAATTTTACTTTTAATTTTTTCTTTAATTTAAACTATTGGATATTCAGATTCTAATATATTATAATATATCAACCATTAATGAATAACTTTAT

ATATATAATAAAAATGTTAATTAATATATCAATTCATATGAATATATAATATTAATAAATAAATTCTTAATAAATAATAATGATAATATATATATATTGT

ATATTAATGTACTATATATATAAATTCATTAAACATTTATATTTGATACCCCCCCAAAATTTTTTCTAAAAATGTGAAGTTTCAATTTAATTAAAATTTT

CTACAATGGTCAAAAACGGCCAAAAAATGGAAAATAAAATGTGCACAGAAAATGCACATAAAATGCACATTTTTGAAAATTTCCGATTTTTGAAAAACCG

TTTGAACCAAACCTCTGATGTACCTTTTTTCGCCCCCCCTGATTACTAAAATTTATCCTCAATTACTAAGGTTTATCCGCATTTTTGCGACAGGAATTTT

GAGTATAAAATCAATTCCACACTAAATTTTTTTTT-TTTTTTTTGTGTCATTTCTCCAACATGGGCAATTTTTTTTTTAGTCGAAGGACAAATTTTAGTA

AAAAAAGTTTGAAATTAGGTTAAACCCTTGTTTAACCAAAAACTATTACTTTTCAATATTATACCCAATTTTATTTAAAATAAATTTTATTATACTTTTA

TTTATGCAAAAAGATCTTCTTATAAACTTTT

>DMR185j_JP_Horokanai_Hokkaido

AATGAAGTGTCTGACTATAGAGTTACTTTGATAGAGTAAAAAAAGTGAATTTTCACCTTCATTATAATTAACAGAATTAAACTATTTCTTTAAGCTTCAA

AAACTTATGTACATTATATACTAAATTATAAAAAGATAAGCTAATTAAGCTACTGGGCTCATACCCCATCAATAAAGGTTACAATCCTTTTCTTTTTAAT

GTATTATAAGCTTTTATTTTTTAATTCTCTTATAATTGGAACCTTAATTGCTATTTCTTCCTACTCTTGGATAGGAATATGAATAGGATTAGAAATTAAT

CTTCTCTCTATGATCCCCCTCATTAGAGACAACAAAAATATAATAGCCTCAGAAGCTGCTTTAAAATATTTCATTATTCAAACAATAGCATCAACATTGT

TATTATTCTCAATTATTATAATATCAATGAAATTTATATATCAAATAAATTTAATCACTTACTTTAATTTAATTTTTAACACTTCATTGTTCATCAAAAT

AGGAGCAGCCCCATTCCATTTTTGATTCCCCGAAATAATAGAAGGATTAAATTGATTAAATGCCATTATCATACTTACTTGACAAAAACTAAGGCCCATA

GTATTACTAACGTATTCTAATACAACCTCCATATATCTAATTTTAACAATTATATTTAGAATAATAATCAGAGGAATTATAGGTCTAAATCAAACTAGAT

TACGGAAAATTATAGCTTATTCATCTATCAACCATATTGGATGAATAATCAGTTCAATTATATTAATTGAAATTGTTTGATTTTACTATTTTATTATTTA

TTGCATTATTACTATTAATATCAGAATTATATTTATAAAATTAAATGTTTTTCATATCAATCAATTATATATTTCAATAAATTACCATATTTTACTTAAA

TTATTCTTTGCCTTAAATTTCATGTCTTTAGGAGGATTACCCCCATTTTTAGGGTTTTTCCCTAAATGACTTACAATTCAAACTTTAATCCAAAGAAATA

TGTACTCAATTGCTTTCATTATAATTTTAATAACTTTAATAACACTTTTTTTTTACCTCCGAATTACTTTTTCAATTTTACTACTAAGAAAAACAGTTTT

AACATTTTACACACAACCAAAAATTGATACTAATTACATTATAGCATTTAATTTTATTACATTATTAAGATTAATTTTCGTTACTTTGATATTCAATTTC

TTATAAATTAAACCTGAAGGATTTAAGTTAAATTAAACTAAGAACCTTCAAAGTTCTAAATAAAGTAAATTCTTTAAGCCTTAGGGCTTAGCCCATCTTT

AAATTTGCAATTTAAAATTCTTTTTGAACTATAAAGCTTGATAAAAGAAACTAATTTCGTATGTAAATTTACAGTTTACCGCCTAAACCTCGGCCATTTT

ATCGAATAAATGGCTATTCTCTACAAATCACAAAGATATCGGAACTTTATACTTTCTATTTGGAAGTTGAGCAGGCATAGTAGGCACTTCCTTAAGTTTA

CTGATTCGTGCTGAATTAGGAAACCCAGGATCTCTAATTGGAGATGATCAAATTTATAACGTAATCGTAACAGCTCATGCTTTCATTATAATTTTTTTTA

TAGTAATACCTATTATAATTGGAGGATTCGGAAACTGATTAGTTCCTTTAATGCTTGGAGCCCCCGACATAGCATTTCCCCGAATAAATAACATAAGATT

TTGACTTTTACCTCCTTCATTAACTCTACTTCTAATAAGAAGATTAGTCGAAAGAGGGGCAGGTACAGGCTGAACAGTTTATCCCCCACTATCAGCCAAT

ATCGCCCATAGAGGAGCATCAGTTGATTTAGCAATTTTTAGACTTCATCTTGCAGGAATTAGCTCAATTTTAGGTGCAGTAAATTTTATTACTACTGTAA

TTAACATACGATCAACAGGAATAACTTTTGATCGAATACCCCTATTTGTTTGATCTGTAGTTTTAACAGCACTTCTTCTGCTATTATCCCTCCCAGTTTT

AGCAGGAGCAATTACTATACTATTAACAGATCGAAATATTAATACAACATTTTTTGATCCCGCAGGTGGAGGAGACCCCATTCTCTACCAACATTTATTT

TGATTTTTTGGTCATCCAGAAGTTTACATTTTAATTTTACCTGGATTTGGTATAATCTCCCATATTATTAGCCAAGAAAGAAGAAAAAAAGAAACATTTG

GAACTTTAGGTATAATTTACGCTATAATAGCTATTGGTTTACTAGGATTTATTGTTTGAGCTCATCATATATTTACAGTAGGTATAGATGTAGACACACG

AGCATATTTTACATCAGCAACAATAATCATTGCTGTTCCTACAGGAATTAAAATTTTTAGATGATTAGCTACTCTCCATGGATCACAATTAAACTACTCC

CCGTCTCTTTTATGGGCATTAGGATTTGTATTCCTATTTACAGTAGGAGGATTAACAGGAGTAATTCTAGCTAATTCATCAATTGACATTATTTTACATG

ATACTTACTATGTAGTTGCACATTTCCATTATGTCCTTTCCATAGGAGCTGTATTTGCTATTATAGCAGGTTTTGTTCATTGATTCCCTTTATTTACAGG

TTTAACAATAAATTCAAAATTTCTTAAAATTCAATTTTTAACAATATTTATTGGTGTTAATATAACATTCTTCCCTCAACATTTCTTAGGATTAAGAGGA

ATACCTCGACGTTATTCAGATTACCCAGATGCTTATACAACTTGAAATATTATCTCATCTATTGGATCTTTAGTTTCTTTAATTAGTATTTTTATCTTTT

TATTTACTATTTGAGAAAGGCTAATTTCATTACGAAAAAGAATTAGATCTTTAAGAATATCTACATCAATTGAATGACTCCAACAAATACCCCCTTCAGA

ACATAGTTATTCTGAACTTCCAATGCTTACTAACTTCTAATATGGCAGATTAGTGCAATGGATTTAAACCCCAAATATAAAGATTAAACTTTTTTTAGAA

ATAGCTACTTGAAATACCATTTTACTTCAGGATAGGGCATCCCCATTAATAGAACAACTCTCATTCTTTCACAACCATGCTCTTCTAATTCTCTTTATAA

TTACCGTTCTAGTAGGTTATTTAATAGGAACTTTATTTTTTAACCAATTTAATTACCGATTTTTATTAGATGGTCAAACTATTGAAATTATTTGAACTAT

TTTACCTGCTGTAACACTAATTTTTATCGCATTACCGTCTTTACGCTTACTTTATCTTCTAGATGAAGTTAATAACCCTTTAGTAACTATCAAAACAATT

GGGCATCAATGATATTGATCATACGAATATAGAGATTTTATAAATTTTGAATTCGATTCCTATATAATTCCTTTGACAGAAATAAAACCTCAAAATTTTC

GTTTATTAGATGTTGATAACCGAGTAATTGTCCCCTTTAACTCCCAAATCCGAATGATAGTAACAGCTGCCGATGTTATTCATTCATGAACTATCCCGGC

TTTTAGTGTAAAAATTGATGCAACACCGGGCCGACTAAATCAAATTAGATTCCTAATTAATCGAACAGGATTATTTTATGGTCAATGCTCAGAAATTTGT

GGAGCAAATCATAGATTTATACCTATTACTGTAGAAAGAATTTCACCTTCATTTTTTACTAAATGAATCTCAAAAATAAATAACCTATCATTAGATGACT

GAAAGTAAGTAATGGTCTCTTAAACCAATTAATAGTAGTTTAACATCTACTTCTGATGGCCAAAAATTTAGTTAAGATATAACATTAGTTTGTCATACTA

AAATAATCATAATTTGATAATTTTTAATTCCACAAATAGCACCTTTAAACTGACTATCTTTATTTTTTTTAATTATTATTATTTTTTTACTTTTTAATGT

ATTAAATTACTTTAGATTCTTACAGCCCTTAAAAACCCAATCTCATAATCCTACAATTAAAAAAATTAATTGAAAATGATAACTAATTTATTTTCATCTT

TTGATCCTAGAACTTCTTTTAATTTAAGATTAAACTGATTAAGAATACTATTAGGGCTAATATTTATCCCCCCAATATTTTGATTAGTTCCTTCACGCCA

TAATTTTCTATGAATTAAAATTATTTTAACATTACACCAAGAATTTAAGGTTTTAATTGGTAATAATAATATTAAAGGAAGAACCTTAATATTTATTTCA

TTATTTTCTATAATTGTTTTCAATAACTTTTTAGGATTATTTCCGTATATTTTTACAGGAACAAGACATTTAATTATAACATTATCTCTTGCCTTACCTT

TATGAATTAGATTCATATTATATGGGTGAATTAATAACACTATCCACATACTTGCTCATTTAGTCCCCCAAGGAACACCCCCAGCTCTTATGGCATTCAT

AGTAGTAATTGAATCAATTAGAAATATTATTCGTCCTGGTACTTTAGCTGTTCGATTAGCTGCTAATATAATTGCTGGACATTTACTAATAACTTTACTA

GGAAACACAGGATTAAATTTATCAATTTTTATACTAAGTATTCTTATTATCACACAAATTCTTTTATTAATTTTAGAATCTGCTGTTGCGATCATTCAAT

CTTATGTATTTGCTGTATTAAGAACTTTATACTCTAGAGAAATTAATTAATGTCAAGACATAAAAATCACCCTTATCATTTAGTTGATGCAAGACCTTGA

CCTATTTTAGGTGCTTTTAGAGCTATAATTACAATAATTGGAATTATCAAATGATTCCATTTTTATAATAATTCTTTATTTTACTTAGGGACATTAATCA

CAATTTTAATTATAATTCAATGATGACGAGATATCACTCGTGAGGGAACTTTCCAAGGACTTCATACTTACGCTGTAACTATAGGTTTACGTTGAGGAAT

AATTTTATTTATTACATCAGAAGTATTTTTCTTTATTTCTTTTTTTTGAGCCTTTTTTCATAGTAGCTTAACACCCGCTATTGAACTAGGGATACTCTGA

CCACCCAAAGGAATTACCCCATTTAACCCTATTCAAATTCCATTATTAAACACTTTAATTCTTTTAACTTCAGGATTAACTGTAACTTGAGCTCACCATA

GATTAATAGAAAATGACTATAACCAAACAATGCAAAGTCTTGGTTTAACAGTTTTACTAGGAGTATATTTTACTTTATTACAAGGTTATGAATATTTAGA

AGCCCCCTTTACTATAGCAGATTCTGTTTATGGATCAACATTTTTTATTGCTACTGGTTTCCATGGATTACATGTTATTATTGGCACAACCTTTTTAGCT

GTTTGTTTAATACGGCATTTTAATAACCATTTTACTTGTATCCATCACTTTGGATTTGAAGCTGCTGCTTGATACTGACATTTTGTTGATGTAGTATGAC

TATTTCTTTATATTTCTATTTACTGATGAGGTAGATATTTATATAGTATAATAATTATAATTGATTTCCAATCAAAAGATCTAAAAAAA-TTAGTATAAA

TAATCATTATAATTTGAAATATAGGGTTAATTATTTTTTCTATCTCTTTTATCTTAATTATACTATCTTTTACAATCTCTAAAAAAAGATTTATAGACCG

AGAAAAAGCTTCTCCATTCGAGTGCGGATTTGACCCCAAAAGATCCGCCCGTTTACCCTTTTCTTTGCATTTTTTTTTAATTGCAGTAATTTTCTTAATT

TTTGATGTTGAAATTACTCTTCTTATCCCTTTAATTCTAACAATAAAAATTACTAATATTACTATATATACCTATATTGCTCTTTTCTTTTTAATGATTC

TTTTAATAGGACTTTACCATGAATGAAACCAAGGGGCCTTAAATTGAGCTCTTTAGGGTAATAGTTAAGTATAACATTTAAGTTGCATTTAAAAAGTATT

GATTTTTCAATTTACCTTAAATAAGAAACAATTAATTGTATTTAGTTTCGACCTAAAATTTAGGTGTATGAACACCCTTATTTAAATTAATTGAAACCAA

AAAGAGGTATATCACTGTTAATGATACTAATGAGAAAAACTCCAATTAAGGAAATAAGATATTCAAGAGTAAGCTTCTAACTTAACTCTTTAGCAGTGAA

AGTCTGTTAATATTTCTATTTATATAGTTTAATAAAACATTATTTTTTCATAATAAAATTAGAATAAATTTATTCTTATAAATATTTAAAAGTAAATTTT

ACTTCCCTGATAACTTCACTATCATACTCTATATAAGCTATTTAAATTAAATATATAAAATTATAAAAATTACCCATATTATAATTAAAAGTATAAAAAT

CTTATAATTGTTATTAAATATAAACTGTAAAAATACAGAAGTATTACTAATTTTACTATATAAATTTTGTCTTCCATAATACTCTGATCAGCCTTGATCA

ATAGTTTTATATAACTTACTACCTAATTTAATTGGATAATAATTTAAACCAAATGTTGAAATATAGGGTATATTCCACATAGAAGAAAAAAAAAGTCTTG

AATTTAACAAATAAAAAGATTTTAATCTATCATTTAAAGTAAACTTAGAAAGTTCAAACCCAAACCAAGCCCCAAAAAATGATACAATTAAAGCTATAAT

TTTTATTGTAAAAGGTAAACAAATAAAATAAGGTGTTGGGAATATCAACCATATTAATATTCTTCCCCCAACAATAACTAAAAAAATTAAACCTGATATC

CCTTGAAGTATAATTTTTCTATTATCATTAATTTTACTTAAAGAATAAAAACAAAAATTTCCTACCAATACATAATAAATTAAACGAAATGTATAACAAA

CAGTTAATCCTGTTGAAAAAAAGAAAATAACATAAATATAGATATTTAAGTATCTTATAGATAAAACCTCTAAAATTAAATCCTTTGAATAAAACCCCGA

TAAAAAGGGTAAACCACATAATGCTAAATTTGAAATTATAAAATAAGTACAAGTTAAAGGTATAACTTTAATTAACCCCCCTATATACCGAATATCTTGA

CAATTTCTTAATCTGTGAATTATACATCCTGCGCATATAAATAATAAAGCCTTAAATAAAGCGTGAGTTAATAGGTGAAAAAAAGCTAATTGATACTCCC

CTAAAGCTAAAATACTAATTATCAAACCCAATTGTCTTAAAGTAGACAGAGCAATAATTTTTTTTAAATCAAACTCAAAGTTTGCGCCTATTCCTGCTAT

AAATATAGTTATTGTTCCAATAAATAATAAAATTAATATTAAATTACTTGTTAATGCAAAATTAAAACGAATTAATAAGTAAACACCTGCTGTTACTAAG

GTAGAAGAATGAACTAAAGAAGAAACAGGTGTTGGAGCCGCTATTGCCGCAGGCAACCAAGAAGAAAATGGAATTTGAGCTCTTTTAGTTATAGCTGCTA

ATATAATTAATAAAATAATAATATATATTTCTATACTATTTTTATATACATCAATATAAAAAATATAGTTAAATCCCCCAAAATTTATTATTCATGCAAT

TGCTATTAATAAAGCAACATCTCCAATACGGTTAGTTAAAGCTGTAATTATCCCTGCATTATAAGATTTAATATTTTGATAATAAATAACTAAACAATAA

GAAACTAACCCTAACCCATCTCATCCTAATAAAATCCTAATTAAATTAGGAGAAATAATCAATAACATTATAGATAAAACAAATATAGAAACTAATATAA

TAAATCGATGCAAATAGATATCTCCTTCTATATACTCTTCACTATAGTAAATTACTATAGAAGAAATAAATAAAACAAAACTCATAAATAATAATGACAT

CCAGTCAAGTAAAATGGTTATAATAATTCTACAAGAATTAATCCTTAATATTTCATATTCTAATATTAGTCTATAATCTAAAATTATGAAATTTAAGCTT

AATAAAAATCTTAATACTCTAAAAAATAAAAACGTTACAAAATAAATTAAACAAATAGAAATAATTTAAAGTAAATTTTACATCTTTGATACCACAAATC

AATATTTTTTATTAAACTATTTAAATTACAATCATAAAACTAAAAATTCTCTCTTTAAAATTAAGATATTCAAGGGTAACCAATGAAGTAATAATAATAA

ATACTCACGAACAAACCCTCTTGAAAAAGAATATAAATTTCTAACTAACTTGCCGTGTTGACTATAAGAATATAAATATAAAGAATATGCAGCTCTAAAA

AAAGATATTAAAGATAAAAAACCCATTGTTCAACTCCTTCATCTAACTAATCTATTAATTAAAATAATCTCACCCAGTAAGTTTAATGAAGGAGGAGCCG

CTATATTACAACAACTAAATAAAAATCATCATATTCTTATTATTGGTATTAAATTGATTAGCCCCTTATTTAAATAAATACTTCGGCTATTTAACCGTTC

ATAAGAAATATTTGCTAAACAAAATAATCCTGAAGAACATAAACCATGGGCAATTATTATAACTAAAGCCCCTCTTATACCCCAATAACTTAAAGTTAAA

ATCCCTCTTAATACTAAACCTATATGCGCTACAGAAGAATAAGCAATTAAAGCTTTAATATCAACTTGACGTAAACATATTAATGAAACAAAAAATCCCC

CTACTATTCTAATTGTAATAAAAATATAGTTTACTTGTAAACCTACAGTTAAAAAAATATTTATTAAACGTATTAATCCATACCCCCCTAATTTTAATAT

AACTCCAGCTAAAATTATAGAGCCAGCAACTGGAGCTTCAACATGGGCTTTAGGGAGCCAAAGATGAACAAAAAATATAGGCATTTTAATAAAAAACACT

ATATTTATACATATAAATAACAAGAATCTTTTTACATCATAACATAAAAAGAAAAAATCTAAAGAATGAAATTTTTCATAATAATAAAAAATCCTAATTA

TTATAGGTAATGAAGCAAATAATGTATAAAATAACAAGTAAACACCTGCTTGCAAACGCTCAGGTTGATACCCCCAACCAATAATTAATAAAAGAGTGGG

AATTAAGCTAAACTCAAAAAACAAATAAAAAATAAATAAATTTAAAGAACTAAATGTTATAACTAAAGATAATAATAAAATAATTATTACTAATAAAAAT

AAATTATAAAAATAGTTTTTTCTATAAATACTTTCAGAAGCTAATAATATTAAAGAACAAATCCACAAACTTAATAAAATTATTATAAAAGATAAAAGGT

CATACCCTATAAAATAAGAAATATTTATATACAAATAATTAAAACTAAATCTTAACCCAAATATAAATGTAATAAAAAAATATATATATTGATTAAATCA

GTATCTCTTTTTAATACAACTTAAAGGTACCAATATTAATATTATAAAAATAAACTTTATCATAATACATTAAAAGTTTGAAAATAATCATTTCCATGTG

TTCGTATTATAGAAACTAATAACGATAAACCTAAAGCTCCTTCACAAACTCTTATAGTTAAAAATACTATACCAAAATAAAATTCAAAATTAAAATATAT

TAAAAATAGATATAAATTAAAGTATAATCCTAAAATAATATACTCTAACCTTAATAATATTAAAAGTAAATGTTTACGTTTAATACAAAAAGAAACTAAT

CCTGTAAAATATATAATCACTGAAAATAACATACAAAAAATTAACATTAGTTTTAATAATTTAATAAAAATACTGGTCTTGTAAATCAGAAATAAGGATT

TTCTTTTAAAACTTCAGAGAAAGAGTAAACCTCTATCATTAATCTCCAAAATTAATATTTTAAATAAACTATTCTCTGTATAATCTTATTACTAATAACA

TTATCTTTTATTAGTTCAATAACTTTTATATTTTTAAGTCACCCTTTATCTATAGGGCTTATTTTATTAATACAAACTATTATTATAGCTTTAACTATAG

GTTTTTTTAATATTAATTTCTGATATTCTTATATTTTATTTCTTATTATAATTGGAGGAATATTAGTTTTATTTATTTATATAACAAGAGTAGCTTCAAA

TGAAAAATTTTCATTTTCTATTAAAATTACATTAATAATTAGAATTATAACTTTAGGATTTTTATTCAGTATTGCTATAATAGACCCCTACTTTTCAGAC

ATCAACTCAATTTACACAGAAAACTTAGATAATTATAAAGAATATAATATATCATTTAGCAAATATTTAAGTTATCCTAATATTATCATTATATACATAA

TAATTATTTATTTATTAATTACATTAATTGCAGTTGTAAAAATTACTCAAATTGAAAAAGGACCTTTACGTCAAACTAACTAATGAAAACACCATTACGA

AAAGCTTCCCCCCTATTAAAAATTATTAATAATAGAATTATTGATTTACCTACACCATCTAATATTTCTGCTTGATGAAATTTCGGATCCTTATTAGGAC

TCTGTCTTTTTATTCAAATTATCACTGGAATTTTCTTAGCTATACATTTTACAGCCCATATTGACATAGCATTTAATAGAGTAATTCATATTTGTCGAGA

TGTAAATTATGGCTGATTATTACGAACAATTCATGCTAACGGGGCTTCTTTTTTCTTTATTTGTATTTATCTACATATTGGCCGAGGAATATATTATAGA

AGCTATAATTTACATTTAACATGAACTATTGGAGTAATCATCCTTTTTATAGTAATAGCTACAGCATTCTTAGGATATGTATTACCTTGAGGGCAAATAT

CTTTTTGAGGGGCCACTGTTATTACCAATCTTCTCTCTGCTATTCCTTATCTAGGAAATATGATTGTCCAATGATTATGGGGGGGATTTGCAGTAGATAA

CGCCACTTTAACTCGATTTTTTACATTACATTTTATTCTTCCTTTTATTATTTTAGCTTTAATAATTATTCATTTATTATTTCTTCATCAAACTGGTTCT

AATAACCCTTTAGGATTAAATAGAAATATCGATAAAGTACCTTTCCATCCATATTTTACTTACAAAGATACTTTTGGATTCATTATAATAACTATATTAT

TAATATTTTTAGTTTTAATTAATCCCTATCTTTTAGGAGACCCAGAAAACTTTACACCCGCTAACCCTTTAGTAACCCCAGTCCATATCCAACCAGAATG

ATACTTTTTATTCGCATACGCTATTTTACGATCAATTCCTAATAAATTAGGTGGAGTAATTGCTTTAGTTATATCAATTGCTATTTTATTAATTATACCT

TTAATTAACAAAAAAAAATTTAGTAGAACTCAATTTTACCCATTAAATAAAATTTTATTTTGATCTTTTGTCTCTATTGTAATCTTATTAACATGAATCG

GAGCTCGCCCAGTGGAAGATCCCTACATTTTAACAGGTCAAATTTTAACAATTATATATTTTAGTTATTATTTCTTAAACCCCTTAATTCATAAAATATG

AGATTATATTATTTTTAAAACTTAGTTAATGAACTTGTTAAAGTGTATATTTTGAAAATATAAAAAAGAGTTTATTCTCTATTAACTTTACTAAATTTTA

TTCACTAAATAAAATAAGAAAAGATAACCAACTTTAAACCTAAAAAAAAAAATAAAAAATTTAAAGAAACTGGTAAATAACTTTTTCAACATATATATAT

TAACTTATCATAACGGTACCGAGGTAAAGTACCTCGAACTCAAATCCAAATAAATGCTATAAAAGTTAACTTCAAAAAAAAAAATAAAGAAAAAATATCT

CCCCCTATAAATAACAACACACATAATATACTTATAAATAAAATTCTAGCATACTCAGCCAAAAAGATTAAAGCAAATCCCCCTCTTCTGTACTCTACAT

TAAACCCAGAAACTAACTCTGATTCTCCTTCTGCAAAATCAAAAGGAGTCCGATTAGTCTCAGCTAAACTTGAAGATAATCATATTATTCTTAAAGGTAA

GCACAGAAAAAAAAATCATACTATTTCTTGATATTTTATAAAATCTATTATATTTAAATTTAAAATTAACAATAAAAAAGATAATAAAATTAATGATAAA

CTTACTTCGTACGAAATTGTTTGAGCTACTGATCGAATACCCCCTAATAAAGCATAATTAGAATTTGATGATCAACCAGAAATTATGATAGTATAGACCC

TTAACCTAGAACAACATAAAAAATATAAAATACCTAAATTAAACCTAAATATAAATGTCAAAAAAGGTATACATATCCATAATAAAAGAGCTAAAAATAA

ATTAAAAACTGGAGATATATAATAAATAAAAAAATTTGATATTAAAGGATAAGTTTGTTCTTTAGAAAATAACTTAATAGCATCACTAAAAGGTTGAGGA

ATACCTATAAATCCAACTTTATTGGGCCCTTTACGAATTTGGATATACCCTAAAACTTTACGCTCTATTAATGTTAAAAAAGCTACACCAATTAAAACAC

AAATAATTAAAACTAAACTTGTAAATAATAAAAGAAATAAATCTTGTAATATAATGTATTACTTGTGTTAAACACATATTTAAATTCTAAATTTAAAGCA

CTAATCTGCCAAAGTAATATTCATATTCAAATTATATTAAATTTTAAAGGTATCTGATCCTTTCGTACTAAAATACCTATGTTTTTTAAAGATAGAAACC

AACCTGGCTCACGCCGGTTTAAACTCAGATCATGTAAAATTTTAAAGGTCGAACAGACCTAACCTTTTAGCCCCTACACCAAAAGTTAATTTTAATCCAA

CATCGAGGTCGCAAACTTTTTTTTCGATAAGAACTCTAAAAAAAAATTACGCTGTTATCCCTAAGGTAATTTAATCTTGTAATCATTAAAAATGGATCAT

TCAATCATAAATTAATGTTTTTAAATAAAAAAAGTTTAATCAATTTTTCTGCTGCCCCAGCAAAATAGTTTAAATTATTAAATATATAAATATACTAAAA

TTAAATAATAATTTAAACTATAAAACTCTATAGGGTCTTCTCGTCTTTTAAAATTATATAAGCTTTTTTACTTATAAATAAAATTCTATATTCAATTAAA

TTGAGACAGTTACTTTCTCGTCCAACCGTTCATTCCAGCTTTCAATTAAAAAACTAATGATTATGCTACCTTTGCACGGTCAAATTACCGCGGCCATTCA

AATCCTCATTGGGCAGGTCAGACTTTAAATTATAATCAAAAAGACATGTTTTTAATAAACAGGCGAAAAGTGTATTTGCCGAGTTCCTTAATTTAACCTT

GAAGTTTTAATTTAATTACTAAATTAAAATATATACTAATTTTATCATTATTCTATATAAACCAATATTACATATATTATCTTAATAAACTACTTAAAAA

TAATATAAATCTTATTCTAACAAAAATTATTTATAACAAACTAAAGATTAACACTTCCAATTCTACTAATTTTTATTCAAAATATACATTTTTAACATTT

TATTTTAAAGCTTATCCCCTAAAATATTACTTTTTATATATAAAATACTAAATAATTAATATAATACAATAAAAAAACTAAATTAAATTTATTTCTTAAG

AAACTAGATATCTTAAAAAACGTATAACGTTTCATTTCTAATATAATATTTTAAAAATTTATGCCACAATTAAATTTATATTATATTAGCTCTTTATAAT

TCGAGAACACTAAATAATTAAATTATTTTAATAAACCCTGATACACAAGGTACAAAAAATTAATTTTTCTTTTTAAAAAATAAATCTCTATATATTTATA

TTATCTATCTCTATACAAATTAACTATAATAAAATTTTTATATTCTAAAATATACTAATATCAAAAATATTTTTTTTATATATATATATATA----TTAT

AAATTTTTCCTTTCAAATTAAATTGATTTTCACAACTAACTTTTTAATGTAAATAAAATGCTTTATTACAAGCTCTAATTTGCCATTCCAGGTACACTTT

CCAGTACACCTACTATGTTACGACTTATCCCTCTTTAGAGAGGGAGCGACGGGCGATATGTACATATTCTAGAGCTATACTCATATAATTAAACTAAACT

ATATTACTTTCAAATCCACTTTATAAAATAATGTTAATTATTTTAACCATCTAAATAATTTTATTGTAACCCATCTCTCCTTATCTATACGCTGTATCTT

GATCTGATTTTTTTTATACTTATAAATTTTGAACATTCCAAATTCTTTAAAAACATTCAACCTACGACGATATACAAACCTTTAAAATAAGTATGATTAA

TCGTGGATCATCAATTATAGGACAGGTTCCTCTGAGTAGACTAAAATACCGCCAAATTCTTAAAATTTCAAGAACATAACTACTACTATTCAAGCATCTA

AAATTTGCATTTTTAATAATAGGGTATCTAATCCTAGTTTTTTATAAAAATCTCATAAACTCATTTTTCACATTTAAAAAATTAATTATACTTACTAATT

TCACCTAATAAATACAATATAAATTAATAATAAAATAACTTATTATATACTGAACAAATTTAATTGCATTGTTTGTGTAACCGCAACTGCTGGCACAAAC

TTGGTCAATACTATTATAAATTCCTAAATCAAAATTTCTTTTAAATTTAATCTTCACTATTGCAATTCTTTAATTAAATATATAGAATAATTTATCTTTT

TAAAATAAATTCATTAAACACTAAAATTTACATATAAAATAATTTAAAAATTAAAATCTCAAGCTAGAATAAAACTTTATTTTTTTTTCAATATACATAA

ATTATATAAAATAAAAGTACCCCCC-TACCATTTTAATTAAGTGAATTAATTTTTAATGCCTTAAATTTATTTCCAATTTATTTTTATTATATAGTTTTT

TATTTAAAAATAAAACTTTATACCTAAAATTATATACATAAATTATATTTAAAATTTATAATTTAAAGAACTATAAAAATTAAATTAAAATTTAAAATCT

ATCTAATTTATAAATAAACAAATTAAATTAAACTAACTTACCTGATAAATTTAACTATATACGCGCAGGTATATTAATTATTTAATTATAATATAATACA

TTTGTTTTATTAAATAATTATTAAGCAAAATAGCATTAATTTATTATATAAAAGATCTAGGTACTATTAATTTAAAGTAAATTTTTTTTTTTTTTGTAGT

ATAATTTATACTAATATACGGCATTTAATATCTTCTATATAAATGTTTATATTATTTATATTTATCTATTTATAATTCTTAATAATAATTTAGATAATTA

TTAATTTATATATTAAATATTTATATATATATATATAATAATAAGGATATATATATATATATGTATATAGATAAATTAAATTTAGTTATATATAAAAAAA

TTAATATTAATTTTACTTTTAATTTTTTCTTTAATTTAAACTATTGGATATTCAGATTCTAATATATTATAATATATCAACCATTAATGAATAACTTTAT

ATATATAATAAAAATGTTAATTAATATATCAATTCATATGAATATATAATATTAATAAATAAATTCTTAATAAATAATAATGATAATATATATATATTGT

ATATTAATGTACTATATATATAAATTCATTAAACATTTATATTTGATACCCCCCCAAAATTTTTTCTAAAAATGTGAAGTTTCAATTTAATTAAAATTTT

CTACAATGGTCAAAAACGGCCAAAAAATGGAAAATAAAATGTGCACAGAAAATGCACATAAAATGCACATTTTTGAAAATTTCCGATTTTTGAAAAACCG

TTTGAACCAAACCTCTGATGTACCTTTTTTCGCCCCCCCTGATTACTAAAATTTATCCTCAATTACTAAGGTTTATCCGCATTTTTGCGACAGGAATTTT

GAGTATAAAATCAATTCCACACTAAATTTTTTTTT-TTTTTTTTGTGTCATTTCTCCAACATGGGCAATTTTTTTTTTAGTCGAAGGACAAATTTTAGTA

AAAAAAGTTTGAAATTAGGTTAAACCCTTGTTTAACCAAAAACTATTACTTTTCAATATTATACCCAATTTTATTTAAAATAAATTTTATTATACTTTTA

TTTATGCAAAAAGATCTTCTTATAAACTTTT

>DMR72j_JP_Nanae_Hokkaido

AATGAAGTGTCTGACTATAGAGTTACTTTGATAGGGTAAAAAAAGTGAATTTTCACCTTCATTATAATTAACAGAATTAAACTATTTCTTTAAGCTTCAA

AAACTTATGTACATTATATACTAAATTATAAAAAGATAAGCTAATTAAGCTACTGGGCTCATACCCCATCAATAAAGGTTACAATCCTTTTCTTTTTAAT

GTATTATAAGCTTTTATTTTTTAATTCTCTTATAATTGGAACCTTAATTGCTATTTCTTCCTACTCTTGAATAGGAATATGAATAGGATTAGAAATTAAT

CTTCTCTCTATGATCCCCCTCATTAGAGACAACAAAAATATAATAGCCTCAGAAGCTGCTTTAAAATATTTCATTATTCAAACAATAGCATCAACATTGT

TATTATTCTCAATTATTATAATATCAATGAAATTTATATATCAAATAAATTTAATCACTTACTTTAATTTAATTTTTAACACTTCATTGTTCATCAAAAT

AGGAGCAGCCCCATTCCATTTTTGATTCCCCGAAATAATAGAAGGATTAAATTGATTAAATGCCATTATCATACTTACTTGACAAAAACTAAGGCCCATA

GTATTACTAACGTATTCTAATACAACCTCCATATATCTAATTTTAACAATTATATTTAGAATAATAATCAGAGGAATTATAGGCCTAAATCAAACTAGAT

TACGGAAAATTATAGCTTATTCATCTATCAACCATATTGGATGAATAATCAGTTCAATTATATTAATTGAAATTGTTTGATTTTACTATTTTATTATTTA

TTGCATTATTACTATTAATATCAGAATTATATTTATAAAATTAAATGTTTTTCATATCAATCAATTATATATTTCAATAAATTACCATATTTTACTTAAA

TTATTCTTTGCCTTAAATTTCATGTCTTTAGGAGGATTACCCCCATTTTTAGGGTTTTTCCCTAAATGACTTACAATTCAAACTTTAATCCAAAGAAATA

TGTACTCAATTGCTTTCATTATAATTTTAATAACTTTAATAACACTTTTTTTTTACCTCCGAATTACTTTTTCAATTTTACTACTAAGAAAAACAGTTTT

AACATTTTATACACAACCAAAAATTGATACTAATTACATTATAGCATTTAATTTTATTACATTATTAAGATTAATTTTCGTTACTTTGATATTCAATTTC

TTATAAATTAAACCTGAAGGATTTAAGTTAAATTAAACTAAGAACCTTCAAAGTTCTAAATAAAGTAAATTCTTTAAGCCTTAGGGCTTAGCCCATCTTT

AAATTTGCAATTTAAAATTCTTTTTGAACTATAAAGCTTGATAAAAGAAACTAATTTCGTATGTAAATTTACAGTTTACCGCCTAAACCTCGGCCATTTT

ATCGAATAAATGGCTATTCTCTACAAATCACAAAGATATCGGAACTTTATACTTTCTATTTGGAAGTTGAGCAGGAATAGTAGGCACTTCCTTAAGTTTA

CTGATTCGTGCTGAATTGGGAAACCCAGGATCTCTAATTGGAGATGATCAAATTTATAACGTAATCGTAACAGCTCATGCTTTCATTATAATTTTTTTTA

TAGTAATACCTATTATAATTGGAGGATTCGGAAACTGATTAGTTCCTTTAATGCTTGGAGCCCCCGACATAGCATTCCCCCGAATAAATAACATAAGATT

TTGACTTTTACCTCCTTCATTAACTCTACTTCTAATAAGAAGATTAGTCGAAAGAGGGGCAGGTACAGGCTGAACAGTTTATCCCCCACTATCAGCCAAT

ATCGCCCATAGAGGAGCATCAGTTGATTTAGCAATTTTTAGACTTCATCTTGCAGGAATTAGCTCAATTTTAGGTGCAGTAAATTTTATTACTACTGTAA

TTAACATACGATCAACAGGAATAACTTTTGATCGAATACCCCTATTTGTTTGATCTGTAGTTTTAACAGCACTTCTTCTGCTATTATCCCTCCCAGTTTT

AGCAGGAGCAATTACTATACTATTAACAGATCGAAATATTAATACAACATTTTTTGATCCCGCAGGTGGAGGAGACCCCATTCTCTACCAACATTTATTT

TGATTTTTTGGTCATCCAGAAGTTTACATTTTAATTTTACCTGGATTTGGTATAATCTCCCATATTATTAGCCAAGAAAGAAGAAAAAAAGAAACATTTG

GAACTTTAGGTATAATTTACGCTATAATAGCTATTGGTTTACTAGGATTTATTGTTTGGGCTCATCATATATTTACAGTAGGTATAGATGTAGACACACG

GGCATATTTTACATCAGCAACAATAATCATTGCTGTTCCTACAGGAATTAAAATTTTTAGATGATTAGCTACTCTCCATGGATCACAATTAAACTACTCC

CCGTCTCTTTTATGGGCATTAGGATTTGTATTCCTATTTACAGTAGGAGGATTAACAGGGGTAATTCTAGCTAATTCATCAATTGACATTATTTTACATG

ATACTTACTATGTAGTTGCACATTTCCATTATGTCCTTTCCATAGGAGCTGTATTTACTATTATAGCAGGTTTTGTTCATTGATTCCCTTTATTTACAGG

TTTAACAATAAATTCAAAATTTCTTAAAATTCAATTTTTAACAATATTTATTGGTGTTAATATAACATTCTTCCCTCAACATTTCTTAGGATTAAGAGGA

ATACCTCGACGTTATTCAGATTACCCAGATGCTTACACAACTTGAAATATTATCTCATCTATTGGATCTTTAGTTTCTTTAATTAGTATTTTTATCTTTT

TATTTACTATTTGAGAAAGGCTAATTTCATTACGAAAAAGAATTAGGTCTTTAAGAATATCTACATCAATTGAATGACTCCAACAAATACCCCCTTCAGA

ACATAGTTATTCGGAACTTCCAATGCTTACTAACTTCTAATATGGCAGATTAGTGCAATGGATTTAAACCCCAAATATAAAGATTAAACTTTTTTTAGAA

ATAGCTACTTGAAATACCATTTTACTTCAGGATAGGGCATCCCCATTAATAGAGCAACTCTCATTCTTTCATAACCATGCTCTTCTAATTCTCTTTATAA

TTACCGTTCTAGTAGGTTATTTAATAGGAACTTTATTTTTTAACCAATTTAATTACCGATTTTTATTAGATGGTCAAACTATTGAAATTATTTGAACTAT

TTTACCTGCTGTAACACTAATTTTTATCGCATTACCGTCTTTACGCTTACTTTATCTTCTAGATGAAGTTAATAACCCTTTAGTAACTATCAAAACAATT

GGGCATCAATGATATTGATCATACGAATATAGAGATTTTATAAATTTTGAATTCGATTCCTATATAATTCCTTTGACAGAAATAAAACCTCAAAATTTTC

GTTTATTAGATGTTGATAACCGAGTAATTGTCCCCTTTAACTCCCAAATCCGAATGATAGTAACAGCTGCCGATGTTATTCATTCATGAACTATCCCGGC

TTTTAGTGTAAAAATTGATGCAACACCGGGCCGACTAAATCAAACTAGATTCCTAATTAATCGAACAGGATTATTTTATGGTCAATGCTCAGAAATTTGT

GGAGCAAATCATAGATTTATACCTATTACTGTAGAAAGAATTTCACCTTCATTTTTTACTAAATGAATCTCAAAAATAAATAACCTATCATTAGATGACT

GAAAGTAAGTAATGGTCTCTTAAACCAATTAATAGTAGTTTAACATCTACTTCTGATGGCCAAAAATTTAGTTAAGATATAACATTAGTTTGTCATACTA

AAATAATCATAATTTGATAATTTTTAATTCCACAAATAGCACCTTTAAACTGACTATCTTTATTTTTTTTAATTATTATTATTTTTTTACTTTTTAATGT

ATTAAATTACTTTAGATTCTTACAGCCCTTAAAAACCCAATCTCATAACCCTACAATTAAAAAAATTAATTGAAAATGATAACTAATTTATTTTCATCTT

TTGATCCTAGAACTTCTTTTAATTTAAGATTAAACTGATTAAGAATACTATTAGGGCTAATATTTATCCCCCCAATATTTTGATTAGTTCCTTCACGCCA

TAATTTTCTATGAATTAAAATTATTTTAACATTACACCAAGAATTTAAGGTTTTAATTGGTAATAATAATATTAAAGGAAGAACCTTAATATTTATTTCA

TTATTTTCTATAATTGTTTTCAATAACTTTTTAGGATTATTTCCGTATATTTTTACAGGAACAAGACATTTAATTATAACATTATCTCTTGCCTTACCTT

TATGAATTAGATTCATATTATACGGGTGAATTAATAACACTATCCACATACTTGCTCATTTAGTTCCCCAAGGAACACCCCCAGCTCTTATGGCATTCAT

AGTAGTAATTGAATCAATTAGAAATATTATTCGTCCTGGTACTTTAGCTGTTCGATTAGCTGCTAATATAATTGCTGGACATTTACTAATAACTTTACTA

GGAAACACAGGATTAAATTTATCAATTTTTATACTAAGTATTCTTATTATCACACAAATTCTTTTATTAATTTTAGAATCTGCTGTTGCGATCATTCAAT

CTTATGTATTTGCTGTATTAAGAACTTTATACTCTAGAGAAATTAATTAATGTCAAGACATAAAAATCACCCTTATCATTTAGTTGATGCAAGACCTTGA

CCTATTTTAGGTGCTTTTAGAGCTATAATTACAATAATTGGAATTATCAAATGATTCCATTTTTATAATAATTCTTTATTTTACTTAGGGACATTAATCA

CAATTTTAATTATAATTCAATGATGACGAGATATCACTCGTGAGGGAACTTTCCAAGGACTTCATACTTACGCTGTAACTATAGGTTTACGTTGAGGAAT

AATTTTATTTATTACATCAGAAGTATTTTTCTTTATTTCTTTTTTTTGAGCCTTTTTTCATAGTAGATTAACACCCGCTATTGAACTAGGGATACTCTGA

CCACCCAAAGGAATTACCCCATTTAACCCAATTCAAATTCCATTATTAAACACTTTAATTCTTTTAACTTCAGGATTAACTGTAACTTGAGCTCACCATA

GATTAATAGAAAATGACTATAACCAAACAATGCAAGGTCTTGGTTTAACAGTTTTACTAGGAGTATATTTTACTTTATTACAAGGTTACGAATATTTAGA

AGCCCCCTTTACTATAGCAGATTCTGTTTATGGATCAACATTTTTTATTGCTACTGGTTTCCACGGATTACATGTTATTATTGGCACAACCTTTTTAGCT

GTTTGTTTAATACGACATTTTAATAACCATTTTACTTGTATCCATCACTTTGGATTTGAAGCTGCTGCTTGATACTGACATTTTGTTGATGTAGTATGAC

TATTTCTTTATATTTCTATTTACTGATGAGGTAGATATTTATATAGTATAATAATTATAATTGATTTCCAATCAAAAGATCTAAAAAAA-TTAGTATAAA

TAATCATTATAATTTGAAATATAGGGTTAATTATTTTTTCTATCTCTTTTATCTTAATTATACTATCTTTTACAATCTCTAAAAAAAGATTTATAGACCG

AGAAAAAGCTTCTCCATTCGAGTGCGGATTTGACCCCAAAAGATCAGCCCGTTTACCCTTTTCTTTGCATTTTTTTTTAATTGCAGTAATTTTCTTAATT

TTTGATGTTGAAATTACTCTTCTTATCCCTTTAATTCTAACAATAAAAATTACTAATATTACTATATATACCTATATTGCTCTTTTCTTTTTAATGATTC

TTTTAATAGGACTTTACCATGAATGAAACCAAGGAGCCTTAAATTGAGCTCTTTAGGGTAATAGTTAAGTATAACATTTAAGTTGCATTTAAAAAGTATT

GATTTTTCAATTTACCTTAAATAAGAAACAATTAATTGTATTTAGTTTCGACCTAAAATTTAGGTGTATGAACACCCTTATTTAAATTAATTGAAACCAA

AAAGAGGTATATCACTGTTAATGATACTAATGAGAAAAACTCCAATTAAGGAAATAAGATATTCAAGAGTAAGCTTCTAACTTAACTCTTTAGCAGTGAA

AGTCTGTTAATATTTCTATTTATATAGTTTAATAAAACATTATTTTTTCATAATAAAATTAGAATAAATTTATTCTTATAAATATTTAAAAGTAAATTTT

ACTTCCCTGATAACTTCACTATCATACTCTATATAAGCTATTTAAATTAAATATATAAAATTATAAAAATTACCCATATTATAATTAAAAGTATAAAAAT

CTTATAATTGTTATTAAATATAAACTGTAAAAATACAGAAGTATTACTAATTTTACTATATAAATTTTGTCTTCCATAATACTCTGATCAGCCTTGATCA

ATAGTTTTATATAACTTACTACCTAATTTAATTGGATAATAATTTAAACCAAATGTTGAAATATAGGGTATATTCCACATAGAAGAAAAAAAAAGTCTTG

AATTTAACAAATAAAAAGATTTTAATCTATCATTTAAAGTAAACTTAGAAAGCTCAAACCCAAACCAAGCCCCAAAAAATGATACAATTAAAGCTATAAT

TTTTATTGTAAAAGGTAAACAAATAAAATAAGGTGTTGGGAATATCAACCATATTAATATTCTTCCCCCAACAATAACTAAAAAAATTAAACCTGATATC

CCTTGAAGTATAATTTTTCTATTATCATTAATTTTACTTAAAGAATAAAAACAAAAATTTCCTACCAAAACATAATAAATTAAACGAAATGTATAACAAA

CAGTTAATCCTGTTGAAAAAAAGAAAATAACATAAATATAAATATTTAAGTATCTTATAGATAAAACCTCTAAAATTAAATCCTTTGAATAAAACCCCGA

TAAAAAGGGTAAACCACATAATGCTAAATTTGAAATTATAAAATAAGTACAAGTTAAAGGTATAACTTTAATTAACCCCCCTATATACCGAATATCTTGA

CAATTTCTTAATCTGTGAATTATACACCCTGCGCATATAAATAATAAAGCCTTAAATAAAGCGTGAGTTAATAGGTGAAAAAAAGCTAATTGATACTCCC

CTAAAGCTAAAATACTAATTATCAAACCCAATTGTCTTAAAGTAGACAGAGCAATAATTTTTTTTAAATCAAACTCAAAGTTTGCGCCTATTCCTGCTAT

AAATATAGTTATTGTTCCAATAAATAATAAAATTAATATTAAATTACTTGTTAATGCAAAATTAAAACGAATTAATAAGTAAACACCTGCTGTTACTAAG

GTAGAAGAATGAACTAAAGAAGAAACAGGTGTTGGAGCTGCTATTGCTGCAGGCAACCAAGAAGAAAATGGAATTTGAGCTCTTTTAGTTATAGCTGCTA

ATATAATTAATAAAATAATAATATATATTTCTATACTATTTTTATATACATCAATATAAAAAATATAGTTAAATCCCCCAAAATTTATTATTCATGCAAT

TGCTATTAATAAAGCAACATCTCCAATACGGTTAGTTAAAGCTGTAATTATCCCTGCATTGTAAGATTTAATATTTTGATAATAAATAACTAAACAATAA

GAAACTAACCCTAACCCATCTCATCCTAATAAAATCCTAATTAAATTAGGAGAAATAATCAATAACATTATAGATAAAACAAATATAGAAACTAATATAA

TAAATCGATGCAAATAAATATCTCCTTCTATATACTCTTCACTATAGTAAATTACTATAGAAGAAATAAATAAAACAAAACTCATAAATAATAATGACAT

CCAGTCAAGTAAAATGGTTATAATAATTCTACAAGAATTAATCCTTAATATTTCATATTCTAATATTAGTCTATAATCTAAAATTATGAAATTTAAGCTT

AATAAAAATCTTAATACTCTAAAAAATAAAAACGTTACAAAATAAATTAAACAAATAGAAATAATTTAAAGTAAATTTTACATCTTTGATACCACAAATC

AATATTTTTTATTAAACTATTTAAATTACAATCATAAAACTAAAAATTCTCTCTTTAAAATTAAGATATTCAAGGGTAACCAATGAAGTAATAATAATAA

ATACTCACGAACAAACCCTCTTGAAAAAGAATATAAATTTCTAACTAACTTGCCATGTTGACTATAAGAATATAAATATAAAGAATACGCAGCTCTAAAA

AAAGATATTAAAGATAAAAAAACCATTGTTCAACTCCTTCATCTAACTAATCTATTAATTAAAATAATCTCACCCAGTAAGTTTAATGAAGGAGGAGCCG

CTATATTACAACAACTAAATAAAAATCATCATATTCTTATTATTGGTATTAAATTGATTAGCCCCTTATTTAAATAAATACTTCGGCTATTTAGCCGTTC

ATAAGAAATATTTGCTAAACAAAATAATCCTGAAGAACATAAACCATGGGCAATTATTATAACTAAAGCCCCTCTTATACCCCAATAACTTAAAGTTAAA

ATCCCTCTTAATACTAAACCTATATGCGCTACAGAAGAATAAGCAATTAAAGCTTTAATATCAACTTGACGTAAACATATTAATGAAACAAAAAATCCCC

CTACTATTCTAATTGTAATAAAAATATAGTTTACTTGTAAACCTACAGTTAAAAAAATATTTATTAAACGTATTAATCCATACCCCCCTAATTTTAATAT

AACTCCAGCTAAAATTATAGAGCCAGCAACTGGGGCTTCAACATGGGCTTTAGGGAGCCAAAGATGAACAAAAAATATAGGCATTTTAATAAAAAACACT

ATATTTATACATATAAATAACAAGAATCTTTTTACATCATAACATAAAAAGAAAAAATCTAAAGAATGAAATTTTTCATAATAATAAAAAATCCTAATTA

TTATAGGTAATGAAGCAAATAATGTGTAAAATAACAAGTAAACACCTGCTTGCAAACGCTCAGGTTGATACCCCCAACCAATAATTAATAAAAGAGTGGG

AATTAAGCTAAACTCAAAAAACAAATAAAAAATAAATAAATTTAAAGAACTAAATGTTATAACTAAAGATAATAATAAAATAATTATTACTAATAAAAAT

AAATTATAAAAATAGTTTTTTCTATAAATACTTTCAGAAGCTAATAATATTAAAGAACAAATCCATAAACTTAATAAAATTATTATAAAAGATAAAAGGT

CATACCCTATAAAATAAGAAATATTTATATACAAATAATTAAAACTAAATCTTAACCCAAATATAAATGTAATAAAAAAATATATATATTGATTAAATCA

GTATCTCTTTTTAATACAACTTAAAGGTACCAATATTAATATTATAAAAATAAACTTTATCATAATACATTAAAAGTTTGAAAATAATCATTTCCATGTG

TTCGTATTATAGAAACTAATAACGATAAACCTAAAGCTCCTTCACAAACTCTTATAGTTAAAAATACTATACCAAAATAAAATTCAAAATTAAAATATAT

TAAAAATAGATATAAATTAAAGTATAATCCTAAAATAATATACTCTAACCTTAATAATATTAAAAGTAAATGTTTACGTTTAATACAAAAAGAAACTAAT

CCTGTAAAATATATAATCACTGAAAATAACATACAAAAAATTAACATTAGTTTTAATAATTTAATAAAAATACTGGTCTTGTAAATCAGAAATAAGGATT

TTCTTTTAAAACTTCAGAGAAAGAGTAAACCTCTATCATTAATCTCCAAAATTAATATTTTAAATAAACTATTCTCTGTATAATCTTATTACTAATAACA

TTATCTTTTATTAGTTCAATAACTTTTATATTTTTAAGTCACCCTTTATCTATAGGGCTTATTTTATTAATACAAACTATTATTATAGCTTTAACTATAG

GTTTTTTTAATATTAATTTCTGATATTCTTATATTTTATTCCTTATTATAATTGGAGGAATATTAGTTTTATTTATTTATATAACAAGAGTAGCTTCAAA

TGAAAAATTTTCATTTTCTATTAAAATTACATTAATAATTAGAATTATAACTTTAGGATTTTTATTCAGTATTGCTATAATAGACCCCTACTTTTCAGAC

ATCAACTCAATTTACACAGAAAACTTAGATAATTATAAAGAATATAATATATCATTTAGAAAATATTTAAGTTATCCTAATATTATCATTATATACATAA

TAATTATTTATTTATTAATTACATTAATTGCAGTTGTAAAAATTACTCAAATTGAAAAAGGACCTTTACGTCAAACTAACTAATGAAAACACCATTACGA

AAAGCTTCCCCCCTATTAAAAATTATTAATAATAGAATTATTGATTTACCTACACCATCTAATATTTCTGCTTGATGAAATTTCGGATCCTTATTAGGAC

TCTGTCTTTTTATTCAAATTATCACTGGAATTTTCTTAGCTATACATTTTACAGCCCATATTGACATAGCATTTAATAGAGTAATTCATATTTGTCGAGA

TGTAAATTATGGCTGATTATTACGAACAATTCATGCTAACGGGGCTTCTTTTTTCTTTATTTGTATTTATCTACATATTGGCCGAGGAATATATTATAGA

AGCTATAATTTACATTTAACATGAACTATTGGAGTAATCATCCTTTTTATAGTAATAGCTACAGCATTCTTAGGGTATGTATTACCTTGAGGGCAAATAT

CTTTTTGAGGAGCTACTGTTATTACCAATCTTCTCTCTGCTATTCCTTATCTAGGAAATATGATTGTCCAATGATTATGGGGGGGATTTGCAGTAGATAA

CGCCACTTTAACTCGATTTTTTACATTACATTTTATTCTTCCTTTTATTATTTTAGCTTTAATAATTATTCATTTATTATTTCTTCATCAAACTGGTTCT

AATAACCCTTTAGGATTAAATAGAAATATCGATAAAGTACCTTTCCATCCATATTTTACTTACAAAGATACTTTTGGATTCATTATAATAACTATATTAT

TAATATTTCTAGTTTTAATTAATCCCTATCTTTTAGGAGACCCAGAAAACTTCACACCCGCTAACCCTTTAGTAACCCCAGTCCATATCCAACCAGAATG

ATACTTTTTATTCGCATACGCTATTTTACGATCAATTCCTAATAAATTAGGTGGAGTAATTGCTTTAGTTATATCAATTGCTATTTTATTAATTATACCT

TTAATTAACAAAAAAAAATTTAGTAGAACTCAATTTTACCCATTAAATAAAATTTTATTTTGATCTTTTGTCTCTATTGTAATCTTATTAACATGAATCG

GAGCTCGCCCAGTGGAAGATCCATACATTTTAACAGGTCAAATTTTAACAATTATATATTTTAGTTATTATTTCTTAAACCCCTTAATTCATAAAATATG

AGATTATATTATTTTTAAAACTTAGTTAATGAACTTGTTAAAGTGTATATTTTGAAAATATAAAAAAGAGTTTATTCTCTATTAACTTTACTAAATTTTA

TTCACTAAATAAAATAAGAAAAGATAACCAACTTTAAACCTAAAAAAAAAAATAAAAAATTTAAAGAAACTGGTAAATAACTTTTTCAACATATATATAT

TAACTTATCATAACGGTACCGAGGTAAAGTACCTCGAACTCAAACCCAAATAAATGCTATAAAAGTTAACTTCAAAAAAAAAAATAAAGAAAAAATATCT

CCCCCTATAAATAACAACACACATAATATACTTATAAATAAAATTCTAGCATACTCAGCCAAAAAGATTAAAGCAAATCCCCCTCTTCTGTACTCTACAT

TAAACCCAGAAACTAACTCTGATTCTCCTTCTGCAAAATCAAAAGGAGTCCGGTTAGTCTCAGCTAAACTTGAAGATAATCATATTATTCTTAAAGGTAA

GCACAGAAAAAAAAATCATACTATTTCTTGATATTTTATAAAATCTATTATATTTAAATTTAAAATTAACAATAAAAAAGATAATAAAATTAATGATAAA

CTTACTTCGTATGAAATTGTTTGAGCTACTGATCGAATACCCCCTAATAAAGCATAATTAGAATTTGACGATCAACCAGAAATTATGATAGTATAGACCC

TTAACCTAGAACAACATAAAAAATATAAAATACCTAAATTAAACCTAAATATAAATGTCAAAAAAGGTATACATATCCATAATAAAAGAGCTAAAAATAA

ATTAAAAACTGGAGATATATAATAAATAAAAAAATTTGATATTAAAGGATAAGTTTGTTCTTTAGAAAATAACTTAATAGCATCACTAAAAGGTTGAGGA

ATACCTATAAATCCAACTTTATTGGGCCCTTTACGAATTTGGATATATCCTAAAACTTTACGCTCTATTAATGTTAAAAAAGCTACACCAATTAAAACAC

AAATAATTAAAACTAAACTTGTAAATAATAAAAGAAATAAATCTTGTAATATAATGTATTACTTGTGTTAAACACATATTTAAATTCTAAATTTAAAGCA

CTAATCTGCCAAAGTAATATTCATATTCAAATTATATTAAATTTTAAAGGTATCTGATCCTTTCGTACTAAAATACCTATGTTTTTTAAAGATAGAAACC

AACCTGGCTCACGCCGGTTTAAACTCAGATCATGTAAAATTTTAAAGGTCGAACAGACCTAACCTTTTAGCCCCTACACCAAAAGTTAATTTTAATCCAA

CATCGAGGTCGCAAACTTTTTTTTCGATAAGAACTCTAAAAAAAAATTACGCTGTTATCCCTAAGGTAATTTAATCTTGTAATCATTAAAAATGGATCAT

TCAATCATAAATTAATGTTTTTAAATAAAAAAAGTTTAATCAATTTTTCTGCTGCCCCAGCAAAATAGTTTAAATTATTAAATATATAAATATACTAAAA

TTAAATAATAATTTAAACTATAAAACTCTATAGGGTCTTCTCGTCTTTTAAAATTATATAAGCTTTTTTACTTATAAATAAAATTCTATATTCAATTAAA

TTGAGACAGTTACTTTCTCGTCCAACCGTTCATTCCAGCTTTCAATTAAAAAACTAATGATTATGCTACCTTTGCACGGTCAAATTACCGCGGCCATTCA

AATCCTCATTGGGCAGGTCAGACTTTAAATTATAATCAAAAAGACATGTTTTTAATAAACAGGCGAAAAGTGTATTTGCCGAGTTCCTTAATTTAACCTT

GAAGTTTTAATTTAATTACTAAATTAAAATATATACTAATTTTATCATTATTCTATATAAACCAATATTACATATATTATCTTAATAAACTACTTAAAAA

TAATATAAATCTTATTCTAACAAAAATTATTTATAACAAACTAAAGATTAACACTTCCAATTCTACTAATTTTTATTCAAAATATACATTTTTAACATTT

TATTTTAAAGCTTATCCCCTAAAATATTACTTTTTATATATAAAATACTAAATAATTAATATAATACAATAAAAAAACTAAATTAAATTTATTTCTTAAG

AAACTAGATATCTTAAAAAACGTATAACGTTTCATTTCTAATATAATATTTTAAAAATTTATGCCACAATTAAATTTATATTATATTAGCTCTTTATAAT

TCGAGAACACTAAATAATTAAATTATTTTAATAAACCCTGATACACAAGGTACAAAAAATTAATTTTTCTTTTTAAAAAATAAATCTCTATATATTTATA

TTATCTATCTCTATACAAATTAACTATAATAAAATTTTTATATTCTAAAATATACTAATATCAAAAATATTTTTTTTATATATATATATATA----TTAT

AAATTTTTCCTTTCAAATTAAATTGATTTTCACAACTAACTTTTTAATGTAAATAAAATGCTTTATTACAAGCTCTAATTTGCCATTCCAGGTACACTTT

CCAGTACACCTACTATGTTACGACTTATCCCTCTTTAGAGAGGGAGCGACGGGCGATATGTACATATTCTAGAGCTATACTCATATAATTAAACTAAACT

ATATTACTTTCAAATCCACTTTATAAAATAATGTTAATTATTTTAACCATCTAAATAATTTTATTGTAACCCATCTCTCCTTATCTATACGCTGTATCTT

GATCTGATTTTTTTTATACTTATAAATTTTGAACATTCCAAATTCTTTAAAAACATTCAACCTACGACGATATACAAACCTTTAAAATAAGTACGATTAA

TCGTGGACCATCAATTATAGGACAGGTTCCTCTGAGTAGACTAAAATACCGCCAAATTCTTAAAATTTCAAGAACATAACTACTACTATTCAAGCATCTA

AAATTTGCATTTTTAATAATAGGGTATCTAATCCTAGTTTTTTATAAAAATCTCATAAACTCATTTTTCACATTTAAAAAATTAATTATACTTACTAATT

TCACCTAATAAATACAATATAAATTAATAATAAAATAACTTATTATATACTGAACAAATTTAATTGTATTGTTTGTGTAACCGCAACTGCTGGCACAAAC

TTGGTCAATACTATTATAAATTCCTAAATCAAAATTTCTTTTAAATTTAATCTTCACTATTGCAATTCTTTAATTAAATATATAGAATAATTTATCTTTT

TAAAATAAATTCATTAAACACTAAAATTTACATATAAAATAATTTAAAAATTAAAATCTCAAGCTAGAATAAAACTTTATTTTTTTTTCAATATACATAA

ATTATATAAAATAAAAGTACCCCCC-TACCATTTTAATTAAATGAATTAATTTTTAATGCCTTAAATTTATTTCCAATTTATTTTTATTATATAGTTTTT

TATTTAAAAATAAAACTTTATACCTAAAATTATATACATAAATTATATTTAAAATTTATAATTTAAAGAACTATAAAAATTAAATTAAAATTTAAAATCT

ATCTAATTTATAGATAAACAAATTAAATTAAACTAACTTACCTGATAAATTTAACTATATACGCGCAGGTATATTAATTATTTAATTATAATATAATACA

TTTGTTTTATTAAATAATTATTAAGCAAAATAGCATTAATTTATTATATAAAAGATCTAGGTACTATTAATTTAAAGTAAATTTTTTTTTTTTTTGTAGT

ATAATTTATACTAATATACGGCATTTAATATCTTCTATATAAATGTTTATATTATTTATATTTATCTATTTATAATTCTTAATAATAATTTAGATAATTA

TTAATTTATATATTAAATATTTATATATATATATATAATAATAAGGATATATATATATATATGTATATAGATAAATTAAATTTAGTTATATATAAAAAAA

TTAATATTAATTTTACTTTTAATTTTTTCTTTAATTTAAACTATTGGATATTCAGATTCTAATATATTATAATATATCAACCATTAATGAATAACTTTAT

ATATATAATAAAAATGTTAATTAATATATCAATTCATATGAATATATAATATTAATAAATAAATTCTTAATAAATAATAATGATAATATATATATATTGT

ATATTAATGTACTATATATATAAATTCATTAAACATTTATATTTGATACCCCCCCAAAATTTTTTCTAAAAATGTGAAGTTTCAATTTAATTAAAATTTT

CTACAATGGTCAAAAACGGCCAAAAAATGGAAAATAAAGTGTGCACAGAAAATGCACATAAAATGCACATTTTTGAAAATTTCCGATTTTTGAAAAACCG

TTTGAACCAAACCTCTGATGTACCTTTTTTCGCCCCCCCTGATTACTAAAATTTATCCTCAATTACTAAGATTTATCCGCATTTTTGCGACAGGAATTTT

GAGTATAAAATCAATTCCACACTAAATTTTTTTTT-TTTTTTTTGTGTCATTTCTCCAACATGGGCAATTTTTTTTTTAGTCGAAGGACAAATTTTAGTA

AAAAAAGTTTGAAATTAGGTTAAACCCCTGTTTAACCAAAAACTATTACTTTTCAATATTATACCCAATTTTATTTAAAATAAATTTTATTATACTTTTA

TTTATGCAAAAAGATCTTCTTATAAACTTTT

>DMR74j_JP_Nanae_Hokkaido

AATGAAGTGTCTGACTATAGAGTTACTTTGATAGGGTAAAAAAAGTGAATTTTCACCTTCATTATAATTAACAGAATTAAACTATTTCTTTAAGCTTCAA

AAACTTATGTACATTATATACTAAATTATAAAAAGATAAGCTAATTAAGCTACTGGGCTCATACCCCATCAATAAAGGTTACAATCCTTTTCTTTTTAAT

GTATTATAAGCTTTTATTTTTTAATTCTCTTATAATTGGAACCTTAATTGCTATTTCTTCCTACTCTTGAATAGGAATATGAATAGGATTAGAAATTAAT

CTTCTCTCTATGATCCCCCTCATTAGAGACAACAAAAATATAATAGCCTCAGAAGCTGCTTTAAAATATTTCATTATTCAAACAATAGCATCAACATTGT

TATTATTCTCAATTATTATAATATCAATGAAATTTATATATCAAATAAATTTAATCACTTACTTTAATTTAATTTTTAACACTTCATTGTTCATCAAAAT

AGGAGCAGCCCCATTCCATTTTTGATTCCCCGAAATAATAGAAGGATTAAATTGATTAAATGCCATTATCATACTTACTTGACAAAAACTAAGGCCCATA

GTATTACTAACGTATTCTAATACAACCTCCATATATCTAATTTTAACAATTATATTTAGAATAATAATCAGAGGAATTATAGGCCTAAATCAAACTAGAT

TACGGAAAATTATAGCTTATTCATCTATCAACCATATTGGATGAATAATCAGTTCAATTATATTAATTGAAATTGTTTGATTTTACTATTTTATTATTTA

TTGCATTATTACTATTAATATCAGAATTATATTTATAAAATTAAATGTTTTTCATATCAATCAATTATATATTTCAATAAATTACCATATTTTACTTAAA

TTATTCTTTGCCTTAAATTTCATGTCTTTAGGAGGATTACCCCCATTTTTAGGGTTTTTCCCTAAATGACTTACAATTCAAACTTTAATCCAAAGAAATA

TGTACTCAATTGCTTTCATTATAATTTTAATAACTTTAATAACACTTTTTTTTTACCTCCGAATTACTTTTTCAATTTTACTACTAAGAAAAACAGTTTT

AACATTTTATACACAACCAAAAATTGATACTAATTACATTATAGCATTTAATTTTATTACATTATTAAGATTAATTTTCGTTACTTTGATATTCAATTTC

TTATAAATTAAACCTGAAGGATTTAAGTTAAATTAAACTAAGAACCTTCAAAGTTCTAAATAAAGTAAATTCTTTAAGCCTTAGGGCTTAGCCCATCTTT

AAATTTGCAATTTAAAATTCTTTTTGAACTATAAAGCTTGATAAAAGAAACTAATTTCGTATGTAAATTTACAGTTTACCGCCTAAACCTCGGCCATTTT

ATCGAATAAATGGCTATTCTCTACAAATCACAAAGATATCGGAACTTTATACTTTCTATTTGGAAGTTGAGCAGGAATAGTAGGCACTTCCTTAAGTTTA

CTGATTCGTGCTGAATTGGGAAACCCAGGATCTCTAATTGGAGATGATCAAATTTATAACGTAATCGTAACAGCTCATGCTTTCATTATAATTTTTTTTA

TAGTAATACCTATTATAATTGGAGGATTCGGAAACTGATTAGTTCCTTTAATGCTTGGAGCCCCCGACATAGCATTCCCCCGAATAAATAACATAAGATT

TTGACTTTTACCTCCTTCATTAACTCTACTTCTAATAAGAAGATTAGTCGAAAGAGGGGCAGGTACAGGCTGAACAGTTTATCCCCCACTATCAGCCAAT

ATCGCCCATAGAGGAGCATCAGTTGATTTAGCAATTTTTAGACTTCATCTTGCAGGAATTAGCTCAATTTTAGGTGCAGTAAATTTTATTACTACTGTAA

TTAACATACGATCAACAGGAATAACTTTTGATCGAATACCCCTATTTGTTTGATCTGTAGTTTTAACAGCACTTCTTCTGCTATTATCCCTCCCAGTTTT

AGCAGGAGCAATTACTATACTATTAACAGATCGAAATATTAATACAACATTTTTTGATCCCGCAGGTGGAGGAGACCCCATTCTCTACCAACATTTATTT

TGATTTTTTGGTCATCCAGAAGTTTACATTTTAATTTTACCTGGATTTGGTATAATCTCCCATATTATTAGCCAAGAAAGAAGAAAAAAAGAAACATTTG

GAACTTTAGGTATAATTTACGCTATAATAGCTATTGGTTTACTAGGATTTATTGTTTGGGCTCATCATATATTTACAGTAGGTATAGATGTAGACACACG

GGCATATTTTACATCAGCAACAATAATCATTGCTGTTCCTACAGGAATTAAAATTTTTAGATGATTAGCTACTCTCCATGGATCACAATTAAACTACTCC

CCGTCTCTTTTATGGGCATTAGGATTTGTATTCCTATTTACAGTAGGAGGATTAACAGGGGTAATTCTAGCTAATTCATCAATTGACATTATTTTACATG

ATACTTACTATGTAGTTGCACATTTCCATTATGTCCTTTCCATAGGAGCTGTATTTACTATTATAGCAGGTTTTGTTCATTGATTCCCTTTATTTACAGG

TTTAACAATAAATTCAAAATTTCTTAAAATTCAATTTTTAACAATATTTATTGGTGTTAATATAACATTCTTCCCTCAACATTTCTTAGGATTAAGAGGA

ATACCTCGACGTTATTCAGATTACCCAGATGCTTACACAACTTGAAATATTATCTCATCTATTGGATCTTTAGTTTCTTTAATTAGTATTTTTATCTTTT

TATTTACTATTTGAGAAAGGCTAATTTCATTACGAAAAAGAATTAGGTCTTTAAGAATATCTACATCAATTGAATGACTCCAACAAATACCCCCTTCAGA

ACATAGTTATTCGGAACTTCCAATGCTTACTAACTTCTAATATGGCAGATTAGTGCAATGGATTTAAACCCCAAATATAAAGATTAAACTTTTTTTAGAA

ATAGCTACTTGAAATACCATTTTACTTCAGGATAGGGCATCCCCATTAATAGAGCAACTCTCATTCTTTCATAACCATGCTCTTCTAATTCTCTTTATAA

TTACCGTTCTAGTAGGTTATTTAATAGGAACTTTATTTTTTAACCAATTTAATTACCGATTTTTATTAGATGGTCAAACTATTGAAATTATTTGAACTAT

TTTACCTGCTGTAACACTAATTTTTATCGCATTACCGTCTTTACGCTTACTTTATCTTCTAGATGAAGTTAATAACCCTTTAGTAACTATCAAAACAATT

GGGCATCAATGATATTGATCATACGAATATAGAGATTTTATAAATTTTGAATTCGATTCCTATATAATTCCTTTGACAGAAATAAAACCTCAAAATTTTC

GTTTATTAGATGTTGATAACCGAGTAATTGTCCCCTTTAACTCCCAAATCCGAATGATAGTAACAGCTGCCGATGTTATTCATTCATGAACTATCCCGGC

TTTTAGTGTAAAAATTGATGCAACACCGGGCCGACTAAATCAAACTAGATTCCTAATTAATCGAACAGGATTATTTTATGGTCAATGCTCAGAAATTTGT

GGAGCAAATCATAGATTTATACCTATTACTGTAGAAAGAATTTCACCTTCATTTTTTACTAAATGAATCTCAAAAATAAATAACCTATCATTAGATGACT

GAAAGTAAGTAATGGTCTCTTAAACCAATTAATAGTAGTTTAACATCTACTTCTGATGGCCAAAAATTTAGTTAAGATATAACATTAGTTTGTCATACTA

AAATAATCATAATTTGATAATTTTTAATTCCACAAATAGCACCTTTAAACTGACTATCTTTATTTTTTTTAATTATTATTATTTTTTTACTTTTTAATGT

ATTAAATTACTTTAGATTCTTACAGCCCTTAAAAACCCAATCTCATAACCCTACAATTAAAAAAATTAATTGAAAATGATAACTAATTTATTTTCATCTT

TTGATCCTAGAACTTCTTTTAATTTAAGATTAAACTGATTAAGAATACTATTAGGGCTAATATTTATCCCCCCAATATTTTGATTAGTTCCTTCACGCCA

TAATTTTCTATGAATTAAAATTATTTTAACATTACACCAAGAATTTAAGGTTTTAATTGGTAATAATAATATTAAAGGAAGAACCTTAATATTTATTTCA

TTATTTTCTATAATTGTTTTCAATAACTTTTTAGGATTATTTCCGTATATTTTTACAGGAACAAGACATTTAATTATAACATTATCTCTTGCCTTACCTT

TATGAATTAGATTCATATTATACGGGTGAATTAATAACACTATCCACATACTTGCTCATTTAGTTCCCCAAGGAACACCCCCAGCTCTTATGGCATTCAT

AGTAGTAATTGAATCAATTAGAAATATTATTCGTCCTGGTACTTTAGCTGTTCGATTAGCTGCTAATATAATTGCTGGACATTTACTAATAACTTTACTA

GGAAACACAGGATTAAATTTATCAATTTTTATACTAAGTATTCTTATTATCACACAAATTCTTTTATTAATTTTAGAATCTGCTGTTGCGATCATTCAAT

CTTATGTATTTGCTGTATTAAGAACTTTATACTCTAGAGAAATTAATTAATGTCAAGACATAAAAATCACCCTTATCATTTAGTTGATGCAAGACCTTGA

CCTATTTTAGGTGCTTTTAGAGCTATAATTACAATAATTGGAATTATCAAATGATTCCATTTTTATAATAATTCTTTATTTTACTTAGGGACATTAATCA

CAATTTTAATTATAATTCAATGATGACGAGATATCACTCGTGAGGGAACTTTCCAAGGACTTCATACTTACGCTGTAACTATAGGTTTACGTTGAGGAAT

AATTTTATTTATTACATCAGAAGTATTTTTCTTTATTTCTTTTTTTTGAGCCTTTTTTCATAGTAGATTAACACCCGCTATTGAACTAGGGATACTCTGA

CCACCCAAAGGAATTACCCCATTTAACCCAATTCAAATTCCATTATTAAACACTTTAATTCTTTTAACTTCAGGATTAACTGTAACTTGAGCTCACCATA

GATTAATAGAAAATGACTATAACCAAACAATGCAAGGTCTTGGTTTAACAGTTTTACTAGGAGTATATTTTACTTTATTACAAGGTTACGAATATTTAGA

AGCCCCCTTTACTATAGCAGATTCTGTTTATGGATCAACATTTTTTATTGCTACTGGTTTCCACGGATTACATGTTATTATTGGCACAACCTTTTTAGCT

GTTTGTTTAATACGACATTTTAATAACCATTTTACTTGTATCCATCACTTTGGATTTGAAGCTGCTGCTTGATACTGACATTTTGTTGATGTAGTATGAC

TATTTCTTTATATTTCTATTTACTGATGAGGTAGATATTTATATAGTATAATAATTATAATTGATTTCCAATCAAAAGATCTAAAAAAA-TTAGTATAAA

TAATCATTATAATTTGAAATATAGGGTTAATTATTTTTTCTATCTCTTTTATCTTAATTATACTATCTTTTACAATCTCTAAAAAAAGATTTATAGACCG

AGAAAAAGCTTCTCCATTCGAGTGCGGATTTGACCCCAAAAGATCAGCCCGTTTACCCTTTTCTTTGCATTTTTTTTTAATTGCAGTAATTTTCTTAATT

TTTGATGTTGAAATTACTCTTCTTATCCCTTTAATTCTAACAATAAAAATTACTAATATTACTATATATACCTATATTGCTCTTTTCTTTTTAATGATTC

TTTTAATAGGACTTTACCATGAATGAAACCAAGGAGCCTTAAATTGAGCTCTTTAGGGTAATAGTTAAGTATAACATTTAAGTTGCATTTAAAAAGTATT

GATTTTTCAATTTACCTTAAATAAGAAACAATTAATTGTATTTAGTTTCGACCTAAAATTTAGGTGTATGAACACCCTTATTTAAATTAATTGAAACCAA

AAAGAGGTATATCACTGTTAATGATACTAATGAGAAAAACTCCAATTAAGGAAATAAGATATTCAAGAGTAAGCTTCTAACTTAACTCTTTAGCAGTGAA

AGTCTGTTAATATTTCTATTTATATAGTTTAATAAAACATTATTTTTTCATAATAAAATTAGAATAAATTTATTCTTATAAATATTTAAAAGTAAATTTT

ACTTCCCTGATAACTTCACTATCATACTCTATATAAGCTATTTAAATTAAATATATAAAATTATAAAAATTACCCATATTATAATTAAAAGTATAAAAAT

CTTATAATTGTTATTAAATATAAACTGTAAAAATACAGAAGTATTACTAATTTTACTATATAAATTTTGTCTTCCATAATACTCTGATCAGCCTTGATCA

ATAGTTTTATATAACTTACTACCTAATTTAATTGGATAATAATTTAAACCAAATGTTGAAATATAGGGTATATTCCACATAGAAGAAAAAAAAAGTCTTG

AATTTAACAAATAAAAAGATTTTAATCTATCATTTAAAGTAAACTTAGAAAGCTCAAACCCAAACCAAGCCCCAAAAAATGATACAATTAAAGCTATAAT

TTTTATTGTAAAAGGTAAACAAATAAAATAAGGTGTTGGGAATATCAACCATATTAATATTCTTCCCCCAACAATAACTAAAAAAATTAAACCTGATATC

CCTTGAAGTATAATTTTTCTATTATCATTAATTTTACTTAAAGAATAAAAACAAAAATTTCCTACCAAAACATAATAAATTAAACGAAATGTATAACAAA

CAGTTAATCCTGTTGAAAAAAAGAAAATAACATAAATATAAATATTTAAGTATCTTATAGATAAAACCTCTAAAATTAAATCCTTTGAATAAAACCCCGA

TAAAAAGGGTAAACCACATAATGCTAAATTTGAAATTATAAAATAAGTACAAGTTAAAGGTATAACTTTAATTAACCCCCCTATATACCGAATATCTTGA

CAATTTCTTAATCTGTGAATTATACACCCTGCGCATATAAATAATAAAGCCTTAAATAAAGCGTGAGTTAATAGGTGAAAAAAAGCTAATTGATACTCCC

CTAAAGCTAAAATACTAATTATCAAACCCAATTGTCTTAAAGTAGACAGAGCAATAATTTTTTTTAAATCAAACTCAAAGTTTGCGCCTATTCCTGCTAT

AAATATAGTTATTGTTCCAATAAATAATAAAATTAATATTAAATTACTTGTTAATGCAAAATTAAAACGAATTAATAAGTAAACACCTGCTGTTACTAAG

GTAGAAGAATGAACTAAAGAAGAAACAGGTGTTGGAGCTGCTATTGCTGCAGGCAACCAAGAAGAAAATGGAATTTGAGCTCTTTTAGTTATAGCTGCTA

ATATAATTAATAAAATAATAATATATATTTCTATACTATTTTTATATACATCAATATAAAAAATATAGTTAAATCCCCCAAAATTTATTATTCATGCAAT

TGCTATTAATAAAGCAACATCTCCAATACGGTTAGTTAAAGCTGTAATTATCCCTGCATTGTAAGATTTAATATTTTGATAATAAATAACTAAACAATAA

GAAACTAACCCTAACCCATCTCATCCTAATAAAATCCTAATTAAATTAGGAGAAATAATCAATAACATTATAGATAAAACAAATATAGAAACTAATATAA

TAAATCGATGCAAATAAATATCTCCTTCTATATACTCTTCACTATAGTAAATTACTATAGAAGAAATAAATAAAACAAAACTCATAAATAATAATGACAT

CCAGTCAAGTAAAATGGTTATAATAATTCTACAAGAATTAATCCTTAATATTTCATATTCTAATATTAGTCTATAATCTAAAATTATGAAATTTAAGCTT

AATAAAAATCTTAATACTCTAAAAAATAAAAACGTTACAAAATAAATTAAACAAATAGAAATAATTTAAAGTAAATTTTACATCTTTGATACCACAAATC

AATATTTTTTATTAAACTATTTAAATTACAATCATAAAACTAAAAATTCTCTCTTTAAAATTAAGATATTCAAGGGTAACCAATGAAGTAATAATAATAA

ATACTCACGAACAAACCCTCTTGAAAAAGAATATAAATTTCTAACTAACTTGCCATGTTGACTATAAGAATATAAATATAAAGAATACGCAGCTCTAAAA

AAAGATATTAAAGATAAAAAAACCATTGTTCAACTCCTTCATCTAACTAATCTATTAATTAAAATAATCTCACCCAGTAAGTTTAATGAAGGAGGAGCCG

CTATATTACAACAACTAAATAAAAATCATCATATTCTTATTATTGGTATTAAATTGATTAGCCCCTTATTTAAATAAATACTTCGGCTATTTAGCCGTTC

ATAAGAAATATTTGCTAAACAAAATAATCCTGAAGAACATAAACCATGGGCAATTATTATAACTAAAGCCCCTCTTATACCCCAATAACTTAAAGTTAAA

ATCCCTCTTAATACTAAACCTATATGCGCTACAGAAGAATAAGCAATTAAAGCTTTAATATCAACTTGACGTAAACATATTAATGAAACAAAAAATCCCC

CTACTATTCTAATTGTAATAAAAATATAGTTTACTTGTAAACCTACAGTTAAAAAAATATTTATTAAACGTATTAATCCATACCCCCCTAATTTTAATAT

AACTCCAGCTAAAATTATAGAGCCAGCAACTGGGGCTTCAACATGGGCTTTAGGGAGCCAAAGATGAACAAAAAATATAGGCATTTTAATAAAAAACACT

ATATTTATACATATAAATAACAAGAATCTTTTTACATCATAACATAAAAAGAAAAAATCTAAAGAATGAAATTTTTCATAATAATAAAAAATCCTAATTA

TTATAGGTAATGAAGCAAATAATGTGTAAAATAACAAGTAAACACCTGCTTGCAAACGCTCAGGTTGATACCCCCAACCAATAATTAATAAAAGAGTGGG

AATTAAGCTAAACTCAAAAAACAAATAAAAAATAAATAAATTTAAAGAACTAAATGTTATAACTAAAGATAATAATAAAATAATTATTACTAATAAAAAT

AAATTATAAAAATAGTTTTTTCTATAAATACTTTCAGAAGCTAATAATATTAAAGAACAAATCCATAAACTTAATAAAATTATTATAAAAGATAAAAGGT

CATACCCTATAAAATAAGAAATATTTATATACAAATAATTAAAACTAAATCTTAACCCAAATATAAATGTAATAAAAAAATATATATATTGATTAAATCA

GTATCTCTTTTTAATACAACTTAAAGGTACCAATATTAATATTATAAAAATAAACTTTATCATAATACATTAAAAGTTTGAAAATAATCATTTCCATGTG

TTCGTATTATAGAAACTAATAACGATAAACCTAAAGCTCCTTCACAAACTCTTATAGTTAAAAATACTATACCAAAATAAAATTCAAAATTAAAATATAT

TAAAAATAGATATAAATTAAAGTATAATCCTAAAATAATATACTCTAACCTTAATAATATTAAAAGTAAATGTTTACGTTTAATACAAAAAGAAACTAAT

CCTGTAAAATATATAATCACTGAAAATAACATACAAAAAATTAACATTAGTTTTAATAATTTAATAAAAATACTGGTCTTGTAAATCAGAAATAAGGATT

TTCTTTTAAAACTTCAGAGAAAGAGTAAACCTCTATCATTAATCTCCAAAATTAATATTTTAAATAAACTATTCTCTGTATAATCTTATTACTAATAACA

TTATCTTTTATTAGTTCAATAACTTTTATATTTTTAAGTCACCCTTTATCTATAGGGCTTATTTTATTAATACAAACTATTATTATAGCTTTAACTATAG

GTTTTTTTAATATTAATTTCTGATATTCTTATATTTTATTCCTTATTATAATTGGAGGAATATTAGTTTTATTTATTTATATAACAAGAGTAGCTTCAAA

TGAAAAATTTTCATTTTCTATTAAAATTACATTAATAATTAGAATTATAACTTTAGGATTTTTATTCAGTATTGCTATAATAGACCCCTACTTTTCAGAC

ATCAACTCAATTTACACAGAAAACTTAGATAATTATAAAGAATATAATATATCATTTAGAAAATATTTAAGTTATCCTAATATTATCATTATATACATAA

TAATTATTTATTTATTAATTACATTAATTGCAGTTGTAAAAATTACTCAAATTGAAAAAGGACCTTTACGTCAAACTAACTAATGAAAACACCATTACGA

AAAGCTTCCCCCCTATTAAAAATTATTAATAATAGAATTATTGATTTACCTACACCATCTAATATTTCTGCTTGATGAAATTTCGGATCCTTATTAGGAC

TCTGTCTTTTTATTCAAATTATCACTGGAATTTTCTTAGCTATACATTTTACAGCCCATATTGACATAGCATTTAATAGAGTAATTCATATTTGTCGAGA

TGTAAATTATGGCTGATTATTACGAACAATTCATGCTAACGGGGCTTCTTTTTTCTTTATTTGTATTTATCTACATATTGGCCGAGGAATATATTATAGA

AGCTATAATTTACATTTAACATGAACTATTGGAGTAATCATCCTTTTTATAGTAATAGCTACAGCATTCTTAGGGTATGTATTACCTTGAGGGCAAATAT

CTTTTTGAGGAGCTACTGTTATTACCAATCTTCTCTCTGCTATTCCTTATCTAGGAAATATGATTGTCCAATGATTATGGGGGGGATTTGCAGTAGATAA

CGCCACTTTAACTCGATTTTTTACATTACATTTTATTCTTCCTTTTATTATTTTAGCTTTAATAATTATTCATTTATTATTTCTTCATCAAACTGGTTCT

AATAACCCTTTAGGATTAAATAGAAATATCGATAAAGTACCTTTCCATCCATATTTTACTTACAAAGATACTTTTGGATTCATTATAATAACTATATTAT

TAATATTTCTAGTTTTAATTAATCCCTATCTTTTAGGAGACCCAGAAAACTTCACACCCGCTAACCCTTTAGTAACCCCAGTCCATATCCAACCAGAATG

ATACTTTTTATTCGCATACGCTATTTTACGATCAATTCCTAATAAATTAGGTGGAGTAATTGCTTTAGTTATATCAATTGCTATTTTATTAATTATACCT

TTAATTAACAAAAAAAAATTTAGTAGAACTCAATTTTACCCATTAAATAAAATTTTATTTTGATCTTTTGTCTCTATTGTAATCTTATTAACATGAATCG

GAGCTCGCCCAGTGGAAGATCCATACATTTTAACAGGTCAAATTTTAACAATTATATATTTTAGTTATTATTTCTTAAACCCCTTAATTCATAAAATATG

AGATTATATTATTTTTAAAACTTAGTTAATGAACTTGTTAAAGTGTATATTTTGAAAATATAAAAAAGAGTTTATTCTCTATTAACTTTACTAAATTTTA

TTCACTAAATAAAATAAGAAAAGATAACCAACTTTAAACCTAAAAAAAAAAATAAAAAATTTAAAGAAACTGGTAAATAACTTTTTCAACATATATATAT

TAACTTATCATAACGGTACCGAGGTAAAGTACCTCGAACTCAAACCCAAATAAATGCTATAAAAGTTAACTTCAAAAAAAAAAATAAAGAAAAAATATCT

CCCCCTATAAATAACAACACACATAATATACTTATAAATAAAATTCTAGCATACTCAGCCAAAAAGATTAAAGCAAATCCCCCTCTTCTGTACTCTACAT

TAAACCCAGAAACTAACTCTGATTCTCCTTCTGCAAAATCAAAAGGAGTCCGGTTAGTCTCAGCTAAACTTGAAGATAATCATATTATTCTTAAAGGTAA

GCACAGAAAAAAAAATCATACTATTTCTTGATATTTTATAAAATCTATTATATTTAAATTTAAAATTAACAATAAAAAAGATAATAAAATTAATGATAAA

CTTACTTCGTATGAAATTGTTTGAGCTACTGATCGAATACCCCCTAATAAAGCATAATTAGAATTTGACGATCAACCAGAAATTATGATAGTATAGACCC

TTAACCTAGAACAACATAAAAAATATAAAATACCTAAATTAAACCTAAATATAAATGTCAAAAAAGGTATACATATCCATAATAAAAGAGCTAAAAATAA

ATTAAAAACTGGAGATATATAATAAATAAAAAAATTTGATATTAAAGGATAAGTTTGTTCTTTAGAAAATAACTTAATAGCATCACTAAAAGGTTGAGGA

ATACCTATAAATCCAACTTTATTGGGCCCTTTACGAATTTGGATATATCCTAAAACTTTACGCTCTATTAATGTTAAAAAAGCTACACCAATTAAAACAC

AAATAATTAAAACTAAACTTGTAAATAATAAAAGAAATAAATCTTGTAATATAATGTATTACTTGTGTTAAACACATATTTAAATTCTAAATTTAAAGCA

CTAATCTGCCAAAGTAATATTCATATTCAAATTATATTAAATTTTAAAGGTATCTGATCCTTTCGTACTAAAATACCTATGTTTTTTAAAGATAGAAACC

AACCTGGCTCACGCCGGTTTAAACTCAGATCATGTAAAATTTTAAAGGTCGAACAGACCTAACCTTTTAGCCCCTACACCAAAAGTTAATTTTAATCCAA

CATCGAGGTCGCAAACTTTTTTTTCGATAAGAACTCTAAAAAAAAATTACGCTGTTATCCCTAAGGTAATTTAATCTTGTAATCATTAAAAATGGATCAT

TCAATCATAAATTAATGTTTTTAAATAAAAAAAGTTTAATCAATTTTTCTGCTGCCCCAGCAAAATAGTTTAAATTATTAAATATATAAATATACTAAAA

TTAAATAATAATTTAAACTATAAAACTCTATAGGGTCTTCTCGTCTTTTAAAATTATATAAGCTTTTTTACTTATAAATAAAATTCTATATTCAATTAAA

TTGAGACAGTTACTTTCTCGTCCAACCGTTCATTCCAGCTTTCAATTAAAAAACTAATGATTATGCTACCTTTGCACGGTCAAATTACCGCGGCCATTCA

AATCCTCATTGGGCAGGTCAGACTTTAAATTATAATCAAAAAGACATGTTTTTAATAAACAGGCGAAAAGTGTATTTGCCGAGTTCCTTAATTTAACCTT

GAAGTTTTAATTTAATTACTAAATTAAAATATATACTAATTTTATCATTATTCTATATAAACCAATATTACATATATTATCTTAATAAACTACTTAAAAA

TAATATAAATCTTATTCTAACAAAAATTATTTATAACAAACTAAAGATTAACACTTCCAATTCTACTAATTTTTATTCAAAATATACATTTTTAACATTT

TATTTTAAAGCTTATCCCCTAAAATATTACTTTTTATATATAAAATACTAAATAATTAATATAATACAATAAAAAAACTAAATTAAATTTATTTCTTAAG

AAACTAGATATCTTAAAAAACGTATAACGTTTCATTTCTAATATAATATTTTAAAAATTTATGCCACAATTAAATTTATATTATATTAGCTCTTTATAAT

TCGAGAACACTAAATAATTAAATTATTTTAATAAACCCTGATACACAAGGTACAAAAAATTAATTTTTCTTTTTAAAAAATAAATCTCTATATATTTATA

TTATCTATCTCTATACAAATTAACTATAATAAAATTTTTATATTCTAAAATATACTAATATCAAAAATATTTTTTTTATATATATATATATA----TTAT

AAATTTTTCCTTTCAAATTAAATTGATTTTCACAACTAACTTTTTAATGTAAATAAAATGCTTTATTACAAGCTCTAATTTGCCATTCCAGGTACACTTT

CCAGTACACCTACTATGTTACGACTTATCCCTCTTTAGAGAGGGAGCGACGGGCGATATGTACATATTCTAGAGCTATACTCATATAATTAAACTAAACT

ATATTACTTTCAAATCCACTTTATAAAATAATGTTAATTATTTTAACCATCTAAATAATTTTATTGTAACCCATCTCTCCTTATCTATACGCTGTATCTT

GATCTGATTTTTTTTATACTTATAAATTTTGAACATTCCAAATTCTTTAAAAACATTCAACCTACGACGATATACAAACCTTTAAAATAAGTACGATTAA

TCGTGGACCATCAATTATAGGACAGGTTCCTCTGAGTAGACTAAAATACCGCCAAATTCTTAAAATTTCAAGAACATAACTACTACTATTCAAGCATCTA

AAATTTGCATTTTTAATAATAGGGTATCTAATCCTAGTTTTTTATAAAAATCTCATAAACTCATTTTTCACATTTAAAAAATTAATTATACTTACTAATT

TCACCTAATAAATACAATATAAATTAATAATAAAATAACTTATTATATACTGAACAAATTTAATTGTATTGTTTGTGTAACCGCAACTGCTGGCACAAAC

TTGGTCAATACTATTATAAATTCCTAAATCAAAATTTCTTTTAAATTTAATCTTCACTATTGCAATTCTTTAATTAAATATATAGAATAATTTATCTTTT

TAAAATAAATTCATTAAACACTAAAATTTACATATAAAATAATTTAAAAATTAAAATCTCAAGCTAGAATAAAACTTTATTTTTTTTTCAATATACATAA

ATTATATAAAATAAAAGTACCCCCC-TACCATTTTAATTAAATGAATTAATTTTTAATGCCTTAAATTTATTTCCAATTTATTTTTATTATATAGTTTTT

TATTTAAAAATAAAACTTTATACCTAAAATTATATACATAAATTATATTTAAAATTTATAATTTAAAGAACTATAAAAATTAAATTAAAATTTAAAATCT

ATCTAATTTATAGATAAACAAATTAAATTAAACTAACTTACCTGATAAATTTAACTATATACGCGCAGGTATATTAATTATTTAATTATAATATAATACA

TTTGTTTTATTAAATAATTATTAAGCAAAATAGCATTAATTTATTATATAAAAGATCTAGGTACTATTAATTTAAAGTAAATTTTTTTTTTTTTTGTAGT

ATAATTTATACTAATATACGGCATTTAATATCTTCTATATAAATGTTTATATTATTTATATTTATCTATTTATAATTCTTAATAATAATTTAGATAATTA

TTAATTTATATATTAAATATTTATATATATATATATAATAATAAGGATATATATATATATATGTATATAGATAAATTAAATTTAGTTATATATAAAAAAA

TTAATATTAATTTTACTTTTAATTTTTTCTTTAATTTAAACTATTGGATATTCAGATTCTAATATATTATAATATATCAACCATTAATGAATAACTTTAT

ATATATAATAAAAATGTTAATTAATATATCAATTCATATGAATATATAATATTAATAAATAAATTCTTAATAAATAATAATGATAATATATATATATTGT

ATATTAATGTACTATATATATAAATTCATTAAACATTTATATTTGATACCCCCCCAAAATTTTTTCTAAAAATGTGAAGTTTCAATTTAATTAAAATTTT

CTACAATGGTCAAAAACGGCCAAAAAATGGAAAATAAAATGTGCACAGAAAATGCACATAAAATGCACATTTTTGAAAATTTCCGATTTTTGAAAAACCG

TTTGAACCAAACCTCTGATGTACCTTTTTTCGCCCCCCCTGATTACTAAAATTTATCCTCAATTACTAAGGTTTATCCGCATTTTTGCGACAGGAATTTT

GAGTATAAAATCAATTCCACACTAAATTTTTTTTT-TTTTTTTTGTGTCATTTCTCCAACATGGGCAATTTTTTTTTTAGTCGAAGGACAAATTTTAGTA

AAAAAAGTTTGAAATTAGGTTAAACCCCTGTTTAACCAAAAACTATTACTTTTCAATATTATACCCAATTTTATTTAAAATAAATTTTATTATACTTTTA

TTTATGCAAAAAGATCTTCTTATAAACTTTT

>DMR75j_JP_Mori_Honshu

AATGAAGTGTCTGACTATAGAGTTACTTTGATAGAGTAAAAAAAGTGAATTTTCACCTTCATTATAATTAACAGAATTAAACTATTTCTTTAAGCTTCAA

AAACTTATGTACATTATATACTAAATTATAAAAAGATAAGCTAATTAAGCTACTGGGCTCATACCCCATCAATAAAGGTTACAATCCTTTTCTTTTTAAT

GTATTATAAGCTTTTATTTTTTAATTCTCTTATAATTGGAACCTTAATTGCTATTTCTTCCTACTCTTGAATAGGAATATGAATAGGATTAGAAATTAAT

CTTCTCTCTATGATCCCCCTCATTAGAGACAACAAAAATATAATAGCCTCAGAAGCTGCTTTAAAATATTTCATTATTCAAACAATAGCATCAACATTGT

TATTATTCTCAATTATTATAATATCAATGAAATTTATATATCAAATAAATTTAATCACTTACTTTAATTTAATTTTTAACACTTCATTGTTCATCAAAAT

AGGAGCAGCCCCATTCCATTTTTGATTCCCCGAAATAATAGAAGGATTAAATTGATTAAATGCCATTATCATACTTACTTGACAAAAACTAAGGCCCATA

GTATTACTAACGTATTCTAATACAACCTCCATATATCTAATTTTAACAATTATATTTAGAATAATAATCAGAGGAATTATAGGTCTAAATCAAACTAGAT

TACGGAAAATTATAGCTTATTCATCTATCAACCATATTGGATGAATAATCAGTTCAATTATATTAATTGAAATTGTTTGATTTTACTATTTTATTATTTA

TTGCATTATTACTATTAATATCAGAATTATATTTATAAAATTAAATGTTTTTCATATCAATCAATTATATATTTCAATAAATTACCATATTTTACTTAAA

TTATTCTTTGCCTTAAATTTCATGTCTTTAGGAGGATTACCCCCATTTTTAGGGTTTTTCCCTAAATGACTTACAATTCAAACTTTAATCCAAAGAAATA

TATACTCAATTGCTTTCATTATAATTTTAATAACTTTAATAACACTTTTTTTTTACCTCCGAATTACTTTTTCAATTTTACTACTAAGAAAAACAGTTTT

AACATTTTACACACAACCAAAAATTTATACTAATTACATTATAGCATTTAATTTTATTGCATTATTAAGATTAATTTTCGTTACTTTGATATTCAATTTC

TTATAAATTAAACCTGAAGGATTTAAGTTAAATTAAACTAAGAACCTTCAAAGTTCTAAATAAAGTAAATTCTTTAAGCCTTAGGGCTTAGCCCATCTTT

AAATTTGCAATTTAAAATTCTTTTTGAACTATAAAGCTTGATAAAAGAAACTAATTTCGTATGTAAATTTACAGTTTACCGCCTAAACCTCGGCCATTTT

ATCGAATAAATGGCTATTCTCTACAAATCACAAAGATATCGGAACTTTATACTTTCTATTTGGAAGTTGGGCAGGCATAGTAGGCACTTCCTTAAGTTTA

CTGATTCGTGCTGAATTAGGAAACCCAGGATCTCTAATTGGAGATGATCAAATTTATAACGTAATCGTAACAGCTCATGCTTTCATTATAATTTTTTTTA

TAGTAATACCTATTATAATTGGAGGATTCGGAAACTGATTAGTTCCTTTAATGCTTGGAGCCCCCGACATAGCATTTCCCCGAATAAATAACATAAGATT

TTGACTTTTACCTCCTTCATTAACTCTACTTCTAATAAGAAGATTAGTCGAAAGAGGGGCAGGTACAGGCTGAACAGTTTATCCCCCACTATCAGCCAAT

ATTGCCCATAGAGGAGCATCAGTTGATTTAGCAATTTTTAGACTTCATCTTGCAGGAATTAGCTCAATTTTAGGTGCAGTAAATTTTATTACTACTGTAA

TTAACATACGATCAACAGGAATAACTTTTGATCGAATACCCCTATTTGTTTGATCTGTAGTTTTAACAGCACTTCTTCTGCTATTATCCCTCCCAGTTTT

AGCAGGAGCAATTACTATACTATTAACAGATCGAAATATTAATACAACATTTTTTGATCCCGCAGGTGGAGGAGACCCCATTCTCTACCAACATTTATTT

TGATTTTTTGGTCATCCAGAAGTTTACATTTTAATTTTACCTGGATTTGGTATAATCTCCCATATTATTAGCCAAGAAAGAAGAAAAAAAGAAACATTTG

GAACTTTAGGTATAATTTACGCTATAATAGCTATTGGTTTACTAGGATTTATTGTTTGAGCTCATCATATATTTACAGTAGGTATAGATGTAGACACACG

AGCATATTTTACATCAGCAACAATAATCATTGCTGTTCCTACAGGAATTAAAATTTTTAGATGATTAGCTACTCTCCATGGATCACAATTAAACTACTCC

CCGTCTCTTTTATGGGCATTAGGATTTGTATTCCTATTTACAGTAGGAGGATTAACAGGAGTAATTCTAGCTAATTCATCAATTGACATTATTTTACATG

ATACTTACTATGTAGTTGCACATTTCCATTATGTCCTTTCCATAGGAGCTGTATTTGCTATTATAGCAGGTTTTGTTCATTGATTCCCTTTATTTACAGG

TTTAACAATAAATTCAAAATTTCTTAAAATTCAATTTTTAACAATATTTATTGGTGTTAATATAACATTCTTCCCTCAACATTTCTTAGGATTAAGAGGA

ATACCTCGACGTTATTCAGATTACCCAGATGCTTATACAACTTGAAATATTATTTCATCTATTGGATCTTTAGTTTCTTTAATTAGTATTTTTATCTTTT

TATTTACTATTTGAGAAAGGCTAATTTCATTACGAAAAAGAATTAGGTCTTTAAGAATATCTACATCAATTGAATGACTCCAACAAATACCCCCTTCAGA

ACATAGTTATTCTGAACTTCCAATGCTTACTAACTTCTAATATGGCAGATTAGTGCAATGGATTTAAACCCCAAATATAAAGATTAAACTTTTTTTAGAA

ATAGCTACTTGAAATACCATTTTACTTCAGGATAGGGCATCCCCATTAATAGAGCAACTCTCATTCTTTCATAACCATGCTCTTCTAATTCTCTTTATAA

TTACCGTTCTAGTAGGTTATTTAATAGGAACTTTATTTTTTAACCAATTTAATTACCGATTTTTATTAGATGGTCAAACTATTGAAATTATTTGAACTAT

TTTACCTGCTGTAACACTAATTTTTATCGCATTACCGTCTTTACGCTTACTTTATCTTCTAGATGAAGTTAATAACCCTTTAGTAACTATCAAAACAATT

GGGCATCAATGATATTGATCATACGAATATAGAGATTTTATAAATTTTGAATTCGATTCCTATATAATTCCTTTGACAGAAATAAAACCTCAAAATTTTC

GTTTATTAGATGTTGATAACCGAGTAATTGTCCCCTTTAACTCCCAAATCCGAATGATAGTAACAGCTGCCGATGTTATTCATTCATGAACTATCCCGGC

TTTTAGTGTAAAAATTGATGCAACACCAGGCCGACTAAATCAAATTAGATTCCTAATTAATCGAACAGGATTATTTTATGGTCAATGCTCAGAAATTTGT

GGGGCAAATCATAGATTTATACCTATTACTGTAGAAAGAATTTCACCTTCATTTTTTACTAAATGAATCTCAAAAATAAATAACCTATCATTAGATGACT

GAAAGTAAGTAATGGTCTCTTAAACCAATTAATAGTAGTTTAACATCTACTTCTGATGGCCAAAAATTTAGTTAAGATATAACATTAGTTTGTCATACTA

AAATAATCATAATTTGATAATTTTTAATTCCACAAATAGCACCTTTAAACTGACTATCTTTATTTTTTTTAATTATTATTATTTTTTTACTTTTTAATGT

ATTAAATTACTTTAGATTCTTACAGCCCTTAAAAACCCAATCTCATAACCCTACAATTAAAAAAATTAATTGAAAATGATAACTAATTTATTTTCATCTT

TTGATCCTAGAACTTCTTTTAATTTAAGATTAAACTGATTAAGAATACTATTAGGGCTAATATTTATCCCCCCAATATTTTGATTAGTTCCTTCACGCTA

TAATTTTCTATGAATTAAAATTATTTTAACATTACACCAAGAATTTAAGGTTTTAATTGGTAATAATAATATTAAAGGAAGAACCTTAATATTTATTTCA

TTATTTTCTATAATTGTTTTCAATAACTTTTTAGGATTATTTCCGTATATTTTTACAGGAACAAGACATTTAATTATAACATTATCTCTTGCCTTACCTT

TATGAATTAGATTCATATTATACGGGTGAATTAATAACACTACCCACATACTTGCTCATTTAGTCCCCCAAGGAACACCCCCAGCTCTTATGGCATTCAT

AGTAGTAATTGAATCAATTAGAAATATTATTCGTCCTGGTACTTTAGCTGTTCGATTAGCTGCTAATATAATTGCTGGACATTTACTAATAACTTTACTA

GGAAACACAGGAGTAAATTTATCAATTTTTATACTAAGTATTCTTATTATCACACAAATTCTTTTATTAATTTTAGAATCTGCTGTTGCGATCATTCAAT

CTTATGTATTTGCTGTATTAAGAACTTTATACTCTAGAGAAATTAATTAATGTCAAGACATAAAAATCACCCTTATCATTTAGTTGATGCAAGACCTTGA

CCTATTTTAGGTGCTTTTAGAGCTATAATTACAATAATTGGAATTATCAAATGATTCCATTTTTATAATAATTCTTTATTTTACTTAGGGACATTAATCA

CAATTTTAATTATAATTCAATGATGACGAGATATCACTCGTGAGGGAACTTTCCAAGGACTTCATACTTACGCTGTAACTATAGGTTTACGTTGAGGAAT

AATTTTATTTATTACATCAGAAGTATTTTTCTTTATTTCTTTTTTTTGAGCCTTTTTTCATAGTAGCTTAACACCCGCTATTGAACTTGGGATACTCTGA

CCACCCAAAGGAATTACCCCATTTAACCCTATTCAAATTCCATTATTAAACACTTTAATTCTTTTAACTTCAGGATTAACTGTAACTTGAGCTCACCATA

GATTAATAGAAAATGACTATAACCAAACAATGCAAGGTCTTGGTTTAACAGTTTTACTAGGAGTATATTTTACTTTATTACAAGGTTACGAATATTTAGA

AGCCCCCTTTACTATAGCAGATTCTGTTTATGGATCAACATTTTTTATTGCTACTGGTTTCCATGGATTACATGTTATTATTGGCACAACCTTTTTAGCT

GTTTGTTTAATACGGCATTTTAATAACCATTTTACTTGTATCCATCACTTTGGATTTGAAGCTGCTGCTTGATACTGACATTTTGTTGATGTAGTATGAC

TATTTCTTTATATTTCTATTTACTGATGAGGTAGATATTTATATAGTATAATAATTATAATTGATTTCCAATCAAAAGATCTAAAAAAA-TTAGTATAAA

TAATCATTATAATTTGAAATATAGGGTTAATTATTTTTTCTATCTCTTTTATCTTAATTATACTATCTTTTACAATCTCTAAAAAAAGATTTATAGACCG

AGAAAAAGCTTCTCCATTCGAGTGTGGATTTGACCCCAAAAGATCAGCCCGTTTACCCTTTTCTTTGCATTTTTTTTTAATTGCAGTAATTTTCTTAATT

TTTGATGTTGAAATTACTCTTCTTATCCCCTTAATTCTAACAATAAAAATTACTAATATTACTGTATATACCTATATTGCTCTTTTCTTTTTAATGATTC

TTTTAATAGGACTTTACCATGAATGAAACCAAGGGGCCTTAAATTGAGCTCTTTAGGGTAATAGTTAAGTATAACATTTAAGTTGCATTTAAAAAGTATT

GATTTTTCAATTTACCTTAAATAAGAAACAATTAATTGTATTTAGTTTCGACCTAAAATTTAGGTGTATGAACACCCTTATTTAAATTAATTGAAACCAA

AAAGAGGTATATCACTGTTAATGATACTAATGAGAAAAACTCCAATTAAGGAAATAAGATATTCAAGAGTAAGCTTCTAACTTAACTCTTTAGCAGTGAA

AGTCTGTTAATATTTCTATTTATATAGTTTAATAAAACATTATTTTTTCATAATAAAATTAGAATAAATTTATTCTTATAAATATTTAAAAGTAAATTTT

ACTTCCCTGATAACTTCACTATCATACTCTATATAAGCTATTTAAATTAAATATATAAAATTATAAAAATTACCCATATTATAATTAAAAGTATAAAAAT

CTTATAATTGTTATTAAATATAAACTGTAAAAATACAGAAGTATTACTAATTTTACTATATAAATTTTGTCTTCCATAATACTCTGATCAGCCTTGATCA

ATAGTTTTATATAACTTACTACCTAATTTAATTGGATAATAATTTAAACCAAATGTTGAAATATAGGGTATATTCCACATAGAAGAAAGAAAAAGTCTTG

AATTTAACAAATAAAAAGATTTTAATCTATCATTTAAAGTAAACTTAGAAAGTTCAAATCCAAACCAAGCCCCAAAAAATGATACAATTAAAGCTATAAT

TTTTATTGTAAAAGGTAAACAAATAAAATAAGGTGTTGGGAATATCAACCATATTAATATTCTTCCCCCAACAATAACTAAAAAAATTAAACCTGATATC

CCTTGAAGTATAATTTTTCTATTATCATTAATTTTACTTAAAGAATAAAAACAAAAATTTCCTACCAAAACATAATAAATTAAACGAAATGTATAACAAA

CAGTTAATCCTGTTGAAAAAAAGAAAATAACATAAATATAGATATTTAAGTATCTTATAGATAAAACCTCTAAAATTAAATCCTTTGAATAAAACCCCGA

TAAAAAGGGTAAACCACATAATGCTAAATTTGAAATTATAAAATAAGTACAAGTTAAAGGTATAACTTTAATTAACCCCCCTATATACCGAATATCTTGA

CAATTTCTTAATCTGTGAATTATACACCCTGCGCATATAAATAATAAAGCCTTAAATAAAGCGTGAGTTAATAGATGAAAAAAAGCTAATTGATACTCCC

CTAAAGCTAAAATACTAATTATCAAACCCAATTGTCTTAAAGTAGACAGAGCAATAATTTTTTTTAAATCAAACTCAAAGTTTGCGCCTATTCCTGCTAT

AAATATAGTTATTGTTCCAATAAATAATAAAATTAATATTAAATTACTTGTTAATGCAAAATTAAAACGAATTAATAAGTAAACACCTGCTGTTACTAAG

GTAGAAGAATGAACTAAAGAAGAAACAGGTGTTGGAGCCGCTATTGCCGCAGGCAACCAAGAAGAAAATGGAATTTGAGCTCTTTTAGTTATAGCTGCTA

ATATAATTAATAAAATAATAATATATATTTCTATACTATTTTTATATACATCAATATAAAAAATATAGTTAAATCCCCCAAAATTTATTATTCATGCAAT

TGCTATTAATAAAGCAACATCTCCAATACGGTTAGTTAAAGCTGTAATTATCCCTGCATTATAAGATTTAATATTTTGATAATAAATAACTAAACAATAA

GAAACTAACCCTAACCCATCTCATCCTAATAAAATCCTAATTAAATTAGGAGAAATAATCAATAACATTATAGATAAAACAAATATAGAAACTAATATAA

TAAATCGATGCAAATAAATATCTCCTTCTATATACTCTTCACTATAGTAAATTACTATAGAAGAAATAAATAAAACAAAACTCATAAATAATAATGACAT

TCAGTCAAGTAAAATGGTTATAATAATTCTACAAGAATTAATCCTTAATATTTCATATTCTAATATTAGTCTATAATCTAAAATTATGAAATTTAAGCTT

AATAAAAATCTTAATACTCTAAAAAATAAAAACGTTACAAAATAAATTAAACAAATAGAAATAATTTAAAGTAAATTTTACATCTTTGATACCACAAATC

AATATTTTTTATTAAACTATTTAAATTACAATCATAAAACTAAAAATTCTCTCTTTAAAATTAAGATATTCAGGGGTAACCAATGAAGTAATAATAATAA

ATACTCACGAACAAATCCTCTTGAAAAAGAATATAAATTTCTAACTAACTTGCCGTGTTGACTATAAGAATATAAATATAAAGAATACGCAGCTCTAAAA

AAAGATATTAAAGATAAAAAAACCATTGTTCAACTCCTTCATCTAACTAATCTATTAATTAAAATAATCTCACCCAGTAAGTTTAATGAAGGAGGAGCCG

CTATATTACAACAACTAAATAAAAATCATCATATTCTTATTATTGGTATTAAATTGATTAGCCCCTTATTTAAATAAATACTTCGGCTATTTAACCGTTC

ATAGGAAATATTTGCTAAACAAAATAATCCTGAAGAACATAAACCATGGGCAATTATTATAACTAAAGCCCCTCTTATACCCCAATAACTTAAAGTTAAA

ATCCCTCTTAATACTAAACCTATATGCGCTACAGAAGAATAAGCAATTAAAGCTTTAATATCAACTTGACGTAAACATATTAATGAAACAAAAAATCCCC

CTACTATTCTAATTGTAATAAAAATATAGTTTACTTGTAAACCTACAGTTAAAAAAATATTTATTAAACGTATTAATCCATACCCCCCTAATTTTAATAT

AACTCCAGCTAAAATTATAGAGCCAGCAACTGGAGCTTCAACATGGGCTTTAGGGAGCCAAAGATGAACAAAAAATATAGGCATTTTAATAAAAAACACT

ATATTTATACATATAAATAACAAGAATCTTTTTACATCATAACATAAAAAGAAAAAATCTAAAGAATGAAATTTTTCATAATAATAAAAAATCCTAATTA

TTATAGGTAATGAAGCAAATAATGTATAAAATAACAAGTAAACACCTGCTTGCAAACGCTCAGGTTGATACCCCCAACCAATAATTAATAAAAGAGTAGG

AATTAAGCTAAACTCAAAAAACAAATAAAAAATAAATAAATTTAAAGAACTAAATGTTATAACTAAAGATAATAATAAAATAATTATTACTAATAAAAAT

AAATTATAAAAATAGTTTTTTCTATAAATACTTTCAGAAGCTAATAATATTAAAGAACAAATCCACAAACTTAATAAAATTATTATAAAAGATAAAAGGT

CATACCCTATAAAATAAGAAATATTTATATACAAATAATTAAAACTAAATCTTAACCCAAATATAAATGTAATAAAAAAATATATATATTGATTAAATCA

GTATCTCTTTTTAATACAACTTAAAGGTACCAATATTAATATTATAAAAATAAACTTTATCATAATACATTAAAAGTTTGAAAATAATCATTTCCATGTG

TTCGTATTATAGAAACTAATAACGATAAGCCTAAAGCTCCTTCACAAACTCTTATAGTTAAAAATACTATACCAAAATAAAATTCAAAATTAAAATATAT

TAAAAATAGATATAAATTAAAGTATAATCCTAAAATAATATACTCTAACCTTAATAATATTAAAAGTAAATGTTTACGTTTAATACAAAAAGAAACTAAT

CCTGTAAAATATATAATCACTGAAAATAACATACAAAAAATTAACATTAGTTTTAATAATTTAATAAAAATACTGGTCTTGTAAATCAGAAATAAGGATT

TTCTTTTAAAACTTCAGAGAAAGAGTAAACCCCTATCATTAATCTCCAAAATTAATATTTTAAATAAACTATTCTCTGTATAATCTTATTACTAATAACA

TTATCTTTTATTAGTTCAATAACTTTTATATTTTTAAGTCACCCTTTATCTATAGGGCTTATTTTATTAATACAAACTATTATTATAGCTTTAACTATAG

GTTTTTTTAATATTAATTTCTGATATTCTTATATTTTATTTCTTATTATAATTGGAGGAATATTAGTTTTATTTATTTATATAACAAGAGTAGCTTCAAA

TGAAAAATTTTCATTTTCTATTAAAATTACATTAATAATTAGAATTATAACTTTAGGATTTTTATTTAGTATTGCTATAATAGACCCCTACTTTTCAGAC

ATCAACTCAATTTACACAGAAAACTTAGATAATTATAAAGAATATAATATATCATTTAGAAAATATTTAAGTTATCCTAATATTATCATTATATACATAA

TAATTATTTATTTATTAATTACATTAATTGCAGTTGTAAAAATTACTCAAATTGAAAAAGGACCTTTACGTCAAACTAACTAATGAAAACACCATTACGA

AAAGCTTCCCCCCTATTAAAAATTATTAATAATAGAATTATTGATTTACCTACACCATCTAATATTTCTGCTTGATGAAATTTCGGATCCTTATTAGGAC

TCTGTCTTTTTATTCAAATTATCACTGGAATTTTCTTAGCTATACATTTTACAGCCCATATTGATATAGCATTTAATAGAGTAATTCATATTTGTCGAGA

TGTAAATTATGGCTGATTATTACGAACAATTCATGCTAACGGGGCTTCTTTTTTCTTTATTTGTATTTATCTACATATTGGCCGAGGAATATATTATAGA

AGCTATAATTTACATTTAACATGAACTATTGGAGTAATCATCCTTTTTATAGTAATAGCTACAGCATTCTTAGGGTATGTATTACCTTGAGGGCAAATAT

CTTTTTGAGGGGCTACTGTTATTACCAATCTTCTCTCTGCTATTCCTTATCTAGGAAATATGATTGTCCAATGATTATGGGGGGGATTTGCAGTAGATAA

CGCCACTTTAACTCGATTTTTTACATTACATTTTATTCTTCCTTTTATTATTTTAGCTTTAATAATTATTCATTTATTATTTCTTCATCAAACTGGTTCT

AATAACCCTTTAGGATTAAATAGAAATATCGATAAAGTGCCTTTCCATCCATATTTTACTTACAAAGATACTTTTGGATTCATTATAATAACTATATTAT

TAATATTTTTAGTTTTAATTAATCCCTATCTTTTAGGAGACCCAGAAAACTTTACACCCGCTAACCCTTTAGTAACCCCAGTCCATATTCAACCAGAATG

ATACTTTTTATTCGCATACGCTATTTTACGATCAATTCCTAATAAATTAGGTGGAGTAATTGCTTTAGTTATATCAATTGCTATTTTATTAATTATACCT

TTAATTAACAAAAAAAAATTTAGTAGAACTCAATTTTACCCATTAAATAAAATTTTATTTTGATCTTTTGTCTCTATTGTAATCTTATTAACATGAATCG

GAGCTCGCCCAGTGGAAGATCCATACATTTTAACAGGTCAAATTTTAACAATTATATATTTTAGTTATTATTTCTTAAACCCCTTAATTCATAAAATATG

AGATTATATTATTTTTAAAACTTAGTTAATGAACTTGTTAAAGTGTATATTTTGAAAATATAAAAAAGAGTTTATTCTCTATTAACTTTACTAAATTTTA

TTCACTAAATAAAATGAGAAAAGATAACCAACTTTAAACCTAAAAAAAAAAATAAAAAATTTAAAGAAACTGGTAAATAACTTTTTCAACATATATATAT

TAACTTATCATAACGATACCGAGGTAAAGTACCTCGAACTCAAATCCAAATAAATGCTATAAAAGTTAACTTCAAAAAAAAAAATAAAGAAAAAATATCC

CCCCCTATAAATAACAACACACATAATATACTTATAAATAAAATTCTAGCATACTCAGCCAAAAAGATTAAAGCAAATCCCCCTCTTCTATACTCTACAT

TAAACCCAGAAACTAACTCTGATTCTCCTTCTGCAAAATCAAAAGGAGTCCGATTAGTCTCAGCTAAACTTGAAGATAATCATATTATTCTTAAAGGTAA

GCACAGAAAAAAAAATCATACTATTTCTTGATATTTTATAAAATCTATTATATTTAAATTTAAAATTAACAATAAAAAAGATAATAAAATTAATGATAAA

CTTACTTCGTATGAAATTGTTTGAGCTACTGATCGAATACCCCCTAATAAAGCATAATTAGAATTTGATGATCAACCAGAAATTATGATAGTATAGACCC

TTAGCCTAGAACAACATAAAAAATATAAAATACCTAAATTAAACCTAAATATAAATGTCAAAAAAGGTATACATATCCATAATAAAAGAGCTAAAAATAA

ATTAAAAACTGGAGATATATAATAAATAAAAAAATTTGATATTAAAGGATAAGTTTGTTCTTTAGAAAATAACTTAATAGCATCACTAAAAGGTTGAGGA

ATACCTATAAATCCAACTTTATTGGGCCCTTTACGAATTTGAATATACCCTAAAACTTTGCGCTCTATTAATGTTAAAAAAGCTACACCAATTAAAACAC

AAATAATTAAAACTAAACTTGTAAATAATAAAAGAAATAAATCTTGTAATATAATGTATTACTTGTGTTAAACACATATTTAAATTCTAAATTTAAAGCA

CTAATCTGCCAAAGTAATATTCATATTCAAATTATATTAAATTTTAAAGGTATTTGATCCTTTCGTACTAAAATACCTATGTTTTTTAAAGATAGAAACC

AACCTGGCTCACGCCGGTTTAAACTCAGATCATGTAAAATTTTAAAGGTCGAACAGACCTAACCTTTTAGCCCCTACACCAAAAGTTAATTTTAATCCAA

CATCGAGGTCGCAAACTTTTTTTTCGATAAGAACTCTAAAAAAAAATTACGCTGTTATCCCTAAGGTAATTTAATCTTGTAATCATTAAAAATGGATCAT

TCAATCATAAATTAATGTTTTTAAATAAAAAAAGTTTAATCAATTTTTCTGCTGCCCCAGCAAAATAGTTTAAATTATTAAATATATAAATATACTAAAA

TTAAATAATAATTTAAACTATAAAACTCTATAGGGTCTTCTCGTCTTTTAAAATTATATAAGCTTTTTTACTTATAAATAAAATTCTATATTCAATTAAA

TTGAGACAGTTACTTTCTCGTCCAACCGTTCATTCCAGCTTTCAATTAAAAAACTAATGATTATGCTACCTTTGCACGGTCAAATTACCGCGGCCATTCA

AATCCTCATTGGGCAGGTCAGACTTTAAATTATAATCAAAAAGACATGTTTTTAATAAACAGGCGAAAAGTGTATTTGCCGAGTTCCTTAATTTAACCTT

GAAGTTTTAATTTAATTACTAAATTAAAATATATACTAATTTTATCATTATTCTATATAAACCAATATTACATATATTATCTTAATAAACTACTTAAAAA

TAATATAAATCTTATTCTAACAAAAATTATTTATAACAAACTAAAGATTAACACTTCCAATTCTACTAATTTTTATTCAAAATATACATTTTTAACATTT

TATTTTAAAGCTTATCCCCTAAAATATTACTTTTTATATATAAAATACTAAATAATTAATATAATACAATAAAAAAACTAAATTAAATTTATTTCTTAAG

AAACTAGATATCTTAAAAAACGTATAACGTTTCATTTCTAATATAATATTTTAAAAATTTATGCCACAATTAAATTTATATTATATTAGCTCTTTATAAT

TCGAGAACACTAAATAATTAAATTATTTTAATAAACCCTGATACACAAGGTACAAAAAATTAATTTTTCTTTTTAAAAAATAAATCTCTATATATTTATA

TTATCTATCTCTATACAAATTAACTATAATAAAATTTTTATATTCTAAAATATACTAATATCAAAAATATTTTTTTTATATATATATATATA----TTAT

AAATTTTTCCTTTCAAATTAAATTGATTTTCACAACTAACTTTTTAATGTAAATAAAATGCTTTATTACAAGCTCTAATTTGCCATTCCAGGTACACTTT

CCAGTACACCTACTATGTTACGACTTATCCCTCTTTAGAGAGGGAGCGACGGGCGATATGTACATATTCTAGAGCTATACTCATATAATTAAACTAAACT

ATATTACTTTCAAATCCACTTTATAAAATAATGTTAATTATTTTAACCATCTAAATAATTTTATTGTAACCCATCTCTCCTTATCTATACGCTGTATCTT

GATCTGATTTTTTTTATACTTATAAATTTTGAACATTCCAAATTCTTTAAAAACATTCAACCTACGACGATATACAAACCTTTAAAATAAGTACGATTAA

TCGTGGATCATCAATTATAGGACAGGTTCCTCTGAGTAGACTAAAATACCGCCAAATTCTTAAAATTTCAAGAACATAACTACTACTATTCAAGCATCTA

AAATTTGCATTTTTAATAATAGGGTATCTAATCCTAGTTTTTTATAAAAATCTCATAAACTCATTTTTCACATTTAAAAAATTAATTATACTTACTAATT

TCACCTAATAAATACAATATAAATTAATAATAAAATAACTTATTATATACTGAACAAATTTAATTGCATTGTTTGTGTAACCGCAACTGCTGGCACAAAC

TTGGTCAATACTATTATAAATTCCTAAATCAAAATTTCTTTTAAATTTAATCTTCACTATTGCAATTCTTTAATTAAATATACAGAATAATTTATTTTTT

TAAAATAAATTCATTAAACACTAAAATTTACATATAAAATAATTTAAAAATTAAAATCTCAAGCTAGAATAAAACTTTATTTTTTTTTCAATATACATAA

ATTATATAAAATAAAAGTACCCCCC-TACTATTTTAATTAAGTGAATTAATTTTTAATGCCTTAAATTTATTCCCAATTTATTTTTATTATATAGTTTTT

TATTTAAAAATAAAACTTTATACCTAAAATTATATACATAAATTATATTTAAAATTTATAATTTAAAGAACTATAAAAATTAAATTAAAATTTAAAATCT

ATCTAATTTATAGATAAACAAATTAAATTAAACTAACTTACCTGATAAATTTAACTATATACGCGCAGGTATATTAATTATTTAATTATAATATAATACA

TTTGTTTTATTAAATAATTATTAAGCAAAATAGCATTAATTTATTATATAAAAGATCTAGATACTATTAATTTAAAGTAAATTTTTTTTTTTTTTGTAGT

ATAATTTATACTAATATACAGCATTTAATATCTTCTATATAAATGTTTATATTATTTATATTTATCTATTTATAATTCTTAATAATAATTTAGATAATTA

TTAATTTATATATTAAATATTTATATATATATATATAATAATAAGGATATATATATATATATGTATATAGATAAATTAAATTTAGTTATATATAAAAAAA

TTAATATTAATTTTACTTTTAATTTTTTCTTTAATTTAAACTATTGGATATTCAGATTCTAATATATTATAATATATCAACCATTAATGAATAACTTTAT

ATATATAATAAAAATGTTAATTAATATATCAATTCATATGAATATATAATATTAATAAATAAATTCTTAATAAATAATAATGATAATATATATATATTGT

ATATTAATGTACTATATATATAAATTCATTAAACATTTATATTTGATACCCCCCCAAAATTTTTTCTAAAAATGTGAAGTTTCAATTTAATTAAAATTTT

CTACAATGGTCAAAAACGGCCAAAAAATGGAAAATAAAATGTGCACAGAAAATGCACATAAAATGCACATTTTTGAAAATTTCCGATTTTTGAAAAATCG

TTTGAACCAAACCTCTGATGTACCTTTTTTCGCCCCCCCTGATTACTAAAATTTATCCTCAATTACTAAGGTTTATCCGCATTTTTGCGACAGGAATTTT

GAGTATAAAATCAATTCCACACTAAATTTTTTTTT-TTTTTTTTGTGTCATTTCTCCAACATGGGCATTTTTTTTTTTAGTCGAAGGACAAATTTTAGTA

AAAAAAGTTTGAAATTAGGTTAAACCCTTGTTTAACCAAAAACTATTACTTTTCAATATTATACCCAATTTTATTTAAAATAAATTTTATTATACTTTTA

TTTATGCAAAAAGATCTTCTTATAAACTTTT

>DMR76j_JP_Mori_Honshu

AATGAAGTGTCTGACTATAGAGTTACTTTGATAGAGTAAAAAAAGTGAATTTTCACCTTCATTATAATTAACAGAATTAAACTATTTCTTTAAGCTTCAA

AAACTTATGTACATTATATACTAAATTATAAAAAGATAAGCTAATTAAGCTACTGGGCTCATACCCCATCAATAAAGGTTACAATCCTTTTCTTTTTAAT

GTATTATAAGCTTTTATTTTTTAATTCTCTTATAATTGGAACCTTAATTGCTATTTCTTCCTACTCTTGAATAGGAATATGAATAGGATTAGAAATTAAT

CTTCTCTCTATGATCCCCCTCATTAGAGACAACAAAAATATAATAGCCTCAGAAGCTGCTTTAAAATATTTCATTATTCAAACAATAGCATCAACATTGT

TATTATTCTCAATTATTATAATATCAATGAAATTTATATATCAAATAAATTTAATCACTTACTTTAATTTAATTTTTAACACTTCATTGTTCATCAAAAT

AGGAGCAGCCCCATTCCATTTTTGATTCCCCGAAATAATAGAAGGATTAAATTGATTAAATGCCATTATCATACTTACTTGACAAAAACTAAGGCCCATA

GTATTACTAACGTATTCTAATACAACCTCCATATATCTAATTTTAACAATTATATTTAGAATAATAATCAGAGGAATTATAGGTCTAAATCAAACTAGAT

TACGGAAAATTATAGCTTATTCATCTATCAACCATATTGGATGAATAATCAGTTCAATTATATTAATTGAAATTGTTTGATTTTACTATTTTATTATTTA

TTGCATTATTACTATTAATATCAGAATTATATTTATAAAATTAAATGTTTTTCATATCAATCAATTATATATTTCAATAAATTACCATATTTTACTTAAA

TTATTCTTTGCCTTAAATTTCATGTCTTTAGGAGGATTACCCCCATTTTTAGGGTTTTTCCCTAAATGACTTACAATTCAAACTTTAATCCAAAGAAATA

TATACTCAATTGCTTTCATTATAATTTTAATAACTTTAATAACACTTTTTTTTTACCTCCGAATTACTTTTTCAATTTTACTACTAAGAAAAACAGTTTT

AACATTTTACACACAACCAAAAATTTATACTAATTACATTATAGCATTTAATTTTATTGCATTATTAAGATTAATTTTCGTTACTTTGATATTCAATTTC

TTATAAATTAAACCTGAAGGATTTAAGTTAAATTAAACTAAGAACCTTCAAAGTTCTAAATAAAGTAAATTCTTTAAGCCTTAGGGCTTAGCCCATCTTT

AAATTTGCAATTTAAAATTCTTTTTGAACTATAAAGCTTGATAAAAGAAACTAATTTCGTATGTAAATTTACAGTTTACCGCCTAAACCTCGGCCATTTT

ATCGAATAAATGGCTATTCTCTACAAATCACAAAGATATCGGAACTTTATACTTTCTATTTGGAAGTTGGGCAGGCATAGTAGGCACTTCCTTAAGTTTA

CTGATTCGTGCTGAATTAGGAAACCCAGGATCTCTAATTGGAGATGATCAAATTTATAACGTAATCGTAACAGCTCATGCTTTCATTATAATTTTTTTTA

TAGTAATACCTATTATAATTGGAGGATTCGGAAACTGATTAGTTCCTTTAATGCTTGGAGCCCCCGACATAGCATTTCCCCGAATAAATAACATAAGATT

TTGACTTTTACCTCCTTCATTAACTCTACTTCTAATAAGAAGATTAGTCGAAAGAGGGGCAGGTACAGGCTGAACAGTTTATCCCCCACTATCAGCCAAT

ATCGCCCATAGAGGAGCATCAGTTGATTTAGCAATTTTTAGACTTCATCTTGCAGGAATTAGCTCAATTTTAGGTGCAGTAAATTTTATTACTACTGTAA

TTAACATACGATCAACAGGAATAACTTTTGATCGAATACCCCTATTTGTTTGATCTGTAGTTTTAACAGCACTTCTTCTGCTATTATCCCTCCCAGTTTT

AGCAGGAGCAATTACTATACTATTAACAGATCGAAATATTAATACAACATTTTTTGATCCCGCAGGTGGAGGAGACCCCATTCTCTACCAACATTTATTT

TGATTTTTTGGTCATCCAGAAGTTTACATTTTAATTTTACCTGGATTTGGTATAATCTCCCATATTATTAGCCAAGAAAGAAGAAAAAAAGAAACATTTG

GAACTTTAGGTATAATTTACGCTATAATAGCTATTGGTTTACTAGGATTTATTGTTTGAGCTCATCATATATTTACAGTAGGTATAGATGTAGACACACG

AGCATATTTTACATCAGCAACAATAATCATTGCTGTTCCTACAGGAATTAAAATTTTTAGATGATTAGCTACTCTCCATGGATCACAATTAAACTACTCC

CCGTCTCTTTTATGGGCATTAGGATTTGTATTCCTATTTACAGTAGGAGGATTAACAGGAGTAATTCTAGCTAATTCATCAATTGACATTATTTTACATG

ATACTTACTATGTAGTTGCACATTTCCATTATGTCCTTTCCATAGGAGCTGTATTTGCTATTATAGCAGGTTTTGTTCATTGATTCCCTTTATTTACAGG

TTTAACAATAAATTCAAAATTTCTTAAAATTCAATTTTTAACAATATTTATTGGTGTTAATATAACATTCTTCCCTCAACATTTCTTAGGATTAAGAGGA

ATACCTCGACGTTATTCAGATTACCCAGATGCTTATACAACTTGAAATATTATTTCATCTATTGGATCTTTAGTTTCTTTAATTAGTATTTTTATCTTTT

TATTTACTATTTGAGAAAGGCTAATTTCATTACGAAAAAGAATTAGGTCTTTAAGAATATCTACATCAATTGAATGACTCCAACAAATACCCCCTTCAGA

ACATAGTTATTCTGAACTTCCAATGCTTACTAACTTCTAATATGGCAGATTAGTGCAATGGATTTAAACCCCAAATATAAAGATTAAACTTTTTTTAGAA

ATAGCTACTTGAAATACCATTTTACTTCAGGATAGGGCATCCCCATTAATAGAGCAACTCTCATTCTTTCATAACCATGCTCTTCTAATTCTCTTTATAA

TTACCGTTCTAGTAGGTTATTTAATAGGAACTTTATTTTTTAACCAATTTAATTACCGATTTTTATTAGATGGTCAAACTATTGAAATTATTTGAACTAT

TTTACCTGCTGTAACACTAATTTTTATCGCATTACCGTCTTTACGCTTACTTTATCTTCTAGATGAAGTTAATAACCCTTTAGTAACTATCAAAACAATT

GGGCATCAATGATATTGATCATACGAATATAGAGATTTTATAAATTTTGAATTCGATTCCTATATAATTCCTTTGACAGAAATAAAACCTCAAAATTTTC

GTTTATTAGATGTTGATAACCGAGTAATTGTCCCCTTTAACTCCCAAATCCGAATGATAGTAACAGCTGCCGATGTTATTCATTCATGAACTATCCCGGC

TTTTAGTGTAAAAATTGATGCAACACCAGGCCGACTAAATCAAATTAGATTCCTAATTAATCGAACAGGATTATTTTATGGTCAATGCTCAGAAATTTGT

GGGGCAAATCATAGATTTATACCTATTACTGTAGAAAGAATTTCACCTTCATTTTTTACTAAATGAATCTCAAAAATAAATAACCTATCATTAGATGACT

GAAAGTAAGTAATGGTCTCTTAAACCAATTAATAGTAGTTTAACATCTACTTCTGATGGCCAAAAATTTAGTTAAGATATAACATTAGTTTGTCATACTA

AAATAATCATAATTTGATAATTTTTAATTCCACAAATAGCACCTTTAAACTGACTATCTTTATTTTTTTTAATTATTATTATTTTTTTACTTTTTAATGT

ATTAAATTACTTTAGATTCTTACAGCCCTTAAAAACCCAATCTCATAACCCTACAATTAAAAAAATTAATTGAAAATGATAACTAATTTATTTTCATCTT

TTGATCCTAGAACTTCTTTTAATTTAAGATTAAACTGATTAAGAATACTATTAGGGCTAATATTTATCCCCCCAATATTTTGATTAGTTCCTTCACGCTA

TAATTTTCTATGAATTAAAATTATTTTAACATTACACCAAGAATTTAAGGTTTTAATTGGTAATAATAATATTAAAGGAAGAACCTTAATATTTATTTCA

TTATTTTCTATAATTGTTTTCAATAACTTTTTAGGATTATTTCCGTATATTTTTACAGGAACAAGACATTTAATTATAACATTATCTCTTGCCTTACCTT

TATGAATTAGATTCATATTATACGGGTGAATTAATAACACTATCCACATACTTGCTCATTTAGTCCCCCAAGGAACACCCCCAGCTCTTATGGCATTCAT

AGTAGTAATTGAATCAATTAGAAATATTATTCGTCCTGGTACTTTAGCTGTTCGATTAGCTGCTAATATAATTGCTGGACATTTACTAATAACTTTACTA

GGAAACACAGGAGTAAATTTATCAATTTTTATACTAAGTATTCTTATTATCACACAAATTCTTTTATTAATTTTAGAATCTGCTGTTGCGATCATTCAAT

CTTATGTATTTGCTGTATTAAGAACTTTATACTCTAGAGAAATTAATTAATGTCAAGACATAAAAATCACCCTTATCATTTAGTTGATGCAAGACCTTGA

CCTATTTTAGGTGCTTTTAGAGCTATAATTACAATAATTGGAATTATCAAATGATTCCATTTTTATAATAATTCTTTATTTTACTTAGGGACATTAATCA

CAATTTTAATTATAATTCAATGATGACGAGATATCACTCGTGAGGGAACTTTCCAAGGACTTCATACTTACGCTGTAACTATAGGTTTACGTTGAGGAAT

AATTTTATTTATTACATCAGAAGTATTTTTCTTTATTTCTTTTTTTTGAGCCTTTTTTCATAGTAGCTTAACACCCGCTATTGAACTTGGGATACTCTGA

CCACCCAAAGGAATTACCCCATTTAACCCTATTCAAATTCCATTATTAAACACTTTAATTCTTTTAACTTCAGGATTAACTGTAACTTGAGCTCACCATA

GATTAATAGAAAATGACTATAACCAAACAATGCAAGGTCTTGGTTTAACAGTTTTACTAGGAGTATATTTTACTTTATTACAAGGTTACGAATATTTAGA

AGCCCCCTTTACTATAGCAGATTCTGTTTATGGATCAACATTTTTTATTGCTACTGGTTTCCATGGATTACATGTTATTATTGGCACAACCTTTTTAGCT

GTTTGTTTAATACGGCATTTTAATAACCATTTTACTTGTATCCATCACTTTGGATTTGAAGCTGCTGCTTGATACTGACATTTTGTTGATGTAGTATGAC

TATTTCTTTATATTTCTATTTACTGATGAGGTAGATATTTATATAGTATAATAATTATAATTGATTTCCAATCAAAAGATCTAAAAAAA-TTAGTATAAA

TAATCATTATAATTTGAAATATAGGGTTAATTATTTTTTCTATCTCTTTTATCTTAATTATACTATCTTTTACAATCTCTAAAAAAAGATTTATAGACCG

AGAAAAAGCTTCTCCATTCGAGTGCGGATTTGACCCCAAAAGATCAGCCCGTTTACCCTTTTCTTTGCATTTTTTTTTAATTGCAGTAATTTTCTTAATT

TTTGATGTTGAAATTACTCTTCTTATCCCCTTAATTCTAACAATAAAAATTACTAATATTACTGTATATACCTATATTGCTCTTTTCTTTTTAATGATTC

TTTTAATAGGACTTTACCATGAATGAAACCAAGGGGCCTTAAATTGAGCTCTTTAGGGTAATAGTTAAGTATAACATTTAAGTTGCATTTAAAAAGTATT

GATTTTTCAATTTACCTTAAATAAGAAACAATTAATTGTATTTAGTTTCGACCTAAAATTTAGGTGTATGAACACCCTTATTTAAATTAATTGAAACCAA

AAAGAGGTATATCACTGTTAATGATACTAATGAGAAAAACTCCAATTAAGGAAATAAGATATTCAAGAGTAAGCTTCTAACTTAACTCTTTAGCAGTGAA

AGTCTGTTAATATTTCTATTTATATAGTTTAATAAAACATTATTTTTTCATAATAAAATTAGAATAAATTTATTCTTATAAATATTTAAAAGTAAATTTT

ACTTCCCTGATAACTTCACTATCATACTCTATATAAGCTATTTAAATTAAATATATAAAATTATAAAAATTACCCATATTATAATTAAAAGTATAAAAAT

CTTATAATTGTTATTAAATATAAACTGTAAAAATACAGAAGTATTACTAATTTTACTATATAAATTTTGTCTTCCATAATACTCTGATCAGCCTTGATCA

ATAGTTTTATATAACTTACTACCTAATTTAATTGGATAATAATTTAAACCAAATGTTGAAATATAGGGTATATTCCACATAGAAGAAAGAAAAAGTCTTG

AATTTAACAAATAAAAAGATTTTAATCTATCATTTAAAGTAAACTTAGAAAGTTCAAATCCAAACCAAGCCCCAAAAAATGATACAATTAAAGCTATAAT

TTTTATTGTAAAAGGTAAACAAATAAAATAAGGTGTTGGGAATATCAACCATATTAATATTCTTCCCCCAACAATAACTAAAAAAATTAAACCTGATATC

CCTTGAAGTATAATTTTTCTATTATCATTAATTTTACTTAAAGAATAAAAACAAAAATTTCCTACCAAAACATAATAAATTAAACGAAATGTATAACAAA

CAGTTAATCCTGTTGAAAAAAAGAAAATAACATAAATATAGATATTTAAGTATCTTATAGATAAAACCTCTAAAATTAAATCCTTTGAATAAAACCCCGA

TAAAAAGGGTAAACCACATAATGCTAAATTTGAAATTATAAAATAAGTACAAGTTAAAGGTATAACTTTAATTAACCCCCCTATATACCGAATATCTTGA

CAATTTCTTAATCTGTGAATTATACACCCTGCGCATATAAATAATAAAGCCTTAAATAAAGCGTGAGTTAATAGATGAAAAAAAGCTAATTGATACTCCC

CTAAAGCTAAAATACTAATTATCAAACCCAATTGTCTTAAAGTAGACAGAGCAATAATTTTTTTTAAATCAAACTCAAAGTTTGCGCCTATTCCTGCTAT

AAATATAGTTATTGTTCCAATAAATAATAAAATTAATATTAAATTACTTGTTAATGCAAAATTAAAACGAATTAATAAGTAAACACCTGCTGTTACTAAG

GTAGAAGAATGAACTAAAGAAGAAACAGGTGTTGGAGCCGCTATTGCCGCAGGCAACCAAGAAGAAAATGGAATTTGAGCTCTTTTAGTTATAGCTGCTA

ATATAATTAATAAAATAATAATATATATTTCTATACTATTTTTATATACATCAATATAAAAAATATAGTTAAATCCCCCAAAATTTATTATTCATGCAAT

TGCTATTAATAAAGCAACATCTCCAATACGGTTAGTTAAAGCTGTAATTATCCCTGCATTATAAGATTTAATATTTTGATAATAAATAACTAAACAATAA

GAAACTAACCCTAACCCATCTCATCCTAATAAAATCCTAATTAAATTAGGAGAAATAATCAATAACATTATAGATAAAACAAATATAGAAACTAATATAA

TAAATCGATGCAAATAAATATCTCCTTCTATATACTCTTCACTATAGTAAATTACTATAGAAGAAATAAATAAAACAAAACTCATAAATAATAATGACAT

TCAGTCAAGTAAAATGGTTATAATAATTCTACAAGAATTAATCCTTAATATTTCATATTCTAATATTAGTCTATAATCTAAAATTATGAAATTTAAGCTT

AATAAAAATCTTAATACTCTAAAAAATAAAAACGTTACAAAATAAATTAAACAAATAGAAATAATTTAAAGTAAATTTTACATCTTTGATACCACAAATC

AATATTTTTTATTAAACTATTTAAATTACAATCATAAAACTAAAAATTCTCTCTTTAAAATTAAGATATTCAGGGGTAACCAATGAAGTAATAATAATAA

ATACTCACGAACAAATCCTCTTGAAAAAGAATATAAATTTCTAACTAACTTGCCGTGTTGACTATAAGAATATAAATATAAAGAATACGCAGCTCTAAAA

AAAGATATTAAAGATAAAAAAACCATTGTTCAACTCCTTCATCTAACTAATCTATTAATTAAAATAATCTCACCCAGTAAGTTTAATGAAGGAGGAGCCG

CTATATTACAACAACTAAATAAAAATCATCATATTCTTATTATTGGTATTAAATTGATTAGCCCCTTATTTAAATAAATACTTCGGCTATTTAACCGTTC

ATAGGAAATATTTGCTAAACAAAATAATCCTGAAGAACATAAACCATGGGCAATTATTATAACTAAAGCCCCTCTTATACCCCAATAACTTAAAGTTAAA

ATCCCTCTTAATACTAAACCTATATGCGCTACAGAAGAATAAGCAATTAAAGCTTTAATATCAACTTGACGTAAACATATTAATGAAACAAAAAATCCCC

CTACTATTCTAATTGTAATAAAAATATAGTTTACTTGTAAACCTACAGTTAAAAAAATATTTATTAAACGTATTAATCCATACCCCCCTAATTTTAATAT

AACTCCAGCTAAAATTATAGAGCCAGCAACTGGAGCTTCAACATGGGCTTTAGGGAGCCAAAGATGAACAAAAAATATAGGCATTTTAATAAAAAACACT

ATATTTATACATATAAATAACAAGAATCTTTTTACATCATAACATAAAAAGAAAAAATCTAAAGAATGAAATTTTTCATAATAATAAAAAATCCTAATTA

TTATAGGTAATGAAGCAAATAATGTATAAAATAATAAGTAAACACCTGCTTGCAAACGCTCAGGTTGATACCCCCAACCAATAATTAATAAAAGAGTAGG

AATTAAGCTAAACTCAAAAAACAAATAAAAAATAAATAAATTTAAAGAACTAAATGTTATAACTAAAGATAATAATAAAATAATTATTACTAATAAAAAT

AAATTATAAAAATAGTTTTTTCTATAAATACTTTCAGAAGCTAATAATATTAAAGAACAAATCCACAAACTTAATAAAATTATTATAAAAGATAAAAGGT

CATACCCTATAAAATAAGAAATATTTATATACAAATAATTAAAACTAAATCTTAACCCAAATATAAATGTAATAAAAAAATATATATATTGATTAAATCA

GTATCTCTTTTTAATACAACTTAAAGGTACCAATATTAATATTATAAAAATAAACTTTATCATAATACATTAAAAGTTTGAAAATAATCATTTCCATGTG

TTCGTATTATAGAAACTAATAACGATAAGCCTAAAGCTCCTTCACAAACTCTTATAGTTAAAAATACTATACCAAAATAAAATTCAAAATTAAAATATAT

TAAAAATAGATATAAATTAAAGTATAATCCTAAAATAATATACTCTAACCTTAATAATATTAAAAGTAAATGTTTACGTTTAATACAAAAAGAAACTAAT

CCTGTAAAATATATAATCACTGAAAATAACATACAAAAAATTAACATTAGTTTTAATAATTTAATAAAAATACTGGTCTTGTAAATCAGAAATAAGAATT

TTCTTTTAAAACTTCAGAGAAAGAGTAAACCTCTATCATTAATCTCCAAAATTAATATTTTAAATAAACTATTCTCTGTATAATCTTATTACTAATAACA

TTATCTTTTATTAGTTCAATAACTTTTATATTTTTAAGTCACCCTTTATCTATAGGGCTTATTTTATTAATACAAACTATTATTATAGCTTTAACTATAG

GTTTTTTTAATATTAATTTCTGATATTCTTATATTTTATTTCTTATTATAATTGGAGGAATATTAGTTTTATTTATTTATATAACAAGAGTAGCTTCAAA

TGAAAAATTTTCATTTTCTATTAAAATTACATTAATAATTAGAATTATAACTTTAGGATTTTTATTTAGTATTGCTATAATAGACCCCTACTTTTCAGAC

ATCAACTCAATTTACACAGAAAACTTAGATAATTATAAAGAATATAATATATCATTTAGAAAATATTTAAGTTATCCTAATATTATCATTATATACATAA

TAATTATTTATTTATTAATTACATTAATTGCAGTTGTAAAAATTACTCAAATTGAAAAAGGACCTTTACGTCAAACTAACTAATGAAAACACCATTACGA

AAAGCTTCCCCCCTATTAAAAATTATTAATAATAGAATTATTGATTTACCTACACCATCTAATATTTCTGCTTGATGAAATTTCGGATCCTTATTAGGAC

TCTGTCTTTTTATTCAAATTATCACTGGAATTTTCTTAGCTATACATTTTACAGCCCATATTGATATAGCATTTAATAGAGTAATTCATATTTGTCGAGA

TGTAAATTATGGCTGATTATTACGAACAATTCATGCTAACGGGGCTTCTTTTTTCTTTATTTGTATTTATCTACATATTGGCCGAGGAATATATTATAGA

AGCTATAATTTACATTTAACATGAACTATTGGAGTAATCATCCTTTTTATAGTAATAGCTACAGCATTCTTAGGGTATGTATTACCTTGAGGGCAAATAT

CTTTTTGAGGGGCTACTGTTATTACCAATCTTCTCTCTGCTATTCCTTATCTAGGAAATATGATTGTCCAATGATTATGGGGGGGATTTGCAGTAGATAA

CGCCACTTTAACTCGATTTTTTACATTACATTTTATTCTTCCTTTTATTATTTTAGCTTTAATAATTATTCATTTATTATTTCTTCATCAAACTGGTTCT

AATAACCCTTTAGGATTAAATAGAAATATCGATAAAGTACCTTTCCATCCATATTTTACTTACAAAGATACTTTTGGATTCATTATAATAACTATATTAT

TAATATTTTTAGTTTTAATTAATCCCTATCTTTTAGGAGACCCAGAAAACTTTACACCCGCTAACCCTTTAGTAACCCCAGTCCATATTCAACCAGAATG

ATACTTTTTATTCGCATACGCTATTTTACGATCAATTCCTAATAAATTAGGTGGAGTAATTGCTTTAGTTATATCAATTGCTATTTTATTAATTATACCT

TTAATTAACAAAAAAAAATTTAGTAGAACTCAATTTTACCCATTAAATAAAATTTTATTTTGATCTTTTGTCTCTATTGTAATCTTATTAACATGAATCG

GAGCTCGCCCAGTGGAAGATCCATACATTTTAACAGGTCAAATTTTAACAATTATATATTTTAGTTATTATTTCTTAAACCCCTTAATTCATAAAATATG

AGATTATATTATTTTTAAAACTTAGTTAATGAACTTGTTAAAGTGTATATTTTGAAAATATAAAAAAGAGTTTATTCTCTATTAACTTTACTAAATTTTA

TTCACTAAATAAAATGAGAAAAGATAACCAACTTTAAACCTAAAAAAAAAAATAAAAAATTTAAAGAAACTGGTAAATAACTTTTTCAACATATATATAT

TAACTTATCATAACGATACCGAGGTAAAGTACCTCGAACTCAAATCCAAATAAATGCTATAAAAGTTAACTTCAAAAAAAAAAATAAAGAAAAAATATCC

CCCCCTATAAATAACAACACACATAATATACTTATAAATAAAATTCTAGCATACTCAGCCAAAAAGATTAAAGCAAATCCCCCTCTTCTATACTCTACAT

TAAACCCAGAAACTAACTCTGATTCTCCTTCTGCAAAATCAAAAGGAGTCCGATTAGTCTCAGCTAAACTTGAAGATAATCATATTATTCTTAAAGGTAA

GCACAGAAAAAAAAATCATACTATTTCTTGATATTTTATAAAATCTATTATATTTAAATTTAAAATTAACAATAAAAAAGATAATAAAATTAATGATAAA

CTTACTTCGTATGAAATTGTTTGAGCTACTGATCGAATACCCCCTAATAAAGCATAATTAGAATTTGATGATCAACCAGAAATTATGATAGTATAGACCC

TTAGCCTAGAACAACATAAAAAATATAAAATACCTAAATTAAACCTAAATATAAATGTCAAAAAAGGTATACATATCCATAATAAAAGAGCTAAAAATAA

ATTAAAAACTGGAGATATATAATAAATAAAAAAATTTGATATTAAAGGATAAGTTTGTTCTTTAGAAAATAACTTAATAGCATCACTAAAAGGTTGAGGA

ATACCTATAAATCCAACTTTATTGGGCCCTTTACGAATTTGGATATACCCTAAAACTTTGCGCTCTATTAATGTTAAAAAAGCTACACCAATTAAAACAC

AAATAATTAAAACTAAACTTGTAAATAATAAAAGAAATAAATCTTGTAATATAATGTATTACTTGTGTTAAACACATATTTAAATTCTAAATTTAAAGCA

CTAATCTGCCAAAGTAATATTCATATTCAAATTATATTAAATTTTAAAGGTATTTGATCCTTTCGTACTAAAATACCTATGTTTTTTAAAGATAGAAACC

AACCTGGCTCACGCCGGTTTAAACTCAGATCATGTAAAATTTTAAAGGTCGAACAGACCTAACCTTTTAGCCCCTACACCAAAAGTTAATTTTAATCCAA

CATCGAGGTCGCAAACTTTTTTTTCGATAAGAACTCTAAAAAAAAATTACGCTGTTATCCCTAAGGTAATTTAATCTTGTAATCATTAAAAATGGATCAT

TCAATCATAAATTAATGTTTTTAAATAAAAAAAGTTTAATCAATTTTTCTGCTGCCCCAGCAAAATAGTTTAAATTATTAAATATATAAATATACTAAAA

TTAAATAATAATTTAAACTATAAAACTCTATAGGGTCTTCTCGTCTTTTAAAATTATATAAGCTTTTTTACTTATAAATAAAATTCTATATTCAATTAAA

TTGAGACAGTTACTTTCTCGTCCAACCGTTCATTCCAGCTTTCAATTAAAAAACTAATGATTATGCTACCTTTGCACGGTCAAATTACCGCGGCCATTCA

AATCCTCATTGGGCAGGTCAGACTTTAAATTATAATCAAAAAGACATGTTTTTAATAAACAGGCGAAAAGTGTATTTGCCGAGTTCCTTAATTTAACCTT

GAAGTTTTAATTTAATTACTAAATTAAAATATATACTAATTTTATCATTATTCTATATAAACCAATATTACATATATTATCTTAATAAACTACTTAAAAA

TAATATAAATCTTATTCTAACAAAAATTATTTATAACAAACTAAAGATTAACACTTCCAATTCTACTAATTTTTATTCAAAATATACATTTTTAACATTT

TATTTTAAAGCTTATCCCCTAAAATATTACTTTTTATATATAAAATACTAAATAATTAATATAATACAATAAAAAAACTAAATTAAATTTATTTCTTAAG

AAACTAGATATCTTAAAAAACGTATAACGTTTCATTTCTAATATAATATTTTAAAAATTTATGCCACAATTAAATTTATATTATATTAGCTCTTTATAAT

TCGAGAACACTAAATAATTAAATTATTTTAATAAACCCTGATACACAAGGTACAAAAAATTAATTTTTCTTTTTAAAAAATAAATCTCTATATATTTATA

TTATCTATCTCTATACAAATTAACTATAATAAAATTTTTATATTCTAAAATATACTAATATCAAAAATATTTTTTTTATATATATATATATA----TTAT

AAATTTTTCCTTTCAAATTAAATTGATTTTCACAACTAACTTTTTAATGTAAATAAAATGCTTTATTACAAGCTCTAATTTGCCATTCCAGGTACACTTT

CCAGTACACCTACTATGTTACGACTTATCCCTCTTTAGAGAGGGAGCGACGGGCGATATGTACATATTCTAGAGCTATACTCATATAATTAAACTAAACT

ATATTACTTTCAAATCCACTTTATAAAATAATGTTAATTATTTTAACCATCTAAATAATTTTATTGTAACCCATCTCTCCTTATCTATACGCTGTATCTT

GATCTGATTTTTTTTATACTTATAAATTTTGAACATTCCAAATTCTTTAAAAACATTCAACCTACGACGATATACAAACCTTTAAAATAAGTACGATTAA

TCGTGGATCATCAATTATAGGACAGGTTCCTCTGAGTAGACTAAAATACCGCCAAATTCTTAAAATTTCAAGAACATAACTACTACTATTCAAGCATCTA

AAATTTGCATTTTTAATAATAGGGTATCTAATCCTAGTTTTTTATAAAAATCTCATAAACTCATTTTTCACATTTAAAAAATTAATTATACTTACTAATT

TCACCTAATAAATACAATATAAATTAATAATAAAATAACTTATTATATACTGAACAAATTTAATTGCATTGTTTGTGTAACCGCAACTGCTGGCACAAAC

TTGGTCAATACTATTATAAATTCCTAAATCAAAATTTCTTTTAAATTTAATCTTCACTATTGCAATTCTTTAATTAAATATACAGAATAATTTATTTTTT

TAAAATAAATTCATTAAACACTAAAATTTACATATAAAATAATTTAAAAATTAAAATCTCAAGCTAGAATAAAACTTTATTTTTTTTTCAATATACATAA

ATTATATAAAATAAAAGTACCCCCC-TACTATTTTAATTAAATGAATTAATTTTTAATGCCTTAAATTTATTTCCAATTTATTTTTATTATATAGTTTTT

TATTTAAAAATAAAACTTTATACCTAAAATTATATACATAAATTATATTTAAAATTTATAATTTAAAGAACTATAAAAATTAAATTAAAATTTAAAATCT

ATCTAATTTATAGATAAACAAATTAAATTAAACTAACTTACCTGATAAATTTAACTATATACGCGCAGGTATATTAATTATTTAATTATAATATAATACA

TTTGTTTTATTAAATAATTATTAAGCAAAATAGCATTAATTTATTATATAAAAGATCTAGATACTATTAATTTAAAGTAAATTTTTTTTTTTTTTGTAGT

ATAATTTATACTAATATACAGCATTTAATATCTTCTATATAAATGTTTATATTATTTATATTTATCTATTTATAATTCTTAATAATAATTTAGATAATTA

TTAATTTATATATTAAATATTTATATATATATATATAATAATAAGGATATATATATATATATGTATATAGATAAATTAAATTTAGTTATATATAAAAAAA

TTAATATTAATTTTACTTTTAATTTTTTCTTTAATTTAAACTATTGGATATTCAGATTCTAATATATTATAATATATCAACCATTAATGAATAACTTTAT

ATATATAATAAAAATGTTAATTAATATATCAATTCATATGAATATATAATATTAATAAATAAATTCTTAATAAATAATAGTGATAATATATATATATTGT

ATATTAATGTACTATATATATAAATTCATTAAACATTTATATTTGATACCCCCCCAAAATTTTTTCTAAAAATGTGAAGTTTCAATTTAATTAAAATTTT

CTACAATGGTCAAAAACGGCCAAAAAATGGAAAATAAAATGTGCACAGAAAATGCACATAAAATGCACATTTTTGAAAATTTCCGATTTTTGAAAAACCG

TTTGAACCAAACCTCTGATGTACCTTTTTTCGCCCCCCCTGATTACTAAAATTTATCCTCAATTACTAAGGTTTATCCGCATTTTTGCGACAGGAATTTT

GAGTATAAAATCAATTCCACACTAAATTTTTTTTT-TTTTTTTTGTGTCATTTCTCCAACATGGGCAATTTTTTTTTTAGTCGAAGGACAAATTTTAGTA

AAAAAAGTTTGAAATTAGGTTAAACCCTTGTTTAACCAAAAACTATTACTTTTCAATATTATACCCAATTTTATTTAAAATAAATTTTATTATACTTTTA

TTTATGCAAAAAGATCTTCTTATAAACTTTT

>DMR77j_JP_Mori_Honshu

AATGAAGTGTCTGACTATAGAGTTACTTTGATAGAGTAAAAAAAGTGAATTTTCACCTTCATTATAATTAACAGAATTAAACTATTTCTTTAAGCTTCAA

AAACTTATGTACATTATATACTAAATTATAAAAAGATAAGCTAATTAAGCTACTGGGCTCATACCCCATCAATAAAGGTTACAATCCTTTTCTTTTTAAT

GTATTATAAGCTTTTATTTTTTAATTCTCTTATAATTGGAACCTTAATTGCTATTTCTTCCTACTCTTGAATAGGAATATGAATAGGATTAGAAATTAAT

CTTCTCTCTATGATCCCCCTCATTAGAGACAACAAAAATATAATAGCCTCAGAAGCTGCTTTAAAATATTTCATTATTCAAACAATAGCATCAACATTGT

TATTATTCTCAATTATTATAATATCAATGAAATTTATATATCAAATAAATTTAATCACTTACTTTAATTTAATTTTTAACACTTCATTGTTCATCAAAAT

AGGAGCAGCCCCATTCCATTTTTGATTCCCCGAAATAATAGAAGGATTAAATTGATTAAATGCCATTATCATACTTACTTGACAAAAACTAAGGCCCATA

GTATTACTAACGTATTCTAATACAACCTCCATATATCTAATTTTAACAATTATATTTAGAATAATAATCAGAGGAATTATAGGTCTAAATCAAACTAGAT

TACGGAAAATTATAGCTTATTCATCTATCAACCATATTGGATGAATAATCAGTTCAATTATATTAATTGAAATTGTTTGATTTTACTATTTTATTATTTA

TTGCATTATTACTATTAATATCAGAATTATATTTATAAAATTAAATGTTTTTCATATCAATCAATTATATATTTCAATAAATTACCATATTTTACTTAAA

TTATTCTTTGCCTTAAATTTCATGTCTTTAGGAGGATTACCCCCATTTTTAGGGTTTTTCCCTAAATGACTTACAATTCAAACTTTAATCCAAAGAAATA

TATACTCAATTGCTTTCATTATAATTTTAATAACTTTAATAACACTTTTTTTTTACCTCCGAATTACTTTTTCAATTTTACTACTAAGAAAAACAGTTTT

AACATTTTACACACAACCAAAAATTTATACTAATTACATTATAGCATTTAATTTTATTGCATTATTAAGATTAATTTTCGTTACTTTGATATTCAATTTC

TTATAAATTAAACCTGAAGGATTTAAGTTAAATTAAACTAAGAACCTTCAAAGTTCTAAATAAAGTAAATTCTTTAAGCCTTAGGGCTTAGCCCATCTTT

AAATTTGCAATTTAAAATTCTTTTTGAACTATAAAGCTTGATAAAAGAAACTAATTTCGTATGTAAATTTACAGTTTACCGCCTAAACCTCGGCCATTTT

ATCGAATAAATGGCTATTCTCTACAAATCACAAAGATATCGGAACTTTATACTTTCTATTTGGAAGTTGGGCAGGCATAGTAGGCACTTCCTTAAGTTTA

CTGATTCGTGCTGAATTAGGAAACCCAGGATCTCTAATTGGAGATGATCAAATTTATAACGTAATCGTAACAGCTCATGCTTTCATTATAATTTTTTTTA

TAGTAATACCTATTATAATTGGAGGATTCGGAAACTGATTAGTTCCTTTAATGCTTGGAGCCCCCGACATAGCATTTCCCCGAATAAATAACATAAGATT

TTGACTTTTACCTCCTTCATTAACTCTACTTCTAATAAGAAGATTAGTCGAAAGAGGGGCAGGTACAGGCTGAACAGTTTATCCCCCACTATCAGCCAAT

ATTGCCCATAGAGGAGCATCAGTTGATTTAGCAATTTTTAGACTTCATCTTGCAGGAATTAGCTCAATTTTAGGTGCAGTAAATTTTATTACTACTGTAA

TTAACATACGATCAACAGGAATAACTTTTGATCGAATACCCCTATTTGTTTGATCTGTAGTTTTAACAGCACTTCTTCTGCTATTATCCCTCCCAGTTTT

AGCAGGAGCAATTACTATACTATTAACAGATCGAAATATTAATACAACATTTTTTGATCCCGCAGGTGGAGGAGACCCCATTCTCTACCAACATTTATTT

TGATTTTTTGGTCATCCAGAAGTTTACATTTTAATTTTACCTGGATTTGGTATAATCTCCCATATTATTAGCCAAGAAAGAAGAAAAAAAGAAACATTTG

GAACTTTAGGTATAATTTACGCTATAATAGCTATTGGTTTACTAGGATTTATTGTTTGAGCTCATCATATATTTACAGTAGGTATAGATGTAGACACACG

AGCATATTTTACATCAGCAACAATAATCATTGCTGTTCCTACAGGAATTAAAATTTTTAGATGATTAGCTACTCTCCATGGATCACAATTAAACTACTCC

CCGTCTCTTTTATGGGCATTAGGATTTGTATTCCTATTTACAGTAGGAGGATTAACAGGAGTAATTCTAGCTAATTCATCAATTGACATTATTTTACATG

ATACTTACTATGTAGTTGCACATTTCCATTATGTCCTTTCCATAGGAGCTGTATTTGCTATTATAGCAGGTTTTGTTCATTGATTCCCTTTATTTACAGG

TTTAACAATAAATTCAAAATTTCTTAAAATTCAATTTTTAACAATATTTATTGGTGTTAATATAACATTCTTCCCTCAACATTTCTTAGGATTAAGAGGA

ATACCTCGACGTTATTCAGATTACCCAGATGCTTATACAACTTGAAATATTATTTCATCTATTGGATCTTTAGTTTCTTTAATTAGTATTTTTATCTTTT

TATTTACTATTTGAGAAAGGCTAATTTCATTACGAAAAAGAATTAGGTCTTTAAGAATATCTACATCAATTGAATGACTCCAACAAATACCCCCTTCAGA

ACATAGTTATTCTGAACTTCCAATGCTTACTAACTTCTAATATGGCAGATTAGTGCAATGGATTTAAACCCCAAATATAAAGATTAAACTTTTTTTAGAA

ATAGCTACTTGAAATACCATTTTACTTCAGGATAGGGCATCCCCATTAATAGAGCAACTCTCATTCTTTCATAACCATGCTCTTCTAATTCTCTTTATAA

TTACCGTTCTAGTAGGTTATTTAATAGGAACTTTATTTTTTAACCAATTTAATTACCGATTTTTATTAGATGGTCAAACTATTGAAATTATTTGAACTAT

TTTACCTGCTGTAACACTAATTTTTATCGCATTACCGTCTTTACGCTTACTTTATCTTCTAGATGAAGTTAATAACCCTTTAGTAACTATCAAAACAATT

GGGCATCAATGATATTGATCATACGAATATAGAGATTTTATAAATTTTGAATTCGATTCCTATATAATTCCTTTGACAGAAATAAAACCTCAAAATTTTC

GTTTATTAGATGTTGATAACCGAGTAATTGTCCCCTTTAACTCCCAAATCCGAATGATAGTAACAGCTGCCGATGTTATTCATTCATGAACTATCCCGGC

TTTTAGTGTAAAAATTGATGCAACACCAGGCCGACTAAATCAAATTAGATTCCTAATTAATCGAACAGGATTATTTTATGGTCAATGCTCAGAAATTTGT

GGGGCAAATCATAGATTTATACCTATTACTGTAGAAAGAATTTCACCTTCATTTTTTACTAAATGAATCTCAAAAATAAATAACCTATCATTAGATGACT

GAAAGTAAGTAATGGTCTCTTAAACCAATTAATAGTAGTTTAACATCTACTTCTGATGGCCAAAAATTTAGTTAAGATATAACATTAGTTTGTCATACTA

AAATAATCATAATTTGATAATTTTTAATTCCACAAATAGCACCTTTAAACTGACTATCTTTATTTTTTTTAATTATTATTATTTTTTTACTTTTTAATGT

ATTAAATTACTTTAGATTCTTACAGCCCTTAAAAACCCAATCTCATAACCCTACAATTAAAAAAATTAATTGAAAATGATAACTAATTTATTTTCATCTT

TTGATCCTAGAACTTCTTTTAATTTAAGATTAAACTGATTAAGAATACTATTAGGGCTAATATTTATCCCCCCAATATTTTGATTAGTTCCTTCACGCTA

TAATTTTCTATGAATTAAAATTATTTTAACATTACACCAAGAATTTAAGGTTTTAATTGGTAATAATAATATTAAAGGAAGAACCTTAATATTTATTTCA

TTATTTTCTATAATTGTTTTCAATAACTTTTTAGGATTATTTCCGTATATTTTTACAGGAACAAGACATTTAATTATAACATTATCTCTTGCCTTACCTT

TATGAATTAGATTCATATTATACGGGTGAATTAATAACACTACCCACATACTTGCTCATTTAGTCCCCCAAGGAACACCCCCAGCTCTTATGGCATTCAT

AGTAGTAATTGAATCAATTAGAAATATTATTCGTCCTGGTACTTTAGCTGTTCGATTAGCTGCTAATATAATTGCTGGACATTTACTAATAACTTTACTA

GGAAACACAGGAGTAAATTTATCAATTTTTATACTAAGTATTCTTATTATCACACAAATTCTTTTATTAATTTTAGAATCTGCTGTTGCGATCATTCAAT

CTTATGTATTTGCTGTATTAAGAACTTTATACTCTAGAGAAATTAATTAATGTCAAGACATAAAAATCACCCTTATCATTTAGTTGATGCAAGACCTTGA

CCTATTTTAGGTGCTTTTAGAGCTATAATTACAATAATTGGAATTATCAAATGATTCCATTTTTATAATAATTCTTTATTTTACTTAGGGACATTAATCA

CAATTTTAATTATAATTCAATGATGACGAGATATCACTCGTGAGGGAACTTTCCAAGGACTTCATACTTACGCTGTAACTATAGGTTTACGTTGAGGAAT

AATTTTATTTATTACATCAGAAGTATTTTTCTTTATTTCTTTTTTTTGAGCCTTTTTTCATAGTAGCTTAACACCCGCTATTGAACTTGGGATACTCTGA

CCACCCAAAGGAATTACCCCATTTAACCCTATTCAAATTCCATTATTAAACACTTTAATTCTTTTAACTTCAGGATTAACTGTAACTTGAGCTCACCATA

GATTAATAGAAAATGACTATAACCAAACAATGCAAGGTCTTGGTTTAACAGTTTTACTAGGAGTATATTTTACTTTATTACAAGGTTACGAATATTTAGA

AGCCCCCTTTACTATAGCAGATTCTGTTTATGGATCAACATTTTTTATTGCTACTGGTTTCCATGGATTACATGTTATTATTGGCACAACCTTTTTAGCT

GTTTGTTTAATACGGCATTTTAATAACCATTTTACTTGTATCCATCACTTTGGATTTGAAGCTGCTGCTTGATACTGACATTTTGTTGATGTAGTATGAC

TATTTCTTTATATTTCTATTTACTGATGAGGTAGATATTTATATAGTATAATAATTATAATTGATTTCCAATCAAAAGATCTAAAAAAA-TTAGTATAAA

TAATCATTATAATTTGAAATATAGGGTTAATTATTTTTTCTATCTCTTTTATCTTAATTATACTATCTTTTACAATCTCTAAAAAAAGATTTATAGACCG

AGAAAAAGCTTCTCCATTCGAGTGTGGATTTGACCCCAAAAGATCAGCCCGTTTACCCTTTTCTTTGCATTTTTTTTTAATTGCAGTAATTTTCTTAATT

TTTGATGTTGAAATTACTCTTCTTATCCCCTTAATTCTAACAATAAAAATTACTAATATTACTGTATATACCTATATTGCTCTTTTCTTTTTAATGATTC

TTTTAATAGGACTTTACCATGAATGAAACCAAGGGGCCTTAAATTGAGCTCTTTAGGGTAATAGTTAAGTATAACATTTAAGTTGCATTTAAAAAGTATT

GATTTTTCAATTTACCTTAAATAAGAAACAATTAATTGTATTTAGTTTCGACCTAAAATTTAGGTGTATGAACACCCTTATTTAAATTAATTGAAACCAA

AAAGAGGTATATCACTGTTAATGATACTAATGAGAAAAACTCCAATTAAGGAAATAAGATATTCAAGAGTAAGCTTCTAACTTAACTCTTTAGCAGTGAA

AGTCTGTTAATATTTCTATTTATATAGTTTAATAAAACATTATTTTTTCATAATAAAATTAGAATAAATTTATTCTTATAAATATTTAAAAGTAAATTTT

ACTTCCCTGATAACTTCACTATCATACTCTATATAAGCTATTTAAATTAAATATATAAAATTATAAAAATTACCCATATTATAATTAAAAGTATAAAAAT

CTTATAATTGTTATTAAATATAAACTGTAAAAATACAGAAGTATTACTAATTTTACTATATAAATTTTGTCTTCCATAATACTCTGATCAGCCTTGATCA

ATAGTTTTATATAACTTACTACCTAATTTAATTGGATAATAATTTAAACCAAATGTTGAAATATAGGGTATATTCCACATAGAAGAAAGAAAAAGTCTTG

AATTTAACAAATAAAAAGATTTTAATCTATCATTTAAAGTAAACTTAGAAAGTTCAAATCCAAACCAAGCCCCAAAAAATGATACAATTAAAGCTATAAT

TTTTATTGTAAAAGGTAAACAAATAAAATAAGGTGTTGGGAATATCAACCATATTAATATTCTTCCCCCAACAATAACTAAAAAAATTAAACCTGATATC

CCTTGAAGTATAATTTTTCTATTATCATTAATTTTACTTAAAGAATAAAAACAAAAATTTCCTACCAAAACATAATAAATTAAACGAAATGTATAACAAA

CAGTTAATCCTGTTGAAAAAAAGAAAATAACATAAATATAGATATTTAAGTATCTTATAGATAAAACCTCTAAAATTAAATCCTTTGAATAAAACCCCGA

TAAAAAGGGTAAACCACATAATGCTAAATTTGAAATTATAAAATAAGTACAAGTTAAAGGTATAACTTTAATTAACCCCCCTATATACCGAATATCTTGA

CAATTTCTTAATCTGTGAATTATACACCCTGCGCATATAAATAATAAAGCCTTAAATAAAGCGTGAGTTAATAGATGAAAAAAAGCTAATTGATACTCCC

CTAAAGCTAAAATACTAATTATCAAACCCAATTGTCTTAAAGTAGACAGAGCAATAATTTTTTTTAAATCAAACTCAAAGTTTGCGCCTATTCCTGCTAT

AAATATAGTTATTGTTCCAATAAATAATAAAATTAATATTAAATTACTTGTTAATGCAAAATTAAAACGAATTAATAAGTAAACACCTGCTGTTACTAAG

GTAGAAGAATGAACTAAAGAAGAAACAGGTGTTGGAGCCGCTATTGCCGCAGGCAACCAAGAAGAAAATGGAATTTGAGCTCTTTTAGTTATAGCTGCTA

ATATAATTAATAAAATAATAATATATATTTCTATACTATTTTTATATACATCAATATAAAAAATATAGTTAAATCCCCCAAAATTTATTATTCATGCAAT

TGCTATTAATAAAGCAACATCTCCAATACGGTTAGTTAAAGCTGTAATTATCCCTGCATTATAAGATTTAATATTTTGATAATAAATAACTAAACAATAA

GAAACTAACCCTAACCCATCTCATCCTAATAAAATCCTAATTAAATTAGGAGAAATAATCAATAACATTATAGATAAAACAAATATAGAAACTAATATAA

TAAATCGATGCAAATAAATATCTCCTTCTATATACTCTTCACTATAGTAAATTACTATAGAAGAAATAAATAAAACAAAACTCATAAATAATAATGACAT

TCAGTCAAGTAAAATGGTTATAATAATTCTACAAGAATTAATCCTTAATATTTCATATTCTAATATTAGTCTATAATCTAAAATTATGAAATTTAAGCTT

AATAAAAATCTTAATACTCTAAAAAATAAAAACGTTACAAAATAAATTAAACAAATAGAAATAATTTAAAGTAAATTTTACATCTTTGATACCACAAATC

AATATTTTTTATTAAACTATTTAAATTACAATCATAAAACTAAAAATTCTCTCTTTAAAATTAAGATATTCAGGGGTAACCAATGAAGTAATAATAATAA

ATACTCACGAACAAATCCTCTTGAAAAAGAATATAAATTTCTAACTAACTTGCCGTGTTGACTATAAGAATATAAATATAAAGAATACGCAGCTCTAAAA

AAAGATATTAAAGATAAAAAAACCATTGTTCAACTCCTTCATCTAACTAATCTATTAATTAAAATAATCTCACCCAGTAAGTTTAATGAAGGAGGAGCCG

CTATATTACAACAACTAAATAAAAATCATCATATTCTTATTATTGGTATTAAATTGATTAGCCCCTTATTTAAATAAATACTTCGGCTATTTAACCGTTC

ATAGGAAATATTTGCTAAACAAAATAATCCTGAAGAACATAAACCATGGGCAATTATTATAACTAAAGCCCCTCTTATACCCCAATAACTTAAAGTTAAA

ATCCCTCTTAATACTAAACCTATATGCGCTACAGAAGAATAAGCAATTAAAGCTTTAATATCAACTTGACGTAAACATATTAATGAAACAAAAAATCCCC

CTACTATTCTAATTGTAATAAAAATATAGTTTACTTGTAAACCTACAGTTAAAAAAATATTTATTAAACGTATTAATCCATACCCCCCTAATTTTAATAT

AACTCCAGCTAAAATTATAGAGCCAGCAACTGGAGCTTCAACATGGGCTTTAGGGAGCCAAAGATGAACAAAAAATATAGGCATTTTAATAAAAAACACT

ATATTTATACATATAAATAACAAGAATCTTTTTACATCATAACATAAAAAGAAAAAATCTAAAGAATGAAATTTTTCATAATAATAAAAAATCCTAATTA

TTATAGGTAATGAAGCAAATAATGTATAAAATAACAAGTAAACACCTGCTTGCAAACGCTCAGGTTGATACCCCCAACCAATAATTAATAAAAGAGTAGG

AATTAAGCTAAACTCAAAAAACAAATAAAAAATAAATAAATTTAAAGAACTAAATGTTATAACTAAAGATAATAATAAAATAATTATTACTAATAAAAAT

AAATTATAAAAATAGTTTTTTCTATAAATACTTTCAGAAGCTAATAATATTAAAGAACAAATCCACAAACTTAATAAAATTATTATAAAAGATAAAAGGT

CATACCCTATAAAATAAGAAATATTTATATACAAATAATTAAAACTAAATCTTAACCCAAATATAAATGTAATAAAAAAATATATATATTGATTAAATCA

GTATCTCTTTTTAATACAACTTAAAGGTACCAATATTAATATTATAAAAATAAACTTTATCATAATACATTAAAAGTTTGAAAATAATCATTTCCATGTG

TTCGTATTATAGAAACTAATAACGATAAGCCTAAAGCTCCTTCACAAACTCTTATAGTTAAAAATACTATACCAAAATAAAATTCAAAATTAAAATATAT

TAAAAATAGATATAAATTAAAGTATAATCCTAAAATAATATACTCTAACCTTAATAATATTAAAAGTAAATGTTTACGTTTAATACAAAAAGAAACTAAT

CCTGTAAAATATATAATCACTGAAAATAACATACAAAAAATTAACATTAGTTTTAATAATTTAATAAAAATACTGGTCTTGTAAATCAGAAATAAGGATT

TTCTTTTAAAACTTCAGAGAAAGAGTAAACCCCTATCATTAATCTCCAAAATTAATATTTTAAATAAACTATTCTCTGTATAATCTTATTACTAATAACA

TTATCTTTTATTAGTTCAATAACTTTTATATTTTTAAGTCACCCTTTATCTATAGGGCTTATTTTATTAATACAAACTATTATTATAGCTTTAACTATAG

GTTTTTTTAATATTAATTTCTGATATTCTTATATTTTATTTCTTATTATAATTGGAGGAATATTAGTTTTATTTATTTATATAACAAGAGTAGCTTCAAA

TGAAAAATTTTCATTTTCTATTAAAATTACATTAATAATTAGAATTATAACTTTAGGATTTTTATTTAGTATTGCTATAATAGACCCCTACTTTTCAGAC

ATCAACTCAATTTACACAGAAAACTTAGATAATTATAAAGAATATAATATATCATTTAGAAAATATTTAAGTTATCCTAATATTATCATTATATACATAA

TAATTATTTATTTATTAATTACATTAATTGCAGTTGTAAAAATTACTCAAATTGAAAAAGGACCTTTACGTCAAACTAACTAATGAAAACACCATTACGA

AAAGCTTCCCCCCTATTAAAAATTATTAATAATAGAATTATTGATTTACCTACACCATCTAATATTTCTGCTTGATGAAATTTCGGATCCTTATTAGGAC

TCTGTCTTTTTATTCAAATTATCACTGGAATTTTCTTAGCTATACATTTTACAGCCCATATTGATATAGCATTTAATAGAGTAATTCATATTTGTCGAGA

TGTAAATTATGGCTGATTATTACGAACAATTCATGCTAACGGGGCTTCTTTTTTCTTTATTTGTATTTATCTACATATTGGCCGAGGAATATATTATAGA

AGCTATAATTTACATTTAACATGAACTATTGGAGTAATCATCCTTTTTATAGTAATAGCTACAGCATTCTTAGGGTATGTATTACCTTGAGGGCAAATAT

CTTTTTGAGGGGCTACTGTTATTACCAATCTTCTCTCTGCTATTCCTTATCTAGGAAATATGATTGTCCAATGATTATGGGGGGGATTTGCAGTAGATAA

CGCCACTTTAACTCGATTTTTTACATTACATTTTATTCTTCCTTTTATTATTTTAGCTTTAATAATTATTCATTTATTATTTCTTCATCAAACTGGTTCT

AATAACCCTTTAGGATTAAATAGAAATATCGATAAAGTGCCTTTCCATCCATATTTTACTTACAAAGATACTTTTGGATTCATTATAATAACTATATTAT

TAATATTTTTAGTTTTAATTAATCCCTATCTTTTAGGAGACCCAGAAAACTTTACACCCGCTAACCCTTTAGTAACCCCAGTCCATATTCAACCAGAATG

ATACTTTTTATTCGCATACGCTATTTTACGATCAATTCCTAATAAATTAGGTGGAGTAATTGCTTTAGTTATATCAATTGCTATTTTATTAATTATACCT

TTAATTAACAAAAAAAAATTTAGTAGAACTCAATTTTACCCATTAAATAAAATTTTATTTTGATCTTTTGTCTCTATTGTAATCTTATTAACATGAATCG

GAGCTCGCCCAGTGGAAGATCCATACATTTTAACAGGTCAAATTTTAACAATTATATATTTTAGTTATTATTTCTTAAACCCCTTAATTCATAAAATATG

AGATTATATTATTTTTAAAACTTAGTTAATGAACTTGTTAAAGTGTATATTTTGAAAATATAAAAAAGAGTTTATTCTCTATTAACTTTACTAAATTTTA

TTCACTAAATAAAATGAGAAAAGATAACCAACTTTAAACCTAAAAAAAAAAATAAAAAATTTAAAGAAACTGGTAAATAACTTTTTCAACATATATATAT

TAACTTATCATAACGATACCGAGGTAAAGTACCTCGAACTCAAATCCAAATAAATGCTATAAAAGTTAACTTCAAAAAAAAAAATAAAGAAAAAATATCC

CCCCCTATAAATAACAACACACATAATATACTTATAAATAAAATTCTAGCATACTCAGCCAAAAAGATTAAAGCAAATCCCCCTCTTCTATACTCTACAT

TAAACCCAGAAACTAACTCTGATTCTCCTTCTGCAAAATCAAAAGGAGTCCGATTAGTCTCAGCTAAACTTGAAGATAATCATATTATTCTTAAAGGTAA

GCACAGAAAAAAAAATCATACTATTTCTTGATATTTTATAAAATCTATTATATTTAAATTTAAAATTAACAATAAAAAAGATAATAAAATTAATGATAAA

CTTACTTCGTATGAAATTGTTTGAGCTACTGATCGAATACCCCCTAATAAAGCATAATTAGAATTTGATGATCAACCAGAAATTATGATAGTATAGACCC

TTAGCCTAGAACAACATAAAAAATATAAAATACCTAAATTAAACCTAAATATAAATGTCAAAAAAGGTATACATATCCATAATAAAAGAGCTAAAAATAA

ATTAAAAACTGGAGATATATAATAAATAAAAAAATTTGATATTAAAGGATAAGTTTGTTCTTTAGAAAATAACTTAATAGCATCACTAAAAGGTTGAGGA

ATACCTATAAATCCAACTTTATTGGGCCCTTTACGAATTTGAATATACCCTAAAACTTTGCGCTCTATTAATGTTAAAAAAGCTACACCAATTAAAACAC

AAATAATTAAAACTAAACTTGTAAATAATAAAAGAAATAAATCTTGTAATATAATGTATTACTTGTGTTAAACACATATTTAAATTCTAAATTTAAAGCA

CTAATCTGCCAAAGTAATATTCATATTCAAATTATATTAAATTTTAAAGGTATTTGATCCTTTCGTACTAAAATACCTATGTTTTTTAAAGATAGAAACC

AACCTGGCTCACGCCGGTTTAAACTCAGATCATGTAAAATTTTAAAGGTCGAACAGACCTAACCTTTTAGCCCCTACACCAAAAGTTAATTTTAATCCAA

CATCGAGGTCGCAAACTTTTTTTTCGATAAGAACTCTAAAAAAAAATTACGCTGTTATCCCTAAGGTAATTTAATCTTGTAATCATTAAAAATGGATCAT

TCAATCATAAATTAATGTTTTTAAATAAAAAAAGTTTAATCAATTTTTCTGCTGCCCCAGCAAAATAGTTTAAATTATTAAATATATAAATATACTAAAA

TTAAATAATAATTTAAACTATAAAACTCTATAGGGTCTTCTCGTCTTTTAAAATTATATAAGCTTTTTTACTTATAAATAAAATTCTATATTCAATTAAA

TTGAGACAGTTACTTTCTCGTCCAACCGTTCATTCCAGCTTTCAATTAAAAAACTAATGATTATGCTACCTTTGCACGGTCAAATTACCGCGGCCATTCA

AATCCTCATTGGGCAGGTCAGACTTTAAATTATAATCAAAAAGACATGTTTTTAATAAACAGGCGAAAAGTGTATTTGCCGAGTTCCTTAATTTAACCTT

GAAGTTTTAATTTAATTACTAAATTAAAATATATACTAATTTTATCATTATTCTATATAAACCAATATTACATATATTATCTTAATAAACTACTTAAAAA

TAATATAAATCTTATTCTAACAAAAATTATTTATAACAAACTAAAGATTAACACTTCCAATTCTACTAATTTTTATTCAAAATATACATTTTTAACATTT

TATTTTAAAGCTTATCCCCTAAAATATTACTTTTTATATATAAAATACTAAATAATTAATATAATACAATAAAAAAACTAAATTAAATTTATTTCTTAAG

AAACTAGATATCTTAAAAAACGTATAACGTTTCATTTCTAATATAATATTTTAAAAATTTATGCCACAATTAAATTTATATTATATTAGCTCTTTATAAT

TCGAGAACACTAAATAATTAAATTATTTTAATAAACCCTGATACACAAGGTACAAAAAATTAATTTTTCTTTTTAAAAAATAAATCTCTATATATTTATA

TTATCTATCTCTATACAAATTAACTATAATAAAATTTTTATATTCTAAAATATACTAATATCAAAAATATTTTTTTTATATATATATATATA----TTAT

AAATTTTTCCTTTCAAATTAAATTGATTTTCACAACTAACTTTTTAATGTAAATAAAATGCTTTATTACAAGCTCTAATTTGCCATTCCAGGTACACTTT

CCAGTACACCTACTATGTTACGACTTATCCCTCTTTAGAGAGGGAGCGACGGGCGATATGTACATATTCTAGAGCTATACTCATATAATTAAACTAAACT

ATATTACTTTCAAATCCACTTTATAAAATAATGTTAATTATTTTAACCATCTAAATAATTTTATTGTAACCCATCTCTCCTTATCTATACGCTGTATCTT

GATCTGATTTTTTTTATACTTATAAATTTTGAACATTCCAAATTCTTTAAAAACATTCAACCTACGACGATATACAAACCTTTAAAATAAGTACGATTAA

TCGTGGATCATCAATTATAGGACAGGTTCCTCTGAGTAGACTAAAATACCGCCAAATTCTTAAAATTTCAAGAACATAACTACTACTATTCAAGCATCTA

AAATTTGCATTTTTAATAATAGGGTATCTAATCCTAGTTTTTTATAAAAATCTCATAAACTCATTTTTCACATTTAAAAAATTAATTATACTTACTAATT

TCACCTAATAAATACAATATAAATTAATAATAAAATAACTTATTATATACTGAACAAATTTAATTGCATTGTTTGTGTAACCGCAACTGCTGGCACAAAC

TTGGTCAATACTATTATAAATTCCTAAATCAAAATTTCTTTTAAATTTAATCTTCACTATTGCAATTCTTTAATTAAATATACAGAATAATTTATTTTTT

TAAAATAAATTCATTAAACACTAAAATTTACATATAAAATAATTTAAAAATTAAAATCTCAAGCTAGAATAAAACTTTATTTTTTTTTCAATATACATAA

ATTATATAAAATAAAAGTACCCCCC-TACTATTTTAATTAAGTGAATTAATTTTTAATGCCTTAAATTTATTCCCAATTTATTTTTATTATATAGTTTTT

TATTTAAAAATAAAACTTTATACCTAAAATTATATACATAAATTATATTTAAAATTTATAATTTAAAGAACTATAAAAATTAAATTAAAATTTAAAATCT

ATCTAATTTATAGATAAACAAATTAAATTAAACTAACTTACCTGATAAATTTAACTATATACGCGCAGGTATATTAATTATTTAATTATAATATAATACA

TTTGTTTTATTAAATAATTATTAAGCAAAATAGCATTAATTTATTATATAAAAGATCTAGATACTATTAATTTAAAGTAAATTTTTTTTTTTTTTGTAGT

ATAATTTATACTAATATACAGCATTTAATATCTTCTATATAAATGTTTATATTATTTATATTTATCTATTTATAATTCTTAATAATAATTTAGATAATTA

TTAATTTATATATTAAATATTTATATATATATATATAATAATAAGGATATATATATATATATGTATATAGATAAATTAAATTTAGTTATATATAAAAAAA

TTAATATTAATTTTACTTTTAATTTTTTCTTTAATTTAAACTATTGGATATTCAGATTCTAATATATTATAATATATCAACCATTAATGAATAACTTTAT

ATATATAATAAAAATGTTAATTAATATATCAATTCATATGAATATATAATATTAATAAATAAATTCTTAATAAATAATAATGATAATATATATATATTGT

ATATTAATGTACTATATATATAAATTCATTAAACATTTATATTTGATACCCCCCCAAAATTTTTTCTAAAAATGTGAAGTTTCAATTTAATTAAAATTTT

CTACAATGGTCAAAAACGGCCAAAAAATGGAAAATAAAATGTGCACAGAAAATGCACATAAAATGCACATTTTTGAAAATTTCCGATTTTTGAAAAATCG

TTTGAACCAAACCTCTGATGTACCTTTTTTCGCCCCCCCTGATTACTAAAATTTATCCTCAATTACTAAGGTTTATCCGCATTTTTGCGACAGGAATTTT

GAGTATAAAATCAATTCCACACTAAATTTTTTTTT-TTTTTTTTGTGTCATTTCTCCAACATGGGCATTTTTTTTTTTAGTCGAAGGACAAATTTTAGTA

AAAAAAGTTTGAAATTAGGTTAAACCCTTGTTTAACCAAAAACTATTACTTTTCAATATTATACCCAATTTTATTTAAAATAAATTTTATTATACTTTTA

TTTATGCAAAAAGATCTTCTTATAAACTTTT

>DMR148u_USA_Littleton_CO

AATGAAGTGTCTGACTATAGAGTTACTTTGATAGGGTAAAAAAAGTGAATTTTCACCTTCATTATAATTAACAGAATTAAACTATTTCTTTAAGCTTCAA

AAACTTATGTACATTATATACTAAATTATAAAAAGATAAGCTAATTAAGCTACTGGGCTCATACCCCATCAATAAAGGTTACAATCCTTTTCTTTTTAAT

GTATTATAAGCTTTTATTTTTTAATTCTCTTATAATTGGAACCTTAATTGCTATTTCTTCCTACTCTTGGATAGGAATATGAATAGGATTAGAAATTAAT

CTTCTCTCTATGATCCCCCTCATTAGAGACAACAAAAATATAATAGCCTCAGAAGCTGCTTTAAAATATTTCATTATTCAAACAATAGCATCAACATTGT

TATTATTCTCAATTATTATAATATCAATGAAATTTATATATCAAATAAATTTAATCACTTACTTTAATTTAATTTTTAACACTTCATTGTTCATCAAAAT

AGGAGCAGCCCCATTCCATTTTTGATTCCCCGAAATAATAGAAGGATTAAATTGATTAAATGCCATTATCATACTTACTTGACAAAAACTAAGGCCCATA

GTATTACTAACGTATTCTAATACAACCTCCATATATCTAATTTTAACAATTATATTTAGAATAATAATCAGAGGAATTATAGGCCTAAATCAAACTAGAT

TACGGAAAATTATAGCTTATTCATCTATCAACCATATTGGATGAATAATCAGTTCAATTATATTAATTGAAATTGTTTGATTTTACTATTTTATTATTTA

TTGCATTATTACTATTAATATCAGAATTATATTTATAAAATTAAATGTTTTTCATATCAATCAATTATATATTTCAATAAATTACCATATTTTACTTAAA

TTATTCTTTGCCTTAAATTTCATATCTTTAGGAGGATTACCCCCATTTTTAGGATTTTTCCCTAAATGACTTACAATTCAAACTTTAATCCAAAGAAATA

TGTACTCAATTGCTTTCATTATAATTCTAATAACTTTAATAACACTTTTTTTTTACCTTCGAATTACTTTTTCAATTTTACTACTAAGAAAATCAGTTTT

AACATTTTACACACAACCAAAAATTGATACTAATTACATTATAGCATTTAATTTTATTACATTATTAAGATTAATTTTCGTTACTTTGATATTCAATTTC

TTATAAATTAAACCTGAAGGATTTAAGTTAAATTAAACTAAGAACCTTCAAAGTTCTAAATAAAGTAAATTCTTTAAGCCTTAGGGCTTAGCCCATCTTT

AAATTTGCAATTTAAAATTCTTTTTGAACTATAAAGCTTGATAAAAGAAACTAATTTCGTATGTAAATTTACAGTTTACCGCCTAAACCTCGGCCATTTT

ATCGAATAAATGGCTATTCTCTACAAATCACAAAGATATCGGAACTTTATACTTTCTATTTGGAAGTTGAGCAGGAATAGTAGGTACTTCCTTAAGTTTA

CTGATTCGTGCTGAATTGGGAAACCCAGGATCTCTAATTGGAGATGATCAAATTTATAACGTAATCGTAACAGCTCATGCTTTCATTATAATTTTTTTTA

TAGTAATACCTATTATAATTGGAGGATTCGGAAACTGATTAGTTCCTTTAATGCTTGGAGCCCCCGACATAGCATTTCCCCGAATAAATAACATAAGATT

TTGACTTTTACCTCCTTCATTAACTCTACTTCTAATAAGAAGATTAGTCGAAAGAGGAGCAGGTACAGGCTGAACAGTTTATCCCCCACTATCAGCCAAT

ATCGCCCATAGAGGAGCATCAGTTGATTTAGCAATTTTTAGACTTCATCTTGCAGGAATTAGCTCAATTTTAGGTGCAGTAAATTTTATTACTACTGTAA

TTAACATACGATCAACAGGAATAACTTTTGATCGAATACCCCTATTTGTTTGATCTGTAGTTTTAACAGCACTTCTTCTGCTATTATCCCTCCCAGTTTT

AGCAGGAGCAATTACTATACTATTAACAGATCGAAATATTAATACAACATTTTTTGATCCCGCAGGTGGAGGAGACCCCATTCTCTACCAACATTTATTT

TGATTTTTTGGTCATCCAGAAGTTTACATTTTAATTTTACCTGGATTTGGTATAATCTCCCATATTATTAGCCAAGAAAGAAGAAAAAAAGAAACATTTG

GAACTTTAGGTATAATTTACGCTATAATAGCTATTGGTTTACTAGGATTTATTGTTTGAGCTCATCATATATTTACAGTAGGTATAGATGTAGACACACG

AGCATATTTTACATCAGCAACAATAATCATTGCTGTTCCTACAGGAATTAAAATTTTTAGATGATTAGCTACTCTCCATGGATCACAATTAAACTACTCC

CCGTCTCTTTTATGGGCATTAGGATTTGTATTCCTATTTACAGTAGGAGGATTAACAGGAGTAATTCTAGCTAATTCATCAATTGACATTATTTTACATG

ATACTTACTATGTAGTTGCACATTTCCATTATGTCCTTTCCATAGGAGCTGTATTTGCTATTATAGCAGGTTTTGTTCATTGATTCCCTTTATTTACAGG

TTTAACAATAAATTCAAAATTTCTTAAAATTCAATTTTTAACAATATTTATTGGTGTCAATATAACATTCTTCCCTCAACATTTCTTAGGATTAAGAGGA

ATACCTCGACGTTATTCAGATTACCCAGATGCTTACACAACTTGAAATATTATCTCATCTATTGGATCTTTAGTTTCTTTAATTAGTATTTTTATCTTTT

TATTTACTATTTGAGAAAGGCTAATTTCATTACGAAAAAGAATTAGGTCTTTAAGAATATCTACATCAATTGAATGACTCCAACAAATACCCCCTTCAGA

ACATAGTTATTCTGAACTTCCAATGCTTACTAACTTCTAATATGGCAGATTAGTGCAATGGATTTAAACCCCAAATATAAAGATTAAACTTTTTTTAGAA

ATAGCTACTTGAAACACCATTTTACTTCAGGATAGGGCATCCCCATTAATAGAACAACTCTCATTCTTTCATAACCATGCTCTTCTAATTCTCTTTATAA

TTACCGTTCTAGTAGGTTATTTAATAGGAACTTTATTTTTTAACCAATTTAATTACCGATTTTTATTAGATGGTCAAACTATTGAAATTATTTGAACTAT

TTTACCTGCTGTAACACTAATTTTTATCGCATTACCGTCTTTACGCTTACTTTATCTTCTAGATGAAGTTAATAATCCTTTAGTAACTATCAAAACAATT

GGGCATCAATGATATTGATCATACGAATATAGAGATTTTATAAATTTTGAATTCGATTCCTATATAATTCCTTTGACAGAAATAAAACCTCAAAATTTTC

GTTTATTAGATGTTGATAACCGAGTAATTGTCCCCTTTAACTCCCAAATTCGAATGATAGTAACAGCTGCCGATGTTATTCATTCATGAACTATCCCGGC

TTTTAGTGTAAAAATTGATGCAACACCGGGCCGACTAAATCAAATTAGATTTCTAATTAATCGAACAGGATTATTTTATGGTCAATGCTCAGAAATTTGT

GGAGCAAATCATAGATTTATACCTATTACTGTAGAAAGAATTTCACCTTCATTTTTTACTAAATGAATCTCAAAAATAAATAACCTATCATTAGATGACT

GAAAGTAAGTAATGGTCTCTTAAACCAATTAATAGTAGTTTAACATCTACTTCTGATGGCCAAAAATTTAGTTAAGATATAACATTAGTTTGTCATACTA

AAATAATCATAATTTGATAATTTTTAATTCCACAAATAGCACCTTTAAACTGACTATCTTTATTTTTTTTAATTATTATTATTTTTTTACTTTTTAATGT

ATTAAATTACTTTAGATTCTTACAGCCCTTAAAAACCCAATCTCATAGCCCTACAATTAAAAAAATTAATTGAAAATGATAACTAATTTATTTTCATCTT

TTGATCCTAGAACTTCTTTTAATTTAAGATTAAACTGATTAAGAATACTATTAGGGCTAATATTTATCCCCCCAATATTTTGATTAGTTCCTTCACGCCA

TAATTTTCTATGAATTAAAATTATTTTAACATTACACCAAGAATTTAAGGTTTTAATTGGTAATAATAATATTAAAGGAAGAACCTTAATATTTATTTCA

TTATTTTCTATAATTGTTTTCAATAACTTTTTAGGATTATTTCCGTATATTTTTACAGGAACAAGACATTTAATTATAACATTATCTCTTGCCTTACCTT

TATGAATTAGATTCATATTATACGGGTGAATTAATAACACTATCCACATACTTGCTCATTTAGTTCCCCAAGGAACACCCCCAGCTCTTATGGCATTCAT

AGTAGTAATTGAATCAATTAGAAATATTATTCGTCCTGGTACTTTAGCTGTTCGATTAGCTGCTAATATAATTGCTGGACATTTACTAATAACTTTACTA

GGAAACACAGGATTAAATTTATCAATTTTTATACTAAGTATTCTTATTATCACACAAATTCTTTTATTAATTTTAGAATCTGCTGTTGCGATCATTCAAT

CTTATGTATTTGCTGTATTAAGAACTTTATACTCTAGAGAAATTAATTAATGTCAAGACATAAAAATCACCCTTATCATTTAGTTGATGCAAGACCTTGA

CCTATTTTAGGTGCTTTTAGAGCTATAATTACAATAATTGGAATTATCAAATGATTCCATTTTTATAATAATTCTTTATTTTACTTAGGGACATTAATCA

CAATTTTAATTATAATTCAATGATGACGAGATATCACTCGTGAGGGAACTTTCCAAGGACTTCATACTTACGCTGTAACTATAGGTTTACGTTGAGGAAT

AATTTTATTTATTACATCAGAAGTATTTTTCTTTATTTCTTTTTTTTGAGCCTTTTTTCATAGTAGCTTAACACCCGCTATTGAACTAGGGATACTCTGA

CCACCCAAAGGAATTACCCCATTTAACCCAATTCAAATTCCATTATTAAACACTTTAATTCTTTTAACTTCAGGATTAACTGTAACTTGAGCTCATCATA

GATTAATAGAAAATGACTATAACCAAACAATGCAAGGTCTTGGTTTAACAGTTTTACTAGGAGTATATTTTACTTTATTACAAGGTTACGAATATTTAGA

AGCCCCCTTTACTATAGCAGATTCTGTTTATGGATCAACATTTTTTATTGCTACTGGTTTCCATGGATTACATGTTATTATTGGCACAACCTTTTTAGCT

GTTTGTTTAATACGACATTTTAATAACCATTTTACTTGTATCCATCACTTTGGATTTGAAGCTGCTGCTTGATACTGACATTTTGTTGATGTAGTATGAC

TATTTCTTTATATTTCTATTTACTGATGAGGTAGATATTTATATAGTATAATAATTATAATTGATTTCCAATCAAAAGATCTAAAAAAAATTAGTATAAA

TAATCATTATAATTTGAAATATAGGGTTAATTATTTTTTCTATCTCTTTTATCTTAATTATACTATCTTTTACAATCTCTAAAAAAAGATTTATAGACCG

AGAAAAAGCTTCTCCATTCGAGTGCGGATTTGACCCCAAAAGATCAGCCCGTTTACCCTTTTCTTTGCATTTTTTTTTAATTGCAGTAATTTTCTTAATT

TTTGATGTTGAAATTACTCTTCTTATCCCTTTAATTCTAACAATAAAAATTACTAATATTACTATATATACCTATATTGCTCTTTTCTTTTTAATGATTC

TTTTAATAGGACTTTACCATGAATGAAACCAGGGGGCCTTAAATTGAGCTCTTTAGGGTAATAGTTAAGTATAACATTTAAGTTGCATTTAAAAAGTATT

GATTTTTCAATTTACCTTAAATAAGAAACAATTAATTGTATTTAGTTTCGACCTAAAATTTAGGTGTATGAACACCCTTATTTAAATTAATTGAAACCAA

AAAGAGGTATATCACTGTTAATGATACTAATGAGAAAAACTCCAATTAAGGAAATAAGATACTCAAGAGTAAGCTTCTAACTTAACTCTTTAGCAGTGAA

AGTCTGTTAATATTTCTATTTATATAGTTTAATAAAACATTATTTTTTCATAATAAAATTAGAATAAATTTATTCTTATAAATATTTAAAAGTAAATTTT

ACTTCCCTGATAACTTCACTATCATACTCTATATAAGCTATTTAAATTAAATATATAAAATTATAAAAATTACCCATATTATAATTAAAAGTATAAAAAT

CTTATAATTGTTATTAAATATAAACTGTAAAAATACAGAAGTATTACTAATTTTACTATATAAATTTTGTCTTCCATAATACTCTGATCAGCCTTGATCA

ATAGTTTTATATAACTTACTACCTAATTTAATTGGATAATAATTTAAACCAAATGTTGAAATATAGGGTATATTCCACATAGAAGAAAAAAAAAGTCTTG

AATTTAACAAATAAAAAGATTTTAATCTATCATTTAAAGTAAACTTAGAAAGTTCAAACCCAAACCAAGCCCCAAAAAATGATACAATTAAAGCTATAAT

TTTTATTGTAAAAGGTAAACAAATAAAATAAGGTGTTGGGAATATCAACCATATTAATATTCTTCCCCCAACAATAACTAAAAAAATTAAACCTGATATC

CCTTGAAGTATAATTTTTCTATTATCATTAATTTTACTTAAAGAATAAAAACAAAAATTTCCTACCAAAACATAATAAATTAAACGAAATGTATAACAAA

CAGTTAATCCTGTTGAAAAAAAGAAAATAACATAAATATAGATATTTAAGTATCTTATAGATAAAACCTCTAAAATTAAATCCTTTGAATAAAACCCCGA

TAAAAAAGGTAAACCGCATAATGCTAAATTTGAAATTATAAAATAAGTACAAGTTAAAGGTATAACTTTAATTAACCCTCCTATATACCGAATATCTTGA

CAATTTCTTAATCTGTGAATTATACACCCTGCGCATATAAATAATAAAGCCTTAAATAAAGCGTGAGTTAATAGGTGAAAAAAAGCTAATTGATACTCCC

CTAAAGCTAAAATACTAATTATCAAACCCAATTGTCTTAAAGTAGACAGAGCAATAATTTTTTTTAAATCAAACTCGAAGTTTGCACCTATTCCTGCTAT

AAATATAGTTATTGTTCCAATAAATAATAAAATTAGTATTAAATTACTTGTTAATGCAAAATTAAAACGAATTAATAAGTAAACACCTGCTGTTACTAAG

GTAGAAGAATGAACTAAAGAAGAAACAGGTGTTGGAGCTGCTATTGCCGCAGGCAACCAAGAAGAAAATGGAATTTGAGCTCTTTTAGTTATAGCTGCTA

ATATAATTAATAAAATAATAATATATATTTCTATACTATTTTTATATACATCAATATAAAAAATATAGTTAAATCCCCCAAAATTTATTATTCATGCAAT

TGCTATTAATAAAGCAACATCTCCAATACGGTTAGTTAAAGCTGTAATTATCCCTGCATTATAAGATTTAATATTTTGATAATAAATAACTAAACAATAA

GAAACTAACCCTAACCCATCTCATCCTAATAAAATCCTAATTAAATTAGGAGAAATAATCAATAACATTATAGATAAAACAAATATAGAAACTAATATAA

TAAATCGATGCAAATAAATATCTCCTTCTATATACTCTTCACTATAGTAAATTACTATAGAAGAAATAAATAAAACAAAACTCATAAATAATAATGACAT

CCAGTCAAGTAAAATGGTTATAATAATTCTACAAGAATTAATCCTTAATATTTCATATTCTAATATTAGCCTATAATCTAAAATTATGAAATTTAAGCTT

AATAAAAATCTTAATACTCTAAAAAATAAAAACGTTACAAAATAAATTAAACAAATAGAAATAATTTAAAGTAAATTTTACATCTTTGATACCACAAATC

AATATTTTTTATTAAACTATTTAAATTACAATCATAAAACTAAAAATTCTCTCTTTAAAATTAAGATATTCAAGGGTAACCAATGAAGTAATAATAATAA

ATACTCACGAACAAACCCTCTTGAAAAAGAATATAAATTTCTAACTAACTTGCCGTGTTGACTATAAGAATATAAATATAAAGAATACGCAGCTCTAAAA

AAAGATATTAAAGATAAAAAAACCATTGTTCAACTCCTTCATCTAACTAATCTATTAATTAAAATAATCTCACCCAGTAAGTTTAATGAAGGAGGAGCCG

CTATATTACAACAACTAAATAAAAATCATCATATTCTTATTATTGGTATTAAATTGATTAGCCCCTTATTTAAATAAATACTTCGGCTATTTAACCGTTC

ATAAGAAATATTTGCTAAACAAAATAATCCTGAAGAACATAAACCATGGGCAATTATTATAACTAAAGCCCCTCTTATACCCCAATAACTTAAAGTTAAA

ATCCCTCTTAATACTAAACCTATATGCGCTACAGAAGAATAAGCAATTAAAGCTTTAATATCAACTTGACGTAAACATATTAATGAAACAAAAAATCCCC

CTACTATTCTAATTGTAATAAAAATATAGTTTACTTGTAAACCTACAGTTAAAAAAATATTTATTAAACGTATTAATCCATACCCCCCTAATTTTAATAT

AACTCCAGCTAAAATTATAGAGCCAGCAACTGGAGCTTCAACATGGGCTTTAGGGAGCCAAAGATGAACAAAAAATATAGGCATTTTAATAAAAAACACT

ATATTTATACATATAAATAACAAGAATCTTTTTACATCATAACATAAAAAGAAAAAATCTAAAGAATGAAATTTTTCATAATAATAAAAAATCCTAATTA

TTATAGGTAATGAAGCAAATAATGTATAAAATAACAAGTAAACACCTGCTTGCAAACGCTCAGGTTGATACCCCCAACCAATAATTAATAAAAGAGTGGG

AATTAAGCTAAACTCAAAAAACAAATAAAAAATAAATAAATTTAAAGAACTAAATGTTATAACTAAAGATAATAATAAAATAATTATTACTAATAAAAAT

AAATTATAAAAATAGTTTTTTCTATAAATACTTTCAGAAGCTAATAATATTAAAGAACAAATCCACAAACTTAATAAAATTATTATAAAAGATAAAAGGT

CATACCCTATAAAATAAGAAATATTTATATACAAATAATTAAAACTAAATCTTAACCCAAATATAAATGTAATAAAAAAATATATATATTGATTAAATCA

GTATCTCTTTTTAATACAACTTAAAGGTACCAATATTAATATTATAAAAATAAACTTTATCATAATACATTAAAAGTTTGAAAATAATCATTTCCATGTG

TTCGTATTATAGAAACTAATAACGATAAACCTAAAGCTCCTTCACAAACTCTTATAGTTAAAAATACTATACCAAAATAAAATTCAAAATTAAAATATAT

TAAAAATAGATATAAATTAAAGTATAATCCTAAAATAATATACTCTAACCTTAATAATATTAAAAGTAAATGTTTACGTTTAATACAAAAAGAAACTAAT

CCTGTAAAATATATAATCACTGAAAATAACATACAAAAAATTAACATTAGTTTTAATAATTTAATAAAAATACTGGTCTTGTAAATCAGAAATAAGGATT

TTCTTTTAAAACTTCAGAGAAAGAGTAAACCTCTATCATTAATCTCCAAAATTAATATTTTAAATAAACTATTCTCTGTATAATCTTATTACTAATAACA

TTATCTTTTATTAGTTCAATAACTTTTATATTTTTAAGTCACCCTTTATCTATAGGGCTTATTTTATTAATACAAACTATTATTATAGCTTTAACTATAG

GTTTTTTTAATATTAATTTCTGATATTCTTATATTTTATTCCTTATTATAATTGGGGGAATATTAGTTTTATTTATTTATATAACAAGAGTAGCTTCAAA

TGAAAAATTTTCATTTTCTATTAAAATTACATTAATAATTAGAATTATAACTTTAGGATTTTTATTCAGTATTGCTATAATAGACCCTTACTTTTCAGAC

ATCAACTCAATTTACACAGAAAACTTAGATAATTATAAAGAATATAATATATCATTTAGAAAATATTTAAGTTATCCTAATATTATCATTATATACATAA

TAATTATTTATTTATTAATTACATTAATTGCAGTTGTAAAAATTACTCAAATTGAAAAAGGACCTTTACGTCAAACTAACTAATGAAAACACCATTACGA

AAAGCTTCCCCCCTATTAAAAATTATTAATAATAGAATTATTGATTTACCTACACCATCTAATATTTCTGCTTGATGAAATTTCGGATCCTTATTAGGAC

TCTGTCTTTTTATTCAAATTATCACTGGAATTTTCTTAGCTATACATTTTACAGCCCATATTGACATAGCATTTAATAGAGTAATTCATATTTGTCGAGA

TGTAAATTATGGCTGATTATTACGAACAATTCATGCTAACGGGGCTTCTTTTTTCTTTATTTGTATTTATCTACATATTGGCCGAGGAATATATTATAGA

AGCTATAATTTACATTTAACATGAACTATTGGAGTAATCATCCTTTTTATAGTAATAGCTACAGCATTCTTAGGGTATGTATTACCTTGAGGGCAAATAT

CTTTTTGAGGGGCTACTGTTATTACCAATCTTCTCTCTGCTATTCCTTATCTAGGGAATATGATTGTCCAATGATTATGGGGGGGATTTGCAGTAGATAA

CGCCACTTTAACTCGATTTTTTACATTACATTTTATTCTTCCTTTTATTATTTTAGCTTTAATAATTATTCATTTATTATTTCTTCATCAAACTGGTTCC

AATAACCCTTTAGGATTAAATAGAAATATCGATAAAGTACCTTTCCATCCATATTTTACTTACAAAGATACTTTTGGATTCATTATAATAACTATATTAT

TAATATTTCTAGTTTTAATTAATCCCTATCTTTTAGGAGACCCAGAAAACTTTACACCCGCTAATCCTTTAGTAACCCCAGTCCATATCCAACCAGAATG

ATACTTTTTATTCGCATACGCTATTTTACGATCAATTCCTAATAAATTAGGTGGAGTAATTGCTTTAGTTATATCAATCGCTATTTTATTAATTATACCT

TTAATTAACAAAAAAAAATTTAGTAGAACTCAATTTTACCCATTAAATAAAATTTTATTTTGATCTTTTGTCTCTATTGTAATCTTATTAACATGAATCG

GAGCTCGCCCAGTGGAAGATCCATACATTTTAACAGGTCAAATTTTAACAATTATATATTTTAGTTATTATTTCTTAAACCCCTTAATTCATAAAATATG

AGATTATATTATTTTTAAAACTTAGTTAATGAACTTGTTAAAGTGTATATTTTGAAAATATAAAAAAGAGTTTATTCTCTATTAACTTTACTAAATTTTA

TTCACTAAATAAAATAAGAAAAGATAACCAACTTTAAACCTAAAAAAAAAAATAAAAAATTTAAAGAAACTGGTAAATAACTTTTTCAACATATATATAT

TAACTTATCATAACGGTACCGAGGTAAAGTACCTCGAACTCAAATCCAAATAAATGCTATAAAAGTTAACTTCAAAAAAAAAAATAAAGAAAAAATATCC

CCCCCTATAAATAACAACACACATAATATACTTATAAATAAAATTCTAGCATACTCAGCCAAAAAGATTAAAGCAAATCCCCCTCTTCTGTACTCTACAT

TAAACCCAGAAACTAACTCTGATTCTCCTTCTGCAAAATCAAAAGGAGTCCGATTAGTCTCAGCTAAACTTGAAGATAATCATATTATTCTTAAAGGTAA

GCACAGAAAAAAAAATCATACTATTTCTTGATATTTTATAAAATCTATTATATTTAAATTTAAAATTAACAATAAAAAAGATAATAAAATTAATGATAAA

CTTACTTCGTATGAAATTGTTTGAGCTACTGATCGAATACCCCCTAATAAAGCATAATTAGAATTTGACGATCAACCAGAAATTATGATAGTATAGACCC

TTAACCTAGAACAACATAAAAAATATAAAATACCTAAATTAAACCTAAATATAAATGTCAAAAAAGGTATACATATCCATAATAAAAGAGCTAAAAATAA

ATTAAAAACTGGAGATATATAATAAATAAAAAAATTTGATATTAAAGGATAAGTTTGTTCTTTAGAAAATAACTTAATAGCATCACTAAAAGGTTGAGGA

ATACCTATAAATCCAACTTTATTGGGCCCTTTACGAATTTGGATATACCCTAAAACTTTGCGCTCTATTAATGTTAAAAAAGCTACACCAATTAAAACAC

AAATAATTAAAACTAAACTTGTAAATAATAAAAGAAATAAATCTTGTAATATAATGTATTACTTGTGTTAAACACATATTTAAATTCTAAATTTAAAGCA

CTAATCTGCCAAAGTAATATTCATATTCAAATTATATTAAATTTTAAAGGTATCTGATCCTTTCGTACTAAAATACCTATGTTTTTTAAAGATAGAAACC

AACCTGGCTCACGCCGGTTTAAACTCAGATCATGTAAAATTTTAAAGGTCGAACAGACCTAACCTTTTAGCCCCTACACCAAAAGTTAATTTTAATCCAA

CATCGAGGTCGCAAACTTTTTTTTCGATAAGAACTCTAAAAAAAAATTACGCTGTTATCCCTAAGGTAATTTAATCTTGTAATCATTAAAAATGGATCAT

TCAATCATAAATTAATGTTTTTAAATAAAAAAAGTTTAATCAATTTTTCTGCTGCCCCAGCAAAATAGTTTAAATTATTAAATATATAAATATACTAAAA

TTAAATAATAATTTAAACTATAAAACTCTATAGGGTCTTCTCGTCTTTTAAAATTATATAAGCTTTTTTACTTATAAATAAAATTCTATATTCAATTAAA

TTGAGACAGTTACTTTCTCGTCCAACCGTTCATTCCAGCTTTCAATTAAAAAACTAATGATTATGCTACCTTTGCACGGTCAAATTACCGCGGCCATTCA

AATCCTCATTGGGCAGGTCAGACTTTAAATTATAATCAAAAAGACATGTTTTTAATAAACAGGCGAAAAGTGTATTTGCCGAGTTCCTTAATTTAACCTT

GAAGTTTTAATTTAATTACTAAATTAAAATATATACTAATTTTATCATTATTCTATATAAACCAATATTACATATATTATCTTAATAAACTACTTAAAAA

TAATATAAATCTTATTCTAACAAAAATTATTTATAACAAACTAAAGATTAACACTTCCAATTCTACTAATTTTTATTCAAAATATACATTTTTAACATTT

TATTTTAAAGCTTATCCCCTAAAATATTACTTTTTATATATAAAATACTAAATAATTAATATAATACAATAAAAAAACTAAATTAAATTTATTTCTTAAG

AAACTAGATATCTTAAAAAACGTATAACGTTTCATTTCTAATATAATATTTTAAAAATTTATGCCACAATTAAATTTATATTATATTAGCTCTTTATAAT

TCGAGAACACTAAATAATTAAATTATTTTAATAAACCCTGATACACAAGGTACAAAAAATTAATTTTTCTTTTTAAAAAATAAATCTCTATATATTTATA

TTATCTATCTCTATACAAATTAACTATAATAAAATTTTTATATTCTAAAATATACTAATATCAAAAATATTTTTTTTATATATATATATA------TTAT

AAATTTTTCCTTTCAAATTAAATTGATTTTCACAACTAACTTTTTAATGTAAATAAAATGCTTTATTACAAGCTCTAATTTGCCATTCCAGGTACACTTT

CCAGTACACCTACTATGTTACGACTTATCCCTCTTTAGAGAGGGAGCGACGGGCGATATGTACATATTCTAGAGCTATACTCATATAATTAAACTAAACT

ATATTACTTTCAAATCCACTTTATAAAATAATGTTAATTATTTTAACCATCTAAATAATTTTATTGTAACCCATCTCTCCTTATCTATACGCTGTATCTT

GATCTGATTTTTTTTATACTTATAAATTTTGAACATTCCAAATTCTTTAAAAACATTCAACCTACGACGATATACAAACCTTTAAAATAAGTACGATTAA

TCGTGGACCATCAATTATAGGACAGGTTCCTCTGAGTAGACTAAAATACCGCCAAATTCTTAAAATTTCAAGAACATAACTACTACTATTCAAGCATCTA

AAATTTGCATTTTTAATAATAGGGTATCTAATCCTAGTTTTTTATAAAAATCTCATAAACTCATTTTTCACATTTAAAAAATTAATTATACTTACTAATT

TCACCTAATAAATACAATATAAATTAATAATAAAATAACTTATTATATACTGAACAAATTTAATTGTATTGTTTGTGTAACCGCAACTGCTGGCACAAAC

TTGGTCAATACTATTATAAATTCCTAAATCAAAATTTCTTTTAAATTTAATCTTCACTATTGCAATTCTTTAATTAAATATATAGAATAATTTATCTTTT

TAAAATAAATTCATTAAACACTAAAATTTACATATAAAATAATTTAAAAATTAAAATCTCAAGCTAGAATAAAACTTTATTTTTTTTTCAATATACATAA

ATTATATAAAATAAAAGTACCCCCC-TACTATTTTAATTAAATGAATTAATTTTTAATGCCTTAAATTTATTTCCAATTTATTTTTATTATATAGTTTTT

TATTTAAAAATAAAACTTTATACCTAAAATTATATACATAAATTATATTTAAAATTTATAATTTAAAGAACTATAAAAATTAAATTAAAATTTAAAATCT

ATCTAATTTATAGATAAACAAATTAAATTAAACTAACTTACCTGATAAATTTAACTATATACGCGCAGGTATATTAATTATTTAATTATAATATAATACA

TTTGTTTTATTAAATAATTATTAAGCAAAATAGCATTAATTTATTATATAAAAGATCTAGATACTATTAATTTAAAGTAAATTTTTTTTTTTTTTGTAGT

ATAATTTATACTAATATACGGCATTTAATATCTTCTATATAAATGTTTATATTATTTATATTTATCTATTTATAATTCTTAATAATAATTTAGATAATTA

TTAATTTATATATTAAATATTTATATATATATATATAATAATAAGGATATATATATATATATGTATATAGATAAATTAAATTTAGTTATATATAAAAAAA

TTAATATTAATTTTACTTTTAATTTTTTCTTTAATTTAAACTATTGGATATTCAGATTCTAATATATTATAATATATCAACCATTAATGAATAACTTTAT

ATATATAATAAAAATGTTAATTAATATATCAATTCATATGAATATATAATATTAATAAATAAATTCTTAATAAATAATAATGATAATATATATATATTGT

ATATTAATGTACTATATATATAAATTCATTAAACATTTATATTTGATACCCCCCCAAAATTTTTTCTAAAAATGTGAAGTTTCAATTTAATTAAAATTTT

CTACAATGGTCAAAAACGGCCAAAAAATGGAAAATAAAGTGTGCACAGAAAATGCACATAAAATGCACATTTTTGAAAATTTCCGATTTTTGAAAAACCG

TTTGAACCAAACCTCTGATGTACCTTTTTTCGCCCCCCCTGATTACTAAAATTTATCCTCAATTACTAAGGTTTATCCGCATTTTTGCGACAGGAATTTT

GAGTATAAAATCAATTCCACACTAAATTTTTTTTT-TTTTTTTTGTGTCATTTCTCCAACATGGGCAATTTTTTTTTTAGTCGAAGGACAAATTTTAGTA

AAAAAAGTTTGAAATTAGGTTAAACCCTTGTTTAACCAAAAACTATTACTTTTCAATATTATACCCAATTTTATTTAAAATAAATTTTATTATACTTTTA

TTTATGCAAAAAGATCTTCTTATAAACTTTT

>DMR149u_USA_Laytonsville_MD

AATGAAGTGTCTGACTATAGAGTTACTTTGATAGGGTAAAAAAAGTGAATTTTCACCTTCATTATAATTAACAGAATTAAACTATTTCTTTAAGCTTCAA

AAACTTATGTACATTATATACTAAATTATAAAAAGATAAGCTAATTAAGCTACTGGGCTCATACCCCATCAATAAAGGTTACAATCCTTTTCTTTTTAAT

GTATTATAAGCTTTTATTTTTTAATTCTCTTATAATTGGAACCTTAATTGCTATTTCTTCCTACTCTTGGATAGGAATATGAATAGGATTAGAAATTAAT

CTTCTCTCTATGATCCCCCTCATTAGAGACAACAAAAATATAATAGCCTCAGAAGCTGCTTTAAAATATTTCATTATTCAAACAATAGCATCAACATTGT

TATTATTCTCAATTATTATAATATCAATGAAATTTATATATCAAATAAATTTAATCACTTACTTTAATTTAATTTTTAACACTTCATTGTTCATCAAAAT

AGGAGCAGCCCCATTCCATTTTTGATTCCCCGAAATAATAGAAGGATTAAATTGATTAAATGCCATTATCATACTTACTTGGCAAAAACTAAGGCCCATA

GTATTACTAACGTATTCTAATACAACCTCCATATATCTAATTTTAACAATTATATTTAGAATAATAATCAGAGGAATTATAGGCCTAAATCAAACTAGAT

TACGGAAAATTATAGCTTATTCATCTATCAACCATATTGGATGAATAATCAGTTCAATTATATTAATTGAAATTGTTTGATTTTACTATTTTATTATTTA

TTGCATTATTACTATTAATATCAGAATTATATTTATAAAATTAAATGTTTTTCATATCAATCAATTATATATTTCAATAAATTACCATATTTTACTTAAA

TTATTCTTTGCCTTAAATTTCATGTCTTTAGGAGGATTACCCCCATTTTTAGGGTTTTTCCCTAAATGACTTACAATTCAAACTTTAATCCAAAGAAATA

TGTATTCAATTGCTTTCATTATAATTCTAATAACTTTAATAACACTTTTTTTTTACCTCCGAATTACTTTTTCAATTTTACTACTAAGAAAAGCAGTTTT

AACATTTTACACACAACCAAAAATTAATACTAATTACATTATAGCATTTAATTTTATTACATTATTAAGATTAATTTTCGTTACTTTGATATTCAATTTC

TTATAAATTAAACCTGAAGGATTTAAGTTAAATTAAACTAAGAACCTTCAAAGTTCTAAATAAAGTAAATTCTTTAAGCCTTAGGGCTTAGCCCATCTTT

AAATTTGCAATTTAAAATTCTTTTTGAACTATAAAGCTTGATAAAAGAAACTAATTTCGTATGTAAATTTACAGTTTACCGCCTAAACCTCGGCCATTTT

ATCGAATAAATGGCTATTCTCTACAAATCACAAAGATATCGGAACTTTATACTTTCTATTTGGAAGTTGAGCAGGAATAGTAGGCACTTCCTTAAGTTTA

CTGATTCGTGCTGAATTGGGAAACCCAGGATCTCTAATTGGAGATGATCAAATTTATAACGTAATCGTAACAGCTCATGCTTTCATTATAATTTTTTTTA

TAGTAATACCTATTATAATTGGAGGATTCGGAAACTGATTAGTTCCTTTAATGCTTGGAGCCCCCGACATAGCATTTCCCCGAATAAATAACATAAGATT

TTGACTTTTACCTCCTTCATTAACTCTACTTCTAATAAGAAGATTAGTCGAAAGAGGGGCAGGTACAGGCTGAACAGTTTATCCCCCACTATCAGCCAAT

ATCGCCCATAGAGGAGCATCAGTTGATTTAGCAATTTTTAGACTTCATCTTGCAGGAATTAGCTCAATTTTAGGTGCAGTAAATTTTATTACTACTGTAA

TTAACATACGATCAACAGGAATAACTTTTGATCGAATACCCCTATTTGTTTGATCTGTAGTTTTAACAGCACTTCTTCTGCTATTATCCCTCCCAGTTTT

AGCAGGAGCAATTACTATACTATTAACAGATCGAAATATTAATACAACATTTTTTGATCCCGCAGGTGGAGGAGACCCCATTCTCTACCAACATTTATTT

TGATTTTTTGGTCATCCAGAAGTTTACATTTTAATTTTACCTGGATTTGGTATAATCTCCCATATTATTAGCCAAGAAAGAAGAAAAAAAGAAACATTTG

GAACTTTAGGTATAATTTACGCTATAATAGCTATTGGTTTACTAGGATTTATTGTTTGAGCTCATCATATATTTACAGTAGGTATAGATGTAGACACACG

GGCATATTTTACGTCAGCAACAATAATCATTGCTGTTCCTACAGGAATTAAAATTTTTAGATGATTAGCTACTCTCCATGGATCACAATTAAACTACTCC

CCGTCTCTTTTATGGGCATTAGGATTTGTATTCCTATTTACAGTAGGAGGATTAACAGGAGTAATTCTAGCTAATTCATCAATTGACATTATTTTACATG

ATACTTACTATGTAGTTGCACATTTCCATTATGTCCTTTCCATAGGAGCTGTATTTGCTATTATAGCAGGTTTTGTTCATTGATTCCCTTTATTTACAGG

TTTAACAATAAATTCAAAATTTCTTAAAATTCAATTTTTAACAATATTTATTGGTGTTAATATAACATTCTTCCCTCAACATTTCTTAGGATTAAGAGGA

ATACCTCGACGTTATTCAGATTACCCAGATGCTTACACAACTTGAAATATTATCTCATCTATTGGATCTTTAGTTTCTTTAATTAGTATCTTTATCTTTT

TATTTACTATTTGAGAAAGGCTAATTTCATTACGAAAAAGAATTAGGTCTTTAAGAATATCTACATCAATTGAATGACTCCAACAAATACCCCCTTCAGA

ACATAGTTATTCTGAACTTCCAATGCTTACTAACTTCTAATATGGCAGATTAGTGCAATGGATTTAAACCCCAAATATAAAGATTAAACTTTTTTTAGAA

ATAGCTACTTGAAATACCATTTTACTTCAGGATAGGGCATCCCCATTAATAGAGCAACTCTCATTCTTTCATAACCATGCTCTTCTAATTCTCTTTATAA

TTACCGTTCTAGTAGGTTATTTAATAGGAACTTTATTTTTTAACCAATTTAATTACCGATTTTTATTAGATGGTCAAACTATTGAAATTATTTGAACTAT

TTTACCTGCTGTAACACTAATTTTTATCGCATTACCGTCTTTACGCTTACTTTATCTTCTAGATGAAGTTAATAACCCTTTAGTAACTATCAAAACAATT

GGGCATCAATGATATTGATCATACGAATATAGAGATTTTATAAATTTTGAATTCGATTCCTATATAATTCCTTTAACAGAAATAAAACCTCAAAATTTTC

GTTTATTAGATGTTGATAACCGAGTAATTGTCCCCTTTAACTCCCAAATCCGAATGATAGTAACAGCTGCCGATGTTATTCATTCATGAACTATCCCGGC

TTTTAGTGTAAAAATTGATGCAACACCGGGCCGACTAAATCAAATTAGATTCCTAATTAATCGAACAGGATTATTTTATGGTCAATGCTCAGAAATTTGT

GGAGCAAATCATAGATTTATACCTATTACTGTAGAAAGAATTTCACCTTCATTTTTTACTAAATGAATCTCAAAAATAAATAACCTATCATTAGATGACT

GAAAGTAAGTAATGGTCTCTTAAACCAATTAATAGTAGTTTAACATCTACTTCTGATGGCCAAAAATTTAGTTAAGATATAACATTAGTTTGTCATACTA

AAATAATCATAATTTGATAATTTTTAATTCCACAAATAGCACCTTTAAACTGACTATCTTTATTTTTTTTAATTATTATTATTTTTTTACTTTTTAATGT

ATTAAATTACTTTAGATTCTTACAGCCCTTAAAAACCCAATCTCATAACCCTACAATTAAAAAAATTAATTGAAAATGATAACTAATTTATTTTCATCTT

TTGATCCTAGAACTTCTTTTAATTTAAGATTAAACTGATTAAGAATACTATTAGGGCTAATATTTATCCCCCCAGTATTTTGATTAATTCCTTCACGCCA

TAATTTTCTATGAATTAAAATTATTTTAACATTACACCAAGAATTTAAGGTTTTAATTGGTAATAATAATATTAAAGGAAGAACCTTAATATTTATTTCA

TTATTTTCTATAATTGTTTTCAATAACTTTTTAGGATTATTTCCGTATATTTTTACAGGAACAAGGCATTTAATTATAACATTATCTCTTGCCTTACCTT

TATGAATTAGATTCATATTATACGGGTGAATTAATAATACTATCCACATACTTGCTCATTTAGTTCCCCAAGGAACACCCCCAGCTCTTATGGTATTCAT

AGTAGTAATTGAATCAATTAGAAATATTATTCGTCCTGGTACTTTAGCTGTTCGATTAGCTGCTAATATAATTGCTGGACATTTACTAATAACTTTACTA

GGAAACACAAGATTAAATTTATCAATTTTTATACTAAGTATTCTTATTATCACACAAATTCTTTTATTAATTTTAGAATCTGCTGTTGCGATCATTCAAT

CTTATGTATTTGCTGTATTAAGAACTTTATACTCTAGAGAAATTAATTAATGTCAAGACATAAAAATCACCCTTATCATTTAGTTGATGCAAGACCTTGA

CCTATTTTAGGCGCTTTTAGAGCTATAATTACAATAATTGGAATTATCAAATGATTCCATTTTTATAATAATTCTTTATTTTACTTAGGGACATTAATCA

CAATTTTAATTATAATTCAATGATGACGAGATATCACTCGTGAGGGAACTTTCCAAGGACTTCATACTTATGCTGTAACTATAGGTTTACGTTGAGGAAT

AATTTTATTTATTACATCAGAAGTATTTTTCTTTATTTCTTTTTTTTGAGCCTTTTTTCATAGTAGTTTAACACCCGCTATTGAACTAGGGATACTCTGA

CCACCCAAAGGAATTACCCCATTTAACCCAATTCAAATTCCATTATTAAACACTTTAATTCTTTTAACTTCAGGATTAACTGTAACTTGAGCTCACCATA

GATTAATAGAAAATGACTATAACCAAACAATGCAAGGTCTTGGTTTAACAGTTTTACTAGGAGTATATTTTACTTTATTACAAGGTTACGAATATTTAGA

AGCCCCCTTTACTATAGCAGATTCTGTTTATGGATCAACATTTTTTATTGCTACTGGTTTCCATGGATTACATGTTATTATTGGCACAACCTTTTTAGCT

GTTTGTTTAATACGACATTTTAATAACCATTTTACTTGTATCCATCACTTTGGATTTGAAGCTGCTGCTTGATACTGACATTTTGTTGATGTAGTATGAC

TATTTCTTTATATTTCTATTTACTGATGAGGTAGATATTTATATAGTATAATAATTATAATTGATTTCCAATCAAAAGATCTAAAAAAAATTAGTATAAA

TAATCATTATAATTTGAAATATAGGGTTAATTATTTTTTCTATCTCTTTTATCTTAATTATACTATCTTTTACAATCTCTAAAAAAAGATTTATAGACCG

AGAAAAAGCTTCTCCATTCGAGTGCGGATTTGACCCCAAAAGATCAGCCCGTTTACCCTTTTCTTTGCATTTTTTTTTAATTGCAGTAATTTTCTTAATT

TTTGATGTTGAAATTACTCTTCTTATCCCTTTAATTCTAACAATAAAAATTACTAATATTACTATATATACCTATATTGCTCTTTTCTTTTTAATGATTC

TTTTAATAGGACTTTACCATGAATGAAACCAAGGGGCCTTAAATTGAGCTCTTTAGGGTAATAGTTAAGTATAACATTTAAGTTGCATTTAAAAAGTATT

GATTTTTCAATTTACCTTAAATAAGAAACAATTAATTGTATTTAGTTTCGACCTAAAATTTAGGTGTATGAACACCCTTATTTAAATTAATTGAAACCAA

AAAGAGGTATATCACTGTTAATGATACTAATGAGAAAAACTCCAATTAAGGAAATAAGATATTCAAGGGTAAGCTTCTAACTTAACTCTTTAGCAGTGAA

AGTCTGTTAATATTTCTATTTATATAGTTTAATAAAACATTATTTTTTCATAATAAAATTAGAATAAATTTATTCTTATAAATATTTAAAAGTAAATTTT

ACTTCCCTGATAACTTCACTATCATACTCTATATAAGCTATTTAAATTAAATATATAAAATTATAAAAATTACCCATATTATAATTAAAAGTATAAAAAT

CTTATAATTGTTATTAAATATAAACTGTAAAAATACAGAAGTATTACTAATTTTACTATATAAATTTTGTCTTCCATAATACTCTGATCAGCCTTGATCA

ATAGTTTTATATAACTTACTACCTAATTTAATTGGATAATAATTTAAACCAAATGTTGAAATATAGGGTATATTCCACATAGAAGAAAAAAAAAGTCTTG

AATTTAACAAATAAAAAGATTTTAATCTATCATTTAAAGTAAACTTAGAAAGTTCAAACCCAAACCAAGCCCCAAAAAATGATACAATTAAAGCTATAAT

TTTTATTGTAAAAGGTAAACAAATAAAATAAGGTGTTGGGAATATCAACCATATTAATATTCTTCCCCCAACAATAACTAAAAAAATTAAACCTGATATC

CCTTGAAGTATAATTTTTCTATTATCATTAATTTTACTTAAAGAATAAAAACAAAAATTTCCTACCAAAACATAATAAATTAAACGAAATGTATAACAAA

CAGTTAATCCTGTTGAAAAAAAGAAAATAACATAAATATAGATATTTAAGTATCTTATAGATAAAACCTCTAAAATTAAATCCTTTGAATAAAACCCCGA

TAAAAAGGGTAAACCGCATAATGCTAAATTTGAAATTATAAAATAAGTACAAGTTAAAGGTATAACTTTAATTAACCCCCCTATATACCGAATATCTTGA

CAATTTCTTAATCTGTGAATTATACACCCTGCGCATATAAATAATAAAGCCTTAAATAAAGCGTGAGTTAATAGGTGAAAAAAAGCTAATTGATACTCCC

CTAAAGCTAAAATACTAATTATCAAACCCAATTGTCTTAAAGTAGACAGAGCAATAATTTTTTTTAAATCAAACTCAAAGTTTGCGCCTATTCCTGCTAT

AAATATAGTTATTGTTCCAATAAATAATAAAATTAATATTAAATTACTTGTTAATGCAAAATTAAAACGAATTAATAAGTAAACACCTGCTGTTACTAAG

GTAGAAGAATGAACTAAAGAAGAAACAGGTGTTGGAGCTGCTATTGCCGCAGGCAACCAAGAAGAAAATGGAATTTGAGCTCTTTTAGTTATAGCTGCTA

ATATAATTAATAAAATAATAATATATATTTCTATACTATTTTTATATACATCAATATAAAAAATATAGTTAAATCCCCCAAAATTTATTATTCATGCAAT

TGCTATTAATAAAGCAACATCTCCAATACGGTTAGTTAAAGCTGTAATTATCCCTGCATTATAAGATTTAATATTTTGATAATAAATAACTAAACAATAA

GAAACTAACCCTAACCCATCTCATCCTAATAAAATCCTAATTAAATTAGGAGAAATAATCAATAACATTATAGATAAAACAAATATAGAAACTAATATGA

TAAATCGATGCAAATAAATATCTCCTTCTATATACTCTTCACTATAGTAAATTACTATAGAAGAAATAAATAAAACAAAACTCATAAATAATAATGACAT

CCAATCAAGTAAAATGGTTATAATAATTCTACAAGAATTAATCCTTAATATTTCATATTCTAATATTAGTCTATAATCTAAAATTATAAAATTTAAGCTT

AATAAAAATCTTAATACTCTAAAAAATAAAAACGTTACAAAATAAATTAAACAAATAGAAATAATTTAAAGTAAATTTTACATCTTTGATACCACAAATC

AACATTTTTTATTAAACTATTTAAATTACAATCATAAAACTAAAAATTCTCTCTTTAAAATTAAGATATTCAAGGGTAACCAATGAAGTAATAATAATAA

ATACTCACGAACAAACCCTCTTGAAAAAGAATATAAATTTCTAACTAACTTGCCGTGTTGACTATAAGAATATAAATATAAAGAATACGCAGCTCTAAAA

AAAGATATTAAAGATAAAAAAACCATTGTTCAACTCCTTCATCTAACTAATCTATTAATTAAAATAATCTCACCCAGTAAGTTTAATGAAGGAGGAGCCG

CTATATTACAACAACTAAATAAAAATCATCATATTCTTATTATTGGTATTAAATTGATTAGCCCCTTATTTAAATAAATACTTCGGCTATTTAACCGTTC

ATAAGAAATATTTGCTAAACAAAATAATCCTGAAGAACATAAACCATGGGCAATTATTATAACTAAAGCCCCTCTTATACCCCAATAACTTAAAGTTAAA

ATCCCTCTTAATACTAAACCTATATGCGCTACAGAAGAATAAGCAATTAAAGCTTTAATATCAACTTGACGTAAACATATTAATGAAACAAAAAATCCCC

CTACTATTCTAATTGTAATAAAAATATAGTTTACTTGTAAACCTACAGTTAAAAAAATATTTATTAAACGTATTAATCCATACCCTCCTAATTTTAATAT

AACTCCAGCTAAAATTATAGAGCCAGCAACTGGAGCTTCAACATGGGCTTTAGGGAGCCAAAGATGAACAAAAAATATAGGCATTTTAATAAAAAACACT

ATATTTATACATATAAATAACAAGAATCTTTTTACATCATAACATAAAAAGAAAAAATCTAAAGAATGAAATTTTTCATAATAATAAAAAATCCTAATTA

TTATAGGTAATGAAGCAAATAATGTATAAAATAACAAGTAAACCCCTGCTTGCAAACGCTCAGGTTGATACCCCCAACCAATAATTAATAAAAGAGTGGG

AATTAAGCTAAACTCAAAAAACAAATAAAAAATAAATAAATTTAAAGAACTAAATGTTATAACTAAAGATAATAATAAAATAATTATTACTAATAAAAAT

AAATTATAAAAATAGTTTTTTCTATAAATACTTTCAGAAGCTAATAATATTAAAGAACAAATCCACAAACTTAATAAAATTATTATAAAAGATAAAAGGT

CATACCCTATAAAATAAGAAATATTTATATACAAATAATTAAAACTAAATCTTAACCCAAATATAAATGTAATAAAAAAATATATATATTGATTAAATCA

GTATCTCTTTTTAATACAACTTAAAGGTACCAATATTAATATTATAAAAATAAACTTTATCATAATACATTAAAAGTTTGAAAATAATCATTTCCATGTG

TTCGTATTATAGAAACTAATAACGATAAACCTAAAGCTCCTTCACAAACTCTTATAGTTAAAAATACTATACCAAAATAAAATTCAAAATTAAAATATAT

TAAAAATAGATATAAATTAAAATATAACCCTAAAATAATATACTCTAACCTTAATAATATTAAAAGTAAATGTTTACGTTTAATACAAAAAGAAACTAAT

CCTGTAAAATATATAATCACTGAAAATAACATACAAAAAATTAACATTAGTTTTAATAATTTAATAAAAATACTGGTCTTGTAAATCAGAAATAAGGATT

TTCTTTTAAAACTTCAGAGAAAGAGTAAACCTCTATCATTAATCTCCAAAATTAATATTTTAAATAAACTATTCTCTGTATAATCTTATTACTAATAACA

TTATCTTTTATTAGTTCAATAACTTTTATATTTTTAAGTCACCCTTTATCTATAGGGCTTATTTTATTAATACAAACTATTATTATAGCTTTAACTATAG

GTTTTTTTAATATTAATTTCTGATATTCTTATATTTTATTCCTTATTATAATTGGAGGAATATTAGTTTTATTTATTTATATAACAAGAGTAGCTTCAAA

TGAAAAATTTTCATTTTCTATTAAAATTACATTAATAATTAGAATTATAACTTTAGGATTTTTATTCAGTATTGCTATAATAGACCCCTACTTTTCAGAC

ATCAACTCAATTTACACAGAAAACTTAGATAATTATAAAGAATATAATATATCATTTAGAAAATATTTAAGTTATCCTAATATTATCATTATATACATAA

TAATTATTTATTTATTAATTACATTAATTGCAGTTGTAAAAATTACTCAAATTGAAAAAGGACCTTTACGTCAAACTAACTAATGAAAACACCATTACGA

AAAGCTTCCCCCCTATTAAAAATTATTAATAATAGAATTATTGATTTACCTACACCATCTAATATTTCTGCTTGATGAAATTTCGGATCCTTATTAGGAC

TCTGTCTTTTTATTCAAATTATCACTGGAATTTTCTTAGCTATACATTTTACAGCCCATATTGACATAGCATTTAATAGAGTAATTCATATTTGTCGAGA

TGTAAATTATGGCTGATTATTACGAACAATTCATGCTAACGGGGCTTCTTTTTTCTTTATTTGTATTTATCTACATATTGGCCGAGGAATATATTATAGA

AGCTATAATTTGCATTTAACATGAACTATTGGAGTAATCATCCTTTTTATAGTAATAGCTACAGCATTCTTAGGGTATGTATTACCTTGAGGGCAAATAT

CTTTTTGAGGGGCTACTGTTATTACCAATCTTCTCTCTGCTATTCCTTATCTAGGAAATATGATTGTCCAATGATTATGGGGGGGATTTGCAGTAGATAA

CGCCACTTTAACTCGATTTTTTACATTACATTTTATTCTTCCTTTTATTATTTTAGCTTTAATAATTATTCATTTATTATTTCTTCATCAAACTGGTTCC

AATAACCCTTTAGGATTAAATAGAAATATCGATAAAGTACCTTTCCATCCATATTTTACTTATAAAGATACTTTTGGATTCATTATAATAACTATATTAT

TAATATTTCTAGTTTTAATTAATCCCTATCTTTTAGGAGATCCAGAAAACTTTACACCCGCTAACCCTTTAGTAACCCCAGTCCATATCCAACCAGAATG

ATACTTTTTATTCGCATATGCTATTTTACGATCAATTCCTAATAAATTAGGTGGAGTAATTGCTTTAGTTATATCAATTGCTATTTTATTAATTATACCT

TTAATTAACAAAAAAAAATTTAGTAGAACTCAATTTTACCCATTAAATAAAATTTTATTTTGATCTTTTGTCTCTATTGTAATCTTATTAACATGAATCG

GAGCTCGCCCAGTGGAAGATCCATACATTTTAACAGGTCAAATTTTAACAATTATATATTTTAGTTATTATTTCTTAAACCCCTTAATTCATAAAATATG

AGATTATATTATTTTTAAAACTTAGTTAATGAACTTGTTAAAGTGTATATTTTGAAAATATAAAAAAGAGTTTATTCTCTATTAACTTTACTAAATTTTA

TTCACTAAATAAAATAAGAAAAGATAACCAACTTTAAACCTAAAAAAAAAAATAAAAAATTTAAAGAAACTGGTAAATAACTTTTTCAACATATATATAT

TAACTTATCATAACGGTACCGAGGTAAAGTACCTCGAACTCAAATCCAAATAAATGCTATAAAAGTTAACTTCAAAAAAAAAAATAAAGAAAAAATATCT

CCCCCTATAAATAACAACACACATAATATACTTATAAATAAAATTCTAGCATACTCAGCCAAAAAGATTAAAGCAAATCCCCCTCTTCTGTACTCTACAT

TAAACCCAGAAACTAACTCTGATTCTCCTTCTGCAAAATCAAAAGGAGTCCGATTAGTCTCAGCTAAACTTGAAGATAATCATATTATTCTTAAAGGTAA

GCACAGAAAAAAAAATCATACTATTTCTTGATATTTTATAAAATCTATTATATTTAAATTTAAAATTAACAATAAAAAAGATAATAAAATTAATGATAAA

CTTACTTCGTATGAAATTGTTTGAGCTACTGATCGAATACCCCCTAATAAAGCATAATTAGAATTTGACGATCAACCAGAAATTATGATAGTATAGACCC

TTAACCTAGAACAACATAAAAAATATAAAATACCTAAATTAAACCTAAATATAAATGTCAAAAAAGGTATACATATCCATAATAAAAGGGCTAAAAATAA

ATTAAAAACTGGAGATATATAATAAATAAAAAAATTTGATATTAAAGGATAAGTTTGTTCTTTAGAAAATAACTTAATAGCATCACTAAAAGGTTGAGGA

ATACCTATAAATCCAACTTTATTGGGCCCTTTACGAATTTGGATATACCCTAAAACTTTACGCTCTATTAATGTTAAAAAAGCTACACCAATTAAAACAC

AAATAATTAAAACTAAACTCGTAAATAATAAAAGAAATAAATCTTGTAATATAATGTATTACTTGTGTTAAACACATATTTAAATTCTAAATTTAAAGCA

CTAATCTGCCAAAGTAATATTCATATTCAAATTATATTAAATTTTAAAGGTATCTGATCCTTTCGTACTAAAATACCTATGTTTTTTAAAGATAGAAACC

AACCTGGCTCACGCCGGTTTAAACTCAGATCATGTAAAATTTTAAAGGTCGAACAGACCTAACCTTTTAGCCCCTACACCAAAAGTTAATTTTAATCCAA

CATCGAGGTCGCAAACTTTTTTTTCGATAAGAACTCTAAAAAAAA-TTACGCTGTTATCCCTAAGGTAATTTAATCTTGTAATCATTAAAAATGGATCAT

TCAATCATAAATTAATGTTTTTAAATAAAAAAAGTTTAATCAATTTTTCTGCTGCCCCAGCAAAATAGTTTAAATTATTAAATATATAAATATGCTAAAA

TTAAATAATAATTTAAACTATAAAACTCTATAGGGTCTTCTCGTCTTTTAAAATTATATAAGCTTTTTTACTTATAAATAAAATTCTATATTCAATTAAA

TTGAGACAGTTACTTTCTCGTCCAACCGTTCATTCCAGCTTTCAATTAAAAAACTAATGATTATGCTACCTTTGCACGGTCAAATTACCGCGGCCATTCA

AATCCTCATTGGGCAGGTCAGACTTTAAATTATAATCAAAAAGACATGTTTTTAATAAACAGGCGAAAAGTGTATTTGCCGAGTTCCTTAATTTAACCTT

GAAGTTTTAATTTAATTACTAAATTAAAATATATACTAATTTTATCATTATTCTATATAAACCAATATTACATATATTATCTTAATAAACTACTTAAAAA

TAATATAAATCTTATTCTAACAAAAATTATTTATAACAAACTAAAGATTAACACTTCCAATTCTACTAATTTTTATTCAAAATATACATTTTTAACATTT

TATTTTAAAGCTTATCCCCTAAAATATTACTTTTTATATATAAAATACTAAATAATTAATATAATACAATAAAAAAACTAAATTAAATTTATTTCTTAAG

AAACTAGATATCTTAAAAAACGTATAACGTTTCATTTCTAATATAATATTTTAAAAATTTATGCCACAATTAAATTTATATTATATTAGCTCTTTATAAT

TCGAGAACACTAAATAATTAAATTATTTTAATAAACCCTGATACACAAGGTACAAAAAATTAATTTTTCTTTTTAAAAAATAAATCTCTATATATTTATA

TTATCTATCTCTATACAAATTAACTATAATAAAATTTTTATATTCTAAAATATACTAATATCAAAAATATTTTTTTTATATATATATATA------TTAT

AAATTTTTCCTTTCAAATTAAATTGATTTTCACAACTAACTTTTTAATGTAAATAAAATGCTTTATTACAAGCTCTAATTTGCCATTCCAGGTACACTTT

CCAGTACACCTACTATGTTACGACTTATCCCTCTTTAGAGAGGGAGCGACGGGCGATATGTACATATTCTAGAGCTATACTCATATAATTAAACTAAACT

ATATTACTTTCAAATCCACTTTATAAAATAATGTTAATTATTTTAACCATCTAAATAATTTTATTGTAACCCATCTCTCCTTATCTATACGCTGTATCTT

GATCTGATTTTTTTTATACTTATAAATTTTGAACATTCCAAATTCTTTAAAAACATTCAACCTACGACGATATACAAACCTTTAAAATAAGTACGATTAA

TCGTGGACCATCAATTATAGGACAGGTTCCTCTGAGTAGACTAAAATACCGCCAAATTCTTAAAATTTCAAGAACATAACTACTACTATTCAAGCATCTA

AAATTTGCATTTTTAATAATAGGGTATCTAATCCTAGTTTTTTATAAAAATCTCATAAACTCATTTTTCACATTTAAAAAATTAATTATATTTACTAATT

TCACCTAATAAATACAACATAAATTAATAATAAAATAACTTATTATATACTGAACAAATTTAATTGTATTGTTTGTGTAACCGCAACTGCTGGCACAAAC

TTGGTCAATACTATTATAAATTCCTAAATCAAAATTTCTTTTAAATTTAATCTTCACTATTGCAATTCTTTAATTAAATATATAGAATAATTTATCTTTT

TAAAATAAATTCATTAAACACTAAAATTTACATATAAAATAATTTAAAAATTAAAATCTCAAGCTAGAATAAAACTTTATTTTTTTTTCAATATACATAA

ATTATATAAAATAAAAGTACCCCCC-TACTATTTTAATTAAATGAATTAATTTTTAATGCCTTAAATTTATTTCCAATTTATTTTTATTATATAGTTTTT

TATTTAAAAATAAAACTTTATACCTAAAATTATATACATAAATTATATTTAAAATTTATAATTTAAAGAACTATAAAAATTAAATTAAAATTTAAAATCT

ATCTAATTTATAGATAAACAAATTAAATTAAACTAACTTACCTGATAAATTTAACTATATACGCGCAGGTATATTAATTATTTAATTATAATATAATACA

TTTGTTTTATTAAATAATTATTAAGCAAAATAGCATTAATTTATTATATAAAAGATCTAGATACTATTAATTTAAAGTAAATTTTTTTTTTTTTTGTAGT

ATAATTTATACTAATATACGGCATTTAATATCTTCTATATAAATGTTTATATTATTTATATTTATCTATTTATAATTCTTAATAATAATTTAGATAATTA

TTAATTTATATATTAAATATTTATATATATATATATAATAATAAGGATATATATATATATATGTATATAGATAAATTAAATTTAGTTATATATAAAAAAA

TTAATATTAATTTTACTTTTAATTTTTTCTTTAATTTAAACTATTGGATATTCAGATTCTAATATATTATAATATATCAACCATTAATGAATAACTTTAT

ATATATAATAAAAATGTTAATTAATATATCAATTCATATGAATATATAATATTAATAAATAAATTCTTAATAAATAATAATGATAATATATATATATTGT

ATATTAATGTACTATATATATAAATTCATTAAACATTTATATTTGATACCCCCCCAAAATTTTTTCTAAAAATGTGAAGTTTCAATTTAATTAAAATTTT

CTACAATGGTCAAAAACGGCCAAAAAATGGAAAATAAAATGTGCACAGAAAATGCACATAAAATGCACATTTTTGAAAATTTCCGATTTTTGAAAAACCG

TTTGAACCAAACCTCTGATGTACCTTTTTTCGCCCCCCCTGATTACTAAAATTTATCCTCAATTACTAAGGTTTATCCGCATTTTTGCGACAGGAATTTT

GAGTATAAAATCAATTCCACACTAAATTTTTTTTT-TTTTTTTTGTGTCATTTCTCCAACATGGGCAATTTTTTTTTTAGTCGAAGGACAAATTTTAGTA

AAAAAAGTTTGAAATTAGGTTAAACCCTTGTTTAACCAAAAACTATTACTTTTCAATATTATACCCAATTTTATTTAAAATAAATTTTATTACACTTTTA

TTTATGCAAAAAGATCTTCTTATAAACTTTT

>DMR152u_USA_Auburn_AL

AATGAAGTGTCTGACTATAGAGTTACTTTGATAGGGTAAAAAAAGTGAATTTTCACCTTCATTATAATTAACAGAATTAAACTATTTCTTTAAGCTTCAA

AAACTTATGTACATTATATACTAAATTATAAAAAGATAAGCTAATTAAGCTACTGGGCTCATACCCCATCAATAAAGGTTACAATCCTTTTCTTTTTAAT

GTATTATAAGCTTTTATTTTTTAATTCTCTTATAATTGGAACCTTAATTGCTATTTCTTCCTACTCTTGGATAGGAATATGAATAGGATTAGAAATTAAT

CTTCTCTCTATGATCCCCCTCATTAGAGACAACAAAAATATAATAGCCTCAGAAGCTGCTTTAAAATATTTCATTATTCAAACAATAGCATCAACATTGT

TATTATTCTCAATTATTATAATATCAATGAAATTTATATATCAAATAAATTTAATCACTTACTTTAATTTAATTTTTAACACTTCATTGTTCATCAAAAT

AGGAGCAGCCCCATTCCATTTTTGATTCCCCGAAATAATAGAAGGATTAAATTGATTAAATGCCATTATCATACTTACTTGACAAAAACTAAGGCCCATA

GTATTACTAACGTATTCTAATACAACCTCCATATATCTAATTTTAACAATTATATTTAGAATAATAATCAGAGGAATTATAGGCCTAAATCAAACTAGAT

TACGGAAAATTATAGCTTATTCATCTATCAACCATATTGGATGAATAATCAGTTCAATTATATTAATTGAAATTGTTTGATTTTACTATTTTATTATTTA

TTGCATTATTACTATTAATATCAGAATTATATTTATAAAATTAAATGTTTTTCATATCAATCAATTATATATTTCAATAAATTACCATATTTTACTTAAA

TTATTCTTTGCCTTAAATTTCATATCTTTAGGAGGATTACCCCCATTTTTAGGATTTTTCCCTAAATGACTTACAATTCAAACTTTAATCCAAAGAAATA

TGTACTCAATTGCTTTCATTATAATTCTAATAACTTTAATAACACTTTTTTTTTACCTTCGAATTACTTTTTCAATTTTACTACTAAGAAAATCAGTTTT

AACATTTTACACACAACCAAAAATTGATACTAATTACATTATAGCATTTAATTTTATTACATTATTAAGATTAATTTTCGTTACTTTGATATTCAATTTC

TTATAAATTAAACCTGAAGGATTTAAGTTAAATTAAACTAAGAACCTTCAAAGTTCTAAATAAAGTAAATTCTTTAAGCCTTAGGGCTTAGCCCATCTTT

AAATTTGCAATTTAAAATTCTTTTTGAACTATAAAGCTTGATAAAAGAAACTAATTTCGTATGTAAATTTACAGTTTACCGCCTAAACCTCGGCCATTTT

ATCGAATAAATGGCTATTCTCTACAAATCACAAAGATATCGGAACTTTATACTTTCTATTTGGAAGTTGAGCAGGAATAGTAGGTACTTCCTTAAGTTTA

CTGATTCGTGCTGAATTGGGAAACCCAGGATCTCTAATTGGAGATGATCAAATTTATAACGTAATCGTAACAGCTCATGCTTTCATTATAATTTTTTTTA

TAGTAATACCTATTATAATTGGAGGATTCGGAAACTGATTAGTTCCTTTAATGCTTGGAGCCCCCGACATAGCATTTCCCCGAATAAATAACATAAGATT

TTGACTTTTACCTCCTTCATTAACTCTACTTCTAATAAGAAGATTAGTCGAAAGAGGAGCAGGTACAGGCTGAACAGTTTATCCCCCACTATCAGCCAAT

ATCGCCCATAGAGGAGCATCAGTTGATTTAGCAATTTTTAGACTTCATCTTGCAGGAATTAGCTCAATTTTAGGTGCAGTAAATTTTATTACTACTGTAA

TTAACATACGATCAACAGGAATAACTTTTGATCGAATACCCCTATTTGTTTGATCTGTAGTTTTAACAGCACTTCTTCTGCTATTATCCCTCCCAGTTTT

AGCAGGAGCAATTACTATACTATTAACAGATCGAAATATTAATACAACATTTTTTGATCCCGCAGGTGGAGGAGACCCCATTCTCTACCAACATTTATTT

TGATTTTTTGGTCATCCAGAAGTTTACATTTTAATTTTACCTGGATTTGGTATAATCTCCCATATTATTAGCCAAGAAAGAAGAAAAAAAGAAACATTTG

GAACTTTAGGTATAATTTACGCTATAATAGCTATTGGTTTACTAGGATTTATTGTTTGAGCTCATCATATATTTACAGTAGGTATAGATGTAGACACACG

AGCATATTTTACATCAGCAACAATAATCATTGCTGTTCCTACAGGAATTAAAATTTTTAGATGATTAGCTACTCTCCATGGATCACAATTAAACTACTCC

CCGTCTCTTTTATGGGCATTAGGATTTGTATTCCTATTTACAGTAGGAGGATTAACAGGAGTAATTCTAGCTAATTCATCAATTGACATTATTTTACATG

ATACTTACTATGTAGTTGCACATTTCCATTATGTCCTTTCCATAGGAGCTGTATTTGCTATTATAGCAGGTTTTGTTCATTGATTCCCTTTATTTACAGG

TTTAACAATAAATTCAAAATTTCTTAAAATTCAATTTTTAACAATATTTATTGGTGTCAATATAACATTCTTCCCTCAACATTTCTTAGGATTAAGAGGA

ATACCTCGACGTTATTCAGATTACCCAGATGCTTACACAACTTGAAATATTATCTCATCTATTGGATCTTTAGTTTCTTTAATTAGTATTTTTATCTTTT

TATTTACTATTTGAGAAAGGCTAATTTCATTACGAAAAAGAATTAGGTCTTTAAGAATATCTACATCAATTGAATGACTCCAACAAATACCCCCTTCAGA

ACATAGTTATTCTGAACTTCCAATGCTTACTAACTTCTAATATGGCAGATTAGTGCAATGGATTTAAACCCCAAATATAAAGATTAAACTTTTTTTAGAA

ATAGCTACTTGAAACACCATTTTACTTCAGGATAGGGCATCCCCATTAATAGAACAACTCTCATTCTTTCATAACCATGCTCTTCTAATTCTCTTTATAA

TTACCGTTCTAGTAGGTTATTTAATAGGAACTTTATTTTTTAACCAATTTAATTACCGATTTTTATTAGATGGTCAAACTATTGAAATTATTTGAACTAT

TTTACCTGCTGTAACACTAATTTTTATCGCATTACCGTCTTTACGCTTACTTTATCTTCTAGATGAAGTTAATAATCCTTTAGTAACTATCAAAACAATT

GGGCATCAATGATATTGATCATACGAATATAGAGATTTTATAAATTTTGAATTCGATTCCTATATAATTCCTTTGACAGAAATAAAACCTCAAAATTTTC

GTTTATTAGATGTTGATAACCGAGTAATTGTCCCCTTTAACTCCCAAATTCGAATGATAGTAACAGCTGCCGATGTTATTCATTCATGAACTATCCCGGC

TTTTAGTGTAAAAATTGATGCAACACCGGGCCGACTAAATCAAATTAGATTTCTAATTAATCGAACAGGATTATTTTATGGTCAATGCTCAGAAATTTGT

GGAGCAAATCATAGATTTATACCTATTACTGTAGAAAGAATTTCACCTTCATTTTTTACTAAATGAATCTCAAAAATAAATAACCTATCATTAGATGACT

GAAAGTAAGTAATGGTCTCTTAAACCAATTAATAGTAGTTTAACATCTACTTCTGATGGCCAAAAATTTAGTTAAGATATAACATTAGTTTGTCATACTA

AAATAATCATAATTTGATAATTTTTAATTCCACAAATAGCACCTTTAAACTGACTATCTTTATTTTTTTTAATTATTATTATTTTTTTACTTTTTAATGT

ATTAAATTACTTTAGATTCTTACAGCCCTTAAAAACCCAATCTCATAGCCCTACAATTAAAAAAATTAATTGAAAATGATAACTAATTTATTTTCATCTT

TTGATCCTAGAACTTCTTTTAATTTAAGATTAAACTGATTAAGAATACTATTAGGGCTAATATTTATCCCCCCAATATTTTGATTAGTTCCTTCACGCCA

TAATTTTCTATGAATTAAAATTATTTTAACATTACACCAAGAATTTAAGGTTTTAATTGGTAATAATAATATTAAAGGAAGAACCTTAATATTTATTTCA

TTATTTTCTATAATTGTTTTCAATAACTTTTTAGGATTATTTCCGTATATTTTTACAGGAACAAGACATTTAATTATAACATTATCTCTTGCCTTACCTT

TATGAATTAGATTCATATTATACGGGTGAATTAATAACACTATCCACATACTTGCTCATTTAGTTCCCCAAGGAACACCCCCAGCTCTTATGGCATTCAT

AGTAGTAATTGAATCAATTAGAAATATTATTCGTCCTGGTACTTTAGCTGTTCGATTAGCTGCTAATATAATTGCTGGACATTTACTAATAACTTTACTA

GGAAACACAGGATTAAATTTATCAATTTTTATACTAAGTATTCTTATTATCACACAAATTCTTTTATTAATTTTAGAATCTGCTGTTGCGATCATTCAAT

CTTATGTATTTGCTGTATTAAGAACTTTATACTCTAGAGAAATTAATTAATGTCAAGACATAAAAATCACCCTTATCATTTAGTTGATGCAAGACCTTGA

CCTATTTTAGGTGCTTTTAGAGCTATAATTACAATAATTGGAATTATCAAATGATTCCATTTTTATAATAATTCTTTATTTTACTTAGGGACATTAATCA

CAATTTTAATTATAATTCAATGATGACGAGATATCACTCGTGAGGGAACTTTCCAAGGACTTCATACTTACGCTGTAACTATAGGTTTACGTTGAGGAAT

AATTTTATTTATTACATCAGAAGTATTTTTCTTTATTTCTTTTTTTTGAGCCTTTTTTCATAGTAGCTTAACACCCGCTATTGAACTAGGGATACTCTGA

CCACCCAAAGGAATTACCCCATTTAACCCAATTCAAATTCCATTATTAAACACTTTAATTCTTTTAACTTCAGGATTAACTGTAACTTGAGCTCATCATA

GATTAATAGAAAATGACTATAACCAAACAATGCAAGGTCTTGGTTTAACAGTTTTACTAGGAGTATATTTTACTTTATTACAAGGTTACGAATATTTAGA

AGCCCCCTTTACTATAGCAGATTCTGTTTATGGATCAACATTTTTTATTGCTACTGGTTTCCATGGATTACATGTTATTATTGGCACAACCTTTTTAGCT

GTTTGTTTAATACGACATTTTAATAACCATTTTACTTGTATCCATCACTTTGGATTTGAAGCTGCTGCTTGATACTGACATTTTGTTGATGTAGTATGAC

TATTTCTTTATATTTCTATTTACTGATGAGGTAGATATTTATATAGTATAATAATTATAATTGATTTCCAATCAAAAGATCTAAAAAAAATTAGTATAAA

TAATCATTATAATTTGAAATATAGGGTTAATTATTTTTTCTATCTCTTTTATCTTAATTATACTATCTTTTACAATCTCTAAAAAAAGATTTATAGACCG

AGAAAAAGCTTCTCCATTCGAGTGCGGATTTGACCCCAAAAGATCAGCCCGTTTACCCTTTTCTTTGCATTTTTTTTTAATTGCAGTAATTTTCTTAATT

TTTGATGTTGAAATTACTCTTCTTATCCCTTTAATTCTAACAATAAAAATTACTAATATTACTATATATACCTATATTGCTCTTTTCTTTTTAATGATTC

TTTTAATAGGACTTTACCATGAATGAAACCAGGGGGCCTTAAATTGAGCTCTTTAGGGTAATAGTTAAGTATAACATTTAAGTTGCATTTAAAAAGTATT

GATTTTTCAATTTACCTTAAATAAGAAACAATTAATTGTATTTAGTTTCGACCTAAAATTTAGGTGTATGAACACCCTTATTTAAATTAATTGAAACCAA

AAAGAGGTATATCACTGTTAATGATACTAATGAGAAAAACTCCAATTAAGGAAATAAGATACTCAAGAGTAAGCTTCTAACTTAACTCTTTAGCAGTGAA

AGTCTGTTAATATTTCTATTTATATAGTTTAATAAAACATTATTTTTTCATAATAAAATTAGAATAAATTTATTCTTATAAATATTTAAAAGTAAATTTT

ACTTCCCTGATAACTTCACTATCATACTCTATATAAGCTATTTAAATTAAATATATAAAATTATAAAAATTACCCATATTATAATTAAAAGTATAAAAAT

CTTATAATTGTTATTAAATATAAACTGTAAAAATACAGAAGTATTACTAATTTTACTATATAAATTTTGTCTTCCATAATACTCTGATCAGCCTTGATCA

ATAGTTTTATATAACTTACTACCTAATTTAATTGGATAATAATTTAAACCAAATGTTGAAATATAGGGTATATTCCACATAGAAGAAAAAAAAAGTCTTG

AATTTAACAAATAAAAAGATTTTAATCTATCATTTAAAGTAAACTTAGAAAGTTCAAACCCAAACCAAGCCCCAAAAAATGATACAATTAAAGCTATAAT

TTTTATTGTAAAAGGTAAACAAATAAAATAAGGTGTTGGGAATATCAACCATATTAATATTCTTCCCCCAACAATAACTAAAAAAATTAAACCTGATATC
[truncated: 1,008,444 more chars]
